# Supplementary material for: Changing trends in ophthalmological emergencies during the COVID-19 pandemic
Source: PLoS One. 2022 May 27;17(5):e0268975. doi: 10.1371/journal.pone.0268975 (PMC9140243; doi:10.1371/journal.pone.0268975)
Supplement: S1 Appendix — (DOCX) [file pone.0268975.s001.docx]

**Appendix**

**Table 1. Number of visits to the ophthalmological emergency department since 2015.**

| Year | Jan | Feb | Mar | Apr | May | Jun | Jul | Aug | Sep | Oct | Nov | Dec |
| --- | --- | --- | --- | --- | --- | --- | --- | --- | --- | --- | --- | --- |
| *2015* | 1136 | 1119 | 1287 | 1232 | 1434 | 1458 | 1203 | 1026 | 1193 | 1228 | 1167 | 1167 |
| *2016* | 1130 | 1175 | 1225 | 1337 | 1369 | 1356 | 1184 | 1006 | 992 | 1050 | 1082 | 1081 |
| *2017* | 946 | 863 | 1112 | 928 | 1571 | 1512 | 1442 | 1284 | 1364 | 1390 | 1343 | 1431 |
| *2018* | 1292 | 1274 | 1423 | 1417 | 1469 | 1440 | 1452 | 1288 | 1378 | 1472 | 1446 | 1498 |
| *2019* | 1496 | 1449 | 1681 | 1480 | 1577 | 1527 | 1536 | 1354 | 1414 | 1463 | 1393 | 1372 |
| *2020* | 1476 | 1381 | 501 | 302 | 807 | 1030 | 989 | 889 | 761 | 801 | 838 | 988 |
| 2021 | 674 | 843 |  |  |  |  |  |  |  |  |  |  |

**Table 2. Number of diagnoses categorized by group in the representative month of the pandemic outbreak (April 2020) compared with the same month of the year before.** The visits are presented as the number (percentage). The χ^2^ test was used to compare frequencies and p < 0.05 were considered significant.

| ***Groups*** | ***April 2019*** | ***April 2020*** | ***p*** |
| --- | --- | --- | --- |
| *Conjunctiva* | 415 (28.0) | 54 (17.9) | ***< 0.001*** |
| *Cornea* | 495 (33.4) | 101 (33.4) | 0.995 |
| *Lens* | 38 (2.6) | 5 (1.7) | 0.347 |
| *Glaucoma* | 10 (0.7) | 5 (1.7) | 0.089 |
| *Orbit and eyelids* | 162 (10.9) | 32 (10.6) | 0.862 |
| *Lacrimal system* | 16 (1.1) | 2 (0.7) | 0.508 |
| *Ocular inflammation* | 57 (3.8) | 19 (6.3) | 0.055 |
| *Vitreous* | 111 (7.5) | 35 (11.6) | ***0.018*** |
| *Retina and choroid* | 51 (3.4) | 22 (7.4) | ***0.002*** |
| *Trauma* | 48 (3.2) | 13 (4.3) | 0.354 |
| *Neuro-ophthalmology* | 24 (1.6) | 8 (2.6) | 0.220 |
| *Others* | 54 (3.6) | 6 (2.0) | 0.145 |
| *Total* | 1,481 | 302 |  |

**Table 3.** **Leading diagnoses and their International Statistical Classification of Diseases and Related Health Problems, Tenth Revision (ICD-10) codes in the representative month of the COVID-19 pandemic outbreak (April 2020) compared with the same month of the year before.** The visits are presented as the number (percentage).

| ***Diagnosis April 2019*** | ***n*** | ***Diagnosis April 2020*** | ***n*** |
| --- | --- | --- | --- |
| *Conjunctivitis (H10.3)* | 325 (21.9) | *Keratitis* (H16.9) | 48 (15.9) |
| *Keratitis (H16.9)* | 235 (15.9) | *Conjunctivitis* (H10.3) | 35 (11.6) |
| *Corneal ulcer (H16.0)* | 123 (8.3) | *Posterior vitreous detachment* (H43.81) | 30 (9.9) |
| *Corneal foreign body (T15.0)* | 102 (6.9) | *Corneal ulcer* (H16.0) | 30 (9.9) |
| *Posterior vitreous detachment (H43.81)* | 99 (6.7) | *Uveitis* (H20) | 17 (5.6) |
| *Hyposphagma (H 11.3)* | 74 (5.0) | *Hordeolum* (H00.02) | 15 (5.0) |
| *Blepharitis (H01.00)* | 67 (4.5) | *Corneal foreign body* (T15.0) | 12 (4.0) |
| *Hordeolum (H00.02)* | 44 (3.0) | *Hyposphagma* (H 11.3) | 12 (4.0) |
| *Ocular trauma (S05.9)* | 41 (2.8) | *Blepharitis* (H01.00) | 6 (2.0) |
| *No pathology (H53)* | 37 (2.5) | *Ocular trauma (S05.9)* | 6 (1.7) |
| *Uveitis (H20)* | 36 (2.4) | *Cutaneous herpes infection (B00.1)* | 5 (1.7) |

**Table 4.** **Number of diagnoses categorized by group in six representative months after the COVID-19 pandemic outbreak (September 2020–February 2021) compared with the same period of the year before.** The visits are presented as the number (percentage). The χ^2^ test was used to compare frequencies and p < 0.05 was considered significant.

| ***Groups*** | ***Non pandemic*** | ***Pandemic*** | ***p*** |
| --- | --- | --- | --- |
| *Conjunctiva* | 1,967 (23.1) | 822 (16.8) | ***<0.001*** |
| *Cornea* | 2,716 (32.0) | 1,824 (37.2) | ***<0.001*** |
| *Lens* | 165 (1.9) | 120 (2.4) | 0.05 |
| *Glaucoma* | 74 (0.9) | 55 (1.1) | 0.123 |
| *Orbit and eyelids* | 1,102 (13.0) | 635 (13.0) | 0.980 |
| *Lacrimal system* | 89 (1.0) | 40 (0.8) | 0.186 |
| *Ocular inflammation* | 349 (4.1) | 267 (5.4) | ***<0.001*** |
| *Vitreous* | 541 (6.4) | 388 (7.9) | ***0.001*** |
| *Retina and choroid* | 412 (4.8) | 235 (4.8) | 0.887 |
| *Trauma* | 486 (5.7) | 268 (5.5) | 0.541 |
| *Neuro-ophthalmology* | 174 (2.0) | 133 (2.7) | ***0.013*** |
| *Others* | 422 (5.0) | 114 (2.3) | ***<0.001*** |
| *Total* | 8,497 | 4,902 |  |

**Table 5.** **Leading diagnoses and their International Statistical Classification of Diseases and Related Health Problems, Tenth Revision (ICD-10) codes in six representative months after the COVID-19 pandemic outbreak (September 2020–February 2021) compared with the same period of the year before.** The visits are presented as the number (percentage).

| ***Diagnosis non pandemic*** | ***n*** | ***Diagnosis pandemic*** | ***n*** |
| --- | --- | --- | --- |
| *Conjunctivitis (H10.3)* | 1,438 (16.9) | *Corneal ulcer (H16.0)* | 650 (13.3) |
| *Keratitis (H16.9)* | 1,139 (13.4) | *Keratitis (H16.9)* | 633 (12.9) |
| *Corneal ulcer (H16.0)* | 751 (8.8) | *Conjunctivitis (H10.3)* | 515 (10.5) |
| *Corneal foreign body (T15.0)* | 517 (6.1) | *Corneal foreign body (T15.0)* | 387 (7.9) |
| *Posterior vitreous detachment (H43.81)* | 482 (5.7) | *Posterior vitreous detachment (H43.81)* | 354 (7.2) |
| *Hyposphagma (H 11.3)* | 426 (5.0) | *Hyposphagma (H 11.3)* | 224 (4.6) |
| *Hordeolum (H00.02)* | 367 (5.0) | *Blepharitis (H01.00)* | 209 (4.3) |
| *Ocular trauma (S05.9)* | 351 (4.1) | *Uveitis (H20)* | 195 (4.0) |
| *Blepharitis (H01.00)* | 340 (4.0) | *Hordeolum (H00.02)* | 194 (4.0) |
| *Uveitis (H20)* | 244 (2.9) | *Ocular trauma (S05.9)* | 191 (3.9) |
| *No pathology (H53)* | 239 (2.8) | *Migraine (G43.9)* | 88 (1.8) |

**Fig 1. The number per visits per month in the ophthalmological emergency department.** The mean number of visits per month from January 2015 to February 2020 is shown in green. The number of visits per month from March 2020 to February 2021 is shown in blue. Error bars represented the maximum and minimum number of visits each month from January 2015 to February 2020.

**Fig 2. Regression analysis of the number of COVID-19-positive cases and the number of visits per month during the pandemic year.**

| **Month** | **COVID-19 positive cases** |
| --- | --- |
| *Mar 2020* | 799 |
| *Apr 2020* | 1,691 |
| *May 2020* | 321 |
| *Jun 2020* | 91 |
| *Jul 2020* | 97 |
| *Aug 2020* | 404 |
| *Sep 2020* | 954 |
| *Oct 2020* | 767 |
| *Nov 2020* | 450 |
| *Dec 2020* | 348 |
| *Jan 2021* | 487 |
| *Feb 2021* | 652 |
