# Supplementary material for: Changing trends in ophthalmological emergencies during the COVID-19 pandemic
Source: PLoS One. 2022 May 27;17(5):e0268975. doi: 10.1371/journal.pone.0268975 (PMC9140243; doi:10.1371/journal.pone.0268975)
Supplement: S1 Table — (PDF) [file pone.0268975.s002.pdf]

Sexo	Fecha	CIE-10	Grupo	Diagnóstico	COVID: 1 NO 2 SI
2	01/09/2020	H10.3	0	Conjuntivitis	2
2	01/09/2020	H02	4	Lesión palpebral	2
2	01/09/2020	H35.30	8	DMAE	2
1	01/09/2020	H10.3	0	Conjuntivitis	2
2	01/09/2020	H35.3	8	Maculopatía	2
1	01/09/2020	H00.02	4	Orzuelo	2
2	01/09/2020	H10.3	0	Conjuntivitis	2
2	01/09/2020	H43.81	7	DVP	2
1	01/09/2020	H16.9	1	Queratitis	2
2	01/09/2020	H0.41	1	Ojo seco	2
2	01/09/2020	H16.0	1	Úlcera corneal	2
2	01/09/2020	H16.9	1	Queratitis	2
1	01/09/2020	H33.30	8	Desgarro retiniano	2
2	01/09/2020	H43.81	7	DVP	2
2	01/09/2020	H01.00	4	Blefaritis	2
2	01/09/2020	H35.30	8	DMAE	2
2	01/09/2020	H43.81	7	DVP	2
2	01/09/2020	H33.30	8	Desgarro retiniano	2
1	01/09/2020	H02.05	4	Distiquiasis	2
1	01/09/2020	H27.8	2	OCP	2
2	01/09/2020	H02	4	Alteración palpebral	2
2	01/09/2020	H31.42	8	Coroidopatía serosa central	2
2	01/09/2020	H43.81	7	DVP	2
2	01/09/2020	H53	11	No patología	2
1	01/09/2020	H17.8	1	Infiltrados corneales	2
1	01/09/2020	S05.9	9	Traumatismo	2
2	01/09/2020	H20	6	Uveitis	2
1	01/09/2020	H43.81	7	DVP	2
2	01/09/2020	H00.02	4	Orzuelo	2
1	01/09/2020	H16.0	1	Úlcera corneal	2
1	01/09/2020	T15.0	1	Cuerpo extraño	2
1	01/09/2020	H16.0	1	Úlcera corneal	2
1	01/09/2020	H16.0	1	Úlcera corneal	2
1	01/09/2020	H16.9	1	Queratitis	2
1	02/09/2020	T15.0	1	Cuerpo extraño	2
1	02/09/2020	H35.3	8	Maculopatía	2
1	02/09/2020	T15.0	1	Cuerpo extraño	2
1	02/09/2020	H00.02	4	Orzuelo	2
2	02/09/2020	H35.30	8	DMAE	2
2	02/09/2020	H26.9	2	Catarata	2
2	02/09/2020	H10.3	0	Conjuntivitis	2
2	02/09/2020	H16.9	1	Queratitis	2
2	02/09/2020	H01.00	4	Blefaritis	2
2	02/09/2020	H00.02	4	Orzuelo	2
2	02/09/2020	H10.3	0	Conjuntivitis	2
2	02/09/2020	H10.3	0	Conjuntivitis	2
1	02/09/2020	H18.20	1	Edema corneal	2
1	02/09/2020	H26.9	2	Catarata	2
1	02/09/2020	H43.1	7	Hemovítreo	2
2	02/09/2020	H40.9	3	Glaucoma	2
2	02/09/2020	H02	4	Alteración palpebral	2
2	02/09/2020	H01.00	4	Blefaritis	2
1	02/09/2020	H16.9	1	Queratitis	2
1	02/09/2020	H10.3	0	Conjuntivitis	2
2	02/09/2020	H53	8	No patología	2
1	02/09/2020	H16.9	1	Queratitis	2
2	02/09/2020	H43.81	7	DVP	2
2	02/09/2020	H16.9	1	Queratitis	2
1	02/09/2020	H16.9	1	Queratitis	2
2	02/09/2020	H20	6	Uveitis	2
2	02/09/2020	H01.00	4	Blefaritis	2
2	02/09/2020	H16.0	1	Úlcera corneal	2
1	02/09/2020	H10.3	0	Conjuntivitis	2
1	02/09/2020	T15.0	1	Cuerpo extraño	2
1	02/09/2020	S05.9	9	Traumatismo	2
2	02/09/2020	H43.81	7	DVP	2
1	03/09/2020	H16.0	1	Úlcera corneal	2
1	03/09/2020	H16.9	1	Queratitis	2

2	03/09/2020	H16.9	1	Queratitis	2
1	03/09/2020	H27.10	2	Luxación LIO	2
1	03/09/2020	H16.0	1	Úlcera corneal	2
2	03/09/2020	H16.0	1	Úlcera corneal	2
2	03/09/2020	H16.0	1	Úlcera corneal	2
2	03/09/2020	H16.9	1	Queratitis	2
2	03/09/2020	G43.9	10	Migraña	2
1	03/09/2020	H43.81	7	DVP	2
2	03/09/2020	H15.00	6	Escleritis	2
1	03/09/2020	T15.0	1	Cuerpo extraño	2
2	03/09/2020	H10.3	0	Conjuntivitis	2
2	03/09/2020	H01.00	4	Blefaritis	2
1	03/09/2020	H10.3	0	Conjuntivitis	2
1	03/09/2020	H16.0	1	Úlcera corneal	2
1	03/09/2020	H02.05	4	Distiquiasis	2
1	03/09/2020	H16.0	1	Úlcera corneal	2
1	03/09/2020	T15.0	1	Cuerpo extraño	2
1	03/09/2020	H16.9	1	Queratitis	2
1	04/09/2020	H16.9	1	Queratitis	2
1	04/09/2020	H43.81	7	DVP	2
1	04/09/2020	H27.8	2	OCP	2
2	04/09/2020	H53	11	No patología	2
2	04/09/2020	H10.3	0	Conjuntivitis	2
2	04/09/2020	H35.30	8	DMAE	2
2	04/09/2020	H20	6	Uveitis	2
1	04/09/2020	H16.0	1	Úlcera corneal	2
2	04/09/2020	H27.8	2	OCP	2
2	04/09/2020	H10.3	0	Conjuntivitis	2
2	04/09/2020	H02	4	Alteración palpebral	2
2	04/09/2020	H10.3	0	Conjuntivitis	2
2	04/09/2020	H43.81	7	DVP	2
2	04/09/2020	H0.41	1	Ojo seco	2
2	04/09/2020	H16.9	1	Queratitis	2
2	04/09/2020	H43.81	7	DVP	2
1	04/09/2020	H43.81	7	DVP	2
2	04/09/2020	H01.00	4	Blefaritis	2
2	04/09/2020	H43.81	7	DVP	2
1	04/09/2020	H43.1	8	Hemovítreo	2
1	04/09/2020	H16.0	1	Úlcera corneal	2
1	04/09/2020	T15.0	1	Cuerpo extraño	2
2	04/09/2020	T15.0	1	Cuerpo extraño	2
1	04/09/2020	G43.9	10	Migraña	2
2	04/09/2020	H10.3	0	Conjuntivitis	2
2	04/09/2020	H16.0	4	Úlcera corneal	2
1	04/09/2020	H16.9	1	Queratitis	2
1	05/09/2020	H16.9	1	Queratitis	2
2	05/09/2020	H16.0	1	Úlcera corneal	2
1	05/09/2020	H10.3	0	Conjuntivitis	2
1	05/09/2020	T15.0	1	Cuerpo extraño	2
1	05/09/2020	H18.20	1	Edema corneal	2
1	05/09/2020	G43.9	10	Migraña	2
2	05/09/2020	H10.3	0	Conjuntivitis	2
2	05/09/2020	H16.9	1	Queratitis	2
2	05/09/2020	H00.02	4	Orzuelo	2
2	05/09/2020	H43.81	7	DVP	2
2	05/09/2020	H43.81	7	DVP	2
1	05/09/2020	T15.0	1	Cuerpo extraño	2
1	05/09/2020	S05.30	9	Laceración conjuntival	2
1	05/09/2020	H16.0	1	Úlcera corneal	2
1	05/09/2020	H20	6	Uveitis	2
2	05/09/2020	G43.9	10	Migraña	2
2	05/09/2020	H16.9	1	Queratitis	2
2	05/09/2020	H11.3	0	Hiposfagma	2
2	05/09/2020	H53	11	No patología	2
2	05/09/2020	H34.82	8	Trombosis venosa	2
1	05/09/2020	H18.50	1	Distrofia corneal	2
1	05/09/2020	H16.0	1	Úlcera corneal	2
2	05/09/2020	S05.30	9	Laceración conjuntival	2
2	05/09/2020	H53	11	No patología	2
2	05/09/2020	H43.81	7	DVP	2
1	05/09/2020	S05.9	9	Traumatismo	2

2	05/09/2020	T15.0	1	Cuerpo extraño	2
1	05/09/2020	S05.9	9	Traumatismo	2
1	05/09/2020	H16.0	1	Úlcera corneal	2
2	05/09/2020	H16.9	1	Queratitis	2
1	05/09/2020	S05.9	9	Traumatismo	2
1	06/09/2020	H16.0	1	Úlcera corneal	2
1	06/09/2020	S05.9	9	Traumatismo	2
2	06/09/2020	Alta por fuga	11	Alta por fuga	2
2	06/09/2020	H00.02	4	Orzuelo	2
2	06/09/2020	H16.0	1	Úlcera corneal	2
1	06/09/2020	H16.0	1	Úlcera corneal	2
1	06/09/2020	H00.02	4	Orzuelo	2
1	06/09/2020	H16.0	1	Úlcera corneal	2
2	06/09/2020	H16.9	1	Queratitis	2
2	06/09/2020	H11.3	0	Hiposfagma	2
1	06/09/2020	H16.0	1	Úlcera corneal	2
1	06/09/2020	H16.9	1	Queratitis	2
1	06/09/2020	H16.9	1	Queratitis	2
2	06/09/2020	H16.9	1	Queratitis	2
1	06/09/2020	H53	11	No patología	2
2	06/09/2020	H16.0	1	Úlcera corneal	2
2	06/09/2020	H10.3	0	Conjuntivitis	2
1	06/09/2020	H10.3	0	Conjuntivitis	2
2	06/09/2020	H53	11	No patología	2
2	06/09/2020	H16.9	1	Queratitis	2
2	06/09/2020	H16.9	1	Queratitis	2
2	06/09/2020	H11.3	0	Hiposfagma	2
2	06/09/2020	H16.9	1	Queratitis	2
2	06/09/2020	H11.3	0	Hiposfagma	2
2	06/09/2020	H15.00	6	Escleritis	2
2	06/09/2020	Alta por fuga	11	Alta por fuga	2
2	06/09/2020	H00.02	4	Orzuelo	2
2	06/09/2020	S05.6	9	Perforación ocular	2
2	06/09/2020	H16.9	1	Queratitis	2
1	06/09/2020	H20	6	Uveitis	2
1	07/09/2020	T15.0	1	Cuerpo extraño	2
1	07/09/2020	H02	4	Lesión palpebral	2
1	07/09/2020	H53	11	No patología	2
2	07/09/2020	H27.8	2	OCP	2
1	07/09/2020	H10.3	0	Conjuntivitis	2
1	07/09/2020	H16.0	1	Úlcera corneal	2
2	07/09/2020	H16.9	1	Queratitis	2
1	07/09/2020	H16.0	1	Úlcera corneal	2
2	07/09/2020	H16.9	1	Queratitis	2
1	07/09/2020	H33.30	8	Desgarro retiniano	2
2	07/09/2020	T15.0	0	Cuerpo extraño	2
2	07/09/2020	H18.20	1	Edema corneal	2
2	07/09/2020	S05.9	9	Traumatismo	2
1	07/09/2020	H16.0	1	Úlcera corneal	2
1	07/09/2020	H01.00	4	Blefaritis	2
2	07/09/2020	H10.3	0	Conjuntivitis	2
2	07/09/2020	H10.3	0	Conjuntivitis	2
1	07/09/2020	H16.0	1	Úlcera corneal	2
2	07/09/2020	H16.0	1	Úlcera corneal	2
2	07/09/2020	H16.0	1	Úlcera corneal	2
1	07/09/2020	H16.0	1	Úlcera corneal	2
2	07/09/2020	H43.81	7	DVP	2
1	07/09/2020	H16.9	1	Queratitis	2
2	07/09/2020	H43.81	7	DVP	2
1	07/09/2020	H16.0	1	Úlcera corneal	2
1	07/09/2020	H15.1	6	Epiescleritis	2
2	07/09/2020	H43.81	7	DVP	2
1	07/09/2020	H00.02	4	Orzuelo	2
2	08/09/2020	H16.0	1	Úlcera corneal	2
1	08/09/2020	H16.0	1	Úlcera corneal	2
2	08/09/2020	H16.9	1	Queratitis	2
2	08/09/2020	H10.3	0	Conjuntivitis	2
2	08/09/2020	H43.81	7	DVP	2
2	08/09/2020	H10.3	0	Conjuntivitis	2
2	08/09/2020	H16.0	1	Úlcera corneal	2
2	08/09/2020	H16.9	1	Queratitis	2

1	08/09/2020	H53	11	No patología	2
2	08/09/2020	H53	11	No patología	2
2	08/09/2020	Z98.4	1	Post op catarata	2
2	08/09/2020	H01.00	4	Blefaritis	2
2	08/09/2020	H10.3	0	Conjuntivitis	2
2	08/09/2020	H16.9	1	Queratitis	2
1	08/09/2020	H11.3	0	Hiposfagma	2
1	08/09/2020	H10.3	0	Conjuntivitis	2
2	08/09/2020	H00.02	4	Orzuelo	2
2	08/09/2020	H43.81	7	DVP	2
2	08/09/2020	H16.9	1	Queratitis	2
2	08/09/2020	H16.0	1	Úlcera corneal	2
2	08/09/2020	H34.82	8	Trombosis venosa	2
1	08/09/2020	H18.20	1	Edema corneal	2
1	08/09/2020	T15.0	1	Cuerpo extraño	2
1	08/09/2020	T15.0	1	Cuerpo extraño	2
1	08/09/2020	T15.0	1	Cuerpo extraño	2
2	08/09/2020	T15.0	1	Cuerpo extraño	2
2	08/09/2020	H10.3	0	Conjuntivitis	2
2	08/09/2020	H16.0	1	Úlcera corneal	2
1	09/09/2020	H15.1	6	Epiescleritis	2
1	09/09/2020	H16.0	1	Úlcera corneal	2
1	09/09/2020	H16.0	1	Úlcera corneal	2
2	09/09/2020	H43.81	7	DVP	2
1	09/09/2020	H16.9	1	Queratitis	2
2	09/09/2020	S05.9	9	Traumatismo	2
2	09/09/2020	H43.81	7	DVP	2
2	09/09/2020	H01.00	4	Blefaritis	2
2	09/09/2020	H01.00	4	Blefaritis	2
2	09/09/2020	H43.81	7	DVP	2
2	09/09/2020	H53	11	No patología	2
1	09/09/2020	H53	11	No patología	2
2	09/09/2020	H30.9	6	Uveitis posterior	2
1	09/09/2020	H43.81	7	DVP	2
1	09/09/2020	T15.0	1	Cuerpo extraño	2
1	09/09/2020	H05.01	4	Celulitis orbitaria	2
2	09/09/2020	H35.30	8	DMAE	2
2	09/09/2020	H10.3	0	Conjuntivitis	2
1	09/09/2020	T15.0	1	Cuerpo extraño	2
1	09/09/2020	T15.0	1	Cuerpo extraño	2
1	09/09/2020	H01.00	4	Blefaritis	2
1	09/09/2020	T26	9	Causticación	2
2	09/09/2020	T15.0	1	Cuerpo extraño	2
1	09/09/2020	H31.42	8	Coroidopatía serosa central	2
2	09/09/2020	H01.00	4	Blefaritis	2
1	09/09/2020	H00.02	4	Orzuelo	2
2	09/09/2020	H33.30	8	Desgarro retiniano	2
2	09/09/2020	H10.3	0	Conjuntivitis	2
2	09/09/2020	H11.3	0	Hiposfagma	2
1	09/09/2020	H34.9	8	Oclusión arterial	2
1	10/09/2020	T15.0	1	Cuerpo extraño	2
2	10/09/2020	H27.8	2	OCP	2
1	10/09/2020	H20	6	Uveitis	2
2	10/09/2020	H16.9	1	Queratitis	2
2	10/09/2020	H10.3	0	Conjuntivitis	2
2	10/09/2020	G43.9	10	Migraña	2
2	10/09/2020	H10.3	0	Conjuntivitis	2
2	10/09/2020	Alta por fuga	11	Alta por fuga	2
1	10/09/2020	H43.1	7	Hemovítreo	2
1	10/09/2020	H27.8	2	OCP	2
1	10/09/2020	G43.9	10	Migraña	2
1	10/09/2020	T15.0	1	Cuerpo extraño	2
2	10/09/2020	H43.81	7	DVP	2
2	10/09/2020	H11.3	0	Hiposfagma	2
2	10/09/2020	H11.3	0	Hiposfagma	2
1	10/09/2020	G43.109	10	Aura	2
1	10/09/2020	H16.0	1	Úlcera corneal	2
1	10/09/2020	H16.9	1	Queratitis	2
1	10/09/2020	H10.3	0	Conjuntivitis	2
1	10/09/2020	H16.0	1	Úlcera corneal	2
2	10/09/2020	H53	11	No patología	2

1	10/09/2020	H16.0	1	Úlcera corneal	2
1	10/09/2020	H33.0	8	Desprendimiento de retina	2
2	10/09/2020	H11.3	0	Hiposfagma	2
2	10/09/2020	H53	11	No patología	2
1	11/09/2020	H20	6	Uveitis	2
2	11/09/2020	H10.3	0	Conjuntivitis	2
1	11/09/2020	H20	6	Uveitis	2
1	11/09/2020	H27.8	2	OCP	2
2	11/09/2020	H18.50	1	Distrofia corneal	2
1	11/09/2020	H16.0	1	Úlcera corneal	2
1	11/09/2020	Alta por fuga	11	Alta por fuga	2
1	11/09/2020	H01.00	4	Blefaritis	2
1	11/09/2020	H00.02	4	Orzuelo	2
1	11/09/2020	H16.9	1	Queratitis	2
1	11/09/2020	H43.81	7	DVP	2
2	11/09/2020	H10.3	0	Conjuntivitis	2
1	11/09/2020	T15.0	1	Cuerpo extraño	2
1	11/09/2020	H26.9	2	Catarata	2
1	11/09/2020	T15.0	1	Cuerpo extraño	2
2	11/09/2020	H01.00	4	Blefaritis	2
2	11/09/2020	H16.9	1	Queratitis	2
1	11/09/2020	H11.3	0	Hiposfagma	2
2	11/09/2020	H40.21	3	Glaucoma agudo	2
1	11/09/2020	H16.9	1	Queratitis	2
2	11/09/2020	H16.9	1	Queratitis	2
2	11/09/2020	H16.0	1	Úlcera corneal	2
1	11/09/2020	H01.00	4	Blefaritis	2
2	11/09/2020	H10.3	0	Conjuntivitis	2
1	11/09/2020	T26	9	Causticación	2
1	11/09/2020	H16.0	1	Úlcera corneal	2
1	11/09/2020	H10.3	0	Conjuntivitis	2
2	11/09/2020	H16.9	1	Queratitis	2
2	11/09/2020	H16.9	1	Queratitis	2
2	11/09/2020	H16.9	1	Queratitis	2
1	11/09/2020	T15.0	1	Cuerpo extraño	2
2	11/09/2020	B00.1	4	Dermatitis herpética	2
2	11/09/2020	H16.9	1	Queratitis	2
1	11/09/2020	H16.0	1	Úlcera corneal	2
1	11/09/2020	H18.20	1	Edema corneal	2
1	11/09/2020	H16.0	1	Úlcera corneal	2
2	11/09/2020	H16.9	1	Queratitis	2
2	11/09/2020	H01.00	4	Blefaritis	2
1	12/09/2020	H10.3	0	Conjuntivitis	2
2	12/09/2020	H16.0	1	Úlcera corneal	2
2	12/09/2020	H01.00	4	Blefaritis	2
1	12/09/2020	H10.3	0	Conjuntivitis	2
2	12/09/2020	H43.81	7	DVP	2
1	12/09/2020	H11.3	0	Hiposfagma	2
2	12/09/2020	H53	11	No patología	2
2	12/09/2020	H04.32	5	Dacriocistitis aguda	2
1	12/09/2020	H53	11	No patología	2
1	12/09/2020	H10.3	0	Conjuntivitis	2
2	12/09/2020	H43.81	7	DVP	2
1	12/09/2020	H10.3	0	Conjuntivitis	2
1	12/09/2020	S05.9	9	Traumatismo	2
2	12/09/2020	H43.81	7	DVP	2
2	12/09/2020	H35.3	8	Maculopatía	2
2	12/09/2020	B58.0	8	Toxoplasmosis	2
1	12/09/2020	H16.0	1	Úlcera corneal	2
1	12/09/2020	H16.0	1	Úlcera corneal	2
2	12/09/2020	H35.30	8	DMAE	2
1	12/09/2020	H16.9	1	Queratitis	2
1	12/09/2020	H16.0	1	Úlcera corneal	2
1	12/09/2020	H00.02	4	Orzuelo	2
1	12/09/2020	H00.02	4	Orzuelo	2
1	12/09/2020	T15.0	1	Cuerpo extraño	2
2	12/09/2020	H43.81	7	DVP	2
1	12/09/2020	H43.81	7	DVP	2
2	12/09/2020	E11.319	8	Retinopatía diabética	2
2	12/09/2020	H43.81	7	DVP	2
2	12/09/2020	H10.3	0	Conjuntivitis	2

2	12/09/2020	H35.3	11	Maculopatía	2
1	12/09/2020	H16.0	1	Úlcera corneal	2
2	12/09/2020	H16.0	1	Úlcera corneal	2
2	12/09/2020	H00.02	4	Orzuelo	2
1	12/09/2020	T15.0	1	Cuerpo extraño	2
1	13/09/2020	H16.0	1	Úlcera corneal	2
1	13/09/2020	H16.0	1	Úlcera corneal	2
2	13/09/2020	H16.0	1	Úlcera corneal	2
2	13/09/2020	H02.05	4	Distiquiasis	2
1	13/09/2020	T15.0	1	Cuerpo extraño	2
1	13/09/2020	H10.3	0	Conjuntivitis	2
2	13/09/2020	H11.3	0	Hiposfagma	2
2	13/09/2020	H16.0	1	Úlcera corneal	2
2	13/09/2020	H16.9	1	Queratitis	2
2	13/09/2020	H20	6	Uveitis	2
1	13/09/2020	S05.9	9	Traumatismo	2
2	13/09/2020	H16.9	1	Queratitis	2
2	13/09/2020	H16.0	1	Úlcera corneal	2
1	13/09/2020	H10.3	0	Conjuntivitis	2
2	13/09/2020	H15.1	6	epiescleritis	2
1	13/09/2020	H16.0	1	Úlcera corneal	2
2	14/09/2020	H16.0	1	Úlcera corneal	2
1	14/09/2020	H43.81	7	DVP	2
1	14/09/2020	H10.3	0	Conjuntivitis	2
1	14/09/2020	H33.0	8	Desprendimiento de retina	2
1	14/09/2020	H16.0	1	Úlcera corneal	2
2	14/09/2020	H16.0	1	Úlcera corneal	2
1	14/09/2020	H16.9	1	Queratitis	2
1	14/09/2020	H26.9	2	Catarata	2
2	14/09/2020	H11.3	0	Hiposfagma	2
1	14/09/2020	H16.9	1	Queratitis	2
2	14/09/2020	H10.3	0	Conjuntivitis	2
2	14/09/2020	G43.109	10	Aura	2
1	14/09/2020	H11.3	0	Hiposfagma	2
2	14/09/2020	H53	11	No patología	2
2	14/09/2020	H10.3	0	Conjuntivitis	2
2	14/09/2020	H53	11	No patología	2
1	14/09/2020	S05.9	9	Traumatismo	2
2	14/09/2020	H10.3	0	Conjuntivitis	2
1	14/09/2020	H16.0	1	Úlcera corneal	2
2	14/09/2020	H10.3	0	Conjuntivitis	2
2	14/09/2020	H16.9	1	Queratitis	2
2	14/09/2020	H16.0	1	Úlcera corneal	2
1	14/09/2020	H35.30	8	DMAE	2
2	14/09/2020	H43.81	7	DVP	2
2	14/09/2020	H16.0	1	Úlcera corneal	2
1	14/09/2020	S05.9	9	Traumatismo	2
1	14/09/2020	T15.0	1	Cuerpo extraño	2
1	14/09/2020	T26	9	Causticación	2
1	15/09/2020	T26	9	Causticación	2
2	15/09/2020	H10.3	0	Conjuntivitis	2
2	15/09/2020	H18.20	1	Edema corneal	2
2	15/09/2020	S05.9	9	Traumatismo	2
1	15/09/2020	H10.3	0	Conjuntivitis	2
1	15/09/2020	H10.3	0	Conjuntivitis	2
2	15/09/2020	H02	4	Lesión palpebral	2
2	15/09/2020	H16.9	1	Queratitis	2
1	15/09/2020	H15.1	6	Epiescleritis	2
1	15/09/2020	H16.9	1	Queratitis	2
2	15/09/2020	H16.0	1	Úlcera corneal	2
2	15/09/2020	H43.81	7	DVP	2
1	15/09/2020	H53	11	No patología	2
2	15/09/2020	H53.10	11	Problema refractivo	2
1	15/09/2020	H16.9	1	Queratitis	2
1	15/09/2020	H16.0	1	Úlcera corneal	2
1	15/09/2020	H16.0	1	Úlcera corneal	2
2	15/09/2020	H16.0	1	Úlcera corneal	2
1	15/09/2020	H15.1	6	Epiescleritis	2
1	15/09/2020	T15.0	1	Cuerpo extraño	2
1	15/09/2020	H53	11	No patología	2
1	15/09/2020	H20	6	Uveitis	2

1	15/09/2020	H16.0	1	Úlcera corneal	2
1	15/09/2020	H34.82	8	Trombosis venosa	2
1	15/09/2020	H16.9	1	Queratitis	2
1	15/09/2020	H01.00	4	Blefaritis	2
1	15/09/2020	S05.9	9	Traumatismo	2
1	15/09/2020	Z97.0	4	Protesis ocular	2
1	15/09/2020	T15.0	1	Cuerpo extraño	2
1	15/09/2020	T15.0	1	Cuerpo extraño	2
1	15/09/2020	S05.9	9	Traumatismo	2
1	15/09/2020	H16.9	1	Queratitis	2
2	16/09/2020	H18.20	1	Edema corneal	2
2	16/09/2020	H00.02	4	Orzuelo	2
2	16/09/2020	H53	11	No patología	2
2	16/09/2020	H53	11	No patología	2
2	16/09/2020	H53	11	No patología	2
2	16/09/2020	H00.02	4	Orzuelo	2
1	16/09/2020	T15.0	1	Cuerpo extraño	2
2	16/09/2020	H10.3	0	Conjuntivitis	2
2	16/09/2020	H43.81	7	DVP	2
1	16/09/2020	H10.3	0	Conjuntivitis	2
1	16/09/2020	H40.9	3	Glaucoma	2
1	16/09/2020	H16.9	1	Queratitis	2
2	16/09/2020	H10.3	0	Conjuntivitis	2
2	16/09/2020	H16.0	1	Úlcera corneal	2
1	16/09/2020	Z98.4	1	Post op catarata	2
1	16/09/2020	H16.0	1	Úlcera corneal	2
2	16/09/2020	T15.0	1	Cuerpo extraño	2
1	16/09/2020	H10.3	0	Conjuntivitis	2
2	16/09/2020	H20	6	Uveitis	2
1	16/09/2020	H10.3	0	Conjuntivitis	2
2	16/09/2020	H20	6	Uveitis	2
1	16/09/2020	H10.3	0	Conjuntivitis	2
2	16/09/2020	H10.3	0	Conjuntivitis	2
2	16/09/2020	H53	11	No patología	2
1	17/09/2020	H16.9	1	Queratitis	2
2	17/09/2020	H20	6	Uveitis	2
1	17/09/2020	H00.02	4	Orzuelo	2
2	17/09/2020	H43.81	7	DVP	2
2	17/09/2020	H00.02	4	Orzuelo	2
2	17/09/2020	H27.8	2	OCP	2
1	17/09/2020	H16.0	1	Úlcera corneal	2
1	17/09/2020	H16.0	1	Úlcera corneal	2
1	17/09/2020	H11.3	0	hiposfagma	2
2	17/09/2020	H16.0	1	Úlcera corneal	2
1	17/09/2020	H43.81	7	DVP	2
2	17/09/2020	H16.9	1	Queratitis	2
1	17/09/2020	H16.0	1	Úlcera corneal	2
2	17/09/2020	H43.81	7	DVP	2
1	17/09/2020	H16.9	1	Queratitis	2
2	17/09/2020	H01.00	4	blefaritis	2
1	17/09/2020	H16.9	1	queratitis	2
2	17/09/2020	H01.00	4	blefaritis	2
2	17/09/2020	H10.3	0	Conjuntivitis	2
2	17/09/2020	H01.00	4	Blefaritis	2
2	17/09/2020	H43.81	7	DVP	2
1	17/09/2020	H16.0	1	Úlcera corneal	2
2	18/09/2020	H16.9	1	Queratitis	2
2	18/09/2020	G43.9	10	Migraña	2
1	18/09/2020	H33.30	8	Desgarro retiniano	2
2	18/09/2020	S05.9	9	Traumatismo	2
2	18/09/2020	H16.9	1	Queratitis	2
2	18/09/2020	H43.81	7	DVP	2
1	18/09/2020	H11.3	0	Hiposfagma	2
2	18/09/2020	H43.81	7	DVP	2
2	18/09/2020	H53	11	No patología	2
2	18/09/2020	H10.3	9	Conjuntivitis	2
2	18/09/2020	H16.0	1	Úlcera corneal	2
2	18/09/2020	H16.9	1	Queratitis	2
2	18/09/2020	H26.9	2	Catarata	2
1	18/09/2020	H10.81	0	Pingueculitis	2
1	18/09/2020	T15.0	1	Cuerpo extraño	2

2	18/09/2020	H35.0	8	Hemorragia retiniana	2
1	18/09/2020	T15.0	1	Cuerpo extraño	2
1	19/09/2020	H00.02	4	Orzuelo	2
1	19/09/2020	H16.0	1	Úlcera corneal	2
1	19/09/2020	H43.1	7	Hemovítreo	2
1	19/09/2020	H43.81	7	DVP	2
2	19/09/2020	H02	4	Lesión palpebral	2
2	19/09/2020	H27.8	2	OCP	2
2	19/09/2020	H10.3	0	Conjuntivitis	2
1	19/09/2020	T15.0	1	Cuerpo extraño	2
1	19/09/2020	H10.3	0	Conjuntivitis	2
2	19/09/2020	H16.9	1	Queratitis	2
2	19/09/2020	H0.41	1	Ojo seco	2
1	19/09/2020	Alta por fuga	11	Alta por fuga	2
2	19/09/2020	H43.81	7	DVP	2
1	19/09/2020	H00.19	4	Chalazión	2
1	19/09/2020	H10.3	0	Conjuntivitis	2
1	20/09/2020	H10.3	0	Conjuntivitis	2
1	20/09/2020	H11.3	0	Hiposfagma	2
2	20/09/2020	H00.03	4	Celulitis preseptal	2
2	20/09/2020	H10.3	0	Conjuntivitis	2
1	20/09/2020	H16.0	9	Úlcera corneal	2
2	20/09/2020	S05.30	9	Laceración conjuntival	2
1	20/09/2020	H16.0	1	Úlcera corneal	2
2	20/09/2020	H40.9	3	Glaucoma	2
2	20/09/2020	H00.02	4	Orzuelo	2
1	20/09/2020	H16.9	1	Queratitis	2
2	20/09/2020	H16.0	1	Úlcera corneal	2
1	20/09/2020	H20	6	Uveitis	2
1	20/09/2020	T15.0	1	Cuerpo extraño	2
1	20/09/2020	H16.9	1	queratitis	2
1	20/09/2020	H16.0	1	Úlcera corneal	2
1	20/09/2020	T15.0	1	Cuerpo extraño	2
1	20/09/2020	H16.0	1	Úlcera corneal	2
1	20/09/2020	S05.9	9	Traumatismo	2
1	21/09/2020	H16.9	1	queratitis	2
1	21/09/2020	H20	6	Uveitis	2
2	21/09/2020	H16.9	1	Queratitis	2
1	21/09/2020	H53.10	11	Problema refractivo	2
1	21/09/2020	H16.0	1	Úlcera corneal	2
1	21/09/2020	H16.0	1	Úlcera corneal	2
1	21/09/2020	S05.9	9	Traumatismo	2
1	21/09/2020	H11.3	0	Hiposfagma	2
2	21/09/2020	H53	11	No patología	2
1	21/09/2020	H11.3	0	Hiposfagma	2
2	21/09/2020	H16.9	1	Queratitis	2
2	21/09/2020	H10.3	0	Conjuntivitis	2
2	21/09/2020	S05.9	9	Traumatismo	2
1	21/09/2020	H00.03	4	Celulitis preseptal	2
1	21/09/2020	H16.0	1	Úlcera corneal	2
2	21/09/2020	H16.0	1	Úlcera corneal	2
2	21/09/2020	H20	6	Uveitis	2
2	21/09/2020	H16.9	1	Queratitis	2
1	21/09/2020	S05.9	9	Traumatismo	2
2	21/09/2020	H16.0	1	Úlcera corneal	2
2	21/09/2020	S05.30	9	Laceración conjuntival	2
1	21/09/2020	T15.0	1	Cuerpo extraño	2
2	21/09/2020	H16.0	1	Úlcera corneal	2
1	22/09/2020	T15.0	1	Cuerpo extraño	2
1	22/09/2020	H49.9	10	Parálisis oculomotora	2
1	22/09/2020	H16.0	1	Úlcera corneal	2
2	22/09/2020	H10.3	0	Conjuntivitis	2
2	22/09/2020	H0.41	1	ojo seco	2
1	22/09/2020	H16.9	1	Queratitis	2
1	22/09/2020	H0.41	1	Ojo seco	2
2	22/09/2020	H53	11	No patología	2
1	22/09/2020	H18.50	1	Distrofia corneal	2
2	22/09/2020	T15.0	1	Cuerpo extraño	2
1	22/09/2020	H00.19	4	Chalazión	2
2	22/09/2020	H11.3	0	Hiposfagma	2
2	22/09/2020	H10.5	0	Blefaroconjuntivitis	2

1	22/09/2020	H20	6	Uveitis		2
1	22/09/2020	H16.0	1	Úlcera corneal		2
1	22/09/2020	H27.8	2	OCP		2
1	22/09/2020	T15.0	1	Cuerpo extraño		2
2	22/09/2020	H10.3	0	Conjuntivitis		2
1	22/09/2020	H00.02	4	Orzuelo		2
1	22/09/2020	T15.0	1	Cuerpo extraño		2
1	23/09/2020	T15.0	1	Cuerpo extraño		2
1	23/09/2020	H20	6	Uveitis		2
1	23/09/2020	H10.3	0	Conjuntivitis		2
2	23/09/2020	H00.02	4	Orzuelo		2
1	23/09/2020	H00.02	4	Orzuelo		2
2	23/09/2020	H40.9	3	Glaucoma		2
2	23/09/2020	H43.81	7	DVP		2
2	23/09/2020	H01.00	4	Blefaritis		2
2	23/09/2020	H27.8	2	OCP		2
1	23/09/2020	H53	11	No patología		2
2	23/09/2020	H16.9	1	Queratitis		2
2	23/09/2020	H59.88	11	Post op retina		2
1	23/09/2020	H34.82	8	Trombosis venosa		2
2	23/09/2020	H20	6	Uveitis		2
1	23/09/2020	H02	4	Lesión palpebral		2
2	23/09/2020	H35.30	8	DMAE		2
2	23/09/2020	H16.0	1	Úlcera corneal		2
2	23/09/2020	H16.0	1	Úlcera corneal		2
1	23/09/2020	T15.0	1	Cuerpo extraño		2
1	23/09/2020	H10.3	0	Conjuntivitis		2
2	23/09/2020	H10.3	0	Conjuntivitis		2
2	24/09/2020	H10.5	0	Blefaroconjuntivitis		2
2	24/09/2020	H00.02	4	Orzuelo		2
2	24/09/2020	H40.05	3	HTO		2
1	24/09/2020	H53	11	No patología		2
2	24/09/2020	H35.30	8	DMAE		2
2	24/09/2020	H35.30	8	DMAE		2
2	24/09/2020	H20	6	Uveitis		2
1	24/09/2020	H16.0	1	Úlcera corneal		2
1	24/09/2020	H27.1	2	Luxación catarata		2
2	24/09/2020	H10.3	0	Conjuntivitis		2
1	24/09/2020	H16.0	1	Úlcera corneal		2
1	24/09/2020	H40.5	3	Glaucoma secundario		2
2	24/09/2020	H35.3	8	Maculopatía		2
1	24/09/2020	H10.3	0	Conjuntivitis		2
1	24/09/2020	T15.0	1	Cuerpo extraño		2
1	24/09/2020	H33.0	8	Desprendimiento de retina		2
1	24/09/2020	T15.0	1	Cuerpo extraño		2
1	24/09/2020	T15.0	1	Cuerpo extraño		2
1	24/09/2020	H00.02	4	Orzuelo		2
1	24/09/2020	H16.0	1	Úlcera corneal		2
1	25/09/2020	H16.0	1	Úlcera corneal		2
1	25/09/2020	H10.3	0	Conjuntivitis		2
2	25/09/2020	H16.9	1	Queratitis		2
2	25/09/2020	H18.20	1	Edema corneal		2
1	25/09/2020	H16.0	1	Úlcera corneal		2
1	25/09/2020	H00.02	4	Orzuelo		2
1	25/09/2020	H20	6	Uveitis		2
2	25/09/2020	H16.9	1	Queratitis		2
2	25/09/2020	T15.0	1	Cuerpo extraño		2
2	25/09/2020	H33.30	8	Desgarro retiniano		2
1	25/09/2020	H16.0	1	Úlcera corneal		2
2	25/09/2020	H46	11	Neuritis óptica		2
2	25/09/2020	H16.9	1	Queratitis		2
2	25/09/2020	H35.0	8	Hemorragia retiniana		2
2	25/09/2020	H15.1	6	Epiescleritis		2
1	25/09/2020	H16.0	1	Úlcera corneal		2
1	25/09/2020	H10.3	0	Conjuntivitis		2
2	25/09/2020	Alta por fuga	11	Alta por fuga		2
2	25/09/2020	H43.81	7	DVP		2
1	25/09/2020	H16.0	1	Úlcera corneal		2
2	25/09/2020	H10.3	0	Conjuntivitis		2
1	25/09/2020	H16.0	1	Úlcera corneal		2
2	25/09/2020	H20	6	Uveitis		2

2	25/09/2020	H16.0	1	Úlcera corneal	2
1	25/09/2020	H16.0	1	Úlcera corneal	2
1	26/09/2020	H16.9	1	Queratitis	2
1	26/09/2020	H16.0	1	Úlcera corneal	2
2	26/09/2020	T15.0	1	Cuerpo extraño	2
1	26/09/2020	H16.0	1	Úlcera corneal	2
2	26/09/2020	H10.3	0	Conjuntivitis	2
2	26/09/2020	Z98.4	11	Post op catarata	2
1	26/09/2020	H15.1	6	Epiescleritis	2
2	26/09/2020	H04.32	5	Dacriocistitis aguda	2
1	26/09/2020	H00.02	4	Orzuelo	2
2	26/09/2020	H16.9	1	Queratitis	2
2	26/09/2020	H01.00	4	Blefaritis	2
1	26/09/2020	H16.9	1	Queratitis	2
2	26/09/2020	H16.0	1	Úlcera corneal	2
2	26/09/2020	H16.0	1	Úlcera corneal	2
1	26/09/2020	T15.0	1	Cuerpo extraño	2
1	26/09/2020	T15.0	1	Cuerpo extraño	2
2	26/09/2020	H35.3	8	Maculopatía	2
2	26/09/2020	H00.02	4	Orzuelo	2
2	26/09/2020	H43.1	7	Hemovítreo	2
2	26/09/2020	H11.3	0	Hiposfagma	2
1	26/09/2020	H10.3	0	Conjuntivitis	2
2	26/09/2020	H01.00	4	Blefaritis	2
1	26/09/2020	G43.9	10	Migraña	2
1	26/09/2020	H10.3	0	Conjuntivitis	2
1	26/09/2020	H01.00	4	Blefaritis	2
1	26/09/2020	H35.3	8	Maculopatía	2
1	26/09/2020	H16.9	1	Queratitis	2
1	26/09/2020	H16.0	1	Úlcera corneal	2
1	27/09/2020	H16.0	1	Úlcera corneal	2
2	27/09/2020	S05.9	9	Traumatismo	2
1	27/09/2020	H0.41	1	Ojo seco	2
1	27/09/2020	H10.3	0	Conjuntivitis	2
1	27/09/2020	H16.9	1	Queratitis	2
1	27/09/2020	T15.0	1	Cuerpo extraño	2
2	27/09/2020	H01.00	4	Blefaritis	2
2	27/09/2020	H11.3	0	Hiposfagma	2
2	27/09/2020	H16.9	1	Queratitis	2
2	27/09/2020	H00.03	4	Celulitis preseptal	2
1	27/09/2020	H16.0	1	Úlcera corneal	2
2	27/09/2020	H16.0	1	Úlcera corneal	2
1	27/09/2020	T15.0	1	Cuerpo extraño	2
2	27/09/2020	H20	6	Uveitis	2
2	27/09/2020	T15.0	1	Cuerpo extraño	2
1	27/09/2020	S05.9	9	Traumatismo	2
1	27/09/2020	T15.0	1	Cuerpo extraño	2
2	28/09/2020	H16.9	1	Queratitis	2
1	28/09/2020	H20	6	Uveitis	2
2	28/09/2020	H10.3	0	Conjuntivitis	2
2	28/09/2020	H10.3	0	Conjuntivitis	2
1	28/09/2020	H35.30	8	DMAE	2
2	28/09/2020	H35.30	8	DMAE	2
1	28/09/2020	H16.0	1	Úlcera corneal	2
1	28/09/2020	H11.3	0	Hiposfagma	2
2	28/09/2020	H34.82	8	Trombosis venosa	2
1	28/09/2020	H43.81	7	DVP	2
2	28/09/2020	H20	6	Uveitis	2
1	28/09/2020	H16.0	1	Úlcera corneal	2
2	28/09/2020	H43.81	7	DVP	2
2	28/09/2020	S05.00	9	Abrasión conjuntival	2
1	28/09/2020	H11.00	0	Pterigium	2
1	28/09/2020	T15.0	1	Cuerpo extraño	2
1	28/09/2020	H00.19	4	Chalazión	2
2	28/09/2020	H11.3	0	Hiposfagma	2
1	29/09/2020	T15.0	1	Cuerpo extraño	2
2	29/09/2020	H43.81	7	DVP	2
2	29/09/2020	H16.0	1	Úlcera corneal	2
1	29/09/2020	H10.3	0	Conjuntivitis	2
2	29/09/2020	H01.00	4	Blefaritis	2
2	29/09/2020	H10.3	0	Conjuntivitis	2

1	29/09/2020	H20	6	Uveitis	2
1	29/09/2020	H00.02	4	Orzuelo	2
2	29/09/2020	H16.9	1	Queratitis	2
2	29/09/2020	H10.3	0	Conjuntivitis	2
2	29/09/2020	H53.10	11	Problema refractivo	2
2	29/09/2020	H43.1	7	Hemovítreo	2
2	29/09/2020	H16.9	1	Queratitis	2
1	29/09/2020	T15.0	1	Cuerpo extraño	2
2	29/09/2020	H11.3	0	Hiposfagma	2
1	29/09/2020	H00.03	4	Celulitis preseptal	2
1	29/09/2020	H16.0	1	Úlcera corneal	2
1	29/09/2020	H10.3	0	Conjuntivitis	2
2	29/09/2020	H11.3	0	Hiposfagma	2
2	29/09/2020	H16.9	1	Queratitis	2
2	29/09/2020	H10.3	0	Conjuntivitis	2
2	29/09/2020	H40.9	3	Glaucoma	2
1	29/09/2020	H35.30	8	DMAE	2
1	29/09/2020	H16.0	1	Úlcera corneal	2
1	29/09/2020	H16.9	1	Queratitis	2
1	29/09/2020	T15.0	1	Cuerpo extraño	2
1	29/09/2020	Alta por fuga	11	Alta por fuga	2
1	30/09/2020	Z98.4	11	Post op catarata	2
2	30/09/2020	H43.81	7	DVP	2
1	30/09/2020	H43.81	7	DVP	2
1	30/09/2020	H53.9	10	Alteraciones visuales	2
2	30/09/2020	Z98.4	11	Post op catarata	2
1	30/09/2020	Z98.4	11	Post op catarata	2
2	30/09/2020	H0.41	1	ojo seco	2
1	30/09/2020	H43.81	7	DVP	2
2	30/09/2020	H16.9	1	Queratitis	2
1	30/09/2020	H20	6	Uveitis	2
2	30/09/2020	H10.3	0	Conjuntivitis	2
2	30/09/2020	H16.9	1	Queratitis	2
1	30/09/2020	H16.0	1	Úlcera corneal	2
1	30/09/2020	H43.81	7	DVP	2
1	30/09/2020	H43.81	7	DVP	2
2	30/09/2020	H43.81	7	DVP	2
1	30/09/2020	T15.0	1	Cuerpo extraño	2
1	30/09/2020	H16.9	1	Queratitis	2
1	30/09/2020	H16.0	1	Úlcera corneal	2
1	30/09/2020	H16.9	1	Queratitis	2
1	30/09/2020	H16.9	1	Queratitis	2
1	30/09/2020	H35.3	8	Maculopatía	2
1	30/09/2020	H20	6	Uveitis	2
2	30/09/2020	H20	6	Uveitis	2
1	30/09/2020	T15.0	1	Cuerpo extraño	2
2	30/09/2020	G43.9	10	Migraña	2
2	30/09/2020	H16.0	1	Úlcera corneal	2
2	30/09/2020	H16.9	1	Queratitis	2
1	30/09/2020	H16.9	1	Queratitis	2
1	30/09/2020	Z98.4	11	Post op catarata	2
1	30/09/2020	H10.3	0	Conjuntivitis	2
1	30/09/2020	T15.0	1	Cuerpo extraño	2
1	01/10/2020	T15.0	1	Cuerpo extraño	2
2	01/10/2020	H16.9	1	Queratitis	2
1	01/10/2020	H31.42	8	Coroidopatía serosa central	2
2	01/10/2020	H11.3	0	Hiposfagma	2
1	01/10/2020	H53.10	11	Problema refractivo	2
2	01/10/2020	H16.0	1	Úlcera corneal	2
1	01/10/2020	H16.0	1	Úlcera corneal	2
2	01/10/2020	H0.41	1	Ojo seco	2
1	01/10/2020	H43.81	7	DVP	2
2	01/10/2020	H15.00	6	Escleritis	2
1	01/10/2020	S05.9	9	Traumatismo	2
2	01/10/2020	H16.9	1	Queratitis	2
2	01/10/2020	H10.3	0	Conjuntivitis	2
1	01/10/2020	H16.9	1	Queratitis	2
1	01/10/2020	H33.30	9	Desgarro retiniano	2
1	01/10/2020	H00.03	4	Celulitis preseptal	2
1	01/10/2020	S05.9	9	Traumatismo	2
2	01/10/2020	S05.9	9	Traumatismo	2

1	01/10/2020	H16.0	1	Úlcera corneal	2
1	01/10/2020	H16.0	1	Úlcera corneal	2
1	01/10/2020	H16.0	1	Úlcera corneal	2
1	02/10/2020	H16.0	1	Úlcera corneal	2
1	02/10/2020	H16.0	1	Úlcera corneal	2
2	02/10/2020	H33.0	8	Desprendimiento de retina	2
1	02/10/2020	H01.00	4	Blefaritis	2
1	02/10/2020	H0.41	1	Ojo seco	2
2	02/10/2020	H26.9	2	Catarata	2
2	02/10/2020	H00.03	4	Celulitis preseptal	2
2	02/10/2020	H20	6	Uveitis	2
1	02/10/2020	H00.02	4	Orzuelo	2
2	02/10/2020	H11.3	0	Hiposfagma	2
1	02/10/2020	H15.1	6	Epiescleritis	2
2	02/10/2020	H01.00	4	Blefaritis	2
2	02/10/2020	H11.3	0	Hiposfagma	2
1	02/10/2020	T26	9	Causticación	2
2	02/10/2020	H01.00	4	Blefaritis	2
1	02/10/2020	T15.0	1	Cuerpo extraño	2
1	02/10/2020	H16.0	1	Úlcera corneal	2
1	02/10/2020	H16.0	1	Úlcera corneal	2
2	02/10/2020	H59.3	5	Postop via lagrimal	2
2	02/10/2020	H10.5	0	Blefaroconjuntivitis	2
1	02/10/2020	H16.0	1	Úlcera corneal	2
2	02/10/2020	H10.3	0	Conjuntivitis	2
1	02/10/2020	H16.0	1	Úlcera corneal	2
1	02/10/2020	H16.0	1	Úlcera corneal	2
2	02/10/2020	H01.00	4	Blefaritis	2
1	02/10/2020	H16.0	1	Úlcera corneal	2
1	02/10/2020	H16.0	1	Úlcera corneal	2
2	02/10/2020	H43.81	7	DVP	2
1	02/10/2020	T15.0	1	Cuerpo extraño	2
1	03/10/2020	T15.0	1	Cuerpo extraño	2
1	03/10/2020	H16.0	1	Úlcera corneal	2
2	03/10/2020	H11.3	0	Hiposfagma	2
1	03/10/2020	H00.03	4	Celulitis preseptal	2
1	03/10/2020	H16.9	1	Queratitis	2
2	03/10/2020	H10.3	0	Conjuntivitis	2
1	03/10/2020	T15.0	1	Cuerpo extraño	2
1	03/10/2020	H11.3	0	Hiposfagma	2
2	03/10/2020	H10.3	0	Conjuntivitis	2
1	03/10/2020	T15.0	1	Cuerpo extraño	2
2	03/10/2020	H01.00	4	Blefaritis	2
1	03/10/2020	H18.20	1	Edema corneal	2
2	03/10/2020	H20	6	Uveitis	2
1	03/10/2020	T15.0	1	Cuerpo extraño	2
2	03/10/2020	H16.0	1	Úlcera corneal	2
2	03/10/2020	H17.8	1	Infiltrados corneales	2
1	03/10/2020	T15.0	1	Cuerpo extraño	2
1	03/10/2020	H16.0	1	Úlcera corneal	2
1	03/10/2020	H20	6	Uveitis	2
2	03/10/2020	H26.9	2	Catarata	2
2	03/10/2020	H10.81	0	Pingueculitis	2
1	03/10/2020	H16.9	1	Queratitis	2
1	03/10/2020	H10.3	0	Conjuntivitis	2
2	03/10/2020	H11.44	0	Quiste conjuntival	2
1	03/10/2020	H10.3	0	Conjuntivitis	2
2	03/10/2020	H16.9	1	Queratitis	2
1	03/10/2020	H0.41	1	Ojo seco	2
1	03/10/2020	H16.0	1	Úlcera corneal	2
1	03/10/2020	T15.0	1	Cuerpo extraño	2
1	03/10/2020	H40.9	3	Glaucoma	2
2	03/10/2020	H20	6	Uveitis	2
1	03/10/2020	H33.0	8	Desprendimiento de retina	2
2	03/10/2020	H15.1	6	Epiescleritis	2
1	03/10/2020	T15.0	1	Cuerpo extraño	2
2	03/10/2020	H11.3	0	Hiposfagma	2
1	04/10/2020	H10.3	0	Conjuntivitis	2
2	04/10/2020	G43.9	10	Migraña	2
1	04/10/2020	S05.9	9	Traumatismo	2
1	04/10/2020	H16.0	1	Úlcera corneal	2

1	04/10/2020	H16.0	1	Úlcera corneal	2
2	04/10/2020	H10.81	0	Pingueculitis	2
1	04/10/2020	H16.9	1	Queratitis	2
1	04/10/2020	H10.3	0	Conjuntivitis	2
2	04/10/2020	H16.0	1	Úlcera corneal	2
2	04/10/2020	H16.0	1	Úlcera corneal	2
1	04/10/2020	H16.9	1	Queratitis	2
2	04/10/2020	T26	9	Causticación	2
2	04/10/2020	H16.9	1	Queratitis	2
1	04/10/2020	H49.9	10	Parálisis oculomotora	2
2	04/10/2020	H0.41	1	Ojo seco	2
1	04/10/2020	H16.0	1	Úlcera corneal	2
1	04/10/2020	T15.0	1	Cuerpo extraño	2
2	04/10/2020	T15.0	1	Cuerpo extraño	2
2	04/10/2020	H16.9	1	Queratitis	2
1	04/10/2020	H16.9	1	Queratitis	2
2	04/10/2020	H11.3	0	Hiposfagma	2
1	05/10/2020	T15.0	1	Cuerpo extraño	2
2	05/10/2020	T15.0	1	Cuerpo extraño	2
2	05/10/2020	H10.3	0	Conjuntivitis	2
1	05/10/2020	H35.3	8	Maculopatía	2
2	05/10/2020	H17.8	1	Infiltrados corneales	2
1	05/10/2020	H00.03	4	Celulitis preseptal	2
2	05/10/2020	H02	4	Alteración palpebral	2
1	05/10/2020	H16.9	1	Queratitis	2
2	05/10/2020	H16.9	1	Queratitis	2
1	05/10/2020	H10.3	0	Conjuntivitis	2
1	05/10/2020	H16.0	1	Úlcera corneal	2
2	05/10/2020	H33.0	8	Desprendimiento de retina	2
2	05/10/2020	H10.3	0	Conjuntivitis	2
1	05/10/2020	H27.8	2	OCP	2
2	05/10/2020	H40.05	3	HTO	2
1	05/10/2020	H10.3	0	Conjuntivitis	2
1	05/10/2020	S05.9	9	Traumatismo	2
2	05/10/2020	H43.1	7	Hemovítreo	2
1	05/10/2020	H16.9	1	Queratitis	2
1	05/10/2020	H16.0	1	Úlcera corneal	2
2	05/10/2020	H16.9	1	Queratitis	2
2	05/10/2020	H01.00	4	Blefaritis	2
2	05/10/2020	H01.00	4	Blefaritis	2
2	05/10/2020	H43.81	7	DVP	2
2	05/10/2020	H00.03	4	Celulitis preseptal	2
2	05/10/2020	H01.00	4	Blefaritis	2
2	05/10/2020	H16.0	1	Úlcera corneal	2
1	05/10/2020	G43.9	10	Migraña	2
1	05/10/2020	H15.1	6	Epiescleritis	2
2	05/10/2020	H43.81	7	DVP	2
2	05/10/2020	H16.0	1	Úlcera corneal	2
2	05/10/2020	H16.0	1	Úlcera corneal	2
2	05/10/2020	H16.9	1	Queratitis	2
2	05/10/2020	G43.9	10	Migraña	2
2	05/10/2020	H40.05	3	HTO	2
1	06/10/2020	T15.0	1	Cuerpo extraño	2
1	06/10/2020	H20	6	Uveitis	2
2	06/10/2020	H10.5	0	Blefaroconjuntivitis	2
1	06/10/2020	H10.3	0	Conjuntivitis	2
1	06/10/2020	H49.9	4	Parálisis oculomotora	2
1	06/10/2020	H31.42	8	Coroidopatía serosa central	2
2	06/10/2020	H43.81	7	DVP	2
2	06/10/2020	H10.3	0	Conjuntivitis	2
2	06/10/2020	H16.9	1	Queratitis	2
2	06/10/2020	H26.9	2	Catarata	2
2	06/10/2020	H00.19	4	Chalazión	2
2	06/10/2020	H10.3	0	Conjuntivitis	2
2	06/10/2020	H20	6	Uveitis	2
2	06/10/2020	H01.00	4	Blefaritis	2
1	06/10/2020	H16.0	1	Úlcera corneal	2
1	06/10/2020	H0.41	1	Ojo seco	2
1	06/10/2020	H40.9	3	Glaucoma	2
2	06/10/2020	H53.10	11	Problema refractivo	2
2	06/10/2020	G43.9	10	Migraña	2

1	06/10/2020	H26.9	2	Catarata	2
1	06/10/2020	H43.81	7	DVP	2
2	06/10/2020	H50.9	10	Estrabismo	2
1	06/10/2020	H16.0	1	Úlcera corneal	2
2	06/10/2020	H10.3	0	Conjuntivitis	2
2	06/10/2020	H16.0	1	Úlcera corneal	2
1	07/10/2020	H16.0	1	Úlcera corneal	2
1	07/10/2020	H16.9	1	Queratitis	2
1	07/10/2020	H49.9	4	Parálisis oculomotora	2
1	07/10/2020	T15.0	1	Cuerpo extraño	2
2	07/10/2020	B00.1	4	Dermatitis herpética	2
1	07/10/2020	H43.81	7	DVP	2
1	07/10/2020	H16.9	1	Queratitis	2
2	07/10/2020	H16.9	1	Queratitis	2
1	07/10/2020	H50.9	10	Estrabismo	2
1	07/10/2020	H26.9	2	Catarata	2
2	07/10/2020	H10.3	0	Conjuntivitis	2
1	07/10/2020	H10.3	0	Conjuntivitis	2
1	07/10/2020	H27.8	2	OCP	2
1	07/10/2020	H16.9	1	Queratitis	2
1	07/10/2020	H43.81	7	DVP	2
1	07/10/2020	T15.0	1	Cuerpo extraño	2
2	07/10/2020	H35.3	8	Maculopatía	2
1	07/10/2020	H16.0	1	Úlcera corneal	2
2	07/10/2020	H53.10	11	Problema refractivo	2
2	07/10/2020	H43.81	7	DVP	2
2	07/10/2020	H27.8	2	OCP	2
2	07/10/2020	H43.81	7	DVP	2
1	07/10/2020	T15.0	1	Cuerpo extraño	2
2	07/10/2020	H16.9	1	Queratitis	2
2	07/10/2020	H02	4	Alteración palpebral	2
1	07/10/2020	H10.3	0	Conjuntivitis	2
1	07/10/2020	Alta por fuga	11	Alta por fuga	2
1	07/10/2020	H0.41	1	Ojo seco	2
2	07/10/2020	H43.81	7	DVP	2
1	07/10/2020	H16.0	1	Úlcera corneal	2
1	07/10/2020	H16.9	1	Queratitis	2
1	07/10/2020	S05.9	9	Traumatismo	2
2	07/10/2020	H0.41	1	Ojo seco	2
1	07/10/2020	H10.3	0	Conjuntivitis	2
2	07/10/2020	H47.32	8	Drusas de nervio óptico	2
1	07/10/2020	H16.0	1	Úlcera corneal	2
1	08/10/2020	T15.0	1	Cuerpo extraño	2
2	08/10/2020	H35.3	8	Maculopatía	2
1	08/10/2020	H01.00	4	Blefaritis	2
2	08/10/2020	H43.81	7	DVP	2
2	08/10/2020	H11.3	0	Hiposfagma	2
1	08/10/2020	H34.82	8	Trombosis venosa	2
2	08/10/2020	H10.3	0	Conjuntivitis	2
1	08/10/2020	H16.9	1	Queratitis	2
2	08/10/2020	S05.9	9	Traumatismo	2
1	08/10/2020	H10.3	0	Conjuntivitis	2
1	08/10/2020	H43.81	7	DVP	2
1	08/10/2020	H01.00	4	Blefaritis	2
2	08/10/2020	H01.00	4	Blefaritis	2
1	08/10/2020	H53.10	11	Problema refractivo	2
2	08/10/2020	Alta por fuga	11	Alta por fuga	2
2	08/10/2020	H01.00	4	Blefaritis	2
2	08/10/2020	H43.81	7	DVP	2
2	08/10/2020	H11.3	0	Hiposfagma	2
1	08/10/2020	H53	11	No patología	2
2	08/10/2020	H10.3	0	Conjuntivitis	2
1	08/10/2020	H11.3	0	Hiposfagma	2
2	08/10/2020	H43.81	7	DVP	2
1	08/10/2020	H10.3	0	Conjuntivitis	2
1	08/10/2020	H10.3	0	Conjuntivitis	2
1	08/10/2020	S05.6	9	Perforación ocular	2
1	08/10/2020	H16.9	1	Queratitis	2
2	08/10/2020	H00.19	4	Chalazión	2
2	08/10/2020	H49.9	10	Parálisis oculomotora	2
2	08/10/2020	H35.30	8	DMAE	2

2	08/10/2020	H16.9	1	Queratitis	2
2	08/10/2020	H16.0	1	Úlcera corneal	2
2	08/10/2020	H16.9	1	Queratitis	2
1	08/10/2020	H16.9	1	Queratitis	2
2	09/10/2020	H16.9	1	Queratitis	2
2	09/10/2020	H16.0	1	Úlcera corneal	2
2	09/10/2020	H16.9	1	Queratitis	2
2	09/10/2020	H16.9	1	Queratitis	2
1	09/10/2020	H10.3	0	Conjuntivitis	2
2	09/10/2020	H16.9	1	Queratitis	2
2	09/10/2020	H16.9	1	Queratitis	2
1	09/10/2020	H33.0	8	Desprendimiento de retina	2
1	09/10/2020	H16.0	1	Úlcera corneal	2
2	09/10/2020	H10.3	0	Conjuntivitis	2
2	09/10/2020	H43.81	7	DVP	2
1	09/10/2020	G45.3	10	Amaurosis fugax	2
2	09/10/2020	H53	4	No patología	2
1	09/10/2020	T15.0	1	Cuerpo extraño	2
2	09/10/2020	H16.9	1	Queratitis	2
2	09/10/2020	H43.1	7	Hemovítreo	2
2	09/10/2020	H01.00	4	Blefaritis	2
2	09/10/2020	H20	6	Uveitis	2
1	09/10/2020	S05.9	9	Traumatismo	2
1	09/10/2020	T26	9	Causticación	2
2	09/10/2020	H10.3	0	Conjuntivitis	2
1	09/10/2020	H16.0	1	Úlcera corneal	2
1	09/10/2020	H43.81	7	DVP	2
1	10/10/2020	H16.9	1	Queratitis	2
1	10/10/2020	H16.0	1	Úlcera corneal	2
1	10/10/2020	H59.3	5	Postop via lagrimal	2
1	10/10/2020	T15.0	1	Cuerpo extraño	2
1	10/10/2020	H02	9	Alteración palpebral	2
1	10/10/2020	H16.0	1	Úlcera corneal	2
1	10/10/2020	H20	6	Uveitis	2
1	10/10/2020	H16.0	1	Úlcera corneal	2
2	10/10/2020	H01.00	4	Blefaritis	2
1	10/10/2020	H16.0	1	Úlcera corneal	2
1	10/10/2020	H10.3	0	Conjuntivitis	2
2	10/10/2020	H11.3	0	Hiposfagma	2
2	10/10/2020	T15.0	1	Cuerpo extraño	2
2	10/10/2020	H00.02	4	Orzuelo	2
2	10/10/2020	H53	4	No patología	2
2	10/10/2020	H02	4	Alteración palpebral	2
1	10/10/2020	T15.0	1	Cuerpo extraño	2
2	10/10/2020	H05.01	4	Celulitis orbitaria	2
1	10/10/2020	H00.02	4	Orzuelo	2
2	10/10/2020	H43.81	7	DVP	2
1	10/10/2020	T15.0	0	Cuerpo extraño	2
2	10/10/2020	H15.1	6	Epiescleritis	2
2	11/10/2020	H16.9	1	Queratitis	2
1	11/10/2020	H10.81	0	Pingueculitis	2
1	11/10/2020	H20	6	Uveitis	2
2	11/10/2020	H43.81	7	DVP	2
1	11/10/2020	T15.0	1	Cuerpo extraño	2
1	11/10/2020	H16.0	1	Úlcera corneal	2
1	11/10/2020	H11.3	0	Hiposfagma	2
1	11/10/2020	H20	6	Uveitis	2
2	11/10/2020	H16.9	1	Queratitis	2
1	11/10/2020	H10.3	0	Conjuntivitis	2
1	11/10/2020	H20	6	Uveitis	2
2	11/10/2020	S05.9	9	Traumatismo	2
2	11/10/2020	S05.9	9	Traumatismo	2
1	11/10/2020	S05.9	9	Traumatismo	2
1	11/10/2020	S05.9	9	Traumatismo	2
2	11/10/2020	H35.30	8	DMAE	2
2	11/10/2020	H33.0	8	Desprendimiento de retina	2
2	11/10/2020	H15.1	6	Epiescleritis	2
1	11/10/2020	H16.9	1	Queratitis	2
2	11/10/2020	H10.3	0	Conjuntivitis	2
2	11/10/2020	H00.03	4	Celulitis preseptal	2
2	11/10/2020	H16.9	1	Queratitis	2

2	11/10/2020	H16.9		1	Queratitis	2
2	12/10/2020	H11.3		0	Hiposfagma	2
1	12/10/2020	H10.3		0	Conjuntivitis	2
1	12/10/2020	H16.0		1	Úlcera corneal	2
2	12/10/2020	H02		4	Alteración palpebral	2
2	12/10/2020	H33.30		8	Desgarro retiniano	2
1	12/10/2020	H35.0		8	Hemorragia retiniana	2
2	12/10/2020	H43.81		7	DVP	2
2	12/10/2020	H16.0		1	Úlcera corneal	2
1	12/10/2020	H10.3		0	Conjuntivitis	2
1	12/10/2020	H20		6	Uveitis	2
1	12/10/2020	H0.41		1	Ojo seco	2
1	12/10/2020	G43.9		10	Migraña	2
2	12/10/2020	H16.9		1	Queratitis	2
2	12/10/2020	H20		6	Uveitis	2
2	12/10/2020	H10.3		0	Conjuntivitis	2
2	12/10/2020	S05.9		9	Traumatismo	2
1	12/10/2020	H30.9		6	Uveitis posterior	2
2	12/10/2020	H10.3		0	Conjuntivitis	2
2	12/10/2020	H16.9		1	Queratitis	2
1	12/10/2020	H10.3		0	Conjuntivitis	2
2	12/10/2020	H10.3		0	Conjuntivitis	2
1	12/10/2020	T15.0		1	Cuerpo extraño	2
1	12/10/2020	H11.3		0	Hiposfagma	2
1	13/10/2020	H10.3		0	Conjuntivitis	2
1	13/10/2020	H27.8		2	OCP	2
2	13/10/2020	H10.3		0	Conjuntivitis	2
2	13/10/2020	H10.3		0	Conjuntivitis	2
1	13/10/2020	H16.9		1	Queratitis	2
1	13/10/2020	H16.9		1	Queratitis	2
1	13/10/2020	H02		4	Alteración palpebral	2
1	13/10/2020	H01.00		4	Blefaritis	2
2	13/10/2020	H27.8		2	OCP	2
2	13/10/2020	H0.41		1	Ojo seco	2
1	13/10/2020	H11.3		0	Hiposfagma	2
2	13/10/2020	H16.9		1	Queratitis	2
2	13/10/2020	H53.10		11	Problema refractivo	2
1	13/10/2020	T15.0		1	Cuerpo extraño	2
2	13/10/2020	H01.00		4	Blefaritis	2
2	13/10/2020	H00.02		4	Orzuelo	2
2	13/10/2020	H10.3		0	Conjuntivitis	2
2	14/10/2020	H10.3		0	Conjuntivitis	2
1	14/10/2020	H43.81		7	DVP	2
2	14/10/2020	B00.1		4	Dermatitis herpética	2
2	14/10/2020	H43.1		7	Hemovítreo	2
2	14/10/2020	S05.9		9	Traumatismo	2
2	14/10/2020	S05.9		9	Traumatismo	2
2	14/10/2020	H10.3		0	Conjuntivitis	2
1	14/10/2020	T15.0		1	Cuerpo extraño	2
2	14/10/2020	H01.00		4	Blefaritis	2
2	14/10/2020	H10.3		0	Conjuntivitis	2
2	14/10/2020	H53.10		11	Problema refractivo	2
1	14/10/2020	B02.29		11	Neuralgia herpética	2
2	14/10/2020	H11.3		0	Hiposfagma	2
2	14/10/2020	H10.3		0	Conjuntivitis	2
2	14/10/2020	H16.0		1	Úlcera corneal	2
1	14/10/2020	H10.5		4	Blefaroconjuntivitis	2
2	14/10/2020	H53.10		11	Problema refractivo	2
1	14/10/2020	H16.0		1	Úlcera corneal	2
2	14/10/2020	H43.81		7	DVP	2
2	14/10/2020	H16.9		1	Queratitis	2
2	14/10/2020	H10.3		0	Conjuntivitis	2
1	14/10/2020	H16.9		1	Queratitis	2
1	14/10/2020	H20		6	Uveitis	2
1	14/10/2020	T15.0		1	Cuerpo extraño	2
1	14/10/2020	H16.9		1	Queratitis	2
2	14/10/2020	H16.9		1	Queratitis	2
2	14/10/2020	H16.9		1	Queratitis	2
2	14/10/2020	H43.81		7	DVP	2
2	14/10/2020	G43.9		10	Migraña	2
1	14/10/2020	H11.3		0	Hiposfagma	2

1	14/10/2020	H43.81	7	DVP	2
2	14/10/2020	H16.0	1	Úlcera corneal	2
2	14/10/2020	H01.00	4	Blefaritis	2
1	15/10/2020	H16.0	1	Úlcera corneal	2
1	15/10/2020	S05.9	9	Traumatismo	2
1	15/10/2020	T15.0	1	Cuerpo extraño	2
2	15/10/2020	S05.6	9	Perforación ocular	2
2	15/10/2020	H01.00	4	Blefaritis	2
1	15/10/2020	H16.0	1	úlcera corneal	2
2	15/10/2020	H00.02	4	Orzuelo	2
1	15/10/2020	H00.19	4	Chalazión	2
1	15/10/2020	H11.3	0	Hiposfagma	2
2	15/10/2020	H10.3	0	Conjuntivitis	2
2	15/10/2020	S05.9	9	Traumatismo	2
1	15/10/2020	H43.81	7	DVP	2
2	15/10/2020	H01.00	4	Blefaritis	2
1	15/10/2020	H16.9	1	Queratitis	2
2	15/10/2020	H20	6	Uveitis	2
1	16/10/2020	H10.3	0	Conjuntivitis	2
2	16/10/2020	H27.8	2	OCP	2
2	16/10/2020	H10.3	0	Conjuntivitis	2
2	16/10/2020	H17.8	1	Infiltrados corneales	2
2	16/10/2020	H20	6	Uveitis	2
2	16/10/2020	H01.00	4	Blefaritis	2
2	16/10/2020	H43.81	7	DVP	2
2	16/10/2020	H00.02	4	Orzuelo	2
2	16/10/2020	H16.0	1	Úlcera corneal	2
1	16/10/2020	H27.8	2	OCP	2
2	16/10/2020	H43.81	7	DVP	2
1	16/10/2020	T15.0	1	Cuerpo extraño	2
1	16/10/2020	H16.0	1	Úlcera corneal	2
2	16/10/2020	T15.0	1	Cuerpo extraño	2
2	16/10/2020	H16.9	1	Queratitis	2
1	16/10/2020	H33.30	8	Desgarro retiniano	2
2	16/10/2020	H11.3	0	Hiposfagma	2
1	16/10/2020	H43.81	7	DVP	2
1	16/10/2020	H16.9	1	Queratitis	2
1	16/10/2020	T15.0	1	Cuerpo extraño	2
2	16/10/2020	H00.02	4	Orzuelo	2
2	16/10/2020	H15.1	6	Epiescleritis	2
2	16/10/2020	H11.3	0	Hiposfagma	2
2	16/10/2020	H20	6	Uveitis	2
2	16/10/2020	H16.0	1	Úlcera corneal	2
2	16/10/2020	H11.3	0	Hiposfagma	2
2	17/10/2020	H10.3	0	Conjuntivitis	2
1	17/10/2020	H16.0	1	Úlcera corneal	2
1	17/10/2020	H35.3	8	Maculopatía	2
1	17/10/2020	H16.9	1	Queratitis	2
1	17/10/2020	H10.3	0	Conjuntivitis	2
1	17/10/2020	H11.3	0	Hiposfagma	2
1	17/10/2020	H00.02	4	Orzuelo	2
2	17/10/2020	G43.9	10	Migraña	2
1	17/10/2020	H01.00	4	Blefaritis	2
1	17/10/2020	H43.81	7	DVP	2
1	17/10/2020	S05.9	9	Traumatismo	2
2	17/10/2020	H16.0	1	Úlcera corneal	2
2	17/10/2020	H10.3	0	Conjuntivitis	2
2	17/10/2020	H16.0	1	Úlcera corneal	2
1	17/10/2020	T15.0	1	Cuerpo extraño	2
2	17/10/2020	H20	6	Uveitis	2
2	17/10/2020	H16.9	1	Queratitis	2
2	17/10/2020	H43.81	7	DVP	2
2	17/10/2020	H16.0	1	Úlcera corneal	2
1	17/10/2020	H43.81	7	DVP	2
1	17/10/2020	H00.02	4	Orzuelo	2
2	17/10/2020	H43.81	7	DVP	2
1	17/10/2020	H16.0	1	úlcera corneal	2
1	17/10/2020	T15.0	1	Cuerpo extraño	2
1	17/10/2020	H01.00	4	Blefaritis	2
1	18/10/2020	B00.1	4	Dermatitis herpética	2
2	18/10/2020	H16.9	1	Queratitis	2

1	18/10/2020	H16.0	1	Úlcera corneal	2
2	18/10/2020	B02.29	4	Neuralgia herpética	2
1	18/10/2020	H10.3	0	Conjuntivitis	2
2	18/10/2020	H10.3	0	Conjuntivitis	2
2	18/10/2020	H16.9	1	Queratitis	2
1	18/10/2020	H16.0	1	Úlcera corneal	2
2	18/10/2020	H01.00	4	Blefaritis	2
1	18/10/2020	E11.319	8	Retinopatía diabética	2
1	18/10/2020	H10.3	0	Conjuntivitis	2
1	18/10/2020	H10.3	0	Conjuntivitis	2
1	18/10/2020	H20	6	Uveitis	2
2	18/10/2020	H01.00	4	Blefaritis	2
2	18/10/2020	H10.3	0	Conjuntivitis	2
1	18/10/2020	H00.19	4	Chalazión	2
1	18/10/2020	S05.9	9	Traumatismo	2
1	18/10/2020	H16.0	1	Úlcera corneal	2
2	18/10/2020	H16.0	1	Úlcera corneal	2
1	18/10/2020	T15.0	1	Cuerpo extraño	2
2	19/10/2020	H43.81	7	DVP	2
2	19/10/2020	H10.3	0	Conjuntivitis	2
2	19/10/2020	H0.41	1	ojo seco	2
1	19/10/2020	T15.0	1	Cuerpo extraño	2
2	19/10/2020	H16.9	1	Queratitis	2
2	19/10/2020	H16.9	1	Queratitis	2
2	19/10/2020	H10.3	0	Conjuntivitis	2
2	19/10/2020	H43.81	7	DVP	2
1	19/10/2020	H16.0	0	Úlcera corneal	2
2	19/10/2020	H10.3	0	Conjuntivitis	2
1	19/10/2020	E11.319	8	Retinopatía diabética	2
1	19/10/2020	S05.9	9	Traumatismo	2
2	19/10/2020	H0.41	1	Ojo seco	2
1	19/10/2020	H20	6	Uveitis	2
2	19/10/2020	H10.3	0	Conjuntivitis	2
2	19/10/2020	H16.9	1	Queratitis	2
1	19/10/2020	H43.81	7	DVP	2
2	19/10/2020	T15.0	1	Cuerpo extraño	2
2	19/10/2020	H53.9	11	Alteraciones visuales	2
1	19/10/2020	H11.3	0	Hiposfagma	2
2	19/10/2020	H43.81	7	DVP	2
1	19/10/2020	T15.0	1	Cuerpo extraño	2
1	19/10/2020	T15.0	1	Cuerpo extraño	2
2	19/10/2020	H43.81	7	DVP	2
2	19/10/2020	H10.81	0	Pingueculitis	2
2	20/10/2020	H16.9	1	Queratitis	2
2	20/10/2020	H16.0	1	Úlcera corneal	2
2	20/10/2020	H16.9	1	Queratitis	2
1	20/10/2020	H10.3	0	Conjuntivitis	2
1	20/10/2020	H26.9	2	Catarata	2
2	20/10/2020	H16.9	1	Queratitis	2
2	20/10/2020	H16.3	1	Abceso corneal	2
1	20/10/2020	H46	10	Neuritis óptica	2
2	20/10/2020	H10.3	0	Conjuntivitis	2
1	20/10/2020	H02	4	Alteración palpebral	2
2	20/10/2020	H01.00	4	Blefaritis	2
2	20/10/2020	H17.8	1	Infiltrados corneales	2
1	20/10/2020	H00.02	4	Orzuelo	2
2	20/10/2020	H10.3	0	Conjuntivitis	2
1	20/10/2020	H34.9	8	Oclusión arterial	2
2	20/10/2020	H43.81	7	DVP	2
2	20/10/2020	H43.81	7	DVP	2
1	20/10/2020	T15.0	1	Cuerpo extraño	2
1	20/10/2020	H00.03	4	Celulitis preseptal	2
2	20/10/2020	H11.3	0	Hiposfagma	2
1	20/10/2020	H20	6	Uveitis	2
1	20/10/2020	H10.3	0	Conjuntivitis	2
2	20/10/2020	H16.9	1	Queratitis	2
2	20/10/2020	H53.10	11	Problema refractivo	2
2	20/10/2020	H11.3	0	Hiposfagma	2
1	20/10/2020	T15.0	1	Cuerpo extraño	2
1	20/10/2020	H16.0	1	Úlcera corneal	2
1	20/10/2020	S05.6	9	Perforación ocular	2

1	20/10/2020	T15.0	1	Cuerpo extraño	2
1	20/10/2020	H16.9	1	Queratitis	2
1	21/10/2020	S05.9	9	Traumatismo	2
1	21/10/2020	H00.03	4	Celulitis preseptal	2
2	21/10/2020	H00.02	4	Orzuelo	2
1	21/10/2020	H27.8	2	OCP	2
2	21/10/2020	H00.02	4	Orzuelo	2
2	21/10/2020	H43.81	7	DVP	2
2	21/10/2020	H10.3	0	Conjuntivitis	2
1	21/10/2020	Z98.4	11	Post op catarata	2
2	21/10/2020	H01.00	4	Blefaritis	2
1	21/10/2020	H43.81	7	DVP	2
2	21/10/2020	H00.02	4	Orzuelo	2
2	21/10/2020	H10.3	0	Conjuntivitis	2
2	21/10/2020	H17.8	1	Infiltrados corneales	2
1	21/10/2020	H43.81	7	DVP	2
2	21/10/2020	H01.00	4	Blefaritis	2
2	21/10/2020	H16.9	1	Queratitis	2
2	21/10/2020	H27.8	2	OCP	2
2	21/10/2020	H16.0	1	Úlcera corneal	2
2	21/10/2020	H20	6	Uveitis	2
1	21/10/2020	H16.9	1	Queratitis	2
2	21/10/2020	H16.9	1	Queratitis	2
1	21/10/2020	H16.9	1	Queratitis	2
1	21/10/2020	T15.0	1	Cuerpo extraño	2
2	21/10/2020	H11.3	0	Hiposfagma	2
2	21/10/2020	S05.9	9	Traumatismo	2
1	21/10/2020	T15.0	1	Cuerpo extraño	2
1	21/10/2020	H43.81	7	DVP	2
1	22/10/2020	H20	6	Uveitis	2
2	22/10/2020	H47.32	8	Drusas de nervio óptico	2
2	22/10/2020	B00.1	4	Dermatitis herpética	2
1	22/10/2020	H10.3	0	Conjuntivitis	2
2	22/10/2020	S05.9	9	Traumatismo	2
1	22/10/2020	H43.81	7	DVP	2
1	22/10/2020	H33.30	8	Desgarro retiniano	2
2	22/10/2020	H27.8	2	OCP	2
1	22/10/2020	H43.81	7	DVP	2
1	22/10/2020	H00.02	4	Orzuelo	2
2	22/10/2020	H11.3	0	Hiposfagma	2
2	22/10/2020	H16.9	1	Queratitis	2
2	22/10/2020	H16.9	1	Queratitis	2
1	22/10/2020	H18.50	1	Distrofia corneal	2
2	22/10/2020	H43.81	7	DVP	2
1	22/10/2020	H16.0	1	Úlcera corneal	2
1	22/10/2020	H10.3	0	Conjuntivitis	2
2	22/10/2020	H16.0	1	Úlcera corneal	2
2	22/10/2020	H16.0	1	úlcera corneal	2
1	22/10/2020	T15.0	1	Cuerpo extraño	2
1	22/10/2020	H10.3	0	Conjuntivitis	2
1	22/10/2020	H16.0	1	Úlcera corneal	2
1	22/10/2020	T15.0	1	Cuerpo extraño	2
2	23/10/2020	H16.9	1	Queratitis	2
1	23/10/2020	H43.1	7	Hemovítreo	2
1	23/10/2020	H01.00	4	Blefaritis	2
1	23/10/2020	H16.9	1	Queratitis	2
2	23/10/2020	H15.1	6	Epiescleritis	2
1	23/10/2020	H0.41	1	ojo seco	2
2	23/10/2020	B00.1	4	Dermatitis herpética	2
2	23/10/2020	H33.30	8	Desgarro retiniano	2
2	23/10/2020	H01.00	4	Blefaritis	2
1	23/10/2020	H34.82	8	Trombosis venosa	2
2	23/10/2020	H11.3	0	Hiposfagma	2
1	23/10/2020	H34.82	8	Trombosis venosa	2
1	23/10/2020	H33.30	8	Desgarro retiniano	2
2	23/10/2020	H33.0	8	Desprendimiento de retina	2
1	23/10/2020	H16.9	1	Queratitis	2
1	23/10/2020	T15.0	1	Cuerpo extraño	2
1	23/10/2020	H11.3	0	Hiposfagma	2
1	23/10/2020	H20	6	Uveitis	2
1	23/10/2020	H11.3	0	Hiposfagma	2

2	23/10/2020	H0.41	1	ojo seco	2
1	23/10/2020	H11.3	0	Hiposfagma	2
1	23/10/2020	H27.8	2	OCP	2
2	23/10/2020	T15.0	1	Cuerpo extraño	2
1	23/10/2020	T15.0	1	Cuerpo extraño	2
2	23/10/2020	H0.41	1	Ojo seco	2
2	23/10/2020	H20	6	Uveitis	2
2	23/10/2020	G43.9	10	Migraña	2
1	23/10/2020	T26	9	Causticación	2
1	24/10/2020	H10.3	0	Conjuntivitis	2
2	24/10/2020	B02.29	4	Neuralgia herpética	2
1	24/10/2020	H16.9	1	Queratitis	2
1	24/10/2020	H43.81	7	DVP	2
2	24/10/2020	H16.0	1	Úlcera corneal	2
2	24/10/2020	H20	6	Uveitis	2
1	24/10/2020	H20	6	Uveitis	2
1	24/10/2020	H11.3	0	Hiposfagma	2
1	24/10/2020	H33.0	8	Desprendimiento de retina	2
1	24/10/2020	H11.3	0	Hiposfagma	2
1	24/10/2020	H43.81	7	DVP	2
1	24/10/2020	H43.81	7	DVP	2
1	24/10/2020	H16.0	1	Úlcera corneal	2
2	24/10/2020	H0.41	1	Ojo seco	2
2	24/10/2020	H20	6	Uveitis	2
2	24/10/2020	G43.9	10	Migraña	2
1	24/10/2020	H43.81	7	DVP	2
2	24/10/2020	H43.1	7	Hemovítreo	2
1	24/10/2020	H16.9	1	Queratitis	2
1	24/10/2020	H00.02	4	Orzuelo	2
2	24/10/2020	H04.32	5	Dacriocistitis aguda	2
1	24/10/2020	H43.1	7	Hemovítreo	2
1	24/10/2020	H16.0	1	Úlcera corneal	2
1	24/10/2020	H46	10	Neuritis óptica	2
1	24/10/2020	H02	4	Alteración palpebral	2
1	25/10/2020	H16.0	1	Úlcera corneal	2
1	25/10/2020	H16.9	1	Queratitis	2
2	25/10/2020	H11.3	0	Hiposfagma	2
1	25/10/2020	T15.0	1	Cuerpo extraño	2
2	25/10/2020	H16.0	1	Úlcera corneal	2
1	25/10/2020	H11.00	0	Pterigium	2
2	25/10/2020	H17.8	1	Infiltrados corneales	2
1	25/10/2020	H16.9	1	Queratitis	2
1	25/10/2020	H04.32	5	Dacriocistitis aguda	2
1	25/10/2020	H27.10	2	Luxación LIO	2
2	25/10/2020	G43.9	10	Migraña	2
2	25/10/2020	H18.20	1	Edema corneal	2
2	25/10/2020	H33.0	8	Desprendimiento de retina	2
1	25/10/2020	H16.0	1	Úlcera corneal	2
2	25/10/2020	H0.41	1	Ojo seco	2
1	25/10/2020	T15.0	1	Cuerpo extraño	2
1	25/10/2020	H16.0	1	Úlcera corneal	2
2	25/10/2020	S05.9	9	Traumatismo	2
2	25/10/2020	T15.0	1	Cuerpo extraño	2
1	25/10/2020	S05.9	9	Traumatismo	2
1	25/10/2020	T15.0	1	Cuerpo extraño	2
1	26/10/2020	T15.0	1	Cuerpo extraño	2
2	26/10/2020	H27.8	2	OCP	2
2	26/10/2020	H10.3	0	Conjuntivitis	2
2	26/10/2020	H16.9	1	Queratitis	2
1	26/10/2020	H11.3	0	Hiposfagma	2
2	26/10/2020	H16.0	1	Úlcera corneal	2
2	26/10/2020	H43.81	7	DVP	2
2	26/10/2020	H40.05	3	HTO	2
1	26/10/2020	H00.02	4	Orzuelo	2
2	26/10/2020	H01.00	4	Blefaritis	2
2	26/10/2020	H53.10	11	Problema refractivo	2
1	26/10/2020	H16.9	1	Queratitis	2
2	26/10/2020	H43.81	7	DVP	2
2	26/10/2020	H11.3	0	Hiposfagma	2
1	26/10/2020	H16.0	1	Úlcera corneal	2
1	26/10/2020	H00.02	4	Orzuelo	2

2	26/10/2020	H16.9		1	Queratitis	2
2	26/10/2020	H20		6	Uveitis	2
2	26/10/2020	H16.0		1	Úlcera corneal	2
2	26/10/2020	H16.0		1	úlcera corneal	2
1	26/10/2020	H02		4	Alteración palpebral	2
2	26/10/2020	H16.9		1	Queratitis	2
2	26/10/2020	H20		6	Uveitis	2
1	26/10/2020	H18.50		1	Distrofia corneal	2
2	26/10/2020	G43.9		10	Migraña	2
1	26/10/2020	S05.6		9	Perforación ocular	2
1	27/10/2020	H16.0		1	Úlcera corneal	2
1	27/10/2020	H01.00		4	Blefaritis	2
1	27/10/2020	H35.30		8	DMAE	2
1	27/10/2020	H02		4	Lesión palpebral	2
1	27/10/2020	H43.1		7	Hemovítreo	2
2	27/10/2020	H35.30		8	DMAE	2
2	27/10/2020	H16.9		1	Queratitis	2
1	27/10/2020	T15.0		1	Cuerpo extraño	2
1	27/10/2020	H00.19		4	Chalazión	2
1	27/10/2020	H0.41		1	Ojo seco	2
2	27/10/2020	H16.9		1	Queratitis	2
2	27/10/2020	H11.3		0	Hiposfagma	2
2	27/10/2020	H10.5		0	Blefaroconjuntivitis	2
2	27/10/2020	Alta por fuga		11	Alta por fuga	2
2	27/10/2020	H20		6	Uveitis	2
2	27/10/2020	H0.41		1	ojo seco	2
2	27/10/2020	H16.0		1	Úlcera corneal	2
2	27/10/2020	H00.02		4	Orzuelo	2
2	27/10/2020	H01.00		4	Blefaritis	2
2	27/10/2020	G43.9		10	Migraña	2
2	27/10/2020	H53		11	No patología	2
2	27/10/2020	H16.9		1	Queratitis	2
2	27/10/2020	S05.6		9	Perforación ocular	2
1	27/10/2020	H33.0		8	Desprendimiento de retina	2
1	27/10/2020	H43.81		7	DVP	2
2	28/10/2020	H16.9		1	Queratitis	2
1	28/10/2020	T15.0		1	Cuerpo extraño	2
1	28/10/2020	H10.3		0	Conjuntivitis	2
1	28/10/2020	H17.8		1	Infiltrados corneales	2
2	28/10/2020	H02		4	Alteración palpebral	2
2	28/10/2020	H10.3		0	Conjuntivitis	2
1	28/10/2020	H16.9		1	Queratitis	2
2	28/10/2020	H0.41		1	Ojo seco	2
2	28/10/2020	H43.81		7	DVP	2
2	28/10/2020	H16.9		1	Queratitis	2
1	28/10/2020	H00.02		4	Orzuelo	2
2	28/10/2020	H43.81		7	DVP	2
2	28/10/2020	H16.9		1	Queratitis	2
2	28/10/2020	H10.3		0	Conjuntivitis	2
1	28/10/2020	H20		6	Uveitis	2
1	28/10/2020	H20		6	Uveitis	2
2	28/10/2020	H11.44		0	Quiste conjuntival	2
2	28/10/2020	H0.41		1	Ojo seco	2
2	28/10/2020	E11.319		8	Retinopatía diabética	2
2	28/10/2020	H27.8		2	OCP	2
1	28/10/2020	H16.0		1	Úlcera corneal	2
1	28/10/2020	H15.00		6	Escleritis	2
1	28/10/2020	T15.0		1	Cuerpo extraño	2
1	28/10/2020	H49.9		10	Parálisis oculomotora	2
1	28/10/2020	H20		6	Uveitis	2
2	28/10/2020	H16.9		1	Queratitis	2
1	28/10/2020	H43.81		7	DVP	2
1	28/10/2020	Z98.4		2	Post op catarata	2
1	28/10/2020	H00.02		4	Orzuelo	2
1	28/10/2020	H16.0		1	Úlcera corneal	2
2	28/10/2020	H16.0		1	Úlcera corneal	2
1	28/10/2020	H00.02		4	Orzuelo	2
1	29/10/2020	T15.0		1	Cuerpo extraño	2
1	29/10/2020	S05.9		9	Traumatismo	2
2	29/10/2020	H15.1		6	Epiescleritis	2
2	29/10/2020	H35.30		8	DMAE	2

1	29/10/2020	H01.00	4	Blefaritis	2
2	29/10/2020	H16.9	1	Queratitis	2
1	29/10/2020	H16.0	1	Úlcera corneal	2
1	29/10/2020	S05.30	0	Laceración conjuntival	2
2	29/10/2020	H10.5	0	Blefaroconjuntivitis	2
1	29/10/2020	H00.02	4	Orzuelo	2
1	29/10/2020	H43.81	7	DVP	2
2	29/10/2020	H10.3	0	Conjuntivitis	2
2	29/10/2020	H20	6	Uveitis	2
2	29/10/2020	H16.9	1	Queratitis	2
2	29/10/2020	H01.00	4	Blefaritis	2
1	29/10/2020	T15.0	1	Cuerpo extraño	2
1	29/10/2020	H16.0	1	Úlcera corneal	2
1	29/10/2020	H16.9	1	Queratitis	2
1	29/10/2020	T15.0	1	Cuerpo extraño	2
1	29/10/2020	H16.9	1	Queratitis	2
1	29/10/2020	H40.05	3	HTO	2
2	30/10/2020	H10.3	0	Conjuntivitis	2
1	30/10/2020	H00.02	4	Orzuelo	2
1	30/10/2020	H10.3	0	Conjuntivitis	2
2	30/10/2020	H00.02	4	Orzuelo	2
1	30/10/2020	S05.9	9	Traumatismo	2
1	30/10/2020	H16.9	1	Queratitis	2
1	30/10/2020	H11.3	0	Hiposfagma	2
1	30/10/2020	H01.00	4	Blefaritis	2
1	30/10/2020	T15.0	1	Cuerpo extraño	2
2	30/10/2020	H11.3	0	Hiposfagma	2
1	30/10/2020	H16.9	1	Queratitis	2
1	30/10/2020	H43.81	7	DVP	2
2	30/10/2020	H0.41	1	Ojo seco	2
1	30/10/2020	T15.0	1	Cuerpo extraño	2
1	30/10/2020	H35.3	8	Maculopatía	2
2	30/10/2020	H01.00	4	Blefaritis	2
2	30/10/2020	H01.00	4	Blefaritis	2
2	30/10/2020	H11.3	0	Hiposfagma	2
2	30/10/2020	H53.10	11	Problema refractivo	2
1	30/10/2020	T15.0	1	Cuerpo extraño	2
1	30/10/2020	T15.0	1	Cuerpo extraño	2
2	30/10/2020	G43.9	10	Migraña	2
1	30/10/2020	H01.00	4	Blefaritis	2
1	30/10/2020	T15.0	1	Cuerpo extraño	2
1	31/10/2020	S05.9	9	Traumatismo	2
1	31/10/2020	T15.0	1	Cuerpo extraño	2
1	31/10/2020	T15.0	1	Cuerpo extraño	2
1	31/10/2020	H16.9	1	Queratitis	2
1	31/10/2020	H43.81	7	DVP	2
2	31/10/2020	H44.009	8	Endoftalmitis	2
1	31/10/2020	G43.9	10	Migraña	2
2	31/10/2020	H10.3	0	Conjuntivitis	2
1	31/10/2020	H16.0	1	Úlcera corneal	2
1	31/10/2020	H10.3	0	Conjuntivitis	2
1	31/10/2020	H02	4	Alteración palpebral	2
2	31/10/2020	S05.6	9	Perforación ocular	2
1	31/10/2020	T15.0	1	Cuerpo extraño	2
2	31/10/2020	H16.9	1	Queratitis	2
2	31/10/2020	H10.3	0	Conjuntivitis	2
1	31/10/2020	H16.0	1	Úlcera corneal	2
2	31/10/2020	T26	9	Causticación	2
2	31/10/2020	H15.00	6	Escleritis	2
2	31/10/2020	H53	11	No patología	2
2	31/10/2020	H16.9	1	Queratitis	2
2	31/10/2020	H11.3	0	Hiposfagma	2
1	31/10/2020	T15.0	1	Cuerpo extraño	2
2	31/10/2020	H02	4	Alteración palpebral	2
2	31/10/2020	H16.0	1	Úlcera corneal	2
1	31/10/2020	T15.0	1	Cuerpo extraño	2
1	31/10/2020	H16.0	1	Úlcera corneal	2
1	31/10/2020	H16.9	1	Queratitis	2
1	31/10/2020	S05.9	9	Traumatismo	2
1	31/10/2020	T15.0	1	Cuerpo extraño	2
1	31/10/2020	H16.0	1	Úlcera corneal	2

1	31/10/2020	H16.0	1	Úlcera corneal	2
1	31/10/2020	T15.0	1	Cuerpo extraño	2
2	01/11/2020	S05.9	9	Traumatismo	2
2	01/11/2020	H16.9	1	Queratitis	2
1	01/11/2020	H16.9	1	Queratitis	2
1	01/11/2020	H20	6	Uveitis	2
2	01/11/2020	G43.9	10	Migraña	2
2	01/11/2020	H10.3	0	Conjuntivitis	2
1	01/11/2020	H16.9	1	Queratitis	2
1	01/11/2020	H10.3	0	Conjuntivitis	2
1	01/11/2020	H49.9	10	Parálisis oculomotora	2
2	01/11/2020	H11.3	0	Hiposfagma	2
2	01/11/2020	H10.5	0	Blefaroconjuntivitis	2
1	01/11/2020	H10.3	0	Conjuntivitis	2
2	01/11/2020	H15.1	6	Epiescleritis	2
1	01/11/2020	H10.3	0	Conjuntivitis	2
2	01/11/2020	H15.1	6	Epiescleritis	2
1	01/11/2020	H53.9	11	Alteraciones visuales	2
2	01/11/2020	H16.9	1	Queratitis	2
1	01/11/2020	H59.88	8	Post op retina	2
2	01/11/2020	H01.00	4	Blefaritis	2
2	01/11/2020	H16.9	1	Queratitis	2
1	01/11/2020	H11.3	0	Hiposfagma	2
2	01/11/2020	H11.3	0	Hiposfagma	2
1	01/11/2020	T15.0	1	Cuerpo extraño	2
2	01/11/2020	H10.3	0	Conjuntivitis	2
1	01/11/2020	H10.3	0	Conjuntivitis	2
2	01/11/2020	H04.32	5	Dacriocistitis aguda	2
1	01/11/2020	H11.3	0	Hiposfagma	2
1	01/11/2020	S05.9	9	Traumatismo	2
2	02/11/2020	H15.00	6	Escleritis	2
1	02/11/2020	H16.9	1	Queratitis	2
1	02/11/2020	H10.3	0	Conjuntivitis	2
1	02/11/2020	T15.0	1	Cuerpo extraño	2
1	02/11/2020	H16.0	1	Úlcera corneal	2
2	02/11/2020	H43.81	7	DVP	2
2	02/11/2020	H16.9	1	Queratitis	2
1	02/11/2020	H00.02	4	Orzuelo	2
1	02/11/2020	G43.9	10	Migraña	2
1	02/11/2020	S05.6	9	Perforación ocular	2
2	02/11/2020	H16.0	1	Úlcera corneal	2
2	02/11/2020	H04.32	5	Dacriocistitis aguda	2
2	02/11/2020	H00.03	4	Celulitis preseptal	2
1	02/11/2020	H11.3	0	Hiposfagma	2
1	02/11/2020	H59.88	8	Post op retina	2
2	02/11/2020	H11.3	0	Hiposfagma	2
2	02/11/2020	H18.20	1	Edema corneal	2
2	02/11/2020	H35.3	8	Maculopatía	2
2	02/11/2020	H20	6	Uveitis	2
2	02/11/2020	H16.0	1	Úlcera corneal	2
1	02/11/2020	H31.42	8	Coroidopatía serosa central	2
1	02/11/2020	T15.0	1	Cuerpo extraño	2
1	02/11/2020	H02	4	Alteración palpebral	2
2	02/11/2020	H11.3	0	Hiposfagma	2
1	02/11/2020	H16.9	1	Queratitis	2
2	02/11/2020	H26.9	2	Catarata	2
2	02/11/2020	H16.9	1	Queratitis	2
2	02/11/2020	H53.10	11	Problema refractivo	2
2	02/11/2020	H20	6	Uveitis	2
2	02/11/2020	H53	11	No patología	2
1	02/11/2020	H20	6	Uveitis	2
2	02/11/2020	H16.9	1	Queratitis	2
2	02/11/2020	H16.0	1	Úlcera corneal	2
1	02/11/2020	H11.00	0	Pterigium	2
1	02/11/2020	H43.1	7	Hemovítreo	2
2	02/11/2020	H11.00	0	Pterigium	2
2	02/11/2020	E11.319	8	Retinopatía diabética	2
2	02/11/2020	H43.81	7	DVP	2
2	02/11/2020	G43.9	10	Migraña	2
1	03/11/2020	H16.9	1	Queratitis	2
1	03/11/2020	H00.02	4	Orzuelo	2

1	03/11/2020	H16.0	1	Úlcera corneal	2
2	03/11/2020	H01.00	4	Blefaritis	2
2	03/11/2020	H11.3	0	Hiposfagma	2
2	03/11/2020	H16.0	1	Úlcera corneal	2
2	03/11/2020	H27.10	2	Luxación LIO	2
2	03/11/2020	H10.3	0	Conjuntivitis	2
2	03/11/2020	H35.30	8	DMAE	2
1	03/11/2020	H10.3	0	Conjuntivitis	2
1	03/11/2020	H16.9	1	Queratitis	2
2	03/11/2020	H10.3	0	Conjuntivitis	2
2	03/11/2020	H16.0	1	Úlcera corneal	2
1	03/11/2020	H16.0	1	úlcera corneal	2
1	03/11/2020	H16.0	1	Úlcera corneal	2
2	03/11/2020	H00.02	4	Orzuelo	2
1	03/11/2020	H20	6	Uveitis	2
2	03/11/2020	H11.3	0	Hiposfagma	2
1	03/11/2020	H16.0	1	Úlcera corneal	2
2	03/11/2020	H11.3	0	Hiposfagma	2
2	03/11/2020	H16.9	1	Queratitis	2
1	03/11/2020	T15.0	1	Cuerpo extraño	2
1	03/11/2020	H16.9	1	Queratitis	2
2	03/11/2020	H16.9	1	Queratitis	2
2	04/11/2020	G43.9	10	Migraña	2
1	04/11/2020	T15.0	1	Cuerpo extraño	2
2	04/11/2020	H00.02	4	Orzuelo	2
2	04/11/2020	H35.30	8	DMAE	2
1	04/11/2020	S05.9	9	Traumatismo	2
2	04/11/2020	H15.1	6	Epiescleritis	2
1	04/11/2020	H16.0	1	Úlcera corneal	2
1	04/11/2020	H43.81	7	DVP	2
2	04/11/2020	H01.00	4	Blefaritis	2
2	04/11/2020	G43.9	10	Migraña	2
2	04/11/2020	H20	6	Uveitis	2
1	04/11/2020	H35.3	8	Maculopatía	2
2	04/11/2020	H00.03	4	Celulitis preseptal	2
1	04/11/2020	S05.30	0	Laceración conjuntival	2
2	04/11/2020	H10.3	0	Conjuntivitis	2
2	04/11/2020	H43.81	7	DVP	2
2	04/11/2020	H10.81	0	Pingueculitis	2
2	04/11/2020	H01.00	4	Blefaritis	2
2	04/11/2020	G43.9	10	Migraña	2
2	04/11/2020	E11.319	8	Retinopatía diabética	2
2	04/11/2020	H11.3	0	Hiposfagma	2
1	04/11/2020	T15.0	1	Cuerpo extraño	2
1	05/11/2020	H10.3	0	Conjuntivitis	2
1	05/11/2020	T15.0	1	Cuerpo extraño	2
1	05/11/2020	H10.5	0	Blefaroconjuntivitis	2
1	05/11/2020	H16.0	1	Úlcera corneal	2
2	05/11/2020	H10.3	0	Conjuntivitis alérgica	2
2	05/11/2020	H43.81	7	DVP	2
1	05/11/2020	H35.30	8	DMAE	2
1	05/11/2020	T15.0	1	Cuerpo extraño	2
2	05/11/2020	H43.81	7	DVP	2
1	05/11/2020	T26	9	Causticación	2
2	05/11/2020	G43.9	10	Migraña	2
2	05/11/2020	H43.81	7	DVP	2
2	05/11/2020	H04.41	5	Dacriocistitis crónica	2
1	05/11/2020	T15.0	1	Cuerpo extraño	2
1	05/11/2020	T15.0	1	Cuerpo extraño	2
1	05/11/2020	T15.0	1	Cuerpo extraño	2
1	05/11/2020	H11.3	0	Hiposfagma	2
2	05/11/2020	H01.00	4	Blefaritis	2
1	05/11/2020	H16.9	1	Queratitis	2
2	06/11/2020	H10.3	0	Conjuntivitis	2
2	06/11/2020	H10.3	0	Conjuntivitis	2
1	06/11/2020	H16.9	1	Queratitis	2
2	06/11/2020	H11.3	0	Hiposfagma	2
2	06/11/2020	H35.3	8	Maculopatía	2
1	06/11/2020	H16.0	1	Úlcera corneal	2
1	06/11/2020	H16.9	1	Queratitis	2
2	06/11/2020	H0.41	1	Ojo seco	2

2	06/11/2020	H16.9	1	Queratitis	2
2	06/11/2020	H00.02	4	Orzuelo	2
1	06/11/2020	Z98.4	2	Post op catarata	2
2	06/11/2020	H10.3	0	Conjuntivitis	2
2	06/11/2020	H01.00	4	Blefaritis	2
2	06/11/2020	H10.3	0	Conjuntivitis	2
2	06/11/2020	H00.02	4	Orzuelo	2
2	06/11/2020	H16.9	1	Queratitis	2
2	06/11/2020	H10.3	0	Conjuntivitis	2
2	06/11/2020	H10.3	0	Conjuntivitis	2
1	06/11/2020	H16.0	1	Úlcera corneal	2
1	06/11/2020	H59.3	4	Post op párpados	2
2	06/11/2020	G43.9	10	Migraña	2
1	06/11/2020	H20	6	Uveitis	2
1	06/11/2020	Alta por fuga	11	Alta por fuga	2
2	06/11/2020	H10.3	0	Conjuntivitis	2
2	06/11/2020	H27.8	2	OCP	2
2	06/11/2020	H16.9	1	Queratitis	2
1	06/11/2020	T15.0	1	Cuerpo extraño	2
2	06/11/2020	H16.0	1	Úlcera corneal	2
2	06/11/2020	H11.3	0	Hiposfagma	2
2	06/11/2020	H43.81	7	DVP	2
1	06/11/2020	H53	11	No patología	2
1	06/11/2020	H11.3	0	Hiposfagma	2
1	06/11/2020	H16.9	1	Queratitis	2
2	06/11/2020	H16.9	1	Queratitis	2
2	06/11/2020	H11.00	0	Pterigium	2
2	06/11/2020	H16.9	1	Queratitis	2
1	07/11/2020	H16.9	1	Queratitis	2
1	07/11/2020	T15.0	1	Cuerpo extraño	2
2	07/11/2020	H16.9	1	Queratitis	2
1	07/11/2020	S05.9	9	Traumatismo	2
1	07/11/2020	H10.3	0	Conjuntivitis	2
2	07/11/2020	H15.1	6	Epiescleritis	2
2	07/11/2020	H01.00	4	Blefaritis	2
2	07/11/2020	H59.34	4	Postop blefaroplastia	2
2	07/11/2020	H01.00	4	Blefaritis	2
1	07/11/2020	H33.0	8	Desprendimiento de retina	2
2	07/11/2020	H00.02	4	Orzuelo	2
2	07/11/2020	H10.3	0	Conjuntivitis	2
1	07/11/2020	H10.3	0	Conjuntivitis	2
2	07/11/2020	H43.81	7	DVP	2
2	07/11/2020	H00.02	4	Orzuelo	2
1	07/11/2020	H16.0	1	Úlcera corneal	2
1	07/11/2020	H33.30	8	Desgarro retiniano	2
2	07/11/2020	H01.00	4	Blefaritis	2
2	07/11/2020	H53	11	No patología	2
1	07/11/2020	H15.1	6	Epiescleritis	2
2	07/11/2020	H11.3	0	Hiposfagma	2
1	07/11/2020	H16.0	1	Úlcera corneal	2
2	07/11/2020	H0.41	1	Ojo seco	2
1	07/11/2020	H16.0	1	Úlcera corneal	2
2	07/11/2020	H01.00	4	Blefaritis	2
2	07/11/2020	H10.3	0	Conjuntivitis	2
2	07/11/2020	H16.0	1	Úlcera corneal	2
2	07/11/2020	H16.9	1	Queratitis	2
2	07/11/2020	H00.02	4	Orzuelo	2
1	07/11/2020	H0.41	1	ojo seco	2
1	07/11/2020	G43.9	10	Migraña	2
2	07/11/2020	H10.3	0	Conjuntivitis	2
1	07/11/2020	H10.3	0	Conjuntivitis	2
1	08/11/2020	H17.8	1	Infiltrados corneales	2
1	08/11/2020	H10.3	0	Conjuntivitis	2
1	08/11/2020	H0.41	1	Ojo seco	2
2	08/11/2020	H11.3	0	Hiposfagma	2
1	08/11/2020	H16.0	1	Úlcera corneal	2
2	08/11/2020	H00.02	4	Orzuelo	2
1	08/11/2020	H00.02	4	Orzuelo	2
1	08/11/2020	T15.0	1	Cuerpo extraño	2
2	08/11/2020	H16.0	1	Úlcera corneal	2
1	08/11/2020	H10.3	0	Conjuntivitis	2

2	08/11/2020	H11.3	0	Hiposfagma	2
2	08/11/2020	H43.81	7	DVP	2
2	08/11/2020	H16.9	1	Queratitis	2
2	08/11/2020	H16.0	1	Úlcera corneal	2
2	08/11/2020	H11.44	0	Quiste conjuntival	2
1	08/11/2020	H20	6	Uveitis	2
1	08/11/2020	T15.0	1	Cuerpo extraño	2
1	08/11/2020	H27.8	2	OCP	2
2	08/11/2020	H11.3	0	Hiposfagma	2
1	08/11/2020	H16.0	1	Úlcera corneal	2
1	09/11/2020	S05.9	9	Traumatismo	2
1	09/11/2020	H16.9	1	Queratitis	2
1	09/11/2020	H10.3	0	Conjuntivitis	2
2	09/11/2020	H16.9	1	Queratitis	2
2	09/11/2020	H00.02	4	Orzuelo	2
2	09/11/2020	H00.02	4	Orzuelo	2
1	09/11/2020	H43.81	7	DVP	2
2	09/11/2020	H53	11	No patología	2
2	09/11/2020	H10.3	0	Conjuntivitis	2
1	09/11/2020	H10.3	0	Conjuntivitis	2
2	09/11/2020	H10.3	0	Conjuntivitis	2
2	09/11/2020	H10.3	0	Conjuntivitis	2
2	09/11/2020	H16.0	1	Úlcera corneal	2
1	09/11/2020	H35.3	8	Maculopatía	2
1	09/11/2020	H10.3	0	Conjuntivitis	2
2	09/11/2020	H43.81	7	DVP	2
1	09/11/2020	H16.0	1	Úlcera corneal	2
1	09/11/2020	T15.0	1	Cuerpo extraño	2
2	09/11/2020	H16.0	1	Úlcera corneal	2
1	09/11/2020	H01.00	4	Blefaritis	2
2	09/11/2020	H00.19	4	Chalazión	2
2	09/11/2020	H16.0	1	Úlcera corneal	2
1	09/11/2020	S05.9	9	Traumatismo	2
2	09/11/2020	H20	6	Uveitis	2
1	09/11/2020	T15.0	1	Cuerpo extraño	2
2	10/11/2020	H16.9	1	Queratitis	2
1	10/11/2020	H16.0	1	Úlcera corneal	2
2	10/11/2020	H27.8	2	OCP	2
2	10/11/2020	B00.1	4	Dermatitis herpética	2
2	10/11/2020	H00.19	4	Chalazión	2
2	10/11/2020	H33.0	8	Desprendimiento de retina	2
1	10/11/2020	H16.9	1	Queratitis	2
2	10/11/2020	H16.9	1	Queratitis	2
2	10/11/2020	H01.00	4	Blefaritis	2
1	10/11/2020	H16.0	1	Úlcera corneal	2
1	10/11/2020	H16.9	1	Queratitis	2
1	10/11/2020	H16.9	1	Queratitis	2
2	10/11/2020	H0.41	1	Ojo seco	2
2	10/11/2020	H10.3	0	Conjuntivitis	2
2	10/11/2020	H10.3	0	Conjuntivitis	2
2	10/11/2020	H15.1	6	Epiescleritis	2
2	10/11/2020	G43.9	10	Migraña	2
1	10/11/2020	T26	9	Causticación	2
1	11/11/2020	T15.0	1	Cuerpo extraño	2
1	11/11/2020	B00.1	4	Dermatitis herpética	2
1	11/11/2020	H10.3	0	Conjuntivitis	2
1	11/11/2020	H50.9	10	Estrabismo	2
2	11/11/2020	H16.0	1	Úlcera corneal	2
2	11/11/2020	H35.30	8	DMAE	2
2	11/11/2020	H11.00	0	Pterigium	2
2	11/11/2020	H33.30	8	Desgarro retiniano	2
2	11/11/2020	H16.9	1	Queratitis	2
1	11/11/2020	H20	6	Uveitis	2
1	11/11/2020	H43.81	7	DVP	2
2	11/11/2020	S05.6	9	Perforación ocular	2
2	11/11/2020	H16.0	1	Úlcera corneal	2
2	11/11/2020	H27.8	2	OCP	2
2	11/11/2020	Z98.4	2	Post op catarata	2
1	11/11/2020	H20	6	Uveitis	2
2	11/11/2020	S05.9	9	Traumatismo	2
2	11/11/2020	H10.3	0	Conjuntivitis	2

2	11/11/2020	H00.02	4	Orzuelo	2
2	11/11/2020	H00.02	4	Orzuelo	2
2	11/11/2020	H15.00	6	Escleritis	2
1	12/11/2020	S05.30	0	Laceración conjuntival	2
2	12/11/2020	H16.9	1	Queratitis	2
2	12/11/2020	H16.9	1	Queratitis	2
1	12/11/2020	H00.02	4	Orzuelo	2
2	12/11/2020	H43.81	7	DVP	2
2	12/11/2020	H16.9	1	Queratitis	2
2	12/11/2020	H18.20	1	Edema corneal	2
1	12/11/2020	H33.0	8	Desprendimiento de retina	2
1	12/11/2020	H01.00	4	Blefaritis	2
2	12/11/2020	G45.3	10	Amaurosis fugax	2
2	12/11/2020	H35.30	8	DMAE	2
1	12/11/2020	H16.0	1	Úlcera corneal	2
1	12/11/2020	H11.00	0	Pterigium	2
1	12/11/2020	S05.9	9	Traumatismo	2
1	12/11/2020	H01.00	4	Blefaritis	2
2	12/11/2020	H20	6	Uveitis	2
2	12/11/2020	H16.9	1	Queratitis	2
2	12/11/2020	H11.44	0	Quiste conjuntival	2
2	12/11/2020	H26.9	2	Catarata	2
1	12/11/2020	H40.9	3	Glaucoma	2
2	12/11/2020	H16.9	1	Queratitis	2
2	12/11/2020	H16.9	1	Queratitis	2
2	12/11/2020	H20	6	Uveitis	2
2	12/11/2020	H11.3	0	Hiposfagma	2
1	12/11/2020	H16.9	1	Queratitis	2
1	12/11/2020	H10.3	0	Conjuntivitis	2
2	12/11/2020	H10.3	0	Conjuntivitis	2
1	12/11/2020	H11.3	0	Hiposfagma	2
1	12/11/2020	H16.0	1	Úlcera corneal	2
2	12/11/2020	H00.03	4	Celulitis preseptal	2
1	12/11/2020	H16.0	1	Úlcera corneal	2
1	13/11/2020	H00.02	4	Orzuelo	2
1	13/11/2020	H10.5	0	Blefaroconjuntivitis	2
1	13/11/2020	S05.9	9	Traumatismo	2
2	13/11/2020	G43.9	10	Migraña	2
2	13/11/2020	H11.3	0	Hiposfagma	2
1	13/11/2020	H16.0	1	Úlcera corneal	2
1	13/11/2020	H16.0	1	Úlcera corneal	2
1	13/11/2020	Z97.0	4	Protesis ocular	2
2	13/11/2020	H16.9	1	Queratitis	2
1	13/11/2020	H16.9	1	Queratitis	2
1	13/11/2020	T15.0	1	Cuerpo extraño	2
1	13/11/2020	E11.319	8	Retinopatía diabética	2
1	13/11/2020	H16.0	1	Úlcera corneal	2
2	13/11/2020	H43.81	7	DVP	2
2	13/11/2020	H00.02	4	Orzuelo	2
2	13/11/2020	H16.9	1	Queratitis	2
2	13/11/2020	H20	6	Uveitis	2
1	13/11/2020	H27.10	2	Luxación LIO	2
2	13/11/2020	S05.9	9	Traumatismo	2
2	13/11/2020	H16.9	1	Queratitis	2
1	13/11/2020	H16.9	1	Queratitis	2
1	13/11/2020	H20	6	Uveitis	2
2	13/11/2020	H43.81	7	DVP	2
1	13/11/2020	H16.9	1	Queratitis	2
2	13/11/2020	Alta por fuga	11	Alta por fuga	2
2	13/11/2020	H16.0	1	Úlcera corneal	2
1	13/11/2020	H16.9	1	Queratitis	2
1	14/11/2020	H16.0	1	Úlcera corneal	2
1	14/11/2020	H16.9	1	Queratitis	2
1	14/11/2020	T15.0	1	Cuerpo extraño	2
1	14/11/2020	H10.3	0	Conjuntivitis	2
2	14/11/2020	H16.0	4	Úlcera corneal	2
2	14/11/2020	H43.81	7	DVP	2
1	14/11/2020	T15.0	1	Cuerpo extraño	2
1	14/11/2020	H00.02	4	Orzuelo	2
2	14/11/2020	H16.9	1	Queratitis	2
1	14/11/2020	H11.3	0	Hiposfagma	2

1	14/11/2020	T15.0		1	Cuerpo extraño	2
2	14/11/2020	H16.9		1	Queratitis	2
1	14/11/2020	T15.0		1	Cuerpo extraño	2
1	14/11/2020	H16.9		1	Queratitis	2
1	14/11/2020	T15.0		1	Cuerpo extraño	2
1	14/11/2020	T15.0		1	Cuerpo extraño	2
2	14/11/2020	T15.0		1	Cuerpo extraño	2
2	14/11/2020	H16.0		1	Úlcera corneal	2
1	14/11/2020	S05.9		9	Traumatismo	2
2	15/11/2020	H10.5		0	Blefaroconjuntivitis	2
2	15/11/2020	H16.9		1	Queratitis	2
2	15/11/2020	H11.3		0	Hiposfagma	2
1	15/11/2020	H16.0		1	Úlcera corneal	2
2	15/11/2020	H16.0		1	Úlcera corneal	2
1	15/11/2020	H00.02		4	Orzuelo	2
1	15/11/2020	H16.0		1	Úlcera corneal	2
1	15/11/2020	H16.9		1	Queratitis	2
1	15/11/2020	T26		9	Causticación	2
1	15/11/2020	H00.02		4	Orzuelo	2
2	15/11/2020	H17.8		1	Infiltrados corneales	2
2	15/11/2020	H00.03		4	Celulitis preseptal	2
2	15/11/2020	H00.02		4	Orzuelo	2
2	15/11/2020	H16.0		1	Úlcera corneal	2
2	15/11/2020	H16.0		1	Úlcera corneal	2
1	15/11/2020	H04.41		5	Dacriocistitis crónica	2
2	15/11/2020	H00.02		4	Orzuelo	2
2	16/11/2020	H16.0		1	Úlcera corneal	2
1	16/11/2020	H02		4	Alteración palpebral	2
1	16/11/2020	T15.0		1	Cuerpo extraño	2
2	16/11/2020	H0.41		1	Ojo seco	2
2	16/11/2020	H43.81		7	DVP	2
2	16/11/2020	H43.81		7	DVP	2
2	16/11/2020	H01.00		4	Blefaritis	2
2	16/11/2020	H10.3		0	Conjuntivitis	2
2	16/11/2020	H43.81		7	DVP	2
2	16/11/2020	H10.3		0	Conjuntivitis	2
1	16/11/2020	H11.3		0	Hiposfagma	2
1	16/11/2020	H40.05		3	HTO	2
2	16/11/2020	Z98.4		1	Post op catarata	2
2	16/11/2020	E11.319		8	Retinopatía diabética	2
2	16/11/2020	H53		11	No patología	2
2	16/11/2020	H10.3		0	Conjuntivitis	2
1	16/11/2020	S05.9		9	Traumatismo	2
2	16/11/2020	H16.0		4	Úlcera corneal	2
2	16/11/2020	H11.44		0	Quiste conjuntival	2
1	16/11/2020	H16.9		1	Queratitis	2
1	16/11/2020	T15.0		1	Cuerpo extraño	2
2	16/11/2020	H01.00		4	Blefaritis	2
2	16/11/2020	S05.9		9	Traumatismo	2
2	16/11/2020	H10.3		0	Conjuntivitis	2
1	16/11/2020	T15.0		1	Cuerpo extraño	2
2	17/11/2020	S05.9		9	Traumatismo	2
2	17/11/2020	H10.3		0	Conjuntivitis	2
1	17/11/2020	T15.0		1	Cuerpo extraño	2
2	17/11/2020	H20		6	Uveitis	2
2	17/11/2020	H43.81		7	DVP	2
1	17/11/2020	H00.02		4	Orzuelo	2
1	17/11/2020	T15.0		1	Cuerpo extraño	2
1	17/11/2020	H10.3		0	Conjuntivitis	2
2	17/11/2020	H16.0		1	Úlcera corneal	2
2	17/11/2020	H43.81		7	DVP	2
2	17/11/2020	H16.9		1	Queratitis	2
1	17/11/2020	H16.0		1	Úlcera corneal	2
1	17/11/2020	H46		10	Neuritis óptica	2
2	17/11/2020	T26		9	Causticación	2
2	17/11/2020	H10.3		0	Conjuntivitis	2
2	17/11/2020	H26.9		2	Catarata	2
1	17/11/2020	H10.3		0	Conjuntivitis	2
1	17/11/2020	H11.44		0	Quiste conjuntival	2
2	17/11/2020	H43.81		7	DVP	2
1	17/11/2020	S05.9		9	Traumatismo	2

1	17/11/2020	H16.0	1	Úlcera corneal	2
2	17/11/2020	H10.3	0	Conjuntivitis	2
1	17/11/2020	S05.9	9	Traumatismo	2
1	17/11/2020	S05.9	9	Traumatismo	2
2	17/11/2020	H16.0	1	Úlcera corneal	2
2	17/11/2020	H16.0	1	Úlcera corneal	2
1	18/11/2020	H00.02	4	Orzuelo	2
1	18/11/2020	H11.3	0	Hiposfagma	2
2	18/11/2020	H10.3	0	Conjuntivitis	2
2	18/11/2020	H16.9	1	Queratitis	2
1	18/11/2020	H10.3	0	Conjuntivitis	2
2	18/11/2020	H26.9	2	Catarata	2
1	18/11/2020	H0.41	1	ojo seco	2
2	18/11/2020	H26.9	2	Catarata	2
2	18/11/2020	H01.00	4	Blefaritis	2
1	18/11/2020	H01.00	4	Blefaritis	2
2	18/11/2020	H18.20	1	Edema corneal	2
1	18/11/2020	H10.3	0	Conjuntivitis	2
1	18/11/2020	S05.6	9	Perforación ocular	2
1	18/11/2020	H11.00	0	Pterigium	2
2	18/11/2020	H43.81	7	DVP	2
2	18/11/2020	H16.9	1	Queratitis	2
1	18/11/2020	S05.30	0	Laceración conjuntival	2
1	18/11/2020	H43.81	7	DVP	2
1	18/11/2020	H16.0	1	Úlcera corneal	2
2	18/11/2020	H00.02	4	Orzuelo	2
1	18/11/2020	H10.3	0	Conjuntivitis	2
2	18/11/2020	H02	4	Lesión palpebral	2
1	18/11/2020	S05.9	9	Traumatismo	2
1	18/11/2020	H0.41	1	Ojo seco	2
1	18/11/2020	T15.0	1	Cuerpo extraño	2
2	18/11/2020	S05.9	9	Traumatismo	2
1	18/11/2020	H16.9	1	Queratitis	2
1	18/11/2020	T15.0	1	Cuerpo extraño	2
2	19/11/2020	G43.9	10	Migraña	2
2	19/11/2020	H02	4	Lesión palpebral	2
1	19/11/2020	H16.9	1	Queratitis	2
2	19/11/2020	H16.9	1	Queratitis	2
2	19/11/2020	H43.81	7	DVP	2
2	19/11/2020	H16.9	1	Queratitis	2
1	19/11/2020	H16.0	1	Úlcera corneal	2
2	19/11/2020	H35.30	8	DMAE	2
2	19/11/2020	H10.3	0	Conjuntivitis	2
2	19/11/2020	H10.3	0	Conjuntivitis	2
2	19/11/2020	H20	6	Uveitis	2
2	19/11/2020	H10.3	0	Conjuntivitis	2
2	19/11/2020	H15.00	6	Escleritis	2
2	19/11/2020	H02	4	Lesión palpebral	2
1	19/11/2020	H00.02	4	Orzuelo	2
1	19/11/2020	H00.02	4	Orzuelo	2
2	19/11/2020	H11.3	0	Hiposfagma	2
2	19/11/2020	H10.3	0	Conjuntivitis	2
2	19/11/2020	H16.0	1	Úlcera corneal	2
2	19/11/2020	G43.9	10	Migraña	2
2	19/11/2020	H16.9	1	Queratitis	2
1	19/11/2020	H53	11	No patología	2
1	19/11/2020	H27.8	2	OCP	2
2	19/11/2020	H33.30	8	Desgarro retiniano	2
1	19/11/2020	H16.9	1	Queratitis	2
1	19/11/2020	H16.0	1	Úlcera corneal	2
1	19/11/2020	T15.0	1	Cuerpo extraño	2
2	19/11/2020	H43.81	7	DVP	2
1	19/11/2020	H16.9	1	Queratitis	2
1	19/11/2020	H16.0	1	Úlcera corneal	2
1	20/11/2020	H16.0	1	Úlcera corneal	2
1	20/11/2020	H27.8	2	OCP	2
1	20/11/2020	H01.00	4	Blefaritis	2
1	20/11/2020	H02	4	Alteración palpebral	2
1	20/11/2020	H43.81	7	DVP	2
2	20/11/2020	H04.41	5	Dacriocistitis crónica	2
1	20/11/2020	H10.3	0	Conjuntivitis	2

1	20/11/2020	H43.81	7	DVP	2
1	20/11/2020	H10.3	0	Conjuntivitis	2
1	20/11/2020	H18.20	1	Edema corneal	2
1	20/11/2020	H10.3	0	Conjuntivitis	2
1	20/11/2020	H02	4	Lesión palpebral	2
1	20/11/2020	H16.9	1	Queratitis	2
2	20/11/2020	H16.9	1	Queratitis	2
1	20/11/2020	H01.00	4	Blefaritis	2
1	20/11/2020	H33.0	8	Desprendimiento de retina	2
2	20/11/2020	G43.9	10	Migraña	2
1	20/11/2020	S05.9	9	Traumatismo	2
1	20/11/2020	T15.0	1	Cuerpo extraño	2
2	20/11/2020	H01.00	4	Blefaritis	2
2	20/11/2020	H16.9	1	Queratitis	2
2	20/11/2020	T15.0	1	Cuerpo extraño	2
1	21/11/2020	S05.9	9	Traumatismo	2
1	21/11/2020	H0.41	1	Ojo seco	2
1	21/11/2020	H01.00	4	Blefaritis	2
1	21/11/2020	H53.10	11	Problema refractivo	2
1	21/11/2020	H20	6	Uveitis	2
2	21/11/2020	H16.9	1	Queratitis	2
1	21/11/2020	T15.0	1	Cuerpo extraño	2
2	21/11/2020	H16.0	1	Úlcera corneal	2
2	21/11/2020	H16.0	1	Úlcera corneal	2
1	21/11/2020	H10.3	0	Conjuntivitis	2
1	21/11/2020	H16.0	1	Úlcera corneal	2
1	21/11/2020	H10.3	0	Conjuntivitis	2
2	21/11/2020	H11.3	0	Hiposfagma	2
2	21/11/2020	H10.3	0	Conjuntivitis	2
2	21/11/2020	H00.19	4	Chalazión	2
1	21/11/2020	H10.3	0	Conjuntivitis	2
2	21/11/2020	H16.9	1	Queratitis	2
2	21/11/2020	H11.3	0	Hiposfagma	2
1	21/11/2020	B00.1	4	Dermatitis herpética	2
1	21/11/2020	T15.0	1	Cuerpo extraño	2
2	21/11/2020	H10.3	0	Conjuntivitis	2
1	21/11/2020	H16.0	1	Úlcera corneal	2
1	21/11/2020	H11.3	0	Hiposfagma	2
1	21/11/2020	H43.81	7	DVP	2
2	21/11/2020	H15.00	6	Escleritis	2
1	21/11/2020	T15.0	1	Cuerpo extraño	2
2	21/11/2020	G43.9	10	Migraña	2
2	21/11/2020	H16.9	1	Queratitis	2
1	21/11/2020	H17.8	1	Infiltrados corneales	2
1	21/11/2020	H10.3	0	Conjuntivitis	2
2	21/11/2020	H02	4	Lesión palpebral	2
1	21/11/2020	H20	6	Uveitis	2
2	21/11/2020	H59.3	5	Postop via lagrimal	2
1	21/11/2020	H43.1	7	Hemovítreo	2
2	22/11/2020	H16.0	1	Úlcera corneal	2
1	22/11/2020	S05.9	9	Traumatismo	2
1	22/11/2020	H01.00	4	Blefaritis	2
2	22/11/2020	H01.00	4	Blefaritis	2
1	22/11/2020	T15.0	1	Cuerpo extraño	2
1	22/11/2020	T15.0	1	Cuerpo extraño	2
2	22/11/2020	H10.3	0	Conjuntivitis	2
2	22/11/2020	H05.01	4	Celulitis orbitaria	2
2	22/11/2020	H01.00	4	Blefaritis	2
1	22/11/2020	T15.0	1	Cuerpo extraño	2
2	22/11/2020	H10.3	0	Conjuntivitis	2
1	22/11/2020	H10.3	0	Conjuntivitis	2
2	22/11/2020	H00.02	4	Orzuelo	2
2	22/11/2020	H11.44	1	Quiste conjuntival	2
1	22/11/2020	H20	6	Uveitis	2
1	22/11/2020	H18.20	1	Edema corneal	2
1	22/11/2020	H10.3	0	Conjuntivitis	2
2	22/11/2020	H43.81	7	DVP	2
1	22/11/2020	T15.0	1	Cuerpo extraño	2
2	22/11/2020	H10.3	0	Conjuntivitis	2
2	22/11/2020	H16.0	1	Úlcera corneal	2
2	22/11/2020	H16.0	1	Úlcera corneal	2

2	22/11/2020	T26	9	Causticación	2
1	22/11/2020	H10.3	0	Conjuntivitis	2
2	22/11/2020	H10.3	0	Conjuntivitis	2
2	22/11/2020	H11.3	0	Hiposfagma	2
1	22/11/2020	H33.0	8	Desprendimiento de retina	2
2	22/11/2020	H10.3	0	Conjuntivitis	2
2	22/11/2020	H11.3	0	Hiposfagma	2
2	22/11/2020	H16.9	1	Queratitis	2
2	22/11/2020	H16.0	1	Úlcera corneal	2
2	22/11/2020	H10.3	0	Conjuntivitis	2
2	23/11/2020	H33.30	8	Desgarro retiniano	2
2	23/11/2020	H10.3	0	Conjuntivitis	2
1	23/11/2020	H00.02	4	Orzuelo	2
1	23/11/2020	T15.0	1	Cuerpo extraño	2
1	23/11/2020	H01.00	4	Blefaritis	2
1	23/11/2020	H16.0	1	Úlcera corneal	2
2	23/11/2020	H01.00	4	Blefaritis	2
1	23/11/2020	H44.009	8	Endoftalmitis	2
1	23/11/2020	H27.8	2	OCP	2
2	23/11/2020	H43.81	7	DVP	2
2	23/11/2020	H02.05	4	Distiquiasis	2
1	23/11/2020	H15.1	6	Epiescleritis	2
1	23/11/2020	H20	6	Uveitis	2
2	23/11/2020	H00.02	4	Orzuelo	2
1	23/11/2020	H16.9	1	Queratitis	2
1	23/11/2020	H49.9	10	Parálisis oculomotora	2
1	23/11/2020	H17.8	1	Infiltrados corneales	2
2	23/11/2020	H02	4	Lesión palpebral	2
1	23/11/2020	H16.9	1	Queratitis	2
2	23/11/2020	H10.81	0	Pingueculitis	2
1	23/11/2020	H01.00	4	Blefaritis	2
2	23/11/2020	H16.9	1	Queratitis	2
2	23/11/2020	T15.0	1	Cuerpo extraño	2
1	23/11/2020	T15.0	1	Cuerpo extraño	2
2	23/11/2020	G43.109	10	Aura	2
2	23/11/2020	H43.81	7	DVP	2
1	23/11/2020	H16.0	1	Úlcera corneal	2
1	23/11/2020	H00.02	4	Orzuelo	2
2	23/11/2020	H01.00	4	Blefaritis	2
2	23/11/2020	H0.41	1	ojo seco	2
1	23/11/2020	H10.3	0	Conjuntivitis	2
2	23/11/2020	H27.8	2	OCP	2
1	23/11/2020	H11.00	0	Pterigium	2
1	23/11/2020	H16.0	1	Úlcera corneal	2
2	23/11/2020	H10.5	0	Blefaroconjuntivitis	2
1	23/11/2020	T15.0	1	Cuerpo extraño	2
1	23/11/2020	H43.81	7	DVP	2
1	23/11/2020	H16.0	1	Úlcera corneal	2
2	23/11/2020	H0.41	1	Ojo seco	2
2	23/11/2020	H16.0	1	Úlcera corneal	2
2	23/11/2020	H16.0	1	Úlcera corneal	2
2	23/11/2020	H10.3	0	Conjuntivitis	2
2	23/11/2020	H16.9	1	Queratitis	2
2	24/11/2020	H43.81	7	DVP	2
2	24/11/2020	H11.3	0	Hiposfagma	2
2	24/11/2020	H20	6	Uveitis	2
1	24/11/2020	H26.9	2	Catarata	2
1	24/11/2020	H01.00	4	Blefaritis	2
2	24/11/2020	H00.02	4	Orzuelo	2
1	24/11/2020	H20	6	Uveitis	2
1	24/11/2020	H01.00	4	Blefaritis	2
2	24/11/2020	H01.00	4	Blefaritis	2
1	24/11/2020	H40.9	3	Glaucoma	2
2	24/11/2020	H01.00	4	Blefaritis	2
2	24/11/2020	H16.0	1	Úlcera corneal	2
2	24/11/2020	H01.00	4	Blefaritis	2
1	24/11/2020	S05.9	9	Traumatismo	2
1	24/11/2020	H59.3	5	Postop via lagrimal	2
1	24/11/2020	H35.30	8	DMAE	2
2	24/11/2020	H16.0	1	Úlcera corneal	2
2	24/11/2020	H10.3	0	Conjuntivitis	2

2	24/11/2020	H43.1	7	Hemovítreo	2
1	24/11/2020	H00.19	4	Chalazión	2
1	24/11/2020	H20	6	Uveitis	2
2	24/11/2020	G43.9	10	Migraña	2
1	24/11/2020	T15.0	1	Cuerpo extraño	2
1	24/11/2020	H01.00	4	Blefaritis	2
1	24/11/2020	H11.3	0	Hiposfagma	2
1	24/11/2020	S05.9	9	Traumatismo	2
1	24/11/2020	S05.9	9	Traumatismo	2
1	24/11/2020	H16.0	1	Úlcera corneal	2
1	24/11/2020	H10.3	0	Conjuntivitis	2
1	24/11/2020	T15.0	1	Cuerpo extraño	2
1	24/11/2020	H10.3	0	Conjuntivitis	2
1	24/11/2020	H16.0	1	Úlcera corneal	2
1	25/11/2020	H16.9	1	Queratitis	2
2	25/11/2020	H10.3	0	Conjuntivitis	2
2	25/11/2020	H16.0	1	Úlcera corneal	2
2	25/11/2020	H01.00	4	Blefaritis	2
1	25/11/2020	H16.0	1	Úlcera corneal	2
2	25/11/2020	H10.3	0	Conjuntivitis	2
1	25/11/2020	H50.9	10	Estrabismo	2
2	25/11/2020	H11.82	0	Conjuntivocalasia	2
1	25/11/2020	H15.1	6	Epiescleritis	2
2	25/11/2020	H10.3	0	Conjuntivitis	2
1	25/11/2020	H44.009	8	Endoftalmitis	2
2	25/11/2020	H10.3	0	Conjuntivitis	2
2	25/11/2020	H00.02	4	Orzuelo	2
1	25/11/2020	H26.9	2	Catarata	2
1	25/11/2020	H15.1	6	Epiescleritis	2
2	25/11/2020	H16.0	1	Úlcera corneal	2
2	25/11/2020	H16.9	1	Queratitis	2
2	25/11/2020	H16.9	1	Queratitis	2
1	25/11/2020	H10.3	0	Conjuntivitis	2
1	25/11/2020	T15.0	1	Cuerpo extraño	2
1	25/11/2020	H11.3	0	Hiposfagma	2
1	25/11/2020	H16.9	1	Queratitis	2
2	25/11/2020	G43.9	10	Migraña	2
1	26/11/2020	T15.0	1	Cuerpo extraño	2
1	26/11/2020	T15.0	1	Cuerpo extraño	2
2	26/11/2020	H16.0	1	Úlcera corneal	2
2	26/11/2020	H10.3	0	Conjuntivitis	2
1	26/11/2020	H26.9	2	Catarata	2
1	26/11/2020	H43.1	7	Hemovítreo	2
1	26/11/2020	H10.3	0	Conjuntivitis	2
2	26/11/2020	H00.02	4	Orzuelo	2
1	26/11/2020	H16.0	1	Úlcera corneal	2
2	26/11/2020	H59.3	5	Post op párpados	2
2	26/11/2020	H49.9	10	Parálisis oculomotora	2
2	26/11/2020	H11.3	0	Hiposfagma	2
1	26/11/2020	B00.1	4	Dermatitis herpética	2
1	26/11/2020	H16.0	1	Úlcera corneal	2
2	26/11/2020	H40.05	3	HTO	2
1	26/11/2020	H20	6	Uveitis	2
1	26/11/2020	H01.00	4	Blefaritis	2
1	26/11/2020	H16.9	1	Queratitis	2
1	26/11/2020	H16.9	1	Queratitis	2
2	26/11/2020	H16.9	1	Queratitis	2
1	26/11/2020	B00.1	4	Dermatitis herpética	2
2	26/11/2020	H10.81	0	Pingueculitis	2
1	26/11/2020	Alta por fuga	11	Alta por fuga	2
1	26/11/2020	H16.9	1	Queratitis	2
1	26/11/2020	H16.9	1	Queratitis	2
1	26/11/2020	H27.8	2	OCP	2
1	26/11/2020	H43.81	7	DVP	2
2	26/11/2020	H43.81	7	DVP	2
2	26/11/2020	H10.3	0	Conjuntivitis	2
2	26/11/2020	H16.9	1	Queratitis	2
2	26/11/2020	H0.41	1	Ojo seco	2
2	26/11/2020	H16.0	1	Úlcera corneal	2
2	26/11/2020	T15.0	1	Cuerpo extraño	2
1	26/11/2020	T15.0	1	Cuerpo extraño	2

2	27/11/2020	H00.02	4	Orzuelo	2
2	27/11/2020	H16.9	1	Queratitis	2
1	27/11/2020	T15.0	1	Cuerpo extraño	2
2	27/11/2020	H16.9	1	Queratitis	2
1	27/11/2020	H16.0	1	Úlcera corneal	2
1	27/11/2020	S05.9	9	Traumatismo	2
2	27/11/2020	H16.0	1	Úlcera corneal	2
2	27/11/2020	T26.4	9	Quemadura	2
2	27/11/2020	H01.00	4	Blefaritis	2
2	27/11/2020	H35.30	8	DMAE	2
2	27/11/2020	H20	6	Uveitis	2
2	27/11/2020	H04.41	5	Dacriocistitis crónica	2
1	27/11/2020	H43.81	7	DVP	2
2	27/11/2020	H10.3	0	Conjuntivitis	2
2	27/11/2020	H43.81	7	DVP	2
2	27/11/2020	H10.3	0	Conjuntivitis	2
1	27/11/2020	T26	9	Causticación	2
2	27/11/2020	H00.03	4	Celulitis preseptal	2
2	27/11/2020	H47.10	10	Papiledema	2
2	27/11/2020	H16.9	1	Queratitis	2
2	27/11/2020	H11.3	0	Hiposfagma	2
1	27/11/2020	S05.9	9	Traumatismo	2
2	27/11/2020	H26.9	2	Catarata	2
1	27/11/2020	H10.3	0	Conjuntivitis	2
1	27/11/2020	H01.00	4	Blefaritis	2
2	27/11/2020	H00.03	4	Celulitis preseptal	2
2	27/11/2020	H20	6	Uveitis	2
2	27/11/2020	H02	4	Lesión palpebral	2
1	27/11/2020	H00.02	4	Orzuelo	2
2	27/11/2020	H01.00	4	Blefaritis	2
2	27/11/2020	H20	6	Uveitis	2
2	27/11/2020	H43.81	7	DVP	2
1	27/11/2020	H10.3	0	Conjuntivitis	2
2	27/11/2020	H16.9	1	Queratitis	2
1	28/11/2020	H10.3	0	Conjuntivitis	2
2	28/11/2020	H27.8	2	OCP	2
1	28/11/2020	H11.3	0	Hiposfagma	2
2	28/11/2020	H11.3	0	Hiposfagma	2
1	28/11/2020	H18.20	1	Edema corneal	2
2	28/11/2020	T15.0	1	Cuerpo extraño	2
1	28/11/2020	H11.3	0	Hiposfagma	2
1	28/11/2020	H20	6	Uveitis	2
1	28/11/2020	H10.3	0	Conjuntivitis	2
1	28/11/2020	H11.3	0	Hiposfagma	2
2	28/11/2020	H02	4	Lesión palpebral	2
1	28/11/2020	H20	6	Uveitis	2
1	28/11/2020	H00.02	4	Orzuelo	2
2	28/11/2020	H46	10	Neuritis óptica	2
2	28/11/2020	S05.6	9	Perforación ocular	2
2	28/11/2020	H27.8	2	OCP	2
2	28/11/2020	H16.0	3	Atalamia	2
2	28/11/2020	G43.9	10	Migraña	2
2	28/11/2020	H16.9	1	Queratitis	2
2	28/11/2020	H10.3	0	Conjuntivitis	2
2	28/11/2020	H33.0	8	Desprendimiento de retina	2
1	28/11/2020	H43.81	7	DVP	2
1	28/11/2020	H16.0	1	Úlcera corneal	2
1	28/11/2020	G43.9	10	Migraña	2
1	28/11/2020	H00.03	4	Celulitis preseptal	2
1	28/11/2020	H16.9	1	Queratitis	2
2	28/11/2020	H02	4	Lesión palpebral	2
1	28/11/2020	H40.9	3	Glaucoma	2
2	28/11/2020	H10.3	0	Conjuntivitis	2
1	28/11/2020	H16.0	1	Úlcera corneal	2
2	28/11/2020	H16.9	1	Queratitis	2
1	28/11/2020	T26	9	Causticación	2
1	28/11/2020	T15.0	1	Cuerpo extraño	2
1	28/11/2020	H00.02	4	Orzuelo	2
2	29/11/2020	H16.9	1	Queratitis	2
2	29/11/2020	H16.9	1	Queratitis	2
1	29/11/2020	S05.9	9	Traumatismo	2

2	29/11/2020	T15.0	1	Cuerpo extraño	2
1	29/11/2020	H16.0	1	Úlcera corneal	2
2	29/11/2020	H10.3	0	Conjuntivitis	2
2	29/11/2020	H11.82	0	Conjuntivocalasia	2
2	29/11/2020	H16.0	1	Úlcera corneal	2
1	29/11/2020	H16.0	1	Úlcera corneal	2
2	29/11/2020	H00.02	4	Orzuelo	2
1	29/11/2020	H20	6	Uveitis	2
1	29/11/2020	H01.00	4	Blefaritis	2
1	29/11/2020	H16.9	1	Queratitis	2
2	29/11/2020	H01.00	4	Blefaritis	2
1	29/11/2020	H10.3	0	Conjuntivitis	2
1	29/11/2020	T15.0	1	Cuerpo extraño	2
1	29/11/2020	H10.3	0	Conjuntivitis	2
1	29/11/2020	H43.81	7	DVP	2
2	29/11/2020	H00.02	4	Orzuelo	2
1	29/11/2020	H00.02	4	Orzuelo	2
1	29/11/2020	H16.9	1	Queratitis	2
1	29/11/2020	H16.0	1	Úlcera corneal	2
1	29/11/2020	H16.9	1	Queratitis	2
2	29/11/2020	T26	9	Causticación	2
1	29/11/2020	H11.3	0	Hiposfagma	2
1	29/11/2020	T15.0	1	Cuerpo extraño	2
1	29/11/2020	H16.0	1	Úlcera corneal	2
2	29/11/2020	H33.30	8	Desgarro retiniano	2
2	29/11/2020	H43.81	7	DVP	2
1	29/11/2020	H01.00	4	Blefaritis	2
2	29/11/2020	H16.9	1	Queratitis	2
2	29/11/2020	H16.9	1	Queratitis	2
1	29/11/2020	H16.0	1	Úlcera corneal	2
1	29/11/2020	H33.30	8	Desgarro retiniano	2
2	29/11/2020	Alta por fuga	11	Alta por fuga	2
2	30/11/2020	S05.6	9	Perforación ocular	2
2	30/11/2020	H10.3	0	Conjuntivitis	2
2	30/11/2020	H04.41	5	Dacriocistitis crónica	2
1	30/11/2020	H10.5	0	Blefaroconjuntivitis	2
2	30/11/2020	H16.0	1	Úlcera corneal	2
2	30/11/2020	H43.81	7	DVP	2
2	30/11/2020	H16.9	1	Queratitis	2
1	30/11/2020	H16.9	1	Queratitis	2
2	30/11/2020	H0.41	1	Ojo seco	2
2	30/11/2020	H16.9	1	Queratitis	2
2	30/11/2020	H10.5	0	Blefaroconjuntivitis	2
2	30/11/2020	H0.41	1	Ojo seco	2
2	30/11/2020	H00.02	4	Orzuelo	2
2	30/11/2020	H10.81	0	Pingueculitis	2
2	30/11/2020	H16.9	1	Queratitis	2
2	30/11/2020	H16.9	1	Queratitis	2
2	30/11/2020	H46	10	Neuritis óptica	2
2	30/11/2020	H43.81	7	DVP	2
2	30/11/2020	H00.03	4	Celulitis preseptal	2
1	30/11/2020	H0.41	1	Ojo seco	2
1	30/11/2020	B00.1	4	Dermatitis herpética	2
2	30/11/2020	H20	6	Uveitis	2
2	30/11/2020	H16.9	1	Queratitis	2
2	30/11/2020	H16.0	1	Úlcera corneal	2
2	30/11/2020	G43.9	10	Migraña	2
1	30/11/2020	H31.42	8	Coroidopatía serosa central	2
2	30/11/2020	H10.3	0	Conjuntivitis	2
2	30/11/2020	H10.3	0	Conjuntivitis	2
1	01/12/2020	H16.9	1	Queratitis	2
1	01/12/2020	S05.9	9	Traumatismo	2
1	01/12/2020	H10.3	0	Conjuntivitis	2
2	01/12/2020	H10.3	0	Conjuntivitis	2
2	01/12/2020	H11.3	0	Hiposfagma	2
2	01/12/2020	H16.9	1	Queratitis	2
2	01/12/2020	H10.3	0	Conjuntivitis	2
2	01/12/2020	H10.3	0	Conjuntivitis	2
2	01/12/2020	H11.3	0	Hiposfagma	2
1	01/12/2020	G43.9	10	Migraña	2
1	01/12/2020	S05.6	9	Perforación ocular	2

2	01/12/2020	H16.9	1	Queratitis	2
2	01/12/2020	E11.319	8	Retinopatía diabética	2
2	01/12/2020	H10.3	0	Conjuntivitis	2
1	01/12/2020	H16.9	1	Queratitis	2
1	01/12/2020	H16.9	1	Queratitis	2
2	01/12/2020	H43.81	7	DVP	2
1	01/12/2020	E11.319	8	Retinopatía diabética	2
1	01/12/2020	H16.9	1	Queratitis	2
2	01/12/2020	H53.10	11	Problema refractivo	2
2	01/12/2020	H43.81	7	DVP	2
2	01/12/2020	H43.81	7	DVP	2
1	01/12/2020	H10.3	0	Conjuntivitis	2
1	01/12/2020	H43.81	7	DVP	2
1	01/12/2020	T15.0	1	Cuerpo extraño	2
1	01/12/2020	S05.9	9	Traumatismo	2
2	02/12/2020	H00.02	4	Orzuelo	2
2	02/12/2020	H02.05	4	Distiquiasis	2
1	02/12/2020	H11.00	0	Pterigium	2
2	02/12/2020	H01.00	4	Blefaritis	2
2	02/12/2020	H16.9	1	Queratitis	2
2	02/12/2020	H16.9	1	Queratitis	2
1	02/12/2020	H16.0	1	Úlcera corneal	2
2	02/12/2020	H0.41	1	Ojo seco	2
2	02/12/2020	H00.02	4	Orzuelo	2
2	02/12/2020	H16.9	1	Queratitis	2
1	02/12/2020	H16.0	1	Úlcera corneal	2
2	02/12/2020	H10.3	0	Conjuntivitis	2
2	02/12/2020	H43.81	7	DVP	2
1	02/12/2020	S05.9	9	Traumatismo	2
2	02/12/2020	H16.9	1	Queratitis	2
1	02/12/2020	H11.3	0	Hiposfagma	2
2	02/12/2020	H10.3	0	Conjuntivitis	2
2	02/12/2020	H43.81	7	DVP	2
2	02/12/2020	H10.3	0	Conjuntivitis	2
2	02/12/2020	H16.0	1	Úlcera corneal	2
1	02/12/2020	S05.9	9	Traumatismo	2
1	02/12/2020	H16.9	1	Queratitis	2
1	02/12/2020	H01.00	4	Blefaritis	2
2	02/12/2020	H16.0	1	Úlcera corneal	2
1	02/12/2020	H15.1	6	Epiescleritis	2
2	02/12/2020	G43.9	10	Migraña	2
2	02/12/2020	H43.1	7	Hemovítreo	2
1	02/12/2020	S05.30	9	Laceración conjuntival	2
2	02/12/2020	H16.0	1	Úlcera corneal	2
2	03/12/2020	H16.0	1	Úlcera corneal	2
1	03/12/2020	Z98.4	2	Post op catarata	2
2	03/12/2020	H01.00	4	Blefaritis	2
2	03/12/2020	H43.81	7	DVP	2
2	03/12/2020	H16.9	1	Queratitis	2
2	03/12/2020	H02.05	4	Distiquiasis	2
1	03/12/2020	H35.3	8	Maculopatía	2
2	03/12/2020	H16.9	1	Queratitis	2
2	03/12/2020	H10.3	0	Conjuntivitis	2
1	03/12/2020	H16.0	1	Úlcera corneal	2
2	03/12/2020	H00.02	4	Orzuelo	2
1	03/12/2020	H10.3	0	Conjuntivitis	2
2	03/12/2020	H00.02	4	Orzuelo	2
2	03/12/2020	H00.02	4	Orzuelo	2
2	03/12/2020	T15.0	1	Cuerpo extraño	2
1	03/12/2020	H16.9	1	Queratitis	2
2	03/12/2020	H01.00	4	Blefaritis	2
1	03/12/2020	H11.3	0	Hiposfagma	2
1	03/12/2020	H43.81	7	DVP	2
2	03/12/2020	H43.81	7	DVP	2
1	03/12/2020	T15.0	1	Cuerpo extraño	2
1	03/12/2020	T15.0	1	Cuerpo extraño	2
2	03/12/2020	H16.0	1	Úlcera corneal	2
2	03/12/2020	H16.0	1	Úlcera corneal	2
2	03/12/2020	H11.3	0	Hiposfagma	2
1	03/12/2020	T15.0	1	Cuerpo extraño	2
2	03/12/2020	H16.9	1	Queratitis	2

1	03/12/2020	H16.0	1	Úlcera corneal	2
1	03/12/2020	H16.0	1	Úlcera corneal	2
2	03/12/2020	H16.9	1	Queratitis	2
1	03/12/2020	H16.0	1	Úlcera corneal	2
1	03/12/2020	H40.05	3	HTO	2
1	03/12/2020	H16.0	1	Úlcera corneal	2
2	04/12/2020	H27.8	2	OCP	2
2	04/12/2020	H16.9	1	Queratitis	2
1	04/12/2020	H20	6	Uveitis	2
1	04/12/2020	H20	6	Uveitis	2
1	04/12/2020	H16.9	1	Queratitis	2
2	04/12/2020	H16.9	1	Queratitis	2
2	04/12/2020	H00.02	4	Orzuelo	2
2	04/12/2020	H59.3	4	Post op párpados	2
2	04/12/2020	H46	10	Neuritis óptica	2
2	04/12/2020	H01.00	4	Blefaritis	2
1	04/12/2020	H16.9	1	Queratitis	2
2	04/12/2020	H16.9	1	Queratitis	2
1	04/12/2020	H16.9	1	Queratitis	2
2	04/12/2020	H00.02	4	Orzuelo	2
1	04/12/2020	H43.81	7	DVP	2
2	04/12/2020	H16.9	1	Queratitis	2
2	04/12/2020	H26.9	2	Catarata	2
1	04/12/2020	H16.0	1	Úlcera corneal	2
2	04/12/2020	H11.3	0	Hiposfagma	2
2	04/12/2020	H00.03	4	Celulitis preseptal	2
2	04/12/2020	H33.30	8	Desgarro retiniano	2
2	04/12/2020	H10.3	0	Conjuntivitis	2
1	04/12/2020	T15.0	1	Cuerpo extraño	2
2	04/12/2020	H16.9	1	Queratitis	2
1	04/12/2020	H16.0	1	Úlcera corneal	2
1	04/12/2020	H10.3	0	Conjuntivitis	2
1	04/12/2020	T15.0	1	Cuerpo extraño	2
2	04/12/2020	H00.03	4	Celulitis preseptal	2
2	04/12/2020	S05.9	9	Traumatismo	2
2	04/12/2020	H43.81	7	DVP	2
1	04/12/2020	H11.3	0	Hiposfagma	2
1	04/12/2020	S05.9	9	Traumatismo	2
1	04/12/2020	S05.30	9	Laceración conjuntival	2
1	05/12/2020	H16.0	1	Úlcera corneal	2
1	05/12/2020	H16.9	1	Queratitis	2
2	05/12/2020	H10.5	0	Blefaroconjuntivitis	2
1	05/12/2020	T15.0	1	Cuerpo extraño	2
1	05/12/2020	H10.3	0	Conjuntivitis	2
1	05/12/2020	H16.9	1	Queratitis	2
1	05/12/2020	H10.3	0	Conjuntivitis	2
2	05/12/2020	H20	6	Uveitis	2
1	05/12/2020	B00.1	4	Dermatitis herpética	2
2	05/12/2020	H10.3	0	Conjuntivitis	2
2	05/12/2020	H26.9	2	Catarata	2
1	05/12/2020	H33.0	8	Desprendimiento de retina	2
1	05/12/2020	H16.0	1	Úlcera corneal	2
1	05/12/2020	H43.81	7	DVP	2
1	05/12/2020	H16.9	1	Queratitis	2
2	05/12/2020	H10.3	0	Conjuntivitis	2
1	05/12/2020	H02	4	Alteración palpebral	2
2	05/12/2020	H16.9	1	Queratitis	2
1	05/12/2020	T15.0	1	Cuerpo extraño	2
2	05/12/2020	H11.3	0	Hiposfagma	2
2	05/12/2020	H16.9	1	Queratitis	2
2	05/12/2020	H43.81	7	DVP	2
1	05/12/2020	T15.0	1	Cuerpo extraño	2
1	05/12/2020	H16.9	1	Queratitis	2
1	05/12/2020	H16.0	1	Úlcera corneal	2
1	05/12/2020	T15.0	1	Cuerpo extraño	2
2	05/12/2020	H16.9	1	Queratitis	2
1	05/12/2020	H16.9	1	Queratitis	2
2	05/12/2020	H16.9	1	Queratitis	2
2	05/12/2020	H16.0	1	Úlcera corneal	2
2	05/12/2020	H20	6	Uveitis	2
1	05/12/2020	H00.02	4	Orzuelo	2

2	05/12/2020	H16.9	1	Queratitis	2
2	05/12/2020	H44.009	8	Endoftalmitis	2
2	05/12/2020	H10.3	0	Conjuntivitis	2
2	05/12/2020	H16.0	1	Úlcera corneal	2
2	06/12/2020	H16.0	1	Úlcera corneal	2
1	06/12/2020	T15.0	1	Cuerpo extraño	2
1	06/12/2020	H11.3	0	Hiposfagma	2
1	06/12/2020	H11.3	0	Hiposfagma	2
2	06/12/2020	H16.0	1	Úlcera corneal	2
2	06/12/2020	H43.81	7	DVP	2
1	06/12/2020	H20	6	Uveitis	2
2	06/12/2020	H01.00	4	Blefaritis	2
1	06/12/2020	H16.9	1	Queratitis	2
2	06/12/2020	H00.02	4	Orzuelo	2
2	06/12/2020	H10.3	0	Conjuntivitis	2
1	06/12/2020	H16.0	1	Úlcera corneal	2
1	06/12/2020	H00.03	4	Celulitis preseptal	2
1	06/12/2020	H16.0	1	Úlcera corneal	2
2	06/12/2020	H16.9	1	Queratitis	2
1	06/12/2020	T15.0	1	Cuerpo extraño	2
1	06/12/2020	H59.3	4	Post op párpados	2
1	06/12/2020	H16.0	1	Úlcera corneal	2
1	06/12/2020	T15.0	1	Cuerpo extraño	2
2	06/12/2020	H00.02	4	Orzuelo	2
2	06/12/2020	H53.10	11	Problema refractivo	2
2	06/12/2020	H16.9	1	Queratitis	2
2	06/12/2020	H01.00	4	Blefaritis	2
1	06/12/2020	T15.0	1	Cuerpo extraño	2
1	06/12/2020	H33.0	8	Desprendimiento de retina	2
2	06/12/2020	S05.9	9	Traumatismo	2
1	06/12/2020	H16.0	1	Úlcera corneal	2
2	06/12/2020	H16.9	1	Queratitis	2
1	06/12/2020	H20	6	Uveitis	2
1	06/12/2020	T15.0	1	Cuerpo extraño	2
2	06/12/2020	H44.009	8	Endoftalmitis	2
2	07/12/2020	H11.3	0	Hiposfagma	2
1	07/12/2020	H59.3	5	Postop via lagrimal	2
2	07/12/2020	H49.9	10	Parálisis oculomotora	2
2	07/12/2020	H10.3	0	Conjuntivitis	2
1	07/12/2020	H00.02	4	Orzuelo	2
2	07/12/2020	H16.0	1	Úlcera corneal	2
1	07/12/2020	H00.02	4	Orzuelo	2
1	07/12/2020	H10.3	0	Conjuntivitis	2
2	07/12/2020	H16.0	1	Úlcera corneal	2
2	07/12/2020	H16.9	1	Queratitis	2
2	07/12/2020	H34.82	8	Trombosis venosa	2
2	07/12/2020	H43.81	7	DVP	2
2	07/12/2020	H10.3	0	Conjuntivitis	2
2	07/12/2020	H20	6	Uveitis	2
2	07/12/2020	H01.00	4	Blefaritis	2
1	07/12/2020	H10.3	0	Conjuntivitis	2
2	07/12/2020	H16.9	1	Queratitis	2
1	07/12/2020	H16.0	1	Úlcera corneal	2
1	07/12/2020	H20	6	Uveitis	2
1	07/12/2020	H16.9	1	Queratitis	2
1	07/12/2020	H43.81	7	DVP	2
2	07/12/2020	H16.9	1	Queratitis	2
1	07/12/2020	H01.00	4	Blefaritis	2
2	07/12/2020	H35.0	8	Hemorragia retiniana	2
1	07/12/2020	H15.00	6	Escleritis	2
2	07/12/2020	H16.0	1	Úlcera corneal	2
2	07/12/2020	H16.9	1	Queratitis	2
2	07/12/2020	H00.03	4	Celulitis preseptal	2
2	07/12/2020	H16.0	1	Úlcera corneal	2
1	07/12/2020	E11.319	8	Retinopatía diabética	2
1	07/12/2020	H43.81	7	DVP	2
2	07/12/2020	H16.9	1	Queratitis	2
2	07/12/2020	T15.0	1	Cuerpo extraño	2
1	07/12/2020	T15.0	1	Cuerpo extraño	2
1	07/12/2020	T15.0	1	Cuerpo extraño	2
2	07/12/2020	H15.00	6	Escleritis	2

1	08/12/2020	T15.0	1	Cuerpo extraño	2
2	08/12/2020	H16.0	1	Úlcera corneal	2
2	08/12/2020	H10.3	0	Conjuntivitis	2
2	08/12/2020	H16.9	1	Queratitis	2
2	08/12/2020	G43.9	10	Migraña	2
1	08/12/2020	H00.02	4	Orzuelo	2
1	08/12/2020	H16.0	1	Úlcera corneal	2
2	08/12/2020	H16.9	1	Queratitis	2
1	08/12/2020	T15.0	1	Cuerpo extraño	2
2	08/12/2020	H44.009	8	Endoftalmitis	2
1	08/12/2020	H11.3	0	Hiposfagma	2
2	08/12/2020	H33.30	8	Desgarro retiniano	2
2	08/12/2020	H16.0	1	Úlcera corneal	2
1	08/12/2020	H10.3	0	Conjuntivitis	2
2	08/12/2020	H33.30	8	Desgarro retiniano	2
1	08/12/2020	H16.9	1	Queratitis	2
2	08/12/2020	H16.0	1	Úlcera corneal	2
2	08/12/2020	H11.3	0	Hiposfagma	2
1	08/12/2020	H10.3	0	Conjuntivitis	2
2	08/12/2020	H59.88	8	Post op retina	2
1	08/12/2020	H16.0	1	Úlcera corneal	2
2	08/12/2020	H16.9	1	Queratitis	2
2	08/12/2020	G43.109	11	Aura	2
1	08/12/2020	T15.0	1	Cuerpo extraño	2
2	08/12/2020	H16.0	1	Úlcera corneal	2
1	08/12/2020	H02	4	Lesión palpebral	2
1	08/12/2020	H16.9	1	Queratitis	2
2	08/12/2020	H16.9	1	Queratitis	2
2	08/12/2020	H16.0	1	Úlcera corneal	2
2	08/12/2020	H20	6	Uveitis	2
1	08/12/2020	S05.9	9	Traumatismo	2
2	08/12/2020	H10.3	0	Conjuntivitis	2
2	08/12/2020	T15.0	1	Cuerpo extraño	2
1	08/12/2020	H16.9	1	Queratitis	2
2	08/12/2020	H16.0	1	Úlcera corneal	2
2	08/12/2020	H16.9	1	Queratitis	2
1	08/12/2020	H16.0	1	Úlcera corneal	2
1	08/12/2020	H00.02	4	Orzuelo	2
1	08/12/2020	H53.10	11	Problema refractivo	2
1	08/12/2020	T15.0	1	Cuerpo extraño	2
1	09/12/2020	H16.0	1	Úlcera corneal	2
2	09/12/2020	H16.9	1	Queratitis	2
2	09/12/2020	H43.81	7	DVP	2
2	09/12/2020	H11.3	0	Hiposfagma	2
2	09/12/2020	H43.81	7	DVP	2
2	09/12/2020	H01.00	4	Blefaritis	2
2	09/12/2020	H17.8	1	Infiltrados corneales	2
2	09/12/2020	H16.0	1	Úlcera corneal	2
1	09/12/2020	H16.0	1	Úlcera corneal	2
2	09/12/2020	H01.00	4	Blefaritis	2
2	09/12/2020	H16.0	1	Úlcera corneal	2
1	09/12/2020	H35.37	11	MER	2
2	09/12/2020	H16.9	1	Queratitis	2
2	09/12/2020	G43.9	10	Migraña	2
2	09/12/2020	Z98.4	11	Post op catarata	2
2	09/12/2020	H16.9	1	Queratitis	2
2	09/12/2020	H10.3	0	Conjuntivitis	2
1	09/12/2020	H35.30	8	DMAE	2
2	09/12/2020	H02	4	Alteración palpebral	2
1	09/12/2020	B00.1	4	Dermatitis herpética	2
1	09/12/2020	H16.9	1	Queratitis	2
2	09/12/2020	H43.81	7	DVP	2
1	09/12/2020	H01.00	4	Blefaritis	2
1	09/12/2020	H16.0	1	Úlcera corneal	2
1	09/12/2020	T15.0	1	Cuerpo extraño	2
1	09/12/2020	H16.0	1	Úlcera corneal	2
1	09/12/2020	Alta por fuga	11	Alta por fuga	2
1	09/12/2020	T15.0	1	Cuerpo extraño	2
2	09/12/2020	H00.02	4	Orzuelo	2
2	09/12/2020	H16.0	0	Úlcera corneal	2
2	09/12/2020	S05.9	9	Traumatismo	2

2	09/12/2020	H16.9	1	Queratitis	2
2	09/12/2020	H16.0	1	Úlcera corneal	2
1	10/12/2020	H43.81	7	DVP	2
2	10/12/2020	H11.3	0	Hiposfagma	2
1	10/12/2020	Z98.4	2	Post op catarata	2
2	10/12/2020	H01.00	4	Blefaritis	2
2	10/12/2020	H10.3	0	Conjuntivitis	2
1	10/12/2020	H10.3	0	Conjuntivitis	2
1	10/12/2020	T15.0	1	Cuerpo extraño	2
1	10/12/2020	H27.8	2	OCP	2
2	10/12/2020	H10.3	0	Conjuntivitis	2
2	10/12/2020	H16.9	1	Queratitis	2
2	10/12/2020	T15.0	1	Cuerpo extraño	2
1	10/12/2020	H16.0	1	Úlcera corneal	2
1	10/12/2020	H10.3	0	Conjuntivitis	2
2	10/12/2020	H35.3	8	Maculopatía	2
2	10/12/2020	B00.1	4	Dermatitis herpética	2
2	10/12/2020	H16.9	1	Queratitis	2
2	10/12/2020	H26.9	2	Catarata	2
2	10/12/2020	H0.41	1	Ojo seco	2
1	10/12/2020	H00.02	4	Orzuelo	2
1	10/12/2020	H10.3	0	Conjuntivitis	2
2	10/12/2020	H01.00	4	Blefaritis	2
1	10/12/2020	H27.8	2	OCP	2
1	10/12/2020	H10.3	0	Conjuntivitis	2
2	10/12/2020	B00.1	4	Dermatitis herpética	2
1	10/12/2020	H43.81	7	DVP	2
1	10/12/2020	H43.81	7	DVP	2
2	10/12/2020	H16.9	1	Queratitis	2
2	10/12/2020	H04.32	5	Dacriocistitis aguda	2
1	10/12/2020	H01.00	4	Blefaritis	2
2	10/12/2020	T15.0	1	Cuerpo extraño	2
1	10/12/2020	H01.00	4	Blefaritis	2
1	10/12/2020	H50.9	10	Estrabismo	2
1	10/12/2020	T15.0	1	Cuerpo extraño	2
1	10/12/2020	E11.319	8	Retinopatía diabética	2
1	10/12/2020	H16.0	1	Úlcera corneal	2
2	10/12/2020	H10.3	0	Conjuntivitis	2
1	10/12/2020	H43.81	7	DVP	2
1	10/12/2020	H10.3	0	Conjuntivitis	2
1	10/12/2020	H01.00	4	Blefaritis	2
1	10/12/2020	H01.00	4	Blefaritis	2
1	11/12/2020	H05.01	4	Celulitis orbitaria	2
1	11/12/2020	H16.0	1	Úlcera corneal	2
1	11/12/2020	Alta por fuga	11	Alta por fuga	2
2	11/12/2020	H01.00	4	Blefaritis	2
2	11/12/2020	H16.0	1	Úlcera corneal	2
2	11/12/2020	H16.0	1	Úlcera corneal	2
2	11/12/2020	H10.3	0	Conjuntivitis	2
2	11/12/2020	H43.81	7	DVP	2
2	11/12/2020	H16.0	1	Úlcera corneal	2
1	11/12/2020	H16.9	1	Queratitis	2
1	11/12/2020	H16.0	1	Úlcera corneal	2
2	11/12/2020	S05.9	9	Traumatismo	2
2	11/12/2020	H00.02	4	Orzuelo	2
2	11/12/2020	H10.3	0	Conjuntivitis	2
1	11/12/2020	H46	10	Neuritis óptica	2
2	11/12/2020	H16.9	1	Queratitis	2
2	11/12/2020	H16.9	1	Queratitis	2
1	11/12/2020	H16.9	1	Queratitis	2
2	11/12/2020	H40.21	3	Glaucoma agudo	2
2	11/12/2020	H00.02	4	Orzuelo	2
1	11/12/2020	H00.02	4	Orzuelo	2
2	11/12/2020	H43.81	7	DVP	2
1	11/12/2020	H27.8	2	OCP	2
1	11/12/2020	H10.3	0	Conjuntivitis	2
2	11/12/2020	H43.81	7	DVP	2
2	11/12/2020	H16.9	1	Queratitis	2
2	11/12/2020	H16.9	1	Queratitis	2
2	11/12/2020	H35.30	8	DMAE	2
2	11/12/2020	H46	10	Neuritis óptica	2

1	11/12/2020	H00.02	4	Orzuelo	2
2	11/12/2020	H20	6	Uveitis	2
2	11/12/2020	H16.9	1	Queratitis	2
1	11/12/2020	H16.9	1	Queratitis	2
1	11/12/2020	H17.8	1	Infiltrados corneales	2
2	11/12/2020	H10.3	0	Conjuntivitis	2
1	11/12/2020	H16.9	1	Queratitis	2
2	11/12/2020	H35.0	8	Hemorragia retiniana	2
2	11/12/2020	H40.05	3	HTO	2
2	11/12/2020	H20	6	Uveitis	2
1	11/12/2020	T15.0	1	Cuerpo extraño	2
2	11/12/2020	H43.81	7	DVP	2
2	11/12/2020	S05.30	9	Laceración conjuntival	2
1	11/12/2020	G43.9	10	Migraña	2
2	11/12/2020	S05.30	9	Laceración conjuntival	2
1	12/12/2020	B58.0	8	Toxoplasmosis	2
1	12/12/2020	H20	6	Uveitis	2
1	12/12/2020	H16.0	1	Úlcera corneal	2
1	12/12/2020	T15.0	1	Cuerpo extraño	2
1	12/12/2020	H20	6	Uveitis	2
1	12/12/2020	H16.9	1	Queratitis	2
2	12/12/2020	G43.9	10	Migraña	2
2	12/12/2020	H17.8	1	Infiltrados corneales	2
2	12/12/2020	H00.03	4	Celulitis preseptal	2
2	12/12/2020	H16.9	1	Queratitis	2
1	12/12/2020	H43.81	7	DVP	2
1	12/12/2020	H16.0	1	Úlcera corneal	2
1	12/12/2020	H16.0	1	Úlcera corneal	2
2	12/12/2020	H35.30	8	DMAE	2
1	12/12/2020	H11.3	0	Hiposfagma	2
2	12/12/2020	H11.3	0	Hiposfagma	2
1	12/12/2020	H43.81	7	DVP	2
2	12/12/2020	H16.9	1	Queratitis	2
1	12/12/2020	T15.0	1	Cuerpo extraño	2
1	12/12/2020	H16.0	1	Úlcera corneal	2
2	12/12/2020	B00.1	4	Dermatitis herpética	2
2	12/12/2020	H05.01	4	Celulitis orbitaria	2
1	12/12/2020	H10.3	0	Conjuntivitis	2
1	12/12/2020	H27.8	2	OCP	2
2	12/12/2020	H16.9	1	Queratitis	2
1	12/12/2020	T26	9	Causticación	2
1	12/12/2020	H20	6	Uveitis	2
1	12/12/2020	H16.9	1	Queratitis	2
2	12/12/2020	H16.9	1	Queratitis	2
2	12/12/2020	B00.1	4	Dermatitis herpética	2
2	12/12/2020	B00.1	4	Dermatitis herpética	2
1	12/12/2020	T15.0	1	Cuerpo extraño	2
2	12/12/2020	H11.3	0	Hiposfagma	2
1	13/12/2020	H16.0	1	Úlcera corneal	2
1	13/12/2020	H16.9	1	Queratitis	2
1	13/12/2020	H16.0	1	Úlcera corneal	2
1	13/12/2020	H16.0	1	Úlcera corneal	2
2	13/12/2020	H11.3	0	Hiposfagma	2
2	13/12/2020	H16.0	1	Úlcera corneal	2
1	13/12/2020	H00.02	4	Orzuelo	2
2	13/12/2020	H35.30	8	DMAE	2
2	13/12/2020	H10.3	0	Conjuntivitis	2
2	13/12/2020	H20	6	Uveitis	2
2	13/12/2020	H10.3	0	Conjuntivitis	2
2	13/12/2020	H43.81	7	DVP	2
2	13/12/2020	H43.81	7	DVP	2
1	13/12/2020	H00.02	4	Orzuelo	2
2	13/12/2020	H01.00	4	Blefaritis	2
2	13/12/2020	H00.02	4	Orzuelo	2
1	13/12/2020	H15.00	6	Escleritis	2
2	13/12/2020	H10.3	0	Conjuntivitis	2
1	13/12/2020	H40.05	3	HTO	2
1	13/12/2020	H11.3	0	Hiposfagma	2
2	13/12/2020	H00.02	4	Orzuelo	2
2	13/12/2020	H43.81	7	DVP	2
1	13/12/2020	T15.0	1	Cuerpo extraño	2

2	13/12/2020	H00.02	4	Orzuelo	2
2	13/12/2020	H59.3	4	Post op párpados	2
2	13/12/2020	H00.02	4	Orzuelo	2
2	13/12/2020	H01.00	4	Blefaritis	2
2	13/12/2020	H26.9	2	Catarata	2
2	13/12/2020	H16.0	1	Úlcera corneal	2
2	13/12/2020	T15.0	1	Cuerpo extraño	2
2	13/12/2020	H43.81	7	DVP	2
2	13/12/2020	H43.81	7	DVP	2
2	13/12/2020	H40.9	3	Glaucoma	2
1	13/12/2020	H16.0	1	Úlcera corneal	2
2	13/12/2020	H11.3	0	Hiposfagma	2
2	14/12/2020	H16.0	1	Úlcera corneal	2
1	14/12/2020	H00.02	4	Orzuelo	2
2	14/12/2020	H10.3	0	Conjuntivitis	2
1	14/12/2020	H35.30	8	DMAE	2
2	14/12/2020	H27.8	2	OCP	2
2	14/12/2020	H16.0	1	Úlcera corneal	2
2	14/12/2020	H01.00	4	Blefaritis	2
1	14/12/2020	T15.0	1	Cuerpo extraño	2
2	14/12/2020	H11.3	0	Hiposfagma	2
2	14/12/2020	H10.3	0	Conjuntivitis	2
2	14/12/2020	H01.00	4	Blefaritis	2
1	14/12/2020	H16.0	1	Úlcera corneal	2
2	14/12/2020	H10.81	0	Pingueculitis	2
2	14/12/2020	H20	6	Uveitis	2
2	14/12/2020	H16.0	1	Úlcera corneal	2
1	14/12/2020	H10.3	0	Conjuntivitis	2
2	14/12/2020	H10.3	0	Conjuntivitis	2
2	14/12/2020	H16.0	1	Úlcera corneal	2
2	14/12/2020	T15.0	1	Cuerpo extraño	2
2	14/12/2020	H16.9	1	Queratitis	2
1	14/12/2020	H16.9	1	Queratitis	2
2	14/12/2020	H43.81	7	DVP	2
1	14/12/2020	H11.3	0	Hiposfagma	2
2	14/12/2020	H27.8	2	OCP	2
1	14/12/2020	H10.3	0	Conjuntivitis	2
1	14/12/2020	S05.30	9	Laceración conjuntival	2
2	14/12/2020	H16.9	1	Queratitis	2
1	14/12/2020	T15.0	1	Cuerpo extraño	2
1	14/12/2020	H16.0	1	Úlcera corneal	2
1	14/12/2020	H43.81	7	DVP	2
2	14/12/2020	H43.81	7	DVP	2
1	14/12/2020	T15.0	1	Cuerpo extraño	2
2	14/12/2020	H16.0	1	Úlcera corneal	2
2	14/12/2020	B00.1	4	Dermatitis herpética	2
2	14/12/2020	H11.3	0	Hiposfagma	2
1	14/12/2020	H16.9	1	Queratitis	2
1	15/12/2020	H43.81	7	DVP	2
2	15/12/2020	H15.00	6	Escleritis	2
1	15/12/2020	H00.02	4	Orzuelo	2
1	15/12/2020	H16.9	1	Queratitis	2
2	15/12/2020	H16.9	1	Queratitis	2
2	15/12/2020	H01.00	4	Blefaritis	2
1	15/12/2020	H16.9	1	Queratitis	2
2	15/12/2020	H16.9	1	Queratitis	2
1	15/12/2020	T15.0	1	Cuerpo extraño	2
1	15/12/2020	H02	4	Alteración palpebral	2
1	15/12/2020	H11.3	0	Hiposfagma	2
1	15/12/2020	S05.30	9	Laceración conjuntival	2
1	15/12/2020	H10.3	0	Conjuntivitis	2
2	15/12/2020	H16.9	1	Queratitis	2
1	15/12/2020	H16.0	1	Úlcera corneal	2
1	15/12/2020	H43.81	7	DVP	2
1	15/12/2020	T15.0	1	Cuerpo extraño	2
2	15/12/2020	E11.319	8	Retinopatía diabética	2
2	15/12/2020	H43.81	7	DVP	2
2	15/12/2020	H16.9	1	Queratitis	2
2	15/12/2020	H20	6	Uveitis	2
1	15/12/2020	H16.0	1	Úlcera corneal	2
1	15/12/2020	H10.3	0	Conjuntivitis	2

1	15/12/2020	H00.02	4	Orzuelo	2
2	15/12/2020	H16.9	1	Queratitis	2
1	15/12/2020	H10.3	0	Conjuntivitis	2
1	15/12/2020	H16.0	1	Úlcera corneal	2
1	15/12/2020	H16.9	1	Queratitis	2
1	15/12/2020	H16.9	1	Queratitis	2
1	15/12/2020	T26.4	9	Quemadura	2
2	15/12/2020	Alta por fuga	11	Alta por fuga	2
1	16/12/2020	H15.00	6	Escleritis	2
1	16/12/2020	H16.0	1	Úlcera corneal	2
2	16/12/2020	H20	6	Uveitis	2
1	16/12/2020	T15.0	1	Cuerpo extraño	2
2	16/12/2020	H15.1	6	Epiescleritis	2
2	16/12/2020	T26	9	Causticación	2
1	16/12/2020	H16.9	1	Queratitis	2
2	16/12/2020	Z98.4	2	Post op catarata	2
1	16/12/2020	H20	6	Uveitis	2
2	16/12/2020	H16.9	1	Queratitis	2
2	16/12/2020	H35.30	8	DMAE	2
2	16/12/2020	H16.9	1	Queratitis	2
2	16/12/2020	H27.8	2	OCP	2
2	16/12/2020	H16.0	1	Úlcera corneal	2
2	16/12/2020	H20	6	Uveitis	2
1	16/12/2020	H16.0	1	Úlcera corneal	2
1	16/12/2020	H16.0	1	Úlcera corneal	2
2	16/12/2020	H43.81	7	DVP	2
2	16/12/2020	H35.37	8	MER	2
2	16/12/2020	H43.81	7	DVP	2
1	16/12/2020	H16.9	1	Queratitis	2
1	16/12/2020	H16.9	1	Queratitis	2
2	16/12/2020	H16.0	1	Úlcera corneal	2
2	16/12/2020	H43.81	7	DVP	2
2	16/12/2020	H16.0	1	Úlcera corneal	2
2	16/12/2020	G43.9	10	Migraña	2
1	16/12/2020	H11.3	0	Hiposfagma	2
1	16/12/2020	H16.0	1	Úlcera corneal	2
1	16/12/2020	H11.3	0	Hiposfagma	2
1	16/12/2020	H16.0	1	Úlcera corneal	2
2	16/12/2020	H04.32	5	Dacriocistitis aguda	2
1	16/12/2020	S05.6	9	Perforación ocular	2
1	16/12/2020	T15.0	1	Cuerpo extraño	2
1	16/12/2020	H16.0	1	Úlcera corneal	2
2	16/12/2020	H16.0	1	Úlcera corneal	2
2	16/12/2020	Z98.4	2	Post op catarata	2
1	16/12/2020	G43.9	10	Migraña	2
1	16/12/2020	H20	6	Uveitis	2
2	16/12/2020	H20	6	Uveitis	2
1	16/12/2020	S05.9	9	Traumatismo	2
1	16/12/2020	T15.0	1	Cuerpo extraño	2
1	16/12/2020	T15.0	1	Cuerpo extraño	2
2	17/12/2020	H10.3	0	Conjuntivitis	2
1	17/12/2020	H59.3	5	Postop via lagrimal	2
1	17/12/2020	H16.0	1	Úlcera corneal	2
1	17/12/2020	H16.0	0	Úlcera corneal	2
1	17/12/2020	H0.41	1	ojo seco	2
2	17/12/2020	H0.41	1	ojo seco	2
2	17/12/2020	H35.3	8	Maculopatía	2
2	17/12/2020	H16.9	1	Queratitis	2
2	17/12/2020	H10.3	0	Conjuntivitis	2
2	17/12/2020	H10.3	0	Conjuntivitis	2
1	17/12/2020	S05.6	9	Perforación ocular	2
1	17/12/2020	H20	6	Uveitis	2
1	17/12/2020	H11.3	0	Hiposfagma	2
2	17/12/2020	H43.81	7	DVP	2
1	17/12/2020	H10.3	0	Conjuntivitis	2
2	17/12/2020	Z98.4	2	Post op catarata	2
1	17/12/2020	H0.41	1	Ojo seco	2
1	17/12/2020	H16.0	1	Úlcera corneal	2
2	17/12/2020	H16.9	1	Queratitis	2
2	17/12/2020	H43.81	7	DVP	2
1	17/12/2020	H43.81	7	DVP	2

2	17/12/2020	H10.3	0	Conjuntivitis		2
1	17/12/2020	H15.00	6	Escleritis		2
2	17/12/2020	H11.3	0	Hiposfagma		2
2	17/12/2020	H00.02	4	Orzuelo		2
1	17/12/2020	S05.9	9	Traumatismo		2
1	17/12/2020	H10.3	0	Conjuntivitis		2
1	17/12/2020	T15.0	1	Cuerpo extraño		2
2	17/12/2020	H16.0	1	Úlcera corneal		2
1	17/12/2020	H16.0	1	Úlcera corneal		2
2	17/12/2020	H16.0	1	Úlcera corneal		2
1	17/12/2020	T15.0	1	Cuerpo extraño		2
1	17/12/2020	H10.3	0	Conjuntivitis		2
1	17/12/2020	T15.0	1	Cuerpo extraño		2
2	18/12/2020	H43.1	7	Hemovítreo		2
1	18/12/2020	H16.9	1	Queratitis		2
2	18/12/2020	H40.9	3	Glaucoma		2
2	18/12/2020	H10.3	0	Conjuntivitis		2
2	18/12/2020	H16.9	1	Queratitis		2
1	18/12/2020	H02	4	Alteración palpebral		2
1	18/12/2020	H16.9	1	Queratitis		2
2	18/12/2020	H0.41	1	ojo seco		2
1	18/12/2020	H16.0	1	Úlcera corneal		2
2	18/12/2020	H16.9	1	Queratitis		2
1	18/12/2020	H11.3	0	Hiposfagma		2
1	18/12/2020	H10.3	0	Conjuntivitis		2
1	18/12/2020	H16.9	1	Queratitis		2
1	18/12/2020	H00.02	4	Orzuelo		2
1	18/12/2020	H10.3	0	Conjuntivitis		2
1	18/12/2020	H16.0	1	Úlcera corneal		2
2	18/12/2020	H04.41	5	Dacriocistitis crónica		2
1	18/12/2020	H43.81	7	DVP		2
2	18/12/2020	Z97.0	8	Protesis ocular		2
1	18/12/2020	H16.0	1	Úlcera corneal		2
1	18/12/2020	H17.8	1	Infiltrados corneales		2
2	18/12/2020	H59.88	8	Post op retina		2
2	18/12/2020	H15.1	6	Epiescleritis		2
1	18/12/2020	H00.02	4	Orzuelo		2
1	18/12/2020	H53.9	11	Alteraciones visuales		2
2	18/12/2020	H43.81	7	DVP		2
2	18/12/2020	H16.9	1	Queratitis		2
1	18/12/2020	H16.0	1	Úlcera corneal		2
1	18/12/2020	H16.0	1	Úlcera corneal		2
2	18/12/2020	H16.9	1	Queratitis		2
2	18/12/2020	H10.3	0	Conjuntivitis		2
1	18/12/2020	Alta por fuga	11	Alta por fuga		2
1	19/12/2020	T15.0	1	Cuerpo extraño		2
2	19/12/2020	H43.81	7	DVP		2
2	19/12/2020	H02	4	Alteración palpebral		2
2	19/12/2020	H01.00	4	Blefaritis		2
2	19/12/2020	H35.30	8	DMAE		2
1	19/12/2020	T15.0	1	Cuerpo extraño		2
2	19/12/2020	H16.0	1	Úlcera corneal		2
2	19/12/2020	H27.8	2	OCP		2
2	19/12/2020	Z98.4	2	Post op catarata		2
2	19/12/2020	H00.02	4	Orzuelo		2
1	19/12/2020	E11.319	8	Retinopatía diabética		2
2	19/12/2020	H43.1	7	Hemovítreo		2
2	19/12/2020	H16.0	1	Úlcera corneal		2
2	19/12/2020	H0.41	1	Ojo seco		2
2	19/12/2020	H01.00	4	Blefaritis		2
2	19/12/2020	H16.0	1	Úlcera corneal		2
1	19/12/2020	H16.0	1	Úlcera corneal		2
2	19/12/2020	H43.81	7	DVP		2
1	19/12/2020	H00.02	4	Orzuelo		2
1	19/12/2020	S05.9	9	Traumatismo		2
2	19/12/2020	H43.81	7	DVP		2
1	19/12/2020	H00.02	4	Orzuelo		2
2	19/12/2020	H04.32	5	Dacriocistitis aguda		2
1	19/12/2020	S05.9	9	Traumatismo		2
1	19/12/2020	H40.05	3	HTO		2
2	19/12/2020	S05.9	9	Traumatismo		2

1	20/12/2020	H16.9	1	Queratitis	2
2	20/12/2020	H16.9	1	Queratitis	2
1	20/12/2020	H04.32	5	Dacriocistitis aguda	2
2	20/12/2020	H10.3	0	Conjuntivitis	2
2	20/12/2020	H10.3	0	Conjuntivitis	2
2	20/12/2020	H27.8	2	OCP	2
1	20/12/2020	H16.0	1	Úlcera corneal	2
1	20/12/2020	H10.81	0	Pingueculitis	2
1	20/12/2020	H16.0	1	Úlcera corneal	2
2	20/12/2020	H10.3	0	Conjuntivitis	2
1	20/12/2020	H43.81	7	DVP	2
1	20/12/2020	H10.3	0	Conjuntivitis	2
2	20/12/2020	H16.0	1	Úlcera corneal	2
2	20/12/2020	H11.3	0	Hiposfagma	2
2	20/12/2020	H16.0	1	Úlcera corneal	2
1	20/12/2020	T15.0	1	Cuerpo extraño	2
2	20/12/2020	H02	4	Alteración palpebral	2
1	20/12/2020	H10.3	0	Conjuntivitis	2
1	20/12/2020	S05.00	9	Abrasión conjuntival	2
2	20/12/2020	H16.0	1	Úlcera corneal	2
1	20/12/2020	T15.0	1	Cuerpo extraño	2
2	20/12/2020	H15.00	6	Escleritis	2
1	20/12/2020	H10.3	0	Conjuntivitis	2
2	20/12/2020	H16.0	1	Úlcera corneal	2
1	20/12/2020	H16.9	1	Queratitis	2
1	21/12/2020	T15.0	1	Cuerpo extraño	2
2	21/12/2020	H43.81	7	DVP	2
1	21/12/2020	H16.0	1	Úlcera corneal	2
2	21/12/2020	H11.3	0	Hiposfagma	2
2	21/12/2020	H20	6	Uveitis	2
2	21/12/2020	H10.3	0	Conjuntivitis	2
2	21/12/2020	H10.3	0	Conjuntivitis	2
1	21/12/2020	H35.30	8	DMAE	2
1	21/12/2020	H10.3	0	Conjuntivitis	2
2	21/12/2020	H43.81	7	DVP	2
2	21/12/2020	H43.81	7	DVP	2
1	21/12/2020	H26.9	2	Catarata	2
2	21/12/2020	H16.9	1	Queratitis	2
2	21/12/2020	H33.30	8	Desgarro retiniano	2
2	21/12/2020	H11.3	0	Hiposfagma	2
1	21/12/2020	H16.0	1	Úlcera corneal	2
2	21/12/2020	H35.0	8	Hemorragia retiniana	2
1	21/12/2020	H16.9	1	Queratitis	2
2	21/12/2020	H35.3	8	Maculopatía	2
1	21/12/2020	S05.9	9	Traumatismo	2
1	21/12/2020	H11.3	0	Hiposfagma	2
1	21/12/2020	H10.3	0	Conjuntivitis	2
1	21/12/2020	H16.0	1	Úlcera corneal	2
1	21/12/2020	H16.0	1	Úlcera corneal	2
1	21/12/2020	H16.9	1	Queratitis	2
2	21/12/2020	H11.3	0	Hiposfagma	2
1	21/12/2020	T15.0	1	Cuerpo extraño	2
1	21/12/2020	T15.0	1	Cuerpo extraño	2
2	21/12/2020	S05.9	9	Traumatismo	2
2	21/12/2020	H16.0	1	Úlcera corneal	2
2	21/12/2020	H10.3	0	Conjuntivitis	2
1	21/12/2020	T15.0	1	Cuerpo extraño	2
2	22/12/2020	H16.9	1	Queratitis	2
2	22/12/2020	S05.9	9	Traumatismo	2
2	22/12/2020	H16.3	1	Abceso corneal	2
2	22/12/2020	H16.0	1	Úlcera corneal	2
1	22/12/2020	G43.9	10	Migraña	2
2	22/12/2020	H40.5	3	Glaucoma secundario	2
1	22/12/2020	H30.9	6	Uveitis posterior	2
2	22/12/2020	H27.8	2	OCP	2
1	22/12/2020	H00.03	4	Celulitis preseptal	2
1	22/12/2020	H00.02	4	Orzuelo	2
1	22/12/2020	H00.03	4	Celulitis preseptal	2
1	22/12/2020	H01.00	4	Blefaritis	2
2	22/12/2020	H01.00	4	Blefaritis	2
2	22/12/2020	H01.00	4	Blefaritis	2

1	22/12/2020	S05.9	9	Traumatismo		2
2	22/12/2020	H10.3	0	Conjuntivitis		2
2	22/12/2020	H16.0	1	Úlcera corneal		2
2	22/12/2020	H04.32	5	Dacriocistitis aguda		2
2	22/12/2020	H16.9	1	Queratitis		2
1	22/12/2020	H16.0	1	Úlcera corneal		2
1	22/12/2020	T15.0	1	Cuerpo extraño		2
2	22/12/2020	H15.00	6	Escleritis		2
2	22/12/2020	H00.02	4	Orzuelo		2
1	22/12/2020	H16.9	1	Queratitis		2
2	22/12/2020	B00.1	4	Dermatitis herpética		2
2	22/12/2020	H10.3	0	Conjuntivitis		2
1	22/12/2020	H16.9	1	Queratitis		2
2	22/12/2020	S05.9	9	Traumatismo		2
1	23/12/2020	H16.9	1	Queratitis		2
1	23/12/2020	H16.9	1	Queratitis		2
1	23/12/2020	T15.0	1	Cuerpo extraño		2
2	23/12/2020	H43.81	7	DVP		2
2	23/12/2020	H10.3	0	Conjuntivitis		2
1	23/12/2020	H20	6	Uveitis		2
2	23/12/2020	H04.32	5	Dacriocistitis aguda		2
1	23/12/2020	H16.0	1	Úlcera corneal		2
1	23/12/2020	H00.02	4	Orzuelo		2
1	23/12/2020	H40.9	3	Glaucoma		2
2	23/12/2020	T15.0	1	Cuerpo extraño		2
1	23/12/2020	H00.03	4	Celulitis preseptal		2
2	23/12/2020	H49.9	10	Parálisis oculomotora		2
1	23/12/2020	H10.3	0	Conjuntivitis		2
1	23/12/2020	H43.81	7	DVP		2
1	23/12/2020	H16.0	1	Úlcera corneal		2
1	23/12/2020	H20	6	Uveitis		2
1	23/12/2020	H01.00	4	Blefaritis		2
1	23/12/2020	E11.319	8	Retinopatía diabética		2
2	23/12/2020	H16.9	1	Queratitis		2
2	23/12/2020	H43.81	7	DVP		2
2	23/12/2020	H10.3	0	Conjuntivitis		2
1	23/12/2020	S05.30	9	Laceración conjuntival		2
1	23/12/2020	T15.0	1	Cuerpo extraño		2
2	23/12/2020	H46	10	Neuritis óptica		2
1	23/12/2020	H17.8	1	Infiltrados corneales		2
2	23/12/2020	S05.30	9	Laceración conjuntival		2
1	23/12/2020	T15.0	1	Cuerpo extraño		2
1	23/12/2020	S05.9	9	Traumatismo		2
2	23/12/2020	H43.81	7	DVP		2
2	23/12/2020	H16.9	1	Queratitis		2
2	24/12/2020	H16.9	1	Queratitis		2
1	24/12/2020	H53.10	11	Problema refractivo		2
1	24/12/2020	H15.00	6	Escleritis		2
2	24/12/2020	H00.03	4	Celulitis preseptal		2
1	24/12/2020	H0.41	1	Ojo seco		2
2	24/12/2020	H10.3	0	Conjuntivitis		2
1	24/12/2020	H16.0	1	Úlcera corneal		2
2	24/12/2020	H16.3	1	Abceso corneal		2
1	24/12/2020	H11.3	0	Hiposfagma		2
1	24/12/2020	T15.0	1	Cuerpo extraño		2
1	24/12/2020	H01.00	4	Blefaritis		2
1	24/12/2020	H11.3	0	Hiposfagma		2
2	24/12/2020	H11.3	0	Hiposfagma		2
2	24/12/2020	S05.30	9	Laceración conjuntival		2
2	24/12/2020	H16.9	1	Queratitis		2
2	24/12/2020	H15.00	6	Escleritis		2
2	24/12/2020	H16.9	1	Queratitis		2
2	24/12/2020	H11.3	0	Hiposfagma		2
2	24/12/2020	H27.8	2	OCP		2
1	24/12/2020	H01.00	4	Blefaritis		2
1	24/12/2020	Alta por fuga	11	Alta por fuga		2
1	25/12/2020	T15.0	1	Cuerpo extraño		2
2	25/12/2020	H16.9	1	Queratitis		2
1	25/12/2020	T15.0	1	Cuerpo extraño		2
1	25/12/2020	T15.0	1	Cuerpo extraño		2
2	25/12/2020	H16.9	1	Queratitis		2

2	25/12/2020	H44.009		8	Endoftalmitis	2
1	25/12/2020	H16.0		1	Úlcera corneal	2
2	25/12/2020	H16.0		1	Úlcera corneal	2
2	25/12/2020	H16.0		1	Úlcera corneal	2
1	25/12/2020	H16.0		1	Úlcera corneal	2
2	25/12/2020	H11.3		0	Hiposfagma	2
1	25/12/2020	S05.9		9	Traumatismo	2
2	25/12/2020	H00.02		4	Orzuelo	2
1	25/12/2020	H43.81		7	DVP	2
2	25/12/2020	H00.02		4	Orzuelo	2
2	25/12/2020	G43.109		10	Aura	2
1	25/12/2020	H11.3		0	Hiposfagma	2
1	25/12/2020	T15.0		1	Cuerpo extraño	2
2	25/12/2020	S05.30		9	Laceración conjuntival	2
2	25/12/2020	H16.9		1	Queratitis	2
2	25/12/2020	H04.41		5	Dacriocistitis crónica	2
1	25/12/2020	T15.0		1	Cuerpo extraño	2
1	25/12/2020	H16.9		1	Queratitis	2
2	26/12/2020	H16.9		1	Queratitis	2
2	26/12/2020	H43.81		7	DVP	2
2	26/12/2020	H43.81		7	DVP	2
2	26/12/2020	H00.02		4	Orzuelo	2
1	26/12/2020	T15.0		1	Cuerpo extraño	2
1	26/12/2020	H43.1		7	Hemovítreo	2
1	26/12/2020	Z98.4		2	Post op catarata	2
1	26/12/2020	H02		4	Alteración palpebral	2
1	26/12/2020	H59.88		8	Post op retina	2
2	26/12/2020	H16.0		1	Úlcera corneal	2
2	26/12/2020	H04.32		5	Dacriocistitis aguda	2
2	26/12/2020	T15.0		1	Cuerpo extraño	2
1	26/12/2020	S05.9		9	Traumatismo	2
1	26/12/2020	H20		6	Uveitis	2
2	26/12/2020	H00.02		4	Orzuelo	2
2	26/12/2020	H20		6	Uveitis	2
1	26/12/2020	H11.3		0	Hiposfagma	2
1	26/12/2020	T15.0		1	Cuerpo extraño	2
1	26/12/2020	H17.8		1	Infiltrados corneales	2
2	26/12/2020	H10.3		0	Conjuntivitis	2
2	26/12/2020	H11.3		0	Hiposfagma	2
1	26/12/2020	T15.0		1	Cuerpo extraño	2
2	26/12/2020	H01.00		4	Blefaritis	2
2	26/12/2020	H16.0		1	Úlcera corneal	2
1	26/12/2020	S05.9		9	Traumatismo	2
2	26/12/2020	H43.1		7	Hemovítreo	2
1	27/12/2020	S05.9		9	Traumatismo	2
2	27/12/2020	T15.0		1	Cuerpo extraño	2
2	27/12/2020	H16.9		1	Queratitis	2
2	27/12/2020	H16.9		1	Queratitis	2
2	27/12/2020	H43.81		7	DVP	2
2	27/12/2020	H16.0		1	Úlcera corneal	2
1	27/12/2020	H16.0		1	Úlcera corneal	2
2	27/12/2020	H44.009		8	Endoftalmitis	2
2	27/12/2020	H43.81		7	DVP	2
1	27/12/2020	H20		6	Uveitis	2
2	27/12/2020	S05.9		9	Traumatismo	2
2	27/12/2020	S05.9		9	Traumatismo	2
2	27/12/2020	H16.9		1	Queratitis	2
2	27/12/2020	H00.02		4	Orzuelo	2
2	27/12/2020	H00.02		4	Orzuelo	2
2	27/12/2020	H18.20		1	Edema corneal	2
1	27/12/2020	H02		4	Alteración palpebral	2
1	27/12/2020	S05.9		9	Traumatismo	2
2	27/12/2020	H16.0		1	Úlcera corneal	2
1	27/12/2020	H10.3		0	Conjuntivitis	2
2	27/12/2020	H35.3		8	Maculopatía	2
2	27/12/2020	H11.3		0	Hiposfagma	2
1	27/12/2020	S05.9		9	Traumatismo	2
2	27/12/2020	S05.9		9	Traumatismo	2
1	27/12/2020	H11.3		0	Hiposfagma	2
2	27/12/2020	H16.9		1	Queratitis	2
2	27/12/2020	H16.0		1	Úlcera corneal	2

1	27/12/2020	T15.0	1	Cuerpo extraño	2
1	27/12/2020	Alta por fuga	11	Alta por fuga	2
1	28/12/2020	H16.0	1	Úlcera corneal	2
2	28/12/2020	H11.3	0	Hiposfagma	2
2	28/12/2020	Alta por fuga	11	Alta por fuga	2
2	28/12/2020	H10.3	0	Conjuntivitis	2
1	28/12/2020	H16.0	1	Úlcera corneal	2
2	28/12/2020	H16.0	1	Úlcera corneal	2
1	28/12/2020	H40.9	3	Glaucoma	2
2	28/12/2020	H01.00	4	Blefaritis	2
1	28/12/2020	H43.81	7	DVP	2
2	28/12/2020	H43.81	7	DVP	2
2	28/12/2020	H10.5	0	Blefaroconjuntivitis	2
2	28/12/2020	H10.3	0	Conjuntivitis	2
2	28/12/2020	H01.00	4	Blefaritis	2
2	28/12/2020	H10.3	0	Conjuntivitis	2
2	28/12/2020	H00.19	4	Chalazión	2
1	28/12/2020	H16.0	1	Úlcera corneal	2
2	28/12/2020	H40.05	3	HTO	2
2	28/12/2020	H16.0	1	Úlcera corneal	2
2	28/12/2020	H43.81	7	DVP	2
2	28/12/2020	H04.32	5	Dacriocistitis aguda	2
1	28/12/2020	H01.00	4	Blefaritis	2
2	28/12/2020	H43.81	7	DVP	2
2	28/12/2020	H43.81	7	DVP	2
1	28/12/2020	H43.81	7	DVP	2
2	28/12/2020	H43.81	7	DVP	2
1	28/12/2020	T15.0	1	Cuerpo extraño	2
1	28/12/2020	H11.3	0	Hiposfagma	2
2	28/12/2020	H16.0	1	Úlcera corneal	2
2	28/12/2020	H11.3	0	Hiposfagma	2
2	28/12/2020	G43.9	10	Migraña	2
1	28/12/2020	B00.1	4	Dermatitis herpética	2
1	28/12/2020	S05.9	9	Traumatismo	2
1	28/12/2020	H43.1	7	Hemovítreo	2
1	28/12/2020	H00.03	4	Celulitis preseptal	2
1	28/12/2020	H16.9	1	Queratitis	2
2	29/12/2020	H16.0	1	Úlcera corneal	2
1	29/12/2020	T15.0	1	Cuerpo extraño	2
1	29/12/2020	H04.32	5	Dacriocistitis aguda	2
1	29/12/2020	H10.3	0	Conjuntivitis	2
2	29/12/2020	H16.0	1	Úlcera corneal	2
2	29/12/2020	H16.9	1	Queratitis	2
1	29/12/2020	H16.0	1	Úlcera corneal	2
2	29/12/2020	H18.20	1	Edema corneal	2
2	29/12/2020	H11.3	0	Hiposfagma	2
2	29/12/2020	H35.3	8	Maculopatía	2
1	29/12/2020	H10.3	0	Conjuntivitis	2
2	29/12/2020	H10.3	0	Conjuntivitis	2
1	29/12/2020	H43.81	7	DVP	2
2	29/12/2020	S05.9	9	Traumatismo	2
1	29/12/2020	H26.9	2	Catarata	2
1	29/12/2020	H16.9	1	Queratitis	2
2	29/12/2020	H16.9	1	Queratitis	2
2	29/12/2020	H00.02	4	Orzuelo	2
2	29/12/2020	H10.3	0	Conjuntivitis	2
1	29/12/2020	H50.9	10	Estrabismo	2
2	29/12/2020	H00.02	4	Orzuelo	2
2	29/12/2020	H01.00	4	Blefaritis	2
2	29/12/2020	H10.3	0	Conjuntivitis	2
2	29/12/2020	H16.9	1	Queratitis	2
1	29/12/2020	H16.0	1	Úlcera corneal	2
1	29/12/2020	H43.81	7	DVP	2
2	29/12/2020	H35.30	8	DMAE	2
2	29/12/2020	H11.3	0	Hiposfagma	2
2	29/12/2020	H16.9	1	Queratitis	2
2	29/12/2020	H17.8	1	Infiltrados corneales	2
2	29/12/2020	H16.9	1	Queratitis	2
2	29/12/2020	H00.02	4	Orzuelo	2
1	29/12/2020	T26	9	Causticación	2
1	29/12/2020	H20	6	Uveitis	2

1	29/12/2020	T15.0	1	Cuerpo extraño	2
1	29/12/2020	H10.3	0	Conjuntivitis	2
1	29/12/2020	H33.30	8	Desgarro retiniano	2
2	30/12/2020	H01.00	4	Blefaritis	2
2	30/12/2020	H10.3	0	Conjuntivitis	2
1	30/12/2020	H0.41	1	Ojo seco	2
1	30/12/2020	H16.9	1	Queratitis	2
2	30/12/2020	H35.30	8	DMAE	2
2	30/12/2020	H10.3	0	Conjuntivitis	2
2	30/12/2020	H10.81	0	Pingueculitis	2
1	30/12/2020	H16.0	1	Úlcera corneal	2
1	30/12/2020	H11.00	0	Pterigium	2
2	30/12/2020	H43.81	7	DVP	2
1	30/12/2020	H11.3	0	Hiposfagma	2
2	30/12/2020	H16.9	1	Queratitis	2
1	30/12/2020	E11.319	8	Retinopatía diabética	2
1	30/12/2020	H43.81	7	DVP	2
2	30/12/2020	H43.81	7	DVP	2
2	30/12/2020	H35.0	8	Hemorragia retiniana	2
1	30/12/2020	H16.0	1	Úlcera corneal	2
2	30/12/2020	H01.00	4	Blefaritis	2
1	30/12/2020	H16.9	1	Queratitis	2
2	30/12/2020	H16.9	1	Queratitis	2
1	30/12/2020	T15.0	1	Cuerpo extraño	2
1	31/12/2020	T26	9	Causticación	2
2	31/12/2020	H20	6	Uveitis	2
1	31/12/2020	H10.3	0	Conjuntivitis	2
1	31/12/2020	H27.1	2	Luxación catarata	2
2	31/12/2020	H16.0	1	Úlcera corneal	2
2	31/12/2020	H01.00	4	Blefaritis	2
2	31/12/2020	H16.9	1	Queratitis	2
2	31/12/2020	H16.9	1	Queratitis	2
1	31/12/2020	H16.0	1	Úlcera corneal	2
1	31/12/2020	H00.19	4	Chalazión	2
1	31/12/2020	S05.9	9	Traumatismo	2
2	31/12/2020	H00.02	4	Orzuelo	2
2	31/12/2020	H00.02	4	Orzuelo	2
1	31/12/2020	H10.3	0	Conjuntivitis	2
1	31/12/2020	T15.0	1	Cuerpo extraño	2
1	31/12/2020	H16.0	1	Úlcera corneal	2
1	31/12/2020	H11.00	0	Pterigium	2
1	31/12/2020	H17.8	1	Infiltrados corneales	2
1	31/12/2020	H33.0	8	Desprendimiento de retina	2
2	31/12/2020	H20	6	Uveitis	2
1	31/12/2020	Z98.4	11	Post op catarata	2
2	31/12/2020	H16.9	1	Queratitis	2
2	31/12/2020	H00.19	4	Chalazión	2
1	31/12/2020	H16.9	1	Queratitis	2
1	31/12/2020	S05.9	9	Traumatismo	2
2	31/12/2020	H43.81	7	DVP	2
2	31/12/2020	B00.1	4	Dermatitis herpética	2
1	01/01/2021	H16.9	1	Queratitis	2
1	01/01/2021	H10.3	0	Conjuntivitis	2
2	01/01/2021	B00.1	4	Dermatitis herpética	2
2	01/01/2021	H16.9	1	Queratitis	2
1	01/01/2021	H10.3	0	Conjuntivitis	2
1	01/01/2021	H10.3	0	Conjuntivitis	2
1	01/01/2021	H16.9	1	Queratitis	2
2	01/01/2021	H10.3	0	Conjuntivitis	2
1	01/01/2021	T15.0	1	Cuerpo extraño	2
2	01/01/2021	T26	9	Causticación	2
1	01/01/2021	H16.0	1	Úlcera corneal	2
2	01/01/2021	H10.3	0	Conjuntivitis	2
1	01/01/2021	H26.9	2	Catarata	2
2	01/01/2021	H43.81	7	DVP	2
2	01/01/2021	H01.00	4	Blefaritis	2
2	01/01/2021	T26	9	Causticación	2
2	01/01/2021	H16.9	1	Queratitis	2
2	01/01/2021	H16.9	1	Queratitis	2
1	01/01/2021	H16.0	1	Úlcera corneal	2
1	01/02/2021	H16.0	1	Úlcera corneal	2

1	01/02/2021	H26.9	2	Catarata	2
1	01/02/2021	H20	6	Uveitis	2
1	01/02/2021	H10.3	0	Conjuntivitis	2
2	01/02/2021	H11.3	0	Hiposfagma	2
2	01/02/2021	H16.0	1	Úlcera corneal	2
2	01/02/2021	H10.3	0	Conjuntivitis	2
2	01/02/2021	H10.3	0	Conjuntivitis	2
1	01/02/2021	H02	4	Alteración palpebral	2
2	01/02/2021	H01.00	4	Blefaritis	2
1	01/02/2021	H16.0	1	Úlcera corneal	2
2	01/02/2021	H10.3	0	Conjuntivitis	2
1	01/02/2021	H16.0	1	Úlcera corneal	2
1	01/02/2021	H16.0	1	Úlcera corneal	2
1	01/02/2021	H00.02	4	Orzuelo	2
2	01/02/2021	H16.9	1	Queratitis	2
1	01/02/2021	H10.3	0	Conjuntivitis	2
1	01/02/2021	H16.0	1	Úlcera corneal	2
1	01/02/2021	H20	6	Uveitis	2
2	01/02/2021	H00.02	4	Orzuelo	2
1	01/02/2021	T15.0	1	Cuerpo extraño	2
1	01/02/2021	H35.0	8	Hemorragia retiniana	2
2	01/02/2021	H34.82	8	Trombosis venosa	2
2	01/02/2021	H26.9	2	Catarata	2
1	01/02/2021	T15.0	1	Cuerpo extraño	2
2	01/02/2021	H00.03	4	Celulitis preseptal	2
1	01/02/2021	T26	9	Causticación	2
2	01/03/2021	H16.9	1	Queratitis	2
1	01/03/2021	H10.3	1	Conjuntivitis	2
1	01/03/2021	H16.9	1	Queratitis	2
2	01/03/2021	H16.0	1	Úlcera corneal	2
1	01/03/2021	H16.0	1	Úlcera corneal	2
2	01/03/2021	H10.3	0	Conjuntivitis	2
1	01/03/2021	H40.05	3	HTO	2
1	01/03/2021	H16.0	1	Úlcera corneal	2
2	01/03/2021	H10.3	0	Conjuntivitis	2
2	01/03/2021	H40.05	3	HTO	2
1	01/03/2021	H16.9	1	Queratitis	2
2	01/03/2021	H16.9	1	Queratitis	2
2	01/03/2021	H26.9	2	Catarata	2
2	01/03/2021	H10.3	0	Conjuntivitis	2
1	01/03/2021	H16.0	1	Úlcera corneal	2
2	01/03/2021	H00.02	4	Orzuelo	2
1	01/03/2021	H10.3	0	Conjuntivitis	2
2	01/03/2021	H16.0	1	Úlcera corneal	2
1	01/03/2021	H16.0	1	Úlcera corneal	2
2	01/03/2021	H00.02	4	Orzuelo	2
1	01/03/2021	H16.0	1	Úlcera corneal	2
2	01/03/2021	H35.37	8	MER	2
2	01/03/2021	H10.3	0	Conjuntivitis	2
1	01/03/2021	H43.81	7	DVP	2
1	01/03/2021	H16.0	1	Úlcera corneal	2
2	01/03/2021	H16.9	1	Queratitis	2
1	01/03/2021	S05.9	9	Traumatismo	2
2	01/03/2021	H00.02	4	Orzuelo	2
2	01/03/2021	H00.02	4	Orzuelo	2
1	01/03/2021	S05.9	9	Traumatismo	2
2	01/03/2021	H16.9	1	Queratitis	2
1	01/03/2021	H16.9	1	Queratitis	2
2	01/03/2021	S05.9	9	Traumatismo	2
2	01/03/2021	H05.01	4	Celulitis orbitaria	2
1	01/03/2021	H16.0	1	Úlcera corneal	2
1	01/03/2021	H16.9	1	Queratitis	2
1	01/04/2021	H05.01	4	Celulitis orbitaria	2
2	01/04/2021	H43.1	7	Hemovítreo	2
2	01/04/2021	H43.1	7	Hemovítreo	2
1	01/04/2021	S05.9	9	Traumatismo	2
1	01/04/2021	S05.9	9	Traumatismo	2
2	01/04/2021	H43.81	7	DVP	2
2	01/04/2021	H20	6	Uveitis	2
1	01/04/2021	H16.0	1	Úlcera corneal	2
1	01/04/2021	H02	4	Alteración palpebral	2

2	01/04/2021	H43.81	7	DVP	2
1	01/04/2021	H15.1	6	Epiescleritis	2
2	01/04/2021	H01.00	4	Blefaritis	2
2	01/04/2021	H43.81	7	DVP	2
1	01/04/2021	H35.30	8	DMAE	2
2	01/04/2021	H11.3	0	Hiposfagma	2
2	01/04/2021	H18.50	1	Distrofia corneal	2
2	01/04/2021	H15.1	6	Epiescleritis	2
2	01/04/2021	H10.3	0	Conjuntivitis	2
2	01/04/2021	H20	6	Uveitis	2
2	01/04/2021	H40.9	3	Glaucoma	2
2	01/04/2021	H10.3	0	Conjuntivitis	2
2	01/04/2021	H16.9	1	Queratitis	2
2	01/04/2021	H16.9	1	Queratitis	2
2	01/04/2021	G43.9	10	Migraña	2
2	01/04/2021	H10.3	0	Conjuntivitis	2
2	01/04/2021	H17.8	1	Infiltrados corneales	2
2	01/04/2021	S05.9	9	Traumatismo	2
2	01/04/2021	H10.81	0	Pingueculitis	2
1	01/04/2021	H16.0	1	Úlcera corneal	2
2	01/05/2021	H15.1	6	Epiescleritis	2
2	01/05/2021	H43.81	7	DVP	2
1	01/05/2021	G43.9	10	Migraña	2
1	01/05/2021	H16.0	1	Úlcera corneal	2
1	01/05/2021	T15.0	1	Cuerpo extraño	2
2	01/05/2021	H43.81	7	DVP	2
2	01/05/2021	H49.9	10	Parálisis oculomotora	2
1	01/05/2021	H43.1	7	Hemovítreo	2
2	01/05/2021	H10.3	0	Conjuntivitis	2
2	01/05/2021	H16.0	1	Úlcera corneal	2
1	01/05/2021	T15.0	1	Cuerpo extraño	2
1	01/05/2021	H10.3	0	Conjuntivitis	2
1	01/05/2021	H16.0	1	Úlcera corneal	2
2	01/05/2021	H16.0	1	Úlcera corneal	2
2	01/05/2021	T15.0	1	Cuerpo extraño	2
2	01/05/2021	S05.9	9	Traumatismo	2
1	01/05/2021	H43.81	7	DVP	2
1	01/05/2021	H10.3	0	Conjuntivitis	2
1	01/05/2021	H33.0	8	Desprendimiento de retina	2
2	01/05/2021	H34.82	8	Trombosis venosa	2
2	01/05/2021	H10.3	0	Conjuntivitis	2
1	01/05/2021	S05.9	9	Traumatismo	2
2	01/05/2021	H00.19	4	Chalazión	2
2	01/05/2021	H34.82	8	Trombosis venosa	2
1	01/05/2021	S05.9	9	Traumatismo	2
2	01/05/2021	H15.00	6	Escleritis	2
1	01/06/2021	T15.0	1	Cuerpo extraño	2
2	01/06/2021	S05.9	9	Traumatismo	2
2	01/06/2021	H16.0	1	Úlcera corneal	2
1	01/06/2021	H05.01	4	Celulitis orbitaria	2
2	01/06/2021	H16.9	1	Queratitis	2
2	01/06/2021	H0.41	1	Ojo seco	2
2	01/06/2021	H43.81	7	DVP	2
1	01/06/2021	H02	4	Alteración palpebral	2
2	01/06/2021	H11.3	0	Hiposfagma	2
1	01/06/2021	H01.00	4	Blefaritis	2
2	01/06/2021	H20	6	Uveitis	2
2	01/06/2021	H16.3	1	Abceso corneal	2
2	01/06/2021	H01.00	4	Blefaritis	2
1	01/06/2021	H17.8	1	Infiltrados corneales	2
1	01/06/2021	H53.10	11	Problema refractivo	2
2	01/06/2021	H16.0	1	Úlcera corneal	2
1	01/06/2021	H10.3	0	Conjuntivitis	2
2	01/06/2021	B00.1	4	Dermatitis herpética	2
1	01/06/2021	T15.0	1	Cuerpo extraño	2
2	01/06/2021	H16.9	1	Queratitis	2
2	01/06/2021	H16.0	1	Úlcera corneal	2
1	01/06/2021	H16.0	1	Úlcera corneal	2
2	01/06/2021	H11.3	0	Hiposfagma	2
1	01/06/2021	H11.3	0	Hiposfagma	2
1	01/06/2021	H10.81	0	Pingueculitis	2

2	01/06/2021	H16.9	1	Queratitis	2
1	01/07/2021	H16.9	1	Queratitis	2
2	01/07/2021	H43.81	7	DVP	2
2	01/07/2021	H10.3	0	Conjuntivitis	2
2	01/07/2021	H20	6	Uveitis	2
2	01/07/2021	H10.3	0	Conjuntivitis	2
2	01/07/2021	S05.9	9	Traumatismo	2
1	01/07/2021	H16.0	1	Úlcera corneal	2
1	01/07/2021	H01.00	4	Blefaritis	2
2	01/07/2021	H10.3	1	Conjuntivitis	2
2	01/07/2021	H33.30	8	Desgarro retiniano	2
2	01/07/2021	H16.9	1	Queratitis	2
1	01/07/2021	S05.9	9	Traumatismo	2
1	01/08/2021	H01.00	4	Blefaritis	2
1	01/08/2021	H16.0	1	Úlcera corneal	2
2	01/08/2021	H16.9	1	Queratitis	2
2	01/08/2021	H43.81	7	DVP	2
1	01/08/2021	H16.0	1	Úlcera corneal	2
2	01/08/2021	Alta por fuga	11	Alta por fuga	2
2	01/08/2021	H43.81	7	DVP	2
1	01/08/2021	H16.0	1	Úlcera corneal	2
2	01/08/2021	H16.0	1	Úlcera corneal	2
1	01/08/2021	H35.3	8	Maculopatía	2
2	01/08/2021	H16.0	1	Úlcera corneal	2
2	01/08/2021	H16.9	1	Queratitis	2
2	01/08/2021	H00.02	4	Orzuelo	2
2	01/08/2021	H16.9	1	Queratitis	2
2	01/08/2021	H16.9	1	Queratitis	2
2	01/08/2021	H33.0	8	Desprendimiento de retina	2
1	01/08/2021	H16.0	1	Úlcera corneal	2
1	01/09/2021	H20	6	Uveitis	2
2	01/09/2021	H16.0	1	Úlcera corneal	2
1	01/10/2021	H00.03	4	Celulitis preseptal	2
1	01/10/2021	T15.0	1	Cuerpo extraño	2
2	01/10/2021	H43.81	7	DVP	2
2	01/10/2021	H11.3	0	Hiposfagma	2
1	01/10/2021	S05.6	9	Perforación ocular	2
2	01/10/2021	H11.00	0	Pterigium	2
1	01/10/2021	H16.9	1	Queratitis	2
2	01/10/2021	S05.9	9	Traumatismo	2
1	01/10/2021	S05.9	9	Traumatismo	2
1	01/10/2021	H16.0	1	Úlcera corneal	2
1	01/10/2021	S05.9	9	Traumatismo	2
1	01/10/2021	H01.00	4	Blefaritis	2
1	01/10/2021	H10.3	0	Conjuntivitis	2
2	01/11/2021	H46	10	Neuritis óptica	2
1	01/11/2021	H26.9	2	Catarata	2
2	01/11/2021	H11.3	0	Hiposfagma	2
1	01/11/2021	H16.0	1	Úlcera corneal	2
2	01/11/2021	H33.0	8	Desprendimiento de retina	2
1	01/11/2021	H15.1	6	Epiescleritis	2
1	01/11/2021	H01.00	4	Blefaritis	2
2	01/11/2021	H20	6	Uveitis	2
1	01/11/2021	H01.00	4	Blefaritis	2
1	01/11/2021	H05.01	4	Celulitis orbitaria	2
1	01/11/2021	H04.32	5	Dacriocistitis aguda	2
2	01/11/2021	H16.9	1	Queratitis	2
1	01/11/2021	H15.1	6	Epiescleritis	2
1	01/11/2021	T15.0	1	Cuerpo extraño	2
2	01/11/2021	H20	6	Uveitis	2
1	01/11/2021	H46	10	Neuritis óptica	2
1	01/11/2021	H43.81	7	DVP	2
2	01/11/2021	S05.9	9	Traumatismo	2
1	01/12/2021	H34.82	8	Trombosis venosa	2
2	01/12/2021	H43.81	7	DVP	2
1	01/12/2021	S05.9	9	Traumatismo	2
2	01/12/2021	H16.0	1	Úlcera corneal	2
2	01/12/2021	S05.9	9	Traumatismo	2
1	01/12/2021	G43.9	10	Migraña	2
2	01/12/2021	H0.41	1	Ojo seco	2
2	01/12/2021	H11.3	0	Hiposfagma	2

1	01/12/2021	H16.0	1	Úlcera corneal	2
1	01/12/2021	H20	6	Uveitis	2
2	01/12/2021	H16.9	1	Queratitis	2
2	01/12/2021	H11.3	0	Hiposfagma	2
2	01/12/2021	H43.81	7	DVP	2
1	01/12/2021	H20	6	Uveitis	2
2	01/12/2021	H05.01	4	Celulitis orbitaria	2
1	1/13/21 10:05	B00.1	4	Dermatitis herpética	2
1	1/13/21 10:10	H16.9	1	Queratitis	2
2	1/13/21 11:28	H10.81	0	Pingueculitis	2
1	1/13/21 11:37	H16.0	1	Úlcera corneal	2
2	1/13/21 12:48	H17.8	1	Infiltrados corneales	2
2	1/13/21 13:54	H16.9	1	Queratitis	2
2	1/13/21 14:02	H10.3	0	Conjuntivitis	2
2	1/13/21 15:30	H43.81	7	DVP	2
1	1/13/21 15:39	H11.00	0	Pterigium	2
2	1/13/21 16:17	H16.0	1	Úlcera corneal	2
1	1/13/21 18:08	H02	4	Alteración palpebral	2
2	1/13/21 18:14	H43.81	7	DVP	2
1	1/13/21 18:45	Alta por fuga	11	Alta por fuga	2
2	1/13/21 18:54	H40.9	3	Glaucoma	2
1	1/14/21 0:47	H16.9	1	Queratitis	2
1	1/14/21 5:46	H16.9	1	Queratitis	2
2	1/14/21 8:25	G43.109	10	Aura	2
2	1/14/21 9:47	H20	6	Uveitis	2
2	1/14/21 10:54	H01.00	4	Blefaritis	2
1	1/14/21 11:21	H20	6	Uveitis	2
2	1/14/21 11:23	H43.1	7	Hemovítreo	2
2	1/14/21 12:00	H01.00	4	Blefaritis	2
1	1/14/21 12:31	H01.00	4	Blefaritis	2
1	1/14/21 13:00	S05.9	9	Traumatismo	2
1	1/14/21 13:36	H10.3	0	Conjuntivitis	2
2	1/14/21 14:59	H10.3	0	Conjuntivitis	2
2	1/14/21 15:56	S05.9	9	Traumatismo	2
2	1/14/21 16:02	H34.82	8	Trombosis venosa	2
1	1/14/21 17:17	H53.10	11	Problema refractivo	2
2	1/14/21 17:34	H00.19	4	Chalazión	2
2	1/14/21 18:11	G43.109	10	Aura	2
1	1/14/21 18:18	H35.3	8	Maculopatía	2
1	1/14/21 18:49	H01.00	4	Blefaritis	2
1	1/14/21 19:01	H10.3	0	Conjuntivitis	2
2	1/14/21 20:49	B00.1	4	Dermatitis herpética	2
1	1/14/21 20:55	B00.1	4	Dermatitis herpética	2
1	1/14/21 22:04	T15.0	1	Cuerpo extraño	2
1	1/15/21 0:58	H16.0	1	Úlcera corneal	2
2	1/15/21 8:49	H01.00	4	Blefaritis	2
2	1/15/21 10:13	H18.50	1	Distrofia corneal	2
1	1/15/21 10:24	H00.03	4	Celulitis preseptal	2
2	1/15/21 11:21	H16.9	1	Queratitis	2
1	1/15/21 11:22	H35.30	8	DMAE	2
1	1/15/21 12:16	H40.05	3	HTO	2
2	1/15/21 12:49	H43.81	7	DVP	2
2	1/15/21 12:56	H20	6	Uveitis	2
2	1/15/21 13:11	H10.3	0	Conjuntivitis	2
1	1/15/21 14:26	H02	4	Alteración palpebral	2
2	1/15/21 16:43	S05.9	9	Traumatismo	2
2	1/15/21 17:48	H27.8	2	OCP	2
2	1/15/21 18:13	H43.81	7	DVP	2
2	1/15/21 19:59	H16.0	1	Úlcera corneal	2
2	1/15/21 20:03	T26	9	Causticación	2
2	1/15/21 20:17	B00.1	4	Dermatitis herpética	2
1	1/15/21 22:55	B00.1	4	Dermatitis herpética	2
2	1/15/21 23:13	H16.0	1	Úlcera corneal	2
1	1/16/21 0:23	T15.0	1	Cuerpo extraño	2
2	1/16/21 1:01	H10.3	0	Conjuntivitis	2
1	1/16/21 6:37	H16.9	1	Queratitis	2
1	1/16/21 7:46	S05.9	9	Traumatismo	2
1	1/16/21 9:06	T15.0	1	Cuerpo extraño	2
1	1/16/21 10:07	H01.00	4	Blefaritis	2
1	1/16/21 11:13	H16.9	1	Queratitis	2
2	1/16/21 11:41	H10.3	0	Conjuntivitis	2

2	1/16/21 11:55	H10.3		0	Conjuntivitis	2
2	1/16/21 11:57	H01.00		4	Blefaritis	2
1	1/16/21 12:13	H10.3		0	Conjuntivitis	2
1	1/16/21 12:50	S05.9		9	Traumatismo	2
1	1/16/21 13:03	H16.0		1	Úlcera corneal	2
1	1/16/21 14:35	H16.0		1	Úlcera corneal	2
2	1/16/21 15:30	H16.9		1	Queratitis	2
1	1/16/21 16:02	H43.81		7	DVP	2
2	1/16/21 17:23	H01.00		4	Blefaritis	2
1	1/16/21 17:32	H16.0		1	Úlcera corneal	2
1	1/16/21 18:19	H20		6	Uveitis	2
1	1/16/21 18:57	B00.1		4	Dermatitis herpética	2
1	1/16/21 20:34	T15.0		1	Cuerpo extraño	2
2	1/16/21 21:04	H05.01		4	Celulitis orbitaria	2
2	1/16/21 21:27	H43.81		7	DVP	2
1	1/16/21 22:10	T15.0		1	Cuerpo extraño	2
1	1/16/21 23:28	H20		6	Uveitis	2
1	1/16/21 23:49	T15.0		1	Cuerpo extraño	2
1	1/17/21 2:48	H43.1		7	Hemovítreo	2
2	1/17/21 8:32	H02		4	Alteración palpebral	2
2	1/17/21 9:36	H16.0		1	Úlcera corneal	2
1	1/17/21 9:45	H16.0		1	Úlcera corneal	2
2	1/17/21 10:56	H43.1		7	Hemovítreo	2
2	1/17/21 11:22	H10.81		0	Pingueculitis	2
1	1/17/21 11:55	H02		4	Alteración palpebral	2
1	1/17/21 12:16	H16.9		1	Queratitis	2
1	1/17/21 12:34	H16.9		1	Queratitis	2
2	1/17/21 12:57	H16.0		1	Úlcera corneal	2
2	1/17/21 13:33	S05.9		9	Traumatismo	2
1	1/17/21 15:19	H20		6	Uveitis	2
1	1/17/21 16:43	H16.0		1	Úlcera corneal	2
1	1/17/21 16:51	H10.3		0	Conjuntivitis	2
1	1/17/21 17:49	S05.9		9	Traumatismo	2
1	1/17/21 18:47	H16.9		1	Queratitis	2
2	1/17/21 18:52	H0.41		1	Ojo seco	2
2	1/17/21 18:53	H00.19		4	Chalazión	2
2	1/17/21 19:09	H16.9		1	Queratitis	2
1	1/17/21 19:46	T15.0		1	Cuerpo extraño	2
1	1/17/21 20:20	S05.9		9	Traumatismo	2
1	1/17/21 21:02	H16.0		1	Úlcera corneal	2
2	1/17/21 22:04	S05.9		9	Traumatismo	2
1	1/17/21 22:29	T26		9	Causticación	2
1	1/17/21 23:18	H33.30		8	Desgarro retiniano	2
2	1/18/21 5:33	H16.0		1	Úlcera corneal	2
2	1/18/21 8:34	H00.02		4	Orzuelo	2
2	1/18/21 10:44	H18.20		1	Edema corneal	2
2	1/18/21 11:05	H04.32		5	Dacriocistitis aguda	2
1	1/18/21 11:53	H16.0		1	Úlcera corneal	2
1	1/18/21 12:18	H40.9		3	Glaucoma	2
2	1/18/21 13:19	H43.81		7	DVP	2
2	1/18/21 13:29	H11.3		0	Hiposfagma	2
1	1/18/21 14:29	H00.02		4	Orzuelo	2
1	1/18/21 15:45	H16.0		1	Úlcera corneal	2
1	1/18/21 16:19	H11.3		0	Hiposfagma	2
2	1/18/21 17:52	H16.9		1	Queratitis	2
1	1/18/21 17:59	H10.3		0	Conjuntivitis	2
2	1/18/21 18:03	H02		4	Alteración palpebral	2
1	1/18/21 18:14	H40.9		3	Glaucoma	2
2	1/18/21 18:24	H11.3		0	Hiposfagma	2
2	1/18/21 18:28	G43.9		10	Migraña	2
2	1/18/21 18:35	H16.9		1	Queratitis	2
2	1/18/21 18:58	G43.9		10	Migraña	2
2	1/18/21 20:38	H43.81		7	DVP	2
2	1/18/21 22:02	H16.0		1	Úlcera corneal	2
1	1/19/21 0:39	H16.9		1	Queratitis	2
1	1/19/21 2:54	H16.9		1	Queratitis	2
1	1/19/21 7:16	H43.81		7	DVP	2
1	1/19/21 10:29	H16.0		1	Úlcera corneal	2
2	1/19/21 11:22	H35.30		8	DMAE	2
1	1/19/21 11:31	H10.3		0	Conjuntivitis	2
1	1/19/21 11:48	H20		6	Uveitis	2

1	1/19/21 12:03	H43.81	7	DVP	2
2	1/19/21 12:46	H11.3	0	Hiposfagma	2
2	1/19/21 14:03	H10.3	0	Conjuntivitis	2
2	1/19/21 14:46	H10.3	0	Conjuntivitis	2
1	1/19/21 15:19	H33.30	8	Desgarro retiniano	2
2	1/19/21 15:38	H16.0	1	Úlcera corneal	2
1	1/19/21 15:49	T15.0	1	Cuerpo extraño	2
2	1/19/21 16:50	H16.0	1	Úlcera corneal	2
1	1/19/21 17:18	E11.319	8	Retinopatía diabética	2
2	1/19/21 17:21	H43.81	7	DVP	2
2	1/19/21 18:27	H00.02	4	Orzuelo	2
2	1/19/21 18:42	H01.00	4	Blefaritis	2
1	1/19/21 18:42	H16.9	1	Queratitis	2
2	1/19/21 20:18	H11.3	0	Hiposfagma	2
2	1/19/21 20:23	G43.9	10	Migraña	2
2	1/19/21 20:48	H00.02	4	Orzuelo	2
1	1/19/21 22:40	S05.9	9	Traumatismo	2
2	1/19/21 23:35	H17.8	1	Infiltrados corneales	2
1	1/19/21 23:51	H16.0	1	Úlcera corneal	2
2	1/20/21 2:07	H04.32	5	Dacriocistitis aguda	2
2	1/20/21 4:53	H16.0	1	Úlcera corneal	2
1	1/20/21 6:21	H16.9	1	Queratitis	2
1	1/20/21 7:11	S05.9	9	Traumatismo	2
2	1/20/21 8:00	H43.81	7	DVP	2
2	1/20/21 9:13	H43.81	7	DVP	2
2	1/20/21 10:12	H16.9	1	Queratitis	2
1	1/20/21 10:41	H16.0	1	Úlcera corneal	2
2	1/20/21 10:53	H40.05	3	HTO	2
1	1/20/21 11:19	H43.81	7	DVP	2
2	1/20/21 11:21	H10.3	0	Conjuntivitis	2
1	1/20/21 11:31	H16.0	1	Úlcera corneal	2
1	1/20/21 11:35	H16.0	1	Úlcera corneal	2
2	1/20/21 11:49	H18.20	1	Edema corneal	2
1	1/20/21 12:58	H26.9	2	Catarata	2
1	1/20/21 13:41	H02	4	Alteración palpebral	2
1	1/20/21 14:13	H16.0	1	Úlcera corneal	2
1	1/20/21 14:20	H16.0	1	Úlcera corneal	2
1	1/20/21 14:39	H00.03	4	Celulitis preseptal	2
1	1/20/21 15:51	H16.0	1	Úlcera corneal	2
1	1/20/21 15:56	H33.0	8	Desprendimiento de retina	2
1	1/20/21 16:28	H43.1	8	Hemovítreo	2
1	1/20/21 16:51	T15.0	1	Cuerpo extraño	2
2	1/20/21 17:07	H05.01	4	Celulitis orbitaria	2
1	1/20/21 17:45	H43.81	7	DVP	2
2	1/20/21 17:57	H05.01	4	Celulitis orbitaria	2
2	1/20/21 18:00	H43.81	7	DVP	2
1	1/20/21 18:01	H11.3	0	Hiposfagma	2
1	1/20/21 18:50	G43.9	10	Migraña	2
1	1/20/21 19:22	T15.0	1	Cuerpo extraño	2
2	1/20/21 21:34	H04.32	5	Dacriocistitis aguda	2
2	1/20/21 22:24	G43.9	10	Migraña	2
2	1/20/21 22:53	H16.0	1	Úlcera corneal	2
2	1/20/21 23:11	B58.0	8	Toxoplasmosis	2
2	1/21/21 8:08	H10.3	0	Conjuntivitis	2
2	1/21/21 9:02	H10.3	0	Conjuntivitis	2
2	1/21/21 9:41	B00.1	4	Dermatitis herpética	2
2	1/21/21 11:12	H16.9	1	Queratitis	2
2	1/21/21 11:13	H04.32	5	Dacriocistitis aguda	2
1	1/21/21 11:25	H43.81	7	DVP	2
2	1/21/21 12:21	H16.9	1	Queratitis	2
2	1/21/21 12:33	H20	6	Uveitis	2
2	1/21/21 13:38	H18.20	1	Edema corneal	2
2	1/21/21 14:32	G43.109	10	Aura	2
1	1/21/21 15:00	H04.32	5	Dacriocistitis aguda	2
2	1/21/21 16:14	H33.30	8	Desgarro retiniano	2
2	1/21/21 16:36	G43.9	10	Migraña	2
2	1/21/21 17:30	H43.81	7	DVP	2
2	1/21/21 17:43	H43.81	7	DVP	2
2	1/21/21 19:12	H53.10	11	Problema refractivo	2
2	1/22/21 0:03	H16.0	1	Úlcera corneal	2
2	1/22/21 3:51	S05.9	9	Traumatismo	2

1	1/22/21 10:30	H27.8	2	OCP	2
1	1/22/21 11:19	H18.20	1	Edema corneal	2
2	1/22/21 11:55	H43.81	7	DVP	2
1	1/22/21 13:42	H16.0	1	Úlcera corneal	2
2	1/22/21 14:03	H50.9	10	Estrabismo	2
2	1/22/21 14:18	H16.0	1	Úlcera corneal	2
2	1/22/21 14:48	H15.1	6	Epiescleritis	2
1	1/22/21 15:40	H00.02	4	Orzuelo	2
2	1/22/21 16:15	H16.0	1	Úlcera corneal	2
1	1/22/21 17:40	H16.0	1	Úlcera corneal	2
2	1/22/21 19:17	H16.0	1	Úlcera corneal	2
1	1/22/21 20:10	H16.0	1	Úlcera corneal	2
2	1/23/21 7:11	S05.30	0	Laceración conjuntival	2
2	1/23/21 8:18	H20	6	Uveitis	2
1	1/23/21 9:21	H11.3	0	Hiposfagma	2
1	1/23/21 10:30	H16.9	1	Queratitis	2
2	1/23/21 10:33	H26.9	2	Catarata	2
1	1/23/21 11:18	H11.3	0	Hiposfagma	2
2	1/23/21 11:46	H27.8	2	OCP	2
1	1/23/21 11:54	S05.9	9	Traumatismo	2
2	1/23/21 13:01	H26.9	2	Catarata	2
2	1/23/21 15:02	H11.3	0	Hiposfagma	2
1	1/23/21 15:11	S05.9	9	Traumatismo	2
2	1/23/21 15:44	H20	6	Uveitis	2
1	1/23/21 16:13	H16.9	1	Queratitis	2
2	1/23/21 16:25	H16.9	1	Queratitis	2
2	1/23/21 16:26	H15.00	6	Escleritis	2
1	1/23/21 16:35	H16.0	1	Úlcera corneal	2
1	1/23/21 17:10	B00.1	4	Dermatitis herpética	2
2	1/23/21 17:27	H16.9	1	Queratitis	2
1	1/23/21 17:58	H16.9	1	Queratitis	2
2	1/23/21 18:36	H10.81	0	Pingueculitis	2
1	1/23/21 19:50	H16.0	1	Úlcera corneal	2
1	1/23/21 22:32	H05.01	4	Celulitis orbitaria	2
2	1/23/21 22:54	H16.9	1	Queratitis	2
1	1/24/21 0:31	H10.3	0	Conjuntivitis	2
1	1/24/21 5:38	S05.9	9	Traumatismo	2
1	1/24/21 10:15	H16.9	1	Queratitis	2
2	1/24/21 10:19	H43.1	7	Hemovítreo	2
2	1/24/21 10:58	S05.9	9	Traumatismo	2
1	1/24/21 11:14	H16.9	1	Queratitis	2
2	1/24/21 11:21	H16.9	1	Queratitis	2
1	1/24/21 12:25	H16.9	1	Queratitis	2
2	1/24/21 14:54	H15.1	6	Epiescleritis	2
2	1/24/21 15:05	H16.0	1	Úlcera corneal	2
1	1/24/21 16:43	H16.9	1	Queratitis	2
2	1/24/21 17:03	G43.9	10	Migraña	2
2	1/24/21 18:10	H16.0	1	Úlcera corneal	2
2	1/24/21 18:11	H0.41	1	Ojo seco	2
2	1/24/21 19:36	H50.9	10	Estrabismo	2
2	1/24/21 19:55	H01.00	4	Blefaritis	2
1	1/24/21 22:14	H16.9	1	Queratitis	2
2	1/25/21 8:09	H43.1	7	Hemovítreo	2
2	1/25/21 9:14	H10.3	0	Conjuntivitis	2
2	1/25/21 10:25	H01.00	4	Blefaritis	2
2	1/25/21 10:27	H43.1	7	Hemovítreo	2
2	1/25/21 10:34	H33.30	8	Desgarro retiniano	2
2	1/25/21 10:35	H43.81	7	DVP	2
2	1/25/21 10:51	H10.3	0	Conjuntivitis	2
2	1/25/21 11:04	H20	6	Uveitis	2
2	1/25/21 11:53	T15.0	1	Cuerpo extraño	2
1	1/25/21 11:57	H10.3	0	Conjuntivitis	2
2	1/25/21 13:48	H17.8	1	Infiltrados corneales	2
2	1/25/21 14:04	H43.81	7	DVP	2
2	1/25/21 14:33	H15.00	6	Escleritis	2
1	1/25/21 15:18	H43.81	7	DVP	2
2	1/25/21 15:31	H16.0	1	Úlcera corneal	2
2	1/25/21 16:57	H02	4	Alteración palpebral	2
1	1/25/21 16:58	H11.3	0	Hiposfagma	2
2	1/25/21 17:34	H16.9	1	Queratitis	2
1	1/25/21 18:31	S05.9	9	Traumatismo	2

1	1/25/21 18:34	H10.3		0	Conjuntivitis	2
2	1/25/21 19:56	T26		9	Causticación	2
1	1/25/21 20:23	T15.0		1	Cuerpo extraño	2
2	1/25/21 20:27	S05.9		9	Traumatismo	2
1	1/25/21 20:38	H53.10		11	Problema refractivo	2
2	1/26/21 9:41	H43.81		7	DVP	2
2	1/26/21 11:26	H16.9		1	Queratitis	2
2	1/26/21 11:42	H20		6	Uveitis	2
2	1/26/21 12:34	H16.0		1	Úlcera corneal	2
2	1/26/21 12:47	H43.81		7	DVP	2
1	1/26/21 13:19	H16.0		1	Úlcera corneal	2
2	1/26/21 13:24	H01.00		4	Blefaritis	2
1	1/26/21 13:30	H43.81		7	DVP	2
2	1/26/21 14:15	H16.9		1	Queratitis	2
1	1/26/21 14:24	H02		4	Alteración palpebral	2
2	1/26/21 16:47	B00.1		4	Dermatitis herpética	2
2	1/26/21 17:41	H16.0		1	Úlcera corneal	2
2	1/26/21 19:35	H20		6	Uveitis	2
2	1/26/21 20:19	H0.41		1	Ojo seco	2
2	1/26/21 21:04	H16.0		1	Úlcera corneal	2
2	1/26/21 21:26	H10.3		0	Conjuntivitis	2
1	1/26/21 21:48	T15.0		1	Cuerpo extraño	2
2	1/27/21 9:55	H00.02		4	Orzuelo	2
2	1/27/21 10:22	H33.0		8	Desprendimiento de retina	2
2	1/27/21 10:26	B00.1		4	Dermatitis herpética	2
2	1/27/21 11:20	E11.319		8	Retinopatía diabética	2
1	1/27/21 12:08	T15.0		1	Cuerpo extraño	2
2	1/27/21 12:29	H15.1		6	Epiescleritis	2
2	1/27/21 12:39	H43.81		7	DVP	2
1	1/27/21 13:07	T15.0		1	Cuerpo extraño	2
1	1/27/21 13:15	T15.0		1	Cuerpo extraño	2
2	1/27/21 13:17	H00.02		4	Orzuelo	2
2	1/27/21 13:29	H16.9		1	Queratitis	2
2	1/27/21 13:52	H02		4	Alteración palpebral	2
2	1/27/21 14:50	H10.81		0	Pingueculitis	2
2	1/27/21 15:35	H16.0		1	Úlcera corneal	2
2	1/27/21 15:40	H35.30		8	DMAE	2
2	1/27/21 15:47	H16.9		1	Queratitis	2
2	1/27/21 16:25	H10.3		0	Conjuntivitis	2
2	1/27/21 16:45	S05.9		9	Traumatismo	2
2	1/27/21 16:47	H11.3		0	Hiposfagma	2
2	1/27/21 16:59	H43.81		7	DVP	2
1	1/27/21 17:01	T26		9	Causticación	2
1	1/27/21 17:32	H18.50		1	Distrofia corneal	2
2	1/27/21 18:23	S05.30		9	Laceración conjuntival	2
1	1/27/21 18:47	T15.0		1	Cuerpo extraño	2
1	1/27/21 19:53	T15.0		1	Cuerpo extraño	2
2	1/27/21 20:33	H10.3		0	Conjuntivitis	2
2	1/27/21 21:08	H10.3		0	Conjuntivitis	2
1	1/27/21 22:57	H15.1		6	Epiescleritis	2
2	1/28/21 7:29	H20		6	Uveitis	2
2	1/28/21 8:38	G43.9		10	Migraña	2
2	1/28/21 9:26	H00.03		4	Celulitis preseptal	2
2	1/28/21 9:56	H01.00		4	Blefaritis	2
2	1/28/21 10:01	H16.0		1	Úlcera corneal	2
2	1/28/21 10:46	H16.9		1	Queratitis	2
1	1/28/21 11:18	H10.3		0	Conjuntivitis	2
2	1/28/21 11:37	H20		6	Uveitis	2
2	1/28/21 12:04	H01.00		4	Blefaritis	2
1	1/28/21 12:37	T15.0		1	Cuerpo extraño	2
2	1/28/21 13:27	H16.3		1	Abceso corneal	2
2	1/28/21 13:42	S05.9		9	Traumatismo	2
2	1/28/21 14:01	H16.0		1	Úlcera corneal	2
1	1/28/21 14:47	H16.9		1	Queratitis	2
1	1/28/21 14:48	H44.009		8	Endoftalmitis	2
2	1/28/21 15:12	H43.81		7	DVP	2
1	1/28/21 15:21	H16.0		1	Úlcera corneal	2
1	1/28/21 17:03	S05.9		9	Traumatismo	2
1	1/28/21 17:26	S05.6		9	Perforación ocular	2
1	1/28/21 17:46	H10.81		0	Pingueculitis	2
1	1/28/21 18:48	H00.02		4	Orzuelo	2

2	1/28/21 18:49	H43.81	7	DVP	2
1	1/28/21 20:02	T15.0	1	Cuerpo extraño	2
1	1/28/21 20:40	T15.0	1	Cuerpo extraño	2
1	1/28/21 21:51	T15.0	1	Cuerpo extraño	2
2	1/28/21 22:05	H10.3	0	Conjuntivitis	2
2	1/28/21 22:29	H11.3	0	Hiposfagma	2
2	1/28/21 23:47	H16.9	1	Queratitis	2
2	1/29/21 8:44	H35.3	8	Maculopatía	2
2	1/29/21 8:46	H43.81	7	DVP	2
1	1/29/21 9:25	H16.0	1	Úlcera corneal	2
2	1/29/21 9:37	H10.3	0	Conjuntivitis	2
1	1/29/21 9:50	H10.3	0	Conjuntivitis	2
1	1/29/21 9:54	H35.30	8	DMAE	2
2	1/29/21 9:57	H16.0	1	Úlcera corneal	2
1	1/29/21 11:16	H16.9	1	Queratitis	2
1	1/29/21 11:17	H10.81	0	Pingueculitis	2
2	1/29/21 11:52	H43.81	7	DVP	2
1	1/29/21 12:09	H40.05	3	HTO	2
2	1/29/21 12:32	H11.3	0	Hiposfagma	2
1	1/29/21 12:39	H16.9	1	Queratitis	2
2	1/29/21 12:55	Z97.0	4	Protesis ocular	2
2	1/29/21 13:02	H05.01	4	Celulitis orbitaria	2
1	1/29/21 14:04	H27.8	2	OCP	2
2	1/29/21 14:28	H16.0	1	Úlcera corneal	2
2	1/29/21 15:23	H10.3	0	Conjuntivitis	2
1	1/29/21 15:54	H16.9	1	Queratitis	2
2	1/29/21 16:02	H10.3	0	Conjuntivitis	2
1	1/29/21 16:16	T15.0	1	Cuerpo extraño	2
2	1/29/21 17:25	H10.3	0	Conjuntivitis	2
2	1/29/21 17:31	H40.9	3	Glaucoma	2
2	1/29/21 17:39	H16.9	1	Queratitis	2
1	1/29/21 17:43	T15.0	1	Cuerpo extraño	2
1	1/29/21 17:49	H11.3	0	Hiposfagma	2
2	1/29/21 17:55	H43.81	7	DVP	2
2	1/29/21 18:41	S05.9	9	Traumatismo	2
2	1/29/21 18:46	T26	9	Causticación	2
2	1/29/21 18:58	T15.0	1	Cuerpo extraño	2
1	1/29/21 19:51	H11.3	0	Hiposfagma	2
1	1/29/21 21:04	H16.0	1	Úlcera corneal	2
2	1/29/21 22:40	H43.81	7	DVP	2
1	1/30/21 8:30	H10.3	0	Conjuntivitis	2
1	1/30/21 10:28	T15.0	1	Cuerpo extraño	2
1	1/30/21 10:28	T15.0	1	Cuerpo extraño	2
1	1/30/21 10:30	H00.02	4	Orzuelo	2
2	1/30/21 10:43	H16.9	1	Queratitis	2
2	1/30/21 10:51	H10.81	0	Pingueculitis	2
2	1/30/21 11:02	H16.3	1	Abceso corneal	2
1	1/30/21 12:33	H16.0	1	Úlcera corneal	2
2	1/30/21 12:33	H20	6	Uveitis	2
1	1/30/21 13:20	H00.02	4	Orzuelo	2
2	1/30/21 13:21	H01.00	4	Blefaritis	2
1	1/30/21 13:45	S05.9	9	Traumatismo	2
2	1/30/21 14:48	H00.02	4	Orzuelo	2
2	1/30/21 15:11	H16.9	1	Queratitis	2
1	1/30/21 15:20	S05.30	9	Laceración conjuntival	2
2	1/30/21 15:27	H16.0	1	Úlcera corneal	2
1	1/30/21 16:00	H44.009	8	Endoftalmitis	2
1	1/30/21 16:08	H16.9	1	Queratitis	2
2	1/30/21 16:17	H11.3	0	Hiposfagma	2
2	1/30/21 16:29	S05.9	9	Traumatismo	2
2	1/30/21 16:53	H17.8	1	Infiltrados corneales	2
2	1/30/21 17:17	H20	6	Uveitis	2
1	1/30/21 17:51	H10.3	0	Conjuntivitis	2
2	1/30/21 18:04	H40.05	3	HTO	2
2	1/30/21 18:24	H16.0	1	Úlcera corneal	2
2	1/30/21 18:51	H16.9	1	Queratitis	2
2	1/30/21 20:16	H10.3	0	Conjuntivitis	2
1	1/30/21 20:53	H16.9	1	Queratitis	2
1	1/30/21 22:23	H20	6	Uveitis	2
1	1/31/21 0:16	H40.9	3	Glaucoma	2
1	1/31/21 6:05	T15.0	1	Cuerpo extraño	2

2	1/31/21 7:28	T15.0		1	Cuerpo extraño	2
1	1/31/21 9:56	H05.01		4	Celulitis orbitaria	2
1	1/31/21 11:11	H16.0		1	Úlcera corneal	2
2	1/31/21 12:21	H10.3		0	Conjuntivitis	2
2	1/31/21 12:22	H16.0		1	Úlcera corneal	2
1	1/31/21 12:37	H11.82		0	Conjuntivocalasia	2
1	1/31/21 12:50	H16.9		1	Queratitis	2
2	1/31/21 13:09	H11.3		0	Hiposfagma	2
2	1/31/21 13:36	H20		6	Uveitis	2
1	1/31/21 14:12	T15.0		1	Cuerpo extraño	2
1	1/31/21 15:59	S05.9		9	Traumatismo	2
2	1/31/21 16:25	H16.9		1	Queratitis	2
1	1/31/21 17:45	H16.0		1	Úlcera corneal	2
1	1/31/21 20:35	H34.82		8	Trombosis venosa	2
1	1/31/21 23:02	T15.0		1	Cuerpo extraño	2
2	02/01/2021	H16.0		1	Úlcera corneal	2
1	02/01/2021	H20		6	Uveitis	2
2	02/01/2021	S05.9		9	Traumatismo	2
1	02/01/2021	H01.00		4	Blefaritis	2
2	02/01/2021	H16.0		1	Úlcera corneal	2
2	02/01/2021	H16.0		1	Úlcera corneal	2
2	02/01/2021	H10.3		0	Conjuntivitis	2
2	02/01/2021	H27.8		2	OCP	2
2	02/01/2021	H43.81		7	DVP	2
1	02/01/2021	T15.0		1	Cuerpo extraño	2
1	02/01/2021	H16.9		1	Queratitis	2
1	02/01/2021	H20.9		6	Uveítis	2
2	02/01/2021	H11.3		0	Hiposfagma	2
1	02/01/2021	H16.9		1	Queratitis	2
2	02/01/2021	H11.3		0	Hiposfagma	2
2	02/01/2021	H17.8		1	Infiltrados corneales	2
2	02/01/2021	H11.3		0	Hiposfagma	2
2	02/01/2021	H16.9		1	Queratitis	2
1	02/01/2021	G43.9		10	Migraña	2
2	02/01/2021	H10.3		0	Conjuntivitis	2
2	02/01/2021	H00.02		4	Orzuelo	2
2	02/01/2021	H16.9		1	Queratitis	2
2	02/01/2021	Z98.4		2	Post op catarata	2
1	02/01/2021	T15.0		1	Cuerpo extraño	2
2	02/01/2021	H10.3		0	Conjuntivitis	2
1	02/01/2021	H16.0		1	Úlcera corneal	2
1	02/01/2021	H16.0		1	Úlcera corneal	2
1	02/01/2021	T15.0		1	Cuerpo extraño	2
1	02/01/2021	H16.0		1	Úlcera corneal	2
2	02/01/2021	H16.0		1	Úlcera corneal	2
2	02/01/2021	H11.3		0	Hiposfagma	2
1	02/02/2021	H16.9		1	Queratitis	2
1	02/02/2021	H33.0		8	Desprendimiento de retina	2
1	02/02/2021	T15.0		1	Cuerpo extraño	2
2	02/02/2021	H16.0		1	Úlcera corneal	2
1	02/02/2021	T15.0		1	Cuerpo extraño	2
1	02/02/2021	H02		4	Alteración palpebral	2
2	02/02/2021	H20		6	Uveitis	2
2	02/02/2021	H16.0		1	Úlcera corneal	2
2	02/02/2021	H01.00		4	Blefaritis	2
2	02/02/2021	Alta por fuga		11	Alta por fuga	2
2	02/02/2021	H16.9		1	Queratitis	2
1	02/02/2021	H26.9		2	Catarata	2
1	02/02/2021	H40.9		3	Glaucoma	2
1	02/02/2021	H16.0		1	Úlcera corneal	2
2	02/02/2021	H10.3		0	Conjuntivitis	2
1	02/02/2021	H43.81		7	DVP	2
2	02/02/2021	H05.01		4	Celulitis orbitaria	2
1	02/02/2021	H18.50		1	Distrofia corneal	2
2	02/02/2021	H53.9		11	Alteraciones visuales	2
2	02/02/2021	H00.02		4	Orzuelo	2
2	02/02/2021	H00.02		4	Orzuelo	2
2	02/02/2021	H16.9		1	Queratitis	2
1	02/02/2021	H16.0		1	Úlcera corneal	2
1	02/02/2021	H16.0		1	Úlcera corneal	2
2	02/02/2021	B00.1		4	Dermatitis herpética	2

1	02/02/2021	T15.0		1	Cuerpo extraño	2
1	02/03/2021	H16.0		1	Úlcera corneal	2
1	02/03/2021	T15.0		1	Cuerpo extraño	2
2	02/03/2021	H20		6	Uveitis	2
1	02/03/2021	H35.3		8	Maculopatía	2
2	02/03/2021	H43.81		7	DVP	2
2	02/03/2021	H0.41		1	Ojo seco	2
2	02/03/2021	H40.9		3	Glaucoma	2
2	02/03/2021	H01.00		4	Blefaritis	2
2	02/03/2021	H01.00		4	Blefaritis	2
2	02/03/2021	H02		4	Alteración palpebral	2
2	02/03/2021	H16.9		1	Queratitis	2
2	02/03/2021	H00.02		4	Orzuelo	2
1	02/03/2021	H43.81		7	DVP	2
1	02/03/2021	S05.9		9	Traumatismo	2
2	02/03/2021	G43.9		10	Migraña	2
2	02/03/2021	H01.00		4	Blefaritis	2
2	02/03/2021	H16.0		1	Úlcera corneal	2
1	02/03/2021	H16.9		1	Queratitis	2
1	02/03/2021	H16.9		1	Queratitis	2
1	02/03/2021	B00.1		4	Dermatitis herpética	2
1	02/03/2021	H16.0		1	Úlcera corneal	2
2	02/03/2021	H43.81		7	DVP	2
2	02/03/2021	H16.0		1	Úlcera corneal	2
2	02/03/2021	Alta por fuga		11	Alta por fuga	2
2	02/03/2021	H02		4	Alteración palpebral	2
1	02/04/2021	H10.3		0	Conjuntivitis	2
1	02/04/2021	T26		9	Causticación	2
1	02/04/2021	H10.3		0	Conjuntivitis	2
1	02/04/2021	H10.3		0	Conjuntivitis	2
1	02/04/2021	H10.3		0	Conjuntivitis	2
2	02/04/2021	H16.9		1	Queratitis	2
2	02/04/2021	H00.02		4	Orzuelo	2
2	02/04/2021	H43.81		7	DVP	2
2	02/04/2021	H01.00		4	Blefaritis	2
1	02/04/2021	H01.00		4	Blefaritis	2
1	02/04/2021	H33.0		8	Desprendimiento de retina	2
2	02/04/2021	H0.41		1	Ojo seco	2
2	02/04/2021	H11.3		0	Hiposfagma	2
1	02/04/2021	H16.9		1	Queratitis	2
1	02/04/2021	H16.9		1	Queratitis	2
2	02/04/2021	H43.81		7	DVP	2
2	02/04/2021	H16.9		1	Queratitis	2
2	02/04/2021	B00.1		4	Dermatitis herpética	2
1	02/04/2021	H11.3		0	Hiposfagma	2
1	02/04/2021	H16.9		1	Queratitis	2
2	02/04/2021	H43.81		7	DVP	2
2	02/04/2021	H0.41		1	Ojo seco	2
2	02/04/2021	G43.9		10	Migraña	2
2	02/04/2021	H15.00		6	Escleritis	2
1	02/04/2021	H27.10		2	Luxación LIO	2
1	02/04/2021	S05.9		9	Traumatismo	2
1	02/04/2021	T15.0		1	Cuerpo extraño	2
2	02/04/2021	H10.3		0	Conjuntivitis	2
2	02/04/2021	H16.9		1	Queratitis	2
1	02/05/2021	H16.9		1	Queratitis	2
2	02/05/2021	H16.9		1	Queratitis	2
1	02/05/2021	H16.9		1	Queratitis	2
2	02/05/2021	H16.0		1	Úlcera corneal	2
2	02/05/2021	H00.03		4	Celulitis preseptal	2
1	02/05/2021	H10.3		0	Conjuntivitis	2
2	02/05/2021	H18.20		1	Edema corneal	2
1	02/05/2021	H43.81		7	DVP	2
2	02/05/2021	G43.9		10	Migraña	2
2	02/05/2021	H33.0		8	Desprendimiento de retina	2
2	02/05/2021	S05.00		9	Abrasión conjuntival	2
2	02/05/2021	S05.9		9	Traumatismo	2
2	02/05/2021	H10.81		0	Pingueculitis	2
2	02/05/2021	H16.0		1	Úlcera corneal	2
1	02/05/2021	H27.10		2	Luxación LIO	2
1	02/05/2021	S05.9		9	Traumatismo	2

1	02/05/2021	H16.0	1	Úlcera corneal	2
2	02/05/2021	H43.81	7	DVP	2
2	02/05/2021	Alta por fuga	11	Alta por fuga	2
1	02/05/2021	H40.9	3	Glaucoma	2
2	02/05/2021	H16.0	1	Úlcera corneal	2
2	02/05/2021	H05.01	4	Celulitis orbitaria	2
2	02/05/2021	H16.0	1	Úlcera corneal	2
1	02/05/2021	H20	6	Uveitis	2
2	02/05/2021	H04.32	5	Dacriocistitis aguda	2
1	02/06/2021	T15.0	1	Cuerpo extraño	2
1	02/06/2021	S05.9	9	Traumatismo	2
2	02/06/2021	H01.00	4	Blefaritis	2
1	02/06/2021	H11.00	0	Pterigium	2
1	02/06/2021	H33.0	8	Desprendimiento de retina	2
1	02/06/2021	H16.9	1	Queratitis	2
2	02/06/2021	G43.9	10	Migraña	2
2	02/06/2021	H18.50	1	Distrofia corneal	2
2	02/06/2021	H43.81	7	DVP	2
1	02/06/2021	H10.81	0	Pingueculitis	2
2	02/06/2021	H00.02	4	Orzuelo	2
1	02/06/2021	H43.81	7	DVP	2
1	02/06/2021	H16.9	1	Queratitis	2
2	02/06/2021	H05.01	4	Celulitis orbitaria	2
2	02/06/2021	H16.0	1	Úlcera corneal	2
1	02/06/2021	H16.9	1	Queratitis	2
2	02/06/2021	H43.81	7	DVP	2
1	02/06/2021	H00.03	4	Celulitis preseptal	2
2	02/06/2021	H43.81	7	DVP	2
1	02/06/2021	H10.3	0	Conjuntivitis	2
1	02/06/2021	Alta por fuga	11	Alta por fuga	2
1	02/06/2021	H00.02	4	Orzuelo	2
2	02/06/2021	H16.0	1	Úlcera corneal	2
2	02/06/2021	H16.9	1	Queratitis	2
1	02/06/2021	H10.81	0	Pingueculitis	2
2	02/06/2021	H11.3	0	Hiposfagma	2
1	02/06/2021	S05.9	9	Traumatismo	2
1	02/06/2021	T15.0	1	Cuerpo extraño	2
1	02/07/2021	S05.9	9	Traumatismo	2
1	02/07/2021	T15.0	1	Cuerpo extraño	2
2	02/07/2021	H0.41	1	Ojo seco	2
2	02/07/2021	S05.9	9	Traumatismo	2
1	02/07/2021	H20	6	Uveitis	2
2	02/07/2021	H16.0	1	Úlcera corneal	2
1	02/07/2021	T15.0	1	Cuerpo extraño	2
2	02/07/2021	B00.1	4	Dermatitis herpética	2
1	02/07/2021	H16.0	1	Úlcera corneal	2
1	02/07/2021	H10.3	0	Conjuntivitis	2
1	02/07/2021	H10.3	0	Conjuntivitis	2
1	02/07/2021	G43.9	10	Migraña	2
2	02/07/2021	Z98.4	2	Post op catarata	2
1	02/07/2021	H11.3	0	Hiposfagma	2
2	02/07/2021	H16.9	1	Queratitis	2
1	02/07/2021	H40.9	3	Glaucoma	2
1	02/07/2021	S05.9	9	Traumatismo	2
2	02/08/2021	H11.3	0	Hiposfagma	2
2	02/08/2021	S05.9	9	Traumatismo	2
2	02/08/2021	H10.3	0	Conjuntivitis	2
2	02/08/2021	H43.81	7	DVP	2
2	02/08/2021	H00.19	4	Chalazión	2
2	02/08/2021	H40.05	3	HTO	2
1	02/08/2021	H10.3	0	Conjuntivitis	2
2	02/08/2021	H10.3	0	Conjuntivitis	2
1	02/08/2021	H20	6	Uveitis	2
1	02/08/2021	G43.9	10	Migraña	2
2	02/08/2021	H43.1	7	Hemovítreo	2
2	02/08/2021	H59.88	8	Post op retina	2
1	02/08/2021	B00.1	4	Dermatitis herpética	2
2	02/08/2021	H43.81	7	DVP	2
2	02/08/2021	H43.81	7	DVP	2
1	02/08/2021	H16.0	1	Úlcera corneal	2
1	02/08/2021	H20	6	Uveitis	2

1	02/08/2021	H16.9	1	Queratitis	2
1	02/08/2021	H01.00	4	Blefaritis	2
1	02/08/2021	H16.9	1	Queratitis	2
1	02/09/2021	H01.00	4	Blefaritis	2
1	02/09/2021	B00.1	4	Dermatitis herpética	2
2	02/09/2021	H33.30	8	Desgarro retiniano	2
2	02/09/2021	H00.02	4	Orzuelo	2
1	02/09/2021	H47.10	10	Papiledema	2
1	02/09/2021	H0.41	1	Ojo seco	2
2	02/09/2021	H43.81	7	DVP	2
1	02/09/2021	H43.81	7	DVP	2
2	02/09/2021	H01.00	4	Blefaritis	2
1	02/09/2021	H35.30	8	DMAE	2
2	02/09/2021	H16.0	1	Úlcera corneal	2
1	02/09/2021	H02	4	Alteración palpebral	2
2	02/09/2021	H43.81	7	DVP	2
1	02/09/2021	H10.3	0	Conjuntivitis	2
2	02/09/2021	H43.81	7	DVP	2
2	02/09/2021	H15.00	6	Escleritis	2
2	02/09/2021	H40.9	3	Glaucoma	2
2	02/09/2021	H11.3	0	Hiposfagma	2
1	02/09/2021	H16.9	1	Queratitis	2
1	02/09/2021	H20	6	Uveitis	2
1	02/09/2021	H43.81	7	DVP	2
1	02/09/2021	H16.9	1	Queratitis	2
1	02/09/2021	H10.3	0	Conjuntivitis	2
1	02/09/2021	T15.0	1	Cuerpo extraño	2
1	02/09/2021	S05.9	9	Traumatismo	2
2	02/10/2021	S05.9	9	Traumatismo	2
1	02/10/2021	H10.3	0	Conjuntivitis	2
2	02/10/2021	H33.0	8	Desprendimiento de retina	2
2	02/10/2021	H35.3	8	Maculopatía	2
2	02/10/2021	H05.01	4	Celulitis orbitaria	2
1	02/10/2021	H16.9	1	Queratitis	2
2	02/10/2021	H10.3	0	Conjuntivitis	2
1	02/10/2021	H20	6	Uveitis	2
1	02/10/2021	H33.0	8	Desprendimiento de retina	2
2	02/10/2021	H20	6	Uveitis	2
2	02/10/2021	H00.02	4	Orzuelo	2
2	02/10/2021	H53.9	11	Alteraciones visuales	2
2	02/10/2021	G43.9	10	Migraña	2
1	02/10/2021	H43.81	7	DVP	2
1	02/10/2021	H30.9	6	Uveitis posterior	2
1	02/10/2021	H16.0	1	Úlcera corneal	2
2	02/10/2021	H43.81	7	DVP	2
2	02/10/2021	H16.0	1	Úlcera corneal	2
1	02/10/2021	H01.00	4	Blefaritis	2
1	02/10/2021	H11.3	0	Hiposfagma	2
1	02/10/2021	H20	6	Uveitis	2
2	02/10/2021	H43.81	7	DVP	2
1	02/10/2021	H16.9	1	Queratitis	2
1	02/10/2021	H16.0	1	Úlcera corneal	2
2	02/10/2021	H34.82	8	Trombosis venosa	2
1	02/10/2021	H16.0	1	Úlcera corneal	2
2	02/10/2021	H50.9	10	Estrabismo	2
2	02/10/2021	H01.00	4	Blefaritis	2
1	02/10/2021	T15.0	1	Cuerpo extraño	2
2	02/10/2021	H16.9	1	Queratitis	2
2	02/10/2021	H20	6	Uveitis	2
1	02/10/2021	H16.9	1	Queratitis	2
1	02/10/2021	Alta por fuga	11	Alta por fuga	2
1	02/10/2021	H18.20	1	Edema corneal	2
1	02/10/2021	H16.9	1	Queratitis	2
1	02/10/2021	T15.0	1	Cuerpo extraño	2
2	02/11/2021	S05.9	9	Traumatismo	2
1	02/11/2021	H16.9	1	Queratitis	2
1	02/11/2021	H16.9	1	Queratitis	2
1	02/11/2021	H20	6	Uveitis	2
2	02/11/2021	H0.41	1	Ojo seco	2
2	02/11/2021	H00.02	4	Orzuelo	2
1	02/11/2021	H16.9	1	Queratitis	2

1	02/11/2021	S05.9		9	Traumatismo	2
2	02/11/2021	H0.41		1	Ojo seco	2
2	02/11/2021	H10.3		0	Conjuntivitis	2
2	02/11/2021	H15.1		6	Epiescleritis	2
1	02/11/2021	T15.0		1	Cuerpo extraño	2
1	02/11/2021	H43.81		7	DVP	2
1	02/11/2021	T15.0		1	Cuerpo extraño	2
1	02/11/2021	H16.0		1	Úlcera corneal	2
2	02/11/2021	H16.9		1	Queratitis	2
2	02/11/2021	G43.9		10	Migraña	2
2	02/11/2021	H33.0		8	Desprendimiento de retina	2
2	02/11/2021	H10.3		0	Conjuntivitis	2
1	02/11/2021	B00.1		4	Dermatitis herpética	2
2	02/11/2021	S05.9		9	Traumatismo	2
2	02/11/2021	H00.02		4	Orzuelo	2
1	02/11/2021	H33.30		8	Desgarro retiniano	2
1	02/11/2021	G43.9		10	Migraña	2
1	02/11/2021	H16.0		1	Úlcera corneal	2
2	02/11/2021	H11.3		0	Hiposfagma	2
1	02/11/2021	H33.30		8	Desgarro retiniano	2
2	02/12/2021	H16.0		1	Úlcera corneal	2
1	02/12/2021	H16.9		1	Queratitis	2
1	02/12/2021	H16.0		1	Úlcera corneal	2
2	02/12/2021	H00.03		4	Celulitis preseptal	2
2	02/12/2021	H16.0		1	Úlcera corneal	2
1	02/12/2021	H05.01		4	Celulitis orbitaria	2
1	02/12/2021	H43.81		7	DVP	2
2	02/12/2021	H0.41		1	Ojo seco	2
1	02/12/2021	H16.9		1	Queratitis	2
2	02/12/2021	H00.03		4	Celulitis preseptal	2
2	02/12/2021	H11.3		0	Hiposfagma	2
2	02/12/2021	Alta por fuga		11	Alta por fuga	2
1	02/12/2021	H04.32		5	Dacriocistitis aguda	2
1	02/12/2021	T15.0		1	Cuerpo extraño	2
1	02/12/2021	H10.3		0	Conjuntivitis	2
2	02/12/2021	H16.0		1	Úlcera corneal	2
1	02/12/2021	H16.0		1	Úlcera corneal	2
1	02/12/2021	H10.3		0	Conjuntivitis	2
1	02/12/2021	H43.81		7	DVP	2
2	02/12/2021	H16.0		1	Úlcera corneal	2
1	02/12/2021	H59.3		4	Postop via lagrimal	2
1	02/12/2021	Alta por fuga		11	Alta por fuga	2
2	02/12/2021	H40.9		3	Glaucoma	2
2	02/12/2021	H17.8		1	Infiltrados corneales	2
1	02/12/2021	H00.02		4	Orzuelo	2
1	02/12/2021	H16.9		1	Queratitis	2
1	02/12/2021	H16.0		1	Úlcera corneal	2
1	02/12/2021	T15.0		1	Cuerpo extraño	2
2	02/12/2021	H00.02		4	Orzuelo	2
2	02/12/2021	H01.00		4	Blefaritis	2
1	02/12/2021	H20		6	Uveitis	2
2	2/13/21 8:27	H10.3		0	Conjuntivitis	2
2	2/13/21 9:18	H05.01		4	Celulitis orbitaria	2
1	2/13/21 9:27	H20		6	Uveitis	2
2	2/13/21 10:34	H43.81		7	DVP	2
2	2/13/21 10:40	H16.0		1	Úlcera corneal	2
1	2/13/21 10:44	H43.81		7	DVP	2
2	2/13/21 10:45	H35.30		8	DMAE	2
1	2/13/21 10:57	T15.0		1	Cuerpo extraño	2
2	2/13/21 11:20	H10.3		0	Conjuntivitis	2
2	2/13/21 11:26	G43.9		10	Migraña	2
2	2/13/21 11:47	H16.9		1	Queratitis	2
2	2/13/21 12:11	H20		6	Uveitis	2
1	2/13/21 12:53	T26		9	Causticación	2
1	2/13/21 13:16	H05.01		4	Celulitis orbitaria	2
1	2/13/21 13:31	H40.9		3	Glaucoma	2
2	2/13/21 13:35	H40.05		3	HTO	2
2	2/13/21 14:24	H16.0		1	Úlcera corneal	2
1	2/13/21 14:48	H16.9		1	Queratitis	2
2	2/13/21 15:07	H16.0		1	Úlcera corneal	2
2	2/13/21 15:15	H40.05		3	HTO	2

1	2/13/21 15:51	H16.9	1	Queratitis	2
2	2/13/21 16:54	H20	6	Uveitis	2
1	2/13/21 17:16	H43.81	7	DVP	2
1	2/13/21 18:38	H43.81	7	DVP	2
2	2/13/21 19:20	H43.81	7	DVP	2
1	2/13/21 20:34	H10.3	0	Conjuntivitis	2
1	2/13/21 20:39	H16.9	1	Queratitis	2
2	2/14/21 0:23	H02	4	Alteración palpebral	2
2	2/14/21 0:55	H11.44	0	Quiste conjuntival	2
1	2/14/21 9:57	H16.0	1	Úlcera corneal	2
2	2/14/21 10:11	T26	9	Causticación	2
1	2/14/21 10:26	H16.0	1	Úlcera corneal	2
1	2/14/21 11:41	H10.3	0	Conjuntivitis	2
1	2/14/21 11:50	H00.19	4	Chalazión	2
1	2/14/21 11:59	T15.0	1	Cuerpo extraño	2
1	2/14/21 13:05	H40.9	3	Glaucoma	2
2	2/14/21 13:15	H33.0	8	Desprendimiento de retina	2
2	2/14/21 15:31	H0.41	1	Ojo seco	2
2	2/14/21 15:44	H50.9	10	Estrabismo	2
1	2/14/21 15:45	H16.0	1	Úlcera corneal	2
2	2/14/21 16:12	H0.41	1	Ojo seco	2
1	2/14/21 17:31	H10.3	1	Conjuntivitis	2
1	2/14/21 17:41	H16.0	1	Úlcera corneal	2
2	2/14/21 17:51	H00.19	4	Chalazión	2
2	2/14/21 18:21	H16.9	1	Queratitis	2
1	2/14/21 18:51	H10.3	0	Conjuntivitis	2
2	2/14/21 19:16	H16.0	1	Úlcera corneal	2
1	2/14/21 20:29	Z97.0	4	Protesis ocular	2
2	2/14/21 22:37	H16.0	1	Úlcera corneal	2
1	2/15/21 9:33	H10.3	0	Conjuntivitis	2
2	2/15/21 10:12	H02	4	Alteración palpebral	2
2	2/15/21 10:13	H10.3	0	Conjuntivitis	2
1	2/15/21 10:34	H43.81	7	DVP	2
2	2/15/21 10:52	S05.9	9	Traumatismo	2
1	2/15/21 10:57	H16.0	1	Úlcera corneal	2
1	2/15/21 11:01	H01.00	4	Blefaritis	2
2	2/15/21 11:14	H43.81	7	DVP	2
2	2/15/21 11:25	H01.00	4	Blefaritis	2
1	2/15/21 11:27	H00.02	4	Orzuelo	2
2	2/15/21 11:29	G43.9	10	Migraña	2
2	2/15/21 11:39	H01.00	4	Blefaritis	2
2	2/15/21 11:55	H04.32	5	Dacriocistitis aguda	2
2	2/15/21 11:56	H10.3	0	Conjuntivitis	2
2	2/15/21 12:04	H20	6	Uveitis	2
2	2/15/21 12:19	H11.3	0	Hiposfagma	2
2	2/15/21 12:30	H10.3	0	Conjuntivitis	2
1	2/15/21 12:35	H02	4	Lesión palpebral	2
2	2/15/21 12:39	H10.3	0	Conjuntivitis	2
2	2/15/21 14:10	H10.3	0	Conjuntivitis	2
2	2/15/21 15:03	H10.3	0	Conjuntivitis	2
2	2/15/21 15:05	H16.9	1	Queratitis	2
2	2/15/21 15:07	H10.3	0	Conjuntivitis	2
2	2/15/21 16:07	H00.02	4	Orzuelo	2
2	2/15/21 16:14	H10.81	0	Pingueculitis	2
1	2/15/21 16:27	H10.3	0	Conjuntivitis	2
1	2/15/21 16:52	H43.81	7	DVP	2
2	2/15/21 17:15	H16.0	1	Úlcera corneal	2
2	2/15/21 18:01	H35.30	8	DMAE	2
1	2/15/21 18:03	H20	6	Uveitis	2
2	2/15/21 20:08	H16.0	1	Úlcera corneal	2
1	2/15/21 20:28	T15.0	1	Cuerpo extraño	2
2	2/15/21 20:40	H16.0	1	Úlcera corneal	2
1	2/15/21 21:11	T15.0	1	Cuerpo extraño	2
2	2/15/21 21:46	H16.0	1	Úlcera corneal	2
1	2/16/21 8:36	H26.9	2	Catarata	2
2	2/16/21 8:36	H16.0	1	Úlcera corneal	2
1	2/16/21 9:00	H33.0	8	Desprendimiento de retina	2
1	2/16/21 9:02	H16.9	1	Queratitis	2
1	2/16/21 9:15	H16.9	1	Queratitis	2
2	2/16/21 9:28	H43.81	7	DVP	2
1	2/16/21 9:42	H43.81	7	DVP	2

2	2/16/21 10:13	H11.3	0	Hiposfagma	2
1	2/16/21 10:15	H01.00	4	Blefaritis	2
2	2/16/21 11:17	H01.00	4	Blefaritis	2
2	2/16/21 11:25	H40.05	3	HTO	2
1	2/16/21 11:28	S05.30	0	Laceración conjuntival	2
2	2/16/21 12:03	H17.8	1	Infiltrados corneales	2
2	2/16/21 12:10	H02	4	Lesión palpebral	2
2	2/16/21 12:24	H05.01	4	Celulitis orbitaria	2
1	2/16/21 12:49	H01.00	4	Blefaritis	2
2	2/16/21 16:05	H0.41	1	Ojo seco	2
1	2/16/21 16:08	H16.0	1	Úlcera corneal	2
2	2/16/21 16:11	H11.3	0	Hiposfagma	2
2	2/16/21 16:22	H40.05	3	HTO	2
2	2/16/21 16:26	H15.1	6	Epiescleritis	2
2	2/16/21 16:41	H02	4	Alteración palpebral	2
1	2/16/21 16:56	H00.02	4	Orzuelo	2
1	2/16/21 17:40	H00.02	4	Orzuelo	2
1	2/16/21 17:43	H16.0	1	Úlcera corneal	2
1	2/16/21 18:26	T15.0	1	Cuerpo extraño	2
2	2/16/21 18:44	H05.01	4	Celulitis orbitaria	2
1	2/16/21 19:09	H16.0	1	Úlcera corneal	2
2	2/16/21 19:55	H05.01	4	Celulitis orbitaria	2
2	2/16/21 20:08	H53	11	No patología	2
1	2/16/21 20:26	T15.0	1	Cuerpo extraño	2
1	2/16/21 20:27	H16.0	1	Úlcera corneal	2
1	2/16/21 21:06	G43.9	10	Migraña	2
1	2/16/21 21:45	H33.30	8	Desgarro retiniano	2
2	2/16/21 21:57	H46	10	Neuritis óptica	2
1	2/16/21 22:37	H11.3	0	Hiposfagma	2
1	2/17/21 8:50	H43.81	7	DVP	2
2	2/17/21 9:57	H17.8	1	Infiltrados corneales	2
2	2/17/21 10:51	H20	6	Uveitis	2
1	2/17/21 10:59	Z98.4	2	Post op catarata	2
1	2/17/21 11:11	H43.81	7	DVP	2
2	2/17/21 11:17	H27.8	2	OCP	2
2	2/17/21 11:28	H10.3	0	Conjuntivitis	2
1	2/17/21 11:58	H27.8	2	OCP	2
2	2/17/21 12:10	H35.30	8	DMAE	2
2	2/17/21 12:18	H11.3	0	Hiposfagma	2
2	2/17/21 13:45	H16.9	1	Queratitis	2
2	2/17/21 14:04	T15.0	1	Cuerpo extraño	2
2	2/17/21 14:43	H16.0	1	Úlcera corneal	2
1	2/17/21 15:26	H16.0	1	Úlcera corneal	2
2	2/17/21 15:51	Z98.4	2	Post op catarata	2
1	2/17/21 16:18	T15.0	1	Cuerpo extraño	2
2	2/17/21 16:45	H10.3	0	Conjuntivitis	2
1	2/17/21 16:54	H11.3	0	Hiposfagma	2
1	2/17/21 17:01	H16.9	1	Queratitis	2
1	2/17/21 17:29	H16.0	1	Úlcera corneal	2
2	2/17/21 17:57	H33.30	8	Desgarro retiniano	2
1	2/17/21 18:02	H16.0	1	Úlcera corneal	2
1	2/17/21 18:09	H16.0	1	Úlcera corneal	2
1	2/17/21 18:59	T26	9	Causticación	2
2	2/17/21 19:03	H11.44	0	Quiste conjuntival	2
1	2/17/21 19:04	H33.0	8	Desprendimiento de retina	2
1	2/17/21 20:12	T15.0	1	Cuerpo extraño	2
1	2/17/21 20:21	H16.0	1	Úlcera corneal	2
1	2/17/21 20:32	H59.3	5	Postop via lagrimal	2
2	2/17/21 20:36	S05.30	9	Laceración conjuntival	2
1	2/17/21 20:37	H16.9	1	Queratitis	2
2	2/17/21 20:47	H0.41	1	Ojo seco	2
1	2/17/21 20:50	T15.0	1	Cuerpo extraño	2
1	2/17/21 20:57	H43.81	7	DVP	2
1	2/17/21 21:03	T15.0	1	Cuerpo extraño	2
1	2/17/21 21:26	H16.9	1	Queratitis	2
1	2/17/21 22:37	H00.02	4	Orzuelo	2
2	2/17/21 23:26	H02	4	Alteración palpebral	2
1	2/18/21 1:25	H16.9	1	Queratitis	2
2	2/18/21 8:15	H02	4	Alteración palpebral	2
1	2/18/21 9:16	H10.3	0	Conjuntivitis	2
1	2/18/21 10:03	H16.9	1	Queratitis	2

2	2/18/21 10:06	H10.3	0	Conjuntivitis	2
1	2/18/21 10:11	H01.00	4	Blefaritis	2
1	2/18/21 11:27	H16.9	1	Queratitis	2
2	2/18/21 11:49	H16.9	1	Queratitis	2
2	2/18/21 11:59	H16.0	1	Úlcera corneal	2
2	2/18/21 12:19	H11.3	0	Hiposfagma	2
1	2/18/21 12:19	H53.10	11	Problema refractivo	2
1	2/18/21 12:22	G43.9	10	Migraña	2
1	2/18/21 12:34	T15.0	1	Cuerpo extraño	2
2	2/18/21 12:49	H01.00	4	Blefaritis	2
2	2/18/21 13:16	H15.00	6	Escleritis	2
1	2/18/21 13:23	H43.81	7	DVP	2
2	2/18/21 14:34	H26.9	2	Catarata	2
2	2/18/21 15:15	H10.3	0	Conjuntivitis	2
2	2/18/21 15:22	H16.9	1	Queratitis	2
2	2/18/21 15:37	H0.41	1	Ojo seco	2
1	2/18/21 15:40	H16.9	1	Queratitis	2
2	2/18/21 16:18	H16.9	1	Queratitis	2
1	2/18/21 16:33	H35.30	8	DMAE	2
1	2/18/21 17:56	H16.0	1	Úlcera corneal	2
1	2/18/21 18:47	H11.3	0	Hiposfagma	2
1	2/18/21 20:12	H00.02	4	Orzuelo	2
2	2/18/21 21:07	H11.3	0	Hiposfagma	2
2	2/18/21 21:58	H16.9	1	Queratitis	2
2	2/18/21 22:16	H11.3	0	Hiposfagma	2
1	2/18/21 22:34	H10.3	0	Conjuntivitis	2
2	2/18/21 23:51	G43.9	10	Migraña	2
2	2/19/21 1:32	H16.9	1	Queratitis	2
2	2/19/21 1:51	H15.1	6	Epiescleritis	2
1	2/19/21 2:33	H16.9	1	Queratitis	2
1	2/19/21 9:18	H01.00	4	Blefaritis	2
2	2/19/21 9:33	H16.9	1	Queratitis	2
2	2/19/21 9:57	H43.81	7	DVP	2
2	2/19/21 11:21	H33.0	8	Desprendimiento de retina	2
2	2/19/21 12:02	H01.00	4	Blefaritis	2
2	2/19/21 12:13	H16.9	1	Queratitis	2
2	2/19/21 12:48	H00.02	4	Orzuelo	2
1	2/19/21 14:04	H00.02	4	Orzuelo	2
1	2/19/21 14:06	H01.00	4	Blefaritis	2
1	2/19/21 14:42	H16.9	1	Queratitis	2
1	2/19/21 15:04	H16.9	1	Queratitis	2
2	2/19/21 15:51	H43.81	7	DVP	2
1	2/19/21 17:12	S05.9	9	Traumatismo	2
1	2/19/21 18:40	T15.0	1	Cuerpo extraño	2
1	2/19/21 18:52	T15.0	1	Cuerpo extraño	2
1	2/19/21 19:40	T15.0	1	Cuerpo extraño	2
2	2/19/21 19:45	H10.81	0	Pingueculitis	2
2	2/19/21 20:32	H16.0	1	Úlcera corneal	2
1	2/19/21 20:53	H16.0	1	Úlcera corneal	2
1	2/20/21 0:09	H00.03	4	Celulitis preseptal	2
2	2/20/21 0:14	H16.9	1	Queratitis	2
1	2/20/21 0:16	H11.00	0	Pterigium	2
1	2/20/21 2:38	H16.9	1	Queratitis	2
1	2/20/21 7:37	H16.9	1	Queratitis	2
2	2/20/21 9:10	H01.00	4	Blefaritis	2
2	2/20/21 9:11	H10.5	0	Blefaroconjuntivitis	2
1	2/20/21 9:56	S05.9	9	Traumatismo	2
1	2/20/21 10:18	S05.9	9	Traumatismo	2
2	2/20/21 10:59	H27.8	2	OCP	2
1	2/20/21 11:38	H33.0	8	Desprendimiento de retina	2
2	2/20/21 11:38	H11.3	0	Hiposfagma	2
1	2/20/21 11:55	T15.0	1	Cuerpo extraño	2
2	2/20/21 12:05	H20	6	Uveitis	2
1	2/20/21 12:11	H59.3	4	Post op párpados	2
1	2/20/21 13:05	H10.3	0	Conjuntivitis	2
1	2/20/21 13:28	H16.0	1	Úlcera corneal	2
1	2/20/21 13:35	H16.0	1	Úlcera corneal	2
1	2/20/21 13:42	H16.9	1	Queratitis	2
1	2/20/21 14:50	T15.0	1	Cuerpo extraño	2
1	2/20/21 15:26	H27.8	2	OCP	2
1	2/20/21 15:48	H16.0	1	Úlcera corneal	2

2	2/20/21 15:54	H0.41	1	Ojo seco	2
1	2/20/21 16:18	S05.9	9	Traumatismo	2
1	2/20/21 16:54	S05.9	9	Traumatismo	2
1	2/20/21 17:09	H16.9	1	Queratitis	2
2	2/20/21 17:51	H16.0	1	Úlcera corneal	2
2	2/20/21 18:50	H11.44	0	Quiste conjuntival	2
2	2/20/21 19:10	H10.3	0	Conjuntivitis	2
1	2/20/21 20:00	H16.0	1	Úlcera corneal	2
1	2/20/21 20:08	T15.0	1	Cuerpo extraño	2
1	2/20/21 20:41	H16.0	1	Úlcera corneal	2
1	2/20/21 21:12	H16.9	1	Queratitis	2
1	2/20/21 21:13	H16.0	1	Úlcera corneal	2
1	2/20/21 21:25	H20	6	Uveitis	2
2	2/20/21 21:46	H16.0	1	Úlcera corneal	2
1	2/21/21 7:13	H10.3	0	Conjuntivitis	2
2	2/21/21 8:39	H05.01	4	Celulitis orbitaria	2
2	2/21/21 9:01	H16.9	1	Queratitis	2
2	2/21/21 10:02	H00.02	4	Orzuelo	2
1	2/21/21 10:29	H11.00	0	Pterigium	2
1	2/21/21 10:29	H20	6	Uveitis	2
2	2/21/21 10:42	H16.0	1	Úlcera corneal	2
2	2/21/21 11:02	H00.02	4	Orzuelo	2
2	2/21/21 11:07	H00.02	4	Orzuelo	2
1	2/21/21 11:11	H16.9	1	Queratitis	2
1	2/21/21 11:18	H16.0	1	Úlcera corneal	2
2	2/21/21 11:30	H05.01	4	Celulitis orbitaria	2
1	2/21/21 12:12	Z98.4	2	Post op catarata	2
2	2/21/21 12:41	H01.00	4	Blefaritis	2
1	2/21/21 12:46	H01.00	4	Blefaritis	2
1	2/21/21 13:56	H11.3	0	Hiposfagma	2
2	2/21/21 15:04	H33.30	8	Desgarro retiniano	2
2	2/21/21 15:30	H16.9	1	Queratitis	2
1	2/21/21 16:23	H53.10	11	Problema refractivo	2
1	2/21/21 17:23	T15.0	1	Cuerpo extraño	2
2	2/21/21 17:27	H16.9	1	Queratitis	2
2	2/21/21 17:31	H10.3	0	Conjuntivitis	2
1	2/21/21 17:57	H00.02	4	Orzuelo	2
2	2/21/21 19:48	H16.9	1	Queratitis	2
2	2/21/21 19:55	H20	6	Uveitis	2
1	2/21/21 20:10	T15.0	1	Cuerpo extraño	2
1	2/21/21 21:03	H05.01	4	Celulitis orbitaria	2
2	2/21/21 22:36	G43.9	10	Migraña	2
1	2/21/21 23:23	H10.3	0	Conjuntivitis	2
2	2/22/21 7:26	H10.3	0	Conjuntivitis	2
2	2/22/21 8:00	H11.3	0	Hiposfagma	2
1	2/22/21 8:33	T15.0	1	Cuerpo extraño	2
2	2/22/21 9:24	H16.0	1	Úlcera corneal	2
2	2/22/21 10:05	H16.9	1	Queratitis	2
1	2/22/21 10:40	H10.3	0	Conjuntivitis	2
2	2/22/21 11:06	H35.3	8	Maculopatía	2
2	2/22/21 11:39	S05.9	9	Traumatismo	2
1	2/22/21 11:53	H16.9	1	Queratitis	2
2	2/22/21 12:30	H43.81	7	DVP	2
1	2/22/21 12:42	H11.3	0	Hiposfagma	2
2	2/22/21 13:37	H26.9	2	Catarata	2
1	2/22/21 13:46	H02	4	Alteración palpebral	2
1	2/22/21 14:07	H01.00	4	Blefaritis	2
2	2/22/21 15:04	H10.3	0	Conjuntivitis	2
1	2/22/21 15:18	G43.9	10	Migraña	2
1	2/22/21 15:50	H15.1	6	Epiescleritis	2
2	2/22/21 15:54	H01.00	4	Blefaritis	2
2	2/22/21 16:16	H43.81	7	DVP	2
2	2/22/21 16:26	H11.3	0	Hiposfagma	2
2	2/22/21 17:10	H11.3	0	Hiposfagma	2
2	2/22/21 18:01	H18.50	1	Distrofia corneal	2
1	2/22/21 18:19	T15.0	1	Cuerpo extraño	2
1	2/22/21 19:37	H10.3	1	Conjuntivitis	2
2	2/22/21 20:05	H15.1	6	Epiescleritis	2
2	2/22/21 20:09	H16.0	1	Úlcera corneal	2
2	2/22/21 20:12	G43.9	10	Migraña	2
1	2/22/21 20:15	T15.0	1	Cuerpo extraño	2

1	2/22/21 21:13	T15.0	1	Cuerpo extraño	2
2	2/22/21 23:49	H10.3	0	Conjuntivitis	2
2	2/23/21 8:08	H11.3	0	Hiposfagma	2
2	2/23/21 9:19	H33.30	8	Desgarro retiniano	2
2	2/23/21 9:49	H10.3	0	Conjuntivitis	2
2	2/23/21 10:02	H10.3	0	Conjuntivitis	2
2	2/23/21 10:24	H43.81	7	DVP	2
1	2/23/21 10:41	T15.0	1	Cuerpo extraño	2
1	2/23/21 12:24	H05.01	4	Celulitis orbitaria	2
2	2/23/21 12:28	H20	6	Uveitis	2
1	2/23/21 12:54	H16.0	1	Úlcera corneal	2
2	2/23/21 12:58	H43.81	7	DVP	2
2	2/23/21 13:00	H01.00	4	Blefaritis	2
2	2/23/21 14:12	G43.9	10	Migraña	2
2	2/23/21 15:17	H43.81	7	DVP	2
2	2/23/21 16:14	H10.3	0	Conjuntivitis	2
1	2/23/21 16:23	S05.9	9	Traumatismo	2
2	2/23/21 16:39	H16.9	1	Queratitis	2
1	2/23/21 17:25	H53.9	11	Alteraciones visuales	2
1	2/23/21 20:12	H11.82	0	Conjuntivocalasia	2
1	2/23/21 20:45	T15.0	1	Cuerpo extraño	2
2	2/23/21 22:38	H16.0	1	Úlcera corneal	2
2	2/23/21 23:02	H15.1	6	Epiescleritis	2
1	2/23/21 23:16	S05.9	9	Traumatismo	2
1	2/23/21 23:24	H10.3	0	Conjuntivitis	2
1	2/24/21 0:16	T15.0	1	Cuerpo extraño	2
2	2/24/21 4:52	H16.0	1	Úlcera corneal	2
2	2/24/21 9:26	H10.3	0	Conjuntivitis	2
2	2/24/21 9:45	H10.3	0	Conjuntivitis	2
1	2/24/21 9:57	S05.9	9	Traumatismo	2
2	2/24/21 10:13	H16.9	1	Queratitis	2
2	2/24/21 10:24	H10.3	0	Conjuntivitis	2
2	2/24/21 10:39	H43.81	7	DVP	2
2	2/24/21 10:40	H43.81	7	DVP	2
2	2/24/21 10:42	H34.82	8	Trombosis venosa	2
1	2/24/21 11:03	Z97.0	4	Protesis ocular	2
2	2/24/21 11:12	H59.88	8	Post op retina	2
2	2/24/21 11:24	H11.3	0	Hiposfagma	2
2	2/24/21 12:43	H15.1	6	Epiescleritis	2
2	2/24/21 13:13	H16.9	1	Queratitis	2
2	2/24/21 13:19	H15.1	6	Epiescleritis	2
2	2/24/21 13:41	H16.9	1	Queratitis	2
2	2/24/21 13:59	H00.03	4	Celulitis preseptal	2
2	2/24/21 14:18	H10.3	0	Conjuntivitis	2
2	2/24/21 14:19	H43.81	7	DVP	2
1	2/24/21 15:21	B00.1	4	Dermatitis herpética	2
2	2/24/21 15:34	H20	6	Uveitis	2
2	2/24/21 15:37	H16.9	1	Queratitis	2
1	2/24/21 15:50	H16.0	1	úlcera corneal	2
1	2/24/21 16:11	S05.9	9	Traumatismo	2
2	2/24/21 16:20	H0.41	1	Ojo seco	2
2	2/24/21 16:26	H16.9	1	Queratitis	2
2	2/24/21 16:38	H16.0	1	úlcera corneal	2
2	2/24/21 16:51	H35.30	8	DMAE	2
1	2/24/21 16:56	S05.9	9	Traumatismo	2
1	2/24/21 17:18	H43.81	7	DVP	2
1	2/24/21 17:20	H26.9	2	Catarata	2
2	2/24/21 18:04	H16.9	1	Queratitis	2
1	2/24/21 18:41	H43.81	7	DVP	2
1	2/24/21 18:45	T15.0	1	Cuerpo extraño	2
2	2/24/21 18:52	H10.3	0	Conjuntivitis	2
2	2/24/21 19:09	Alta por fuga	11	Alta por fuga	2
1	2/24/21 19:12	H16.0	1	úlcera corneal	2
1	2/24/21 19:51	S05.30	0	Laceración conjuntival	2
1	2/24/21 19:56	T15.0	1	Cuerpo extraño	2
1	2/24/21 20:30	H20	6	Uveitis	2
2	2/24/21 20:50	G43.9	10	Migraña	2
2	2/24/21 21:25	H20	6	Uveitis	2
2	2/24/21 21:34	T15.0	1	Cuerpo extraño	2
1	2/25/21 5:02	H16.0	1	Úlcera corneal	2
1	2/25/21 6:10	H43.1	7	Hemovítreo	2

2	2/25/21 7:58	H20		6	Uveitis	2
2	2/25/21 8:14	H43.81		7	DVP	2
2	2/25/21 9:44	H43.81		7	DVP	2
1	2/25/21 9:51	H02		4	Alteración palpebral	2
2	2/25/21 10:00	H33.30		8	Desgarro retiniano	2
2	2/25/21 10:37	H43.81		7	DVP	2
1	2/25/21 10:51	H11.3		0	Hiposfagma	2
1	2/25/21 11:38	H43.81		7	DVP	2
2	2/25/21 11:38	H16.9		1	Queratitis	2
2	2/25/21 11:41	H10.3		0	Conjuntivitis	2
1	2/25/21 11:46	H04.32		5	Dacriocistitis aguda	2
1	2/25/21 12:07	H16.0		1	Úlcera corneal	2
1	2/25/21 12:16	H20		6	Uveitis	2
2	2/25/21 12:28	H00.02		4	Orzuelo	2
2	2/25/21 12:46	H10.3		0	Conjuntivitis	2
1	2/25/21 12:56	H10.81		0	Pingueculitis	2
2	2/25/21 14:49	H16.0		1	Úlcera corneal	2
2	2/25/21 15:07	H26.9		2	Catarata	2
2	2/25/21 15:40	H43.81		7	DVP	2
2	2/25/21 15:44	H10.3		0	Conjuntivitis	2
1	2/25/21 16:56	H16.0		1	Úlcera corneal	2
1	2/25/21 17:03	T15.0		1	Cuerpo extraño	2
1	2/25/21 17:06	H16.9		1	Queratitis	2
2	2/25/21 17:31	H0.41		1	Ojo seco	2
1	2/25/21 17:34	H10.3		0	Conjuntivitis	2
1	2/25/21 17:40	H33.0		8	Desprendimiento de retina	2
1	2/25/21 18:05	H16.0		1	Úlcera corneal	2
2	2/25/21 18:20	G43.9		10	Migraña	2
1	2/25/21 18:20	H16.9		1	Queratitis	2
2	2/25/21 18:26	H10.3		0	Conjuntivitis	2
1	2/25/21 19:07	H16.0		1	Úlcera corneal	2
1	2/25/21 19:22	E11.319		8	Retinopatía diabética	2
1	2/25/21 19:25	H10.3		0	Conjuntivitis	2
2	2/25/21 19:31	S05.9		9	Traumatismo	2
1	2/25/21 19:55	T15.0		1	Cuerpo extraño	2
2	2/25/21 20:23	H43.81		7	DVP	2
1	2/25/21 21:08	H10.3		0	Conjuntivitis	2
1	2/25/21 23:39	H43.81		7	DVP	2
2	2/26/21 0:43	H16.0		1	Úlcera corneal	2
1	2/26/21 9:16	H16.9		1	Queratitis	2
2	2/26/21 9:38	H46		10	Neuritis óptica	2
2	2/26/21 9:48	S05.30		0	Laceración conjuntival	2
2	2/26/21 9:55	H01.00		4	Blefaritis	2
1	2/26/21 10:15	H43.1		7	Hemovítreo	2
1	2/26/21 10:18	H10.3		0	Conjuntivitis	2
2	2/26/21 10:43	S05.9		9	Traumatismo	2
2	2/26/21 10:46	H11.3		0	Hiposfagma	2
2	2/26/21 11:18	H01.00		4	Blefaritis	2
2	2/26/21 11:26	H10.3		0	Conjuntivitis	2
2	2/26/21 11:44	H11.44		0	Quiste conjuntival	2
2	2/26/21 12:18	H10.3		0	Conjuntivitis	2
2	2/26/21 12:54	H16.9		1	Queratitis	2
2	2/26/21 14:09	H0.41		1	Ojo seco	2
2	2/26/21 14:13	H0.41		1	Ojo seco	2
2	2/26/21 15:24	H10.3		0	Conjuntivitis	2
1	2/26/21 15:58	S05.9		9	Traumatismo	2
2	2/26/21 15:59	H43.81		7	DVP	2
1	2/26/21 16:02	H11.3		0	Hiposfagma	2
1	2/26/21 16:08	T15.0		1	Cuerpo extraño	2
2	2/26/21 17:14	S05.9		9	Traumatismo	2
2	2/26/21 17:17	H35.3		8	Maculopatía	2
2	2/26/21 18:01	H10.3		0	Conjuntivitis	2
1	2/26/21 18:56	T15.0		1	Cuerpo extraño	2
1	2/26/21 19:09	H16.9		1	Queratitis	2
2	2/26/21 19:52	H16.0		1	Úlcera corneal	2
2	2/26/21 20:11	H16.9		1	Queratitis	2
1	2/26/21 20:11	S05.9		9	Traumatismo	2
1	2/26/21 20:40	H10.3		0	Conjuntivitis	2
1	2/26/21 20:44	T15.0		1	Cuerpo extraño	2
1	2/26/21 20:51	T15.0		1	Cuerpo extraño	2
1	2/26/21 21:28	H16.0		1	Úlcera corneal	2

2	2/26/21 21:51	H16.9	1	Queratitis	2
1	2/26/21 22:29	H16.0	1	Úlcera corneal	2
1	2/26/21 22:39	H00.03	4	Celulitis preseptal	2
2	2/26/21 23:27	H16.9	1	Queratitis	2
1	2/27/21 3:18	H16.9	1	Queratitis	2
1	2/27/21 7:27	H16.9	1	Queratitis	2
2	2/27/21 8:02	H16.0	1	Úlcera corneal	2
2	2/27/21 9:26	H00.02	4	Orzuelo	2
1	2/27/21 9:36	H16.9	1	Queratitis	2
2	2/27/21 10:16	H11.3	0	Hiposfagma	2
1	2/27/21 10:37	G43.9	10	Migraña	2
2	2/27/21 10:41	H35.3	8	Maculopatía	2
2	2/27/21 10:54	H43.81	7	DVP	2
1	2/27/21 10:57	T15.0	1	Cuerpo extraño	2
2	2/27/21 11:11	H20	6	Uveitis	2
1	2/27/21 11:19	H33.0	8	Desprendimiento de retina	2
1	2/27/21 11:36	S05.6	9	Perforación ocular	2
2	2/27/21 11:42	H20	6	Uveitis	2
1	2/27/21 12:00	T15.0	1	Cuerpo extraño	2
1	2/27/21 12:19	H00.02	4	Orzuelo	2
2	2/27/21 12:35	H17.8	1	Infiltrados corneales	2
1	2/27/21 12:36	H16.0	1	Úlcera corneal	2
1	2/27/21 12:45	T15.0	1	Cuerpo extraño	2
2	2/27/21 13:30	H11.3	0	Hiposfagma	2
2	2/27/21 13:35	H10.3	0	Conjuntivitis	2
2	2/27/21 13:56	H43.81	7	DVP	2
2	2/27/21 13:59	H16.9	1	Queratitis	2
1	2/27/21 14:13	T15.0	1	Cuerpo extraño	2
2	2/27/21 14:16	H0.41	1	Ojo seco	2
1	2/27/21 14:59	H16.0	1	Úlcera corneal	2
1	2/27/21 15:02	T15.0	1	Cuerpo extraño	2
1	2/27/21 15:19	T15.0	1	Cuerpo extraño	2
1	2/27/21 15:28	T15.0	1	Cuerpo extraño	2
2	2/27/21 15:37	H00.02	4	Orzuelo	2
2	2/27/21 15:50	G43.9	10	Migraña	2
1	2/27/21 16:19	T15.0	1	Cuerpo extraño	2
1	2/27/21 16:34	H20	6	Uveitis	2
1	2/27/21 16:37	H16.9	1	Queratitis	2
2	2/27/21 16:51	H16.9	1	Queratitis	2
1	2/27/21 16:51	T15.0	1	Cuerpo extraño	2
1	2/27/21 17:14	H16.0	1	Úlcera corneal	2
2	2/27/21 17:47	H01.00	4	Blefaritis	2
1	2/27/21 18:00	H10.3	0	Conjuntivitis	2
1	2/27/21 18:01	H33.0	8	Desprendimiento de retina	2
1	2/27/21 18:06	S05.9	9	Traumatismo	2
2	2/27/21 18:26	H16.0	1	Úlcera corneal	2
2	2/27/21 19:06	H11.3	0	Hiposfagma	2
1	2/27/21 20:32	H43.81	7	DVP	2
1	2/27/21 22:29	T15.0	1	Cuerpo extraño	2
1	2/27/21 22:43	H16.9	1	Queratitis	2
2	2/28/21 3:20	H00.02	4	Orzuelo	2
2	2/28/21 9:55	S05.9	9	Traumatismo	2
1	2/28/21 10:14	H10.3	0	Conjuntivitis	2
2	2/28/21 10:15	H16.0	2	Atalamia	2
1	2/28/21 10:55	T15.0	1	Cuerpo extraño	2
1	2/28/21 11:12	T15.0	1	Cuerpo extraño	2
2	2/28/21 11:17	H16.9	1	Queratitis	2
2	2/28/21 11:29	H11.3	0	Hiposfagma	2
2	2/28/21 12:16	G43.9	10	Migraña	2
2	2/28/21 12:16	H35.3	8	Maculopatía	2
2	2/28/21 12:24	H33.30	8	Desgarro retiniano	2
2	2/28/21 12:26	T15.0	1	Cuerpo extraño	2
2	2/28/21 13:20	H16.0	1	Úlcera corneal	2
2	2/28/21 13:30	H02	4	Alteración palpebral	2
1	2/28/21 14:18	H02	4	Alteración palpebral	2
2	2/28/21 14:49	H16.9	1	Queratitis	2
2	2/28/21 16:03	H11.3	0	Hiposfagma	2
2	2/28/21 16:18	H16.0	1	Úlcera corneal	2
2	2/28/21 17:45	H43.81	7	DVP	2
2	2/28/21 17:45	H10.3	0	Conjuntivitis	2
1	2/28/21 18:29	H16.9	1	Queratitis	2

2	2/28/21 18:39	H10.3	0	Conjuntivitis	2
1	2/28/21 18:46	H16.0	1	Úlcera corneal	2
1	2/28/21 19:04	H20	6	Uveitis	2
1	2/28/21 21:51	H16.9	1	Queratitis	2
1	2/28/21 22:30	T15.0	1	Cuerpo extraño	2
2	2/28/21 22:33	H20	6	Uveitis	2
Sexo	Fecha	Diagnóstico	Grupo	Diagnóstico	COVID: 1 NO 2 SI
1	01/09/2019	S05.9	9	Traumatismo	1
1	01/09/2019	H15.1	6	Epiescleritis	1
1	01/09/2019	H20	6	Uveitis	1
1	01/09/2019	H16.9	1	Queratitis	1
1	01/09/2019	H20	6	Uveitis	1
1	01/09/2019	H15.1	6	Epiescleritis	1
2	01/09/2019	S05.9	9	Traumatismo	1
2	01/09/2019	H11.3	1	Hiposfagma	1
2	01/09/2019	H00.02	4	Orzuelo	1
2	01/09/2019	H10.3	0	Conjuntivitis	1
1	01/09/2019	H35.3	8	Maculopatía	1
1	01/09/2019	H16.9	1	Queratitis	1
2	01/09/2019	H16.9	1	Queratitis	1
2	01/09/2019	H10.3	0	Conjuntivitis	1
2	01/09/2019	H16.9	1	Queratitis	1
1	01/09/2019	H16.0	1	Úlcera corneal	1
1	01/09/2019	H10.3	0	Conjuntivitis	1
2	01/09/2019	H0.41	1	Ojo seco	1
2	01/09/2019	H43.81	7	DVP	1
1	01/09/2019	H16.0	1	Úlcera corneal	1
2	01/09/2019	H10.3	0	Conjuntivitis	1
1	01/09/2019	H10.3	0	Conjuntivitis	1
2	01/09/2019	H35.3	8	Maculopatía	1
2	01/09/2019	H11.3	0	Hiposfagma	1
1	01/09/2019	T15.0	1	Cuerpo extraño	1
1	01/09/2019	H16.9	1	Queratitis	1
2	01/09/2019	H11.3	0	Hiposfagma	1
2	01/09/2019	H02	4	Lesión palpebral	1
2	01/09/2019	H43.1	8	Hemovítreo	1
2	01/09/2019	H43.81	7	DVP	1
1	01/09/2019	H33.0	8	Desprendimiento de retina	1
1	01/09/2019	H16.0	1	Úlcera corneal	1
2	01/09/2019	H10.5	4	Blefaroconjuntivitis	1
2	01/09/2019	H10.3	0	Conjuntivitis	1
2	01/09/2019	T15.0	1	Cuerpo extraño	1
2	01/09/2019	H00.02	4	Orzuelo	1
1	01/09/2019	H15.1	6	Epiescleritis	1
1	01/09/2019	H33.0	8	Desprendimiento de retina	1
2	01/09/2019	H15.1	6	Epiescleritis	1
2	01/09/2019	H53	11	No patología	1
1	01/09/2019	H40.9	3	Glaucoma	1
2	01/09/2019	H16.0	1	Úlcera corneal	1
2	01/09/2019	H04.32	5	Dacriocistitis aguda	1
1	01/09/2019	T15.0	1	Cuerpo extraño	1
2	01/09/2019	H10.3	0	Conjuntivitis	1
1	02/09/2019	T15.0	1	Cuerpo extraño	1
1	02/09/2019	H16.0	1	Úlcera corneal	1
2	02/09/2019	H16.0	1	Úlcera corneal	1
1	02/09/2019	H17.8	1	Infiltrados corneales	1
2	02/09/2019	G43.9	10	Migraña	1
1	02/09/2019	H10.3	0	Conjuntivitis	1
2	02/09/2019	S05.30	9	Laceración conjuntival	1
1	02/09/2019	H20	6	Uveitis	1
1	02/09/2019	H43.81	7	DVP	1
2	02/09/2019	H43.81	7	DVP	1
1	02/09/2019	H10.3	0	Conjuntivitis	1
1	02/09/2019	H10.3	0	Conjuntivitis	1
2	02/09/2019	H02	4	Lesión palpebral	1
2	02/09/2019	H16.9	1	Queratitis	1

2	02/09/2019	H27.8	2	OCP	1
2	02/09/2019	H20	6	Uveitis	1
2	02/09/2019	H16.9	1	Queratitis	1
2	02/09/2019	H10.3	0	Conjuntivitis	1
1	02/09/2019	H10.3	0	Conjuntivitis	1
2	02/09/2019	H10.3	0	Conjuntivitis	1
2	02/09/2019	H00.02	4	Orzuelo	1
2	02/09/2019	H11.44	0	Quiste conjuntival	1
2	02/09/2019	H16.9	1	Queratitis	1
1	02/09/2019	T15.0	1	Cuerpo extraño	1
1	02/09/2019	H43.81	7	DVP	1
2	02/09/2019	T15.0	1	Cuerpo extraño	1
2	02/09/2019	H00.02	4	Orzuelo	1
1	02/09/2019	H10.3	0	Conjuntivitis	1
1	02/09/2019	S05.9	9	Traumatismo	1
2	02/09/2019	H26.9	2	Catarata	1
2	02/09/2019	H53.9	11	Alteraciones visuales	1
2	02/09/2019	H16.9	1	Queratitis	1
2	02/09/2019	H10.3	0	Conjuntivitis	1
1	02/09/2019	H16.9	1	Queratitis	1
1	02/09/2019	H10.3	0	Conjuntivitis	1
1	02/09/2019	H16.0	1	Úlcera corneal	1
1	02/09/2019	H10.3	0	Conjuntivitis	1
2	02/09/2019	H16.9	1	Queratitis	1
2	02/09/2019	H11.44	0	Quiste conjuntival	1
1	02/09/2019	T15.0	1	Cuerpo extraño	1
2	02/09/2019	H16.9	1	Queratitis	1
2	02/09/2019	H16.0	1	Úlcera corneal	1
2	02/09/2019	H10.3	0	Conjuntivitis	1
2	02/09/2019	H16.9	1	Queratitis	1
2	02/09/2019	H17.8	1	Infiltrados corneales	1
2	02/09/2019	H43.81	7	DVP	1
1	02/09/2019	T15.0	1	Cuerpo extraño	1
1	02/09/2019	H16.9	1	Queratitis	1
2	02/09/2019	H16.9	1	Queratitis	1
1	02/09/2019	T15.0	1	Cuerpo extraño	1
1	02/09/2019	H11.3	0	Hiposfagma	1
2	02/09/2019	H10.3	0	Conjuntivitis	1
1	02/09/2019	S05.9	9	Traumatismo	1
1	02/09/2019	H20	6	Uveitis	1
2	02/09/2019	H16.0	1	Úlcera corneal	1
2	02/09/2019	T15.0	1	Cuerpo extraño	1
2	03/09/2019	H20	6	Uveitis	1
1	03/09/2019	E11.319	8	Retinopatía diabética	1
2	03/09/2019	T15.0	1	Cuerpo extraño	1
2	03/09/2019	H11.3	0	Hiposfagma	1
2	03/09/2019	H11.3	0	Hiposfagma	1
1	03/09/2019	H53	11	No patología	1
2	03/09/2019	H01.00	4	Blefaritis	1
1	03/09/2019	H0.41	1	Ojo seco	1
2	03/09/2019	H16.9	1	Queratitis	1
1	03/09/2019	H34.9	8	Oclusión arterial	1
2	03/09/2019	H10.3	0	Conjuntivitis	1
1	03/09/2019	H43.81	7	DVP	1
1	03/09/2019	H10.3	0	Conjuntivitis	1
2	03/09/2019	H10.3	0	Conjuntivitis	1
2	03/09/2019	H00.03	4	Celulitis preseptal	1
1	03/09/2019	H26.9	2	Catarata	1
2	03/09/2019	H10.3	0	Conjuntivitis	1
2	03/09/2019	H00.02	4	Orzuelo	1
2	03/09/2019	H10.3	0	Conjuntivitis	1
2	03/09/2019	H01.00	4	Blefaritis	1
1	03/09/2019	H16.9	1	Queratitis	1
1	03/09/2019	H16.0	1	Úlcera corneal	1
2	03/09/2019	H16.0	1	Úlcera corneal	1
2	03/09/2019	H02	4	Lesión palpebral	1

2	03/09/2019	H00.02	4	Orzuelo	1
1	03/09/2019	H53	11	No patología	1
1	03/09/2019	H16.9	1	Queratitis	1
1	03/09/2019	H35.30	8	DMAE	1
2	03/09/2019	H16.9	1	Queratitis	1
2	03/09/2019	H43.81	7	DVP	1
1	03/09/2019	H16.0	1	Úlcera corneal	1
1	03/09/2019	H10.3	0	Conjuntivitis	1
1	03/09/2019	H11.3	0	Hiposfagma	1
1	03/09/2019	H17.8	1	Infiltrados corneales	1
2	03/09/2019	H35.30	8	DMAE	1
1	03/09/2019	H53	11	No patología	1
1	03/09/2019	H10.3	0	Conjuntivitis	1
1	03/09/2019	S05.9	9	Traumatismo	1
1	03/09/2019	H10.3	0	Conjuntivitis	1
1	03/09/2019	H00.02	4	Orzuelo	1
1	03/09/2019	H10.3	0	Conjuntivitis	1
2	03/09/2019	H10.3	0	Conjuntivitis	1
2	03/09/2019	H53	11	No patología	1
1	03/09/2019	H10.3	0	Conjuntivitis	1
2	03/09/2019	H10.3	0	Conjuntivitis	1
2	04/09/2019	H10.3	0	Conjuntivitis	1
1	04/09/2019	H11.3	0	Hiposfagma	1
1	04/09/2019	S05.9	9	Traumatismo	1
1	04/09/2019	H10.3	0	Conjuntivitis	1
1	04/09/2019	H16.9	1	Queratitis	1
2	04/09/2019	T15.0	1	Cuerpo extraño	1
2	04/09/2019	H10.3	0	Conjuntivitis	1
1	04/09/2019	H10.3	0	Conjuntivitis	1
2	04/09/2019	H10.3	0	Conjuntivitis	1
1	04/09/2019	H53	11	No patología	1
1	04/09/2019	H10.3	0	Conjuntivitis	1
1	04/09/2019	H20	6	Uveitis	1
1	04/09/2019	H10.3	0	Conjuntivitis	1
2	04/09/2019	H35.30	8	DMAE	1
2	04/09/2019	H53	11	No patología	1
1	04/09/2019	H16.0	1	Úlcera corneal	1
1	04/09/2019	H53	11	No patología	1
2	04/09/2019	H27.8	2	OCP	1
2	04/09/2019	H02.05	4	Distiquiasis	1
2	04/09/2019	H40.21	3	Glaucoma agudo	1
2	04/09/2019	H10.3	0	Conjuntivitis	1
2	04/09/2019	H02	4	Alteración palpebral	1
1	04/09/2019	H17.8	1	Infiltrados corneales	1
2	04/09/2019	H43.81	7	DVP	1
2	04/09/2019	H02	4	Alteración palpebral	1
1	04/09/2019	H10.3	0	Conjuntivitis	1
1	04/09/2019	T15.0	1	Cuerpo extraño	1
2	04/09/2019	H00.03	4	Celulitis preseptal	1
2	04/09/2019	H10.3	0	Conjuntivitis	1
1	04/09/2019	S05.9	9	Traumatismo	1
2	04/09/2019	H01.00	4	Blefaritis	1
2	04/09/2019	H10.81	0	Pingueculitis	1
1	04/09/2019	H20	6	Uveitis	1
1	04/09/2019	H10.3	0	Conjuntivitis	1
1	04/09/2019	H16.9	1	Queratitis	1
2	04/09/2019	H35.3	8	Maculopatía	1
2	04/09/2019	H16.9	1	Queratitis	1
2	04/09/2019	H53	11	No patología	1
2	04/09/2019	H17.8	1	Infiltrados corneales	1
1	04/09/2019	G43.109	10	Aura	1
1	04/09/2019	H01.00	4	Blefaritis	1
2	04/09/2019	H43.81	7	DVP	1
1	04/09/2019	T15.0	1	Cuerpo extraño	1
1	04/09/2019	H10.3	0	Conjuntivitis	1
1	04/09/2019	H16.9	1	Queratitis	1

1	04/09/2019	H53	11	No patología	1
2	04/09/2019	H53	11	No patología	1
2	04/09/2019	H10.3	0	Conjuntivitis	1
2	04/09/2019	H10.3	0	Conjuntivitis	1
1	04/09/2019	H16.0	1	Úlcera corneal	1
2	04/09/2019	H16.0	1	Úlcera corneal	1
2	04/09/2019	H43.81	7	DVP	1
1	04/09/2019	H31.42	8	Coroidopatía serosa central	1
1	04/09/2019	T15.0	1	Cuerpo extraño	1
2	04/09/2019	T15.0	1	Cuerpo extraño	1
1	04/09/2019	H35.3	8	Maculopatía	1
2	04/09/2019	H11.3	0	Hiposfagma	1
2	04/09/2019	S05.30	9	Laceración conjuntival	1
1	05/09/2019	H20	6	Uveitis	1
1	05/09/2019	Alta por fuga	11	Alta por fuga	1
1	05/09/2019	H10.3	0	Conjuntivitis	1
1	05/09/2019	H10.3	0	Conjuntivitis	1
2	05/09/2019	H10.3	0	Conjuntivitis	1
1	05/09/2019	H10.3	0	Conjuntivitis	1
1	05/09/2019	H40.21	3	Glaucoma agudo	1
2	05/09/2019	H50.9	10	Estrabismo	1
2	05/09/2019	H00.02	4	Orzuelo	1
2	05/09/2019	H20	6	Uveitis	1
2	05/09/2019	H10.3	0	Conjuntivitis	1
2	05/09/2019	H16.0	1	Úlcera corneal	1
1	05/09/2019	H26.9	2	Catarata	1
1	05/09/2019	H35.0	8	Hemorragia retiniana	1
2	05/09/2019	H10.81	0	Pingueculitis	1
1	05/09/2019	H43.81	7	DVP	1
2	05/09/2019	S05.9	9	Traumatismo	1
1	05/09/2019	H10.3	0	Conjuntivitis	1
1	05/09/2019	H02.05	4	Distiquiasis	1
2	05/09/2019	H01.00	4	Blefaritis	1
1	05/09/2019	H43.81	7	DVP	1
1	05/09/2019	H16.0	1	Úlcera corneal	1
2	05/09/2019	S05.30	9	Laceración conjuntival	1
1	05/09/2019	S05.9	9	Traumatismo	1
2	05/09/2019	H10.3	0	Conjuntivitis	1
2	05/09/2019	H01.00	4	Blefaritis	1
1	05/09/2019	H59.88	8	Post op retina	1
1	05/09/2019	H43.81	7	DVP	1
1	05/09/2019	H10.3	0	Conjuntivitis	1
2	05/09/2019	H10.3	0	Conjuntivitis	1
1	05/09/2019	H01.00	4	Blefaritis	1
2	05/09/2019	H10.3	0	Conjuntivitis	1
2	05/09/2019	G43.109	10	Aura	1
2	05/09/2019	H11.3	0	Hiposfagma	1
2	05/09/2019	H10.3	0	Conjuntivitis	1
1	05/09/2019	H16.9	1	Queratitis	1
1	05/09/2019	S05.9	9	Traumatismo	1
1	05/09/2019	H16.9	1	Queratitis	1
1	05/09/2019	H16.9	1	Queratitis	1
1	05/09/2019	H10.3	0	Conjuntivitis	1
2	05/09/2019	S05.9	9	Traumatismo	1
1	05/09/2019	H16.9	1	Queratitis	1
2	05/09/2019	H11.00	0	Pterigium	1
1	05/09/2019	H00.02	4	Orzuelo	1
1	06/09/2019	H10.3	0	Conjuntivitis	1
1	06/09/2019	H01.00	4	Blefaritis	1
2	06/09/2019	G43.109	10	Aura	1
1	06/09/2019	S05.9	9	Traumatismo	1
1	06/09/2019	H20	6	Uveitis	1
2	06/09/2019	H10.3	0	Conjuntivitis	1
2	06/09/2019	H35.30	8	DMAE	1
2	06/09/2019	H00.02	4	Orzuelo	1
2	06/09/2019	H01.00	4	Blefaritis	1

1	06/09/2019	H02	4	Alteración palpebral	1
1	06/09/2019	Z98.4	2	Post op catarata	1
2	06/09/2019	H01.00	4	Blefaritis	1
2	06/09/2019	H00.02	4	Orzuelo	1
1	06/09/2019	H33.0	8	Desprendimiento de retina	1
2	06/09/2019	H0.41	1	Ojo seco	1
2	06/09/2019	H16.9	1	Queratitis	1
1	06/09/2019	H20	6	Uveitis	1
2	06/09/2019	T15.0	1	Cuerpo extraño	1
2	06/09/2019	H20	6	Uveitis	1
1	06/09/2019	H10.3	0	Conjuntivitis	1
2	06/09/2019	H16.9	1	Queratitis	1
1	06/09/2019	H16.0	1	Úlcera corneal	1
2	06/09/2019	H10.3	0	Conjuntivitis	1
2	06/09/2019	H16.9	1	Queratitis	1
1	06/09/2019	H0.41	1	Ojo seco	1
1	06/09/2019	T15.0	1	Cuerpo extraño	1
2	06/09/2019	H20	6	Uveitis	1
2	06/09/2019	H10.3	0	Conjuntivitis	1
1	06/09/2019	H10.3	0	Conjuntivitis	1
2	06/09/2019	H10.3	0	Conjuntivitis	1
1	06/09/2019	H10.3	0	Conjuntivitis	1
2	06/09/2019	H16.0	1	Úlcera corneal	1
2	06/09/2019	H01.00	4	Blefaritis	1
2	06/09/2019	H10.81	0	Pingueculitis	1
1	06/09/2019	H16.0	1	Úlcera corneal	1
2	06/09/2019	H16.0	1	Úlcera corneal	1
1	07/09/2019	H16.9	1	Queratitis	1
1	07/09/2019	T15.0	1	Cuerpo extraño	1
1	07/09/2019	H16.9	1	Queratitis	1
2	07/09/2019	H15.1	6	Epiescleritis	1
2	07/09/2019	H16.0	1	Úlcera corneal	1
1	07/09/2019	T15.0	1	Cuerpo extraño	1
1	07/09/2019	T15.0	1	Cuerpo extraño	1
2	07/09/2019	H10.3	0	Conjuntivitis	1
2	07/09/2019	H00.02	4	Orzuelo	1
1	07/09/2019	H16.0	1	Úlcera corneal	1
1	07/09/2019	H10.3	0	Conjuntivitis	1
1	07/09/2019	T15.0	1	Cuerpo extraño	1
2	07/09/2019	H01.00	4	Blefaritis	1
2	07/09/2019	H10.3	0	Conjuntivitis	1
1	07/09/2019	H34.82	8	Trombosis venosa	1
1	07/09/2019	S05.30	9	Laceración conjuntival	1
1	07/09/2019	H11.3	0	Hiposfagma	1
1	07/09/2019	S05.9	9	Traumatismo	1
1	07/09/2019	H10.3	0	Conjuntivitis	1
2	07/09/2019	H00.03	4	Celulitis preseptal	1
2	07/09/2019	H10.3	0	Conjuntivitis	1
2	07/09/2019	H0.41	1	Ojo seco	1
2	07/09/2019	H00.02	4	Orzuelo	1
2	07/09/2019	H53.9	11	Alteraciones visuales	1
1	07/09/2019	S05.9	9	Traumatismo	1
2	07/09/2019	H11.3	0	Hiposfagma	1
2	07/09/2019	H11.44	0	Quiste conjuntival	1
2	07/09/2019	H01.00	4	Blefaritis	1
1	07/09/2019	H02	4	Alteración palpebral	1
2	07/09/2019	H43.81	7	DVP	1
1	07/09/2019	S05.9	9	Traumatismo	1
1	07/09/2019	H02.05	4	Distiquiasis	1
2	07/09/2019	T15.0	1	Cuerpo extraño	1
1	07/09/2019	S05.9	9	Traumatismo	1
2	07/09/2019	H16.0	1	Úlcera corneal	1
1	07/09/2019	B00.1	4	Dermatitis herpética	1
2	07/09/2019	H02.05	4	Distiquiasis	1
1	07/09/2019	S05.9	9	Traumatismo	1
1	07/09/2019	H33.30	8	Desgarro retiniano	1

2	07/09/2019	S05.9	9	Traumatismo	1
1	07/09/2019	H16.0	1	Úlcera corneal	1
2	07/09/2019	B00.1	4	Dermatitis herpética	1
2	07/09/2019	H33.30	8	Desgarro retiniano	1
2	07/09/2019	H16.0	1	Úlcera corneal	1
1	07/09/2019	H00.02	4	Orzuelo	1
2	07/09/2019	H10.3	0	Conjuntivitis	1
2	08/09/2019	H16.9	1	Queratitis	1
1	08/09/2019	H11.3	0	Hiposfagma	1
2	08/09/2019	H16.0	1	Úlcera corneal	1
2	08/09/2019	H10.3	0	Conjuntivitis	1
2	08/09/2019	H00.02	4	Orzuelo	1
1	08/09/2019	H47.10	10	Papiledema	1
2	08/09/2019	H00.03	4	Celulitis preseptal	1
1	08/09/2019	T15.0	1	Cuerpo extraño	1
2	08/09/2019	H10.3	0	Conjuntivitis	1
1	08/09/2019	T15.0	1	Cuerpo extraño	1
1	08/09/2019	H16.0	1	Úlcera corneal	1
2	08/09/2019	H43.81	7	DVP	1
2	08/09/2019	H10.3	0	Conjuntivitis	1
1	08/09/2019	H43.81	7	DVP	1
1	08/09/2019	H16.0	1	Úlcera corneal	1
1	08/09/2019	H53	11	No patología	1
2	08/09/2019	H16.0	1	Úlcera corneal	1
2	08/09/2019	H53	11	No patología	1
1	08/09/2019	H43.81	7	DVP	1
1	08/09/2019	H11.3	0	Hiposfagma	1
2	08/09/2019	H33.0	8	Desprendimiento de retina	1
2	08/09/2019	H16.9	1	Queratitis	1
1	08/09/2019	H02	4	Alteración palpebral	1
2	08/09/2019	H00.03	4	Celulitis preseptal	1
1	08/09/2019	H10.3	0	Conjuntivitis	1
2	08/09/2019	H10.3	0	Conjuntivitis	1
2	08/09/2019	H53.9	11	Alteraciones visuales	1
2	08/09/2019	H10.3	0	Conjuntivitis	1
2	08/09/2019	H53	11	No patología	1
2	08/09/2019	H53	11	No patología	1
2	08/09/2019	H53	11	No patología	1
2	08/09/2019	H16.9	1	Queratitis	1
1	08/09/2019	H16.0	1	Úlcera corneal	1
1	08/09/2019	H10.3	0	Conjuntivitis	1
2	08/09/2019	H43.81	7	DVP	1
2	08/09/2019	H16.0	1	Úlcera corneal	1
2	08/09/2019	H16.9	1	Queratitis	1
2	08/09/2019	H16.9	1	Queratitis	1
2	08/09/2019	H16.9	1	Queratitis	1
1	08/09/2019	H43.81	7	DVP	1
2	08/09/2019	H11.3	0	Hiposfagma	1
1	08/09/2019	H16.9	1	Queratitis	1
1	09/09/2019	H10.3	0	Conjuntivitis	1
2	09/09/2019	H10.3	0	Conjuntivitis	1
1	09/09/2019	H10.3	0	Conjuntivitis	1
2	09/09/2019	H16.9	1	Queratitis	1
1	09/09/2019	H00.02	4	Orzuelo	1
2	09/09/2019	H10.5	4	Blefaroconjuntivitis	1
2	09/09/2019	H35.3	8	Maculopatía	1
2	09/09/2019	H18.50	1	Distrofia corneal	1
1	09/09/2019	H01.00	4	Blefaritis	1
2	09/09/2019	H10.3	0	Conjuntivitis	1
2	09/09/2019	H16.9	1	Queratitis	1
1	09/09/2019	H53	11	No patología	1
2	09/09/2019	H00.02	4	Orzuelo	1
2	09/09/2019	H11.3	0	Hiposfagma	1
1	09/09/2019	H16.0	1	Úlcera corneal	1
1	09/09/2019	H01.00	4	Blefaritis	1
2	09/09/2019	H17.8	1	Infiltrados corneales	1

2	09/09/2019	H10.3	0	Conjuntivitis	1
1	09/09/2019	S05.9	9	Traumatismo	1
2	09/09/2019	H10.3	0	Conjuntivitis	1
1	09/09/2019	H10.3	0	Conjuntivitis	1
1	09/09/2019	H10.3	0	Conjuntivitis	1
2	09/09/2019	H10.3	0	Conjuntivitis	1
2	09/09/2019	H11.3	0	Hiposfagma	1
2	09/09/2019	H35.3	8	Maculopatía	1
2	09/09/2019	H44.009	8	Endoftalmitis	1
1	09/09/2019	H10.3	0	Conjuntivitis	1
2	09/09/2019	H16.9	1	Queratitis	1
2	09/09/2019	H0.41	1	Ojo seco	1
1	09/09/2019	H10.3	0	Conjuntivitis	1
2	09/09/2019	H44.009	8	Endoftalmitis	1
1	09/09/2019	H20	6	Uveitis	1
2	09/09/2019	H53	11	No patología	1
2	09/09/2019	H43.81	7	DVP	1
1	09/09/2019	G43.109	10	Aura	1
2	09/09/2019	H16.0	1	Úlcera corneal	1
1	09/09/2019	H10.3	0	Conjuntivitis	1
1	09/09/2019	H35.0	8	Hemorragia retiniana	1
1	09/09/2019	H00.02	4	Orzuelo	1
1	09/09/2019	H10.3	0	Conjuntivitis	1
1	09/09/2019	T15.0	1	Cuerpo extraño	1
2	09/09/2019	H53	11	No patología	1
2	09/09/2019	H43.81	7	DVP	1
1	09/09/2019	H16.9	1	Queratitis	1
2	09/09/2019	H16.0	1	Úlcera corneal	1
2	09/09/2019	H11.3	0	Hiposfagma	1
1	10/09/2019	H10.3	0	Conjuntivitis	1
1	10/09/2019	T15.0	1	Cuerpo extraño	1
1	10/09/2019	H10.3	0	Conjuntivitis	1
2	10/09/2019	H11.3	0	Hiposfagma	1
2	10/09/2019	H53	11	No patología	1
1	10/09/2019	H10.3	0	Conjuntivitis	1
1	10/09/2019	H00.02	4	Orzuelo	1
1	10/09/2019	H10.3	0	Conjuntivitis	1
2	10/09/2019	H0.41	1	Ojo seco	1
2	10/09/2019	H00.03	4	Celulitis preseptal	1
1	10/09/2019	H15.1	6	Epiescleritis	1
1	10/09/2019	H10.3	0	Conjuntivitis	1
2	10/09/2019	H16.0	1	Úlcera corneal	1
2	10/09/2019	H26.9	2	Catarata	1
2	10/09/2019	H53	11	No patología	1
2	10/09/2019	H27.8	2	OCP	1
2	10/09/2019	H10.3	0	Conjuntivitis	1
2	10/09/2019	G43.109	10	Aura	1
2	10/09/2019	H16.0	1	Úlcera corneal	1
2	10/09/2019	H11.44	0	Quiste conjuntival	1
2	10/09/2019	H00.02	4	Orzuelo	1
1	10/09/2019	H26.9	2	Catarata	1
1	10/09/2019	T26	9	Causticación	1
1	10/09/2019	H40.21	3	Glaucoma agudo	1
2	10/09/2019	G43.109	10	Aura	1
2	10/09/2019	H02	4	Lesión palpebral	1
2	10/09/2019	H02.05	4	Distiquiasis	1
2	10/09/2019	H16.9	1	Queratitis	1
1	10/09/2019	S05.9	9	Traumatismo	1
1	10/09/2019	H53	11	No patología	1
2	10/09/2019	H27.8	2	OCP	1
2	10/09/2019	H43.81	7	DVP	1
2	10/09/2019	T15.0	1	Cuerpo extraño	1
2	10/09/2019	H43.81	7	DVP	1
2	10/09/2019	H11.3	0	Hiposfagma	1
1	10/09/2019	H20	6	Uveitis	1
2	10/09/2019	H53	11	No patología	1

2	10/09/2019	H16.0	1	Úlcera corneal	1
2	10/09/2019	H0.41	1	Ojo seco	1
1	10/09/2019	H20	6	Uveitis	1
2	10/09/2019	H16.0	1	Úlcera corneal	1
1	10/09/2019	S05.9	9	Traumatismo	1
2	10/09/2019	H16.0	1	Úlcera corneal	1
1	10/09/2019	H15.1	6	Epiescleritis	1
1	10/09/2019	H43.81	7	DVP	1
2	10/09/2019	H0.41	1	Ojo seco	1
2	10/09/2019	H10.3	0	Conjuntivitis	1
1	10/09/2019	H16.9	1	Queratitis	1
1	10/09/2019	H16.0	1	Úlcera corneal	1
2	10/09/2019	S05.6	9	Perforación ocular	1
2	10/09/2019	H16.0	1	Úlcera corneal	1
1	10/09/2019	H34.9	8	Oclusión arterial	1
2	10/09/2019	S05.9	9	Traumatismo	1
2	11/09/2019	H16.9	1	Queratitis	1
1	11/09/2019	H16.9	1	Queratitis	1
1	11/09/2019	T15.0	1	Cuerpo extraño	1
2	11/09/2019	H16.0	1	Úlcera corneal	1
1	11/09/2019	H35.3	8	Maculopatía	1
2	11/09/2019	H00.02	4	Orzuelo	1
1	11/09/2019	H11.3	0	Hiposfagma	1
2	11/09/2019	H0.41	1	Ojo seco	1
2	11/09/2019	H16.9	1	Queratitis	1
2	11/09/2019	H16.9	1	Queratitis	1
2	11/09/2019	H16.9	1	Queratitis	1
2	11/09/2019	H00.02	4	Orzuelo	1
1	11/09/2019	H10.3	0	Conjuntivitis	1
2	11/09/2019	H10.3	0	Conjuntivitis	1
2	11/09/2019	H26.9	2	Catarata	1
2	11/09/2019	H43.81	7	DVP	1
1	11/09/2019	T15.0	1	Cuerpo extraño	1
1	11/09/2019	H10.3	0	Conjuntivitis	1
2	11/09/2019	H27.8	2	OCP	1
1	11/09/2019	H59.3	4	Post op párpados	1
2	11/09/2019	H44.009	8	Endoftalmitis	1
1	11/09/2019	H20	6	Uveitis	1
2	11/09/2019	H00.02	4	Orzuelo	1
2	11/09/2019	H53	11	No patología	1
2	11/09/2019	H15.00	6	Escleritis	1
2	11/09/2019	H15.00	6	Escleritis	1
2	11/09/2019	H33.30	8	Desgarro retiniano	1
1	11/09/2019	T15.0	1	Cuerpo extraño	1
2	11/09/2019	H01.00	4	Blefaritis	1
1	11/09/2019	T15.0	1	Cuerpo extraño	1
1	11/09/2019	H59.88	8	Post op retina	1
1	11/09/2019	H34.9	8	Oclusión arterial	1
1	11/09/2019	T15.0	1	Cuerpo extraño	1
2	11/09/2019	T15.0	1	Cuerpo extraño	1
2	11/09/2019	H16.0	1	Úlcera corneal	1
2	11/09/2019	H53	11	No patología	1
2	11/09/2019	H15.1	6	Epiescleritis	1
1	11/09/2019	H34.82	8	Trombosis venosa	1
1	11/09/2019	H33.30	8	Desgarro retiniano	1
2	11/09/2019	H15.00	6	Escleritis	1
2	11/09/2019	T15.0	1	Cuerpo extraño	1
2	11/09/2019	H01.00	4	Blefaritis	1
1	11/09/2019	H10.3	0	Conjuntivitis	1
1	11/09/2019	S05.9	9	Traumatismo	1
1	11/09/2019	H16.9	1	Queratitis	1
2	11/09/2019	H11.3	0	Hiposfagma	1
1	11/09/2019	S05.9	9	Traumatismo	1
2	11/09/2019	H16.9	1	Queratitis	1
2	11/09/2019	H11.3	0	Hiposfagma	1
1	11/09/2019	H10.3	0	Conjuntivitis	1

1	11/09/2019	H10.3	0	Conjuntivitis	1
2	11/09/2019	H11.3	0	Hiposfagma	1
1	11/09/2019	H16.9	1	Queratitis	1
2	11/09/2019	Alta por fuga	11	Alta por fuga	1
1	11/09/2019	T15.0	1	Cuerpo extraño	1
1	12/09/2019	H16.9	1	Queratitis	1
1	12/09/2019	H16.0	1	Úlcera corneal	1
2	12/09/2019	H16.9	1	Queratitis	1
1	12/09/2019	H00.02	4	Orzuelo	1
1	12/09/2019	H16.9	1	Queratitis	1
1	12/09/2019	H01.00	4	Blefaritis	1
1	12/09/2019	H35.3	8	Maculopatía	1
1	12/09/2019	H10.3	0	Conjuntivitis	1
1	12/09/2019	H16.0	1	Úlcera corneal	1
1	12/09/2019	H16.0	1	Úlcera corneal	1
1	12/09/2019	H10.3	0	Conjuntivitis	1
1	12/09/2019	H53	11	No patología	1
2	12/09/2019	H00.02	4	Orzuelo	1
2	12/09/2019	H16.9	1	Queratitis	1
1	12/09/2019	H10.3	0	Conjuntivitis	1
1	12/09/2019	H10.3	0	Conjuntivitis	1
1	12/09/2019	H16.0	1	Úlcera corneal	1
2	12/09/2019	H53	11	No patología	1
2	12/09/2019	H11.3	0	Hiposfagma	1
1	12/09/2019	H10.3	0	Conjuntivitis	1
2	12/09/2019	H43.81	7	DVP	1
1	12/09/2019	H18.20	1	Edema corneal	1
2	12/09/2019	H33.30	8	Desgarro retiniano	1
2	12/09/2019	H01.00	4	Blefaritis	1
2	12/09/2019	H11.3	0	Hiposfagma	1
1	12/09/2019	H53	11	No patología	1
2	12/09/2019	H16.9	1	Queratitis	1
1	12/09/2019	H33.0	8	Desprendimiento de retina	1
2	12/09/2019	H10.3	0	Conjuntivitis	1
1	12/09/2019	H16.0	1	Úlcera corneal	1
2	12/09/2019	H02	4	Lesión palpebral	1
1	12/09/2019	H16.0	1	Úlcera corneal	1
2	12/09/2019	H10.3	0	Conjuntivitis	1
1	12/09/2019	T15.0	1	Cuerpo extraño	1
2	12/09/2019	H10.5	4	Blefaroconjuntivitis	1
1	12/09/2019	H10.3	0	Conjuntivitis	1
1	12/09/2019	H10.3	0	Conjuntivitis	1
1	12/09/2019	H31.42	8	Coroidopatía serosa central	1
2	12/09/2019	S05.9	9	Traumatismo	1
2	12/09/2019	H16.9	1	Queratitis	1
1	12/09/2019	H10.3	0	Conjuntivitis	1
1	12/09/2019	S05.9	9	Traumatismo	1
2	12/09/2019	H16.9	1	Queratitis	1
2	12/09/2019	H43.1	7	Hemovítreo	1
1	12/09/2019	H11.3	0	Hiposfagma	1
1	12/09/2019	H16.0	1	Úlcera corneal	1
1	12/09/2019	H15.00	6	Escleritis	1
1	12/09/2019	H16.0	1	Úlcera corneal	1
2	12/09/2019	H43.81	7	DVP	1
1	12/09/2019	H00.02	4	Orzuelo	1
2	12/09/2019	T15.0	1	Cuerpo extraño	1
2	12/09/2019	H16.0	1	Úlcera corneal	1
2	12/09/2019	H20	6	Uveitis	1
2	12/09/2019	H53	11	No patología	1
1	12/09/2019	H16.0	1	Úlcera corneal	1
1	13/09/2019	H10.3	0	Conjuntivitis	1
1	13/09/2019	H16.0	1	Úlcera corneal	1
1	13/09/2019	H16.0	1	Úlcera corneal	1
2	13/09/2019	H01.00	4	Blefaritis	1
1	13/09/2019	H10.3	0	Conjuntivitis	1
2	13/09/2019	H10.5	4	Blefaroconjuntivitis	1

2	13/09/2019	H43.81	7	DVP	1
2	13/09/2019	H16.9	1	Queratitis	1
2	13/09/2019	H16.0	1	Úlcera corneal	1
2	13/09/2019	H16.0	1	Úlcera corneal	1
1	13/09/2019	H59.3	4	Post op párpados	1
2	13/09/2019	H11.44	0	Quiste conjuntival	1
1	13/09/2019	H16.9	1	Queratitis	1
1	13/09/2019	H02	4	Lesión palpebral	1
1	13/09/2019	H16.0	1	Úlcera corneal	1
1	13/09/2019	H43.81	7	DVP	1
2	13/09/2019	H10.3	0	Conjuntivitis	1
2	13/09/2019	H16.0	1	Úlcera corneal	1
1	13/09/2019	H20	6	Uveitis	1
1	13/09/2019	H10.3	0	Conjuntivitis	1
1	13/09/2019	H16.9	1	Queratitis	1
1	13/09/2019	H20	6	Uveitis	1
2	13/09/2019	S05.30	9	Laceración conjuntival	1
2	13/09/2019	H44.009	8	Endoftalmitis	1
1	13/09/2019	H43.1	7	Hemovítreo	1
1	13/09/2019	H43.81	7	DVP	1
2	13/09/2019	H10.3	0	Conjuntivitis	1
2	13/09/2019	H16.0	1	Úlcera corneal	1
2	13/09/2019	H15.1	6	Epiescleritis	1
1	13/09/2019	T15.0	1	Cuerpo extraño	1
2	13/09/2019	H0.41	1	Ojo seco	1
2	13/09/2019	H53	11	No patología	1
1	13/09/2019	H16.0	1	Úlcera corneal	1
1	13/09/2019	H27.8	2	OCP	1
2	13/09/2019	H15.1	6	Epiescleritis	1
1	13/09/2019	H26.9	2	Catarata	1
2	13/09/2019	H11.3	0	Hiposfagma	1
1	13/09/2019	H10.3	0	Conjuntivitis	1
1	13/09/2019	H00.02	4	Orzuelo	1
2	13/09/2019	H47.32	10	Drusas de nervio óptico	1
2	13/09/2019	H53	11	No patología	1
2	13/09/2019	H01.00	4	Blefaritis	1
1	13/09/2019	H43.81	7	DVP	1
2	13/09/2019	H35.3	8	Maculopatía	1
2	13/09/2019	H43.81	7	DVP	1
2	13/09/2019	H11.00	0	Pterigium	1
2	13/09/2019	H16.0	1	Úlcera corneal	1
2	13/09/2019	H11.3	0	Hiposfagma	1
2	13/09/2019	H33.30	8	Desgarro retiniano	1
2	13/09/2019	H11.3	0	Hiposfagma	1
1	13/09/2019	H10.3	0	Conjuntivitis	1
2	13/09/2019	H43.81	7	DVP	1
1	13/09/2019	H02	4	Alteración palpebral	1
1	13/09/2019	T15.0	1	Cuerpo extraño	1
1	13/09/2019	H01.00	4	Blefaritis	1
1	13/09/2019	H16.0	1	Úlcera corneal	1
1	13/09/2019	T15.0	1	Cuerpo extraño	1
1	13/09/2019	T15.0	1	Cuerpo extraño	1
1	13/09/2019	S05.30	9	Laceración conjuntival	1
2	13/09/2019	H01.00	4	Blefaritis	1
1	13/09/2019	H16.0	1	Úlcera corneal	1
1	14/09/2019	H16.0	1	Úlcera corneal	1
1	14/09/2019	S05.9	9	Traumatismo	1
1	14/09/2019	S05.9	9	Traumatismo	1
1	14/09/2019	T15.0	1	Cuerpo extraño	1
2	14/09/2019	H53	11	No patología	1
1	14/09/2019	T15.0	1	Cuerpo extraño	1
1	14/09/2019	H10.3	0	Conjuntivitis	1
2	14/09/2019	H11.3	0	Hiposfagma	1
2	14/09/2019	H04.32	5	Dacriocistitis aguda	1
1	14/09/2019	H49.9	10	Parálisis oculomotora	1
2	14/09/2019	H16.9	1	Queratitis	1

1	14/09/2019	H10.3	0	Conjuntivitis	1
2	14/09/2019	H04.32	5	Dacriocistitis aguda	1
2	14/09/2019	H10.3	0	Conjuntivitis	1
2	14/09/2019	H16.0	1	Úlcera corneal	1
1	14/09/2019	T15.0	1	Cuerpo extraño	1
2	14/09/2019	H17.8	1	Infiltrados corneales	1
1	14/09/2019	H10.3	0	Conjuntivitis	1
1	14/09/2019	T15.0	1	Cuerpo extraño	1
2	14/09/2019	H53.10	11	Problema refractivo	1
2	14/09/2019	H10.3	0	Conjuntivitis	1
1	14/09/2019	H10.3	0	Conjuntivitis	1
1	14/09/2019	H11.44	0	Quiste conjuntival	1
1	14/09/2019	H10.3	0	Conjuntivitis	1
2	14/09/2019	H00.02	4	Orzuelo	1
1	14/09/2019	H10.3	0	Conjuntivitis	1
1	14/09/2019	H16.0	1	Úlcera corneal	1
2	14/09/2019	H11.3	0	Hiposfagma	1
1	14/09/2019	H20	6	Uveitis	1
1	14/09/2019	H00.02	4	Orzuelo	1
2	14/09/2019	H16.0	1	Úlcera corneal	1
2	14/09/2019	H00.02	4	Orzuelo	1
2	14/09/2019	S05.00	9	Abrasión conjuntival	1
1	14/09/2019	H00.02	4	Orzuelo	1
2	14/09/2019	H10.3	0	Conjuntivitis	1
1	14/09/2019	H10.3	0	Conjuntivitis	1
1	14/09/2019	S05.30	9	Laceración conjuntival	1
1	14/09/2019	H16.0	1	Úlcera corneal	1
2	14/09/2019	H10.3	0	Conjuntivitis	1
1	14/09/2019	H10.3	0	Conjuntivitis	1
2	14/09/2019	H20	6	Uveitis	1
2	14/09/2019	H10.3	0	Conjuntivitis	1
2	14/09/2019	H16.9	1	Queratitis	1
1	14/09/2019	H11.3	0	Hiposfagma	1
1	14/09/2019	H16.9	1	Queratitis	1
2	14/09/2019	H17.8	1	Infiltrados corneales	1
1	14/09/2019	H00.02	4	Orzuelo	1
2	14/09/2019	H53.9	11	Alteraciones visuales	1
1	14/09/2019	B00.1	4	Dermatitis herpética	1
1	14/09/2019	H16.0	1	Úlcera corneal	1
2	14/09/2019	H16.0	1	Úlcera corneal	1
2	14/09/2019	S05.9	9	Traumatismo	1
2	15/09/2019	H02	4	Lesión palpebral	1
1	15/09/2019	S05.9	9	Traumatismo	1
2	15/09/2019	S05.9	9	Traumatismo	1
1	15/09/2019	H20	6	Uveitis	1
2	15/09/2019	S05.9	9	Traumatismo	1
2	15/09/2019	H11.3	0	Hiposfagma	1
2	15/09/2019	H10.3	0	Conjuntivitis	1
1	15/09/2019	H16.0	1	Úlcera corneal	1
1	15/09/2019	H16.0	1	Úlcera corneal	1
1	15/09/2019	H10.3	0	Conjuntivitis	1
2	15/09/2019	S05.9	9	Traumatismo	1
2	15/09/2019	T15.0	1	Cuerpo extraño	1
1	15/09/2019	S05.9	9	Traumatismo	1
2	15/09/2019	H16.0	1	Úlcera corneal	1
1	15/09/2019	H10.3	0	Conjuntivitis	1
2	15/09/2019	H10.3	0	Conjuntivitis	1
2	15/09/2019	H02.05	4	Distiquiasis	1
1	15/09/2019	H00.02	4	Orzuelo	1
2	15/09/2019	T15.0	1	Cuerpo extraño	1
1	15/09/2019	H00.02	4	Orzuelo	1
1	15/09/2019	H10.3	0	Conjuntivitis	1
2	15/09/2019	H16.0	1	Úlcera corneal	1
2	15/09/2019	H15.00	6	Escleritis	1
1	15/09/2019	H16.0	1	Úlcera corneal	1
2	15/09/2019	H16.9	1	Queratitis	1

1	15/09/2019	T15.0	1	Cuerpo extraño	1
2	15/09/2019	H16.9	1	Queratitis	1
1	15/09/2019	H00.03	4	Celulitis preseptal	1
2	15/09/2019	H17.8	1	Infiltrados corneales	1
2	15/09/2019	H16.0	1	Úlcera corneal	1
2	15/09/2019	H26.9	2	Catarata	1
1	15/09/2019	H10.3	0	Conjuntivitis	1
2	15/09/2019	H10.3	0	Conjuntivitis	1
1	15/09/2019	H10.3	0	Conjuntivitis	1
1	15/09/2019	H10.3	0	Conjuntivitis	1
1	15/09/2019	H10.3	0	Conjuntivitis	1
1	15/09/2019	H10.3	0	Conjuntivitis	1
1	15/09/2019	H35.3	8	Maculopatía	1
1	15/09/2019	T15.0	1	Cuerpo extraño	1
1	15/09/2019	H20	6	Uveitis	1
2	15/09/2019	H15.1	6	Epiescleritis	1
1	15/09/2019	S05.6	9	Perforación ocular	1
2	15/09/2019	H11.3	0	Hiposfagma	1
1	15/09/2019	T15.0	1	Cuerpo extraño	1
2	15/09/2019	S05.9	9	Traumatismo	1
2	15/09/2019	H10.3	0	Conjuntivitis	1
1	15/09/2019	H53	11	No patología	1
1	15/09/2019	H10.3	0	Conjuntivitis	1
1	15/09/2019	H10.3	0	Conjuntivitis	1
1	15/09/2019	H10.3	0	Conjuntivitis	1
2	15/09/2019	Alta por fuga	11	Alta por fuga	1
1	15/09/2019	Alta por fuga	11	Alta por fuga	1
2	15/09/2019	H16.0	1	Úlcera corneal	1
2	15/09/2019	S05.9	9	Traumatismo	1
2	16/09/2019	H11.3	0	Hiposfagma	1
2	16/09/2019	H00.02	4	Orzuelo	1
1	16/09/2019	H0.41	1	Ojo seco	1
1	16/09/2019	H00.02	4	Orzuelo	1
1	16/09/2019	H43.1	7	Hemovítreo	1
2	16/09/2019	H16.9	1	Queratitis	1
2	16/09/2019	H10.3	0	Conjuntivitis	1
2	16/09/2019	E11.319	8	Retinopatía diabética	1
1	16/09/2019	T15.0	1	Cuerpo extraño	1
2	16/09/2019	H16.0	1	Úlcera corneal	1
2	16/09/2019	H27.8	2	OCP	1
2	16/09/2019	H16.9	1	Queratitis	1
2	16/09/2019	H10.3	0	Conjuntivitis	1
2	16/09/2019	H16.9	1	Queratitis	1
1	16/09/2019	Alta por fuga	11	Alta por fuga	1
2	16/09/2019	H10.3	0	Conjuntivitis	1
2	16/09/2019	H43.81	7	DVP	1
2	16/09/2019	H01.00	4	Blefaritis	1
2	16/09/2019	H11.3	0	Hiposfagma	1
2	16/09/2019	H16.9	1	Queratitis	1
1	16/09/2019	H16.9	1	Queratitis	1
2	16/09/2019	H01.00	4	Blefaritis	1
2	16/09/2019	H16.0	1	Úlcera corneal	1
2	16/09/2019	H04.32	5	Dacriocistitis aguda	1
2	16/09/2019	H11.3	0	Hiposfagma	1
1	16/09/2019	H16.0	1	Úlcera corneal	1
1	16/09/2019	H53.9	11	Alteraciones visuales	1
1	16/09/2019	H27.8	2	OCP	1
2	16/09/2019	H16.9	1	Queratitis	1
2	16/09/2019	H35.0	8	Hemorragia retiniana	1
2	16/09/2019	H16.0	1	Úlcera corneal	1
2	16/09/2019	H16.9	1	Queratitis	1
1	16/09/2019	H11.3	0	Hiposfagma	1
2	16/09/2019	H27.8	2	OCP	1
2	16/09/2019	H35.30	8	DMAE	1
1	16/09/2019	H01.00	4	Blefaritis	1
2	16/09/2019	H27.8	2	OCP	1

2	16/09/2019	H16.9	1	Queratitis	1
2	16/09/2019	H16.0	1	Úlcera corneal	1
1	16/09/2019	H16.9	1	Queratitis	1
2	16/09/2019	H10.3	0	Conjuntivitis	1
2	16/09/2019	H10.3	0	Conjuntivitis	1
2	16/09/2019	H16.9	1	Queratitis	1
1	16/09/2019	H10.3	0	Conjuntivitis	1
1	16/09/2019	H10.3	0	Conjuntivitis	1
2	16/09/2019	T15.0	1	Cuerpo extraño	1
1	16/09/2019	H43.81	7	DVP	1
1	16/09/2019	H59.3	4	Post op párpados	1
2	16/09/2019	H43.81	7	DVP	1
2	16/09/2019	H11.3	0	Hiposfagma	1
2	16/09/2019	H10.3	0	Conjuntivitis	1
1	16/09/2019	H16.0	1	Úlcera corneal	1
1	16/09/2019	H26.9	2	Catarata	1
1	16/09/2019	H11.3	0	Hiposfagma	1
2	16/09/2019	H04.32	5	Dacriocistitis aguda	1
1	16/09/2019	H10.3	0	Conjuntivitis	1
2	17/09/2019	H16.9	1	Queratitis	1
2	17/09/2019	H53.10	11	Problema refractivo	1
2	17/09/2019	H17.8	1	Infiltrados corneales	1
2	17/09/2019	H10.3	0	Conjuntivitis	1
2	17/09/2019	H10.81	4	Pingueculitis	1
2	17/09/2019	H16.9	1	Queratitis	1
2	17/09/2019	H16.9	1	Queratitis	1
1	17/09/2019	H00.02	4	Orzuelo	1
2	17/09/2019	S05.30	9	Laceración conjuntival	1
1	17/09/2019	H10.3	0	Conjuntivitis	1
2	17/09/2019	H20	6	Uveitis	1
2	17/09/2019	H16.0	1	Úlcera corneal	1
1	17/09/2019	H20	6	Uveitis	1
2	17/09/2019	H0.41	1	Ojo seco	1
1	17/09/2019	H16.9	1	Queratitis	1
2	17/09/2019	H35.3	8	Maculopatía	1
1	17/09/2019	H00.02	4	Orzuelo	1
1	17/09/2019	H53.9	11	Alteraciones visuales	1
2	17/09/2019	H43.81	7	DVP	1
1	17/09/2019	T15.0	1	Cuerpo extraño	1
1	17/09/2019	H33.0	8	Desprendimiento de retina	1
1	17/09/2019	H01.00	4	Blefaritis	1
2	17/09/2019	H43.81	7	DVP	1
2	17/09/2019	H16.9	1	Queratitis	1
2	17/09/2019	H27.8	7	OCP	1
1	17/09/2019	H01.00	4	Blefaritis	1
2	17/09/2019	H10.3	0	Conjuntivitis	1
2	17/09/2019	H40.9	3	Glaucoma	1
1	17/09/2019	H16.9	1	Queratitis	1
2	17/09/2019	H10.3	0	Conjuntivitis	1
1	17/09/2019	H17.8	1	Infiltrados corneales	1
1	17/09/2019	H17.8	1	Infiltrados corneales	1
1	17/09/2019	H16.9	1	Queratitis	1
2	17/09/2019	H10.3	0	Conjuntivitis	1
1	17/09/2019	H16.0	1	Úlcera corneal	1
2	17/09/2019	H10.3	0	Conjuntivitis	1
1	17/09/2019	T15.0	1	Cuerpo extraño	1
1	17/09/2019	H10.3	0	Conjuntivitis	1
2	17/09/2019	H15.1	6	Epiescleritis	1
1	17/09/2019	H16.9	1	Queratitis	1
2	18/09/2019	H16.0	1	Úlcera corneal	1
2	18/09/2019	H16.9	1	Queratitis	1
2	18/09/2019	H00.02	4	Orzuelo	1
1	18/09/2019	H27.8	2	OCP	1
2	18/09/2019	H10.3	0	Conjuntivitis	1
2	18/09/2019	H15.1	6	Epiescleritis	1
2	18/09/2019	H15.00	6	Escleritis	1

1	18/09/2019	H35.30	8	DMAE	1
1	18/09/2019	H10.3	0	Conjuntivitis	1
2	18/09/2019	H10.3	0	Conjuntivitis	1
2	18/09/2019	H15.00	6	Escleritis	1
2	18/09/2019	H16.9	1	Queratitis	1
1	18/09/2019	T15.0	1	Cuerpo extraño	1
1	18/09/2019	H26.9	2	Catarata	1
1	18/09/2019	H34.82	8	Trombosis venosa	1
2	18/09/2019	H10.3	0	Conjuntivitis	1
2	18/09/2019	Alta por fuga	11	Alta por fuga	1
2	18/09/2019	H16.9	1	Queratitis	1
2	18/09/2019	H16.9	1	Queratitis	1
1	18/09/2019	H11.3	0	Hiposfagma	1
2	18/09/2019	H43.81	7	DVP	1
2	18/09/2019	H01.00	4	Blefaritis	1
1	18/09/2019	Alta por fuga	11	Alta por fuga	1
1	18/09/2019	Alta por fuga	11	Alta por fuga	1
2	18/09/2019	Alta por fuga	11	Alta por fuga	1
2	18/09/2019	H16.9	1	Queratitis	1
1	18/09/2019	H10.3	0	Conjuntivitis	1
2	18/09/2019	H34.82	8	Trombosis venosa	1
1	18/09/2019	H35.3	8	Maculopatía	1
1	18/09/2019	H10.3	0	Conjuntivitis	1
2	18/09/2019	H00.02	4	Orzuelo	1
1	18/09/2019	H10.3	0	Conjuntivitis	1
2	18/09/2019	H43.81	7	DVP	1
1	18/09/2019	H35.30	8	DMAE	1
1	18/09/2019	H10.3	0	Conjuntivitis	1
2	18/09/2019	Z98.4	2	Post op catarata	1
2	18/09/2019	H16.9	1	Queratitis	1
1	18/09/2019	S05.9	9	Traumatismo	1
2	18/09/2019	H11.00	0	Pterigium	1
2	18/09/2019	H35.3	8	Maculopatía	1
1	18/09/2019	S05.9	9	Traumatismo	1
2	18/09/2019	H16.9	1	Queratitis	1
2	18/09/2019	S05.9	9	Traumatismo	1
2	18/09/2019	H10.3	0	Conjuntivitis	1
2	18/09/2019	H11.3	0	Hiposfagma	1
1	18/09/2019	T15.0	1	Cuerpo extraño	1
2	18/09/2019	H53	11	No patología	1
2	18/09/2019	H10.3	0	Conjuntivitis	1
1	18/09/2019	H53	11	No patología	1
1	18/09/2019	S05.9	9	Traumatismo	1
2	18/09/2019	H16.9	1	Queratitis	1
2	18/09/2019	T15.0	1	Cuerpo extraño	1
2	18/09/2019	H16.9	1	Queratitis	1
1	18/09/2019	H15.1	6	Epiescleritis	1
2	18/09/2019	H00.19	4	Chalazión	1
1	18/09/2019	H16.9	1	Queratitis	1
1	19/09/2019	H10.3	0	Conjuntivitis	1
1	19/09/2019	B00.1	4	Dermatitis herpética	1
2	19/09/2019	H11.3	0	Hiposfagma	1
2	19/09/2019	H27.8	2	OCP	1
1	19/09/2019	H35.30	8	DMAE	1
2	19/09/2019	H20	6	Uveitis	1
1	19/09/2019	H27.8	2	OCP	1
2	19/09/2019	H27.8	2	OCP	1
2	19/09/2019	H00.02	4	Orzuelo	1
1	19/09/2019	H16.0	1	Úlcera corneal	1
2	19/09/2019	H01.00	4	Blefaritis	1
2	19/09/2019	H00.02	4	Orzuelo	1
2	19/09/2019	H53	11	No patología	1
2	19/09/2019	H0.41	1	Ojo seco	1
2	19/09/2019	Z98.4	2	Post op catarata	1
2	19/09/2019	G43.9	10	Migraña	1
2	19/09/2019	H59.3	5	Postop via lagrimal	1

2	19/09/2019	H11.3	0	Hiposfagma	1
1	19/09/2019	H43.81	7	DVP	1
2	19/09/2019	H26.9	2	Catarata	1
1	19/09/2019	H16.9	1	Queratitis	1
1	19/09/2019	H11.3	0	Hiposfagma	1
2	19/09/2019	T15.0	1	Cuerpo extraño	1
2	19/09/2019	H04.32	5	Dacriocistitis aguda	1
2	19/09/2019	H00.02	4	Orzuelo	1
2	19/09/2019	S05.30	9	Laceración conjuntival	1
1	19/09/2019	H15.00	6	Escleritis	1
2	19/09/2019	H33.30	8	Desgarro retiniano	1
2	19/09/2019	H16.0	1	Úlcera corneal	1
2	19/09/2019	H10.3	0	Conjuntivitis	1
1	19/09/2019	H11.3	0	Hiposfagma	1
1	19/09/2019	T15.0	1	Cuerpo extraño	1
2	19/09/2019	H16.0	1	Úlcera corneal	1
2	19/09/2019	H01.00	4	Blefaritis	1
2	19/09/2019	Alta por fuga	11	Alta por fuga	1
1	20/09/2019	H10.3	0	Conjuntivitis	1
1	20/09/2019	T15.0	1	Cuerpo extraño	1
1	20/09/2019	H43.81	7	DVP	1
2	20/09/2019	H10.5	4	Blefaroconjuntivitis	1
1	20/09/2019	T15.0	1	Cuerpo extraño	1
2	20/09/2019	H16.9	1	Queratitis	1
1	20/09/2019	H53	11	No patología	1
1	20/09/2019	H10.3	0	Conjuntivitis	1
2	20/09/2019	H16.9	1	Queratitis	1
2	20/09/2019	H35.3	8	Maculopatía	1
2	20/09/2019	H00.02	4	Orzuelo	1
1	20/09/2019	H15.1	6	Epiescleritis	1
2	20/09/2019	H16.9	1	Queratitis	1
1	20/09/2019	H00.02	4	Orzuelo	1
2	20/09/2019	H16.0	1	Úlcera corneal	1
2	20/09/2019	H16.9	1	Queratitis	1
2	20/09/2019	G43.109	10	Aura	1
2	20/09/2019	H17.8	1	Infiltrados corneales	1
2	20/09/2019	H43.81	7	DVP	1
2	20/09/2019	H16.9	1	Queratitis	1
2	20/09/2019	H16.9	1	Queratitis	1
1	20/09/2019	H16.9	1	Queratitis	1
2	20/09/2019	H0.41	1	Ojo seco	1
2	20/09/2019	H20	6	Uveitis	1
1	20/09/2019	H10.3	0	Conjuntivitis	1
2	20/09/2019	H11.3	0	Hiposfagma	1
1	20/09/2019	H33.0	8	Desprendimiento de retina	1
1	20/09/2019	S05.30	9	Laceración conjuntival	1
2	20/09/2019	H16.9	1	Queratitis	1
1	20/09/2019	H16.9	1	Queratitis	1
2	20/09/2019	H16.9	1	Queratitis	1
2	20/09/2019	H10.3	0	Conjuntivitis	1
2	20/09/2019	H10.3	0	Conjuntivitis	1
2	20/09/2019	H10.3	0	Conjuntivitis	1
2	20/09/2019	S05.30	9	Laceración conjuntival	1
2	20/09/2019	H10.3	0	Conjuntivitis	1
2	20/09/2019	H33.30	8	Desgarro retiniano	1
2	20/09/2019	H43.81	7	DVP	1
2	20/09/2019	H10.5	4	Blefaroconjuntivitis	1
2	20/09/2019	H16.0	1	Úlcera corneal	1
2	20/09/2019	H43.81	7	DVP	1
1	20/09/2019	H43.1	7	Hemovítreo	1
2	20/09/2019	H16.9	1	Queratitis	1
2	20/09/2019	H02	4	Alteración palpebral	1
2	20/09/2019	H10.3	0	Conjuntivitis	1
2	20/09/2019	H10.3	0	Conjuntivitis	1
2	20/09/2019	H10.3	0	Conjuntivitis	1
2	20/09/2019	H10.3	0	Conjuntivitis	1

2	20/09/2019	H00.02	4	Orzuelo	1
1	20/09/2019	T15.0	1	Cuerpo extraño	1
1	20/09/2019	T15.0	1	Cuerpo extraño	1
2	21/09/2019	H16.9	1	Queratitis	1
2	21/09/2019	H04.41	5	Dacriocistitis crónica	1
1	21/09/2019	H16.0	1	Úlcera corneal	1
1	21/09/2019	H16.0	1	Úlcera corneal	1
2	21/09/2019	H53.9	11	Alteraciones visuales	1
2	21/09/2019	H16.9	1	Queratitis	1
2	21/09/2019	H10.3	0	Conjuntivitis	1
2	21/09/2019	H10.3	0	Conjuntivitis	1
2	21/09/2019	H16.9	1	Queratitis	1
2	21/09/2019	H10.3	0	Conjuntivitis	1
2	21/09/2019	H02	4	Lesión palpebral	1
1	21/09/2019	H10.3	0	Conjuntivitis	1
2	21/09/2019	H16.9	1	Queratitis	1
1	21/09/2019	S05.9	9	Traumatismo	1
2	21/09/2019	H10.3	0	Conjuntivitis	1
2	21/09/2019	H16.9	1	Queratitis	1
2	21/09/2019	H16.9	1	Queratitis	1
1	21/09/2019	T26	9	Causticación	1
2	21/09/2019	T15.0	1	Cuerpo extraño	1
2	21/09/2019	H10.3	0	Conjuntivitis	1
1	21/09/2019	T15.0	1	Cuerpo extraño	1
1	21/09/2019	H16.0	1	Úlcera corneal	1
2	21/09/2019	H43.81	7	DVP	1
2	21/09/2019	H16.0	1	Úlcera corneal	1
1	21/09/2019	H10.3	0	Conjuntivitis	1
1	21/09/2019	H00.02	4	Orzuelo	1
2	21/09/2019	H01.00	4	Blefaritis	1
2	21/09/2019	H16.0	1	Úlcera corneal	1
1	21/09/2019	H34.9	8	Oclusión arterial	1
1	21/09/2019	H16.9	1	Queratitis	1
2	21/09/2019	H27.8	2	OCP	1
1	21/09/2019	H01.00	4	Blefaritis	1
2	21/09/2019	H59.3	4	Post op párpados	1
2	21/09/2019	T15.0	1	Cuerpo extraño	1
1	21/09/2019	H16.9	1	Queratitis	1
2	21/09/2019	H16.9	1	Queratitis	1
1	21/09/2019	H10.3	0	Conjuntivitis	1
1	21/09/2019	T15.0	1	Cuerpo extraño	1
1	21/09/2019	H16.9	1	Queratitis	1
2	21/09/2019	H16.9	1	Queratitis	1
1	21/09/2019	H02.05	4	Distiquiasis	1
1	21/09/2019	H16.0	1	Úlcera corneal	1
1	22/09/2019	H16.9	1	Queratitis	1
1	22/09/2019	H10.3	0	Conjuntivitis	1
2	22/09/2019	H16.9	1	Queratitis	1
1	22/09/2019	H10.3	0	Conjuntivitis	1
1	22/09/2019	H40.05	3	HTO	1
2	22/09/2019	H10.3	0	Conjuntivitis	1
1	22/09/2019	H16.9	1	Queratitis	1
2	22/09/2019	H16.9	1	Queratitis	1
2	22/09/2019	H16.9	1	Queratitis	1
1	22/09/2019	H59.88	8	Post op retina	1
1	22/09/2019	H20	6	Uveitis	1
1	22/09/2019	H10.3	0	Conjuntivitis	1
2	22/09/2019	H10.3	0	Conjuntivitis	1
1	22/09/2019	H53.9	11	Alteraciones visuales	1
2	22/09/2019	H53	11	No patología	1
2	22/09/2019	H00.02	4	Orzuelo	1
2	22/09/2019	G43.9	10	Migraña	1
1	22/09/2019	H53	11	No patología	1
1	22/09/2019	H00.02	4	Orzuelo	1
1	22/09/2019	H16.0	1	Úlcera corneal	1
2	22/09/2019	H02.05	4	Distiquiasis	1

2	22/09/2019	H10.3	0	Conjuntivitis	1
1	22/09/2019	H10.3	0	Conjuntivitis	1
1	22/09/2019	H00.02	4	Orzuelo	1
2	22/09/2019	H10.3	0	Conjuntivitis	1
2	22/09/2019	H16.9	1	Queratitis	1
2	22/09/2019	H16.9	1	Queratitis	1
2	22/09/2019	H16.9	1	Queratitis	1
2	22/09/2019	H16.0	1	Úlcera corneal	1
1	22/09/2019	H10.3	0	Conjuntivitis	1
2	22/09/2019	H43.81	7	DVP	1
2	22/09/2019	H15.1	6	Epiescleritis	1
1	22/09/2019	H10.3	0	Conjuntivitis	1
1	22/09/2019	H11.3	0	Hiposfagma	1
1	22/09/2019	H00.02	4	Orzuelo	1
2	22/09/2019	H11.3	0	Hiposfagma	1
2	22/09/2019	H16.0	1	Úlcera corneal	1
2	22/09/2019	H16.9	1	Queratitis	1
2	22/09/2019	H33.30	8	Desgarro retiniano	1
1	23/09/2019	H16.9	1	Queratitis	1
1	23/09/2019	H10.3	0	Conjuntivitis	1
2	23/09/2019	H16.9	1	Queratitis	1
2	23/09/2019	H00.02	4	Orzuelo	1
1	23/09/2019	H10.3	0	Conjuntivitis	1
2	23/09/2019	H53	11	No patología	1
1	23/09/2019	H10.3	0	Conjuntivitis	1
1	23/09/2019	H16.0	1	Úlcera corneal	1
1	23/09/2019	H16.9	1	Queratitis	1
1	23/09/2019	H43.81	7	DVP	1
1	23/09/2019	G43.109	10	Aura	1
2	23/09/2019	S05.9	9	Traumatismo	1
2	23/09/2019	H33.30	8	Desgarro retiniano	1
2	23/09/2019	H16.9	1	Queratitis	1
1	23/09/2019	H43.81	7	DVP	1
2	23/09/2019	H53.10	11	Problema refractivo	1
2	23/09/2019	H43.81	7	DVP	1
1	23/09/2019	H10.3	0	Conjuntivitis	1
2	23/09/2019	H10.3	0	Conjuntivitis	1
2	23/09/2019	S05.9	9	Traumatismo	1
2	23/09/2019	H11.44	0	Quiste conjuntival	1
1	23/09/2019	H53	11	No patología	1
2	23/09/2019	H10.3	0	Conjuntivitis	1
2	23/09/2019	H00.02	4	Orzuelo	1
1	23/09/2019	H33.30	8	Desgarro retiniano	1
2	23/09/2019	H02.05	4	Distiquiasis	1
2	23/09/2019	S05.9	9	Traumatismo	1
1	23/09/2019	S05.9	9	Traumatismo	1
2	23/09/2019	H16.9	1	Queratitis	1
1	23/09/2019	H10.3	0	Conjuntivitis	1
1	23/09/2019	H16.9	1	Queratitis	1
1	23/09/2019	H43.81	7	DVP	1
1	23/09/2019	H10.3	0	Conjuntivitis	1
2	23/09/2019	H10.3	0	Conjuntivitis	1
1	23/09/2019	H10.3	0	Conjuntivitis	1
1	23/09/2019	H10.3	0	Conjuntivitis	1
1	23/09/2019	H35.3	8	Maculopatía	1
1	23/09/2019	H10.3	0	Conjuntivitis	1
1	23/09/2019	H53	11	No patología	1
2	23/09/2019	H04.32	5	Dacriocistitis aguda	1
2	23/09/2019	H16.0	1	Úlcera corneal	1
2	23/09/2019	H10.3	0	Conjuntivitis	1
2	23/09/2019	H00.02	4	Orzuelo	1
1	23/09/2019	H16.0	1	Úlcera corneal	1
2	23/09/2019	H01.00	4	Blefaritis	1
1	23/09/2019	H16.9	1	Queratitis	1
1	23/09/2019	H20	6	Uveitis	1
1	23/09/2019	H16.0	1	Úlcera corneal	1

1	23/09/2019	T15.0	1	Cuerpo extraño	1
1	23/09/2019	T15.0	1	Cuerpo extraño	1
2	23/09/2019	H16.0	1	Úlcera corneal	1
1	24/09/2019	H16.9	1	Queratitis	1
2	24/09/2019	H16.9	1	Queratitis	1
1	24/09/2019	H16.9	1	Queratitis	1
1	24/09/2019	T15.0	1	Cuerpo extraño	1
1	24/09/2019	H33.0	8	Desprendimiento de retina	1
1	24/09/2019	H10.5	4	Blefaroconjuntivitis	1
1	24/09/2019	H20	6	Uveitis	1
2	24/09/2019	G43.109	10	Aura	1
2	24/09/2019	H01.00	4	Blefaritis	1
2	24/09/2019	H00.02	4	Orzuelo	1
1	24/09/2019	T26	9	Causticación	1
2	24/09/2019	H53	11	No patología	1
2	24/09/2019	H59.3	4	Post op párpados	1
2	24/09/2019	H18.50	1	Distrofia corneal	1
2	24/09/2019	H01.00	4	Blefaritis	1
2	24/09/2019	H53	11	No patología	1
1	24/09/2019	H43.81	7	DVP	1
2	24/09/2019	H16.0	1	Úlcera corneal	1
1	24/09/2019	H20	6	Uveitis	1
1	24/09/2019	H43.81	7	DVP	1
1	24/09/2019	S05.9	9	Traumatismo	1
2	24/09/2019	H10.3	0	Conjuntivitis	1
1	24/09/2019	G43.9	10	Migraña	1
1	24/09/2019	T15.0	1	Cuerpo extraño	1
1	24/09/2019	H10.3	0	Conjuntivitis	1
1	24/09/2019	H10.81	0	Pingueculitis	1
2	24/09/2019	H16.9	1	Queratitis	1
1	24/09/2019	H01.00	4	Blefaritis	1
2	24/09/2019	H16.9	1	Queratitis	1
2	24/09/2019	H16.9	1	Queratitis	1
1	24/09/2019	T15.0	1	Cuerpo extraño	1
1	24/09/2019	E11.319	8	Retinopatía diabética	1
1	24/09/2019	T15.0	1	Cuerpo extraño	1
2	24/09/2019	H16.0	1	Úlcera corneal	1
1	24/09/2019	T15.0	1	Cuerpo extraño	1
1	24/09/2019	H10.3	0	Conjuntivitis	1
2	25/09/2019	H16.0	1	Úlcera corneal	1
1	25/09/2019	H16.9	1	Queratitis	1
1	25/09/2019	H16.0	1	Úlcera corneal	1
2	25/09/2019	H00.02	4	Orzuelo	1
1	25/09/2019	T15.0	1	Cuerpo extraño	1
2	25/09/2019	S05.9	9	Traumatismo	1
2	25/09/2019	H01.00	4	Blefaritis	1
1	25/09/2019	H01.00	4	Blefaritis	1
2	25/09/2019	Z98.4	2	Post op catarata	1
1	25/09/2019	H35.3	8	Maculopatía	1
2	25/09/2019	H35.30	8	DMAE	1
1	25/09/2019	H43.81	7	DVP	1
1	25/09/2019	H00.03	4	Celulitis preseptal	1
1	25/09/2019	H01.00	4	Blefaritis	1
2	25/09/2019	H53	11	No patología	1
2	25/09/2019	H11.3	0	Hiposfagma	1
1	25/09/2019	H16.9	1	Queratitis	1
2	25/09/2019	H00.02	4	Orzuelo	1
1	25/09/2019	H27.8	2	OCP	1
1	25/09/2019	H16.9	1	Queratitis	1
1	25/09/2019	H10.3	0	Conjuntivitis	1
2	25/09/2019	H33.30	8	Desgarro retiniano	1
2	25/09/2019	H43.81	7	DVP	1
1	25/09/2019	H11.3	0	Hiposfagma	1
2	25/09/2019	H10.3	0	Conjuntivitis	1
1	25/09/2019	H53	11	No patología	1
2	25/09/2019	H43.81	7	DVP	1

1	25/09/2019	H01.00	4	Blefaritis	1
2	25/09/2019	H16.0	1	Úlcera corneal	1
2	25/09/2019	H43.81	7	DVP	1
2	25/09/2019	H10.3	0	Conjuntivitis	1
2	25/09/2019	H16.0	1	Úlcera corneal	1
2	25/09/2019	H16.0	1	Úlcera corneal	1
1	25/09/2019	S05.9	9	Traumatismo	1
2	25/09/2019	B00.1	4	Dermatitis herpética	1
2	25/09/2019	S05.9	9	Traumatismo	1
1	26/09/2019	H16.9	1	Queratitis	1
1	26/09/2019	S05.9	9	Traumatismo	1
1	26/09/2019	T15.0	1	Cuerpo extraño	1
2	26/09/2019	H10.3	0	Conjuntivitis	1
2	26/09/2019	H0.41	1	Ojo seco	1
2	26/09/2019	H0.41	1	Ojo seco	1
2	26/09/2019	H11.3	0	Hiposfagma	1
2	26/09/2019	H16.9	1	Queratitis	1
2	26/09/2019	H02	4	Alteración palpebral	1
2	26/09/2019	H16.9	1	Queratitis	1
2	26/09/2019	H59.3	5	Postop via lagrimal	1
2	26/09/2019	H16.9	1	Queratitis	1
2	26/09/2019	H43.81	7	DVP	1
2	26/09/2019	H10.3	0	Conjuntivitis	1
2	26/09/2019	H10.3	0	Conjuntivitis	1
1	26/09/2019	T15.0	1	Cuerpo extraño	1
1	26/09/2019	H0.41	1	Ojo seco	1
2	26/09/2019	H16.9	1	Queratitis	1
2	26/09/2019	H16.9	1	Queratitis	1
2	26/09/2019	H16.9	1	Queratitis	1
2	26/09/2019	H11.3	0	Hiposfagma	1
1	26/09/2019	H11.3	0	Hiposfagma	1
2	26/09/2019	H00.02	4	Orzuelo	1
2	26/09/2019	H10.3	0	Conjuntivitis	1
2	26/09/2019	H00.02	4	Orzuelo	1
1	26/09/2019	T15.0	1	Cuerpo extraño	1
2	26/09/2019	H0.41	1	Ojo seco	1
2	26/09/2019	H00.02	4	Orzuelo	1
2	26/09/2019	H10.3	0	Conjuntivitis	1
2	26/09/2019	H11.3	0	Hiposfagma	1
1	26/09/2019	H16.9	1	Queratitis	1
2	26/09/2019	H00.19	4	Chalazión	1
1	26/09/2019	H27.8	2	OCP	1
1	26/09/2019	H16.0	1	Úlcera corneal	1
1	26/09/2019	H16.9	1	Queratitis	1
1	26/09/2019	H16.0	1	Úlcera corneal	1
1	26/09/2019	H10.3	0	Conjuntivitis	1
2	26/09/2019	S05.9	9	Traumatismo	1
1	26/09/2019	H40.21	3	Glaucoma agudo	1
1	26/09/2019	H53	11	No patología	1
1	26/09/2019	H11.3	0	Hiposfagma	1
2	26/09/2019	H11.44	0	Quiste conjuntival	1
1	26/09/2019	H16.9	1	Queratitis	1
2	26/09/2019	H10.3	0	Conjuntivitis	1
2	26/09/2019	H16.0	1	Úlcera corneal	1
2	27/09/2019	H10.3	0	Conjuntivitis	1
1	27/09/2019	H11.00	0	Pterigium	1
2	27/09/2019	H16.0	1	Úlcera corneal	1
1	27/09/2019	H11.3	0	Hiposfagma	1
2	27/09/2019	H10.3	0	Conjuntivitis	1
2	27/09/2019	H16.9	1	Queratitis	1
2	27/09/2019	H35.30	8	DMAE	1
2	27/09/2019	H16.9	1	Queratitis	1
2	27/09/2019	H59.3	5	Postop via lagrimal	1
1	27/09/2019	H27.8	2	OCP	1
2	27/09/2019	H27.8	2	OCP	1
1	27/09/2019	H47.10	10	Papiledema	1

1	27/09/2019	H11.3	0	Hiposfagma	1
2	27/09/2019	H10.3	0	Conjuntivitis	1
1	27/09/2019	H00.02	4	Orzuelo	1
2	27/09/2019	H16.9	1	Queratitis	1
1	27/09/2019	H43.81	7	DVP	1
2	27/09/2019	H27.8	2	OCP	1
2	27/09/2019	H16.9	1	Queratitis	1
2	27/09/2019	H16.9	1	Queratitis	1
2	27/09/2019	H16.0	1	Úlcera corneal	1
2	27/09/2019	H43.81	7	DVP	1
2	27/09/2019	H16.9	1	Queratitis	1
1	27/09/2019	H16.9	1	Queratitis	1
2	27/09/2019	H10.3	0	Conjuntivitis	1
2	27/09/2019	H16.0	1	Úlcera corneal	1
1	27/09/2019	T15.0	1	Cuerpo extraño	1
1	27/09/2019	H16.0	1	Úlcera corneal	1
2	27/09/2019	H01.00	4	Blefaritis	1
2	27/09/2019	H01.00	4	Blefaritis	1
2	27/09/2019	H16.0	1	Úlcera corneal	1
2	27/09/2019	H15.1	6	Epiescleritis	1
1	27/09/2019	H53.9	11	Alteraciones visuales	1
1	27/09/2019	H16.0	1	Úlcera corneal	1
2	27/09/2019	H16.0	1	Úlcera corneal	1
2	27/09/2019	G43.109	10	Aura	1
1	27/09/2019	H33.30	8	Desgarro retiniano	1
2	27/09/2019	H27.8	2	OCP	1
2	27/09/2019	T15.0	1	Cuerpo extraño	1
2	27/09/2019	H16.9	1	Queratitis	1
1	27/09/2019	H10.3	0	Conjuntivitis	1
2	27/09/2019	Alta por fuga	11	Alta por fuga	1
1	27/09/2019	H43.81	7	DVP	1
2	27/09/2019	H43.81	7	DVP	1
1	27/09/2019	H16.9	1	Queratitis	1
1	27/09/2019	H10.3	0	Conjuntivitis	1
2	27/09/2019	H10.3	0	Conjuntivitis	1
1	27/09/2019	H43.81	7	DVP	1
1	27/09/2019	H11.3	0	Hiposfagma	1
1	27/09/2019	H11.3	0	Hiposfagma	1
2	27/09/2019	H11.3	0	Hiposfagma	1
1	27/09/2019	H43.81	7	DVP	1
1	27/09/2019	H16.0	1	Úlcera corneal	1
2	27/09/2019	H10.3	0	Conjuntivitis	1
1	27/09/2019	T15.0	1	Cuerpo extraño	1
1	28/09/2019	H11.3	0	Hiposfagma	1
1	28/09/2019	H02.05	4	Distiquiasis	1
1	28/09/2019	H10.3	0	Conjuntivitis	1
2	28/09/2019	H43.81	7	DVP	1
2	28/09/2019	H16.9	1	Queratitis	1
1	28/09/2019	H16.0	1	Úlcera corneal	1
1	28/09/2019	H01.00	4	Blefaritis	1
1	28/09/2019	H0.41	1	Ojo seco	1
1	28/09/2019	H10.3	0	Conjuntivitis	1
1	28/09/2019	H00.02	4	Orzuelo	1
1	28/09/2019	H11.3	0	Hiposfagma	1
1	28/09/2019	H10.3	0	Conjuntivitis	1
1	28/09/2019	H16.0	1	Úlcera corneal	1
2	28/09/2019	H17.8	1	Infiltrados corneales	1
2	28/09/2019	H10.3	0	Conjuntivitis	1
1	28/09/2019	H20	6	Uveitis	1
2	28/09/2019	H43.81	7	DVP	1
2	28/09/2019	H10.3	0	Conjuntivitis	1
2	28/09/2019	H10.3	0	Conjuntivitis	1
2	28/09/2019	H11.3	0	Hiposfagma	1
2	28/09/2019	H10.3	0	Conjuntivitis	1
2	28/09/2019	H15.1	6	Epiescleritis	1
1	28/09/2019	H16.9	1	Queratitis	1

1	28/09/2019	H33.0		8	Desprendimiento de retina	1
2	28/09/2019	H10.3		0	Conjuntivitis	1
1	28/09/2019	H43.81		7	DVP	1
2	28/09/2019	H10.3		0	Conjuntivitis	1
1	28/09/2019	H16.0		1	Úlcera corneal	1
2	28/09/2019	H16.9		1	Queratitis	1
2	28/09/2019	Z98.4		2	Post op catarata	1
2	28/09/2019	H10.81		0	Pingueculitis	1
2	28/09/2019	H0.41		1	Ojo seco	1
2	28/09/2019	H16.0		1	Úlcera corneal	1
2	28/09/2019	H0.41		1	Ojo seco	1
2	28/09/2019	H16.0		1	Úlcera corneal	1
1	28/09/2019	H16.0		1	Úlcera corneal	1
1	28/09/2019	H10.3		0	Conjuntivitis	1
2	28/09/2019	H15.1		6	Epiescleritis	1
1	28/09/2019	H10.3		0	Conjuntivitis	1
1	28/09/2019	T15.0		1	Cuerpo extraño	1
2	28/09/2019	H11.3		0	Hiposfagma	1
1	28/09/2019	H17.8		1	Infiltrados corneales	1
1	28/09/2019	T15.0		1	Cuerpo extraño	1
1	28/09/2019	T15.0		1	Cuerpo extraño	1
2	28/09/2019	H53		11	No patología	1
1	28/09/2019	H33.30		8	Desgarro retiniano	1
1	28/09/2019	H10.3		0	Conjuntivitis	1
2	29/09/2019	H16.0		1	Úlcera corneal	1
1	29/09/2019	T15.0		1	Cuerpo extraño	1
2	29/09/2019	H16.0		1	Úlcera corneal	1
2	29/09/2019	H02.05		4	Distiquiasis	1
1	29/09/2019	H26.9		2	Catarata	1
2	29/09/2019	H11.3		0	Hiposfagma	1
1	29/09/2019	H00.02		4	Orzuelo	1
2	29/09/2019	T15.0		1	Cuerpo extraño	1
2	29/09/2019	H16.9		1	Queratitis	1
2	29/09/2019	H10.3		0	Conjuntivitis	1
2	29/09/2019	H16.9		1	Queratitis	1
2	29/09/2019	H01.00		4	Blefaritis	1
2	29/09/2019	H16.0		1	Úlcera corneal	1
2	29/09/2019	H16.0		1	Úlcera corneal	1
1	29/09/2019	H01.00		4	Blefaritis	1
2	29/09/2019	H53.9		11	Alteraciones visuales	1
2	29/09/2019	H16.9		1	Queratitis	1
1	29/09/2019	H35.3		8	Maculopatía	1
2	29/09/2019	H02		4	Lesión palpebral	1
2	29/09/2019	H16.0		1	Úlcera corneal	1
1	29/09/2019	H16.0		1	Úlcera corneal	1
2	29/09/2019	H10.3		0	Conjuntivitis	1
1	29/09/2019	T15.0		1	Cuerpo extraño	1
2	29/09/2019	H01.00		4	Blefaritis	1
2	29/09/2019	H16.0		1	Úlcera corneal	1
2	29/09/2019	H16.0		1	Úlcera corneal	1
2	29/09/2019	H17.8		1	Infiltrados corneales	1
1	29/09/2019	H43.81		7	DVP	1
1	29/09/2019	H11.44		0	Quiste conjuntival	1
1	29/09/2019	H33.30		8	Desgarro retiniano	1
1	29/09/2019	T15.0		1	Cuerpo extraño	1
2	29/09/2019	H43.81		7	DVP	1
2	29/09/2019	T26		9	Causticación	1
1	29/09/2019	H10.3		0	Conjuntivitis	1
1	30/09/2019	T26		9	Causticación	1
2	30/09/2019	H10.3		0	Conjuntivitis	1
2	30/09/2019	H00.02		4	Orzuelo	1
1	30/09/2019	H11.3		0	Hiposfagma	1
1	30/09/2019	H16.9		1	Queratitis	1
1	30/09/2019	H43.81		7	DVP	1
1	30/09/2019	H10.3		0	Conjuntivitis	1
2	30/09/2019	H16.9		1	Queratitis	1

2	30/09/2019	H17.8	1	Infiltrados corneales	1
2	30/09/2019	H16.9	1	Queratitis	1
1	30/09/2019	H10.3	0	Conjuntivitis	1
2	30/09/2019	H10.3	0	Conjuntivitis	1
2	30/09/2019	H01.00	4	Blefaritis	1
2	30/09/2019	H16.0	1	Úlcera corneal	1
2	30/09/2019	H16.9	1	Queratitis	1
1	30/09/2019	H33.0	8	Desprendimiento de retina	1
2	30/09/2019	H40.9	3	Glaucoma	1
2	30/09/2019	S05.9	9	Traumatismo	1
1	30/09/2019	H16.0	1	Úlcera corneal	1
1	30/09/2019	H10.3	0	Conjuntivitis	1
1	30/09/2019	H35.3	8	Maculopatía	1
2	30/09/2019	H43.81	7	DVP	1
1	30/09/2019	H10.3	0	Conjuntivitis	1
1	30/09/2019	T15.0	1	Cuerpo extraño	1
2	30/09/2019	B02.29	10	Neuralgia herpética	1
2	30/09/2019	H20	6	Uveitis	1
1	30/09/2019	G45.3	10	Amaurosis fugax	1
2	30/09/2019	H10.3	0	Conjuntivitis	1
1	30/09/2019	H00.02	4	Orzuelo	1
2	30/09/2019	H17.8	1	Infiltrados corneales	1
1	30/09/2019	H16.0	1	Úlcera corneal	1
2	30/09/2019	H10.3	0	Conjuntivitis	1
1	30/09/2019	T15.0	1	Cuerpo extraño	1
2	30/09/2019	H43.81	7	DVP	1
2	30/09/2019	H43.81	7	DVP	1
2	30/09/2019	H10.3	0	Conjuntivitis	1
1	30/09/2019	H15.00	6	Escleritis	1
1	30/09/2019	S05.9	9	Traumatismo	1
2	30/09/2019	H20	6	Uveitis	1
1	30/09/2019	H16.0	1	Úlcera corneal	1
2	30/09/2019	H43.81	7	DVP	1
2	30/09/2019	H16.9	1	Queratitis	1
2	30/09/2019	S05.30	9	Laceración conjuntival	1
2	01/10/2019	H20	6	Uveitis	1
1	01/10/2019	H16.9	1	Queratitis	1
2	01/10/2019	H16.0	1	Úlcera corneal	1
1	01/10/2019	H16.9	1	Queratitis	1
2	01/10/2019	H16.9	1	Queratitis	1
1	01/10/2019	H02	4	Alteración palpebral	1
1	01/10/2019	H53.9	11	Alteraciones visuales	1
1	01/10/2019	H16.9	1	Queratitis	1
2	01/10/2019	H10.3	0	Conjuntivitis	1
2	01/10/2019	H02.05	4	Distiquiasis	1
2	01/10/2019	H00.02	4	Orzuelo	1
1	01/10/2019	H26.9	2	Catarata	1
2	01/10/2019	H10.3	0	Conjuntivitis	1
2	01/10/2019	H17.8	1	Infiltrados corneales	1
2	01/10/2019	H02	4	Alteración palpebral	1
1	01/10/2019	H33.30	8	Desgarro retiniano	1
1	01/10/2019	H43.81	7	DVP	1
1	01/10/2019	H01.00	4	Blefaritis	1
2	01/10/2019	H53.10	11	Problema refractivo	1
1	01/10/2019	H16.9	1	Queratitis	1
2	01/10/2019	H10.3	0	Conjuntivitis	1
1	01/10/2019	H43.81	7	DVP	1
1	01/10/2019	H53	11	No patología	1
2	01/10/2019	H10.3	0	Conjuntivitis	1
1	01/10/2019	H11.3	0	Hiposfagma	1
1	01/10/2019	H11.3	0	Hiposfagma	1
2	01/10/2019	H49.9	10	Parálisis oculomotora	1
1	01/10/2019	H10.3	0	Conjuntivitis	1
1	01/10/2019	H16.9	1	Queratitis	1
1	01/10/2019	H33.30	8	Desgarro retiniano	1
1	01/10/2019	H11.3	0	Hiposfagma	1

2	01/10/2019	H01.00	4	Blefaritis	1
2	01/10/2019	H16.9	1	Queratitis	1
2	01/10/2019	H43.1	7	Hemovítreo	1
2	01/10/2019	H43.81	7	DVP	1
1	01/10/2019	T15.0	1	Cuerpo extraño	1
1	01/10/2019	H16.9	1	Queratitis	1
1	01/10/2019	T15.0	1	Cuerpo extraño	1
2	01/10/2019	H10.3	0	Conjuntivitis	1
1	01/10/2019	T15.0	1	Cuerpo extraño	1
1	01/10/2019	H53	11	No patología	1
1	01/10/2019	H16.9	1	Queratitis	1
1	01/10/2019	H16.0	1	Úlcera corneal	1
1	02/10/2019	H16.9	1	Queratitis	1
1	02/10/2019	H16.0	1	Úlcera corneal	1
1	02/10/2019	S05.9	9	Traumatismo	1
2	02/10/2019	H16.0	1	Úlcera corneal	1
2	02/10/2019	H53	11	No patología	1
2	02/10/2019	H16.0	1	Úlcera corneal	1
1	02/10/2019	H53	11	No patología	1
1	02/10/2019	H10.3	0	Conjuntivitis	1
2	02/10/2019	H16.0	1	Úlcera corneal	1
2	02/10/2019	H02.05	4	Distiquiasis	1
2	02/10/2019	H43.1	7	Hemovítreo	1
2	02/10/2019	Alta por fuga	11	Alta por fuga	1
2	02/10/2019	H20	6	Uveitis	1
1	02/10/2019	H53	11	No patología	1
2	02/10/2019	H16.9	1	Queratitis	1
2	02/10/2019	H53	11	No patología	1
1	02/10/2019	H00.02	4	Orzuelo	1
1	02/10/2019	H53	11	No patología	1
2	02/10/2019	H43.81	7	DVP	1
2	02/10/2019	H35.30	8	DMAE	1
2	02/10/2019	H53	11	No patología	1
1	02/10/2019	H01.00	4	Blefaritis	1
2	02/10/2019	H10.3	0	Conjuntivitis	1
2	02/10/2019	H53	11	No patología	1
1	02/10/2019	H00.19	4	Chalazión	1
1	02/10/2019	T15.0	1	Cuerpo extraño	1
2	02/10/2019	H16.9	1	Queratitis	1
1	02/10/2019	H04.32	5	Dacriocistitis aguda	1
1	02/10/2019	H20	6	Uveitis	1
1	02/10/2019	H16.9	1	Queratitis	1
1	02/10/2019	H00.02	4	Orzuelo	1
1	02/10/2019	H35.3	8	Maculopatía	1
2	02/10/2019	H16.0	1	Úlcera corneal	1
2	02/10/2019	H10.5	4	Blefaroconjuntivitis	1
1	02/10/2019	H43.81	7	DVP	1
1	02/10/2019	H16.9	1	Queratitis	1
2	02/10/2019	H20	6	Uveitis	1
2	02/10/2019	H43.81	7	DVP	1
2	02/10/2019	H11.3	0	Hiposfagma	1
2	02/10/2019	S05.9	9	Traumatismo	1
1	02/10/2019	H43.81	7	DVP	1
2	02/10/2019	H01.00	4	Blefaritis	1
1	02/10/2019	H16.0	1	Úlcera corneal	1
1	02/10/2019	H11.3	0	Hiposfagma	1
2	02/10/2019	H10.3	0	Conjuntivitis	1
1	02/10/2019	H10.3	0	Conjuntivitis	1
1	02/10/2019	H10.3	0	Conjuntivitis	1
1	02/10/2019	H15.1	6	Epiescleritis	1
2	02/10/2019	H10.3	0	Conjuntivitis	1
2	02/10/2019	H11.3	0	Hiposfagma	1
2	02/10/2019	H16.9	1	Queratitis	1
2	02/10/2019	H53	11	No patología	1
2	02/10/2019	H53	11	No patología	1
2	02/10/2019	H10.3	0	Conjuntivitis	1

2	02/10/2019	H16.0	1	Úlcera corneal	1
2	02/10/2019	H16.9	1	Queratitis	1
2	02/10/2019	H02	4	Alteración palpebral	1
2	02/10/2019	H16.9	1	Queratitis	1
2	02/10/2019	H16.9	1	Queratitis	1
1	02/10/2019	H10.3	0	Conjuntivitis	1
2	02/10/2019	H00.02	4	Orzuelo	1
1	02/10/2019	T26	9	Causticación	1
1	02/10/2019	S05.9	9	Traumatismo	1
1	03/10/2019	T15.0	1	Cuerpo extraño	1
2	03/10/2019	H43.81	7	DVP	1
1	03/10/2019	H11.3	0	Hiposfagma	1
2	03/10/2019	H00.02	4	Orzuelo	1
1	03/10/2019	H10.3	0	Conjuntivitis	1
2	03/10/2019	H02	4	Lesión palpebral	1
1	03/10/2019	S05.9	9	Traumatismo	1
1	03/10/2019	H16.0	1	Úlcera corneal	1
1	03/10/2019	H10.3	0	Conjuntivitis	1
1	03/10/2019	H11.3	0	Hiposfagma	1
1	03/10/2019	H16.0	1	Úlcera corneal	1
2	03/10/2019	H15.1	6	Epiescleritis	1
1	03/10/2019	H16.9	1	Queratitis	1
1	03/10/2019	H10.3	0	Conjuntivitis	1
1	03/10/2019	H43.81	7	DVP	1
1	03/10/2019	H35.30	8	DMAE	1
1	03/10/2019	H43.81	7	DVP	1
2	03/10/2019	H10.3	0	Conjuntivitis	1
1	03/10/2019	T15.0	1	Cuerpo extraño	1
1	03/10/2019	H16.0	1	Úlcera corneal	1
1	03/10/2019	S05.9	9	Traumatismo	1
2	03/10/2019	H16.9	1	Queratitis	1
2	03/10/2019	T15.0	1	Cuerpo extraño	1
1	03/10/2019	H43.81	7	DVP	1
1	03/10/2019	H16.9	1	Queratitis	1
1	03/10/2019	H16.0	1	Úlcera corneal	1
1	03/10/2019	S05.9	9	Traumatismo	1
2	03/10/2019	S05.9	9	Traumatismo	1
2	03/10/2019	H16.0	1	Úlcera corneal	1
2	03/10/2019	H16.9	1	Queratitis	1
1	03/10/2019	H16.0	1	Úlcera corneal	1
2	03/10/2019	H53	11	No patología	1
2	03/10/2019	H16.9	1	Queratitis	1
1	03/10/2019	H01.00	4	Blefaritis	1
1	03/10/2019	H17.8	1	Infiltrados corneales	1
2	03/10/2019	H10.3	0	Conjuntivitis	1
1	03/10/2019	H43.81	7	DVP	1
1	03/10/2019	H16.9	1	Queratitis	1
2	03/10/2019	H16.9	1	Queratitis	1
2	03/10/2019	H10.3	0	Conjuntivitis	1
1	03/10/2019	H10.3	0	Conjuntivitis	1
1	03/10/2019	H16.0	1	Úlcera corneal	1
1	04/10/2019	H10.3	0	Conjuntivitis	1
2	04/10/2019	H16.0	1	Úlcera corneal	1
1	04/10/2019	H16.9	1	Queratitis	1
1	04/10/2019	H53	11	No patología	1
1	04/10/2019	H15.00	6	Escleritis	1
1	04/10/2019	H16.9	1	Queratitis	1
1	04/10/2019	H11.3	0	Hiposfagma	1
2	04/10/2019	H16.9	1	Queratitis	1
2	04/10/2019	H02	4	Lesión palpebral	1
2	04/10/2019	H43.81	7	DVP	1
2	04/10/2019	H16.9	1	Queratitis	1
2	04/10/2019	G43.109	10	Aura	1
2	04/10/2019	H02	4	Alteración palpebral	1
2	04/10/2019	H11.3	0	Hiposfagma	1
2	04/10/2019	H16.9	1	Queratitis	1

1	04/10/2019	H16.9	1	Queratitis	1
2	04/10/2019	H43.81	7	DVP	1
2	04/10/2019	H27.8	2	OCP	1
2	04/10/2019	H10.3	0	Conjuntivitis	1
2	04/10/2019	H02	4	Lesión palpebral	1
2	04/10/2019	H01.00	4	Blefaritis	1
2	04/10/2019	H10.3	0	Conjuntivitis	1
2	04/10/2019	H10.3	0	Conjuntivitis	1
1	04/10/2019	H16.9	1	Queratitis	1
2	04/10/2019	H01.00	4	Blefaritis	1
1	04/10/2019	H10.3	0	Conjuntivitis	1
2	04/10/2019	H16.9	1	Queratitis	1
2	04/10/2019	H16.0	1	Úlcera corneal	1
2	04/10/2019	H10.3	0	Conjuntivitis	1
1	04/10/2019	H11.3	0	Hiposfagma	1
2	04/10/2019	H10.3	0	Conjuntivitis	1
1	04/10/2019	H53	11	No patología	1
1	04/10/2019	H43.81	7	DVP	1
2	04/10/2019	H16.0	1	Úlcera corneal	1
1	04/10/2019	H00.02	4	Orzuelo	1
1	04/10/2019	H16.0	1	Úlcera corneal	1
1	04/10/2019	H16.9	1	Queratitis	1
1	04/10/2019	T15.0	1	Cuerpo extraño	1
1	04/10/2019	T15.0	1	Cuerpo extraño	1
2	04/10/2019	H16.9	1	Queratitis	1
2	04/10/2019	H16.9	1	Queratitis	1
2	04/10/2019	H43.81	7	DVP	1
1	04/10/2019	H16.0	1	Úlcera corneal	1
2	04/10/2019	H16.0	1	Úlcera corneal	1
2	05/10/2019	H16.0	1	Úlcera corneal	1
2	05/10/2019	H10.3	0	Conjuntivitis	1
1	05/10/2019	H01.00	4	Blefaritis	1
1	05/10/2019	H33.0	8	Desprendimiento de retina	1
1	05/10/2019	H00.02	4	Orzuelo	1
1	05/10/2019	H01.00	4	Blefaritis	1
1	05/10/2019	H59.3	4	Post op párpados	1
2	05/10/2019	H02	4	Lesión palpebral	1
2	05/10/2019	H27.8	2	OCP	1
2	05/10/2019	H02.05	4	Distiquiasis	1
1	05/10/2019	H43.81	7	DVP	1
2	05/10/2019	H10.3	0	Conjuntivitis	1
2	05/10/2019	H10.3	0	Conjuntivitis	1
2	05/10/2019	H43.81	7	DVP	1
2	05/10/2019	H16.0	1	Úlcera corneal	1
1	05/10/2019	H10.3	0	Conjuntivitis	1
1	05/10/2019	S05.9	9	Traumatismo	1
1	05/10/2019	H16.9	1	Queratitis	1
1	05/10/2019	S05.9	9	Traumatismo	1
2	05/10/2019	H20	6	Uveitis	1
2	05/10/2019	H02	4	Lesión palpebral	1
2	05/10/2019	H11.3	0	Hiposfagma	1
1	05/10/2019	T15.0	1	Cuerpo extraño	1
1	05/10/2019	T15.0	1	Cuerpo extraño	1
2	05/10/2019	H01.00	4	Blefaritis	1
1	05/10/2019	H01.00	4	Blefaritis	1
2	05/10/2019	H16.9	1	Queratitis	1
1	05/10/2019	H16.0	1	Úlcera corneal	1
2	05/10/2019	H16.0	1	Úlcera corneal	1
2	05/10/2019	S05.9	9	Traumatismo	1
2	05/10/2019	H10.3	0	Conjuntivitis	1
1	05/10/2019	H10.3	0	Conjuntivitis	1
2	05/10/2019	H16.9	1	Queratitis	1
1	05/10/2019	H16.0	1	Úlcera corneal	1
2	05/10/2019	H10.3	0	Conjuntivitis	1
2	05/10/2019	H01.00	4	Blefaritis	1
2	05/10/2019	H16.0	1	Úlcera corneal	1

1	05/10/2019	H59.88	8	Post op retina	1
1	05/10/2019	T15.0	1	Cuerpo extraño	1
2	05/10/2019	H16.9	1	Queratitis	1
1	05/10/2019	H20	6	Uveitis	1
1	05/10/2019	H20	6	Uveitis	1
1	05/10/2019	H16.0	1	Úlcera corneal	1
1	05/10/2019	H43.81	7	DVP	1
1	05/10/2019	H16.9	1	Queratitis	1
2	05/10/2019	E11.319	8	Retinopatía diabética	1
2	05/10/2019	H15.1	6	Epiescleritis	1
1	05/10/2019	H00.02	4	Orzuelo	1
2	05/10/2019	H10.3	0	Conjuntivitis	1
1	05/10/2019	T15.0	1	Cuerpo extraño	1
2	05/10/2019	T26	9	Causticación	1
1	05/10/2019	H16.0	1	Úlcera corneal	1
2	05/10/2019	H33.30	8	Desgarro retiniano	1
2	05/10/2019	H00.02	4	Orzuelo	1
1	05/10/2019	H10.5	4	Blefaroconjuntivitis	1
2	06/10/2019	B00.1	4	Dermatitis herpética	1
2	06/10/2019	H10.3	0	Conjuntivitis	1
2	06/10/2019	H17.8	1	Infiltrados corneales	1
2	06/10/2019	H16.0	1	Úlcera corneal	1
1	06/10/2019	H35.3	8	Maculopatía	1
2	06/10/2019	H20	6	Uveitis	1
2	06/10/2019	T15.0	1	Cuerpo extraño	1
2	06/10/2019	H33.30	8	Desgarro retiniano	1
1	06/10/2019	H16.0	1	Úlcera corneal	1
2	06/10/2019	H00.03	4	Celulitis preseptal	1
2	06/10/2019	H16.9	1	Queratitis	1
1	06/10/2019	H16.0	1	Úlcera corneal	1
1	06/10/2019	H20	6	Uveitis	1
2	06/10/2019	S05.30	9	Laceración conjuntival	1
1	06/10/2019	H00.19	4	Chalazión	1
1	06/10/2019	H05.01	4	Celulitis orbitaria	1
2	06/10/2019	H59.3	4	Post op párpados	1
1	06/10/2019	H43.81	7	DVP	1
1	06/10/2019	H16.0	1	Úlcera corneal	1
2	06/10/2019	H15.00	6	Escleritis	1
1	06/10/2019	H16.0	1	Úlcera corneal	1
1	06/10/2019	H43.1	7	Hemovítreo	1
2	06/10/2019	H18.20	1	Edema corneal	1
1	06/10/2019	H16.9	1	Queratitis	1
1	06/10/2019	H10.5	4	Blefaroconjuntivitis	1
2	06/10/2019	H43.81	7	DVP	1
1	06/10/2019	H17.8	1	Infiltrados corneales	1
2	06/10/2019	H16.9	1	Queratitis	1
1	06/10/2019	H16.0	1	Úlcera corneal	1
2	06/10/2019	H16.9	1	Queratitis	1
2	06/10/2019	H16.9	1	Queratitis	1
1	06/10/2019	H43.81	7	DVP	1
1	06/10/2019	S05.9	9	Traumatismo	1
1	06/10/2019	H43.81	7	DVP	1
2	06/10/2019	H16.0	1	Úlcera corneal	1
1	06/10/2019	H33.0	8	Desprendimiento de retina	1
1	06/10/2019	H20	6	Uveitis	1
2	06/10/2019	H15.1	6	Epiescleritis	1
2	06/10/2019	H10.3	0	Conjuntivitis	1
1	07/10/2019	H16.0	1	Úlcera corneal	1
1	07/10/2019	H16.9	1	Queratitis	1
1	07/10/2019	H10.3	0	Conjuntivitis	1
2	07/10/2019	H10.3	0	Conjuntivitis	1
1	07/10/2019	H35.30	8	DMAE	1
2	07/10/2019	H00.02	4	Orzuelo	1
2	07/10/2019	H33.30	8	Desgarro retiniano	1
1	07/10/2019	H10.3	0	Conjuntivitis	1
2	07/10/2019	H43.81	7	DVP	1

2	07/10/2019	H10.3	0	Conjuntivitis	1
2	07/10/2019	H16.9	1	Queratitis	1
2	07/10/2019	H15.1	6	Epiescleritis	1
2	07/10/2019	H02	4	Lesión palpebral	1
2	07/10/2019	H01.00	4	Blefaritis	1
1	07/10/2019	S05.9	9	Traumatismo	1
1	07/10/2019	H43.81	7	DVP	1
1	07/10/2019	T15.0	1	Cuerpo extraño	1
2	07/10/2019	H16.9	1	Queratitis	1
2	07/10/2019	H02	4	Lesión palpebral	1
1	07/10/2019	H11.3	0	Hiposfagma	1
2	07/10/2019	H10.3	0	Conjuntivitis	1
1	07/10/2019	H10.3	0	Conjuntivitis	1
1	07/10/2019	H02	4	Alteración palpebral	1
2	07/10/2019	H43.81	7	DVP	1
2	07/10/2019	H10.3	0	Conjuntivitis	1
1	07/10/2019	H11.3	0	Hiposfagma	1
1	07/10/2019	H33.0	8	Desprendimiento de retina	1
2	07/10/2019	H26.9	2	Catarata	1
2	07/10/2019	H16.9	1	Queratitis	1
1	07/10/2019	H10.3	0	Conjuntivitis	1
2	07/10/2019	H43.81	7	DVP	1
1	07/10/2019	H16.0	1	Úlcera corneal	1
1	07/10/2019	H34.82	8	Trombosis venosa	1
2	07/10/2019	H02	4	Alteración palpebral	1
2	07/10/2019	H11.3	0	Hiposfagma	1
2	07/10/2019	H16.9	1	Queratitis	1
1	07/10/2019	H16.0	1	Úlcera corneal	1
1	07/10/2019	S05.9	9	Traumatismo	1
1	07/10/2019	H10.3	0	Conjuntivitis	1
1	07/10/2019	H20	6	Uveitis	1
2	07/10/2019	H10.3	0	Conjuntivitis	1
1	07/10/2019	T15.0	1	Cuerpo extraño	1
1	07/10/2019	H16.9	1	Queratitis	1
2	07/10/2019	T15.0	1	Cuerpo extraño	1
1	07/10/2019	H16.0	1	Úlcera corneal	1
2	07/10/2019	H53	11	No patología	1
1	07/10/2019	H43.81	7	DVP	1
2	07/10/2019	H40.9	3	Glaucoma	1
2	07/10/2019	H10.3	0	Conjuntivitis	1
2	07/10/2019	H10.3	0	Conjuntivitis	1
2	07/10/2019	H53.9	11	Alteraciones visuales	1
2	08/10/2019	H16.9	1	Queratitis	1
2	08/10/2019	H00.02	4	Orzuelo	1
2	08/10/2019	H10.3	0	Conjuntivitis	1
1	08/10/2019	H27.8	2	OCP	1
2	08/10/2019	H16.0	1	Úlcera corneal	1
2	08/10/2019	H02	4	Alteración palpebral	1
2	08/10/2019	H10.3	0	Conjuntivitis	1
2	08/10/2019	H33.30	8	Desgarro retiniano	1
2	08/10/2019	H16.9	1	Queratitis	1
2	08/10/2019	H16.0	1	Úlcera corneal	1
2	08/10/2019	H27.8	2	OCP	1
1	08/10/2019	H00.02	4	Orzuelo	1
2	08/10/2019	H16.9	1	Queratitis	1
1	08/10/2019	H16.9	1	Queratitis	1
2	08/10/2019	H43.81	7	DVP	1
2	08/10/2019	H53.9	11	Alteraciones visuales	1
1	08/10/2019	H16.9	1	Queratitis	1
2	08/10/2019	H16.0	1	Úlcera corneal	1
2	08/10/2019	H33.30	8	Desgarro retiniano	1
2	08/10/2019	H10.3	0	Conjuntivitis	1
2	08/10/2019	H02	4	Lesión palpebral	1
2	08/10/2019	H16.0	1	Úlcera corneal	1
2	08/10/2019	H16.0	1	Úlcera corneal	1
2	08/10/2019	H15.1	6	Epiescleritis	1

2	08/10/2019	H04.32	5	Dacriocistitis aguda	1
2	08/10/2019	H53	11	No patología	1
1	08/10/2019	H16.9	1	Queratitis	1
2	08/10/2019	H16.0	1	Úlcera corneal	1
2	08/10/2019	H16.0	1	Úlcera corneal	1
1	08/10/2019	H16.9	1	Queratitis	1
1	08/10/2019	H16.0	1	Úlcera corneal	1
1	08/10/2019	H11.3	0	Hiposfagma	1
1	08/10/2019	H10.3	0	Conjuntivitis	1
2	08/10/2019	H43.81	7	DVP	1
1	09/10/2019	H16.0	1	Úlcera corneal	1
2	09/10/2019	H16.0	1	Úlcera corneal	1
2	09/10/2019	H16.9	1	Queratitis	1
1	09/10/2019	H16.9	1	Queratitis	1
2	09/10/2019	H43.81	7	DVP	1
2	09/10/2019	H20	6	Uveitis	1
2	09/10/2019	H53	11	No patología	1
2	09/10/2019	H15.1	6	Epiescleritis	1
2	09/10/2019	H43.81	7	DVP	1
2	09/10/2019	H16.9	1	Queratitis	1
2	09/10/2019	H10.3	0	Conjuntivitis	1
1	09/10/2019	S05.30	9	Laceración conjuntival	1
2	09/10/2019	H16.9	1	Queratitis	1
2	09/10/2019	H35.3	8	Maculopatía	1
1	09/10/2019	H16.0	1	Úlcera corneal	1
2	09/10/2019	H01.00	4	Blefaritis	1
1	09/10/2019	H16.0	1	Úlcera corneal	1
2	09/10/2019	H43.81	7	DVP	1
2	09/10/2019	H0.41	1	Ojo seco	1
2	09/10/2019	H16.0	1	Úlcera corneal	1
2	09/10/2019	H10.3	0	Conjuntivitis	1
1	09/10/2019	H43.81	7	DVP	1
2	09/10/2019	H16.0	1	Úlcera corneal	1
1	09/10/2019	H16.9	1	Queratitis	1
2	09/10/2019	H16.9	1	Queratitis	1
1	09/10/2019	H20	6	Uveitis	1
2	09/10/2019	H16.0	1	Úlcera corneal	1
1	09/10/2019	H10.3	0	Conjuntivitis	1
1	09/10/2019	H16.9	1	Queratitis	1
2	09/10/2019	H43.81	7	DVP	1
2	09/10/2019	H16.0	1	Úlcera corneal	1
2	09/10/2019	H16.9	1	Queratitis	1
2	09/10/2019	H43.81	7	DVP	1
2	09/10/2019	G43.109	10	Aura	1
2	09/10/2019	H53.10	11	Problema refractivo	1
2	09/10/2019	H53	11	No patología	1
1	09/10/2019	H10.3	0	Conjuntivitis	1
1	09/10/2019	H43.81	7	DVP	1
2	09/10/2019	H11.3	0	Hiposfagma	1
1	09/10/2019	H01.00	4	Blefaritis	1
1	09/10/2019	H16.9	1	Queratitis	1
2	09/10/2019	H17.8	1	Infiltrados corneales	1
1	09/10/2019	H10.3	0	Conjuntivitis	1
2	09/10/2019	H02.05	4	Distiquiasis	1
2	09/10/2019	H16.0	1	Úlcera corneal	1
1	09/10/2019	H11.3	0	Hiposfagma	1
1	09/10/2019	H10.3	0	Conjuntivitis	1
2	09/10/2019	H00.19	4	Chalazión	1
1	09/10/2019	T15.0	1	Cuerpo extraño	1
2	09/10/2019	H43.81	7	DVP	1
1	09/10/2019	H16.9	1	Queratitis	1
2	10/10/2019	S05.30	9	Laceración conjuntival	1
2	10/10/2019	H43.81	7	DVP	1
1	10/10/2019	H10.3	0	Conjuntivitis	1
2	10/10/2019	H43.81	7	DVP	1
2	10/10/2019	H02	4	Alteración palpebral	1

2	10/10/2019	H16.9	1	Queratitis	1
2	10/10/2019	H43.81	7	DVP	1
1	10/10/2019	H16.0	1	Úlcera corneal	1
1	10/10/2019	H10.3	0	Conjuntivitis	1
1	10/10/2019	H43.81	7	DVP	1
2	10/10/2019	H47.10	10	Papiledema	1
2	10/10/2019	H16.9	1	Queratitis	1
1	10/10/2019	G43.109	10	Aura	1
2	10/10/2019	H0.41	1	Ojo seco	1
2	10/10/2019	H53	11	No patología	1
2	10/10/2019	H16.9	1	Queratitis	1
2	10/10/2019	H11.00	0	Pterigium	1
2	10/10/2019	H0.41	1	Ojo seco	1
2	10/10/2019	H53.9	11	Alteraciones visuales	1
1	10/10/2019	Alta por fuga	11	Alta por fuga	1
2	10/10/2019	H10.5	4	Blefaroconjuntivitis	1
2	10/10/2019	H53	11	No patología	1
2	10/10/2019	H17.8	1	Infiltrados corneales	1
2	10/10/2019	H35.3	8	Maculopatía	1
2	10/10/2019	H20	6	Uveitis	1
2	10/10/2019	H16.0	1	Úlcera corneal	1
1	10/10/2019	H27.8	2	OCP	1
1	10/10/2019	T15.0	1	Cuerpo extraño	1
1	10/10/2019	H11.3	0	Hiposfagma	1
1	10/10/2019	H10.3	0	Conjuntivitis	1
1	10/10/2019	H17.8	1	Infiltrados corneales	1
1	10/10/2019	H11.3	0	Hiposfagma	1
2	10/10/2019	H43.81	7	DVP	1
1	10/10/2019	H10.3	0	Conjuntivitis	1
2	10/10/2019	H10.3	0	Conjuntivitis	1
2	10/10/2019	H10.3	0	Conjuntivitis	1
2	10/10/2019	H16.9	1	Queratitis	1
1	10/10/2019	H43.81	7	DVP	1
2	10/10/2019	H16.9	1	Queratitis	1
2	10/10/2019	H16.9	1	Queratitis	1
2	10/10/2019	H27.8	2	OCP	1
1	10/10/2019	H16.0	1	Úlcera corneal	1
2	10/10/2019	H43.81	7	DVP	1
2	10/10/2019	H10.81	0	Pingueculitis	1
1	10/10/2019	H16.9	1	Queratitis	1
1	10/10/2019	T15.0	1	Cuerpo extraño	1
2	10/10/2019	H20	6	Uveitis	1
1	10/10/2019	H20	6	Uveitis	1
1	10/10/2019	H16.9	1	Queratitis	1
1	10/10/2019	H16.0	1	Úlcera corneal	1
2	10/10/2019	H17.8	1	Infiltrados corneales	1
1	10/10/2019	H10.81	0	Pingueculitis	1
2	10/10/2019	H16.0	1	Úlcera corneal	1
1	11/10/2019	T15.0	1	Cuerpo extraño	1
1	11/10/2019	H16.9	1	Queratitis	1
2	11/10/2019	H00.02	4	Orzuelo	1
1	11/10/2019	H10.3	0	Conjuntivitis	1
1	11/10/2019	H10.3	0	Conjuntivitis	1
2	11/10/2019	H16.0	1	Úlcera corneal	1
2	11/10/2019	H10.3	0	Conjuntivitis	1
2	11/10/2019	H10.3	0	Conjuntivitis	1
2	11/10/2019	H11.3	0	Hiposfagma	1
2	11/10/2019	H16.9	1	Queratitis	1
1	11/10/2019	H17.8	1	Infiltrados corneales	1
1	11/10/2019	H16.9	1	Queratitis	1
2	11/10/2019	H02	4	Lesión palpebral	1
2	11/10/2019	H10.3	0	Conjuntivitis	1
2	11/10/2019	H10.3	0	Conjuntivitis	1
1	11/10/2019	H53	11	No patología	1
2	11/10/2019	H00.03	4	Celulitis preseptal	1
1	11/10/2019	H27.8	2	OCP	1

2	11/10/2019	H33.30	8	Desgarro retiniano	1
2	11/10/2019	H20	6	Uveitis	1
1	11/10/2019	H16.9	1	Queratitis	1
2	11/10/2019	H10.3	0	Conjuntivitis	1
2	11/10/2019	H43.81	7	DVP	1
2	11/10/2019	H11.00	0	Pterigium	1
2	11/10/2019	H35.37	8	MER	1
1	11/10/2019	H00.02	4	Orzuelo	1
2	11/10/2019	H59.3	4	Post op párpados	1
2	11/10/2019	H10.3	0	Conjuntivitis	1
2	11/10/2019	H10.3	0	Conjuntivitis	1
1	11/10/2019	H10.3	0	Conjuntivitis	1
1	11/10/2019	H35.30	8	DMAE	1
1	11/10/2019	H16.9	1	Queratitis	1
2	11/10/2019	H16.9	1	Queratitis	1
2	11/10/2019	S05.6	9	Perforación ocular	1
2	11/10/2019	H16.9	1	Queratitis	1
2	11/10/2019	H43.81	7	DVP	1
1	11/10/2019	H16.9	1	Queratitis	1
2	11/10/2019	H00.02	4	Orzuelo	1
2	11/10/2019	H01.00	4	Blefaritis	1
1	11/10/2019	H15.00	6	Escleritis	1
2	11/10/2019	H10.81	0	Pingueculitis	1
2	11/10/2019	H43.81	7	DVP	1
2	11/10/2019	H10.3	0	Conjuntivitis	1
2	11/10/2019	H05.82	4	Trocleítis	1
2	11/10/2019	H43.81	7	DVP	1
2	11/10/2019	T26	9	Causticación	1
1	11/10/2019	H59.3	4	Post op párpados	1
2	11/10/2019	H16.9	1	Queratitis	1
2	11/10/2019	H10.3	0	Conjuntivitis	1
1	11/10/2019	H16.9	1	Queratitis	1
2	11/10/2019	S05.9	9	Traumatismo	1
2	11/10/2019	H20	6	Uveitis	1
1	11/10/2019	T15.0	1	Cuerpo extraño	1
1	12/10/2019	H16.9	1	Queratitis	1
1	12/10/2019	H10.3	0	Conjuntivitis	1
1	12/10/2019	H16.9	1	Queratitis	1
1	12/10/2019	H00.02	4	Orzuelo	1
2	12/10/2019	H11.3	0	Hiposfagma	1
2	12/10/2019	H26.9	2	Catarata	1
2	12/10/2019	H16.9	1	Queratitis	1
2	12/10/2019	S05.6	9	Perforación ocular	1
2	12/10/2019	H00.03	4	Celulitis preseptal	1
2	12/10/2019	H11.3	0	Hiposfagma	1
1	12/10/2019	H00.02	4	Orzuelo	1
2	12/10/2019	H10.3	0	Conjuntivitis	1
2	12/10/2019	H00.02	4	Orzuelo	1
2	12/10/2019	H43.81	7	DVP	1
1	12/10/2019	H10.3	0	Conjuntivitis	1
1	12/10/2019	H10.5	4	Blefaroconjuntivitis	1
1	12/10/2019	H16.0	1	Úlcera corneal	1
2	12/10/2019	H53	11	No patología	1
1	12/10/2019	H16.0	1	Úlcera corneal	1
2	12/10/2019	H04.32	5	Dacriocistitis aguda	1
1	12/10/2019	T15.0	1	Cuerpo extraño	1
1	12/10/2019	H35.3	8	Maculopatía	1
2	12/10/2019	H11.3	0	Hiposfagma	1
1	12/10/2019	H16.0	1	Úlcera corneal	1
1	12/10/2019	H00.02	4	Orzuelo	1
2	12/10/2019	Alta por fuga	11	Alta por fuga	1
2	12/10/2019	G43.9	10	Migraña	1
1	12/10/2019	S05.30	9	Laceración conjuntival	1
1	12/10/2019	H11.3	0	Hiposfagma	1
2	12/10/2019	H11.3	0	Hiposfagma	1
1	12/10/2019	T15.0	1	Cuerpo extraño	1

2	12/10/2019	H20	6	Uveitis	1
1	12/10/2019	H10.3	0	Conjuntivitis	1
1	12/10/2019	H10.3	0	Conjuntivitis	1
2	12/10/2019	H53	11	No patología	1
2	12/10/2019	H43.81	7	DVP	1
1	12/10/2019	T15.0	1	Cuerpo extraño	1
2	12/10/2019	H10.3	0	Conjuntivitis	1
2	12/10/2019	H11.3	0	Hiposfagma	1
2	12/10/2019	H10.3	0	Conjuntivitis	1
2	12/10/2019	H10.3	0	Conjuntivitis	1
2	12/10/2019	H33.30	8	Desgarro retiniano	1
2	12/10/2019	S05.9	9	Traumatismo	1
2	12/10/2019	H17.8	1	Infiltrados corneales	1
2	12/10/2019	T15.0	1	Cuerpo extraño	1
2	12/10/2019	H16.0	1	Úlcera corneal	1
1	12/10/2019	H11.3	0	Hiposfagma	1
1	12/10/2019	H16.0	1	Úlcera corneal	1
2	12/10/2019	H16.0	1	Úlcera corneal	1
2	13/10/2019	T15.0	1	Cuerpo extraño	1
2	13/10/2019	H10.3	0	Conjuntivitis	1
1	13/10/2019	H01.00	4	Blefaritis	1
2	13/10/2019	H10.3	0	Conjuntivitis	1
2	13/10/2019	H16.9	1	Queratitis	1
1	13/10/2019	H10.3	0	Conjuntivitis	1
1	13/10/2019	T15.0	1	Cuerpo extraño	1
2	13/10/2019	H02	4	Lesión palpebral	1
2	13/10/2019	H00.02	4	Orzuelo	1
2	13/10/2019	H10.3	0	Conjuntivitis	1
1	13/10/2019	B00.1	4	Dermatitis herpética	1
2	13/10/2019	H43.1	7	Hemovítreo	1
2	13/10/2019	H53	11	No patología	1
2	13/10/2019	H16.9	1	Queratitis	1
2	13/10/2019	H53.9	11	Alteraciones visuales	1
2	13/10/2019	H33.30	8	Desgarro retiniano	1
1	13/10/2019	H10.3	0	Conjuntivitis	1
1	13/10/2019	H00.02	4	Orzuelo	1
2	13/10/2019	H02	4	Lesión palpebral	1
1	13/10/2019	S05.9	9	Traumatismo	1
2	13/10/2019	H00.02	4	Orzuelo	1
2	13/10/2019	H10.3	0	Conjuntivitis	1
2	13/10/2019	H10.3	0	Conjuntivitis	1
2	13/10/2019	H10.3	0	Conjuntivitis	1
1	13/10/2019	G43.9	10	Migraña	1
2	13/10/2019	H16.9	1	Queratitis	1
1	13/10/2019	H01.00	4	Blefaritis	1
2	13/10/2019	H10.81	0	Pingueculitis	1
2	13/10/2019	H00.02	4	Orzuelo	1
1	13/10/2019	H16.0	1	Úlcera corneal	1
2	13/10/2019	H10.3	0	Conjuntivitis	1
1	13/10/2019	S05.9	9	Traumatismo	1
1	13/10/2019	H11.3	0	Hiposfagma	1
2	13/10/2019	H16.9	1	Queratitis	1
1	13/10/2019	S05.6	9	Perforación ocular	1
1	13/10/2019	H16.0	1	Úlcera corneal	1
2	13/10/2019	H10.3	0	Conjuntivitis	1
1	13/10/2019	H16.0	1	Úlcera corneal	1
2	13/10/2019	H16.9	1	Queratitis	1
1	13/10/2019	S05.9	9	Traumatismo	1
2	13/10/2019	H33.0	8	Desprendimiento de retina	1
2	14/10/2019	H11.3	0	Hiposfagma	1
2	14/10/2019	H15.1	6	Epiescleritis	1
2	14/10/2019	H16.9	1	Queratitis	1
2	14/10/2019	H02	4	Lesión palpebral	1
2	14/10/2019	H11.3	0	Hiposfagma	1
2	14/10/2019	H0.41	1	Ojo seco	1
2	14/10/2019	H10.3	0	Conjuntivitis	1

2	14/10/2019	H10.3	0	Conjuntivitis	1
1	14/10/2019	H10.3	0	Conjuntivitis	1
2	14/10/2019	H00.02	4	Orzuelo	1
2	14/10/2019	H10.3	0	Conjuntivitis	1
1	14/10/2019	H35.3	8	Maculopatía	1
2	14/10/2019	H35.3	8	Maculopatía	1
2	14/10/2019	H0.41	1	Ojo seco	1
2	14/10/2019	H20	6	Uveitis	1
1	14/10/2019	H53	11	No patología	1
2	14/10/2019	H20	6	Uveitis	1
2	14/10/2019	H10.3	0	Conjuntivitis	1
1	14/10/2019	H20	6	Uveitis	1
2	14/10/2019	H02	4	Lesión palpebral	1
2	14/10/2019	H10.3	0	Conjuntivitis	1
1	14/10/2019	H16.9	1	Queratitis	1
1	14/10/2019	H10.3	0	Conjuntivitis	1
1	14/10/2019	S05.9	9	Traumatismo	1
1	14/10/2019	T15.0	1	Cuerpo extraño	1
2	14/10/2019	H10.3	0	Conjuntivitis	1
1	14/10/2019	H10.3	0	Conjuntivitis	1
1	14/10/2019	H10.3	0	Conjuntivitis	1
2	14/10/2019	H10.3	0	Conjuntivitis	1
2	14/10/2019	H01.00	4	Blefaritis	1
2	14/10/2019	H16.0	1	Úlcera corneal	1
1	14/10/2019	H43.81	7	DVP	1
2	14/10/2019	G43.109	10	Aura	1
2	14/10/2019	H53	11	No patología	1
2	14/10/2019	H43.81	7	DVP	1
1	14/10/2019	H43.1	7	Hemovítreo	1
2	14/10/2019	H35.0	8	Hemorragia retiniana	1
2	14/10/2019	H43.1	7	Hemovítreo	1
2	14/10/2019	H01.00	4	Blefaritis	1
2	14/10/2019	H10.3	0	Conjuntivitis	1
1	14/10/2019	H16.0	1	Úlcera corneal	1
2	14/10/2019	H16.0	1	Úlcera corneal	1
2	14/10/2019	S05.30	9	Laceración conjuntival	1
2	14/10/2019	H16.9	1	Queratitis	1
1	14/10/2019	H10.3	0	Conjuntivitis	1
2	14/10/2019	H16.9	1	Queratitis	1
1	14/10/2019	H43.81	7	DVP	1
2	14/10/2019	H10.3	0	Conjuntivitis	1
2	14/10/2019	G43.9	10	Migraña	1
2	14/10/2019	H16.9	1	Queratitis	1
2	14/10/2019	H0.41	1	Ojo seco	1
1	14/10/2019	T15.0	1	Cuerpo extraño	1
1	14/10/2019	H10.3	0	Conjuntivitis	1
2	14/10/2019	H43.81	7	DVP	1
1	14/10/2019	H34.82	8	Trombosis venosa	1
1	14/10/2019	S05.9	9	Traumatismo	1
2	14/10/2019	H10.3	0	Conjuntivitis	1
2	14/10/2019	H20	6	Uveitis	1
2	14/10/2019	H16.9	1	Queratitis	1
2	14/10/2019	H16.9	1	Queratitis	1
1	14/10/2019	H16.9	1	Queratitis	1
2	14/10/2019	H01.00	4	Blefaritis	1
1	14/10/2019	H53	11	No patología	1
1	14/10/2019	H00.03	4	Celulitis preseptal	1
2	14/10/2019	S05.9	9	Traumatismo	1
2	14/10/2019	H43.81	7	DVP	1
1	14/10/2019	H16.0	1	Úlcera corneal	1
2	14/10/2019	H16.0	1	Úlcera corneal	1
2	14/10/2019	H16.9	1	Queratitis	1
2	14/10/2019	H43.81	7	DVP	1
1	14/10/2019	H16.0	1	Úlcera corneal	1
2	15/10/2019	S05.9	9	Traumatismo	1
2	15/10/2019	S05.9	9	Traumatismo	1

1	15/10/2019	S05.9	9	Traumatismo	1
1	15/10/2019	H53	11	No patología	1
2	15/10/2019	H43.81	7	DVP	1
2	15/10/2019	H59.3	5	Postop via lagrimal	1
1	15/10/2019	H16.0	1	Úlcera corneal	1
2	15/10/2019	H53	11	No patología	1
2	15/10/2019	H04.32	5	Dacriocistitis aguda	1
1	15/10/2019	H00.02	4	Orzuelo	1
1	15/10/2019	H53	11	No patología	1
2	15/10/2019	H10.3	0	Conjuntivitis	1
2	15/10/2019	H43.81	7	DVP	1
1	15/10/2019	H43.81	7	DVP	1
1	15/10/2019	H20	6	Uveitis	1
2	15/10/2019	H16.0	1	Úlcera corneal	1
1	15/10/2019	H16.0	1	Úlcera corneal	1
2	15/10/2019	H10.3	0	Conjuntivitis	1
2	15/10/2019	H11.3	0	Hiposfagma	1
2	15/10/2019	H43.81	7	DVP	1
2	15/10/2019	H10.3	0	Conjuntivitis	1
1	15/10/2019	H16.0	1	Úlcera corneal	1
1	15/10/2019	S05.9	9	Traumatismo	1
1	15/10/2019	H10.3	0	Conjuntivitis	1
1	15/10/2019	T15.0	1	Cuerpo extraño	1
1	15/10/2019	H26.9	2	Catarata	1
2	15/10/2019	H16.0	1	Úlcera corneal	1
2	15/10/2019	H43.81	7	DVP	1
1	15/10/2019	H10.3	0	Conjuntivitis	1
2	15/10/2019	H11.3	0	Hiposfagma	1
2	15/10/2019	H16.9	1	Queratitis	1
1	15/10/2019	H16.9	1	Queratitis	1
2	15/10/2019	S05.30	9	Laceración conjuntival	1
1	15/10/2019	H00.02	4	Orzuelo	1
2	15/10/2019	S05.9	9	Traumatismo	1
1	15/10/2019	H16.0	1	Úlcera corneal	1
2	15/10/2019	H16.9	1	Queratitis	1
1	15/10/2019	H49.9	10	Parálisis oculomotora	1
1	15/10/2019	H16.0	1	Úlcera corneal	1
1	15/10/2019	H16.0	1	Úlcera corneal	1
1	15/10/2019	H46	10	Neuritis óptica	1
2	16/10/2019	H16.0	1	Úlcera corneal	1
1	16/10/2019	T15.0	1	Cuerpo extraño	1
2	16/10/2019	H43.81	7	DVP	1
2	16/10/2019	H16.9	1	Queratitis	1
2	16/10/2019	H10.3	0	Conjuntivitis	1
2	16/10/2019	H43.81	7	DVP	1
2	16/10/2019	H16.9	1	Queratitis	1
2	16/10/2019	H16.9	1	Queratitis	1
2	16/10/2019	H00.02	4	Orzuelo	1
2	16/10/2019	H01.00	4	Blefaritis	1
1	16/10/2019	H10.3	0	Conjuntivitis	1
1	16/10/2019	H16.9	1	Queratitis	1
2	16/10/2019	H10.3	0	Conjuntivitis	1
1	16/10/2019	H18.50	1	Distrofia corneal	1
1	16/10/2019	H10.3	0	Conjuntivitis	1
1	16/10/2019	H40.9	3	Glaucoma	1
2	16/10/2019	H11.00	0	Pterigium	1
1	16/10/2019	H01.00	4	Blefaritis	1
2	16/10/2019	H53	11	No patología	1
2	16/10/2019	Alta por fuga	11	Alta por fuga	1
1	16/10/2019	H16.0	1	Úlcera corneal	1
2	16/10/2019	H26.9	2	Catarata	1
2	16/10/2019	H01.00	4	Blefaritis	1
2	16/10/2019	T15.0	1	Cuerpo extraño	1
1	16/10/2019	H43.81	7	DVP	1
1	16/10/2019	H16.0	1	Úlcera corneal	1
2	16/10/2019	H01.00	4	Blefaritis	1

2	16/10/2019	H01.00	4	Blefaritis	1
2	16/10/2019	H11.00	0	Pterigium	1
2	16/10/2019	S05.30	9	Laceración conjuntival	1
2	16/10/2019	H16.9	1	Queratitis	1
2	16/10/2019	H01.00	4	Blefaritis	1
2	16/10/2019	H26.9	2	Catarata	1
2	16/10/2019	H11.3	0	Hiposfagma	1
1	16/10/2019	H02	4	Lesión palpebral	1
2	16/10/2019	H01.00	4	Blefaritis	1
2	16/10/2019	H02.05	4	Distiquiasis	1
1	16/10/2019	H02	4	Alteración palpebral	1
1	16/10/2019	T15.0	1	Cuerpo extraño	1
2	16/10/2019	H11.3	0	Hiposfagma	1
2	16/10/2019	H43.81	7	DVP	1
1	16/10/2019	H16.0	1	Úlcera corneal	1
2	16/10/2019	H16.9	1	Queratitis	1
2	16/10/2019	H43.81	7	DVP	1
2	16/10/2019	H16.0	1	Úlcera corneal	1
2	16/10/2019	H15.1	6	Epiescleritis	1
1	16/10/2019	H16.9	1	Queratitis	1
1	16/10/2019	H43.81	7	DVP	1
1	16/10/2019	H16.0	1	Úlcera corneal	1
1	16/10/2019	H00.03	4	Celulitis preseptal	1
2	16/10/2019	H59.88	8	Post op retina	1
1	16/10/2019	S05.9	9	Traumatismo	1
1	17/10/2019	H16.9	1	Queratitis	1
1	17/10/2019	H10.3	0	Conjuntivitis	1
1	17/10/2019	H17.8	1	Infiltrados corneales	1
1	17/10/2019	T15.0	1	Cuerpo extraño	1
2	17/10/2019	H43.81	7	DVP	1
2	17/10/2019	H16.0	1	Úlcera corneal	1
2	17/10/2019	H10.3	0	Conjuntivitis	1
2	17/10/2019	H00.02	4	Orzuelo	1
2	17/10/2019	H16.9	1	Queratitis	1
1	17/10/2019	H18.20	1	Edema corneal	1
2	17/10/2019	H16.9	1	Queratitis	1
2	17/10/2019	H16.0	1	Úlcera corneal	1
1	17/10/2019	H11.3	0	Hiposfagma	1
2	17/10/2019	H16.9	1	Queratitis	1
1	17/10/2019	H10.3	0	Conjuntivitis	1
2	17/10/2019	H16.0	1	Úlcera corneal	1
2	17/10/2019	H16.0	1	Úlcera corneal	1
2	17/10/2019	H16.9	1	Queratitis	1
2	17/10/2019	G43.9	10	Migraña	1
2	17/10/2019	S05.30	9	Laceración conjuntival	1
2	17/10/2019	H16.0	1	Úlcera corneal	1
1	17/10/2019	S05.6	9	Perforación ocular	1
1	17/10/2019	H35.3	8	Maculopatía	1
1	17/10/2019	H16.0	1	Úlcera corneal	1
1	17/10/2019	H16.9	1	Queratitis	1
2	17/10/2019	H53	11	No patología	1
1	17/10/2019	H10.3	0	Conjuntivitis	1
1	17/10/2019	T15.0	1	Cuerpo extraño	1
2	17/10/2019	H20	6	Uveitis	1
1	17/10/2019	H16.0	1	Úlcera corneal	1
1	17/10/2019	H33.30	8	Desgarro retiniano	1
2	17/10/2019	H00.02	4	Orzuelo	1
1	17/10/2019	H16.0	1	Úlcera corneal	1
2	17/10/2019	H11.3	0	Hiposfagma	1
2	18/10/2019	H16.0	1	Úlcera corneal	1
2	18/10/2019	H16.0	1	Úlcera corneal	1
1	18/10/2019	H10.3	0	Conjuntivitis	1
1	18/10/2019	H18.20	1	Edema corneal	1
2	18/10/2019	H01.00	4	Blefaritis	1
1	18/10/2019	H16.9	1	Queratitis	1
1	18/10/2019	H26.9	2	Catarata	1

2	18/10/2019	H10.5		4	Blefaroconjuntivitis	1
2	18/10/2019	H0.41		1	Ojo seco	1
2	18/10/2019	H53		11	No patología	1
1	18/10/2019	S05.9		11	Traumatismo	1
1	18/10/2019	H01.00		4	Blefaritis	1
2	18/10/2019	H43.81		7	DVP	1
1	18/10/2019	H16.9		1	Queratitis	1
1	18/10/2019	H11.3		0	Hiposfagma	1
2	18/10/2019	S05.30		9	Laceración conjuntival	1
2	18/10/2019	H35.30		8	DMAE	1
1	18/10/2019	S05.9		9	Traumatismo	1
1	18/10/2019	H16.0		1	Úlcera corneal	1
2	18/10/2019	H16.0		1	Úlcera corneal	1
1	18/10/2019	H10.3		0	Conjuntivitis	1
2	18/10/2019	G43.9		10	Migraña	1
1	18/10/2019	H16.0		1	Úlcera corneal	1
1	18/10/2019	T15.0		1	Cuerpo extraño	1
1	18/10/2019	H10.3		0	Conjuntivitis	1
1	18/10/2019	H53		11	No patología	1
1	18/10/2019	H17.8		1	Infiltrados corneales	1
1	18/10/2019	H16.9		1	Queratitis	1
1	18/10/2019	T15.0		1	Cuerpo extraño	1
2	18/10/2019	H10.3		0	Conjuntivitis	1
2	18/10/2019	H16.0		1	Úlcera corneal	1
2	18/10/2019	H53		11	No patología	1
2	18/10/2019	H10.3		0	Conjuntivitis	1
2	18/10/2019	H01.00		4	Blefaritis	1
1	18/10/2019	H26.9		2	Catarata	1
2	18/10/2019	H16.9		1	Queratitis	1
2	18/10/2019	H01.00		4	Blefaritis	1
1	18/10/2019	H11.3		0	Hiposfagma	1
2	18/10/2019	H53		11	No patología	1
1	18/10/2019	T15.0		1	Cuerpo extraño	1
2	18/10/2019	H11.00		0	Pterigium	1
1	18/10/2019	H16.0		1	Úlcera corneal	1
1	18/10/2019	H16.9		1	Queratitis	1
1	18/10/2019	H43.81		7	DVP	1
1	18/10/2019	H53		11	No patología	1
1	18/10/2019	H16.0		1	Úlcera corneal	1
1	18/10/2019	H00.19		4	Chalazión	1
2	18/10/2019	H00.19		4	Chalazión	1
1	18/10/2019	H16.9		1	Queratitis	1
2	18/10/2019	H16.0		1	Úlcera corneal	1
1	18/10/2019	H53		11	No patología	1
2	18/10/2019	T15.0		1	Cuerpo extraño	1
1	18/10/2019	T15.0		1	Cuerpo extraño	1
1	18/10/2019	H20		6	Uveitis	1
2	18/10/2019	H10.3		0	Conjuntivitis	1
1	18/10/2019	T15.0		1	Cuerpo extraño	1
1	18/10/2019	H16.9		1	Queratitis	1
1	18/10/2019	H16.0		1	Úlcera corneal	1
1	18/10/2019	H16.0		1	Úlcera corneal	1
2	18/10/2019	T26		9	Causticación	1
1	18/10/2019	T15.0		1	Cuerpo extraño	1
2	19/10/2019	H16.0		1	Úlcera corneal	1
1	19/10/2019	H10.3		0	Conjuntivitis	1
2	19/10/2019	H53		11	No patología	1
1	19/10/2019	H10.3		0	Conjuntivitis	1
1	19/10/2019	H11.3		0	Hiposfagma	1
2	19/10/2019	H00.03		4	Celulitis preseptal	1
1	19/10/2019	H10.3		0	Conjuntivitis	1
1	19/10/2019	T15.0		1	Cuerpo extraño	1
2	19/10/2019	H02		4	Lesión palpebral	1
2	19/10/2019	H43.81		7	DVP	1
1	19/10/2019	H20		6	Uveitis	1
2	19/10/2019	H0.41		1	Ojo seco	1

2	19/10/2019	H10.5	4	Blefaroconjuntivitis	1
1	19/10/2019	H11.3	0	Hiposfagma	1
2	19/10/2019	H40.05	3	HTO	1
1	19/10/2019	H02	4	Lesión palpebral	1
1	19/10/2019	H20	6	Uveitis	1
1	19/10/2019	H16.9	1	Queratitis	1
2	19/10/2019	H10.3	0	Conjuntivitis	1
1	19/10/2019	H16.9	1	Queratitis	1
2	19/10/2019	H15.00	6	Escleritis	1
2	19/10/2019	H00.02	4	Orzuelo	1
1	19/10/2019	H10.3	0	Conjuntivitis	1
1	19/10/2019	H11.3	0	Hiposfagma	1
2	19/10/2019	H0.41	1	Ojo seco	1
1	19/10/2019	H31.42	8	Coroidopatía serosa central	1
1	19/10/2019	H16.0	1	Úlcera corneal	1
1	19/10/2019	H20	6	Uveitis	1
2	19/10/2019	H27.10	2	Luxación LIO	1
1	19/10/2019	H16.9	1	Queratitis	1
2	19/10/2019	H16.9	1	Queratitis	1
2	19/10/2019	H0.41	1	Ojo seco	1
1	19/10/2019	H11.3	0	Hiposfagma	1
1	19/10/2019	S05.9	9	Traumatismo	1
1	19/10/2019	H16.0	1	Úlcera corneal	1
2	19/10/2019	H04.32	5	Dacriocistitis aguda	1
1	19/10/2019	H16.9	1	Queratitis	1
2	19/10/2019	H43.81	7	DVP	1
2	19/10/2019	H16.0	1	Úlcera corneal	1
1	19/10/2019	H43.81	7	DVP	1
1	19/10/2019	H10.3	0	Conjuntivitis	1
1	19/10/2019	H11.00	0	Pterigium	1
1	19/10/2019	H53	11	No patología	1
1	20/10/2019	H16.9	1	Queratitis	1
1	20/10/2019	H16.0	1	Úlcera corneal	1
1	20/10/2019	H53	11	No patología	1
1	20/10/2019	S05.9	9	Traumatismo	1
1	20/10/2019	S05.9	9	Traumatismo	1
1	20/10/2019	H16.0	1	Úlcera corneal	1
2	20/10/2019	H20	6	Uveitis	1
1	20/10/2019	H31.42	8	Coroidopatía serosa central	1
1	20/10/2019	H17.8	1	Infiltrados corneales	1
1	20/10/2019	H10.3	0	Conjuntivitis	1
2	20/10/2019	H16.0	1	Úlcera corneal	1
2	20/10/2019	H11.3	0	Hiposfagma	1
2	20/10/2019	H16.9	1	Queratitis	1
2	20/10/2019	H53	11	No patología	1
2	20/10/2019	H16.0	1	Úlcera corneal	1
2	20/10/2019	H00.02	4	Orzuelo	1
1	20/10/2019	H16.0	1	Úlcera corneal	1
1	20/10/2019	T15.0	1	Cuerpo extraño	1
1	20/10/2019	H33.30	8	Desgarro retiniano	1
2	20/10/2019	H16.9	1	Queratitis	1
1	20/10/2019	H16.9	1	Queratitis	1
2	20/10/2019	H10.3	0	Conjuntivitis	1
1	20/10/2019	H20	6	Uveitis	1
2	20/10/2019	H11.3	0	Hiposfagma	1
2	20/10/2019	H11.3	0	Hiposfagma	1
2	20/10/2019	S05.9	9	Traumatismo	1
1	20/10/2019	H16.0	1	Úlcera corneal	1
2	20/10/2019	H43.1	7	Hemovítreo	1
1	20/10/2019	H10.3	0	Conjuntivitis	1
1	20/10/2019	S05.30	9	Laceración conjuntival	1
1	20/10/2019	B00.1	4	Dermatitis herpética	1
2	20/10/2019	S05.9	9	Traumatismo	1
1	20/10/2019	H16.9	1	Queratitis	1
2	20/10/2019	H10.3	0	Conjuntivitis	1
2	20/10/2019	H10.3	0	Conjuntivitis	1

2	20/10/2019	H10.3	0	Conjuntivitis		1
1	20/10/2019	H59.3	4	Post op párpados		1
2	20/10/2019	H16.0	1	Úlcera corneal		1
2	20/10/2019	H10.3	0	Conjuntivitis		1
2	20/10/2019	H11.44	0	Quiste conjuntival		1
2	20/10/2019	H16.0	1	Úlcera corneal		1
1	20/10/2019	T15.0	1	Cuerpo extraño		1
2	20/10/2019	H10.81	0	Pingueculitis		1
2	20/10/2019	G43.109	10	Aura		1
1	20/10/2019	H27.8	2	OCP		1
2	20/10/2019	H16.0	1	Úlcera corneal		1
1	20/10/2019	S05.9	9	Traumatismo		1
2	20/10/2019	S05.9	9	Traumatismo		1
2	20/10/2019	H16.0	1	Úlcera corneal		1
1	20/10/2019	H53	11	No patología		1
2	20/10/2019	H16.0	1	Úlcera corneal		1
1	21/10/2019	H16.9	1	Queratitis		1
1	21/10/2019	Alta por fuga	11	Alta por fuga		1
2	21/10/2019	H16.9	1	Queratitis		1
2	21/10/2019	H0.41	1	Ojo seco		1
1	21/10/2019	H53	11	No patología		1
1	21/10/2019	H31.42	8	Coroidopatía serosa central		1
2	21/10/2019	H11.3	0	Hiposfagma		1
2	21/10/2019	H35.30	8	DMAE		1
2	21/10/2019	H16.9	1	Queratitis		1
2	21/10/2019	H20	6	Uveitis		1
2	21/10/2019	H16.0	1	Úlcera corneal		1
2	21/10/2019	H15.1	6	Epiescleritis		1
1	21/10/2019	H53.9	11	Alteraciones visuales		1
2	21/10/2019	H16.0	1	Úlcera corneal		1
1	21/10/2019	H43.81	7	DVP		1
2	21/10/2019	H33.30	8	Desgarro retiniano		1
1	21/10/2019	H53.9	11	Alteraciones visuales		1
1	21/10/2019	H0.41	1	Ojo seco		1
1	21/10/2019	T15.0	1	Cuerpo extraño		1
1	21/10/2019	H40.05	3	HTO		1
1	21/10/2019	H00.02	4	Orzuelo		1
1	21/10/2019	H43.81	7	DVP		1
1	21/10/2019	H01.00	4	Blefaritis		1
1	21/10/2019	H20	6	Uveitis		1
2	21/10/2019	H01.00	4	Blefaritis		1
2	21/10/2019	H16.9	1	Queratitis		1
2	21/10/2019	T15.0	1	Cuerpo extraño		1
1	21/10/2019	H11.3	0	Hiposfagma		1
1	21/10/2019	H15.00	6	Escleritis		1
2	21/10/2019	H11.44	0	Quiste conjuntival		1
2	21/10/2019	H16.0	1	Úlcera corneal		1
2	21/10/2019	H16.0	1	Úlcera corneal		1
1	21/10/2019	H16.0	1	Úlcera corneal		1
1	21/10/2019	H43.81	7	DVP		1
2	21/10/2019	S05.9	9	Traumatismo		1
2	21/10/2019	H00.02	4	Orzuelo		1
1	21/10/2019	H43.81	7	DVP		1
2	21/10/2019	H10.3	0	Conjuntivitis		1
2	21/10/2019	H43.81	7	DVP		1
1	21/10/2019	H16.0	1	Úlcera corneal		1
1	21/10/2019	H17.8	1	Infiltrados corneales		1
1	21/10/2019	H16.9	1	Queratitis		1
2	21/10/2019	H16.9	1	Queratitis		1
2	21/10/2019	H10.3	0	Conjuntivitis		1
1	21/10/2019	H16.0	1	Úlcera corneal		1
2	21/10/2019	H16.0	1	Úlcera corneal		1
1	21/10/2019	H01.00	4	Blefaritis		1
2	21/10/2019	H16.9	1	Queratitis		1
2	21/10/2019	H00.02	4	Orzuelo		1
2	22/10/2019	H11.3	0	Hiposfagma		1

2	22/10/2019	H01.00	4	Blefaritis	1
2	22/10/2019	H16.9	1	Queratitis	1
2	22/10/2019	Z98.4	2	Post op catarata	1
2	22/10/2019	H10.81	0	Pingueculitis	1
1	22/10/2019	H16.9	1	Queratitis	1
1	22/10/2019	H10.3	0	Conjuntivitis	1
2	22/10/2019	H01.00	4	Blefaritis	1
1	22/10/2019	H53.10	11	Problema refractivo	1
1	22/10/2019	H01.00	4	Blefaritis	1
2	22/10/2019	H11.3	0	Hiposfagma	1
1	22/10/2019	H53	11	No patología	1
2	22/10/2019	H16.9	1	Queratitis	1
1	22/10/2019	H16.0	1	Úlcera corneal	1
1	22/10/2019	H02	4	Alteración palpebral	1
1	22/10/2019	H16.9	1	Queratitis	1
1	22/10/2019	H16.9	1	Queratitis	1
1	22/10/2019	H40.21	3	Glaucoma agudo	1
1	22/10/2019	H16.9	1	Queratitis	1
2	22/10/2019	H16.9	1	Queratitis	1
2	22/10/2019	H16.0	1	Úlcera corneal	1
2	22/10/2019	H16.9	1	Queratitis	1
2	22/10/2019	T15.0	1	Cuerpo extraño	1
1	22/10/2019	H43.81	7	DVP	1
2	22/10/2019	H11.3	0	Hiposfagma	1
2	22/10/2019	H33.0	8	Desprendimiento de retina	1
2	22/10/2019	H43.81	7	DVP	1
1	22/10/2019	H20	6	Uveitis	1
1	22/10/2019	H10.3	0	Conjuntivitis	1
1	22/10/2019	H17.8	1	Infiltrados corneales	1
1	22/10/2019	H00.02	4	Orzuelo	1
2	22/10/2019	H10.3	0	Conjuntivitis	1
1	22/10/2019	T15.0	1	Cuerpo extraño	1
2	22/10/2019	H53.9	11	Alteraciones visuales	1
1	22/10/2019	H00.02	4	Orzuelo	1
2	22/10/2019	H11.3	0	Hiposfagma	1
1	22/10/2019	H16.0	1	Úlcera corneal	1
2	22/10/2019	H16.9	1	Queratitis	1
2	22/10/2019	H10.3	0	Conjuntivitis	1
1	22/10/2019	S05.30	9	Laceración conjuntival	1
1	22/10/2019	S05.9	9	Traumatismo	1
2	22/10/2019	H11.3	0	Hiposfagma	1
1	23/10/2019	H16.0	1	Úlcera corneal	1
1	23/10/2019	H16.0	1	Úlcera corneal	1
2	23/10/2019	H16.0	1	Úlcera corneal	1
2	23/10/2019	H04.32	5	Dacriocistitis aguda	1
1	23/10/2019	H10.3	0	Conjuntivitis	1
1	23/10/2019	H43.81	7	DVP	1
2	23/10/2019	H10.3	0	Conjuntivitis	1
2	23/10/2019	Alta por fuga	11	Alta por fuga	1
2	23/10/2019	H15.1	6	Epiescleritis	1
1	23/10/2019	H10.3	0	Conjuntivitis	1
1	23/10/2019	H16.0	1	Úlcera corneal	1
2	23/10/2019	H43.81	7	DVP	1
2	23/10/2019	H16.9	1	Queratitis	1
2	23/10/2019	H00.19	4	Chalazión	1
2	23/10/2019	H10.3	0	Conjuntivitis	1
2	23/10/2019	H16.9	1	Queratitis	1
1	23/10/2019	H02	4	Alteración palpebral	1
2	23/10/2019	H16.9	1	Queratitis	1
2	23/10/2019	H00.02	4	Orzuelo	1
2	23/10/2019	H43.81	7	DVP	1
1	23/10/2019	H27.8	2	OCP	1
2	23/10/2019	H43.81	7	DVP	1
1	23/10/2019	H43.81	7	DVP	1
2	23/10/2019	H16.0	1	Úlcera corneal	1
2	23/10/2019	H11.3	0	Hiposfagma	1

2	23/10/2019	H16.9	1	Queratitis	1
1	23/10/2019	H16.9	1	Queratitis	1
1	23/10/2019	H10.3	0	Conjuntivitis	1
2	23/10/2019	H11.3	0	Hiposfagma	1
1	23/10/2019	H27.8	2	OCP	1
1	23/10/2019	H35.30	8	DMAE	1
1	23/10/2019	H10.3	0	Conjuntivitis	1
1	23/10/2019	H10.3	0	Conjuntivitis	1
2	23/10/2019	H53	11	No patología	1
1	23/10/2019	H20	6	Uveitis	1
2	23/10/2019	H16.0	1	Úlcera corneal	1
1	23/10/2019	H43.81	7	DVP	1
1	23/10/2019	T15.0	1	Cuerpo extraño	1
1	23/10/2019	H16.9	1	Queratitis	1
2	23/10/2019	H16.0	1	Úlcera corneal	1
1	23/10/2019	H16.0	1	Úlcera corneal	1
1	23/10/2019	H44.009	8	Endoftalmitis	1
1	23/10/2019	H33.30	8	Desgarro retiniano	1
1	23/10/2019	H16.0	1	Úlcera corneal	1
2	24/10/2019	T26	9	Causticación	1
1	24/10/2019	H10.3	0	Conjuntivitis	1
2	24/10/2019	H16.9	1	Queratitis	1
1	24/10/2019	H10.3	0	Conjuntivitis	1
2	24/10/2019	H40.21	3	Glaucoma agudo	1
2	24/10/2019	H00.03	4	Celulitis preseptal	1
1	24/10/2019	H20	6	Uveitis	1
2	24/10/2019	H43.81	7	DVP	1
2	24/10/2019	H59.88	8	Post op retina	1
2	24/10/2019	H34.82	8	Trombosis venosa	1
2	24/10/2019	H16.9	1	Queratitis	1
2	24/10/2019	H0.41	1	Ojo seco	1
1	24/10/2019	H20	6	Uveitis	1
2	24/10/2019	H10.3	0	Conjuntivitis	1
2	24/10/2019	H0.41	1	Ojo seco	1
1	24/10/2019	H0.41	1	Ojo seco	1
1	24/10/2019	H40.5	3	Glaucoma secundario	1
1	24/10/2019	H53	11	No patología	1
2	24/10/2019	H02	4	Lesión palpebral	1
1	24/10/2019	H16.9	1	Queratitis	1
2	24/10/2019	H16.9	1	Queratitis	1
2	24/10/2019	H20	6	Uveitis	1
2	24/10/2019	H43.81	7	DVP	1
2	24/10/2019	H20	6	Uveitis	1
2	24/10/2019	H53	11	No patología	1
2	24/10/2019	H43.81	7	DVP	1
2	24/10/2019	H10.3	0	Conjuntivitis	1
2	24/10/2019	H20	6	Uveitis	1
2	24/10/2019	H10.3	0	Conjuntivitis	1
2	24/10/2019	H11.3	0	Hiposfagma	1
2	24/10/2019	H43.81	7	DVP	1
2	24/10/2019	H53	11	No patología	1
2	24/10/2019	H00.02	4	Orzuelo	1
2	24/10/2019	H16.9	1	Queratitis	1
2	24/10/2019	H10.3	0	Conjuntivitis	1
1	24/10/2019	H27.8	2	OCP	1
1	24/10/2019	H16.9	1	Queratitis	1
1	24/10/2019	H11.3	0	Hiposfagma	1
2	24/10/2019	H27.8	2	OCP	1
2	24/10/2019	H11.3	0	Hiposfagma	1
2	24/10/2019	S05.9	9	Traumatismo	1
1	24/10/2019	G43.9	10	Migraña	1
2	24/10/2019	H17.8	1	Infiltrados corneales	1
1	24/10/2019	H16.9	1	Queratitis	1
2	24/10/2019	H53	11	No patología	1
1	24/10/2019	T15.0	1	Cuerpo extraño	1
1	24/10/2019	H11.3	0	Hiposfagma	1

2	24/10/2019	H59.3	4	Post op párpados	1
1	24/10/2019	H10.3	0	Conjuntivitis	1
1	24/10/2019	H10.3	0	Conjuntivitis	1
2	24/10/2019	H35.30	8	DMAE	1
1	24/10/2019	T15.0	1	Cuerpo extraño	1
2	24/10/2019	H10.81	0	Pingueculitis	1
2	24/10/2019	S05.9	9	Traumatismo	1
1	24/10/2019	H16.0	1	Úlcera corneal	1
1	25/10/2019	H10.3	0	Conjuntivitis	1
2	25/10/2019	H10.3	0	Conjuntivitis	1
2	25/10/2019	H53	11	No patología	1
2	25/10/2019	H10.3	0	Conjuntivitis	1
1	25/10/2019	H10.3	0	Conjuntivitis	1
2	25/10/2019	H16.0	1	Úlcera corneal	1
2	25/10/2019	H10.3	0	Conjuntivitis	1
2	25/10/2019	H53	11	No patología	1
2	25/10/2019	H53	11	No patología	1
2	25/10/2019	H10.3	0	Conjuntivitis	1
2	25/10/2019	H01.00	4	Blefaritis	1
1	25/10/2019	H10.3	0	Conjuntivitis	1
1	25/10/2019	H16.9	1	Queratitis	1
2	25/10/2019	H00.02	4	Orzuelo	1
2	25/10/2019	H53	11	No patología	1
2	25/10/2019	H10.3	0	Conjuntivitis	1
1	25/10/2019	H35.0	8	Hemorragia retiniana	1
1	25/10/2019	H10.3	0	Conjuntivitis	1
1	25/10/2019	B00.1	4	Dermatitis herpética	1
2	25/10/2019	H01.00	4	Blefaritis	1
2	25/10/2019	H53	11	No patología	1
2	25/10/2019	H10.3	0	Conjuntivitis	1
1	25/10/2019	H01.00	4	Blefaritis	1
1	25/10/2019	H10.3	0	Conjuntivitis	1
2	25/10/2019	H10.3	0	Conjuntivitis	1
1	25/10/2019	H16.9	1	Queratitis	1
2	25/10/2019	H43.81	7	DVP	1
2	25/10/2019	H16.0	1	Úlcera corneal	1
2	25/10/2019	H16.0	1	Úlcera corneal	1
2	25/10/2019	T15.0	1	Cuerpo extraño	1
1	25/10/2019	H16.9	1	Queratitis	1
2	25/10/2019	H10.3	0	Conjuntivitis	1
1	25/10/2019	H16.0	1	Úlcera corneal	1
2	25/10/2019	H53	11	No patología	1
1	25/10/2019	H16.0	1	Úlcera corneal	1
1	25/10/2019	H18.20	1	Edema corneal	1
1	25/10/2019	H10.3	0	Conjuntivitis	1
2	25/10/2019	H16.9	1	Queratitis	1
1	25/10/2019	H50.9	10	Estrabismo	1
1	25/10/2019	Alta por fuga	11	Alta por fuga	1
1	25/10/2019	S05.30	9	Laceración conjuntival	1
2	25/10/2019	H10.3	0	Conjuntivitis	1
2	25/10/2019	H53	11	No patología	1
1	25/10/2019	H01.00	4	Blefaritis	1
1	26/10/2019	H16.0	1	Úlcera corneal	1
1	26/10/2019	S05.9	9	Traumatismo	1
2	26/10/2019	H27.10	2	Luxación LIO	1
2	26/10/2019	H02	4	Alteración palpebral	1
2	26/10/2019	H20	6	Uveitis	1
1	26/10/2019	H16.0	1	Úlcera corneal	1
2	26/10/2019	H16.0	1	Úlcera corneal	1
1	26/10/2019	H46	10	Neuritis óptica	1
2	26/10/2019	H10.3	0	Conjuntivitis	1
1	26/10/2019	H10.3	0	Conjuntivitis	1
2	26/10/2019	S05.9	9	Traumatismo	1
2	26/10/2019	H10.3	0	Conjuntivitis	1
2	26/10/2019	H49.9	10	Parálisis oculomotora	1
2	26/10/2019	Z98.4	2	Post op catarata	1

2	26/10/2019	H53	11	No patología	1
1	26/10/2019	H16.9	1	Queratitis	1
1	26/10/2019	H16.9	1	Queratitis	1
2	26/10/2019	H02.05	4	Distiquiasis	1
2	26/10/2019	H0.41	1	Ojo seco	1
2	26/10/2019	H20	6	Uveitis	1
1	26/10/2019	H10.3	0	Conjuntivitis	1
2	26/10/2019	Alta por fuga	11	Alta por fuga	1
1	26/10/2019	H16.0	1	Úlcera corneal	1
1	26/10/2019	H43.1	7	Hemovítreo	1
1	26/10/2019	H16.9	1	Queratitis	1
1	26/10/2019	H00.02	4	Orzuelo	1
2	26/10/2019	H16.9	1	Queratitis	1
2	26/10/2019	H16.0	1	Úlcera corneal	1
1	26/10/2019	H00.02	4	Orzuelo	1
1	26/10/2019	H20	6	Uveitis	1
1	26/10/2019	H00.02	4	Orzuelo	1
1	26/10/2019	H10.3	0	Conjuntivitis	1
1	26/10/2019	S05.6	9	Perforación ocular	1
2	26/10/2019	H16.9	1	Queratitis	1
2	26/10/2019	H11.82	0	Conjuntivocalasia	1
1	26/10/2019	H02	4	Lesión palpebral	1
2	26/10/2019	H16.0	1	Úlcera corneal	1
1	26/10/2019	H10.3	0	Conjuntivitis	1
2	26/10/2019	H43.81	7	DVP	1
1	26/10/2019	H10.3	0	Conjuntivitis	1
1	26/10/2019	T15.0	1	Cuerpo extraño	1
1	26/10/2019	T15.0	1	Cuerpo extraño	1
1	26/10/2019	H10.3	0	Conjuntivitis	1
2	27/10/2019	H02	4	Lesión palpebral	1
1	27/10/2019	T15.0	1	Cuerpo extraño	1
1	27/10/2019	H02	4	Lesión palpebral	1
2	27/10/2019	H53	11	No patología	1
1	27/10/2019	H53	11	No patología	1
1	27/10/2019	T15.0	1	Cuerpo extraño	1
2	27/10/2019	H59.3	4	Post op párpados	1
2	27/10/2019	Z98.4	2	Post op catarata	1
1	27/10/2019	H10.3	0	Conjuntivitis	1
1	27/10/2019	H34.9	8	Oclusión arterial	1
2	27/10/2019	H0.41	1	Ojo seco	1
2	27/10/2019	H16.9	1	Queratitis	1
2	27/10/2019	H10.3	0	Conjuntivitis	1
2	27/10/2019	H35.3	8	Maculopatía	1
1	27/10/2019	H10.3	0	Conjuntivitis	1
2	27/10/2019	H16.9	1	Queratitis	1
2	27/10/2019	H10.3	0	Conjuntivitis	1
2	27/10/2019	H00.02	4	Orzuelo	1
1	27/10/2019	H16.0	1	Úlcera corneal	1
1	27/10/2019	T15.0	1	Cuerpo extraño	1
2	27/10/2019	H02.05	4	Distiquiasis	1
1	27/10/2019	S05.30	9	Laceración conjuntival	1
1	27/10/2019	H43.81	7	DVP	1
2	27/10/2019	H10.3	0	Conjuntivitis	1
1	27/10/2019	H10.3	0	Conjuntivitis	1
1	27/10/2019	H10.3	0	Conjuntivitis	1
1	27/10/2019	T15.0	1	Cuerpo extraño	1
1	27/10/2019	H43.81	7	DVP	1
1	27/10/2019	H16.0	1	Úlcera corneal	1
2	27/10/2019	H16.9	1	Queratitis	1
2	27/10/2019	H10.3	0	Conjuntivitis	1
1	27/10/2019	T15.0	1	Cuerpo extraño	1
2	27/10/2019	H33.30	8	Desgarro retiniano	1
2	27/10/2019	H16.0	1	Úlcera corneal	1
1	27/10/2019	H10.3	0	Conjuntivitis	1
1	28/10/2019	T15.0	1	Cuerpo extraño	1
2	28/10/2019	H00.02	4	Orzuelo	1

1	28/10/2019	H20	6	Uveitis		1
1	28/10/2019	H10.3	0	Conjuntivitis		1
2	28/10/2019	H20	6	Uveitis		1
1	28/10/2019	H10.3	0	Conjuntivitis		1
1	28/10/2019	H27.8	2	OCP		1
2	28/10/2019	H10.3	0	Conjuntivitis		1
2	28/10/2019	H16.0	1	Úlcera corneal		1
1	28/10/2019	H16.0	1	Úlcera corneal		1
2	28/10/2019	H44.009	8	Endoftalmitis		1
2	28/10/2019	H40.05	3	HTO		1
1	28/10/2019	H53	11	No patología		1
1	28/10/2019	H10.3	0	Conjuntivitis		1
2	28/10/2019	H16.9	1	Queratitis		1
2	28/10/2019	H11.3	0	Hiposfagma		1
2	28/10/2019	H16.9	1	Queratitis		1
1	28/10/2019	H16.9	1	Queratitis		1
2	28/10/2019	H35.3	8	Maculopatía		1
2	28/10/2019	H16.9	1	Queratitis		1
2	28/10/2019	H10.81	0	Pingueculitis		1
1	28/10/2019	H35.30	8	DMAE		1
2	28/10/2019	H16.9	1	Queratitis		1
2	28/10/2019	H43.81	7	DVP		1
2	28/10/2019	H10.3	0	Conjuntivitis		1
1	28/10/2019	H40.9	3	Glaucoma		1
2	28/10/2019	H16.9	1	Queratitis		1
2	28/10/2019	H43.81	7	DVP		1
2	28/10/2019	H01.00	4	Blefaritis		1
2	28/10/2019	H16.0	1	Úlcera corneal		1
2	28/10/2019	H01.00	4	Blefaritis		1
2	28/10/2019	H01.00	4	Blefaritis		1
2	28/10/2019	H16.9	1	Queratitis		1
2	28/10/2019	H43.81	7	DVP		1
2	28/10/2019	H00.02	4	Orzuelo		1
2	28/10/2019	H17.8	1	Infiltrados corneales		1
2	28/10/2019	H10.3	0	Conjuntivitis		1
1	28/10/2019	T15.0	1	Cuerpo extraño		1
2	28/10/2019	H16.9	1	Queratitis		1
2	28/10/2019	H16.9	1	Queratitis		1
2	28/10/2019	H17.8	1	Infiltrados corneales		1
1	28/10/2019	H16.9	1	Queratitis		1
2	28/10/2019	H16.9	1	Queratitis		1
1	28/10/2019	H16.9	1	Queratitis		1
1	28/10/2019	S05.9	9	Traumatismo		1
1	28/10/2019	H11.3	0	Hiposfagma		1
2	28/10/2019	H16.9	1	Queratitis		1
1	28/10/2019	H16.9	1	Queratitis		1
1	29/10/2019	H16.0	1	Úlcera corneal		1
1	29/10/2019	H10.3	0	Conjuntivitis		1
1	29/10/2019	H16.9	1	Queratitis		1
1	29/10/2019	H10.3	0	Conjuntivitis		1
2	29/10/2019	H16.9	1	Queratitis		1
2	29/10/2019	H53	11	No patología		1
2	29/10/2019	H11.3	0	Hiposfagma		1
2	29/10/2019	H16.9	1	Queratitis		1
1	29/10/2019	H16.9	1	Queratitis		1
1	29/10/2019	H43.1	7	Hemovítreo		1
2	29/10/2019	H16.9	1	Queratitis		1
1	29/10/2019	H15.00	6	Escleritis		1
2	29/10/2019	H10.3	0	Conjuntivitis		1
2	29/10/2019	H17.8	1	Infiltrados corneales		1
1	29/10/2019	H02.05	4	Distiquiasis		1
1	29/10/2019	S05.9	9	Traumatismo		1
2	29/10/2019	H59.88	8	Post op retina		1
2	29/10/2019	H16.9	1	Queratitis		1
1	29/10/2019	H17.8	1	Infiltrados corneales		1
1	29/10/2019	H10.3	0	Conjuntivitis		1

1	29/10/2019	H16.3	1	Abceso corneal	1
1	29/10/2019	H17.8	1	Infiltrados corneales	1
2	29/10/2019	H53	11	No patología	1
2	29/10/2019	H10.3	0	Conjuntivitis	1
2	29/10/2019	H33.30	8	Desgarro retiniano	1
1	29/10/2019	H11.3	0	Hiposfagma	1
2	29/10/2019	G43.109	10	Aura	1
2	29/10/2019	H43.81	7	DVP	1
2	29/10/2019	H53	11	No patología	1
1	29/10/2019	T15.0	1	Cuerpo extraño	1
2	29/10/2019	H27.8	2	OCP	1
2	29/10/2019	H43.81	7	DVP	1
2	29/10/2019	H53.9	11	Alteraciones visuales	1
1	29/10/2019	T15.0	1	Cuerpo extraño	1
2	29/10/2019	H01.00	4	Blefaritis	1
2	29/10/2019	H35.3	8	Maculopatía	1
1	29/10/2019	H10.3	0	Conjuntivitis	1
1	29/10/2019	H11.3	0	Hiposfagma	1
1	29/10/2019	H16.9	1	Queratitis	1
1	29/10/2019	H10.3	0	Conjuntivitis	1
1	29/10/2019	H16.0	1	Úlcera corneal	1
1	29/10/2019	H16.9	1	Queratitis	1
1	29/10/2019	H10.3	0	Conjuntivitis	1
1	29/10/2019	T15.0	1	Cuerpo extraño	1
2	29/10/2019	H02	4	Alteración palpebral	1
1	29/10/2019	T15.0	1	Cuerpo extraño	1
1	29/10/2019	H16.0	1	Úlcera corneal	1
1	29/10/2019	H0.41	1	Ojo seco	1
1	29/10/2019	T15.0	1	Cuerpo extraño	1
1	30/10/2019	H16.0	1	Úlcera corneal	1
2	30/10/2019	H00.02	4	Orzuelo	1
2	30/10/2019	H43.81	7	DVP	1
2	30/10/2019	H10.3	0	Conjuntivitis	1
2	30/10/2019	H16.9	1	Queratitis	1
2	30/10/2019	H01.00	4	Blefaritis	1
2	30/10/2019	H10.3	0	Conjuntivitis	1
1	30/10/2019	H27.8	2	OCP	1
1	30/10/2019	T15.0	1	Cuerpo extraño	1
2	30/10/2019	H16.9	1	Queratitis	1
2	30/10/2019	H16.9	1	Queratitis	1
2	30/10/2019	H01.00	4	Blefaritis	1
1	30/10/2019	H16.3	1	Abceso corneal	1
2	30/10/2019	H10.3	0	Conjuntivitis	1
1	30/10/2019	H04.32	5	Dacriocistitis aguda	1
2	30/10/2019	H10.3	0	Conjuntivitis	1
1	30/10/2019	H11.00	0	Pterigium	1
2	30/10/2019	H43.81	7	DVP	1
1	30/10/2019	T15.0	1	Cuerpo extraño	1
1	30/10/2019	H01.00	4	Blefaritis	1
2	30/10/2019	H10.3	0	Conjuntivitis	1
1	30/10/2019	H00.02	4	Orzuelo	1
2	30/10/2019	H33.0	8	Desprendimiento de retina	1
2	30/10/2019	H43.81	7	DVP	1
1	30/10/2019	H02.05	4	Distiquiasis	1
1	30/10/2019	H00.02	4	Orzuelo	1
2	30/10/2019	H43.81	7	DVP	1
2	30/10/2019	H16.9	1	Queratitis	1
2	30/10/2019	H43.81	7	DVP	1
2	30/10/2019	H11.3	0	Hiposfagma	1
2	30/10/2019	H00.02	4	Orzuelo	1
2	30/10/2019	H53	11	No patología	1
1	30/10/2019	H16.0	1	Úlcera corneal	1
1	30/10/2019	H10.3	0	Conjuntivitis	1
1	30/10/2019	G43.109	10	Aura	1
2	30/10/2019	H43.81	7	DVP	1
2	30/10/2019	H10.3	0	Conjuntivitis	1

1	30/10/2019	T15.0	1	Cuerpo extraño	1
1	30/10/2019	H53	11	No patología	1
2	30/10/2019	H11.3	0	Hiposfagma	1
1	30/10/2019	H16.9	1	Queratitis	1
2	30/10/2019	H10.3	0	Conjuntivitis	1
1	30/10/2019	H10.3	0	Conjuntivitis	1
2	30/10/2019	H10.3	0	Conjuntivitis	1
2	30/10/2019	T15.0	1	Cuerpo extraño	1
2	31/10/2019	T15.0	1	Cuerpo extraño	1
1	31/10/2019	H02	4	Alteración palpebral	1
2	31/10/2019	H16.9	1	Queratitis	1
2	31/10/2019	H16.9	1	Queratitis	1
1	31/10/2019	H15.00	6	Escleritis	1
1	31/10/2019	G43.109	10	Aura	1
2	31/10/2019	H16.9	1	Queratitis	1
2	31/10/2019	H10.3	0	Conjuntivitis	1
2	31/10/2019	H10.3	0	Conjuntivitis	1
1	31/10/2019	H16.9	1	Queratitis	1
2	31/10/2019	H0.41	1	Ojo seco	1
2	31/10/2019	H11.00	0	Pterigium	1
1	31/10/2019	H53	11	No patología	1
2	31/10/2019	S05.9	9	Traumatismo	1
2	31/10/2019	H11.3	0	Hiposfagma	1
1	31/10/2019	H10.3	0	Conjuntivitis	1
2	31/10/2019	H10.81	0	Pingueculitis	1
1	31/10/2019	H53	11	No patología	1
1	31/10/2019	T15.0	1	Cuerpo extraño	1
1	31/10/2019	H00.19	4	Chalazión	1
2	31/10/2019	H01.00	4	Blefaritis	1
2	31/10/2019	H16.0	1	Úlcera corneal	1
1	31/10/2019	H10.3	0	Conjuntivitis	1
2	31/10/2019	H18.20	1	Edema corneal	1
2	31/10/2019	H00.19	4	Chalazión	1
2	31/10/2019	H43.81	7	DVP	1
2	31/10/2019	H20	6	Uveitis	1
2	31/10/2019	S05.9	9	Traumatismo	1
2	31/10/2019	H10.3	0	Conjuntivitis	1
2	31/10/2019	H10.3	0	Conjuntivitis	1
1	31/10/2019	T15.0	1	Cuerpo extraño	1
1	31/10/2019	H16.9	1	Queratitis	1
2	31/10/2019	H33.30	8	Desgarro retiniano	1
2	31/10/2019	H11.44	0	Quiste conjuntival	1
1	31/10/2019	H10.3	0	Conjuntivitis	1
1	31/10/2019	H11.3	0	Hiposfagma	1
1	31/10/2019	T15.0	1	Cuerpo extraño	1
1	31/10/2019	H17.8	1	Infiltrados corneales	1
2	31/10/2019	H16.9	1	Queratitis	1
2	31/10/2019	H00.02	4	Orzuelo	1
1	01/11/2019	H16.9	1	Queratitis	1
1	01/11/2019	S05.9	9	Traumatismo	1
1	01/11/2019	H10.3	0	Conjuntivitis	1
2	01/11/2019	H20	6	Uveitis	1
2	01/11/2019	H43.81	7	DVP	1
2	01/11/2019	H18.20	1	Edema corneal	1
2	01/11/2019	H11.3	0	Hiposfagma	1
2	01/11/2019	H59.3	5	Postop via lagrimal	1
2	01/11/2019	H16.9	1	Queratitis	1
1	01/11/2019	H16.0	1	Úlcera corneal	1
1	01/11/2019	H11.3	0	Hiposfagma	1
1	01/11/2019	H16.0	1	Úlcera corneal	1
1	01/11/2019	H16.0	1	Úlcera corneal	1
2	01/11/2019	H0.41	1	Ojo seco	1
1	01/11/2019	H16.0	1	Úlcera corneal	1
1	01/11/2019	H53	11	No patología	1
1	01/11/2019	H16.0	1	Úlcera corneal	1
2	01/11/2019	H01.00	4	Blefaritis	1

2	01/11/2019	H16.0	1	Úlcera corneal	1
2	01/11/2019	H10.3	0	Conjuntivitis	1
1	01/11/2019	T15.0	1	Cuerpo extraño	1
2	01/11/2019	H00.02	4	Orzuelo	1
2	01/11/2019	H01.00	4	Blefaritis	1
2	01/11/2019	Z98.4	2	Post op catarata	1
2	01/11/2019	H53	11	No patología	1
2	01/11/2019	H16.9	1	Queratitis	1
1	01/11/2019	H53	11	No patología	1
2	01/11/2019	H16.0	1	Úlcera corneal	1
1	01/11/2019	H16.9	1	Queratitis	1
1	01/11/2019	H02	4	Alteración palpebral	1
1	01/11/2019	H10.3	0	Conjuntivitis	1
1	01/11/2019	H10.3	0	Conjuntivitis	1
1	01/11/2019	H16.0	1	Úlcera corneal	1
2	01/11/2019	H10.3	0	Conjuntivitis	1
1	01/11/2019	H16.9	1	Queratitis	1
1	01/11/2019	H0.41	1	Ojo seco	1
1	01/11/2019	H53	11	No patología	1
1	01/11/2019	H16.0	1	Úlcera corneal	1
2	01/11/2019	H33.30	8	Desgarro retiniano	1
1	01/11/2019	H02	4	Lesión palpebral	1
2	01/11/2019	H16.9	1	Queratitis	1
2	01/11/2019	T15.0	1	Cuerpo extraño	1
2	01/11/2019	H27.8	2	OCP	1
2	01/11/2019	H0.41	1	Ojo seco	1
2	01/11/2019	H16.9	1	Queratitis	1
2	01/11/2019	H10.3	0	Conjuntivitis	1
2	01/11/2019	H16.0	1	Úlcera corneal	1
1	01/11/2019	H10.3	0	Conjuntivitis	1
2	01/11/2019	H16.9	1	Queratitis	1
1	01/11/2019	H00.03	4	Celulitis preseptal	1
2	01/11/2019	H10.3	0	Conjuntivitis	1
1	01/11/2019	H04.32	5	Dacriocistitis aguda	1
2	01/11/2019	G43.9	10	Migraña	1
1	02/11/2019	H10.3	0	Conjuntivitis	1
2	02/11/2019	H33.0	8	Desprendimiento de retina	1
1	02/11/2019	H10.3	0	Conjuntivitis	1
2	02/11/2019	H16.9	1	Queratitis	1
2	02/11/2019	H16.9	1	Queratitis	1
1	02/11/2019	H15.1	6	Epiescleritis	1
2	02/11/2019	H20	6	Uveitis	1
2	02/11/2019	H10.3	0	Conjuntivitis	1
1	02/11/2019	H16.9	1	Queratitis	1
1	02/11/2019	H10.3	0	Conjuntivitis	1
2	02/11/2019	H00.02	4	Orzuelo	1
2	02/11/2019	H10.3	0	Conjuntivitis	1
1	02/11/2019	H02.05	4	Distiquiasis	1
2	02/11/2019	H16.0	1	Úlcera corneal	1
2	02/11/2019	H43.81	7	DVP	1
1	02/11/2019	H01.00	4	Blefaritis	1
2	02/11/2019	H16.9	1	Queratitis	1
1	02/11/2019	H10.3	0	Conjuntivitis	1
1	02/11/2019	H0.41	1	Ojo seco	1
2	02/11/2019	H16.3	1	Abceso corneal	1
1	02/11/2019	H10.3	0	Conjuntivitis	1
1	02/11/2019	H18.20	1	Edema corneal	1
2	02/11/2019	T15.0	1	Cuerpo extraño	1
2	02/11/2019	G43.109	10	Aura	1
2	02/11/2019	H20	6	Uveitis	1
1	02/11/2019	S05.9	9	Traumatismo	1
2	02/11/2019	H40.9	3	Glaucoma	1
2	02/11/2019	H16.0	1	Úlcera corneal	1
2	02/11/2019	S05.30	9	Laceración conjuntival	1
1	02/11/2019	H00.02	4	Orzuelo	1
2	02/11/2019	H00.02	4	Orzuelo	1

2	02/11/2019	H00.02		4	Orzuelo	1
1	02/11/2019	H01.00		4	Blefaritis	1
1	02/11/2019	G43.9		10	Migraña	1
2	02/11/2019	H10.3		0	Conjuntivitis	1
2	02/11/2019	H10.3		0	Conjuntivitis	1
2	02/11/2019	H33.0		8	Desprendimiento de retina	1
1	02/11/2019	H20		6	Uveitis	1
1	02/11/2019	H53		11	No patología	1
1	02/11/2019	H11.3		0	Hiposfagma	1
1	02/11/2019	T15.0		1	Cuerpo extraño	1
1	02/11/2019	G43.9		10	Migraña	1
1	02/11/2019	H10.81		0	Pingueculitis	1
1	02/11/2019	H16.9		1	Queratitis	1
1	02/11/2019	H16.9		1	Queratitis	1
2	02/11/2019	H53		11	No patología	1
1	02/11/2019	S05.9		9	Traumatismo	1
1	02/11/2019	S05.9		9	Traumatismo	1
1	02/11/2019	H10.3		0	Conjuntivitis	1
1	02/11/2019	H11.3		0	Hiposfagma	1
2	02/11/2019	H16.9		1	Queratitis	1
1	02/11/2019	H11.3		0	Hiposfagma	1
2	02/11/2019	H53		11	No patología	1
2	02/11/2019	H53		11	No patología	1
2	02/11/2019	H0.41		1	Ojo seco	1
2	02/11/2019	H0.41		1	Ojo seco	1
1	02/11/2019	H10.3		0	Conjuntivitis	1
2	02/11/2019	H10.3		0	Conjuntivitis	1
2	02/11/2019	H00.02		4	Orzuelo	1
1	02/11/2019	S05.9		9	Traumatismo	1
2	02/11/2019	H04.32		5	Dacriocistitis aguda	1
1	02/11/2019	H53		11	No patología	1
2	02/11/2019	H16.9		1	Queratitis	1
1	02/11/2019	H10.3		0	Conjuntivitis	1
2	03/11/2019	H11.44		0	Quiste conjuntival	1
1	03/11/2019	H10.3		0	Conjuntivitis	1
1	03/11/2019	H33.0		8	Desprendimiento de retina	1
2	03/11/2019	H10.3		0	Conjuntivitis	1
2	03/11/2019	H10.3		0	Conjuntivitis	1
2	03/11/2019	H16.9		1	Queratitis	1
2	03/11/2019	H10.3		1	Conjuntivitis	1
1	03/11/2019	H01.00		4	Blefaritis	1
1	03/11/2019	H16.0		1	Úlcera corneal	1
1	03/11/2019	H01.00		4	Blefaritis	1
1	03/11/2019	H11.3		0	Hiposfagma	1
2	03/11/2019	H10.3		0	Conjuntivitis	1
2	03/11/2019	H10.3		0	Conjuntivitis	1
2	03/11/2019	H17.8		1	Infiltrados corneales	1
1	03/11/2019	H10.3		1	Conjuntivitis	1
1	03/11/2019	H01.00		4	Blefaritis	1
2	03/11/2019	H01.00		4	Blefaritis	1
2	03/11/2019	H0.41		1	Ojo seco	1
1	03/11/2019	H16.9		1	Queratitis	1
1	03/11/2019	H43.81		7	DVP	1
2	03/11/2019	H01.00		4	Blefaritis	1
1	03/11/2019	H10.3		0	Conjuntivitis	1
1	03/11/2019	T15.0		1	Cuerpo extraño	1
2	03/11/2019	H02		4	Alteración palpebral	1
2	03/11/2019	H16.0		1	Úlcera corneal	1
1	03/11/2019	H16.0		1	Úlcera corneal	1
2	03/11/2019	H16.9		1	Queratitis	1
2	03/11/2019	H11.3		0	Hiposfagma	1
1	03/11/2019	H02.05		4	Distiquiasis	1
2	03/11/2019	S05.9		9	Traumatismo	1
1	03/11/2019	H16.9		1	Queratitis	1
1	03/11/2019	H33.0		8	Desprendimiento de retina	1
2	03/11/2019	H53		11	No patología	1

1	03/11/2019	T15.0	1	Cuerpo extraño	1
2	03/11/2019	H16.9	1	Queratitis	1
2	03/11/2019	H01.00	4	Blefaritis	1
2	03/11/2019	H16.0	1	Úlcera corneal	1
2	03/11/2019	H53	11	No patología	1
2	03/11/2019	H53	11	No patología	1
1	03/11/2019	T15.0	1	Cuerpo extraño	1
2	03/11/2019	H33.0	8	Desprendimiento de retina	1
1	03/11/2019	H16.0	1	Úlcera corneal	1
2	03/11/2019	H11.3	0	Hiposfagma	1
1	03/11/2019	T15.0	1	Cuerpo extraño	1
2	03/11/2019	H01.00	4	Blefaritis	1
1	03/11/2019	H10.3	0	Conjuntivitis	1
1	03/11/2019	H10.3	0	Conjuntivitis	1
1	03/11/2019	T15.0	1	Cuerpo extraño	1
2	03/11/2019	S05.30	9	Laceración conjuntival	1
2	03/11/2019	H10.3	0	Conjuntivitis	1
1	03/11/2019	H00.02	4	Orzuelo	1
1	03/11/2019	T15.0	1	Cuerpo extraño	1
2	03/11/2019	H15.1	6	Epiescleritis	1
2	03/11/2019	H34.82	8	Trombosis venosa	1
2	03/11/2019	H16.0	1	Úlcera corneal	1
2	03/11/2019	H43.81	7	DVP	1
2	04/11/2019	H10.3	1	Conjuntivitis	1
1	04/11/2019	H16.9	1	Queratitis	1
2	04/11/2019	H10.3	0	Conjuntivitis	1
1	04/11/2019	H35.37	8	MER	1
2	04/11/2019	H11.3	0	Hiposfagma	1
2	04/11/2019	H33.30	8	Desgarro retiniano	1
2	04/11/2019	H02.05	4	Distiquiasis	1
2	04/11/2019	H53	11	No patología	1
2	04/11/2019	H16.9	1	Queratitis	1
2	04/11/2019	H16.9	1	Queratitis	1
2	04/11/2019	H10.3	0	Conjuntivitis	1
1	04/11/2019	H10.3	0	Conjuntivitis	1
2	04/11/2019	H53	11	No patología	1
1	04/11/2019	H43.1	7	Hemovítreo	1
2	04/11/2019	H16.9	1	Queratitis	1
2	04/11/2019	H01.00	4	Blefaritis	1
1	04/11/2019	H10.3	0	Conjuntivitis	1
1	04/11/2019	H10.3	0	Conjuntivitis	1
1	04/11/2019	T15.0	1	Cuerpo extraño	1
2	04/11/2019	H18.20	1	Edema corneal	1
1	04/11/2019	H33.0	8	Desprendimiento de retina	1
1	04/11/2019	H16.9	1	Queratitis	1
1	04/11/2019	H43.81	7	DVP	1
2	04/11/2019	G43.9	10	Migraña	1
2	04/11/2019	G43.9	10	Migraña	1
2	04/11/2019	H11.3	0	Hiposfagma	1
1	04/11/2019	S05.9	9	Traumatismo	1
1	04/11/2019	S05.30	9	Laceración conjuntival	1
2	04/11/2019	H16.0	1	Úlcera corneal	1
1	04/11/2019	T15.0	1	Cuerpo extraño	1
1	04/11/2019	H43.81	7	DVP	1
2	04/11/2019	T15.0	1	Cuerpo extraño	1
1	04/11/2019	H00.02	4	Orzuelo	1
1	05/11/2019	H10.3	0	Conjuntivitis	1
2	05/11/2019	H16.9	1	Queratitis	1
2	05/11/2019	H10.81	0	Pingueculitis	1
2	05/11/2019	H01.00	4	Blefaritis	1
1	05/11/2019	H10.3	0	Conjuntivitis	1
2	05/11/2019	H11.3	0	Hiposfagma	1
2	05/11/2019	H16.0	1	Úlcera corneal	1
1	05/11/2019	H10.3	0	Conjuntivitis	1
2	05/11/2019	H16.9	1	Queratitis	1
2	05/11/2019	H10.3	0	Conjuntivitis	1

1	05/11/2019	H20		6	Uveitis	1
1	05/11/2019	H17.8		1	Infiltrados corneales	1
2	05/11/2019	H11.3		0	Hiposfagma	1
1	05/11/2019	S05.9		9	Traumatismo	1
2	05/11/2019	H01.00		4	Blefaritis	1
2	05/11/2019	G43.9		10	Migraña	1
2	05/11/2019	H11.3		0	Hiposfagma	1
2	05/11/2019	H33.0		8	Desprendimiento de retina	1
1	05/11/2019	H00.02		4	Orzuelo	1
2	05/11/2019	H10.3		0	Conjuntivitis	1
1	05/11/2019	H20		6	Uveitis	1
2	05/11/2019	H05.01		4	Celulitis orbitaria	1
1	05/11/2019	H53.10		11	Problema refractivo	1
1	05/11/2019	H33.0		8	Desprendimiento de retina	1
2	05/11/2019	S05.9		9	Traumatismo	1
1	05/11/2019	H16.9		1	Queratitis	1
2	05/11/2019	H43.81		7	DVP	1
1	05/11/2019	H16.0		1	Úlcera corneal	1
1	05/11/2019	H01.00		4	Blefaritis	1
2	05/11/2019	H43.81		7	DVP	1
1	05/11/2019	H35.3		8	Maculopatía	1
2	05/11/2019	H01.00		4	Blefaritis	1
2	05/11/2019	H20		6	Uveitis	1
2	05/11/2019	H16.0		1	Úlcera corneal	1
2	05/11/2019	H16.9		1	Queratitis	1
2	05/11/2019	H43.81		7	DVP	1
2	05/11/2019	H53		11	No patología	1
1	05/11/2019	H35.3		8	Maculopatía	1
2	05/11/2019	H10.3		0	Conjuntivitis	1
1	05/11/2019	H16.0		1	Úlcera corneal	1
2	05/11/2019	H10.3		0	Conjuntivitis	1
1	05/11/2019	T15.0		1	Cuerpo extraño	1
1	05/11/2019	S05.9		9	Traumatismo	1
2	05/11/2019	H16.9		1	Queratitis	1
2	05/11/2019	H10.3		0	Conjuntivitis	1
2	05/11/2019	H00.02		4	Orzuelo	1
2	05/11/2019	H10.5		4	Blefaroconjuntivitis	1
1	06/11/2019	H16.9		1	Queratitis	1
2	06/11/2019	H53		11	No patología	1
1	06/11/2019	H40.5		6	Glaucoma secundario	1
2	06/11/2019	H17.8		1	Infiltrados corneales	1
2	06/11/2019	H34.82		8	Trombosis venosa	1
2	06/11/2019	H53		11	No patología	1
2	06/11/2019	H10.3		0	Conjuntivitis	1
2	06/11/2019	H16.9		1	Queratitis	1
1	06/11/2019	H16.9		1	Queratitis	1
1	06/11/2019	H20		6	Uveitis	1
1	06/11/2019	S05.9		9	Traumatismo	1
1	06/11/2019	H16.0		1	Úlcera corneal	1
1	06/11/2019	H10.3		0	Conjuntivitis	1
2	06/11/2019	S05.9		9	Traumatismo	1
1	06/11/2019	H17.8		1	Infiltrados corneales	1
2	06/11/2019	H16.0		1	Úlcera corneal	1
2	06/11/2019	H16.0		1	Úlcera corneal	1
2	06/11/2019	G43.9		10	Migraña	1
2	06/11/2019	H10.3		0	Conjuntivitis	1
1	06/11/2019	H16.0		1	Úlcera corneal	1
1	06/11/2019	H16.9		1	Queratitis	1
2	06/11/2019	H02.05		4	Distiquiasis	1
2	06/11/2019	T15.0		1	Cuerpo extraño	1
2	06/11/2019	H34.82		8	Trombosis venosa	1
2	06/11/2019	H00.02		4	Orzuelo	1
2	06/11/2019	T15.0		1	Cuerpo extraño	1
2	06/11/2019	H16.9		1	Queratitis	1
2	06/11/2019	H16.9		1	Queratitis	1
1	06/11/2019	G43.109		10	Aura	1

1	06/11/2019	H0.41	1	Ojo seco	1
2	06/11/2019	H10.3	0	Conjuntivitis	1
1	06/11/2019	S05.30	9	Laceración conjuntival	1
1	06/11/2019	H16.9	1	Queratitis	1
2	06/11/2019	H53	11	No patología	1
1	06/11/2019	T15.0	1	Cuerpo extraño	1
1	06/11/2019	H16.0	1	Úlcera corneal	1
1	06/11/2019	H11.3	0	Hiposfagma	1
2	06/11/2019	H53	11	No patología	1
1	06/11/2019	Alta por fuga	11	Alta por fuga	1
2	07/11/2019	H53.9	11	Alteraciones visuales	1
2	07/11/2019	H16.9	1	Queratitis	1
1	07/11/2019	Z98.4	2	Post op catarata	1
1	07/11/2019	H02.05	4	Distiquiasis	1
1	07/11/2019	H16.0	1	Úlcera corneal	1
2	07/11/2019	H16.9	1	Queratitis	1
1	07/11/2019	Alta por fuga	11	Alta por fuga	1
1	07/11/2019	H10.3	0	Conjuntivitis	1
2	07/11/2019	H10.3	0	Conjuntivitis	1
2	07/11/2019	H16.9	1	Queratitis	1
2	07/11/2019	H43.81	7	DVP	1
2	07/11/2019	H0.41	1	Ojo seco	1
1	07/11/2019	H43.81	7	DVP	1
2	07/11/2019	H0.41	1	Ojo seco	1
1	07/11/2019	H33.0	8	Desprendimiento de retina	1
2	07/11/2019	H05.01	4	Celulitis orbitaria	1
1	07/11/2019	H16.9	1	Queratitis	1
2	07/11/2019	H01.00	4	Blefaritis	1
2	07/11/2019	H53	11	No patología	1
2	07/11/2019	H10.3	0	Conjuntivitis	1
1	07/11/2019	H27.8	2	OCP	1
2	07/11/2019	H10.3	0	Conjuntivitis	1
2	07/11/2019	H10.3	0	Conjuntivitis	1
2	07/11/2019	H11.3	0	Hiposfagma	1
2	07/11/2019	H43.81	7	DVP	1
2	07/11/2019	H43.81	7	DVP	1
1	07/11/2019	H16.0	1	Úlcera corneal	1
2	07/11/2019	H01.00	4	Blefaritis	1
2	07/11/2019	H00.02	4	Orzuelo	1
2	07/11/2019	H53	11	No patología	1
1	07/11/2019	H43.81	7	DVP	1
2	07/11/2019	T15.0	1	Cuerpo extraño	1
2	07/11/2019	H00.02	4	Orzuelo	1
2	07/11/2019	H53	11	No patología	1
1	07/11/2019	H43.81	7	DVP	1
2	07/11/2019	H11.3	0	Hiposfagma	1
2	07/11/2019	H53	11	No patología	1
2	07/11/2019	T15.0	1	Cuerpo extraño	1
1	07/11/2019	T15.0	1	Cuerpo extraño	1
1	07/11/2019	T15.0	1	Cuerpo extraño	1
1	07/11/2019	H16.9	1	Queratitis	1
1	08/11/2019	H10.3	0	Conjuntivitis	1
2	08/11/2019	H10.3	0	Conjuntivitis	1
2	08/11/2019	Alta por fuga	11	Alta por fuga	1
2	08/11/2019	H0.41	1	Ojo seco	1
2	08/11/2019	H16.9	1	Queratitis	1
1	08/11/2019	H20	6	Uveitis	1
1	08/11/2019	H17.8	1	Infiltrados corneales	1
1	08/11/2019	H16.0	1	Úlcera corneal	1
2	08/11/2019	H17.8	1	Infiltrados corneales	1
2	08/11/2019	H17.8	1	Infiltrados corneales	1
2	08/11/2019	H16.0	1	Úlcera corneal	1
2	08/11/2019	H16.9	1	Queratitis	1
1	08/11/2019	H00.02	4	Orzuelo	1
1	08/11/2019	H17.8	1	Infiltrados corneales	1
2	08/11/2019	H16.9	1	Queratitis	1

1	08/11/2019	H35.30	8	DMAE	1
2	08/11/2019	H16.9	1	Queratitis	1
1	08/11/2019	H11.3	0	Hiposfagma	1
2	08/11/2019	H16.9	1	Queratitis	1
1	08/11/2019	H16.9	1	Queratitis	1
2	08/11/2019	H10.5	4	Blefaroconjuntivitis	1
2	08/11/2019	H16.9	1	Queratitis	1
2	08/11/2019	H53	11	No patología	1
2	08/11/2019	H00.02	4	Orzuelo	1
2	08/11/2019	H11.3	0	Hiposfagma	1
1	08/11/2019	H20	6	Uveitis	1
2	08/11/2019	H02	4	Alteración palpebral	1
2	08/11/2019	G43.109	10	Aura	1
2	08/11/2019	H16.9	1	Queratitis	1
2	08/11/2019	H16.9	1	Queratitis	1
2	08/11/2019	H0.41	1	Ojo seco	1
2	08/11/2019	H43.1	7	Hemovítreo	1
2	08/11/2019	Alta por fuga	11	Alta por fuga	1
2	08/11/2019	H35.30	8	DMAE	1
2	08/11/2019	H40.9	3	Glaucoma	1
2	08/11/2019	H35.3	8	Maculopatía	1
1	08/11/2019	H16.9	1	Queratitis	1
1	08/11/2019	H00.02	4	Orzuelo	1
2	08/11/2019	H16.9	1	Queratitis	1
2	08/11/2019	G43.9	10	Migraña	1
1	08/11/2019	H10.3	0	Conjuntivitis	1
2	08/11/2019	G43.9	10	Migraña	1
1	08/11/2019	H10.81	0	Pingueculitis	1
2	08/11/2019	H10.3	0	Conjuntivitis	1
2	08/11/2019	H11.3	0	Hiposfagma	1
1	08/11/2019	H31.42	8	Coroidopatía serosa central	1
2	09/11/2019	T15.0	1	Cuerpo extraño	1
2	09/11/2019	H35.37	8	MER	1
2	09/11/2019	H02.05	4	Distiquiasis	1
1	09/11/2019	H10.3	0	Conjuntivitis	1
2	09/11/2019	H10.3	0	Conjuntivitis	1
1	09/11/2019	H10.3	0	Conjuntivitis	1
1	09/11/2019	H16.9	1	Queratitis	1
1	09/11/2019	H0.41	1	Ojo seco	1
1	09/11/2019	H10.3	0	Conjuntivitis	1
2	09/11/2019	H00.19	4	Chalazión	1
2	09/11/2019	H27.8	2	OCP	1
1	09/11/2019	H59.3	4	Post op párpados	1
1	09/11/2019	T15.0	1	Cuerpo extraño	1
1	09/11/2019	H16.0	1	Úlcera corneal	1
1	09/11/2019	H20	6	Uveitis	1
1	09/11/2019	H16.0	1	Úlcera corneal	1
2	09/11/2019	H26.9	2	Catarata	1
1	09/11/2019	H02	4	Alteración palpebral	1
1	09/11/2019	H10.3	0	Conjuntivitis	1
1	09/11/2019	H11.3	0	Hiposfagma	1
1	09/11/2019	T15.0	1	Cuerpo extraño	1
2	09/11/2019	H16.0	1	Úlcera corneal	1
1	09/11/2019	H10.3	0	Conjuntivitis	1
2	09/11/2019	H00.19	4	Chalazión	1
2	09/11/2019	H53	11	No patología	1
2	09/11/2019	H10.3	0	Conjuntivitis	1
1	09/11/2019	H11.3	0	Hiposfagma	1
2	09/11/2019	H20	6	Uveitis	1
1	09/11/2019	H10.3	0	Conjuntivitis	1
1	09/11/2019	H43.1	7	Hemovítreo	1
2	09/11/2019	H16.9	1	Queratitis	1
2	09/11/2019	H10.81	0	Pingueculitis	1
2	09/11/2019	H53.10	11	Problema refractivo	1
1	09/11/2019	H16.0	1	Úlcera corneal	1
1	09/11/2019	H20	6	Uveitis	1

1	09/11/2019	H0.41	1	Ojo seco	1
2	09/11/2019	H17.8	1	Infiltrados corneales	1
2	09/11/2019	H10.3	0	Conjuntivitis	1
1	09/11/2019	T15.0	1	Cuerpo extraño	1
2	09/11/2019	H16.9	1	Queratitis	1
2	09/11/2019	H00.02	4	Orzuelo	1
1	09/11/2019	T15.0	1	Cuerpo extraño	1
1	09/11/2019	H0.41	1	Ojo seco	1
1	09/11/2019	H10.3	0	Conjuntivitis	1
1	09/11/2019	T15.0	1	Cuerpo extraño	1
2	09/11/2019	H16.9	1	Queratitis	1
1	09/11/2019	G45.3	10	Amaurosis fugax	1
1	09/11/2019	T15.0	1	Cuerpo extraño	1
1	09/11/2019	H10.81	4	Pingueculitis	1
1	09/11/2019	H20	6	Uveitis	1
2	10/11/2019	H16.0	1	Úlcera corneal	1
2	10/11/2019	T15.0	1	Cuerpo extraño	1
1	10/11/2019	H16.0	1	Úlcera corneal	1
1	10/11/2019	H53	11	No patología	1
2	10/11/2019	H10.3	0	Conjuntivitis	1
1	10/11/2019	H16.0	1	Úlcera corneal	1
1	10/11/2019	T15.0	1	Cuerpo extraño	1
2	10/11/2019	H17.8	1	Infiltrados corneales	1
2	10/11/2019	H16.9	1	Queratitis	1
2	10/11/2019	H53	11	No patología	1
2	10/11/2019	H17.8	1	Infiltrados corneales	1
2	10/11/2019	H10.3	0	Conjuntivitis	1
2	10/11/2019	H16.9	1	Queratitis	1
1	10/11/2019	H17.8	1	Infiltrados corneales	1
1	10/11/2019	H10.3	0	Conjuntivitis	1
2	10/11/2019	T15.0	1	Cuerpo extraño	1
1	10/11/2019	H02.05	4	Distiquiasis	1
2	10/11/2019	H43.81	7	DVP	1
2	10/11/2019	H11.82	0	Conjuntivocalasia	1
1	10/11/2019	T26	9	Causticación	1
2	10/11/2019	H10.3	0	Conjuntivitis	1
1	10/11/2019	H16.9	1	Queratitis	1
1	10/11/2019	H10.3	0	Conjuntivitis	1
1	10/11/2019	H16.0	1	Úlcera corneal	1
2	10/11/2019	H10.3	0	Conjuntivitis	1
2	10/11/2019	H10.3	0	Conjuntivitis	1
2	10/11/2019	H53.9	11	Alteraciones visuales	1
2	10/11/2019	H11.3	0	Hiposfagma	1
1	10/11/2019	H00.02	4	Orzuelo	1
1	10/11/2019	H17.8	1	Infiltrados corneales	1
2	10/11/2019	H00.19	4	Chalazión	1
2	10/11/2019	H02	4	Alteración palpebral	1
2	10/11/2019	H0.41	1	Ojo seco	1
2	10/11/2019	H16.0	1	Úlcera corneal	1
1	10/11/2019	H16.0	1	Úlcera corneal	1
2	10/11/2019	H16.0	1	Úlcera corneal	1
1	10/11/2019	H00.02	4	Orzuelo	1
2	10/11/2019	H43.1	7	Hemovítreo	1
1	10/11/2019	H16.9	1	Queratitis	1
2	10/11/2019	H10.3	0	Conjuntivitis	1
1	10/11/2019	H16.9	1	Queratitis	1
2	10/11/2019	H10.3	0	Conjuntivitis	1
1	10/11/2019	H01.00	4	Blefaritis	1
1	10/11/2019	H16.0	1	Úlcera corneal	1
2	11/11/2019	H10.3	0	Conjuntivitis	1
1	11/11/2019	H10.3	0	Conjuntivitis	1
2	11/11/2019	T26	9	Causticación	1
1	11/11/2019	H43.1	7	Hemovítreo	1
2	11/11/2019	H35.37	8	MER	1
2	11/11/2019	H10.3	0	Conjuntivitis	1
2	11/11/2019	H43.81	7	DVP	1

2	11/11/2019	H10.3	0	Conjuntivitis	1
1	11/11/2019	H16.0	1	Úlcera corneal	1
2	11/11/2019	H43.1	7	Hemovítreo	1
1	11/11/2019	H01.00	4	Blefaritis	1
2	11/11/2019	H20	6	Uveitis	1
1	11/11/2019	H10.3	0	Conjuntivitis	1
2	11/11/2019	H20	6	Uveitis	1
2	11/11/2019	H11.3	0	Hiposfagma	1
2	11/11/2019	H01.00	4	Blefaritis	1
2	11/11/2019	H43.81	7	DVP	1
2	11/11/2019	H10.3	0	Conjuntivitis	1
2	11/11/2019	H43.81	7	DVP	1
2	11/11/2019	H10.3	0	Conjuntivitis	1
1	11/11/2019	H10.3	0	Conjuntivitis	1
2	11/11/2019	H10.3	0	Conjuntivitis	1
2	11/11/2019	H53.9	11	Alteraciones visuales	1
1	11/11/2019	H10.3	0	Conjuntivitis	1
2	11/11/2019	H01.00	4	Blefaritis	1
1	11/11/2019	H43.81	7	DVP	1
2	11/11/2019	H01.00	4	Blefaritis	1
2	11/11/2019	H11.3	0	Hiposfagma	1
2	11/11/2019	H00.19	4	Chalazión	1
2	11/11/2019	H00.02	4	Orzuelo	1
2	11/11/2019	H53.9	11	Alteraciones visuales	1
2	11/11/2019	H01.00	4	Blefaritis	1
1	11/11/2019	T15.0	1	Cuerpo extraño	1
2	11/11/2019	H10.3	0	Conjuntivitis	1
2	11/11/2019	H11.3	0	Hiposfagma	1
2	11/11/2019	H16.0	1	Úlcera corneal	1
1	11/11/2019	T15.0	1	Cuerpo extraño	1
2	11/11/2019	H35.37	8	MER	1
2	11/11/2019	H10.3	0	Conjuntivitis	1
1	11/11/2019	H11.3	0	Hiposfagma	1
2	11/11/2019	H43.81	7	DVP	1
1	11/11/2019	H10.3	0	Conjuntivitis	1
1	11/11/2019	H43.81	7	DVP	1
1	11/11/2019	T15.0	1	Cuerpo extraño	1
1	11/11/2019	H10.3	0	Conjuntivitis	1
2	11/11/2019	H10.5	4	Blefaroconjuntivitis	1
2	11/11/2019	H15.00	6	Escleritis	1
1	11/11/2019	H16.0	1	Úlcera corneal	1
2	12/11/2019	H00.02	4	Orzuelo	1
1	12/11/2019	H16.9	1	Queratitis	1
2	12/11/2019	H10.3	0	Conjuntivitis	1
2	12/11/2019	H53	11	No patología	1
1	12/11/2019	T15.0	1	Cuerpo extraño	1
1	12/11/2019	H15.1	6	Epiescleritis	1
1	12/11/2019	E11.319	8	Retinopatía diabética	1
1	12/11/2019	H02	4	Alteración palpebral	1
1	12/11/2019	H10.3	0	Conjuntivitis	1
2	12/11/2019	H01.00	4	Blefaritis	1
2	12/11/2019	H00.02	4	Orzuelo	1
2	12/11/2019	H16.9	1	Queratitis	1
2	12/11/2019	T15.0	1	Cuerpo extraño	1
2	12/11/2019	H01.00	4	Blefaritis	1
2	12/11/2019	H11.3	0	Hiposfagma	1
2	12/11/2019	H20	6	Uveitis	1
2	12/11/2019	H43.81	7	DVP	1
1	12/11/2019	H53	11	No patología	1
2	12/11/2019	H02	4	Alteración palpebral	1
2	12/11/2019	H40.9	3	Glaucoma	1
2	12/11/2019	H10.3	0	Conjuntivitis	1
1	12/11/2019	T15.0	1	Cuerpo extraño	1
1	12/11/2019	Alta por fuga	11	Alta por fuga	1
2	12/11/2019	H59.3	5	Postop via lagrimal	1
1	12/11/2019	H10.3	0	Conjuntivitis	1

1	12/11/2019	T15.0	1	Cuerpo extraño	1
2	12/11/2019	H53	11	No patología	1
2	12/11/2019	H02	4	Alteración palpebral	1
1	12/11/2019	T26	9	Causticación	1
2	12/11/2019	T15.0	1	Cuerpo extraño	1
2	12/11/2019	H10.3	0	Conjuntivitis	1
1	12/11/2019	S05.9	9	Traumatismo	1
2	12/11/2019	H53	11	No patología	1
2	12/11/2019	H53	11	No patología	1
1	12/11/2019	S05.9	9	Traumatismo	1
1	12/11/2019	S05.9	9	Traumatismo	1
1	12/11/2019	H01.00	4	Blefaritis	1
1	13/11/2019	T15.0	1	Cuerpo extraño	1
1	13/11/2019	H16.9	1	Queratitis	1
2	13/11/2019	S05.9	9	Traumatismo	1
2	13/11/2019	H43.81	7	DVP	1
2	13/11/2019	H00.03	4	Celulitis preseptal	1
2	13/11/2019	H10.3	0	Conjuntivitis	1
2	13/11/2019	H35.3	8	Maculopatía	1
1	13/11/2019	H53	11	No patología	1
2	13/11/2019	H10.3	0	Conjuntivitis	1
1	13/11/2019	H10.3	0	Conjuntivitis	1
2	13/11/2019	H0.41	1	Ojo seco	1
1	13/11/2019	H10.3	0	Conjuntivitis	1
2	13/11/2019	H27.8	2	OCP	1
2	13/11/2019	H10.3	0	Conjuntivitis	1
1	13/11/2019	H20	6	Uveitis	1
2	13/11/2019	Alta por fuga	11	Alta por fuga	1
2	13/11/2019	H53.10	11	Problema refractivo	1
1	13/11/2019	H40.21	3	Glaucoma agudo	1
1	13/11/2019	H10.3	0	Conjuntivitis	1
2	13/11/2019	H0.41	1	Ojo seco	1
2	13/11/2019	H02	4	Lesión palpebral	1
2	13/11/2019	H16.9	1	Queratitis	1
2	13/11/2019	H16.9	1	Queratitis	1
1	13/11/2019	H16.0	1	Úlcera corneal	1
2	13/11/2019	H43.81	7	DVP	1
2	13/11/2019	H53	11	No patología	1
1	13/11/2019	H16.0	1	Úlcera corneal	1
2	13/11/2019	H0.41	1	Ojo seco	1
2	13/11/2019	H11.3	0	Hiposfagma	1
2	13/11/2019	H33.30	8	Desgarro retiniano	1
1	13/11/2019	H35.3	8	Maculopatía	1
1	13/11/2019	H16.9	1	Queratitis	1
1	13/11/2019	H16.9	1	Queratitis	1
2	13/11/2019	H10.3	0	Conjuntivitis	1
1	13/11/2019	T15.0	1	Cuerpo extraño	1
1	13/11/2019	H16.0	1	Úlcera corneal	1
2	13/11/2019	H16.9	1	Queratitis	1
1	13/11/2019	H10.3	0	Conjuntivitis	1
1	13/11/2019	S05.9	9	Traumatismo	1
2	13/11/2019	H43.81	7	DVP	1
2	13/11/2019	H11.3	0	Hiposfagma	1
2	13/11/2019	H16.0	1	Úlcera corneal	1
2	13/11/2019	H16.9	1	Queratitis	1
1	13/11/2019	H11.3	0	Hiposfagma	1
2	13/11/2019	H10.3	0	Conjuntivitis	1
2	13/11/2019	H16.0	1	Úlcera corneal	1
1	14/11/2019	S05.9	9	Traumatismo	1
2	14/11/2019	H16.0	1	Úlcera corneal	1
2	14/11/2019	H11.3	0	Hiposfagma	1
2	14/11/2019	H10.3	0	Conjuntivitis	1
2	14/11/2019	H53	11	No patología	1
1	14/11/2019	H33.0	8	Desprendimiento de retina	1
1	14/11/2019	H53.9	11	Alteraciones visuales	1
1	14/11/2019	H35.30	8	DMAE	1

1	14/11/2019	H10.3	0	Conjuntivitis	1
2	14/11/2019	H02.05	4	Distiquiasis	1
1	14/11/2019	H16.0	1	Úlcera corneal	1
2	14/11/2019	H00.02	4	Orzuelo	1
2	14/11/2019	H43.81	7	DVP	1
2	14/11/2019	H10.3	0	Conjuntivitis	1
2	14/11/2019	H10.3	0	Conjuntivitis	1
2	14/11/2019	H11.44	0	Quiste conjuntival	1
2	14/11/2019	H04.32	5	Dacriocistitis aguda	1
1	14/11/2019	H33.0	8	Desprendimiento de retina	1
2	14/11/2019	Alta por fuga	11	Alta por fuga	1
1	14/11/2019	H10.3	0	Conjuntivitis	1
2	14/11/2019	H53.9	11	Alteraciones visuales	1
2	14/11/2019	H44.009	8	Endoftalmitis	1
2	14/11/2019	H10.3	0	Conjuntivitis	1
2	14/11/2019	H02	4	Alteración palpebral	1
2	14/11/2019	H43.1	7	Hemovítreo	1
1	14/11/2019	H10.3	0	Conjuntivitis	1
2	14/11/2019	H16.9	1	Queratitis	1
2	14/11/2019	H10.3	0	Conjuntivitis	1
2	14/11/2019	H00.02	4	Orzuelo	1
2	14/11/2019	H01.00	4	Blefaritis	1
2	14/11/2019	H11.3	0	Hiposfagma	1
1	14/11/2019	H10.3	0	Conjuntivitis	1
1	14/11/2019	H16.9	1	Queratitis	1
2	14/11/2019	H35.3	8	Maculopatía	1
1	14/11/2019	H00.02	4	Orzuelo	1
1	14/11/2019	H16.9	1	Queratitis	1
1	14/11/2019	H10.3	0	Conjuntivitis	1
1	14/11/2019	H16.0	1	Úlcera corneal	1
2	14/11/2019	H16.0	3	Atalamia	1
1	14/11/2019	H53	11	No patología	1
2	14/11/2019	H16.9	1	Queratitis	1
1	14/11/2019	H16.9	1	Queratitis	1
1	14/11/2019	G43.109	10	Aura	1
1	14/11/2019	H10.3	0	Conjuntivitis	1
1	14/11/2019	S05.9	9	Traumatismo	1
2	14/11/2019	H20	6	Uveitis	1
1	14/11/2019	H10.3	0	Conjuntivitis	1
1	14/11/2019	T15.0	1	Cuerpo extraño	1
2	14/11/2019	H0.41	1	Ojo seco	1
1	14/11/2019	H16.9	1	Queratitis	1
2	14/11/2019	H15.1	6	Epiescleritis	1
1	14/11/2019	H43.81	7	DVP	1
2	14/11/2019	H16.0	1	Úlcera corneal	1
1	14/11/2019	H00.02	4	Orzuelo	1
1	14/11/2019	H16.0	1	Úlcera corneal	1
2	14/11/2019	H16.9	1	Queratitis	1
1	14/11/2019	H11.3	0	Hiposfagma	1
1	15/11/2019	H16.9	1	Queratitis	1
2	15/11/2019	H16.9	1	Queratitis	1
1	15/11/2019	H53	11	No patología	1
1	15/11/2019	T15.0	1	Cuerpo extraño	1
1	15/11/2019	H16.9	1	Queratitis	1
2	15/11/2019	H10.3	0	Conjuntivitis	1
2	15/11/2019	H27.8	2	OCP	1
1	15/11/2019	H02	4	Lesión palpebral	1
1	15/11/2019	H10.3	0	Conjuntivitis	1
1	15/11/2019	H10.3	0	Conjuntivitis	1
1	15/11/2019	H11.3	0	Hiposfagma	1
1	15/11/2019	H11.3	0	Hiposfagma	1
2	15/11/2019	H16.9	1	Queratitis	1
2	15/11/2019	H00.02	4	Orzuelo	1
2	15/11/2019	H10.3	0	Conjuntivitis	1
2	15/11/2019	H53	11	No patología	1
2	15/11/2019	H16.9	1	Queratitis	1

1	15/11/2019	H11.3	0	Hiposfagma	1
2	15/11/2019	H10.3	0	Conjuntivitis	1
1	15/11/2019	H18.50	1	Distrofia corneal	1
2	15/11/2019	H33.30	8	Desgarro retiniano	1
2	15/11/2019	H15.1	6	Epiescleritis	1
2	15/11/2019	H27.8	2	OCP	1
2	15/11/2019	H10.3	0	Conjuntivitis	1
1	15/11/2019	H33.0	8	Desprendimiento de retina	1
1	15/11/2019	H10.3	0	Conjuntivitis	1
2	15/11/2019	H10.3	0	Conjuntivitis	1
2	15/11/2019	H00.02	4	Orzuelo	1
2	15/11/2019	H35.30	8	DMAE	1
2	15/11/2019	H53	11	No patología	1
2	15/11/2019	H27.8	2	OCP	1
2	15/11/2019	H43.81	7	DVP	1
2	15/11/2019	H16.0	1	Úlcera corneal	1
1	15/11/2019	H16.0	1	Úlcera corneal	1
1	15/11/2019	H10.3	0	Conjuntivitis	1
1	15/11/2019	T15.0	1	Cuerpo extraño	1
1	15/11/2019	H20	6	Uveitis	1
2	15/11/2019	H35.30	8	DMAE	1
2	15/11/2019	H11.3	0	Hiposfagma	1
1	15/11/2019	E11.319	8	Retinopatía diabética	1
2	15/11/2019	H11.00	0	Pterigium	1
1	15/11/2019	S05.9	9	Traumatismo	1
2	15/11/2019	H35.30	8	DMAE	1
2	15/11/2019	H11.44	0	Quiste conjuntival	1
1	15/11/2019	S05.9	9	Traumatismo	1
2	15/11/2019	H53	11	No patología	1
1	15/11/2019	H10.3	0	Conjuntivitis	1
1	15/11/2019	H10.3	0	Conjuntivitis	1
2	15/11/2019	H10.3	0	Conjuntivitis	1
1	15/11/2019	H10.3	0	Conjuntivitis	1
2	15/11/2019	H16.9	1	Queratitis	1
2	15/11/2019	H16.9	1	Queratitis	1
1	15/11/2019	T15.0	1	Cuerpo extraño	1
1	16/11/2019	H16.9	1	Queratitis	1
1	16/11/2019	H16.0	1	Úlcera corneal	1
1	16/11/2019	H10.3	0	Conjuntivitis	1
1	16/11/2019	S05.30	9	Laceración conjuntival	1
2	16/11/2019	H00.02	4	Orzuelo	1
1	16/11/2019	H10.3	0	Conjuntivitis	1
1	16/11/2019	H17.8	1	Infiltrados corneales	1
2	16/11/2019	H10.3	0	Conjuntivitis	1
1	16/11/2019	H10.3	0	Conjuntivitis	1
1	16/11/2019	H17.8	1	Infiltrados corneales	1
2	16/11/2019	H44.009	8	Endoftalmitis	1
2	16/11/2019	H10.3	0	Conjuntivitis	1
2	16/11/2019	H00.02	4	Orzuelo	1
1	16/11/2019	T15.0	1	Cuerpo extraño	1
2	16/11/2019	H10.3	0	Conjuntivitis	1
1	16/11/2019	H16.0	1	Úlcera corneal	1
1	16/11/2019	H10.3	0	Conjuntivitis	1
2	16/11/2019	H10.3	0	Conjuntivitis	1
2	16/11/2019	H16.0	1	Úlcera corneal	1
2	16/11/2019	H59.3	5	Postop via lagrimal	1
2	16/11/2019	H16.0	1	Úlcera corneal	1
1	16/11/2019	H16.9	1	Queratitis	1
1	16/11/2019	T15.0	1	Cuerpo extraño	1
1	16/11/2019	H53	11	No patología	1
2	16/11/2019	H11.3	0	Hiposfagma	1
1	16/11/2019	H53	11	No patología	1
2	16/11/2019	H49.9	10	Parálisis oculomotora	1
2	16/11/2019	H0.41	1	Ojo seco	1
1	16/11/2019	H00.02	4	Orzuelo	1
2	16/11/2019	H10.3	0	Conjuntivitis	1

2	16/11/2019	H00.19	4	Chalazión	1
1	16/11/2019	T15.0	1	Cuerpo extraño	1
2	16/11/2019	H16.9	1	Queratitis	1
1	16/11/2019	H16.0	1	Úlcera corneal	1
1	16/11/2019	H16.9	1	Queratitis	1
1	16/11/2019	S05.9	9	Traumatismo	1
1	16/11/2019	T15.0	1	Cuerpo extraño	1
1	16/11/2019	T15.0	1	Cuerpo extraño	1
1	16/11/2019	H53	11	No patología	1
1	16/11/2019	T15.0	1	Cuerpo extraño	1
1	16/11/2019	T15.0	1	Cuerpo extraño	1
1	16/11/2019	H10.3	0	Conjuntivitis	1
1	16/11/2019	H16.9	1	Queratitis	1
1	16/11/2019	H53	11	No patología	1
2	16/11/2019	H16.0	1	Úlcera corneal	1
1	17/11/2019	T15.0	1	Cuerpo extraño	1
2	17/11/2019	H16.0	1	Úlcera corneal	1
1	17/11/2019	H16.0	1	Úlcera corneal	1
1	17/11/2019	H16.0	1	Úlcera corneal	1
2	17/11/2019	H04.32	5	Dacriocistitis aguda	1
2	17/11/2019	H00.02	4	Orzuelo	1
1	17/11/2019	H16.0	1	Úlcera corneal	1
2	17/11/2019	H18.20	1	Edema corneal	1
1	17/11/2019	H10.3	0	Conjuntivitis	1
1	17/11/2019	H33.30	8	Desgarro retiniano	1
2	17/11/2019	T15.0	1	Cuerpo extraño	1
2	17/11/2019	H43.81	7	DVP	1
2	17/11/2019	H11.3	0	Hiposfagma	1
2	17/11/2019	H15.00	6	Escleritis	1
2	17/11/2019	H10.3	0	Conjuntivitis	1
2	17/11/2019	H11.3	0	Hiposfagma	1
2	17/11/2019	H04.32	5	Dacriocistitis aguda	1
2	17/11/2019	H16.0	1	Úlcera corneal	1
1	17/11/2019	T15.0	1	Cuerpo extraño	1
2	17/11/2019	H00.02	4	Orzuelo	1
1	17/11/2019	H16.0	1	Úlcera corneal	1
2	17/11/2019	H16.0	1	Úlcera corneal	1
1	17/11/2019	T15.0	1	Cuerpo extraño	1
2	17/11/2019	H10.3	0	Conjuntivitis	1
1	17/11/2019	H00.02	4	Orzuelo	1
1	17/11/2019	H16.0	1	Úlcera corneal	1
1	17/11/2019	H16.0	1	Úlcera corneal	1
1	17/11/2019	S05.9	9	Traumatismo	1
1	17/11/2019	T15.0	1	Cuerpo extraño	1
2	17/11/2019	H30.9	6	Uveitis posterior	1
1	17/11/2019	H11.3	0	Hiposfagma	1
2	17/11/2019	H16.0	1	Úlcera corneal	1
1	17/11/2019	H11.3	0	Hiposfagma	1
1	17/11/2019	H16.0	1	Úlcera corneal	1
1	17/11/2019	H16.0	1	Úlcera corneal	1
1	17/11/2019	H10.3	0	Conjuntivitis	1
2	17/11/2019	H43.81	7	DVP	1
1	17/11/2019	H02	4	Alteración palpebral	1
2	18/11/2019	Alta por fuga	11	Alta por fuga	1
1	18/11/2019	H11.00	0	Pterigium	1
1	18/11/2019	H26.9	2	Catarata	1
2	18/11/2019	H00.02	4	Orzuelo	1
1	18/11/2019	H16.9	1	Queratitis	1
2	18/11/2019	H53	11	No patología	1
2	18/11/2019	T15.0	1	Cuerpo extraño	1
1	18/11/2019	H11.3	0	Hiposfagma	1
1	18/11/2019	H53	11	No patología	1
1	18/11/2019	B00.1	4	Dermatitis herpética	1
2	18/11/2019	H16.9	1	Queratitis	1
2	18/11/2019	H53	11	No patología	1
2	18/11/2019	H10.3	0	Conjuntivitis	1

2	18/11/2019	H53	11	No patología	1
2	18/11/2019	H01.00	4	Blefaritis	1
1	18/11/2019	H17.8	1	Infiltrados corneales	1
1	18/11/2019	H16.9	1	Queratitis	1
1	18/11/2019	Alta por fuga	11	Alta por fuga	1
2	18/11/2019	H44.009	8	Endoftalmitis	1
2	18/11/2019	H43.81	7	DVP	1
2	18/11/2019	H53.9	11	Alteraciones visuales	1
2	18/11/2019	H10.3	0	Conjuntivitis	1
2	18/11/2019	H11.3	0	Hiposfagma	1
1	18/11/2019	H05.01	4	Celulitis orbitaria	1
1	18/11/2019	H04.32	5	Dacriocistitis aguda	1
2	18/11/2019	H02	4	Lesión palpebral	1
2	18/11/2019	H43.81	7	DVP	1
1	18/11/2019	H11.3	0	Hiposfagma	1
1	18/11/2019	H10.5	4	Blefaroconjuntivitis	1
2	18/11/2019	H16.9	1	Queratitis	1
2	18/11/2019	H27.8	2	OCP	1
2	18/11/2019	H10.3	0	Conjuntivitis	1
2	18/11/2019	H10.3	0	Conjuntivitis	1
1	18/11/2019	H00.19	4	Chalazión	1
2	18/11/2019	H35.3	8	Maculopatía	1
1	18/11/2019	T15.0	1	Cuerpo extraño	1
1	18/11/2019	H20	6	Uveitis	1
1	18/11/2019	H20	6	Uveitis	1
1	18/11/2019	H10.3	0	Conjuntivitis	1
1	18/11/2019	H20	6	Uveitis	1
1	18/11/2019	H16.9	1	Queratitis	1
1	18/11/2019	H16.0	1	Úlcera corneal	1
1	18/11/2019	T15.0	1	Cuerpo extraño	1
2	19/11/2019	H53	11	No patología	1
2	19/11/2019	G43.109	10	Aura	1
1	19/11/2019	T15.0	1	Cuerpo extraño	1
1	19/11/2019	H00.19	4	Chalazión	1
2	19/11/2019	H00.02	4	Orzuelo	1
2	19/11/2019	H43.81	7	DVP	1
1	19/11/2019	H16.0	1	Úlcera corneal	1
2	19/11/2019	H16.9	1	Queratitis	1
1	19/11/2019	H10.3	0	Conjuntivitis	1
2	19/11/2019	H43.81	7	DVP	1
2	19/11/2019	H10.3	0	Conjuntivitis	1
1	19/11/2019	H27.8	2	OCP	1
1	19/11/2019	H11.3	0	Hiposfagma	1
1	19/11/2019	H16.0	1	Úlcera corneal	1
2	19/11/2019	H16.0	1	Úlcera corneal	1
2	19/11/2019	H53	11	No patología	1
2	19/11/2019	G43.109	10	Aura	1
1	19/11/2019	H11.3	0	Hiposfagma	1
2	19/11/2019	H16.0	1	Úlcera corneal	1
2	19/11/2019	H01.00	4	Blefaritis	1
1	19/11/2019	T15.0	1	Cuerpo extraño	1
2	19/11/2019	H16.9	1	Queratitis	1
2	19/11/2019	H16.0	1	Úlcera corneal	1
2	19/11/2019	H01.00	4	Blefaritis	1
2	19/11/2019	H11.3	0	Hiposfagma	1
1	19/11/2019	H16.9	1	Queratitis	1
1	19/11/2019	S05.9	9	Traumatismo	1
1	19/11/2019	H16.9	1	Queratitis	1
2	19/11/2019	H15.1	6	Epiescleritis	1
2	19/11/2019	G43.109	10	Aura	1
2	19/11/2019	H10.3	0	Conjuntivitis	1
1	19/11/2019	H02	4	Alteración palpebral	1
1	19/11/2019	H00.02	4	Orzuelo	1
2	19/11/2019	H00.02	4	Orzuelo	1
2	19/11/2019	H10.3	0	Conjuntivitis	1
2	19/11/2019	H10.3	0	Conjuntivitis	1

1	19/11/2019	H16.0	1	Úlcera corneal	1
2	19/11/2019	H11.3	0	Hiposfagma	1
2	19/11/2019	H16.0	1	Úlcera corneal	1
1	19/11/2019	H00.02	4	Orzuelo	1
1	19/11/2019	H02.05	4	Distiquiasis	1
2	19/11/2019	H11.3	0	Hiposfagma	1
1	20/11/2019	H16.9	1	Queratitis	1
2	20/11/2019	H10.3	0	Conjuntivitis	1
1	20/11/2019	H16.9	1	Queratitis	1
1	20/11/2019	H00.02	4	Orzuelo	1
2	20/11/2019	H43.81	7	DVP	1
2	20/11/2019	H0.41	1	Ojo seco	1
2	20/11/2019	H02	4	Alteración palpebral	1
2	20/11/2019	H17.8	1	Infiltrados corneales	1
2	20/11/2019	G45.3	10	Amaurosis fugax	1
2	20/11/2019	S05.9	9	Traumatismo	1
1	20/11/2019	H18.20	1	Edema corneal	1
1	20/11/2019	H16.0	1	Úlcera corneal	1
2	20/11/2019	H01.00	4	Blefaritis	1
2	20/11/2019	H20	6	Uveitis	1
2	20/11/2019	H16.9	1	Queratitis	1
2	20/11/2019	H02	4	Lesión palpebral	1
2	20/11/2019	H35.30	8	DMAE	1
2	20/11/2019	H27.8	2	OCP	1
2	20/11/2019	H33.0	8	Desprendimiento de retina	1
2	20/11/2019	H16.9	1	Queratitis	1
2	20/11/2019	H27.8	2	OCP	1
2	20/11/2019	H20	6	Uveitis	1
2	20/11/2019	H11.3	0	Hiposfagma	1
2	20/11/2019	H01.00	4	Blefaritis	1
2	20/11/2019	H00.02	4	Orzuelo	1
1	20/11/2019	H10.3	0	Conjuntivitis	1
1	20/11/2019	S05.30	9	Laceración conjuntival	1
1	20/11/2019	H35.30	8	DMAE	1
2	20/11/2019	S05.9	9	Traumatismo	1
2	20/11/2019	H11.3	0	Hiposfagma	1
1	20/11/2019	S05.00	9	Abrasión conjuntival	1
2	20/11/2019	H43.1	7	Hemovítreo	1
2	20/11/2019	H43.81	7	DVP	1
1	20/11/2019	H53	11	No patología	1
2	20/11/2019	H16.9	1	Queratitis	1
1	20/11/2019	T15.0	1	Cuerpo extraño	1
2	20/11/2019	H00.02	4	Orzuelo	1
2	20/11/2019	H11.3	0	Hiposfagma	1
1	20/11/2019	H0.41	1	Ojo seco	1
1	20/11/2019	H16.9	1	Queratitis	1
1	20/11/2019	H11.3	0	Hiposfagma	1
2	20/11/2019	H53	11	No patología	1
2	20/11/2019	H35.3	8	Maculopatía	1
2	20/11/2019	H00.02	4	Orzuelo	1
1	20/11/2019	H10.3	0	Conjuntivitis	1
1	21/11/2019	Alta por fuga	11	Alta por fuga	1
1	21/11/2019	H18.50	1	Distrofia corneal	1
1	21/11/2019	H00.03	4	Celulitis preseptal	1
1	21/11/2019	H43.1	7	Hemovítreo	1
1	21/11/2019	H01.00	4	Blefaritis	1
2	21/11/2019	H43.81	7	DVP	1
2	21/11/2019	H20	6	Uveitis	1
2	21/11/2019	H10.3	0	Conjuntivitis	1
1	21/11/2019	H10.3	0	Conjuntivitis	1
2	21/11/2019	H43.81	7	DVP	1
2	21/11/2019	H02	4	Alteración palpebral	1
2	21/11/2019	H15.1	6	Epiescleritis	1
1	21/11/2019	H01.00	4	Blefaritis	1
2	21/11/2019	H01.00	4	Blefaritis	1
1	21/11/2019	H00.02	4	Orzuelo	1

2	21/11/2019	H10.3	0	Conjuntivitis		1
2	21/11/2019	H01.00	4	Blefaritis		1
1	21/11/2019	H43.81	7	DVP		1
2	21/11/2019	H01.00	4	Blefaritis		1
2	21/11/2019	H40.9	3	Glaucoma		1
2	21/11/2019	H01.00	4	Blefaritis		1
1	21/11/2019	H01.00	4	Blefaritis		1
1	21/11/2019	S05.9	9	Traumatismo		1
1	21/11/2019	H10.3	0	Conjuntivitis		1
2	21/11/2019	T15.0	1	Cuerpo extraño		1
2	21/11/2019	H35.3	8	Maculopatía		1
2	21/11/2019	H01.00	4	Blefaritis		1
2	21/11/2019	H16.9	1	Queratitis		1
1	21/11/2019	H11.3	0	Hiposfagma		1
2	21/11/2019	H43.81	7	DVP		1
2	21/11/2019	H10.3	0	Conjuntivitis		1
2	21/11/2019	H53	11	No patología		1
2	21/11/2019	H40.9	3	Glaucoma		1
1	21/11/2019	H43.81	7	DVP		1
2	21/11/2019	H01.00	4	Blefaritis		1
2	21/11/2019	H10.3	0	Conjuntivitis		1
1	21/11/2019	H16.9	1	Queratitis		1
1	21/11/2019	H00.02	4	Orzuelo		1
2	21/11/2019	H0.41	1	Ojo seco		1
2	21/11/2019	H16.0	1	Úlcera corneal		1
1	21/11/2019	T15.0	1	Cuerpo extraño		1
2	21/11/2019	H16.9	1	Queratitis		1
2	21/11/2019	H16.0	1	Úlcera corneal		1
2	21/11/2019	G43.109	10	Aura		1
1	21/11/2019	S05.9	9	Traumatismo		1
2	21/11/2019	S05.9	9	Traumatismo		1
1	21/11/2019	H01.00	4	Blefaritis		1
2	21/11/2019	S05.9	9	Traumatismo		1
1	22/11/2019	T15.0	1	Cuerpo extraño		1
1	22/11/2019	H10.81	0	Pingueculitis		1
2	22/11/2019	H10.3	0	Conjuntivitis		1
2	22/11/2019	H10.3	0	Conjuntivitis		1
1	22/11/2019	S05.30	9	Laceración conjuntival		1
1	22/11/2019	H00.19	4	Chalazión		1
2	22/11/2019	H02	4	Alteración palpebral		1
2	22/11/2019	H20	6	Uveitis		1
2	22/11/2019	H04.32	5	Dacriocistitis aguda		1
1	22/11/2019	H10.3	0	Conjuntivitis		1
1	22/11/2019	H16.9	1	Queratitis		1
2	22/11/2019	H43.81	7	DVP		1
2	22/11/2019	H16.9	1	Queratitis		1
2	22/11/2019	H16.9	1	Queratitis		1
1	22/11/2019	H11.3	0	Hiposfagma		1
1	22/11/2019	T15.0	1	Cuerpo extraño		1
1	22/11/2019	H31.42	8	Coroidopatía serosa central		1
1	22/11/2019	T15.0	1	Cuerpo extraño		1
1	22/11/2019	H11.44	0	Quiste conjuntival		1
1	22/11/2019	T15.0	1	Cuerpo extraño		1
2	22/11/2019	H53.10	11	Problema refractivo		1
1	22/11/2019	H11.3	0	Hiposfagma		1
2	22/11/2019	H53	11	No patología		1
2	22/11/2019	H16.0	1	Úlcera corneal		1
2	22/11/2019	H16.0	1	Úlcera corneal		1
2	22/11/2019	H16.9	1	Queratitis		1
1	22/11/2019	H16.9	1	Queratitis		1
1	22/11/2019	T15.0	1	Cuerpo extraño		1
2	22/11/2019	H11.3	0	Hiposfagma		1
1	22/11/2019	H02.05	4	Distiquiasis		1
2	22/11/2019	H53	11	No patología		1
2	22/11/2019	H15.1	6	Epiescleritis		1
1	22/11/2019	H16.9	1	Queratitis		1

1	23/11/2019	H16.9	1	Queratitis	1
1	23/11/2019	T15.0	1	Cuerpo extraño	1
1	23/11/2019	H10.3	0	Conjuntivitis	1
1	23/11/2019	H16.9	1	Queratitis	1
1	23/11/2019	H0.41	1	Ojo seco	1
1	23/11/2019	H02.05	4	Distiquiasis	1
1	23/11/2019	H11.00	0	Pterigium	1
2	23/11/2019	H02.05	4	Distiquiasis	1
2	23/11/2019	H10.3	0	Conjuntivitis	1
2	23/11/2019	H35.3	8	Maculopatía	1
1	23/11/2019	H10.3	0	Conjuntivitis	1
1	23/11/2019	H01.00	4	Blefaritis	1
1	23/11/2019	H11.3	0	Hiposfagma	1
2	23/11/2019	H10.3	0	Conjuntivitis	1
2	23/11/2019	H00.02	4	Orzuelo	1
2	23/11/2019	H16.9	1	Queratitis	1
1	23/11/2019	H43.1	7	Hemovítreo	1
1	23/11/2019	H16.9	1	Queratitis	1
1	23/11/2019	H00.02	4	Orzuelo	1
2	23/11/2019	H16.9	1	Queratitis	1
2	23/11/2019	H16.9	1	Queratitis	1
2	23/11/2019	H00.02	4	Orzuelo	1
1	23/11/2019	H02.05	4	Distiquiasis	1
2	23/11/2019	H10.3	0	Conjuntivitis	1
1	23/11/2019	H16.9	1	Queratitis	1
1	23/11/2019	T15.0	1	Cuerpo extraño	1
2	23/11/2019	H35.30	8	DMAE	1
2	23/11/2019	H53	11	No patología	1
1	23/11/2019	T15.0	1	Cuerpo extraño	1
2	23/11/2019	H43.81	7	DVP	1
2	23/11/2019	H02	4	Lesión palpebral	1
1	23/11/2019	H10.3	0	Conjuntivitis	1
1	23/11/2019	H59.3	4	Post op párpados	1
2	23/11/2019	H11.3	0	Hiposfagma	1
1	23/11/2019	H11.3	0	Hiposfagma	1
2	23/11/2019	H10.3	0	Conjuntivitis	1
2	23/11/2019	H43.81	7	DVP	1
1	23/11/2019	H16.0	1	Úlcera corneal	1
1	23/11/2019	H27.8	2	OCP	1
1	23/11/2019	H16.9	1	Queratitis	1
1	23/11/2019	H16.9	1	Queratitis	1
2	23/11/2019	H33.30	8	Desgarro retiniano	1
1	23/11/2019	H16.0	1	Úlcera corneal	1
2	23/11/2019	H10.3	0	Conjuntivitis	1
2	23/11/2019	H17.8	1	Infiltrados corneales	1
1	23/11/2019	H10.3	0	Conjuntivitis	1
1	24/11/2019	H43.81	7	DVP	1
2	24/11/2019	T15.0	1	Cuerpo extraño	1
2	24/11/2019	H10.3	0	Conjuntivitis	1
2	24/11/2019	H11.3	0	Hiposfagma	1
1	24/11/2019	H01.00	4	Blefaritis	1
1	24/11/2019	H0.41	1	Ojo seco	1
2	24/11/2019	H10.3	0	Conjuntivitis	1
2	24/11/2019	H01.00	4	Blefaritis	1
1	24/11/2019	H16.9	1	Queratitis	1
1	24/11/2019	H43.81	7	DVP	1
1	24/11/2019	H10.3	0	Conjuntivitis	1
2	24/11/2019	H18.50	1	Distrofia corneal	1
1	24/11/2019	H00.02	4	Orzuelo	1
2	24/11/2019	H16.9	1	Queratitis	1
2	24/11/2019	H16.9	1	Queratitis	1
2	24/11/2019	H15.1	6	Epiescleritis	1
1	24/11/2019	H16.9	1	Queratitis	1
1	24/11/2019	H10.3	0	Conjuntivitis	1
2	24/11/2019	H10.3	0	Conjuntivitis	1
1	24/11/2019	H53.9	11	Alteraciones visuales	1

2	24/11/2019	H00.02	4	Orzuelo	1
1	24/11/2019	H16.0	1	Úlcera corneal	1
2	24/11/2019	H11.3	0	Hiposfagma	1
2	24/11/2019	H10.3	0	Conjuntivitis	1
1	24/11/2019	H16.9	1	Queratitis	1
2	24/11/2019	S05.9	9	Traumatismo	1
1	24/11/2019	H53.10	11	Problema refractivo	1
1	24/11/2019	T15.0	1	Cuerpo extraño	1
1	24/11/2019	T15.0	1	Cuerpo extraño	1
2	24/11/2019	G43.9	10	Migraña	1
1	24/11/2019	H46	10	Neuritis óptica	1
2	24/11/2019	H10.3	0	Conjuntivitis	1
2	24/11/2019	H20	6	Uveitis	1
2	24/11/2019	H53.9	11	Alteraciones visuales	1
2	24/11/2019	H16.9	1	Queratitis	1
2	24/11/2019	H16.0	1	Úlcera corneal	1
2	24/11/2019	H10.3	0	Conjuntivitis	1
2	24/11/2019	H10.3	0	Conjuntivitis	1
2	24/11/2019	T15.0	1	Cuerpo extraño	1
1	24/11/2019	H10.3	0	Conjuntivitis	1
1	24/11/2019	H10.3	0	Conjuntivitis	1
1	24/11/2019	S05.9	9	Traumatismo	1
1	24/11/2019	H10.3	0	Conjuntivitis	1
1	24/11/2019	H10.3	0	Conjuntivitis	1
1	24/11/2019	H16.0	1	Úlcera corneal	1
2	25/11/2019	H16.9	1	Queratitis	1
2	25/11/2019	H16.9	1	Queratitis	1
2	25/11/2019	H10.3	0	Conjuntivitis	1
2	25/11/2019	H33.0	8	Desprendimiento de retina	1
1	25/11/2019	H10.3	0	Conjuntivitis	1
2	25/11/2019	H0.41	1	Ojo seco	1
1	25/11/2019	H11.3	0	Hiposfagma	1
2	25/11/2019	H01.00	4	Blefaritis	1
1	25/11/2019	G43.109	10	Aura	1
2	25/11/2019	H43.81	7	DVP	1
2	25/11/2019	H0.41	1	Ojo seco	1
1	25/11/2019	H16.9	1	Queratitis	1
1	25/11/2019	H35.30	8	DMAE	1
2	25/11/2019	H26.9	2	Catarata	1
1	25/11/2019	H53.10	11	Problema refractivo	1
1	25/11/2019	H40.5	3	Glaucoma secundario	1
2	25/11/2019	H01.00	4	Blefaritis	1
2	25/11/2019	H10.3	0	Conjuntivitis	1
1	25/11/2019	T15.0	1	Cuerpo extraño	1
2	25/11/2019	H33.0	8	Desprendimiento de retina	1
2	25/11/2019	H00.02	4	Orzuelo	1
2	25/11/2019	H10.3	0	Conjuntivitis	1
1	25/11/2019	H17.8	1	Infiltrados corneales	1
2	25/11/2019	B00.1	4	Dermatitis herpética	1
2	25/11/2019	T26.4	9	Quemadura	1
1	25/11/2019	H00.02	4	Orzuelo	1
1	25/11/2019	H16.9	1	Queratitis	1
2	25/11/2019	H53.9	11	Alteraciones visuales	1
2	25/11/2019	H11.3	0	Hiposfagma	1
1	25/11/2019	H53.9	11	Alteraciones visuales	1
1	25/11/2019	H35.30	8	DMAE	1
1	25/11/2019	H53	11	No patología	1
1	25/11/2019	H00.02	4	Orzuelo	1
2	25/11/2019	H10.3	0	Conjuntivitis	1
1	25/11/2019	H43.81	7	DVP	1
2	25/11/2019	H35.30	8	DMAE	1
2	25/11/2019	H16.9	1	Queratitis	1
2	25/11/2019	H10.3	0	Conjuntivitis	1
1	25/11/2019	H16.9	1	Queratitis	1
1	25/11/2019	B00.1	4	Dermatitis herpética	1
2	25/11/2019	H16.9	1	Queratitis	1

2	25/11/2019	H0.41	1	Ojo seco	1
1	25/11/2019	H16.9	1	Queratitis	1
2	25/11/2019	H16.9	1	Queratitis	1
1	25/11/2019	H10.3	0	Conjuntivitis	1
1	25/11/2019	T15.0	1	Cuerpo extraño	1
2	25/11/2019	H40.5	3	Glaucoma secundario	1
1	25/11/2019	T15.0	1	Cuerpo extraño	1
1	25/11/2019	S05.9	9	Traumatismo	1
1	26/11/2019	H00.02	4	Orzuelo	1
2	26/11/2019	H02	4	Lesión palpebral	1
1	26/11/2019	H16.9	1	Queratitis	1
1	26/11/2019	H02	4	Alteración palpebral	1
2	26/11/2019	H16.0	1	Úlcera corneal	1
2	26/11/2019	H11.3	0	Hiposfagma	1
2	26/11/2019	H16.9	1	Queratitis	1
2	26/11/2019	H04.32	5	Dacriocistitis aguda	1
1	26/11/2019	H16.0	1	Úlcera corneal	1
1	26/11/2019	H10.3	0	Conjuntivitis	1
1	26/11/2019	H50.9	11	Estrabismo	1
2	26/11/2019	H53.9	11	Alteraciones visuales	1
1	26/11/2019	H35.30	8	DMAE	1
2	26/11/2019	H43.81	7	DVP	1
2	26/11/2019	H00.02	4	Orzuelo	1
1	26/11/2019	H43.81	7	DVP	1
2	26/11/2019	H10.3	0	Conjuntivitis	1
2	26/11/2019	H16.9	1	Queratitis	1
1	26/11/2019	H11.3	0	Hiposfagma	1
2	26/11/2019	H11.3	0	Hiposfagma	1
1	26/11/2019	H16.9	1	Queratitis	1
1	26/11/2019	H31.42	8	Coroidopatía serosa central	1
2	26/11/2019	H18.20	1	Edema corneal	1
2	26/11/2019	H35.30	8	DMAE	1
2	26/11/2019	H11.3	0	Hiposfagma	1
2	26/11/2019	H01.00	4	Blefaritis	1
2	26/11/2019	H20	6	Uveitis	1
1	26/11/2019	H16.9	1	Queratitis	1
2	26/11/2019	H16.9	1	Queratitis	1
1	26/11/2019	S05.9	9	Traumatismo	1
2	26/11/2019	H20	6	Uveitis	1
1	26/11/2019	H20	6	Uveitis	1
2	26/11/2019	H04.32	5	Dacriocistitis aguda	1
1	26/11/2019	H31.42	8	Coroidopatía serosa central	1
1	26/11/2019	H18.20	1	Edema corneal	1
2	26/11/2019	H17.8	1	Infiltrados corneales	1
2	26/11/2019	H16.9	1	Queratitis	1
2	26/11/2019	H0.41	1	Ojo seco	1
1	26/11/2019	H16.0	1	Úlcera corneal	1
2	26/11/2019	H53.9	11	Alteraciones visuales	1
1	26/11/2019	H10.3	0	Conjuntivitis	1
2	26/11/2019	H53	11	No patología	1
1	26/11/2019	H00.02	4	Orzuelo	1
2	26/11/2019	B00.1	4	Dermatitis herpética	1
1	26/11/2019	G43.9	10	Migraña	1
1	26/11/2019	H53	11	No patología	1
2	26/11/2019	H53.9	11	Alteraciones visuales	1
1	26/11/2019	H10.3	0	Conjuntivitis	1
2	26/11/2019	H10.3	0	Conjuntivitis	1
2	26/11/2019	S05.9	9	Traumatismo	1
2	26/11/2019	H04.32	5	Dacriocistitis aguda	1
2	26/11/2019	H00.02	4	Orzuelo	1
2	27/11/2019	G43.9	10	Migraña	1
2	27/11/2019	H33.30	8	Desgarro retiniano	1
1	27/11/2019	T15.0	1	Cuerpo extraño	1
1	27/11/2019	S05.6	9	Perforación ocular	1
2	27/11/2019	T15.0	1	Cuerpo extraño	1
1	27/11/2019	S05.9	9	Traumatismo	1

2	27/11/2019	H01.00	4	Blefaritis	1
1	27/11/2019	H02.05	4	Distiquiasis	1
1	27/11/2019	H10.5	4	Blefaroconjuntivitis	1
1	27/11/2019	H15.1	6	Epiescleritis	1
1	27/11/2019	B58.0	8	Toxoplasmosis	1
2	27/11/2019	H43.1	7	Hemovítreo	1
2	27/11/2019	H33.0	8	Desprendimiento de retina	1
1	27/11/2019	H10.3	0	Conjuntivitis	1
1	27/11/2019	H11.3	0	Hiposfagma	1
2	27/11/2019	H00.02	4	Orzuelo	1
1	27/11/2019	H20	6	Uveitis	1
2	27/11/2019	H43.81	7	DVP	1
2	27/11/2019	H11.3	0	Hiposfagma	1
1	27/11/2019	S05.9	9	Traumatismo	1
2	27/11/2019	H16.9	1	Queratitis	1
2	27/11/2019	T15.0	1	Cuerpo extraño	1
2	27/11/2019	H01.00	4	Blefaritis	1
2	27/11/2019	H43.81	7	DVP	1
1	27/11/2019	H11.3	0	Hiposfagma	1
1	27/11/2019	S05.9	9	Traumatismo	1
1	27/11/2019	H33.30	8	Desgarro retiniano	1
2	27/11/2019	H01.00	4	Blefaritis	1
1	27/11/2019	H33.30	8	Desgarro retiniano	1
1	27/11/2019	H16.0	1	Úlcera corneal	1
1	27/11/2019	H35.3	8	Maculopatía	1
2	27/11/2019	H10.3	0	Conjuntivitis	1
1	27/11/2019	H16.9	1	Queratitis	1
1	27/11/2019	H16.0	1	Úlcera corneal	1
1	27/11/2019	Alta por fuga	11	Alta por fuga	1
2	27/11/2019	T15.0	1	Cuerpo extraño	1
1	27/11/2019	H20	6	Uveitis	1
2	27/11/2019	H35.30	8	DMAE	1
2	27/11/2019	H53	11	No patología	1
1	27/11/2019	S05.9	9	Traumatismo	1
2	27/11/2019	H53.9	11	Alteraciones visuales	1
1	27/11/2019	S05.6	9	Perforación ocular	1
2	27/11/2019	H16.9	1	Queratitis	1
2	27/11/2019	H10.5	4	Blefaroconjuntivitis	1
1	27/11/2019	T15.0	1	Cuerpo extraño	1
2	27/11/2019	H11.3	0	Hiposfagma	1
2	27/11/2019	Alta por fuga	11	Alta por fuga	1
2	27/11/2019	T26	9	Causticación	1
1	27/11/2019	H10.3	0	Conjuntivitis	1
1	27/11/2019	H02	4	Lesión palpebral	1
2	27/11/2019	H0.41	1	Ojo seco	1
1	27/11/2019	H11.3	0	Hiposfagma	1
1	27/11/2019	H16.9	1	Queratitis	1
2	27/11/2019	H53.10	11	Problema refractivo	1
1	27/11/2019	H43.81	7	DVP	1
2	27/11/2019	H01.00	4	Blefaritis	1
2	27/11/2019	S05.9	9	Traumatismo	1
2	27/11/2019	T26	9	Causticación	1
2	27/11/2019	S05.9	9	Traumatismo	1
2	27/11/2019	H10.3	0	Conjuntivitis	1
1	28/11/2019	H16.9	1	Queratitis	1
1	28/11/2019	H15.1	6	Epiescleritis	1
2	28/11/2019	H10.3	0	Conjuntivitis	1
1	28/11/2019	H17.8	1	Infiltrados corneales	1
1	28/11/2019	H33.0	8	Desprendimiento de retina	1
2	28/11/2019	H11.3	0	Hiposfagma	1
2	28/11/2019	H10.3	0	Conjuntivitis	1
2	28/11/2019	S05.9	9	Traumatismo	1
2	28/11/2019	H01.00	4	Blefaritis	1
2	28/11/2019	H10.3	0	Conjuntivitis	1
2	28/11/2019	H43.81	7	DVP	1
2	28/11/2019	H43.81	7	DVP	1

1	28/11/2019	H11.3	0	Hiposfagma		1
2	28/11/2019	H10.3	0	Conjuntivitis		1
1	28/11/2019	H11.3	0	Hiposfagma		1
2	28/11/2019	H10.3	0	Conjuntivitis		1
2	28/11/2019	H35.3	8	Maculopatía		1
1	28/11/2019	H43.81	7	DVP		1
1	28/11/2019	T15.0	1	Cuerpo extraño		1
2	28/11/2019	G43.109	10	Aura		1
2	28/11/2019	H10.81	0	Pingueculitis		1
1	28/11/2019	T15.0	1	Cuerpo extraño		1
2	28/11/2019	H35.30	8	DMAE		1
2	28/11/2019	H16.9	1	Queratitis		1
2	28/11/2019	H01.00	4	Blefaritis		1
1	28/11/2019	H16.9	1	Queratitis		1
2	28/11/2019	H27.8	2	OCP		1
2	28/11/2019	H20	6	Uveitis		1
2	28/11/2019	H53	11	No patología		1
1	28/11/2019	H16.0	1	Úlcera corneal		1
1	28/11/2019	H43.81	7	DVP		1
1	28/11/2019	H27.8	2	OCP		1
1	28/11/2019	H10.3	0	Conjuntivitis		1
1	28/11/2019	H10.3	0	Conjuntivitis		1
2	28/11/2019	H16.9	1	Queratitis		1
1	28/11/2019	T15.0	1	Cuerpo extraño		1
2	28/11/2019	Alta por fuga	11	Alta por fuga		1
2	28/11/2019	H16.9	1	Queratitis		1
2	28/11/2019	H43.81	7	DVP		1
1	28/11/2019	S05.9	9	Traumatismo		1
1	28/11/2019	H20	6	Uveitis		1
1	28/11/2019	H20	6	Uveitis		1
2	28/11/2019	H00.02	4	Orzuelo		1
2	28/11/2019	H10.3	0	Conjuntivitis		1
1	28/11/2019	H10.3	0	Conjuntivitis		1
2	28/11/2019	H10.3	0	Conjuntivitis		1
2	29/11/2019	S05.9	9	Traumatismo		1
1	29/11/2019	H33.0	8	Desprendimiento de retina		1
1	29/11/2019	H10.3	0	Conjuntivitis		1
1	29/11/2019	S05.9	9	Traumatismo		1
1	29/11/2019	H02	4	Alteración palpebral		1
2	29/11/2019	H33.30	8	Desgarro retiniano		1
2	29/11/2019	H11.3	7	Hiposfagma		1
1	29/11/2019	H16.0	1	Úlcera corneal		1
1	29/11/2019	B00.1	4	Dermatitis herpética		1
2	29/11/2019	H15.00	6	Escleritis		1
2	29/11/2019	H43.1	7	Hemovítreo		1
2	29/11/2019	H53.9	11	Alteraciones visuales		1
2	29/11/2019	H10.3	0	Conjuntivitis		1
2	29/11/2019	H16.9	1	Queratitis		1
2	29/11/2019	H20	6	Uveitis		1
2	29/11/2019	H33.30	8	Desgarro retiniano		1
1	29/11/2019	H16.9	1	Queratitis		1
1	29/11/2019	H16.0	1	Úlcera corneal		1
2	29/11/2019	H01.00	4	Blefaritis		1
2	29/11/2019	H26.9	2	Catarata		1
2	29/11/2019	H10.3	0	Conjuntivitis		1
2	29/11/2019	H16.0	1	Úlcera corneal		1
1	29/11/2019	H35.30	8	DMAE		1
2	29/11/2019	H10.3	0	Conjuntivitis		1
2	29/11/2019	H34.9	8	Oclusión arterial		1
1	29/11/2019	H33.0	8	Desprendimiento de retina		1
1	29/11/2019	T15.0	1	Cuerpo extraño		1
2	29/11/2019	H33.30	8	Desgarro retiniano		1
1	29/11/2019	H01.00	4	Blefaritis		1
2	29/11/2019	H16.9	1	Queratitis		1
2	29/11/2019	H10.3	0	Conjuntivitis		1
2	29/11/2019	T15.0	1	Cuerpo extraño		1

2	29/11/2019	H0.41	1	Ojo seco	1
2	29/11/2019	H0.41	1	Ojo seco	1
2	29/11/2019	H00.02	4	Orzuelo	1
1	29/11/2019	T15.0	1	Cuerpo extraño	1
2	29/11/2019	H50.9	10	Estrabismo	1
2	29/11/2019	H00.02	4	Orzuelo	1
1	29/11/2019	S05.9	9	Traumatismo	1
1	29/11/2019	T15.0	1	Cuerpo extraño	1
1	29/11/2019	H04.32	5	Dacriocistitis aguda	1
1	29/11/2019	H43.81	7	DVP	1
2	29/11/2019	H04.32	5	Dacriocistitis aguda	1
1	29/11/2019	H01.00	4	Blefaritis	1
2	29/11/2019	T15.0	1	Cuerpo extraño	1
1	30/11/2019	T15.0	1	Cuerpo extraño	1
2	30/11/2019	H10.3	0	Conjuntivitis	1
1	30/11/2019	H10.3	0	Conjuntivitis	1
1	30/11/2019	H16.0	1	Úlcera corneal	1
2	30/11/2019	H10.3	0	Conjuntivitis	1
1	30/11/2019	H10.3	0	Conjuntivitis	1
1	30/11/2019	H16.0	1	Úlcera corneal	1
1	30/11/2019	H16.9	1	Queratitis	1
2	30/11/2019	H16.0	1	Úlcera corneal	1
2	30/11/2019	H43.81	7	DVP	1
2	30/11/2019	S05.9	9	Traumatismo	1
2	30/11/2019	H16.0	1	Úlcera corneal	1
1	30/11/2019	H10.3	0	Conjuntivitis	1
2	30/11/2019	H16.9	1	Queratitis	1
2	30/11/2019	H16.9	1	Queratitis	1
1	30/11/2019	H00.02	4	Orzuelo	1
1	30/11/2019	T15.0	1	Cuerpo extraño	1
1	30/11/2019	H26.9	2	Catarata	1
2	30/11/2019	H16.9	1	Queratitis	1
2	30/11/2019	H33.30	8	Desgarro retiniano	1
2	30/11/2019	T15.0	1	Cuerpo extraño	1
2	30/11/2019	H10.3	0	Conjuntivitis	1
2	30/11/2019	S05.9	9	Traumatismo	1
2	30/11/2019	G43.109	10	Aura	1
2	30/11/2019	H43.81	7	DVP	1
2	30/11/2019	H0.41	11	Ojo seco	1
1	30/11/2019	H16.0	1	Úlcera corneal	1
2	30/11/2019	H11.3	0	Hiposfagma	1
1	30/11/2019	H0.41	1	Ojo seco	1
1	30/11/2019	T15.0	1	Cuerpo extraño	1
1	30/11/2019	H00.02	4	Orzuelo	1
1	30/11/2019	H10.3	0	Conjuntivitis	1
2	30/11/2019	H16.9	1	Queratitis	1
2	30/11/2019	G43.109	10	Aura	1
1	30/11/2019	S05.9	9	Traumatismo	1
2	30/11/2019	H43.81	7	DVP	1
1	30/11/2019	H43.81	7	DVP	1
1	30/11/2019	H43.81	7	DVP	1
2	30/11/2019	H59.3	4	Post op párpados	1
1	30/11/2019	H10.5	4	Blefaroconjuntivitis	1
1	01/12/2019	H16.9	1	Queratitis	1
1	01/12/2019	H10.3	0	Conjuntivitis	1
2	01/12/2019	H01.00	0	Blefaritis	1
1	01/12/2019	T15.0	1	Cuerpo extraño	1
2	01/12/2019	H16.9	1	Queratitis	1
2	01/12/2019	B00.1	4	Dermatitis herpética	1
1	01/12/2019	T15.0	1	Cuerpo extraño	1
1	01/12/2019	H20	6	Uveitis	1
2	01/12/2019	H16.0	3	Atalamia	1
2	01/12/2019	H00.02	4	Orzuelo	1
2	01/12/2019	H16.9	1	Queratitis	1
2	01/12/2019	H43.81	7	DVP	1
2	01/12/2019	H26.9	2	Catarata	1

1	01/12/2019	H10.3	0	Conjuntivitis	1
1	01/12/2019	H16.0	1	Úlcera corneal	1
2	01/12/2019	H43.81	7	DVP	1
2	01/12/2019	H43.81	7	DVP	1
2	01/12/2019	H15.1	6	Epiescleritis	1
2	01/12/2019	H53.9	11	Alteraciones visuales	1
1	01/12/2019	B00.1	4	Dermatitis herpética	1
1	01/12/2019	H10.81	0	Pingueculitis	1
2	01/12/2019	H00.02	4	Orzuelo	1
1	01/12/2019	H16.0	1	Úlcera corneal	1
2	01/12/2019	H16.0	1	Úlcera corneal	1
2	01/12/2019	H20	6	Uveitis	1
1	01/12/2019	H16.9	1	Queratitis	1
2	01/12/2019	H16.9	1	Queratitis	1
2	01/12/2019	H16.9	1	Queratitis	1
2	01/12/2019	H01.00	4	Blefaritis	1
2	01/12/2019	H17.8	1	Infiltrados corneales	1
2	01/12/2019	H10.3	0	Conjuntivitis	1
2	01/12/2019	S05.30	9	Laceración conjuntival	1
2	01/12/2019	H11.82	0	Conjuntivocalasia	1
2	01/12/2019	H20	6	Uveitis	1
1	01/12/2019	H16.0	1	Úlcera corneal	1
1	01/12/2019	T15.0	1	Cuerpo extraño	1
1	01/12/2019	T15.0	1	Cuerpo extraño	1
1	01/12/2019	T15.0	1	Cuerpo extraño	1
2	01/12/2019	H10.3	0	Conjuntivitis	1
2	01/12/2019	H16.9	1	Queratitis	1
1	01/12/2019	T15.0	1	Cuerpo extraño	1
2	02/12/2019	H01.00	4	Blefaritis	1
2	02/12/2019	H16.0	1	Úlcera corneal	1
2	02/12/2019	H10.3	0	Conjuntivitis	1
2	02/12/2019	H0.41	1	Ojo seco	1
2	02/12/2019	H43.81	7	DVP	1
2	02/12/2019	H01.00	4	Blefaritis	1
1	02/12/2019	H20	6	Uveitis	1
2	02/12/2019	H01.00	4	Blefaritis	1
2	02/12/2019	H01.00	4	Blefaritis	1
2	02/12/2019	H43.81	7	DVP	1
2	02/12/2019	H01.00	4	Blefaritis	1
1	02/12/2019	Alta por fuga	11	Alta por fuga	1
2	02/12/2019	H10.5	4	Blefaroconjuntivitis	1
2	02/12/2019	H10.3	0	Conjuntivitis	1
2	02/12/2019	H01.00	4	Blefaritis	1
1	02/12/2019	H35.3	8	Maculopatía	1
2	02/12/2019	H01.00	4	Blefaritis	1
1	02/12/2019	H26.9	2	Catarata	1
2	02/12/2019	H35.3	8	Maculopatía	1
2	02/12/2019	H0.41	1	Ojo seco	1
2	02/12/2019	H16.9	1	Queratitis	1
2	02/12/2019	T15.0	1	Cuerpo extraño	1
2	02/12/2019	H16.9	1	Queratitis	1
1	02/12/2019	H40.05	3	HTO	1
1	02/12/2019	H16.0	1	Úlcera corneal	1
2	02/12/2019	H04.32	5	Dacriocistitis aguda	1
1	02/12/2019	H43.81	7	DVP	1
1	02/12/2019	H01.00	4	Blefaritis	1
2	02/12/2019	H04.32	5	Dacriocistitis aguda	1
1	02/12/2019	H33.0	8	Desprendimiento de retina	1
1	02/12/2019	H01.00	4	Blefaritis	1
2	02/12/2019	H16.9	1	Queratitis	1
1	02/12/2019	H26.9	2	Catarata	1
2	02/12/2019	H02.05	4	Distiquiasis	1
1	02/12/2019	H20	6	Uveitis	1
1	02/12/2019	T15.0	1	Cuerpo extraño	1
1	02/12/2019	G43.109	10	Aura	1
1	02/12/2019	T15.0	1	Cuerpo extraño	1

1	02/12/2019	H43.81	7	DVP	1
1	02/12/2019	H02	4	Lesión palpebral	1
2	02/12/2019	H10.81	6	Pingueculitis	1
1	03/12/2019	S05.9	9	Traumatismo	1
1	03/12/2019	H16.0	1	Úlcera corneal	1
1	03/12/2019	H10.3	0	Conjuntivitis	1
2	03/12/2019	H10.3	0	Conjuntivitis	1
2	03/12/2019	H20	6	Uveitis	1
2	03/12/2019	H00.02	4	Orzuelo	1
2	03/12/2019	H10.3	0	Conjuntivitis	1
1	03/12/2019	H16.9	1	Queratitis	1
2	03/12/2019	H10.3	0	Conjuntivitis	1
2	03/12/2019	H11.3	0	Hiposfagma	1
2	03/12/2019	H00.02	4	Orzuelo	1
2	03/12/2019	H00.02	4	Orzuelo	1
1	03/12/2019	H10.3	0	Conjuntivitis	1
2	03/12/2019	H18.20	1	Edema corneal	1
2	03/12/2019	H35.30	8	DMAE	1
2	03/12/2019	H33.0	8	Desprendimiento de retina	1
2	03/12/2019	H16.9	1	Queratitis	1
1	03/12/2019	H33.30	8	Desgarro retiniano	1
2	03/12/2019	H11.3	0	Hiposfagma	1
2	03/12/2019	H0.41	1	Ojo seco	1
2	03/12/2019	H33.30	8	Desgarro retiniano	1
1	03/12/2019	S05.9	9	Traumatismo	1
1	03/12/2019	H35.30	8	DMAE	1
1	03/12/2019	H10.3	0	Conjuntivitis	1
1	03/12/2019	H43.81	7	DVP	1
2	03/12/2019	H10.3	0	Conjuntivitis	1
1	03/12/2019	H16.0	1	Úlcera corneal	1
1	03/12/2019	E11.319	8	Retinopatía diabética	1
2	03/12/2019	H43.81	7	DVP	1
2	03/12/2019	H50.9	10	Estrabismo	1
2	03/12/2019	H0.41	1	Ojo seco	1
1	03/12/2019	T15.0	1	Cuerpo extraño	1
2	03/12/2019	H10.3	0	Conjuntivitis	1
2	03/12/2019	T15.0	1	Cuerpo extraño	1
1	03/12/2019	H53.10	11	Problema refractivo	1
1	03/12/2019	S05.9	9	Traumatismo	1
1	03/12/2019	H10.3	0	Conjuntivitis	1
1	03/12/2019	H11.3	0	Hiposfagma	1
2	03/12/2019	H43.81	7	DVP	1
2	04/12/2019	H43.1	7	Hemovítreo	1
2	04/12/2019	H35.30	4	DMAE	1
2	04/12/2019	T15.0	1	Cuerpo extraño	1
2	04/12/2019	H00.02	4	Orzuelo	1
1	04/12/2019	H10.3	0	Conjuntivitis	1
2	04/12/2019	H16.9	1	Queratitis	1
1	04/12/2019	S05.30	9	Laceración conjuntival	1
2	04/12/2019	H16.9	1	Queratitis	1
1	04/12/2019	H10.5	4	Blefaroconjuntivitis	1
2	04/12/2019	H0.41	1	Ojo seco	1
2	04/12/2019	H10.3	0	Conjuntivitis	1
2	04/12/2019	H00.02	4	Orzuelo	1
2	04/12/2019	H0.41	1	Ojo seco	1
1	04/12/2019	H17.8	1	Infiltrados corneales	1
2	04/12/2019	H10.3	0	Conjuntivitis	1
1	04/12/2019	H10.3	0	Conjuntivitis	1
2	04/12/2019	H16.9	1	Queratitis	1
1	04/12/2019	H0.41	1	Ojo seco	1
2	04/12/2019	H01.00	4	Blefaritis	1
2	04/12/2019	H43.81	7	DVP	1
1	04/12/2019	H53	11	No patología	1
2	04/12/2019	H10.5	0	Blefaroconjuntivitis	1
2	04/12/2019	H01.00	4	Blefaritis	1
2	04/12/2019	H11.3	0	Hiposfagma	1

2	04/12/2019	H16.9	1	Queratitis	1
2	04/12/2019	H11.3	0	Hiposfagma	1
2	04/12/2019	H20	6	Uveitis	1
1	04/12/2019	H10.3	0	Conjuntivitis	1
2	04/12/2019	H10.3	0	Conjuntivitis	1
1	04/12/2019	H11.3	0	Hiposfagma	1
2	04/12/2019	S05.9	9	Traumatismo	1
1	04/12/2019	H27.1	2	Luxación catarata	1
2	04/12/2019	H59.3	5	Postop via lagrimal	1
2	04/12/2019	H10.81	0	Pingueculitis	1
1	04/12/2019	H16.9	1	Queratitis	1
1	04/12/2019	H43.81	7	DVP	1
1	04/12/2019	H10.3	0	Conjuntivitis	1
2	04/12/2019	H16.9	1	Queratitis	1
1	04/12/2019	H00.02	4	Orzuelo	1
2	04/12/2019	H43.81	7	DVP	1
2	04/12/2019	H31.42	8	Coroidopatía serosa central	1
1	04/12/2019	H20	6	Uveitis	1
1	04/12/2019	S05.9	9	Traumatismo	1
1	04/12/2019	H43.1	7	Hemovítreo	1
1	04/12/2019	H01.00	0	Blefaritis	1
1	04/12/2019	H20	6	Uveitis	1
2	04/12/2019	H34.82	8	Trombosis venosa	1
2	04/12/2019	H10.3	0	Conjuntivitis	1
2	04/12/2019	H16.9	1	Queratitis	1
2	04/12/2019	H16.9	1	Queratitis	1
2	04/12/2019	H16.9	1	Queratitis	1
2	04/12/2019	H00.02	4	Orzuelo	1
2	04/12/2019	H20	6	Uveitis	1
1	04/12/2019	T15.0	1	Cuerpo extraño	1
1	04/12/2019	H02	4	Lesión palpebral	1
1	04/12/2019	T15.0	1	Cuerpo extraño	1
1	04/12/2019	H33.30	8	Desgarro retiniano	1
2	04/12/2019	S05.9	9	Traumatismo	1
2	04/12/2019	H10.3	0	Conjuntivitis	1
1	04/12/2019	H0.41	1	Ojo seco	1
2	04/12/2019	H16.9	1	Queratitis	1
2	04/12/2019	H16.9	1	Queratitis	1
1	04/12/2019	T15.0	1	Cuerpo extraño	1
1	04/12/2019	H16.0	3	Atalamia	1
1	04/12/2019	H16.0	1	Úlcera corneal	1
1	04/12/2019	H16.9	1	Queratitis	1
1	05/12/2019	H10.5	4	Blefaroconjuntivitis	1
2	05/12/2019	H10.3	0	Conjuntivitis	1
1	05/12/2019	H15.1	6	Epiescleritis	1
2	05/12/2019	H10.5	4	Blefaroconjuntivitis	1
2	05/12/2019	H10.3	0	Conjuntivitis	1
1	05/12/2019	T15.0	1	Cuerpo extraño	1
2	05/12/2019	H20	6	Uveitis	1
2	05/12/2019	H16.9	1	Queratitis	1
1	05/12/2019	H10.3	0	Conjuntivitis	1
2	05/12/2019	H43.81	7	DVP	1
1	05/12/2019	H17.8	1	Infiltrados corneales	1
2	05/12/2019	H0.41	1	Ojo seco	1
2	05/12/2019	H10.3	0	Conjuntivitis	1
1	05/12/2019	T15.0	1	Cuerpo extraño	1
2	05/12/2019	H10.5	4	Blefaroconjuntivitis	1
2	05/12/2019	H10.3	0	Conjuntivitis	1
2	05/12/2019	H0.41	1	Ojo seco	1
2	05/12/2019	H16.0	1	Úlcera corneal	1
2	05/12/2019	H53.9	11	Alteraciones visuales	1
2	05/12/2019	H43.81	7	DVP	1
1	05/12/2019	H10.3	0	Conjuntivitis	1
2	05/12/2019	H01.00	4	Blefaritis	1
1	05/12/2019	H47.10	10	Papiledema	1
1	05/12/2019	H53.9	11	Alteraciones visuales	1

1	05/12/2019	H10.3	0	Conjuntivitis	1
2	05/12/2019	H43.81	7	DVP	1
1	05/12/2019	H10.3	0	Conjuntivitis	1
1	05/12/2019	H16.0	1	Úlcera corneal	1
2	05/12/2019	H53	11	No patología	1
2	05/12/2019	H16.0	1	Úlcera corneal	1
2	05/12/2019	H00.02	2	Orzuelo	1
2	05/12/2019	H10.3	0	Conjuntivitis	1
1	05/12/2019	H33.30	8	Desgarro retiniano	1
1	05/12/2019	T15.0	1	Cuerpo extraño	1
2	05/12/2019	S05.9	9	Traumatismo	1
2	05/12/2019	H10.3	0	Conjuntivitis	1
2	05/12/2019	S05.00	9	Abrasión conjuntival	1
2	05/12/2019	S05.9	9	Traumatismo	1
2	05/12/2019	H10.3	0	Conjuntivitis	1
1	05/12/2019	H16.9	1	Queratitis	1
1	05/12/2019	Alta por fuga	11	Alta por fuga	1
2	05/12/2019	H10.3	0	Conjuntivitis	1
1	05/12/2019	H10.3	0	Conjuntivitis	1
2	05/12/2019	T15.0	1	Cuerpo extraño	1
2	06/12/2019	H16.9	1	Queratitis	1
2	06/12/2019	T15.0	1	Cuerpo extraño	1
1	06/12/2019	S05.9	9	Traumatismo	1
2	06/12/2019	H18.20	1	Edema corneal	1
1	06/12/2019	H16.0	1	Úlcera corneal	1
1	06/12/2019	H10.3	0	Conjuntivitis	1
2	06/12/2019	H49.9	10	Parálisis oculomotora	1
2	06/12/2019	H0.41	1	Ojo seco	1
2	06/12/2019	H16.0	1	Úlcera corneal	1
1	06/12/2019	H20	6	Uveitis	1
1	06/12/2019	H16.0	1	Úlcera corneal	1
2	06/12/2019	B00.1	4	Dermatitis herpética	1
1	06/12/2019	H16.9	1	Queratitis	1
1	06/12/2019	H16.0	1	Úlcera corneal	1
2	06/12/2019	H10.3	0	Conjuntivitis	1
1	06/12/2019	S05.9	9	Traumatismo	1
2	06/12/2019	H16.9	1	Queratitis	1
1	06/12/2019	H11.00	0	Pterigium	1
1	06/12/2019	H53.9	11	Alteraciones visuales	1
2	06/12/2019	S05.9	9	Traumatismo	1
2	06/12/2019	H10.3	0	Conjuntivitis	1
2	06/12/2019	H11.3	0	Hiposfagma	1
2	06/12/2019	S05.9	9	Traumatismo	1
1	06/12/2019	H40.5	3	Glaucoma secundario	1
1	06/12/2019	H10.3	0	Conjuntivitis	1
2	06/12/2019	H53.9	11	Alteraciones visuales	1
1	06/12/2019	H59.88	8	Post op retina	1
2	06/12/2019	H16.9	1	Queratitis	1
1	06/12/2019	T15.0	1	Cuerpo extraño	1
2	06/12/2019	H16.9	1	Queratitis	1
1	06/12/2019	H20	6	Uveitis	1
2	06/12/2019	H43.81	7	DVP	1
1	06/12/2019	H49.9	10	Parálisis oculomotora	1
1	06/12/2019	S05.9	9	Traumatismo	1
2	06/12/2019	H10.3	0	Conjuntivitis	1
1	06/12/2019	H00.02	4	Orzuelo	1
2	06/12/2019	H02	4	Lesión palpebral	1
2	06/12/2019	H16.0	1	Úlcera corneal	1
1	06/12/2019	H20	6	Uveitis	1
2	06/12/2019	H20	6	Uveitis	1
1	06/12/2019	H16.9	1	Queratitis	1
2	06/12/2019	H16.9	1	Queratitis	1
2	06/12/2019	H10.3	0	Conjuntivitis	1
2	06/12/2019	H10.3	0	Conjuntivitis	1
1	06/12/2019	H20	6	Uveitis	1
2	06/12/2019	H43.81	7	DVP	1

2	06/12/2019	H20	6	Uveitis	1
1	06/12/2019	H10.3	0	Conjuntivitis	1
2	06/12/2019	H43.81	7	DVP	1
1	06/12/2019	H10.3	0	Conjuntivitis	1
1	06/12/2019	T15.0	1	Cuerpo extraño	1
2	06/12/2019	H10.3	0	Conjuntivitis	1
1	06/12/2019	T15.0	1	Cuerpo extraño	1
2	06/12/2019	H16.0	1	Úlcera corneal	1
1	07/12/2019	H16.9	1	Queratitis	1
1	07/12/2019	T15.0	1	Cuerpo extraño	1
1	07/12/2019	H00.02	4	Orzuelo	1
1	07/12/2019	H16.0	1	Úlcera corneal	1
1	07/12/2019	H17.8	1	Infiltrados corneales	1
1	07/12/2019	S05.00	9	Abrasión conjuntival	1
2	07/12/2019	H01.00	4	Blefaritis	1
1	07/12/2019	H10.3	0	Conjuntivitis	1
2	07/12/2019	H11.3	0	Hiposfagma	1
1	07/12/2019	H10.3	0	Conjuntivitis	1
2	07/12/2019	H17.8	1	Infiltrados corneales	1
1	07/12/2019	H16.0	1	Úlcera corneal	1
1	07/12/2019	H00.02	4	Orzuelo	1
2	07/12/2019	H43.81	7	DVP	1
1	07/12/2019	H33.0	8	Desprendimiento de retina	1
2	07/12/2019	H01.00	4	Blefaritis	1
2	07/12/2019	H00.02	4	Orzuelo	1
2	07/12/2019	H43.81	7	DVP	1
2	07/12/2019	H16.9	1	Queratitis	1
2	07/12/2019	H10.3	0	Conjuntivitis	1
1	07/12/2019	H10.3	0	Conjuntivitis	1
2	07/12/2019	H10.3	0	Conjuntivitis	1
1	07/12/2019	T15.0	1	Cuerpo extraño	1
2	07/12/2019	H00.02	4	Orzuelo	1
1	07/12/2019	H00.03	4	Celulitis preseptal	1
2	07/12/2019	H10.3	0	Conjuntivitis	1
1	07/12/2019	H16.0	1	Úlcera corneal	1
1	07/12/2019	H10.3	0	Conjuntivitis	1
1	07/12/2019	S05.30	9	Laceración conjuntival	1
1	07/12/2019	H16.9	1	Queratitis	1
1	07/12/2019	H01.00	4	Blefaritis	1
1	07/12/2019	H15.00	6	Escleritis	1
1	07/12/2019	H00.02	4	Orzuelo	1
2	07/12/2019	H16.0	1	Úlcera corneal	1
1	07/12/2019	H16.0	1	Úlcera corneal	1
2	07/12/2019	H16.9	1	Queratitis	1
1	07/12/2019	T15.0	1	Cuerpo extraño	1
1	07/12/2019	Alta por fuga	11	Alta por fuga	1
1	07/12/2019	H15.00	6	Escleritis	1
2	07/12/2019	H43.81	7	DVP	1
1	07/12/2019	H11.3	0	Hiposfagma	1
2	08/12/2019	H40.05	3	HTO	1
2	08/12/2019	H02.05	4	Distiquiasis	1
1	08/12/2019	H16.0	1	Úlcera corneal	1
1	08/12/2019	H10.3	0	Conjuntivitis	1
1	08/12/2019	T15.0	1	Cuerpo extraño	1
2	08/12/2019	H00.19	4	Chalazión	1
2	08/12/2019	H01.00	4	Blefaritis	1
2	08/12/2019	H00.03	4	Celulitis preseptal	1
1	08/12/2019	H20	6	Uveitis	1
2	08/12/2019	H00.02	4	Orzuelo	1
1	08/12/2019	H35.30	8	DMAE	1
1	08/12/2019	T26	9	Causticación	1
2	08/12/2019	H00.02	4	Orzuelo	1
1	08/12/2019	H02.05	4	Distiquiasis	1
2	08/12/2019	H16.9	1	Queratitis	1
2	08/12/2019	H10.3	0	Conjuntivitis	1
2	08/12/2019	G43.9	10	Migraña	1

2	08/12/2019	H16.0	1	Úlcera corneal	1
1	08/12/2019	H10.3	0	Conjuntivitis	1
1	08/12/2019	H35.30	8	DMAE	1
1	08/12/2019	H10.3	0	Conjuntivitis	1
2	08/12/2019	S05.9	9	Traumatismo	1
2	08/12/2019	H10.3	0	Conjuntivitis	1
2	08/12/2019	H16.9	1	Queratitis	1
1	08/12/2019	H10.3	0	Conjuntivitis	1
2	08/12/2019	H35.3	8	Maculopatía	1
2	08/12/2019	H20	6	Uveitis	1
1	08/12/2019	H0.41	1	Ojo seco	1
2	08/12/2019	H16.9	1	Queratitis	1
2	08/12/2019	H02	4	Lesión palpebral	1
1	09/12/2019	S05.00	9	Abrasión conjuntival	1
1	09/12/2019	H10.3	0	Conjuntivitis	1
1	09/12/2019	H20	6	Uveitis	1
1	09/12/2019	H10.3	0	Conjuntivitis	1
1	09/12/2019	T15.0	1	Cuerpo extraño	1
1	09/12/2019	H10.3	0	Conjuntivitis	1
2	09/12/2019	H10.3	0	Conjuntivitis	1
1	09/12/2019	H10.5	4	Blefaroconjuntivitis	1
2	09/12/2019	H04.32	5	Dacriocistitis aguda	1
2	09/12/2019	H04.32	5	Dacriocistitis aguda	1
2	09/12/2019	H20	6	Uveitis	1
2	09/12/2019	E11.319	8	Retinopatía diabética	1
1	09/12/2019	H20	6	Uveitis	1
1	09/12/2019	H18.20	1	Edema corneal	1
1	09/12/2019	H11.3	0	Hiposfagma	1
2	09/12/2019	H00.02	4	Orzuelo	1
1	09/12/2019	H43.81	7	DVP	1
2	09/12/2019	H0.41	1	Ojo seco	1
2	09/12/2019	H01.00	4	Blefaritis	1
2	09/12/2019	H10.3	0	Conjuntivitis	1
2	09/12/2019	H10.5	4	Blefaroconjuntivitis	1
2	09/12/2019	H10.3	0	Conjuntivitis	1
2	09/12/2019	H16.0	1	Úlcera corneal	1
2	09/12/2019	H11.00	0	Pterigium	1
1	09/12/2019	H16.9	1	Queratitis	1
2	09/12/2019	H04.32	5	Dacriocistitis aguda	1
2	09/12/2019	H10.3	0	Conjuntivitis	1
1	09/12/2019	Trocleítis	4	Trocleítis	1
2	09/12/2019	H10.3	0	Conjuntivitis	1
2	09/12/2019	H02	4	Lesión palpebral	1
2	09/12/2019	T15.0	1	Cuerpo extraño	1
2	09/12/2019	H26.9	2	Catarata	1
1	09/12/2019	S05.9	9	Traumatismo	1
1	09/12/2019	H11.3	0	Hiposfagma	1
1	09/12/2019	H16.0	1	Úlcera corneal	1
2	09/12/2019	H10.3	0	Conjuntivitis	1
1	09/12/2019	T15.0	1	Cuerpo extraño	1
1	09/12/2019	T15.0	1	Cuerpo extraño	1
2	09/12/2019	H16.9	1	Queratitis	1
1	09/12/2019	H10.3	0	Conjuntivitis	1
2	09/12/2019	T15.0	1	Cuerpo extraño	1
2	09/12/2019	S05.9	9	Traumatismo	1
1	10/12/2019	T15.0	1	Cuerpo extraño	1
2	10/12/2019	H0.41	1	Ojo seco	1
1	10/12/2019	H00.02	4	Orzuelo	1
2	10/12/2019	H27.8	2	OCP	1
2	10/12/2019	H0.41	1	Ojo seco	1
2	10/12/2019	H00.02	4	Orzuelo	1
1	10/12/2019	H59.88	8	Post op retina	1
1	10/12/2019	H43.1	7	Hemovítreo	1
1	10/12/2019	H16.9	1	Queratitis	1
2	10/12/2019	H02.05	4	Distiquiasis	1
1	10/12/2019	H59.88	8	Post op retina	1

2	10/12/2019	H10.5	4	Blefaroconjuntivitis	1
2	10/12/2019	H17.8	1	Infiltrados corneales	1
1	10/12/2019	H43.1	7	Hemovítreo	1
1	10/12/2019	H43.1	7	Hemovítreo	1
1	10/12/2019	H10.3	0	Conjuntivitis	1
2	10/12/2019	H16.9	1	Queratitis	1
2	10/12/2019	H16.9	1	Queratitis	1
2	10/12/2019	H10.3	0	Conjuntivitis	1
2	10/12/2019	H33.0	8	Desprendimiento de retina	1
2	10/12/2019	H43.81	7	DVP	1
2	10/12/2019	H10.3	0	Conjuntivitis	1
1	10/12/2019	H10.81	0	Pingueculitis	1
1	10/12/2019	H43.81	7	DVP	1
2	10/12/2019	T15.0	1	Cuerpo extraño	1
2	10/12/2019	H00.02	4	Orzuelo	1
2	10/12/2019	H46	10	Neuritis óptica	1
2	10/12/2019	H53.9	11	Alteraciones visuales	1
2	10/12/2019	H10.3	1	Conjuntivitis	1
2	10/12/2019	H00.02	4	Orzuelo	1
2	10/12/2019	H10.3	0	Conjuntivitis	1
1	10/12/2019	H33.0	8	Desprendimiento de retina	1
2	10/12/2019	H10.3	0	Conjuntivitis	1
2	10/12/2019	H20	6	Uveitis	1
1	10/12/2019	H00.19	4	Chalazión	1
1	10/12/2019	H16.0	1	Úlcera corneal	1
1	10/12/2019	H11.3	0	Hiposfagma	1
1	10/12/2019	H02	4	Alteración palpebral	1
2	10/12/2019	S05.30	9	Laceración conjuntival	1
1	11/12/2019	S05.6	9	Perforación ocular	1
1	11/12/2019	H16.9	1	Queratitis	1
2	11/12/2019	H20	6	Uveitis	1
1	11/12/2019	H10.3	0	Conjuntivitis	1
2	11/12/2019	H10.3	0	Conjuntivitis	1
2	11/12/2019	H16.9	1	Queratitis	1
2	11/12/2019	H01.00	4	Blefaritis	1
1	11/12/2019	H01.00	4	Blefaritis	1
1	11/12/2019	H10.3	0	Conjuntivitis	1
1	11/12/2019	H16.9	1	Queratitis	1
1	11/12/2019	H01.00	4	Blefaritis	1
1	11/12/2019	S05.30	9	Laceración conjuntival	1
1	11/12/2019	H01.00	4	Blefaritis	1
2	11/12/2019	H16.9	1	Queratitis	1
2	11/12/2019	H01.00	4	Blefaritis	1
2	11/12/2019	H11.3	0	Hiposfagma	1
1	11/12/2019	T15.0	1	Cuerpo extraño	1
1	11/12/2019	H16.0	1	Úlcera corneal	1
1	11/12/2019	H11.3	0	Hiposfagma	1
2	11/12/2019	H16.9	1	Queratitis	1
2	11/12/2019	H35.30	8	DMAE	1
2	11/12/2019	H53	11	No patología	1
2	11/12/2019	H11.3	0	Hiposfagma	1
2	11/12/2019	H40.05	3	HTO	1
2	11/12/2019	H16.9	1	Queratitis	1
2	11/12/2019	H02	4	Alteración palpebral	1
2	11/12/2019	H10.3	0	Conjuntivitis	1
2	11/12/2019	H00.02	4	Orzuelo	1
1	11/12/2019	H16.0	1	Úlcera corneal	1
2	11/12/2019	H10.3	0	Conjuntivitis	1
2	11/12/2019	H10.3	0	Conjuntivitis	1
2	11/12/2019	H00.02	4	Orzuelo	1
2	11/12/2019	H10.81	0	Pingueculitis	1
1	11/12/2019	H43.81	7	DVP	1
1	11/12/2019	H59.3	4	Post op párpados	1
2	11/12/2019	H02.05	4	Distiquiasis	1
1	11/12/2019	H11.3	0	Hiposfagma	1
1	11/12/2019	H27.8	2	OCP	1

2	11/12/2019	H0.41	1	Ojo seco	1
1	11/12/2019	H16.0	1	Úlcera corneal	1
2	11/12/2019	H10.3	0	Conjuntivitis	1
2	11/12/2019	H10.5	4	Blefaroconjuntivitis	1
2	11/12/2019	H43.81	7	DVP	1
2	11/12/2019	H16.0	1	Úlcera corneal	1
1	11/12/2019	H10.3	0	Conjuntivitis	1
1	11/12/2019	T15.0	1	Cuerpo extraño	1
2	11/12/2019	S05.9	9	Traumatismo	1
1	11/12/2019	H43.1	7	Hemovítreo	1
2	11/12/2019	B00.1	4	Dermatitis herpética	1
2	11/12/2019	B00.1	4	Dermatitis herpética	1
1	11/12/2019	T15.0	1	Cuerpo extraño	1
2	11/12/2019	Alta por fuga	11	Alta por fuga	1
2	11/12/2019	H16.9	1	Queratitis	1
2	11/12/2019	H53.9	11	Alteraciones visuales	1
1	11/12/2019	S05.9	9	Traumatismo	1
1	11/12/2019	H10.3	0	Conjuntivitis	1
1	11/12/2019	H11.3	0	Hiposfagma	1
2	11/12/2019	H10.3	0	Conjuntivitis	1
1	11/12/2019	S05.9	9	Traumatismo	1
1	11/12/2019	H00.02	4	Orzuelo	1
1	12/12/2019	H16.9	1	Queratitis	1
1	12/12/2019	H16.0	1	Úlcera corneal	1
1	12/12/2019	H16.9	1	Queratitis	1
1	12/12/2019	H16.9	1	Queratitis	1
2	12/12/2019	H20	6	Uveitis	1
2	12/12/2019	H53.9	11	Alteraciones visuales	1
2	12/12/2019	H16.0	1	Úlcera corneal	1
2	12/12/2019	H16.9	1	Queratitis	1
1	12/12/2019	H01.00	4	Blefaritis	1
2	12/12/2019	H16.9	1	Queratitis	1
1	12/12/2019	H10.3	0	Conjuntivitis	1
1	12/12/2019	H16.0	1	Úlcera corneal	1
2	12/12/2019	H16.9	1	Queratitis	1
1	12/12/2019	H43.1	7	Hemovítreo	1
2	12/12/2019	H53.9	11	Alteraciones visuales	1
2	12/12/2019	H10.3	0	Conjuntivitis	1
1	12/12/2019	H35.30	8	DMAE	1
1	12/12/2019	H35.3	8	Maculopatía	1
2	12/12/2019	H04.32	5	Dacriocistitis aguda	1
2	12/12/2019	H35.3	8	Maculopatía	1
2	12/12/2019	H00.02	4	Orzuelo	1
1	12/12/2019	H16.9	1	Queratitis	1
2	12/12/2019	H10.3	0	Conjuntivitis	1
1	12/12/2019	S05.9	9	Traumatismo	1
1	12/12/2019	H02.05	4	Distiquiasis	1
1	12/12/2019	H10.3	0	Conjuntivitis	1
1	12/12/2019	H16.9	1	Queratitis	1
1	12/12/2019	H33.30	8	Desgarro retiniano	1
1	12/12/2019	H02	4	Lesión palpebral	1
1	12/12/2019	H0.41	1	Ojo seco	1
2	12/12/2019	H04.32	5	Dacriocistitis aguda	1
2	12/12/2019	G43.109	10	Aura	1
1	12/12/2019	H10.3	0	Conjuntivitis	1
1	12/12/2019	H16.0	1	Úlcera corneal	1
2	12/12/2019	Alta por fuga	11	Alta por fuga	1
1	12/12/2019	T15.0	1	Cuerpo extraño	1
1	12/12/2019	H10.3	0	Conjuntivitis	1
1	12/12/2019	H10.3	0	Conjuntivitis	1
1	13/12/2019	G43.9	10	Migraña	1
1	13/12/2019	H16.9	1	Queratitis	1
2	13/12/2019	H00.02	4	Orzuelo	1
2	13/12/2019	H16.0	1	Úlcera corneal	1
2	13/12/2019	H00.03	4	Celulitis preseptal	1
1	13/12/2019	H40.9	3	Glaucoma	1

2	13/12/2019	H16.9	1	Queratitis	1
2	13/12/2019	H43.81	7	DVP	1
1	13/12/2019	H02.05	4	Distiquiasis	1
2	13/12/2019	H00.02	4	Orzuelo	1
2	13/12/2019	H10.5	4	Blefaroconjuntivitis	1
2	13/12/2019	H02.05	4	Distiquiasis	1
1	13/12/2019	H43.1	7	Hemovítreo	1
1	13/12/2019	H16.0	1	Úlcera corneal	1
2	13/12/2019	H17.8	1	Infiltrados corneales	1
1	13/12/2019	H04.32	5	Dacriocistitis aguda	1
1	13/12/2019	H10.3	0	Conjuntivitis	1
2	13/12/2019	H16.9	1	Queratitis	1
2	13/12/2019	H31.42	8	Coroidopatía serosa central	1
2	13/12/2019	H16.9	1	Queratitis	1
1	13/12/2019	H34.9	8	Oclusión arterial	1
2	13/12/2019	H16.9	1	Queratitis	1
2	13/12/2019	H16.9	1	Queratitis	1
2	13/12/2019	H01.00	4	Blefaritis	1
2	13/12/2019	H43.81	7	DVP	1
2	13/12/2019	H20	6	Uveitis	1
2	13/12/2019	H18.20	1	Edema corneal	1
1	13/12/2019	H53.10	11	Problema refractivo	1
1	13/12/2019	H16.0	1	Úlcera corneal	1
1	13/12/2019	H16.9	1	Queratitis	1
2	13/12/2019	H16.9	1	Queratitis	1
1	13/12/2019	H34.82	8	Trombosis venosa	1
1	13/12/2019	S05.9	9	Traumatismo	1
2	13/12/2019	Trocleítis	4	Trocleítis	1
1	13/12/2019	H10.5	4	Blefaroconjuntivitis	1
1	13/12/2019	S05.30	9	Laceración conjuntival	1
1	13/12/2019	B00.1	4	Dermatitis herpética	1
1	13/12/2019	H43.81	7	DVP	1
1	13/12/2019	T15.0	1	Cuerpo extraño	1
2	13/12/2019	H50.9	10	Estrabismo	1
1	13/12/2019	T15.0	1	Cuerpo extraño	1
1	13/12/2019	T15.0	1	Cuerpo extraño	1
2	13/12/2019	T15.0	1	Cuerpo extraño	1
1	13/12/2019	H11.3	0	Hiposfagma	1
1	13/12/2019	H16.9	1	Queratitis	1
2	13/12/2019	T15.0	1	Cuerpo extraño	1
1	13/12/2019	T15.0	1	Cuerpo extraño	1
1	13/12/2019	H11.44	0	Quiste conjuntival	1
1	13/12/2019	H10.3	0	Conjuntivitis	1
1	13/12/2019	S05.9	9	Traumatismo	1
2	13/12/2019	T15.0	1	Cuerpo extraño	1
1	14/12/2019	T15.0	1	Cuerpo extraño	1
1	14/12/2019	S05.9	9	Traumatismo	1
1	14/12/2019	T15.0	1	Cuerpo extraño	1
2	14/12/2019	H01.00	4	Blefaritis	1
2	14/12/2019	S05.9	9	Traumatismo	1
1	14/12/2019	H16.0	1	Úlcera corneal	1
1	14/12/2019	H40.9	3	Glaucoma	1
1	14/12/2019	S05.9	9	Traumatismo	1
1	14/12/2019	H00.03	4	Celulitis preseptal	1
2	14/12/2019	H00.19	4	Chalazión	1
1	14/12/2019	H43.81	7	DVP	1
2	14/12/2019	H20	6	Uveitis	1
2	14/12/2019	H53	11	No patología	1
2	14/12/2019	H0.41	1	Ojo seco	1
2	14/12/2019	H16.0	1	Úlcera corneal	1
1	14/12/2019	H16.0	1	Úlcera corneal	1
2	14/12/2019	S05.00	9	Abrasión conjuntival	1
1	14/12/2019	H02	4	Lesión palpebral	1
1	14/12/2019	S05.9	9	Traumatismo	1
1	14/12/2019	H17.8	1	Infiltrados corneales	1
2	14/12/2019	H53	11	No patología	1

2	14/12/2019	H16.0	1	Úlcera corneal	1
1	14/12/2019	H10.3	0	Conjuntivitis	1
2	14/12/2019	H16.0	1	Úlcera corneal	1
1	14/12/2019	H10.3	0	Conjuntivitis	1
2	14/12/2019	H16.9	1	Queratitis	1
2	14/12/2019	H11.3	0	Hiposfagma	1
2	14/12/2019	H16.9	1	Queratitis	1
1	14/12/2019	T15.0	1	Cuerpo extraño	1
1	14/12/2019	H00.02	4	Orzuelo	1
2	14/12/2019	H16.0	1	Úlcera corneal	1
2	14/12/2019	T15.0	1	Cuerpo extraño	1
2	14/12/2019	H27.8	2	OCP	1
1	14/12/2019	H33.30	8	Desgarro retiniano	1
2	14/12/2019	H33.30	8	Desgarro retiniano	1
2	14/12/2019	H16.9	1	Queratitis	1
2	14/12/2019	H18.50	1	Distrofia corneal	1
2	14/12/2019	H43.1	7	Hemovítreo	1
2	14/12/2019	H16.9	1	Queratitis	1
2	14/12/2019	H17.8	1	Infiltrados corneales	1
1	14/12/2019	H10.3	0	Conjuntivitis	1
2	14/12/2019	H15.1	6	Epiescleritis	1
2	14/12/2019	H40.9	3	Glaucoma	1
2	14/12/2019	H53.9	11	Alteraciones visuales	1
1	14/12/2019	S05.9	9	Traumatismo	1
1	14/12/2019	H16.9	1	Queratitis	1
2	14/12/2019	H20	6	Uveitis	1
1	14/12/2019	S05.9	9	Traumatismo	1
1	14/12/2019	S05.9	9	Traumatismo	1
1	14/12/2019	H16.0	1	Úlcera corneal	1
2	14/12/2019	H10.3	0	Conjuntivitis	1
1	15/12/2019	H11.3	0	Hiposfagma	1
1	15/12/2019	H16.0	1	Úlcera corneal	1
2	15/12/2019	H10.5	4	Blefaroconjuntivitis	1
1	15/12/2019	H16.0	1	Úlcera corneal	1
2	15/12/2019	H16.0	1	Úlcera corneal	1
1	15/12/2019	H11.3	0	Hiposfagma	1
1	15/12/2019	H01.00	4	Blefaritis	1
1	15/12/2019	T15.0	1	Cuerpo extraño	1
2	15/12/2019	H00.02	4	Orzuelo	1
2	15/12/2019	H02	4	Lesión palpebral	1
1	15/12/2019	T15.0	1	Cuerpo extraño	1
2	15/12/2019	H11.3	0	Hiposfagma	1
2	15/12/2019	H0.41	1	Ojo seco	1
2	15/12/2019	H10.3	0	Conjuntivitis	1
1	15/12/2019	H10.3	0	Conjuntivitis	1
2	15/12/2019	H59.3	4	Post op párpados	1
1	15/12/2019	H02	4	Alteración palpebral	1
1	15/12/2019	H16.0	1	Úlcera corneal	1
1	15/12/2019	H20	6	Uveitis	1
2	15/12/2019	H43.81	7	DVP	1
2	15/12/2019	H0.41	1	Ojo seco	1
1	15/12/2019	H11.3	0	Hiposfagma	1
1	15/12/2019	H34.82	8	Trombosis venosa	1
2	15/12/2019	H11.82	0	Conjuntivocalasia	1
2	15/12/2019	H16.9	1	Queratitis	1
2	15/12/2019	H16.9	1	Queratitis	1
2	15/12/2019	H43.1	7	Hemovítreo	1
2	15/12/2019	H16.9	1	Queratitis	1
2	15/12/2019	H16.9	1	Queratitis	1
1	15/12/2019	H10.3	0	Conjuntivitis	1
1	15/12/2019	H0.41	1	Ojo seco	1
2	15/12/2019	H16.0	1	Úlcera corneal	1
2	15/12/2019	H53	11	No patología	1
2	15/12/2019	H16.9	1	Queratitis	1
1	15/12/2019	T15.0	1	Cuerpo extraño	1
2	15/12/2019	H16.0	1	Úlcera corneal	1

1	15/12/2019	H16.9	1	Queratitis	1
1	15/12/2019	H11.3	0	Hiposfagma	1
2	15/12/2019	H11.00	0	Pterigium	1
1	15/12/2019	S05.9	9	Traumatismo	1
2	15/12/2019	H53	11	No patología	1
2	15/12/2019	H11.3	0	Hiposfagma	1
1	15/12/2019	S05.9	9	Traumatismo	1
1	16/12/2019	T15.0	1	Cuerpo extraño	1
1	16/12/2019	H00.02	4	Orzuelo	1
2	16/12/2019	H0.41	1	Ojo seco	1
2	16/12/2019	H02.05	4	Distiquiasis	1
2	16/12/2019	H00.02	4	Orzuelo	1
2	16/12/2019	H0.41	1	Ojo seco	1
2	16/12/2019	H43.81	7	DVP	1
2	16/12/2019	H47.10	10	Papiledema	1
1	16/12/2019	H11.3	0	Hiposfagma	1
1	16/12/2019	H10.3	0	Conjuntivitis	1
1	16/12/2019	Alta por fuga	11	Alta por fuga	1
2	16/12/2019	H16.9	1	Queratitis	1
1	16/12/2019	H16.0	1	Úlcera corneal	1
1	16/12/2019	H20	6	Uveitis	1
2	16/12/2019	H10.3	0	Conjuntivitis	1
2	16/12/2019	H16.9	1	Queratitis	1
2	16/12/2019	H16.0	1	Úlcera corneal	1
1	16/12/2019	S05.30	9	Laceración conjuntival	1
2	16/12/2019	H0.41	1	Ojo seco	1
2	16/12/2019	H10.3	0	Conjuntivitis	1
1	16/12/2019	H20	6	Uveitis	1
1	16/12/2019	H01.00	4	Blefaritis	1
2	16/12/2019	H43.1	7	Hemovítreo	1
1	16/12/2019	H16.9	1	Queratitis	1
2	16/12/2019	S05.9	9	Traumatismo	1
2	16/12/2019	H00.02	4	Orzuelo	1
1	16/12/2019	H53.9	11	Alteraciones visuales	1
2	16/12/2019	H43.81	7	DVP	1
2	16/12/2019	H01.00	4	Blefaritis	1
2	16/12/2019	H10.3	0	Conjuntivitis	1
2	16/12/2019	T15.0	1	Cuerpo extraño	1
2	16/12/2019	H16.9	1	Queratitis	1
2	16/12/2019	H16.0	1	Úlcera corneal	1
2	16/12/2019	H00.02	4	Orzuelo	1
2	16/12/2019	H16.9	1	Queratitis	1
1	16/12/2019	H35.30	8	DMAE	1
2	16/12/2019	H43.81	7	DVP	1
2	16/12/2019	H16.9	1	Queratitis	1
1	16/12/2019	H53.9	11	Alteraciones visuales	1
2	16/12/2019	H10.3	0	Conjuntivitis	1
1	16/12/2019	Alta por fuga	11	Alta por fuga	1
1	16/12/2019	S05.9	9	Traumatismo	1
2	16/12/2019	H35.30	8	DMAE	1
1	16/12/2019	H11.3	0	Hiposfagma	1
1	16/12/2019	H00.02	4	Orzuelo	1
2	16/12/2019	H35.3	8	Maculopatía	1
1	16/12/2019	H00.03	4	Celulitis preseptal	1
1	16/12/2019	H16.9	1	Queratitis	1
1	16/12/2019	H10.3	0	Conjuntivitis	1
1	16/12/2019	H16.9	1	Queratitis	1
2	16/12/2019	S05.9	9	Traumatismo	1
1	16/12/2019	G43.109	10	Aura	1
1	16/12/2019	T15.0	1	Cuerpo extraño	1
1	16/12/2019	H00.03	4	Celulitis preseptal	1
2	17/12/2019	H10.3	0	Conjuntivitis	1
2	17/12/2019	H11.3	0	Hiposfagma	1
1	17/12/2019	H16.9	1	Queratitis	1
2	17/12/2019	H10.3	0	Conjuntivitis	1
1	17/12/2019	H01.00	4	Blefaritis	1

2	17/12/2019	H53	11	No patología	1
1	17/12/2019	H10.3	0	Conjuntivitis	1
1	17/12/2019	H10.5	4	Blefaroconjuntivitis	1
2	17/12/2019	H20	6	Uveitis	1
2	17/12/2019	H16.0	1	Úlcera corneal	1
2	17/12/2019	H02	4	Alteración palpebral	1
2	17/12/2019	H10.3	0	Conjuntivitis	1
2	17/12/2019	H11.3	0	Hiposfagma	1
1	17/12/2019	H11.3	0	Hiposfagma	1
1	17/12/2019	H20	6	Uveitis	1
1	17/12/2019	Alta por fuga	11	Alta por fuga	1
2	17/12/2019	T26	9	Causticación	1
2	17/12/2019	H53	11	No patología	1
1	17/12/2019	H17.8	1	Infiltrados corneales	1
2	17/12/2019	H16.9	1	Queratitis	1
2	17/12/2019	G45.3	10	Amaurosis fugax	1
2	17/12/2019	G43.109	10	Aura	1
2	17/12/2019	H20	6	Uveitis	1
2	17/12/2019	H53.9	11	Alteraciones visuales	1
1	17/12/2019	H16.9	1	Queratitis	1
2	17/12/2019	S05.9	9	Traumatismo	1
2	17/12/2019	H53.9	11	Alteraciones visuales	1
2	17/12/2019	H33.30	8	Desgarro retiniano	1
1	17/12/2019	B00.1	4	Dermatitis herpética	1
1	17/12/2019	H53.9	11	Alteraciones visuales	1
1	17/12/2019	H53	11	No patología	1
2	17/12/2019	H40.9	3	Glaucoma	1
2	17/12/2019	H10.3	0	Conjuntivitis	1
2	17/12/2019	H10.3	0	Conjuntivitis	1
1	17/12/2019	H16.9	1	Queratitis	1
1	17/12/2019	T15.0	1	Cuerpo extraño	1
1	17/12/2019	H16.0	1	Úlcera corneal	1
2	17/12/2019	H43.81	7	DVP	1
1	17/12/2019	H16.9	1	Queratitis	1
1	17/12/2019	H11.44	0	Quiste conjuntival	1
1	17/12/2019	Alta por fuga	11	Alta por fuga	1
1	17/12/2019	H10.3	0	Conjuntivitis	1
1	17/12/2019	G45.3	10	Amaurosis fugax	1
1	17/12/2019	H16.9	1	Queratitis	1
2	17/12/2019	G43.109	10	Aura	1
1	17/12/2019	T15.0	1	Cuerpo extraño	1
2	17/12/2019	H16.9	1	Queratitis	1
1	17/12/2019	H20	6	Uveitis	1
1	17/12/2019	H16.9	1	Queratitis	1
1	18/12/2019	H16.9	1	Queratitis	1
1	18/12/2019	H02	4	Lesión palpebral	1
1	18/12/2019	H10.3	0	Conjuntivitis	1
1	18/12/2019	Alta por fuga	11	Alta por fuga	1
2	18/12/2019	H16.0	1	Úlcera corneal	1
2	18/12/2019	H27.8	2	OCP	1
2	18/12/2019	H43.81	7	DVP	1
2	18/12/2019	H20	6	Uveitis	1
2	18/12/2019	H10.3	0	Conjuntivitis	1
2	18/12/2019	H01.00	4	Blefaritis	1
1	18/12/2019	H16.0	1	Úlcera corneal	1
1	18/12/2019	H43.81	7	DVP	1
2	18/12/2019	H10.3	0	Conjuntivitis	1
2	18/12/2019	H10.3	0	Conjuntivitis	1
1	18/12/2019	H10.3	0	Conjuntivitis	1
1	18/12/2019	H16.9	1	Queratitis	1
1	18/12/2019	H16.0	1	Úlcera corneal	1
2	18/12/2019	H40.05	3	HTO	1
1	18/12/2019	S05.9	9	Traumatismo	1
1	18/12/2019	H17.8	1	Infiltrados corneales	1
2	18/12/2019	H35.30	8	DMAE	1
1	18/12/2019	H43.81	7	DVP	1

2	18/12/2019	H04.32	5	Dacriocistitis aguda	1
2	18/12/2019	H0.41	1	Ojo seco	1
2	18/12/2019	H43.81	7	DVP	1
1	18/12/2019	H43.81	7	DVP	1
1	18/12/2019	H00.02	4	Orzuelo	1
1	18/12/2019	T15.0	1	Cuerpo extraño	1
2	18/12/2019	H16.9	1	Queratitis	1
1	18/12/2019	S05.00	9	Abrasión conjuntival	1
2	18/12/2019	H01.00	4	Blefaritis	1
1	18/12/2019	T15.0	1	Cuerpo extraño	1
1	18/12/2019	H16.0	1	Úlcera corneal	1
2	18/12/2019	H02	4	Lesión palpebral	1
2	18/12/2019	S05.9	9	Traumatismo	1
1	18/12/2019	H20	6	Uveitis	1
1	18/12/2019	H15.00	6	Escleritis	1
2	18/12/2019	H16.9	1	Queratitis	1
2	18/12/2019	H00.19	4	Chalazión	1
1	18/12/2019	T15.0	1	Cuerpo extraño	1
1	19/12/2019	S05.30	9	Laceración conjuntival	1
2	19/12/2019	H00.03	4	Celulitis preseptal	1
1	19/12/2019	H15.00	6	Escleritis	1
1	19/12/2019	H16.0	1	Úlcera corneal	1
1	19/12/2019	T26	9	Causticación	1
2	19/12/2019	H53.9	11	Alteraciones visuales	1
2	19/12/2019	H18.50	1	Distrofia corneal	1
1	19/12/2019	H02	4	Alteración palpebral	1
1	19/12/2019	H20	6	Uveitis	1
1	19/12/2019	H01.00	4	Blefaritis	1
1	19/12/2019	H10.3	0	Conjuntivitis	1
1	19/12/2019	S05.9	9	Traumatismo	1
2	19/12/2019	H16.9	1	Queratitis	1
2	19/12/2019	H16.0	1	Úlcera corneal	1
2	19/12/2019	H20	6	Uveitis	1
1	19/12/2019	H11.3	0	Hiposfagma	1
1	19/12/2019	H16.9	1	Queratitis	1
1	19/12/2019	T15.0	1	Cuerpo extraño	1
2	19/12/2019	H0.41	1	Ojo seco	1
2	19/12/2019	H43.81	7	DVP	1
2	19/12/2019	H43.81	7	DVP	1
1	19/12/2019	T15.0	1	Cuerpo extraño	1
1	19/12/2019	H10.3	0	Conjuntivitis	1
1	19/12/2019	H20	6	Uveitis	1
1	19/12/2019	H33.30	8	Desgarro retiniano	1
1	19/12/2019	H16.9	1	Queratitis	1
1	19/12/2019	H11.3	0	Hiposfagma	1
1	19/12/2019	H16.9	1	Queratitis	1
1	19/12/2019	H10.3	0	Conjuntivitis	1
2	19/12/2019	S05.9	9	Traumatismo	1
1	19/12/2019	G43.9	10	Migraña	1
1	19/12/2019	H00.19	4	Chalazión	1
1	19/12/2019	H16.0	1	Úlcera corneal	1
1	19/12/2019	H00.02	4	Orzuelo	1
1	19/12/2019	S05.9	9	Traumatismo	1
2	19/12/2019	H53.9	11	Alteraciones visuales	1
1	19/12/2019	H11.00	0	Pterigium	1
1	19/12/2019	H04.32	5	Dacriocistitis aguda	1
1	19/12/2019	T15.0	1	Cuerpo extraño	1
1	19/12/2019	H20	6	Uveitis	1
1	19/12/2019	H16.9	1	Queratitis	1
1	19/12/2019	H11.3	0	Hiposfagma	1
1	19/12/2019	H40.21	3	Glaucoma agudo	1
2	19/12/2019	H10.3	0	Conjuntivitis	1
1	20/12/2019	H16.0	1	Úlcera corneal	1
1	20/12/2019	T15.0	1	Cuerpo extraño	1
2	20/12/2019	H0.41	1	Ojo seco	1
1	20/12/2019	H35.3	8	Maculopatía	1

1	20/12/2019	H00.03	4	Celulitis preseptal	1
2	20/12/2019	H16.9	1	Queratitis	1
1	20/12/2019	S05.30	9	Laceración conjuntival	1
2	20/12/2019	H10.3	0	Conjuntivitis	1
1	20/12/2019	H11.3	0	Hiposfagma	1
2	20/12/2019	H16.9	1	Queratitis	1
1	20/12/2019	H10.3	0	Conjuntivitis	1
2	20/12/2019	H00.03	4	Celulitis preseptal	1
2	20/12/2019	H43.81	7	DVP	1
2	20/12/2019	G43.109	10	Aura	1
1	20/12/2019	T26	9	Causticación	1
2	20/12/2019	H53.9	11	Alteraciones visuales	1
2	20/12/2019	H01.00	4	Blefaritis	1
2	20/12/2019	H01.00	4	Blefaritis	1
2	20/12/2019	H10.81	0	Pingueculitis	1
2	20/12/2019	H01.00	4	Blefaritis	1
2	20/12/2019	H16.9	1	Queratitis	1
2	20/12/2019	H01.00	4	Blefaritis	1
1	20/12/2019	H20	6	Uveitis	1
2	20/12/2019	B00.1	4	Dermatitis herpética	1
2	20/12/2019	B00.1	4	Dermatitis herpética	1
2	20/12/2019	H16.9	1	Queratitis	1
2	20/12/2019	H00.02	4	Orzuelo	1
1	20/12/2019	Alta por fuga	11	Alta por fuga	1
2	20/12/2019	H16.0	1	Úlcera corneal	1
2	20/12/2019	H53.10	11	Problema refractivo	1
1	20/12/2019	T15.0	1	Cuerpo extraño	1
2	20/12/2019	H00.02	4	Orzuelo	1
2	20/12/2019	H43.81	7	DVP	1
2	20/12/2019	H16.9	1	Queratitis	1
2	20/12/2019	H00.02	4	Orzuelo	1
1	20/12/2019	T15.0	1	Cuerpo extraño	1
1	20/12/2019	H31.42	8	Coroidopatía serosa central	1
1	20/12/2019	H16.9	1	Queratitis	1
2	21/12/2019	H11.3	0	Hiposfagma	1
1	21/12/2019	T15.0	1	Cuerpo extraño	1
2	21/12/2019	H40.21	3	Glaucoma agudo	1
2	21/12/2019	H40.05	3	HTO	1
2	21/12/2019	H16.9	1	Queratitis	1
1	21/12/2019	H16.0	1	Úlcera corneal	1
1	21/12/2019	H59.88	8	Post op retina	1
2	21/12/2019	H16.9	1	Queratitis	1
2	21/12/2019	H16.0	1	Úlcera corneal	1
1	21/12/2019	H10.3	0	Conjuntivitis	1
1	21/12/2019	T15.0	1	Cuerpo extraño	1
2	21/12/2019	H16.9	1	Queratitis	1
2	21/12/2019	H35.30	8	DMAE	1
2	21/12/2019	H20	6	Uveitis	1
1	21/12/2019	H11.3	0	Hiposfagma	1
1	21/12/2019	H11.3	0	Hiposfagma	1
2	21/12/2019	H16.9	1	Queratitis	1
1	21/12/2019	H16.9	1	Queratitis	1
2	21/12/2019	H16.9	1	Queratitis	1
2	21/12/2019	H10.3	0	Conjuntivitis	1
1	21/12/2019	S05.9	9	Traumatismo	1
2	21/12/2019	H10.3	0	Conjuntivitis	1
2	21/12/2019	H10.3	0	Conjuntivitis	1
1	21/12/2019	H16.0	1	Úlcera corneal	1
2	21/12/2019	S05.00	9	Abrasión conjuntival	1
2	21/12/2019	H01.00	4	Blefaritis	1
1	21/12/2019	H16.0	1	Úlcera corneal	1
1	21/12/2019	H10.3	0	Conjuntivitis	1
1	21/12/2019	H16.9	1	Queratitis	1
1	21/12/2019	T26	9	Causticación	1
2	21/12/2019	H00.02	4	Orzuelo	1
2	21/12/2019	H20	6	Uveitis	1

2	21/12/2019	T15.0	1	Cuerpo extraño	1
1	21/12/2019	Alta por fuga	11	Alta por fuga	1
1	21/12/2019	T15.0	1	Cuerpo extraño	1
1	21/12/2019	S05.9	9	Traumatismo	1
2	21/12/2019	H16.0	1	Úlcera corneal	1
2	21/12/2019	H10.3	0	Conjuntivitis	1
2	21/12/2019	H40.05	3	HTO	1
1	22/12/2019	B00.1	4	Dermatitis herpética	1
1	22/12/2019	T15.0	1	Cuerpo extraño	1
1	22/12/2019	H16.0	1	Úlcera corneal	1
2	22/12/2019	H01.00	4	Blefaritis	1
2	22/12/2019	H10.3	0	Conjuntivitis	1
1	22/12/2019	H16.0	1	Úlcera corneal	1
2	22/12/2019	H16.0	1	Úlcera corneal	1
2	22/12/2019	H10.3	0	Conjuntivitis	1
2	22/12/2019	H10.5	4	Blefaroconjuntivitis	1
1	22/12/2019	Z98.4	2	Post op catarata	1
2	22/12/2019	H00.03	4	Celulitis preseptal	1
2	22/12/2019	H16.0	1	Úlcera corneal	1
2	22/12/2019	H01.00	4	Blefaritis	1
2	22/12/2019	H16.9	1	Queratitis	1
2	22/12/2019	H16.0	1	Úlcera corneal	1
2	22/12/2019	H10.3	0	Conjuntivitis	1
1	22/12/2019	H10.3	0	Conjuntivitis	1
2	22/12/2019	H10.5	4	Blefaroconjuntivitis	1
1	22/12/2019	H10.5	4	Blefaroconjuntivitis	1
2	22/12/2019	H10.3	0	Conjuntivitis	1
2	22/12/2019	H53	11	No patología	1
1	22/12/2019	H16.9	1	Queratitis	1
1	22/12/2019	H00.02	4	Orzuelo	1
1	22/12/2019	H43.81	7	DVP	1
1	22/12/2019	H10.3	0	Conjuntivitis	1
2	22/12/2019	H10.5	4	Blefaroconjuntivitis	1
2	22/12/2019	H33.30	8	Desgarro retiniano	1
2	22/12/2019	H00.02	4	Orzuelo	1
1	22/12/2019	H00.02	4	Orzuelo	1
1	22/12/2019	H16.9	1	Queratitis	1
1	22/12/2019	H40.9	3	Glaucoma	1
2	22/12/2019	H10.3	0	Conjuntivitis	1
2	22/12/2019	H0.41	1	Ojo seco	1
1	22/12/2019	H01.00	4	Blefaritis	1
2	22/12/2019	H16.0	1	Úlcera corneal	1
1	22/12/2019	E11.319	8	Retinopatía diabética	1
2	22/12/2019	H16.9	1	Queratitis	1
2	22/12/2019	T26	9	Causticación	1
2	22/12/2019	H10.3	0	Conjuntivitis	1
1	22/12/2019	S05.9	9	Traumatismo	1
2	22/12/2019	H02	4	Alteración palpebral	1
2	22/12/2019	T15.0	1	Cuerpo extraño	1
2	22/12/2019	H16.9	1	Queratitis	1
2	23/12/2019	H04.32	5	Dacriocistitis aguda	1
2	23/12/2019	T26.4	9	Quemadura	1
1	23/12/2019	H10.3	0	Conjuntivitis	1
1	23/12/2019	H10.3	0	Conjuntivitis	1
2	23/12/2019	H00.02	4	Orzuelo	1
2	23/12/2019	H20	6	Uveitis	1
1	23/12/2019	H00.02	4	Orzuelo	1
1	23/12/2019	H00.03	4	Celulitis preseptal	1
2	23/12/2019	H11.3	0	Hiposfagma	1
1	23/12/2019	H16.0	1	Úlcera corneal	1
2	23/12/2019	H01.00	4	Blefaritis	1
1	23/12/2019	T15.0	1	Cuerpo extraño	1
2	23/12/2019	H0.41	1	Ojo seco	1
1	23/12/2019	T15.0	1	Cuerpo extraño	1
1	23/12/2019	H0.41	1	Ojo seco	1
2	23/12/2019	H00.02	4	Orzuelo	1

2	23/12/2019	H0.41	1	Ojo seco	1
1	23/12/2019	H33.0	8	Desprendimiento de retina	1
1	23/12/2019	H10.3	1	Conjuntivitis	1
1	23/12/2019	H17.8	1	Infiltrados corneales	1
2	23/12/2019	H04.32	5	Dacriocistitis aguda	1
1	23/12/2019	H27.8	2	OCP	1
2	23/12/2019	H16.9	1	Queratitis	1
2	23/12/2019	H16.9	1	Queratitis	1
2	23/12/2019	H16.0	1	Úlcera corneal	1
2	23/12/2019	G43.9	10	Migraña	1
2	23/12/2019	H18.20	1	Edema corneal	1
1	23/12/2019	H26.9	2	Catarata	1
1	23/12/2019	H16.9	1	Queratitis	1
1	23/12/2019	H26.9	2	Catarata	1
1	23/12/2019	H10.3	0	Conjuntivitis	1
1	23/12/2019	H53.9	11	Alteraciones visuales	1
2	23/12/2019	H43.81	7	DVP	1
1	23/12/2019	H10.3	0	Conjuntivitis	1
2	23/12/2019	H53.9	11	Alteraciones visuales	1
1	23/12/2019	H10.3	0	Conjuntivitis	1
2	23/12/2019	H00.02	4	Orzuelo	1
2	23/12/2019	H43.81	7	DVP	1
1	23/12/2019	H53	11	No patología	1
2	23/12/2019	G45.3	10	Amaurosis fugax	1
1	23/12/2019	H16.9	1	Queratitis	1
2	23/12/2019	H16.0	1	Úlcera corneal	1
2	23/12/2019	H53.9	11	Alteraciones visuales	1
2	23/12/2019	H20	6	Uveitis	1
1	23/12/2019	H10.3	0	Conjuntivitis	1
2	24/12/2019	H20	6	Uveitis	1
2	24/12/2019	H10.3	0	Conjuntivitis	1
2	24/12/2019	H10.3	0	Conjuntivitis	1
2	24/12/2019	H01.00	4	Blefaritis	1
2	24/12/2019	H10.3	0	Conjuntivitis	1
2	24/12/2019	H00.02	4	Orzuelo	1
1	24/12/2019	H01.00	4	Blefaritis	1
2	24/12/2019	S05.9	9	Traumatismo	1
2	24/12/2019	H02	4	Alteración palpebral	1
1	24/12/2019	H43.81	7	DVP	1
2	24/12/2019	H40.9	3	Glaucoma	1
2	24/12/2019	H16.0	1	Úlcera corneal	1
1	24/12/2019	S05.9	9	Traumatismo	1
2	24/12/2019	H53	11	No patología	1
1	24/12/2019	H16.9	1	Queratitis	1
2	24/12/2019	H27.10	2	Luxación LIO	1
2	24/12/2019	H43.81	7	DVP	1
2	24/12/2019	H53.9	11	Alteraciones visuales	1
2	24/12/2019	H02.05	4	Distiquiasis	1
2	24/12/2019	H10.3	0	Conjuntivitis	1
1	24/12/2019	H16.9	1	Queratitis	1
1	24/12/2019	H20	6	Uveitis	1
1	24/12/2019	S05.9	9	Traumatismo	1
2	24/12/2019	H10.3	0	Conjuntivitis	1
1	24/12/2019	H16.9	1	Queratitis	1
1	24/12/2019	H16.9	1	Queratitis	1
1	24/12/2019	H16.9	1	Queratitis	1
2	24/12/2019	H53.9	11	Alteraciones visuales	1
2	24/12/2019	H53.9	11	Alteraciones visuales	1
2	24/12/2019	H10.3	0	Conjuntivitis	1
1	24/12/2019	H16.0	1	Úlcera corneal	1
2	24/12/2019	H43.81	7	DVP	1
1	24/12/2019	S05.9	9	Traumatismo	1
2	24/12/2019	H10.3	0	Conjuntivitis	1
1	25/12/2019	S05.9	9	Traumatismo	1
2	25/12/2019	H16.9	1	Queratitis	1
2	25/12/2019	H16.0	1	Úlcera corneal	1

1	25/12/2019	H20	6	Uveitis	1
1	25/12/2019	H10.3	0	Conjuntivitis	1
2	25/12/2019	H16.9	1	Queratitis	1
2	25/12/2019	H16.9	1	Queratitis	1
1	25/12/2019	H10.3	0	Conjuntivitis	1
1	25/12/2019	H0.41	1	Ojo seco	1
2	25/12/2019	H04.32	5	Dacriocistitis aguda	1
2	25/12/2019	H01.00	4	Blefaritis	1
2	25/12/2019	H16.9	1	Queratitis	1
1	25/12/2019	H33.30	8	Desgarro retiniano	1
1	25/12/2019	H16.9	1	Queratitis	1
1	25/12/2019	H33.0	8	Desprendimiento de retina	1
2	25/12/2019	H10.3	0	Conjuntivitis	1
2	25/12/2019	H10.3	0	Conjuntivitis	1
1	25/12/2019	H10.3	0	Conjuntivitis	1
2	25/12/2019	H02	4	Alteración palpebral	1
2	25/12/2019	H11.3	0	Hiposfagma	1
1	25/12/2019	E11.319	8	Retinopatía diabética	1
1	25/12/2019	H11.3	0	Hiposfagma	1
2	25/12/2019	H20	6	Uveitis	1
2	25/12/2019	H18.20	1	Edema corneal	1
2	25/12/2019	H16.0	1	Úlcera corneal	1
1	25/12/2019	S05.9	9	Traumatismo	1
2	25/12/2019	H11.3	0	Hiposfagma	1
2	25/12/2019	H00.02	4	Orzuelo	1
2	25/12/2019	H16.0	1	Úlcera corneal	1
2	26/12/2019	H00.02	4	Orzuelo	1
1	26/12/2019	H10.3	0	Conjuntivitis	1
2	26/12/2019	H10.3	0	Conjuntivitis	1
1	26/12/2019	H10.3	0	Conjuntivitis	1
1	26/12/2019	H10.3	0	Conjuntivitis	1
2	26/12/2019	H20	6	Uveitis	1
1	26/12/2019	H47.10	10	Papiledema	1
2	26/12/2019	H17.8	1	Infiltrados corneales	1
1	26/12/2019	H16.9	1	Queratitis	1
1	26/12/2019	H16.9	1	Queratitis	1
2	26/12/2019	H10.3	0	Conjuntivitis	1
2	26/12/2019	H16.9	1	Queratitis	1
1	26/12/2019	H16.0	1	Úlcera corneal	1
2	26/12/2019	H11.3	0	Hiposfagma	1
1	26/12/2019	T15.0	1	Cuerpo extraño	1
2	26/12/2019	H16.0	1	Úlcera corneal	1
1	26/12/2019	H16.9	1	Queratitis	1
1	26/12/2019	H11.3	0	Hiposfagma	1
1	26/12/2019	H10.3	0	Conjuntivitis	1
2	26/12/2019	H16.0	1	Úlcera corneal	1
2	26/12/2019	H34.82	8	Trombosis venosa	1
2	26/12/2019	H16.9	1	Queratitis	1
2	26/12/2019	H10.3	0	Conjuntivitis	1
2	26/12/2019	T15.0	1	Cuerpo extraño	1
2	26/12/2019	H10.3	0	Conjuntivitis	1
2	26/12/2019	H43.81	7	DVP	1
2	26/12/2019	H10.3	0	Conjuntivitis	1
2	26/12/2019	H10.3	0	Conjuntivitis	1
2	26/12/2019	H10.3	0	Conjuntivitis	1
2	26/12/2019	H43.81	7	DVP	1
1	26/12/2019	H16.9	1	Queratitis	1
2	26/12/2019	H43.81	7	DVP	1
2	26/12/2019	H35.3	8	Maculopatía	1
2	26/12/2019	H33.0	8	Desprendimiento de retina	1
2	26/12/2019	H16.9	1	Queratitis	1
2	26/12/2019	H53.9	11	Alteraciones visuales	1
2	26/12/2019	H16.9	1	Queratitis	1
2	26/12/2019	T15.0	1	Cuerpo extraño	1
2	26/12/2019	Alta por fuga	11	Alta por fuga	1
2	26/12/2019	H53.9	11	Alteraciones visuales	1

2	26/12/2019	H16.9	1	Queratitis	1
2	26/12/2019	H16.9	1	Queratitis	1
2	26/12/2019	H10.3	0	Conjuntivitis	1
2	26/12/2019	H35.30	8	DMAE	1
2	26/12/2019	H53.9	11	Alteraciones visuales	1
2	26/12/2019	T26.4	9	Quemadura	1
1	26/12/2019	H10.3	0	Conjuntivitis	1
1	27/12/2019	H16.0	1	Úlcera corneal	1
1	27/12/2019	H16.0	1	Úlcera corneal	1
2	27/12/2019	H53.9	11	Alteraciones visuales	1
1	27/12/2019	H43.81	7	DVP	1
1	27/12/2019	H16.0	1	Úlcera corneal	1
1	27/12/2019	H10.3	0	Conjuntivitis	1
1	27/12/2019	S05.30	9	Laceración conjuntival	1
1	27/12/2019	H01.00	4	Blefaritis	1
1	27/12/2019	H43.1	7	Hemovítreo	1
2	27/12/2019	H16.9	1	Queratitis	1
2	27/12/2019	H53.9	11	Alteraciones visuales	1
2	27/12/2019	H43.81	7	DVP	1
1	27/12/2019	T15.0	1	Cuerpo extraño	1
1	27/12/2019	H43.1	7	Hemovítreo	1
2	27/12/2019	H43.81	7	DVP	1
2	27/12/2019	H15.00	6	Escleritis	1
2	27/12/2019	H10.3	0	Conjuntivitis	1
1	27/12/2019	H27.8	2	OCP	1
1	27/12/2019	H53	11	No patología	1
2	27/12/2019	H02.05	4	Distiquiasis	1
2	27/12/2019	H27.8	2	OCP	1
2	27/12/2019	H10.3	0	Conjuntivitis	1
1	27/12/2019	H15.1	6	Epiescleritis	1
1	27/12/2019	H10.3	0	Conjuntivitis	1
1	27/12/2019	H40.21	3	Glaucoma agudo	1
2	27/12/2019	G43.109	10	Aura	1
2	27/12/2019	H16.9	1	Queratitis	1
2	27/12/2019	H04.32	5	Dacriocistitis aguda	1
2	27/12/2019	H27.8	2	OCP	1
2	27/12/2019	H00.02	4	Orzuelo	1
1	27/12/2019	H16.9	1	Queratitis	1
1	27/12/2019	T15.0	1	Cuerpo extraño	1
1	27/12/2019	H16.0	1	Úlcera corneal	1
1	27/12/2019	S05.9	9	Traumatismo	1
2	27/12/2019	H02	4	Alteración palpebral	1
2	27/12/2019	H53	11	No patología	1
1	27/12/2019	T15.0	1	Cuerpo extraño	1
2	27/12/2019	H20	6	Uveitis	1
2	27/12/2019	H11.3	0	Hiposfagma	1
2	27/12/2019	H10.3	0	Conjuntivitis	1
1	27/12/2019	H26.9	2	Catarata	1
1	27/12/2019	H33.0	8	Desprendimiento de retina	1
2	27/12/2019	H00.02	4	Orzuelo	1
2	27/12/2019	H34.82	8	Trombosis venosa	1
1	27/12/2019	S05.9	9	Traumatismo	1
2	27/12/2019	H16.9	1	Queratitis	1
1	27/12/2019	H16.9	1	Queratitis	1
1	27/12/2019	H00.19	4	Chalazión	1
1	27/12/2019	H10.3	0	Conjuntivitis	1
1	27/12/2019	H00.02	4	Orzuelo	1
1	28/12/2019	H10.3	0	Conjuntivitis	1
1	28/12/2019	H35.3	8	Maculopatía	1
2	28/12/2019	H40.21	3	Glaucoma agudo	1
2	28/12/2019	H10.3	0	Conjuntivitis	1
2	28/12/2019	H00.02	4	Orzuelo	1
1	28/12/2019	H10.3	0	Conjuntivitis	1
1	28/12/2019	H35.3	8	Maculopatía	1
2	28/12/2019	H10.3	0	Conjuntivitis	1
2	28/12/2019	H16.0	1	Úlcera corneal	1

1	28/12/2019	H20	6	Uveitis	1
1	28/12/2019	H16.9	1	Queratitis	1
1	28/12/2019	T15.0	1	Cuerpo extraño	1
1	28/12/2019	H53.10	11	Problema refractivo	1
2	28/12/2019	H04.32	5	Dacriocistitis aguda	1
2	28/12/2019	H02.05	4	Distiquiasis	1
2	28/12/2019	H16.9	1	Queratitis	1
2	28/12/2019	H01.00	4	Blefaritis	1
1	28/12/2019	H18.50	1	Distrofia corneal	1
2	28/12/2019	H10.3	0	Conjuntivitis	1
1	28/12/2019	H10.3	0	Conjuntivitis	1
1	28/12/2019	H16.0	1	Úlcera corneal	1
2	28/12/2019	H00.02	4	Orzuelo	1
1	28/12/2019	H40.9	3	Glaucoma	1
1	28/12/2019	H10.3	0	Conjuntivitis	1
1	28/12/2019	H43.81	8	DVP	1
2	28/12/2019	H10.3	0	Conjuntivitis	1
1	28/12/2019	H10.3	0	Conjuntivitis	1
2	28/12/2019	H10.3	0	Conjuntivitis	1
1	28/12/2019	S05.9	9	Traumatismo	1
1	28/12/2019	H16.9	1	Queratitis	1
1	28/12/2019	S05.9	9	Traumatismo	1
2	28/12/2019	H01.00	4	Blefaritis	1
1	28/12/2019	H20	6	Uveitis	1
2	28/12/2019	H43.81	7	DVP	1
1	28/12/2019	H00.02	4	Orzuelo	1
2	28/12/2019	T26	9	Causticación	1
1	28/12/2019	H20	6	Uveitis	1
2	28/12/2019	Alta por fuga	11	Alta por fuga	1
2	28/12/2019	H49.9	10	Parálisis oculomotora	1
2	28/12/2019	H00.02	4	Orzuelo	1
2	28/12/2019	H43.81	7	DVP	1
2	28/12/2019	H11.3	0	Hiposfagma	1
2	28/12/2019	H43.81	7	DVP	1
1	28/12/2019	H10.3	0	Conjuntivitis	1
1	28/12/2019	H26.9	2	Catarata	1
2	28/12/2019	H00.02	4	Orzuelo	1
1	28/12/2019	H10.3	0	Conjuntivitis	1
2	28/12/2019	H43.81	7	DVP	1
1	28/12/2019	H10.3	0	Conjuntivitis	1
2	28/12/2019	H53	11	No patología	1
2	28/12/2019	G43.109	10	Aura	1
2	28/12/2019	S05.00	9	Abrasión conjuntival	1
2	28/12/2019	H16.9	1	Queratitis	1
1	28/12/2019	H10.3	0	Conjuntivitis	1
2	28/12/2019	H00.02	4	Orzuelo	1
2	28/12/2019	B00.1	4	Dermatitis herpética	1
2	28/12/2019	H43.81	7	DVP	1
2	28/12/2019	H01.00	4	Blefaritis	1
1	28/12/2019	H10.3	0	Conjuntivitis	1
1	28/12/2019	H17.8	1	Infiltrados corneales	1
2	28/12/2019	H16.9	1	Queratitis	1
1	28/12/2019	H16.0	1	Úlcera corneal	1
1	28/12/2019	H16.0	1	Úlcera corneal	1
2	29/12/2019	H11.3	0	Hiposfagma	1
1	29/12/2019	H16.0	1	Úlcera corneal	1
1	29/12/2019	H10.3	0	Conjuntivitis	1
2	29/12/2019	S05.9	9	Traumatismo	1
2	29/12/2019	H16.9	1	Queratitis	1
1	29/12/2019	H11.00	0	Pterigium	1
2	29/12/2019	H10.3	0	Conjuntivitis	1
1	29/12/2019	H16.0	1	Úlcera corneal	1
1	29/12/2019	H43.1	7	Hemovítreo	1
2	29/12/2019	H43.81	7	DVP	1
2	29/12/2019	H17.8	1	Infiltrados corneales	1
2	29/12/2019	H53.9	11	Alteraciones visuales	1

1	29/12/2019	H20	6	Uveitis	1
2	29/12/2019	H16.0	1	Úlcera corneal	1
2	29/12/2019	H16.9	1	Queratitis	1
2	29/12/2019	H00.02	4	Orzuelo	1
1	29/12/2019	H20	6	Uveitis	1
2	29/12/2019	H0.41	1	Ojo seco	1
2	29/12/2019	T26	9	Causticación	1
1	29/12/2019	T15.0	1	Cuerpo extraño	1
2	29/12/2019	H01.00	4	Blefaritis	1
2	29/12/2019	H04.32	5	Dacriocistitis aguda	1
2	29/12/2019	H02.05	4	Distiquiasis	1
1	29/12/2019	H10.3	0	Conjuntivitis	1
1	29/12/2019	H26.9	2	Catarata	1
2	29/12/2019	H16.0	1	Úlcera corneal	1
2	29/12/2019	T15.0	1	Cuerpo extraño	1
2	29/12/2019	H40.21	3	Glaucoma agudo	1
2	29/12/2019	H43.81	7	DVP	1
2	29/12/2019	H16.9	1	Queratitis	1
1	29/12/2019	S05.9	9	Traumatismo	1
2	29/12/2019	H01.00	4	Blefaritis	1
1	29/12/2019	T15.0	1	Cuerpo extraño	1
2	29/12/2019	H10.3	0	Conjuntivitis	1
1	29/12/2019	S05.9	9	Traumatismo	1
1	29/12/2019	B00.1	4	Dermatitis herpética	1
1	30/12/2019	H16.9	1	Queratitis	1
1	30/12/2019	H11.3	0	Hiposfagma	1
1	30/12/2019	T15.0	1	Cuerpo extraño	1
1	30/12/2019	H10.3	0	Conjuntivitis	1
1	30/12/2019	H33.0	8	Desprendimiento de retina	1
2	30/12/2019	H16.9	1	Queratitis	1
2	30/12/2019	H16.9	1	Queratitis	1
2	30/12/2019	H01.00	4	Blefaritis	1
1	30/12/2019	H16.9	1	Queratitis	1
1	30/12/2019	H16.0	1	Úlcera corneal	1
1	30/12/2019	Alta por fuga	11	Alta por fuga	1
1	30/12/2019	T15.0	1	Cuerpo extraño	1
2	30/12/2019	H0.41	1	Ojo seco	1
2	30/12/2019	H43.81	7	DVP	1
1	30/12/2019	H00.02	4	Orzuelo	1
1	30/12/2019	H10.3	0	Conjuntivitis	1
2	30/12/2019	H15.1	6	Epiescleritis	1
1	30/12/2019	H33.30	8	Desgarro retiniano	1
2	30/12/2019	H00.02	4	Orzuelo	1
2	30/12/2019	S05.9	9	Traumatismo	1
1	30/12/2019	H11.3	0	Hiposfagma	1
2	30/12/2019	S05.9	9	Traumatismo	1
1	30/12/2019	H16.9	1	Queratitis	1
1	30/12/2019	T15.0	1	Cuerpo extraño	1
1	30/12/2019	H10.3	0	Conjuntivitis	1
1	30/12/2019	H04.32	5	Dacriocistitis aguda	1
1	30/12/2019	H02.05	4	Distiquiasis	1
2	30/12/2019	H01.00	4	Blefaritis	1
2	30/12/2019	T15.0	1	Cuerpo extraño	1
1	30/12/2019	H43.81	7	DVP	1
1	30/12/2019	H00.02	4	Orzuelo	1
1	30/12/2019	H43.81	7	DVP	1
2	30/12/2019	H04.32	5	Dacriocistitis aguda	1
2	30/12/2019	H10.3	0	Conjuntivitis	1
2	30/12/2019	T26	9	Causticación	1
2	30/12/2019	H53.9	11	Alteraciones visuales	1
1	30/12/2019	H10.3	0	Conjuntivitis	1
2	30/12/2019	H16.0	1	Úlcera corneal	1
1	30/12/2019	H10.3	0	Conjuntivitis	1
1	30/12/2019	T15.0	1	Cuerpo extraño	1
1	30/12/2019	H53.9	11	Alteraciones visuales	1
2	30/12/2019	H10.3	0	Conjuntivitis	1

1	30/12/2019	H31.42	8	Coroidopatía serosa central	1
1	30/12/2019	H16.0	1	Úlcera corneal	1
2	30/12/2019	H00.02	4	Orzuelo	1
2	30/12/2019	H40.5	3	Glaucoma secundario	1
2	30/12/2019	T26	9	Causticación	1
2	30/12/2019	S05.9	9	Traumatismo	1
1	30/12/2019	S05.30	9	Laceración conjuntival	1
1	30/12/2019	H00.02	4	Orzuelo	1
2	30/12/2019	H04.32	5	Dacriocistitis aguda	1
1	30/12/2019	H16.0	1	Úlcera corneal	1
1	30/12/2019	H15.1	6	Epiescleritis	1
2	31/12/2019	H00.02	4	Orzuelo	1
1	31/12/2019	H10.3	0	Conjuntivitis	1
1	31/12/2019	H10.3	0	Conjuntivitis	1
2	31/12/2019	H10.3	0	Conjuntivitis	1
1	31/12/2019	H00.03	4	Celulitis preseptal	1
1	31/12/2019	H53.10	11	Problema refractivo	1
2	31/12/2019	H16.0	1	Úlcera corneal	1
1	31/12/2019	H10.3	0	Conjuntivitis	1
2	31/12/2019	H00.02	4	Orzuelo	1
2	31/12/2019	H17.8	1	Infiltrados corneales	1
1	31/12/2019	H0.41	1	Ojo seco	1
2	31/12/2019	H16.0	1	Úlcera corneal	1
2	31/12/2019	H00.02	4	Orzuelo	1
1	31/12/2019	S05.30	9	Laceración conjuntival	1
2	31/12/2019	G43.9	10	Migraña	1
1	31/12/2019	H00.02	4	Orzuelo	1
2	31/12/2019	H11.3	0	Hiposfagma	1
1	31/12/2019	H01.00	4	Blefaritis	1
2	31/12/2019	H53.9	11	Alteraciones visuales	1
2	31/12/2019	H26.9	2	Catarata	1
1	31/12/2019	H10.3	0	Conjuntivitis	1
2	31/12/2019	H16.0	1	Úlcera corneal	1
1	31/12/2019	H16.0	1	Úlcera corneal	1
1	31/12/2019	T15.0	1	Cuerpo extraño	1
1	31/12/2019	T15.0	1	Cuerpo extraño	1
1	31/12/2019	H53.9	11	Alteraciones visuales	1
2	01/01/2020	H10.3	0	Conjuntivitis	1
2	01/01/2020	H16.0	1	Úlcera corneal	1
1	01/01/2020	H16.0	1	Úlcera corneal	1
2	01/01/2020	H10.3	0	Conjuntivitis	1
1	01/01/2020	T15.0	1	Cuerpo extraño	1
1	01/01/2020	H20	6	Uveitis	1
1	01/01/2020	H15.1	6	Epiescleritis	1
1	01/01/2020	T15.0	1	Cuerpo extraño	1
1	01/01/2020	H0.41	1	ojo seco	1
1	01/01/2020	H53	11	No patología	1
1	01/01/2020	H10.3	0	Conjuntivitis	1
1	01/01/2020	H10.3	0	Conjuntivitis	1
1	01/01/2020	H33.0	8	Desprendimiento de retina	1
1	01/01/2020	T15.0	1	Cuerpo extraño	1
1	01/01/2020	S05.9	9	Traumatismo	1
2	01/01/2020	H11.3	0	Hiposfagma	1
2	01/01/2020	S05.9	9	Traumatismo	1
2	01/01/2020	H10.3	0	Conjuntivitis	1
1	01/01/2020	H35.30	8	DMAE	1
1	01/01/2020	H10.3	0	Conjuntivitis	1
1	01/01/2020	H16.9	1	Queratitis	1
2	01/01/2020	H10.3	0	Conjuntivitis	1
1	01/01/2020	T15.0	1	Cuerpo extraño	1
1	01/01/2020	H10.3	0	Conjuntivitis	1
1	01/01/2020	H16.9	1	Queratitis	1
2	01/01/2020	S05.9	9	Traumatismo	1
1	01/01/2020	H33.30	8	Desgarro retiniano	1
2	01/01/2020	H15.1	6	Epiescleritis	1
2	01/01/2020	H33.30	8	Desgarro retiniano	1

1	01/01/2020	H40.21	3	Glaucoma agudo	1
2	01/01/2020	H16.0	1	Úlcera corneal	1
2	01/01/2020	H16.9	1	Queratitis	1
1	02/01/2020	S05.9	9	Traumatismo	1
2	02/01/2020	H11.3	0	Hiposfagma	1
2	02/01/2020	S05.30	9	Laceración conjuntival	1
2	02/01/2020	H16.0	1	Úlcera corneal	1
2	02/01/2020	H10.3	0	Conjuntivitis	1
2	02/01/2020	H10.3	0	Conjuntivitis	1
2	02/01/2020	H10.3	0	Conjuntivitis	1
2	02/01/2020	H16.0	1	Úlcera corneal	1
2	02/01/2020	H16.9	1	Queratitis	1
2	02/01/2020	H35.30	8	DMAE	1
2	02/01/2020	Alta por fuga	11	Alta por fuga	1
1	02/01/2020	H10.3	0	Conjuntivitis	1
1	02/01/2020	H16.9	1	Queratitis	1
1	02/01/2020	B00.1	4	Dermatitis herpética	1
1	02/01/2020	T15.0	1	Cuerpo extraño	1
2	02/01/2020	H10.3	0	Conjuntivitis	1
2	02/01/2020	H10.3	0	Conjuntivitis	1
1	02/01/2020	H16.9	1	Queratitis	1
1	02/01/2020	T26.4	9	Quemadura	1
2	02/01/2020	H10.3	0	Conjuntivitis	1
1	02/01/2020	H16.0	1	Úlcera corneal	1
1	02/01/2020	H17.8	1	Infiltrados corneales	1
1	02/01/2020	H10.3	0	Conjuntivitis	1
2	02/01/2020	H16.9	1	Queratitis	1
2	02/01/2020	H16.9	1	Queratitis	1
2	02/01/2020	H10.81	0	Pingueculitis	1
1	02/01/2020	H33.30	8	Desgarro retiniano	1
1	02/01/2020	S05.9	9	Traumatismo	1
2	02/01/2020	H00.03	4	Celulitis preseptal	1
1	02/01/2020	H16.0	1	Úlcera corneal	1
2	02/01/2020	H35.37	8	MER	1
1	02/01/2020	H49.9	10	Parálisis oculomotora	1
1	02/01/2020	H10.3	0	Conjuntivitis	1
2	02/01/2020	H00.19	4	Chalazión	1
1	02/01/2020	H16.9	1	Queratitis	1
2	02/01/2020	H43.81	7	DVP	1
2	02/01/2020	H11.3	0	Hiposfagma	1
2	02/01/2020	H01.00	4	Blefaritis	1
2	02/01/2020	H11.3	0	Hiposfagma	1
1	02/01/2020	H11.3	0	Hiposfagma	1
2	02/01/2020	H16.9	1	Queratitis	1
2	02/01/2020	H10.3	0	Conjuntivitis	1
2	02/01/2020	H16.9	1	Queratitis	1
1	02/01/2020	H16.9	1	Queratitis	1
1	02/01/2020	H35.30	8	DMAE	1
2	02/01/2020	H43.81	7	DVP	1
1	02/01/2020	H10.81	0	Pingueculitis	1
1	02/01/2020	H10.3	0	Conjuntivitis	1
2	02/01/2020	H16.9	1	Queratitis	1
1	02/01/2020	T26	9	Causticación	1
2	02/01/2020	H10.3	0	Conjuntivitis	1
1	02/01/2020	S05.00	9	Abrasión conjuntival	1
1	02/01/2020	H10.3	0	Conjuntivitis	1
2	02/01/2020	H10.3	0	Conjuntivitis	1
1	02/01/2020	H16.9	1	Queratitis	1
2	02/01/2020	H10.3	0	Conjuntivitis	1
1	02/01/2020	H10.3	0	Conjuntivitis	1
1	02/01/2020	B00.1	4	Dermatitis herpética	1
1	02/01/2020	H16.9	1	Queratitis	1
2	02/01/2020	H10.3	0	Conjuntivitis	1
2	02/01/2020	H01.00	4	Blefaritis	1
2	02/01/2020	H11.3	0	Hiposfagma	1
1	02/01/2020	H43.81	7	DVP	1

1	03/01/2020	H00.19	4	Chalazión		1
1	03/01/2020	H10.3	0	Conjuntivitis		1
2	03/01/2020	H02	4	Alteración palpebral		1
2	03/01/2020	H16.9	1	Queratitis		1
2	03/01/2020	H10.3	0	Conjuntivitis		1
2	03/01/2020	H16.9	1	Queratitis		1
2	03/01/2020	H20	6	Uveitis		1
2	03/01/2020	H33.30	8	Desgarro retiniano		1
2	03/01/2020	H10.3	0	Conjuntivitis		1
1	03/01/2020	T15.0	1	Cuerpo extraño		1
2	03/01/2020	H10.3	0	Conjuntivitis		1
2	03/01/2020	H43.1	7	Hemovítreo		1
2	03/01/2020	G43.9	10	Migraña		1
1	03/01/2020	H47.10	10	Papiledema		1
1	03/01/2020	H10.3	0	Conjuntivitis		1
1	03/01/2020	H16.9	1	Queratitis		1
1	03/01/2020	H16.0	1	Úlcera corneal		1
2	03/01/2020	H10.3	0	Conjuntivitis		1
2	03/01/2020	H00.03	4	Celulitis preseptal		1
1	03/01/2020	Alta por fuga	11	Alta por fuga		1
2	03/01/2020	H16.0	1	Úlcera corneal		1
1	03/01/2020	H10.81	0	Pingueculitis		1
2	03/01/2020	H16.0	1	Úlcera corneal		1
1	03/01/2020	T26	9	Causticación		1
1	03/01/2020	H16.9	1	Queratitis		1
2	03/01/2020	H10.3	0	Conjuntivitis		1
2	03/01/2020	H0.41	1	Ojo seco		1
1	03/01/2020	S05.9	9	Traumatismo		1
2	03/01/2020	Alta por fuga	11	Alta por fuga		1
1	03/01/2020	Alta por fuga	11	Alta por fuga		1
2	03/01/2020	H50.9	10	Estrabismo		1
2	03/01/2020	H18.20	1	Edema corneal		1
2	03/01/2020	H43.81	7	DVP		1
1	03/01/2020	H43.81	7	DVP		1
1	03/01/2020	H11.3	0	Hiposfagma		1
1	03/01/2020	T15.0	1	Cuerpo extraño		1
2	03/01/2020	H16.0	1	Úlcera corneal		1
2	03/01/2020	H43.81	7	DVP		1
2	03/01/2020	G43.9	10	Migraña		1
2	03/01/2020	H20	6	Uveitis		1
2	03/01/2020	H10.3	0	Conjuntivitis		1
2	03/01/2020	B00.1	4	Dermatitis herpética		1
1	03/01/2020	H33.0	8	Desprendimiento de retina		1
2	03/01/2020	H35.30	8	DMAE		1
1	03/01/2020	Alta por fuga	11	Alta por fuga		1
1	03/01/2020	S05.9	9	Traumatismo		1
2	03/01/2020	T26.4	9	Quemadura		1
1	03/01/2020	Alta por fuga	11	Alta por fuga		1
1	03/01/2020	Alta por fuga	11	Alta por fuga		1
1	03/01/2020	H16.9	1	Queratitis		1
2	03/01/2020	H10.3	0	Conjuntivitis		1
1	03/01/2020	H16.9	1	Queratitis		1
1	03/01/2020	S05.9	9	Traumatismo		1
2	03/01/2020	H16.9	1	Queratitis		1
1	03/01/2020	H11.3	0	Hiposfagma		1
1	03/01/2020	H01.00	4	Blefaritis		1
2	03/01/2020	H43.81	7	DVP		1
1	03/01/2020	S05.9	9	Traumatismo		1
2	03/01/2020	H16.9	1	Queratitis		1
1	03/01/2020	T15.0	1	Cuerpo extraño		1
1	03/01/2020	Alta por fuga	11	Alta por fuga		1
1	04/01/2020	H16.9	1	Queratitis		1
2	04/01/2020	H00.19	4	Chalazión		1
1	04/01/2020	H16.9	1	Queratitis		1
2	04/01/2020	H16.9	1	Queratitis		1
1	04/01/2020	H16.9	1	Queratitis		1

2	04/01/2020	H10.3	0	Conjuntivitis	1
1	04/01/2020	H20	6	Uveitis	1
2	04/01/2020	H10.3	0	Conjuntivitis	1
1	04/01/2020	H16.0	1	Úlcera corneal	1
1	04/01/2020	H00.02	4	Orzuelo	1
1	04/01/2020	H10.3	0	Conjuntivitis	1
1	04/01/2020	H10.3	0	Conjuntivitis	1
2	04/01/2020	H10.3	0	Conjuntivitis	1
2	04/01/2020	H10.3	0	Conjuntivitis	1
2	04/01/2020	H10.3	0	Conjuntivitis	1
1	04/01/2020	H16.0	1	Úlcera corneal	1
2	04/01/2020	H10.3	0	Conjuntivitis	1
2	04/01/2020	H16.9	1	Queratitis	1
2	04/01/2020	H16.9	1	Queratitis	1
2	04/01/2020	H11.3	0	Hiposfagma	1
1	04/01/2020	H40.9	3	Glaucoma	1
1	04/01/2020	H10.3	0	Conjuntivitis	1
1	04/01/2020	H00.02	4	Orzuelo	1
1	04/01/2020	H01.00	4	Blefaritis	1
1	04/01/2020	H35.3	8	Maculopatía	1
1	04/01/2020	H35.30	8	DMAE	1
2	04/01/2020	H10.3	0	Conjuntivitis	1
2	04/01/2020	H43.81	7	DVP	1
2	04/01/2020	H11.3	0	Hiposfagma	1
1	04/01/2020	H10.3	0	Conjuntivitis	1
2	04/01/2020	H10.3	0	Conjuntivitis	1
2	04/01/2020	H10.3	0	Conjuntivitis	1
2	04/01/2020	H16.9	1	Queratitis	1
2	04/01/2020	H11.3	0	Hiposfagma	1
2	04/01/2020	H11.3	0	Hiposfagma	1
2	04/01/2020	T26	9	Causticación	1
2	04/01/2020	H10.3	0	Conjuntivitis	1
2	04/01/2020	H11.3	0	Hiposfagma	1
2	04/01/2020	H11.3	0	Hiposfagma	1
2	04/01/2020	H16.0	1	Úlcera corneal	1
2	04/01/2020	H04.32	5	Dacriocistitis aguda	1
1	04/01/2020	H10.3	0	Conjuntivitis	1
1	04/01/2020	H33.0	8	Desprendimiento de retina	1
1	04/01/2020	G45.3	10	Amaurosis fugax	1
1	04/01/2020	H10.3	0	Conjuntivitis	1
2	05/01/2020	H11.3	0	Hiposfagma	1
1	05/01/2020	H16.0	1	Úlcera corneal	1
2	05/01/2020	H10.3	0	Conjuntivitis	1
1	05/01/2020	H10.3	0	Conjuntivitis	1
2	05/01/2020	H02	4	Alteración palpebral	1
2	05/01/2020	H0.41	1	ojo seco	1
1	05/01/2020	H16.9	1	Queratitis	1
2	05/01/2020	H10.3	0	Conjuntivitis	1
2	05/01/2020	H16.0	1	Úlcera corneal	1
1	05/01/2020	H16.9	1	Queratitis	1
2	05/01/2020	H10.3	0	Conjuntivitis	1
2	05/01/2020	H33.30	8	Desgarro retiniano	1
2	05/01/2020	H10.3	0	Conjuntivitis	1
2	05/01/2020	H15.1	6	Epiescleritis	1
1	05/01/2020	H26.9	2	Catarata	1
1	05/01/2020	H10.3	0	Conjuntivitis	1
1	05/01/2020	H00.02	4	Orzuelo	1
2	05/01/2020	H10.3	0	Conjuntivitis	1
1	05/01/2020	H16.9	1	Queratitis	1
1	05/01/2020	H10.3	0	Conjuntivitis	1
2	05/01/2020	H40.5	3	Glaucoma secundario	1
2	05/01/2020	H16.0	1	Úlcera corneal	1
2	05/01/2020	H10.3	0	Conjuntivitis	1
2	05/01/2020	H43.81	7	DVP	1
1	05/01/2020	G43.9	10	Migraña	1
2	05/01/2020	H16.9	1	Queratitis	1

2	05/01/2020	H16.0	1	Úlcera corneal	1
2	05/01/2020	H10.3	0	Conjuntivitis	1
2	05/01/2020	H10.3	0	Conjuntivitis	1
1	05/01/2020	H16.0	1	Úlcera corneal	1
2	05/01/2020	H16.9	1	Queratitis	1
1	05/01/2020	H00.02	4	Orzuelo	1
2	05/01/2020	H16.9	1	Queratitis	1
2	05/01/2020	H00.02	4	Orzuelo	1
1	05/01/2020	Alta por fuga	11	Alta por fuga	1
2	05/01/2020	S05.00	9	Abrasión conjuntival	1
2	06/01/2020	H01.00	4	Blefaritis	1
2	06/01/2020	H16.0	1	Úlcera corneal	1
1	06/01/2020	T15.0	1	Cuerpo extraño	1
2	06/01/2020	H00.02	4	Orzuelo	1
1	06/01/2020	H43.81	7	DVP	1
2	06/01/2020	H16.0	1	Úlcera corneal	1
1	06/01/2020	H10.3	0	Conjuntivitis	1
2	06/01/2020	T15.0	1	Cuerpo extraño	1
1	06/01/2020	H11.3	0	Hiposfagma	1
2	06/01/2020	H01.00	4	Blefaritis	1
2	06/01/2020	H43.81	7	DVP	1
2	06/01/2020	H15.00	6	Escleritis	1
2	06/01/2020	H16.9	1	Queratitis	1
2	06/01/2020	H00.02	4	Orzuelo	1
1	06/01/2020	H11.00	0	Pterigium	1
2	06/01/2020	G43.9	10	Migraña	1
2	06/01/2020	H16.9	1	Queratitis	1
1	06/01/2020	H10.5	0	Blefaroconjuntivitis	1
2	06/01/2020	H11.3	0	Hiposfagma	1
2	06/01/2020	H43.81	7	DVP	1
1	06/01/2020	H16.0	1	Úlcera corneal	1
1	06/01/2020	H16.0	1	Úlcera corneal	1
1	06/01/2020	H16.0	1	Úlcera corneal	1
1	06/01/2020	H10.3	0	Conjuntivitis	1
2	06/01/2020	H11.3	0	Hiposfagma	1
1	06/01/2020	H11.3	0	Hiposfagma	1
1	06/01/2020	H02	4	Alteración palpebral	1
2	07/01/2020	H16.9	1	Queratitis	1
2	07/01/2020	H10.3	0	Conjuntivitis	1
2	07/01/2020	H16.9	1	Queratitis	1
2	07/01/2020	H10.5	0	Blefaroconjuntivitis	1
2	07/01/2020	H16.0	1	Úlcera corneal	1
2	07/01/2020	H16.9	1	Queratitis	1
2	07/01/2020	H10.3	0	Conjuntivitis	1
2	07/01/2020	H00.02	4	Orzuelo	1
2	07/01/2020	H16.9	1	Queratitis	1
1	07/01/2020	H11.3	0	Hiposfagma	1
2	07/01/2020	H10.3	0	Conjuntivitis	1
1	07/01/2020	H43.81	7	DVP	1
2	07/01/2020	H10.3	0	Conjuntivitis	1
1	07/01/2020	H10.3	0	Conjuntivitis	1
2	07/01/2020	H10.3	0	Conjuntivitis	1
2	07/01/2020	H10.3	0	Conjuntivitis	1
2	07/01/2020	H11.3	0	Hiposfagma	1
2	07/01/2020	H10.3	0	Conjuntivitis	1
2	07/01/2020	H01.00	4	Blefaritis	1
1	07/01/2020	H16.9	1	Queratitis	1
1	07/01/2020	H11.3	0	Hiposfagma	1
2	07/01/2020	H10.3	0	Conjuntivitis	1
2	07/01/2020	H00.19	4	Chalazión	1
2	07/01/2020	H43.81	7	DVP	1
1	07/01/2020	H16.9	1	Queratitis	1
2	07/01/2020	H16.0	1	Úlcera corneal	1
1	07/01/2020	H26.9	2	Catarata	1
2	07/01/2020	H11.3	0	Hiposfagma	1
2	07/01/2020	H16.9	1	Queratitis	1

2	07/01/2020	H10.3	0	Conjuntivitis	1
2	07/01/2020	H10.3	0	Conjuntivitis	1
2	07/01/2020	H15.1	6	Epiescleritis	1
2	07/01/2020	H46	10	Neuritis óptica	1
2	07/01/2020	H16.9	1	Queratitis	1
2	07/01/2020	Z98.4	2	Post op catarata	1
2	07/01/2020	H10.3	0	Conjuntivitis	1
2	07/01/2020	H04.32	5	Dacriocistitis aguda	1
2	07/01/2020	H10.3	0	Conjuntivitis	1
1	07/01/2020	Z98.4	2	Post op catarata	1
1	07/01/2020	H10.3	0	Conjuntivitis	1
2	07/01/2020	H16.9	1	Queratitis	1
1	07/01/2020	H18.50	1	Distrofia corneal	1
1	07/01/2020	H10.3	0	Conjuntivitis	1
2	07/01/2020	H50.9	10	Estrabismo	1
2	07/01/2020	S05.9	9	Traumatismo	1
2	07/01/2020	H10.3	0	Conjuntivitis	1
1	07/01/2020	H27.8	2	OCP	1
2	07/01/2020	H15.1	6	Epiescleritis	1
1	07/01/2020	H16.9	1	Queratitis	1
2	07/01/2020	T15.0	1	Cuerpo extraño	1
2	07/01/2020	H16.0	1	Úlcera corneal	1
1	07/01/2020	H16.0	1	Úlcera corneal	1
1	07/01/2020	T15.0	1	Cuerpo extraño	1
2	07/01/2020	H00.02	4	Orzuelo	1
2	07/01/2020	H16.9	1	Queratitis	1
2	07/01/2020	H11.3	0	Hiposfagma	1
1	07/01/2020	H10.3	0	Conjuntivitis	1
1	07/01/2020	H16.0	1	Úlcera corneal	1
2	07/01/2020	H10.3	0	Conjuntivitis	1
2	08/01/2020	H20	6	Uveitis	1
2	08/01/2020	H01.00	4	Blefaritis	1
1	08/01/2020	H10.3	0	Conjuntivitis	1
2	08/01/2020	H43.81	7	DVP	1
2	08/01/2020	H10.3	0	Conjuntivitis	1
2	08/01/2020	H43.81	7	DVP	1
2	08/01/2020	H16.9	1	Queratitis	1
1	08/01/2020	H43.81	7	DVP	1
1	08/01/2020	H43.81	7	DVP	1
1	08/01/2020	H16.9	1	Queratitis	1
2	08/01/2020	S05.9	9	Traumatismo	1
1	08/01/2020	H02	4	Alteración palpebral	1
2	08/01/2020	H00.03	4	Celulitis preseptal	1
2	08/01/2020	H16.9	1	Queratitis	1
1	08/01/2020	H10.3	0	Conjuntivitis	1
1	08/01/2020	H35.30	8	DMAE	1
2	08/01/2020	H16.9	1	Queratitis	1
1	08/01/2020	H43.81	7	DVP	1
2	08/01/2020	H16.9	1	Queratitis	1
2	08/01/2020	H10.3	0	Conjuntivitis	1
2	08/01/2020	H33.30	8	Desgarro retiniano	1
1	08/01/2020	H10.3	0	Conjuntivitis	1
2	08/01/2020	H11.3	0	Hiposfagma	1
1	08/01/2020	H11.3	0	Hiposfagma	1
1	08/01/2020	H02	4	Alteración palpebral	1
2	08/01/2020	H35.30	8	DMAE	1
2	08/01/2020	H10.3	0	Conjuntivitis	1
1	08/01/2020	H10.3	0	Conjuntivitis	1
2	08/01/2020	H00.02	4	Orzuelo	1
2	08/01/2020	H53.10	11	Problema refractivo	1
1	08/01/2020	H43.81	7	DVP	1
2	08/01/2020	H16.9	1	Queratitis	1
2	08/01/2020	H43.81	7	DVP	1
1	08/01/2020	H43.1	7	Hemovítreo	1
2	08/01/2020	H10.3	0	Conjuntivitis	1
2	08/01/2020	H35.3	8	Maculopatía	1

1	09/01/2020	H16.0	1	Úlcera corneal	1
1	09/01/2020	S05.9	9	Traumatismo	1
2	09/01/2020	H00.02	4	Orzuelo	1
1	09/01/2020	H16.0	1	Úlcera corneal	1
2	09/01/2020	Z98.4	2	Post op catarata	1
2	09/01/2020	H35.30	8	DMAE	1
2	09/01/2020	B00.1	4	Dermatitis herpética	1
1	09/01/2020	H20	6	Uveitis	1
2	09/01/2020	H00.02	4	Orzuelo	1
2	09/01/2020	H01.00	4	Blefaritis	1
1	09/01/2020	H50.9	10	Estrabismo	1
1	09/01/2020	H26.9	2	Catarata	1
1	09/01/2020	H33.0	8	Desprendimiento de retina	1
1	09/01/2020	H43.81	7	DVP	1
1	09/01/2020	H26.9	2	Catarata	1
1	09/01/2020	H02	4	Alteración palpebral	1
1	09/01/2020	H43.81	7	DVP	1
2	09/01/2020	H01.00	4	Blefaritis	1
2	09/01/2020	H10.3	0	Conjuntivitis	1
2	09/01/2020	H34.82	8	Trombosis venosa	1
2	09/01/2020	H10.5	0	Blefaroconjuntivitis	1
1	09/01/2020	S05.9	9	Traumatismo	1
1	09/01/2020	H16.9	1	Queratitis	1
2	09/01/2020	H35.30	8	DMAE	1
2	09/01/2020	H16.9	1	Queratitis	1
1	09/01/2020	H34.82	8	Trombosis venosa	1
1	09/01/2020	H16.9	1	Queratitis	1
2	09/01/2020	H43.1	7	Hemovítreo	1
1	09/01/2020	H35.3	8	Maculopatía	1
2	09/01/2020	H10.3	0	Conjuntivitis	1
2	09/01/2020	H10.3	0	Conjuntivitis	1
1	09/01/2020	E11.319	8	Retinopatía diabética	1
1	09/01/2020	H20	6	Uveitis	1
1	09/01/2020	H10.3	0	Conjuntivitis	1
1	09/01/2020	H00.02	4	Orzuelo	1
1	09/01/2020	G43.9	10	Migraña	1
1	09/01/2020	H16.9	1	Queratitis	1
2	09/01/2020	H40.05	3	HTO	1
2	09/01/2020	H46	10	Neuritis óptica	1
1	09/01/2020	H35.30	8	DMAE	1
2	09/01/2020	H10.3	0	Conjuntivitis	1
2	09/01/2020	Alta por fuga	11	Alta por fuga	1
2	09/01/2020	H16.0	1	Úlcera corneal	1
2	09/01/2020	H10.3	0	Conjuntivitis	1
1	09/01/2020	H10.3	0	Conjuntivitis	1
2	09/01/2020	H15.1	6	Epiescleritis	1
1	09/01/2020	T15.0	1	Cuerpo extraño	1
1	09/01/2020	H16.9	1	Queratitis	1
1	09/01/2020	H33.0	8	Desprendimiento de retina	1
1	09/01/2020	Alta por fuga	11	Alta por fuga	1
1	09/01/2020	H02.05	4	Distiquiasis	1
2	09/01/2020	H16.9	1	Queratitis	1
1	10/01/2020	H10.3	0	Conjuntivitis	1
1	10/01/2020	T15.0	1	Cuerpo extraño	1
2	10/01/2020	H01.00	4	Blefaritis	1
2	10/01/2020	H11.3	0	Hiposfagma	1
2	10/01/2020	H16.9	1	Queratitis	1
2	10/01/2020	T15.0	1	Cuerpo extraño	1
2	10/01/2020	H16.9	1	Queratitis	1
2	10/01/2020	G43.9	10	Migraña	1
2	10/01/2020	H34.82	8	Trombosis venosa	1
2	10/01/2020	H11.3	0	Hiposfagma	1
1	10/01/2020	H00.02	4	Orzuelo	1
2	10/01/2020	H02	4	Alteración palpebral	1
2	10/01/2020	H20	6	Uveitis	1
2	10/01/2020	H43.81	7	DVP	1

1	10/01/2020	H10.3	0	Conjuntivitis	1
1	10/01/2020	H10.3	0	Conjuntivitis	1
2	10/01/2020	H02	4	Alteración palpebral	1
1	10/01/2020	H16.0	1	Úlcera corneal	1
1	10/01/2020	H20	6	Uveitis	1
2	10/01/2020	H00.02	4	Orzuelo	1
1	10/01/2020	H16.9	1	Queratitis	1
2	10/01/2020	H16.0	1	Úlcera corneal	1
2	10/01/2020	H16.9	1	Queratitis	1
2	10/01/2020	H10.3	0	Conjuntivitis	1
2	10/01/2020	Alta por fuga	11	Alta por fuga	1
2	10/01/2020	H16.9	1	Queratitis	1
1	10/01/2020	H16.9	1	Queratitis	1
2	10/01/2020	B00.1	4	Dermatitis herpética	1
1	10/01/2020	T15.0	1	Cuerpo extraño	1
1	10/01/2020	H16.9	1	Queratitis	1
2	10/01/2020	H16.9	1	Queratitis	1
1	10/01/2020	H10.3	0	Conjuntivitis	1
2	10/01/2020	H43.81	7	DVP	1
1	10/01/2020	H20	6	Uveitis	1
1	10/01/2020	H50.9	10	Estrabismo	1
2	10/01/2020	S05.00	9	Abrasión conjuntival	1
1	10/01/2020	S05.9	9	Traumatismo	1
1	10/01/2020	H02	4	Alteración palpebral	1
2	10/01/2020	H16.0	1	Úlcera corneal	1
2	10/01/2020	H16.9	1	Queratitis	1
1	10/01/2020	H00.02	4	Orzuelo	1
2	10/01/2020	H10.3	0	Conjuntivitis	1
2	10/01/2020	S05.9	9	Traumatismo	1
1	10/01/2020	H16.0	1	Úlcera corneal	1
1	10/01/2020	H01.00	4	Blefaritis	1
2	10/01/2020	H16.9	1	Queratitis	1
1	10/01/2020	H16.0	1	Úlcera corneal	1
1	10/01/2020	H16.0	1	Úlcera corneal	1
1	10/01/2020	H16.0	1	Úlcera corneal	1
2	10/01/2020	G43.9	10	Migraña	1
1	10/01/2020	H10.3	0	Conjuntivitis	1
2	10/01/2020	H26.9	2	Catarata	1
1	10/01/2020	S05.00	9	Abrasión conjuntival	1
2	10/01/2020	H10.3	0	Conjuntivitis	1
1	11/01/2020	H10.3	0	Conjuntivitis	1
1	11/01/2020	H16.9	1	Queratitis	1
2	11/01/2020	T15.0	1	Cuerpo extraño	1
1	11/01/2020	T15.0	1	Cuerpo extraño	1
1	11/01/2020	H43.81	7	DVP	1
1	11/01/2020	H43.81	7	DVP	1
2	11/01/2020	H16.9	1	Queratitis	1
1	11/01/2020	H50.9	10	Estrabismo	1
1	11/01/2020	H16.0	1	Úlcera corneal	1
2	11/01/2020	H10.3	0	Conjuntivitis	1
1	11/01/2020	H16.9	1	Queratitis	1
2	11/01/2020	S05.9	9	Traumatismo	1
1	11/01/2020	H20	6	Uveitis	1
1	11/01/2020	H10.3	0	Conjuntivitis	1
2	11/01/2020	H16.0	1	Úlcera corneal	1
1	11/01/2020	S05.9	9	Traumatismo	1
1	11/01/2020	H16.0	1	Úlcera corneal	1
2	11/01/2020	H04.32	5	Dacriocistitis aguda	1
2	11/01/2020	H11.3	0	Hiposfagma	1
2	11/01/2020	H16.0	1	Úlcera corneal	1
1	11/01/2020	H10.3	0	Conjuntivitis	1
1	11/01/2020	H00.02	4	Orzuelo	1
1	11/01/2020	H40.05	3	HTO	1
1	11/01/2020	T15.0	1	Cuerpo extraño	1
2	11/01/2020	H43.81	7	DVP	1
2	11/01/2020	H16.9	1	Queratitis	1

2	11/01/2020	H10.3	0	Conjuntivitis	1
2	11/01/2020	H10.3	0	Conjuntivitis	1
1	11/01/2020	T26.4	9	Quemadura	1
1	11/01/2020	Alta por fuga	11	Alta por fuga	1
1	11/01/2020	H16.0	1	Úlcera corneal	1
2	11/01/2020	H16.9	1	Queratitis	1
2	11/01/2020	H16.9	1	Queratitis	1
2	11/01/2020	H11.3	0	Hiposfagma	1
1	11/01/2020	H16.9	1	Queratitis	1
1	11/01/2020	H16.0	1	Úlcera corneal	1
2	11/01/2020	H0.41	1	ojo seco	1
1	11/01/2020	H01.00	4	Blefaritis	1
1	11/01/2020	H16.9	1	Queratitis	1
2	11/01/2020	H16.0	1	Úlcera corneal	1
1	11/01/2020	S05.9	9	Traumatismo	1
1	11/01/2020	H16.9	1	Queratitis	1
1	11/01/2020	H16.9	1	Queratitis	1
1	11/01/2020	H10.3	0	Conjuntivitis	1
2	11/01/2020	H20	6	Uveitis	1
1	11/01/2020	H10.3	0	Conjuntivitis	1
1	12/01/2020	H20	6	Uveitis	1
2	12/01/2020	H16.0	1	Úlcera corneal	1
2	12/01/2020	T26	9	Causticación	1
1	12/01/2020	H00.02	4	Orzuelo	1
2	12/01/2020	H01.00	4	Blefaritis	1
2	12/01/2020	T15.0	1	Cuerpo extraño	1
1	12/01/2020	H16.9	1	Queratitis	1
2	12/01/2020	H10.3	0	Conjuntivitis	1
2	12/01/2020	H10.5	0	Blefaroconjuntivitis	1
1	12/01/2020	H43.81	7	DVP	1
1	12/01/2020	H10.3	0	Conjuntivitis	1
2	12/01/2020	H16.0	1	Úlcera corneal	1
2	12/01/2020	H00.02	4	Orzuelo	1
2	12/01/2020	H01.00	4	Blefaritis	1
2	12/01/2020	T15.0	1	Cuerpo extraño	1
2	12/01/2020	H43.81	7	DVP	1
2	12/01/2020	H15.1	6	Epiescleritis	1
2	12/01/2020	H15.1	6	Epiescleritis	1
1	12/01/2020	H33.0	8	Desprendimiento de retina	1
1	12/01/2020	H16.9	1	Queratitis	1
2	12/01/2020	H01.00	4	Blefaritis	1
1	12/01/2020	H20	6	Uveitis	1
1	12/01/2020	T15.0	1	Cuerpo extraño	1
1	12/01/2020	H15.1	6	Epiescleritis	1
2	12/01/2020	H10.3	0	Conjuntivitis	1
2	12/01/2020	S05.9	9	Traumatismo	1
2	12/01/2020	H46	10	Neuritis óptica	1
1	12/01/2020	H20	6	Uveitis	1
1	12/01/2020	S05.9	9	Traumatismo	1
1	12/01/2020	H11.3	0	Hiposfagma	1
1	12/01/2020	S05.9	9	Traumatismo	1
2	12/01/2020	H16.9	1	Queratitis	1
1	12/01/2020	H16.9	1	Queratitis	1
1	12/01/2020	H10.3	0	Conjuntivitis	1
1	12/01/2020	H16.0	1	Úlcera corneal	1
1	12/01/2020	S05.30	9	Laceración conjuntival	1
2	12/01/2020	H43.81	7	DVP	1
2	12/01/2020	H43.81	7	DVP	1
2	12/01/2020	H10.3	0	Conjuntivitis	1
1	12/01/2020	H16.3	1	Abceso corneal	1
1	12/01/2020	S05.9	9	Traumatismo	1
2	12/01/2020	H10.81	0	Pingueculitis	1
2	12/01/2020	H10.3	0	Conjuntivitis	1
1	12/01/2020	H01.00	4	Blefaritis	1
1	12/01/2020	H40.9	3	Glaucoma	1
1	12/01/2020	H16.0	1	Úlcera corneal	1

1	12/01/2020	T15.0	1	Cuerpo extraño	1
2	12/01/2020	H16.0	1	Úlcera corneal	1
1	12/01/2020	H16.0	1	Úlcera corneal	1
2	12/01/2020	H0.41	1	Ojo seco	1
2	12/01/2020	H04.32	5	Dacriocistitis aguda	1
1	12/01/2020	T15.0	1	Cuerpo extraño	1
1	13/01/2020	H16.0	1	Úlcera corneal	1
1	13/01/2020	H16.9	1	Queratitis	1
2	13/01/2020	H16.9	1	Queratitis	1
1	13/01/2020	H16.0	1	Úlcera corneal	1
1	13/01/2020	H16.0	1	Úlcera corneal	1
2	13/01/2020	H11.3	0	Hiposfagma	1
1	13/01/2020	H43.81	7	DVP	1
2	13/01/2020	H0.41	1	Ojo seco	1
1	13/01/2020	H50.9	10	Estrabismo	1
2	13/01/2020	H00.02	4	Orzuelo	1
1	13/01/2020	H33.30	8	Desgarro retiniano	1
2	13/01/2020	H26.9	2	Catarata	1
2	13/01/2020	H10.3	0	Conjuntivitis	1
1	13/01/2020	H10.3	0	Conjuntivitis	1
2	13/01/2020	H35.3	8	Maculopatía	1
2	13/01/2020	H01.00	4	Blefaritis	1
2	13/01/2020	H02	4	Alteración palpebral	1
2	13/01/2020	H01.00	4	Blefaritis	1
2	13/01/2020	H43.81	7	DVP	1
2	13/01/2020	H16.9	1	Queratitis	1
2	13/01/2020	G43.9	10	Migraña	1
2	13/01/2020	H02	4	Alteración palpebral	1
2	13/01/2020	H10.3	0	Conjuntivitis	1
2	13/01/2020	H01.00	4	Blefaritis	1
2	13/01/2020	H11.3	0	Hiposfagma	1
2	13/01/2020	H16.9	1	Queratitis	1
2	13/01/2020	H26.9	2	Catarata	1
1	13/01/2020	H40.05	3	HTO	1
2	13/01/2020	H00.02	4	Orzuelo	1
2	13/01/2020	S05.9	9	Traumatismo	1
1	13/01/2020	T15.0	1	Cuerpo extraño	1
2	13/01/2020	H16.9	1	Queratitis	1
1	13/01/2020	H10.3	0	Conjuntivitis	1
2	13/01/2020	H0.41	1	Ojo seco	1
2	13/01/2020	S05.9	9	Traumatismo	1
1	13/01/2020	H43.81	7	DVP	1
1	13/01/2020	T15.0	1	Cuerpo extraño	1
1	13/01/2020	H10.3	0	Conjuntivitis	1
1	13/01/2020	H43.81	7	DVP	1
1	13/01/2020	H16.0	1	Úlcera corneal	1
1	13/01/2020	H10.3	0	Conjuntivitis	1
2	13/01/2020	S05.9	9	Traumatismo	1
2	13/01/2020	H10.3	0	Conjuntivitis	1
1	13/01/2020	H16.9	1	Queratitis	1
2	13/01/2020	H16.9	1	Queratitis	1
2	13/01/2020	S05.9	9	Traumatismo	1
1	13/01/2020	E11.319	8	Retinopatía diabética	1
1	13/01/2020	H11.3	0	Hiposfagma	1
1	13/01/2020	S05.9	9	Traumatismo	1
1	13/01/2020	H10.3	0	Conjuntivitis	1
2	13/01/2020	S05.9	9	Traumatismo	1
1	13/01/2020	H10.3	0	Conjuntivitis	1
2	13/01/2020	H16.9	1	Queratitis	1
2	13/01/2020	H16.9	1	Queratitis	1
2	14/01/2020	H10.3	0	Conjuntivitis	1
2	14/01/2020	H10.3	0	Conjuntivitis	1
2	14/01/2020	H10.3	0	Conjuntivitis	1
2	14/01/2020	H01.00	4	Blefaritis	1
1	14/01/2020	H10.3	0	Conjuntivitis	1
2	14/01/2020	H00.02	4	Orzuelo	1

2	14/01/2020	H10.3	0	Conjuntivitis	1
2	14/01/2020	H16.9	1	Queratitis	1
1	14/01/2020	H16.9	1	Queratitis	1
2	14/01/2020	H10.81	0	Pingueculitis	1
1	14/01/2020	H16.0	1	Úlcera corneal	1
1	14/01/2020	H43.81	7	DVP	1
2	14/01/2020	H35.30	8	DMAE	1
1	14/01/2020	H20	6	Uveitis	1
2	14/01/2020	H43.81	7	DVP	1
2	14/01/2020	H01.00	4	Blefaritis	1
1	14/01/2020	H35.30	8	DMAE	1
1	14/01/2020	H16.9	1	Queratitis	1
2	14/01/2020	H43.81	7	DVP	1
2	14/01/2020	H35.30	8	DMAE	1
2	14/01/2020	H00.02	4	Orzuelo	1
1	14/01/2020	H16.9	1	Queratitis	1
2	14/01/2020	S05.9	9	Traumatismo	1
1	14/01/2020	H10.3	0	Conjuntivitis	1
1	14/01/2020	S05.9	9	Traumatismo	1
1	14/01/2020	H43.81	7	DVP	1
1	14/01/2020	H10.3	0	Conjuntivitis	1
1	14/01/2020	H10.3	0	Conjuntivitis	1
1	14/01/2020	T15.0	1	Cuerpo extraño	1
2	14/01/2020	H43.81	7	DVP	1
1	14/01/2020	H16.0	1	Úlcera corneal	1
2	14/01/2020	H10.3	0	Conjuntivitis	1
1	14/01/2020	H40.05	3	HTO	1
2	14/01/2020	H16.9	1	Queratitis	1
1	14/01/2020	H16.0	1	Úlcera corneal	1
1	14/01/2020	H10.3	0	Conjuntivitis	1
2	14/01/2020	H10.3	0	Conjuntivitis	1
1	15/01/2020	H43.81	7	DVP	1
1	15/01/2020	S05.9	9	Traumatismo	1
2	15/01/2020	H10.3	0	Conjuntivitis	1
2	15/01/2020	H10.3	0	Conjuntivitis	1
1	15/01/2020	H16.9	1	Queratitis	1
1	15/01/2020	H27.8	2	OCP	1
2	15/01/2020	T15.0	1	Cuerpo extraño	1
2	15/01/2020	H10.5	0	Blefaroconjuntivitis	1
2	15/01/2020	H11.3	0	Hiposfagma	1
1	15/01/2020	B00.1	4	Dermatitis herpética	1
2	15/01/2020	H10.3	0	Conjuntivitis	1
1	15/01/2020	H43.81	7	DVP	1
2	15/01/2020	H26.9	2	Catarata	1
2	15/01/2020	S05.9	9	Traumatismo	1
2	15/01/2020	H02	4	Alteración palpebral	1
1	15/01/2020	S05.9	9	Traumatismo	1
2	15/01/2020	H00.02	4	Orzuelo	1
1	15/01/2020	S05.9	9	Traumatismo	1
2	15/01/2020	H43.81	7	DVP	1
2	15/01/2020	S05.9	9	Traumatismo	1
2	15/01/2020	H16.9	1	Queratitis	1
2	15/01/2020	H10.3	0	Conjuntivitis	1
2	15/01/2020	H01.00	4	Blefaritis	1
1	15/01/2020	H16.9	1	Queratitis	1
1	15/01/2020	H10.3	0	Conjuntivitis	1
1	15/01/2020	T15.0	1	Cuerpo extraño	1
2	15/01/2020	T15.0	1	Cuerpo extraño	1
2	15/01/2020	H16.0	1	Úlcera corneal	1
2	15/01/2020	G43.109	10	Aura	1
1	15/01/2020	H43.81	7	DVP	1
2	15/01/2020	H10.3	0	Conjuntivitis	1
2	15/01/2020	H33.0	8	Desprendimiento de retina	1
1	15/01/2020	H00.02	4	Orzuelo	1
1	15/01/2020	H43.81	7	DVP	1
2	15/01/2020	H43.81	7	DVP	1

2	15/01/2020	H00.02	4	Orzuelo	1
1	15/01/2020	T15.0	1	Cuerpo extraño	1
2	15/01/2020	H01.00	4	Blefaritis	1
2	15/01/2020	H00.02	4	Orzuelo	1
1	15/01/2020	T15.0	1	Cuerpo extraño	1
2	15/01/2020	H16.0	1	Úlcera corneal	1
1	15/01/2020	H01.00	4	Blefaritis	1
2	15/01/2020	S05.9	9	Traumatismo	1
2	15/01/2020	H16.0	1	Úlcera corneal	1
2	15/01/2020	H16.9	1	Queratitis	1
2	15/01/2020	H10.3	0	Conjuntivitis	1
2	15/01/2020	H16.9	1	Queratitis	1
1	15/01/2020	T15.0	1	Cuerpo extraño	1
2	16/01/2020	H0.41	1	Ojo seco	1
2	16/01/2020	H10.3	0	Conjuntivitis	1
1	16/01/2020	H26.9	2	Catarata	1
2	16/01/2020	H16.0	1	Úlcera corneal	1
2	16/01/2020	H20	6	Uveitis	1
1	16/01/2020	H16.9	1	Queratitis	1
2	16/01/2020	H16.9	1	Queratitis	1
1	16/01/2020	H11.3	0	Hiposfagma	1
2	16/01/2020	H43.81	7	DVP	1
2	16/01/2020	H16.9	1	Queratitis	1
2	16/01/2020	H01.00	4	Blefaritis	1
2	16/01/2020	H11.3	0	Hiposfagma	1
2	16/01/2020	H10.3	0	Conjuntivitis	1
2	16/01/2020	H10.3	0	Conjuntivitis	1
2	16/01/2020	S05.9	9	Traumatismo	1
2	16/01/2020	H10.3	0	Conjuntivitis	1
2	16/01/2020	H10.3	0	Conjuntivitis	1
2	16/01/2020	H11.3	0	Hiposfagma	1
1	16/01/2020	H43.81	7	DVP	1
1	16/01/2020	H10.3	0	Conjuntivitis	1
2	16/01/2020	H43.81	7	DVP	1
1	16/01/2020	H10.3	0	Conjuntivitis	1
2	16/01/2020	H26.9	2	Catarata	1
1	16/01/2020	H16.9	1	Queratitis	1
2	16/01/2020	H10.3	0	Conjuntivitis	1
2	16/01/2020	H53.9	11	Alteraciones visuales	1
1	16/01/2020	H43.81	7	DVP	1
2	16/01/2020	H10.3	0	Conjuntivitis	1
2	16/01/2020	H10.3	0	Conjuntivitis	1
1	16/01/2020	H10.3	0	Conjuntivitis	1
2	16/01/2020	H50.9	10	Estrabismo	1
1	16/01/2020	H10.3	0	Conjuntivitis	1
2	16/01/2020	T26	9	Causticación	1
1	16/01/2020	S05.9	9	Traumatismo	1
2	16/01/2020	S05.9	9	Traumatismo	1
1	16/01/2020	H11.3	0	Hiposfagma	1
2	16/01/2020	G43.9	10	Migraña	1
1	16/01/2020	T15.0	1	Cuerpo extraño	1
1	16/01/2020	H11.3	0	Hiposfagma	1
1	16/01/2020	H10.3	0	Conjuntivitis	1
2	16/01/2020	H02	4	Alteración palpebral	1
2	17/01/2020	H04.32	5	Dacriocistitis aguda	1
1	17/01/2020	H16.9	1	Queratitis	1
2	17/01/2020	H16.9	1	Queratitis	1
2	17/01/2020	H30.9	6	Uveitis posterior	1
1	17/01/2020	H16.9	1	Queratitis	1
2	17/01/2020	H01.00	4	Blefaritis	1
1	17/01/2020	H20	6	Uveitis	1
1	17/01/2020	H10.3	0	Conjuntivitis	1
2	17/01/2020	H35.30	8	DMAE	1
2	17/01/2020	H16.9	1	Queratitis	1
2	17/01/2020	H20	6	Uveitis	1
2	17/01/2020	H01.00	4	Blefaritis	1

2	17/01/2020	H02	4	Alteración palpebral	1
1	17/01/2020	H10.3	0	Conjuntivitis	1
2	17/01/2020	H35.3	8	Maculopatía	1
1	17/01/2020	S05.9	9	Traumatismo	1
2	17/01/2020	H01.00	4	Blefaritis	1
1	17/01/2020	H35.30	8	DMAE	1
1	17/01/2020	H10.3	0	Conjuntivitis	1
2	17/01/2020	H10.3	0	Conjuntivitis	1
1	17/01/2020	H53	11	No patología	1
2	17/01/2020	H11.3	0	Hiposfagma	1
2	17/01/2020	H10.3	0	Conjuntivitis	1
2	17/01/2020	H16.0	1	Úlcera corneal	1
2	17/01/2020	H20	6	Uveitis	1
2	17/01/2020	H35.3	8	Maculopatía	1
1	17/01/2020	H16.9	1	Queratitis	1
2	17/01/2020	H00.02	4	Orzuelo	1
2	17/01/2020	H46	10	Neuritis óptica	1
1	17/01/2020	H10.3	0	Conjuntivitis	1
2	17/01/2020	H10.3	0	Conjuntivitis	1
1	17/01/2020	H16.0	1	Úlcera corneal	1
1	17/01/2020	S05.9	9	Traumatismo	1
2	17/01/2020	G43.9	10	Migraña	1
1	17/01/2020	H16.9	1	Queratitis	1
2	17/01/2020	H00.02	4	Orzuelo	1
2	17/01/2020	H10.3	0	Conjuntivitis	1
2	17/01/2020	H11.3	0	Hiposfagma	1
1	17/01/2020	H15.00	6	Escleritis	1
2	17/01/2020	H17.8	1	Infiltrados corneales	1
1	17/01/2020	T15.0	1	Cuerpo extraño	1
1	17/01/2020	H27.10	2	Luxación LIO	1
1	17/01/2020	T15.0	1	Cuerpo extraño	1
2	17/01/2020	H01.00	4	Blefaritis	1
2	17/01/2020	S05.9	9	Traumatismo	1
2	17/01/2020	S05.9	9	Traumatismo	1
2	17/01/2020	S05.9	9	Traumatismo	1
2	17/01/2020	H16.0	1	Úlcera corneal	1
1	18/01/2020	H11.44	0	Quiste conjuntival	1
1	18/01/2020	H16.9	1	Queratitis	1
2	18/01/2020	H16.0	1	Úlcera corneal	1
2	18/01/2020	H16.9	1	Queratitis	1
2	18/01/2020	H11.3	0	Hiposfagma	1
1	18/01/2020	H16.9	1	Queratitis	1
2	18/01/2020	H16.9	1	Queratitis	1
2	18/01/2020	H16.9	1	Queratitis	1
2	18/01/2020	H16.9	1	Queratitis	1
1	18/01/2020	H16.9	1	Queratitis	1
1	18/01/2020	Z98.4	2	Post op catarata	1
2	18/01/2020	H10.3	0	Conjuntivitis	1
2	18/01/2020	H46	10	Neuritis óptica	1
2	18/01/2020	H10.3	0	Conjuntivitis	1
2	18/01/2020	H00.02	4	Orzuelo	1
1	18/01/2020	H10.3	0	Conjuntivitis	1
2	18/01/2020	H10.3	0	Conjuntivitis	1
1	18/01/2020	H11.3	0	Hiposfagma	1
2	18/01/2020	H02	4	Alteración palpebral	1
2	18/01/2020	H16.9	1	Queratitis	1
2	18/01/2020	H02	4	Alteración palpebral	1
2	18/01/2020	H04.32	5	Dacriocistitis aguda	1
2	18/01/2020	H16.9	1	Queratitis	1
1	18/01/2020	H16.9	1	Queratitis	1
1	18/01/2020	H20	6	Uveitis	1
2	18/01/2020	H00.02	4	Orzuelo	1
1	18/01/2020	H11.3	0	Hiposfagma	1
1	18/01/2020	T15.0	1	Cuerpo extraño	1
1	18/01/2020	H16.0	1	Úlcera corneal	1
2	18/01/2020	H10.3	0	Conjuntivitis	1

1	18/01/2020	H33.30	8	Desgarro retiniano	1
2	18/01/2020	H10.3	0	Conjuntivitis	1
1	18/01/2020	H33.30	8	Desgarro retiniano	1
2	18/01/2020	H20	6	Uveitis	1
2	18/01/2020	H40.05	3	HTO	1
2	18/01/2020	S05.9	9	Traumatismo	1
2	18/01/2020	H10.3	0	Conjuntivitis	1
2	18/01/2020	H10.3	0	Conjuntivitis	1
1	18/01/2020	H00.02	4	Orzuelo	1
1	18/01/2020	H11.3	0	Hiposfagma	1
2	18/01/2020	H00.02	4	Orzuelo	1
1	18/01/2020	H16.0	1	Úlcera corneal	1
2	18/01/2020	H00.02	4	Orzuelo	1
1	18/01/2020	H11.3	0	Hiposfagma	1
1	18/01/2020	H43.81	7	DVP	1
2	18/01/2020	H16.0	1	Úlcera corneal	1
1	18/01/2020	H11.3	0	Hiposfagma	1
1	18/01/2020	H04.32	5	Dacriocistitis aguda	1
2	18/01/2020	H16.0	1	Úlcera corneal	1
1	18/01/2020	H43.81	7	DVP	1
1	18/01/2020	H16.3	1	Abceso corneal	1
1	18/01/2020	H10.3	0	Conjuntivitis	1
1	18/01/2020	H16.0	1	Úlcera corneal	1
2	18/01/2020	H16.0	1	Úlcera corneal	1
1	18/01/2020	H10.3	0	Conjuntivitis	1
1	18/01/2020	H16.0	1	Úlcera corneal	1
1	18/01/2020	H16.0	1	Úlcera corneal	1
1	18/01/2020	S05.9	9	Traumatismo	1
1	18/01/2020	H16.9	1	Queratitis	1
2	18/01/2020	H10.81	0	Pingueculitis	1
1	18/01/2020	H16.0	1	Úlcera corneal	1
2	19/01/2020	H10.3	0	Conjuntivitis	1
1	19/01/2020	S05.9	9	Traumatismo	1
1	19/01/2020	H43.81	7	DVP	1
1	19/01/2020	H10.3	0	Conjuntivitis	1
2	19/01/2020	H16.9	1	Queratitis	1
1	19/01/2020	H43.81	7	DVP	1
2	19/01/2020	H10.3	0	Conjuntivitis	1
2	19/01/2020	T15.0	1	Cuerpo extraño	1
1	19/01/2020	H16.9	1	Queratitis	1
2	19/01/2020	S05.9	9	Traumatismo	1
2	19/01/2020	H00.02	4	Orzuelo	1
1	19/01/2020	H01.00	4	Blefaritis	1
1	19/01/2020	H43.1	7	Hemovítreo	1
2	19/01/2020	H10.3	0	Conjuntivitis	1
1	19/01/2020	T26	9	Causticación	1
1	19/01/2020	H20	6	Uveitis	1
2	19/01/2020	H16.9	1	Queratitis	1
2	19/01/2020	H04.32	5	Dacriocistitis aguda	1
2	19/01/2020	H33.0	8	Desprendimiento de retina	1
2	19/01/2020	H43.81	7	DVP	1
2	19/01/2020	G43.9	10	Migraña	1
2	19/01/2020	S05.30	9	Laceración conjuntival	1
1	19/01/2020	H16.0	1	Úlcera corneal	1
2	19/01/2020	H10.3	0	Conjuntivitis	1
1	19/01/2020	H40.9	3	Glaucoma	1
2	19/01/2020	H43.81	7	DVP	1
1	19/01/2020	H16.9	1	Queratitis	1
1	19/01/2020	H16.9	1	Queratitis	1
2	19/01/2020	S05.9	9	Traumatismo	1
1	19/01/2020	H01.00	4	Blefaritis	1
1	19/01/2020	S05.9	9	Traumatismo	1
1	19/01/2020	H16.0	1	Úlcera corneal	1
2	19/01/2020	G43.9	10	Migraña	1
2	19/01/2020	H10.3	0	Conjuntivitis	1
2	19/01/2020	S05.9	9	Traumatismo	1

1	19/01/2020	H16.9	1	Queratitis	1
1	19/01/2020	H01.00	4	Blefaritis	1
1	19/01/2020	H11.3	0	Hiposfagma	1
2	19/01/2020	H10.3	0	Conjuntivitis	1
1	19/01/2020	H16.9	1	Queratitis	1
2	19/01/2020	Alta por fuga	11	Alta por fuga	1
2	19/01/2020	H10.3	0	Conjuntivitis	1
2	19/01/2020	H10.3	0	Conjuntivitis	1
1	19/01/2020	H16.0	1	Úlcera corneal	1
2	19/01/2020	H16.0	1	Úlcera corneal	1
2	19/01/2020	H00.03	4	Celulitis preseptal	1
2	19/01/2020	H16.9	1	Queratitis	1
1	19/01/2020	S05.9	9	Traumatismo	1
2	19/01/2020	H10.3	0	Conjuntivitis	1
2	19/01/2020	H10.3	0	Conjuntivitis	1
1	19/01/2020	T15.0	1	Cuerpo extraño	1
1	19/01/2020	H10.3	0	Conjuntivitis	1
1	19/01/2020	T15.0	1	Cuerpo extraño	1
2	19/01/2020	H11.3	0	Hiposfagma	1
2	19/01/2020	H34.9	8	Oclusión arterial	1
2	20/01/2020	H16.9	1	Queratitis	1
1	20/01/2020	H16.9	1	Queratitis	1
2	20/01/2020	H35.3	8	Maculopatía	1
1	20/01/2020	H16.9	1	Queratitis	1
2	20/01/2020	H01.00	4	Blefaritis	1
2	20/01/2020	H27.8	2	OCP	1
2	20/01/2020	H10.3	0	Conjuntivitis	1
2	20/01/2020	B00.1	4	Dermatitis herpética	1
2	20/01/2020	H11.3	0	Hiposfagma	1
2	20/01/2020	H01.00	4	Blefaritis	1
2	20/01/2020	H10.3	0	Conjuntivitis	1
1	20/01/2020	H18.20	1	Edema corneal	1
2	20/01/2020	H35.3	8	Maculopatía	1
2	20/01/2020	H16.9	1	Queratitis	1
1	20/01/2020	H43.81	7	DVP	1
1	20/01/2020	H43.81	7	DVP	1
2	20/01/2020	H34.82	8	Trombosis venosa	1
1	20/01/2020	H02	4	Alteración palpebral	1
2	20/01/2020	E11.319	8	Retinopatía diabética	1
2	20/01/2020	G43.9	10	Migraña	1
2	20/01/2020	H01.00	4	Blefaritis	1
2	20/01/2020	T15.0	1	Cuerpo extraño	1
2	20/01/2020	H16.9	1	Queratitis	1
2	20/01/2020	H35.30	8	DMAE	1
1	20/01/2020	H16.0	1	Úlcera corneal	1
2	20/01/2020	H43.81	7	DVP	1
1	20/01/2020	H11.3	0	Hiposfagma	1
2	20/01/2020	H16.9	1	Queratitis	1
2	20/01/2020	H16.0	1	Úlcera corneal	1
1	20/01/2020	H20	6	Uveitis	1
2	20/01/2020	S05.9	9	Traumatismo	1
1	20/01/2020	H02	4	Alteración palpebral	1
1	20/01/2020	H16.9	1	Queratitis	1
2	20/01/2020	H35.30	8	DMAE	1
2	20/01/2020	H16.9	1	Queratitis	1
2	20/01/2020	H16.9	1	Queratitis	1
2	20/01/2020	H00.02	4	Orzuelo	1
2	20/01/2020	H10.3	0	Conjuntivitis	1
1	20/01/2020	H00.02	4	Orzuelo	1
2	20/01/2020	T26	9	Causticación	1
1	20/01/2020	H10.3	0	Conjuntivitis	1
2	20/01/2020	T15.0	1	Cuerpo extraño	1
2	20/01/2020	H26.9	2	Catarata	1
1	20/01/2020	H01.00	4	Blefaritis	1
2	20/01/2020	S05.9	9	Traumatismo	1
1	20/01/2020	H00.03	4	Celulitis preseptal	1

1	20/01/2020	H02	4	Alteración palpebral	1
1	21/01/2020	H16.9	1	Queratitis	1
1	21/01/2020	H16.0	1	Úlcera corneal	1
2	21/01/2020	S05.9	9	Traumatismo	1
1	21/01/2020	H10.3	0	Conjuntivitis	1
2	21/01/2020	H16.9	1	Queratitis	1
2	21/01/2020	H16.0	1	Úlcera corneal	1
1	21/01/2020	H27.8	2	OCP	1
1	21/01/2020	H16.9	1	Queratitis	1
2	21/01/2020	H43.81	7	DVP	1
1	21/01/2020	G43.9	10	Migraña	1
2	21/01/2020	H11.3	0	Hiposfagma	1
1	21/01/2020	H35.30	8	DMAE	1
1	21/01/2020	T15.0	1	Cuerpo extraño	1
1	21/01/2020	H20	6	Uveitis	1
1	21/01/2020	H16.0	1	Úlcera corneal	1
2	21/01/2020	H16.9	1	Queratitis	1
2	21/01/2020	H16.9	1	Queratitis	1
2	21/01/2020	H16.9	1	Queratitis	1
2	21/01/2020	G43.9	10	Migraña	1
1	21/01/2020	H10.3	0	Conjuntivitis	1
1	21/01/2020	H01.00	4	Blefaritis	1
2	21/01/2020	H10.3	0	Conjuntivitis	1
1	21/01/2020	H10.3	0	Conjuntivitis	1
2	21/01/2020	G43.9	10	Migraña	1
2	21/01/2020	H04.32	5	Dacriocistitis aguda	1
1	21/01/2020	T15.0	1	Cuerpo extraño	1
2	21/01/2020	H16.9	1	Queratitis	1
2	21/01/2020	H43.1	7	Hemovítreo	1
2	21/01/2020	H16.9	1	Queratitis	1
1	21/01/2020	H20	6	Uveitis	1
1	21/01/2020	T15.0	1	Cuerpo extraño	1
2	21/01/2020	H10.3	0	Conjuntivitis	1
2	21/01/2020	H16.9	1	Queratitis	1
1	21/01/2020	T15.0	1	Cuerpo extraño	1
2	21/01/2020	H00.02	4	Orzuelo	1
1	21/01/2020	S05.00	9	Abrasión conjuntival	1
1	21/01/2020	H00.03	4	Celulitis preseptal	1
1	21/01/2020	H20	6	Uveitis	1
2	21/01/2020	H16.0	1	Úlcera corneal	1
1	21/01/2020	T15.0	1	Cuerpo extraño	1
1	22/01/2020	H16.9	1	Queratitis	1
2	22/01/2020	H16.0	1	Úlcera corneal	1
2	22/01/2020	H10.3	0	Conjuntivitis	1
2	22/01/2020	H46	10	Neuritis óptica	1
1	22/01/2020	H01.00	4	Blefaritis	1
1	22/01/2020	T15.0	1	Cuerpo extraño	1
2	22/01/2020	H16.0	1	Úlcera corneal	1
2	22/01/2020	T15.0	1	Cuerpo extraño	1
2	22/01/2020	H00.03	4	Celulitis preseptal	1
2	22/01/2020	H04.32	5	Dacriocistitis aguda	1
1	22/01/2020	H01.00	4	Blefaritis	1
2	22/01/2020	H00.02	4	Orzuelo	1
1	22/01/2020	H20	6	Uveitis	1
2	22/01/2020	H43.81	7	DVP	1
1	22/01/2020	H01.00	4	Blefaritis	1
2	22/01/2020	H35.3	8	Maculopatía	1
1	22/01/2020	H01.00	4	Blefaritis	1
2	22/01/2020	H00.02	4	Orzuelo	1
2	22/01/2020	H00.19	4	Chalazión	1
2	22/01/2020	H10.3	0	Conjuntivitis	1
2	22/01/2020	H16.9	1	Queratitis	1
1	22/01/2020	H16.9	1	Queratitis	1
2	22/01/2020	H16.9	1	Queratitis	1
2	22/01/2020	H10.3	0	Conjuntivitis	1
2	22/01/2020	H10.3	0	Conjuntivitis	1

2	22/01/2020	H10.3	0	Conjuntivitis	1
1	22/01/2020	H16.9	1	Queratitis	1
2	22/01/2020	H01.00	4	Blefaritis	1
2	22/01/2020	H00.02	4	Orzuelo	1
1	22/01/2020	H10.3	0	Conjuntivitis	1
1	22/01/2020	H16.9	1	Queratitis	1
1	22/01/2020	H10.3	0	Conjuntivitis	1
2	22/01/2020	H53.10	11	Problema refractivo	1
1	22/01/2020	H10.3	0	Conjuntivitis	1
1	22/01/2020	H11.3	0	Hiposfagma	1
2	22/01/2020	T15.0	1	Cuerpo extraño	1
2	22/01/2020	H43.81	7	DVP	1
1	22/01/2020	T15.0	1	Cuerpo extraño	1
2	22/01/2020	H16.9	1	Queratitis	1
2	22/01/2020	H16.9	1	Queratitis	1
2	22/01/2020	H10.3	0	Conjuntivitis	1
2	22/01/2020	H16.9	1	Queratitis	1
2	22/01/2020	H16.0	1	Úlcera corneal	1
2	22/01/2020	H43.81	7	DVP	1
1	22/01/2020	H16.0	1	Úlcera corneal	1
2	22/01/2020	H10.3	0	Conjuntivitis	1
2	22/01/2020	H01.00	4	Blefaritis	1
2	22/01/2020	H16.9	1	Queratitis	1
1	22/01/2020	T15.0	1	Cuerpo extraño	1
1	22/01/2020	S05.30	9	Laceración conjuntival	1
1	22/01/2020	H10.3	0	Conjuntivitis	1
1	22/01/2020	H0.41	1	Ojo seco	1
1	22/01/2020	H02	4	Alteración palpebral	1
1	22/01/2020	T15.0	1	Cuerpo extraño	1
1	23/01/2020	H16.9	1	Queratitis	1
1	23/01/2020	T15.0	1	Cuerpo extraño	1
2	23/01/2020	H16.9	1	Queratitis	1
1	23/01/2020	H16.0	1	Úlcera corneal	1
2	23/01/2020	H16.9	1	Queratitis	1
2	23/01/2020	H16.0	1	Úlcera corneal	1
2	23/01/2020	H16.0	1	Úlcera corneal	1
2	23/01/2020	H16.9	1	Queratitis	1
1	23/01/2020	H43.1	7	Hemovítreo	1
2	23/01/2020	H16.9	1	Queratitis	1
2	23/01/2020	H00.02	4	Orzuelo	1
1	23/01/2020	H11.3	0	Hiposfagma	1
2	23/01/2020	H44.009	8	Endoftalmitis	1
2	23/01/2020	H16.9	1	Queratitis	1
1	23/01/2020	H16.9	1	Queratitis	1
2	23/01/2020	H00.02	4	Orzuelo	1
2	23/01/2020	H43.81	7	DVP	1
2	23/01/2020	H16.9	1	Queratitis	1
2	23/01/2020	H43.81	7	DVP	1
2	23/01/2020	H0.41	1	ojo seco	1
1	23/01/2020	H16.9	1	Queratitis	1
1	23/01/2020	H00.02	4	Orzuelo	1
1	23/01/2020	H16.9	1	Queratitis	1
1	23/01/2020	H16.9	1	Queratitis	1
2	23/01/2020	H16.9	1	Queratitis	1
1	23/01/2020	T15.0	1	Cuerpo extraño	1
2	23/01/2020	H16.9	1	Queratitis	1
1	23/01/2020	T15.0	1	Cuerpo extraño	1
2	23/01/2020	H43.81	7	DVP	1
2	23/01/2020	H35.30	8	DMAE	1
1	23/01/2020	H16.9	1	Queratitis	1
1	23/01/2020	H16.9	1	Queratitis	1
1	23/01/2020	B00.1	4	Dermatitis herpética	1
2	23/01/2020	H16.9	1	Queratitis	1
1	23/01/2020	H16.9	1	Queratitis	1
2	23/01/2020	H16.9	1	Queratitis	1
2	23/01/2020	Alta por fuga	11	Alta por fuga	1

1	23/01/2020	T15.0	1	Cuerpo extraño	1
1	23/01/2020	H20	6	Uveitis	1
2	23/01/2020	H16.9	1	Queratitis	1
1	23/01/2020	H40.9	3	Glaucoma	1
2	23/01/2020	H16.9	1	Queratitis	1
2	24/01/2020	H10.3	0	Conjuntivitis	1
2	24/01/2020	H16.0	1	Úlcera corneal	1
2	24/01/2020	H00.02	4	Orzuelo	1
2	24/01/2020	H01.00	4	Blefaritis	1
2	24/01/2020	H40.9	3	Glaucoma	1
2	24/01/2020	H16.9	1	Queratitis	1
1	24/01/2020	H16.0	1	Úlcera corneal	1
1	24/01/2020	H16.9	1	Queratitis	1
2	24/01/2020	H10.3	0	Conjuntivitis	1
2	24/01/2020	H35.30	8	DMAE	1
2	24/01/2020	H11.3	0	Hiposfagma	1
2	24/01/2020	H01.00	4	Blefaritis	1
2	24/01/2020	H33.0	8	Desprendimiento de retina	1
2	24/01/2020	H00.02	4	Orzuelo	1
2	24/01/2020	H01.00	4	Blefaritis	1
2	24/01/2020	H16.9	1	Queratitis	1
2	24/01/2020	H01.00	4	Blefaritis	1
1	24/01/2020	H16.9	1	Queratitis	1
2	24/01/2020	H10.3	0	Conjuntivitis	1
1	24/01/2020	H43.81	7	DVP	1
2	24/01/2020	H16.9	1	Queratitis	1
2	24/01/2020	H16.0	1	Úlcera corneal	1
2	24/01/2020	G43.9	10	Migraña	1
1	24/01/2020	T15.0	1	Cuerpo extraño	1
2	24/01/2020	S05.9	9	Traumatismo	1
2	24/01/2020	H11.3	0	Hiposfagma	1
2	24/01/2020	H17.8	1	Infiltrados corneales	1
2	24/01/2020	S05.9	9	Traumatismo	1
1	24/01/2020	S05.00	9	Abrasión conjuntival	1
1	24/01/2020	H16.0	1	Úlcera corneal	1
2	24/01/2020	H16.0	1	Úlcera corneal	1
2	24/01/2020	H10.3	0	Conjuntivitis	1
2	24/01/2020	H0.41	1	Ojo seco	1
2	24/01/2020	B00.1	4	Dermatitis herpética	1
2	24/01/2020	H10.5	0	Blefaroconjuntivitis	1
2	24/01/2020	H01.00	4	Blefaritis	1
2	24/01/2020	H16.9	1	Queratitis	1
2	24/01/2020	H16.0	1	Úlcera corneal	1
1	24/01/2020	H02	4	Alteración palpebral	1
2	24/01/2020	H01.00	4	Blefaritis	1
1	24/01/2020	T15.0	1	Cuerpo extraño	1
1	24/01/2020	T15.0	1	Cuerpo extraño	1
2	24/01/2020	H11.3	0	Hiposfagma	1
1	24/01/2020	T15.0	1	Cuerpo extraño	1
1	25/01/2020	T15.0	1	Cuerpo extraño	1
1	25/01/2020	H40.9	3	Glaucoma	1
2	25/01/2020	H16.0	1	Úlcera corneal	1
1	25/01/2020	H34.9	8	Oclusión arterial	1
2	25/01/2020	H16.0	1	Úlcera corneal	1
1	25/01/2020	H00.02	4	Orzuelo	1
1	25/01/2020	H16.9	1	Queratitis	1
2	25/01/2020	H44.009	8	Endoftalmitis	1
2	25/01/2020	H16.9	1	Queratitis	1
1	25/01/2020	T15.0	1	Cuerpo extraño	1
2	25/01/2020	H16.0	1	Úlcera corneal	1
1	25/01/2020	S05.9	9	Traumatismo	1
1	25/01/2020	H11.3	0	Hiposfagma	1
2	25/01/2020	H04.32	5	Dacriocistitis aguda	1
2	25/01/2020	H16.9	1	Queratitis	1
1	25/01/2020	H11.3	0	Hiposfagma	1
2	25/01/2020	H10.3	0	Conjuntivitis	1

1	25/01/2020	H16.9	1	Queratitis	1
1	25/01/2020	H16.9	1	Queratitis	1
2	25/01/2020	H10.3	0	Conjuntivitis	1
2	25/01/2020	Alta por fuga	11	Alta por fuga	1
2	25/01/2020	H10.3	0	Conjuntivitis	1
1	25/01/2020	H16.0	1	Úlcera corneal	1
1	25/01/2020	H10.3	0	Conjuntivitis	1
2	25/01/2020	H10.3	0	Conjuntivitis	1
2	25/01/2020	H0.41	1	Ojo seco	1
1	25/01/2020	H40.9	3	Glaucoma	1
2	25/01/2020	H01.00	4	Blefaritis	1
2	25/01/2020	H40.05	3	HTO	1
1	25/01/2020	H10.3	0	Conjuntivitis	1
1	25/01/2020	H43.1	7	Hemovítreo	1
2	25/01/2020	H01.00	4	Blefaritis	1
2	25/01/2020	H0.41	1	Ojo seco	1
2	25/01/2020	H11.3	0	Hiposfagma	1
1	25/01/2020	G43.109	10	Aura	1
1	25/01/2020	S05.9	9	Traumatismo	1
2	25/01/2020	H16.0	1	Úlcera corneal	1
1	25/01/2020	H43.81	7	DVP	1
1	25/01/2020	H16.0	1	Úlcera corneal	1
1	25/01/2020	H11.3	0	Hiposfagma	1
2	25/01/2020	H50.9	10	Estrabismo	1
1	25/01/2020	H16.0	1	Úlcera corneal	1
2	25/01/2020	H10.3	0	Conjuntivitis	1
1	25/01/2020	S05.00	9	Abrasión conjuntival	1
2	26/01/2020	S05.9	9	Traumatismo	1
2	26/01/2020	H10.3	0	Conjuntivitis	1
2	26/01/2020	H10.3	0	Conjuntivitis	1
2	26/01/2020	H10.3	0	Conjuntivitis	1
1	26/01/2020	H20	6	Uveitis	1
1	26/01/2020	H11.3	0	Hiposfagma	1
2	26/01/2020	H10.3	0	Conjuntivitis	1
1	26/01/2020	S05.9	9	Traumatismo	1
1	26/01/2020	H11.3	0	Hiposfagma	1
2	26/01/2020	H0.41	1	Ojo seco	1
1	26/01/2020	H20	6	Uveitis	1
1	26/01/2020	H20	6	Uveitis	1
2	26/01/2020	H40.05	3	HTO	1
2	26/01/2020	H01.00	4	Blefaritis	1
1	26/01/2020	H16.0	1	Úlcera corneal	1
2	26/01/2020	H01.00	4	Blefaritis	1
1	26/01/2020	H15.1	6	Epiescleritis	1
2	26/01/2020	H02	4	Alteración palpebral	1
2	26/01/2020	H11.3	0	Hiposfagma	1
1	26/01/2020	H43.81	7	DVP	1
2	26/01/2020	H0.41	1	Ojo seco	1
2	26/01/2020	S05.9	9	Traumatismo	1
2	26/01/2020	H00.02	4	Orzuelo	1
2	26/01/2020	H01.00	4	Blefaritis	1
2	26/01/2020	H53.10	11	Problema refractivo	1
2	26/01/2020	H01.00	4	Blefaritis	1
1	26/01/2020	T15.0	1	Cuerpo extraño	1
2	26/01/2020	H11.3	0	Hiposfagma	1
2	26/01/2020	T15.0	1	Cuerpo extraño	1
1	26/01/2020	H10.3	0	Conjuntivitis	1
2	26/01/2020	G43.9	10	Migraña	1
1	26/01/2020	H10.3	0	Conjuntivitis	1
1	26/01/2020	H02	4	Alteración palpebral	1
2	26/01/2020	H16.9	1	Queratitis	1
2	26/01/2020	H16.0	1	Úlcera corneal	1
2	26/01/2020	H20	6	Uveitis	1
2	27/01/2020	H16.0	1	Úlcera corneal	1
2	27/01/2020	H00.02	4	Orzuelo	1
1	27/01/2020	H00.19	4	Chalazión	1

2	27/01/2020	H16.9	1	Queratitis	1
2	27/01/2020	H10.3	0	Conjuntivitis	1
1	27/01/2020	H10.3	0	Conjuntivitis	1
2	27/01/2020	H10.3	0	Conjuntivitis	1
2	27/01/2020	H00.02	4	Orzuelo	1
2	27/01/2020	H10.3	0	Conjuntivitis	1
1	27/01/2020	H10.3	0	Conjuntivitis	1
1	27/01/2020	H02	4	Alteración palpebral	1
2	27/01/2020	H00.02	4	Orzuelo	1
2	27/01/2020	Alta por fuga	11	Alta por fuga	1
1	27/01/2020	H40.9	3	Glaucoma	1
1	27/01/2020	T15.0	1	Cuerpo extraño	1
2	27/01/2020	S05.9	9	Traumatismo	1
1	27/01/2020	S05.9	9	Traumatismo	1
2	27/01/2020	H10.3	0	Conjuntivitis	1
1	27/01/2020	H16.0	1	Úlcera corneal	1
2	27/01/2020	H00.02	4	Orzuelo	1
2	27/01/2020	H11.00	0	Pterigium	1
2	27/01/2020	H16.0	1	Úlcera corneal	1
1	27/01/2020	H10.3	0	Conjuntivitis	1
2	27/01/2020	H50.9	10	Estrabismo	1
1	27/01/2020	H00.02	4	Orzuelo	1
1	27/01/2020	H00.02	4	Orzuelo	1
1	27/01/2020	H0.41	1	Ojo seco	1
2	27/01/2020	G43.9	10	Migraña	1
1	27/01/2020	H10.3	0	Conjuntivitis	1
2	27/01/2020	G43.9	10	Migraña	1
1	27/01/2020	H17.8	1	Infiltrados corneales	1
2	27/01/2020	H43.81	7	DVP	1
2	27/01/2020	H11.3	0	Hiposfagma	1
2	27/01/2020	H02	4	Alteración palpebral	1
2	27/01/2020	H16.9	1	Queratitis	1
2	27/01/2020	H11.3	0	Hiposfagma	1
2	27/01/2020	H43.1	7	Hemovítreo	1
1	27/01/2020	S05.30	9	Laceración conjuntival	1
1	27/01/2020	S05.9	9	Traumatismo	1
1	27/01/2020	H11.3	0	Hiposfagma	1
2	27/01/2020	H18.50	1	Distrofia corneal	1
1	27/01/2020	H0.41	1	ojo seco	1
2	27/01/2020	H10.3	0	Conjuntivitis	1
1	27/01/2020	S05.9	9	Traumatismo	1
1	27/01/2020	H43.1	7	Hemovítreo	1
2	27/01/2020	H16.9	1	Queratitis	1
1	27/01/2020	H10.3	0	Conjuntivitis	1
1	27/01/2020	H16.0	1	Úlcera corneal	1
2	27/01/2020	H10.3	0	Conjuntivitis	1
1	27/01/2020	H53.10	11	Problema refractivo	1
1	27/01/2020	H33.30	8	Desgarro retiniano	1
1	27/01/2020	B58.0	8	Toxoplasmosis	1
1	27/01/2020	H16.9	1	Queratitis	1
1	27/01/2020	S05.9	9	Traumatismo	1
2	27/01/2020	H16.0	1	Úlcera corneal	1
1	27/01/2020	H16.0	1	Úlcera corneal	1
1	27/01/2020	G43.9	10	Migraña	1
2	27/01/2020	H16.9	1	Queratitis	1
1	27/01/2020	H16.9	1	Queratitis	1
2	27/01/2020	Alta por fuga	11	Alta por fuga	1
1	28/01/2020	T15.0	1	Cuerpo extraño	1
2	28/01/2020	H16.9	1	Queratitis	1
2	28/01/2020	H10.3	0	Conjuntivitis	1
2	28/01/2020	H43.81	7	DVP	1
2	28/01/2020	H16.9	1	Queratitis	1
2	28/01/2020	H10.3	0	Conjuntivitis	1
2	28/01/2020	H10.3	0	Conjuntivitis	1
1	28/01/2020	H02	4	Alteración palpebral	1
2	28/01/2020	H43.81	7	DVP	1

2	28/01/2020	H16.9	1	Queratitis	1
2	28/01/2020	H16.0	1	Úlcera corneal	1
2	28/01/2020	H02	4	Alteración palpebral	1
2	28/01/2020	H27.8	2	OCP	1
2	28/01/2020	H02	4	Alteración palpebral	1
1	28/01/2020	Alta por fuga	11	Alta por fuga	1
1	28/01/2020	H17.8	1	Infiltrados corneales	1
2	28/01/2020	H01.00	4	Blefaritis	1
1	28/01/2020	H10.3	0	Conjuntivitis	1
2	28/01/2020	H16.9	1	Queratitis	1
2	28/01/2020	H16.9	1	Queratitis	1
1	28/01/2020	H16.9	1	Queratitis	1
1	28/01/2020	H16.0	1	Úlcera corneal	1
1	28/01/2020	H26.9	2	Catarata	1
2	28/01/2020	H16.9	1	Queratitis	1
1	28/01/2020	H16.0	1	Úlcera corneal	1
1	28/01/2020	G43.9	10	Migraña	1
2	28/01/2020	H10.3	0	Conjuntivitis	1
2	28/01/2020	H02	4	Alteración palpebral	1
1	28/01/2020	H16.0	1	Úlcera corneal	1
2	28/01/2020	H10.3	0	Conjuntivitis	1
2	28/01/2020	H43.81	7	DVP	1
2	28/01/2020	H16.9	1	Queratitis	1
1	28/01/2020	Z98.4	2	Post op catarata	1
1	28/01/2020	H16.0	1	Úlcera corneal	1
1	28/01/2020	H11.44	0	Quiste conjuntival	1
1	28/01/2020	H16.9	1	Queratitis	1
2	28/01/2020	H16.9	1	Queratitis	1
2	28/01/2020	H16.9	1	Queratitis	1
2	28/01/2020	G43.9	10	Migraña	1
2	28/01/2020	H16.9	1	Queratitis	1
2	28/01/2020	S05.00	9	Abrasión conjuntival	1
2	28/01/2020	H11.3	0	Hiposfagma	1
2	28/01/2020	H16.9	1	Queratitis	1
2	28/01/2020	H11.3	0	Hiposfagma	1
1	28/01/2020	H05.01	4	Celulitis orbitaria	1
1	28/01/2020	H00.02	4	Orzuelo	1
2	28/01/2020	H16.9	1	Queratitis	1
1	28/01/2020	H20	6	Uveitis	1
1	28/01/2020	S05.9	9	Traumatismo	1
1	28/01/2020	H17.8	1	Infiltrados corneales	1
2	28/01/2020	H10.3	0	Conjuntivitis	1
2	29/01/2020	S05.9	9	Traumatismo	1
1	29/01/2020	T15.0	1	Cuerpo extraño	1
2	29/01/2020	H10.3	0	Conjuntivitis	1
1	29/01/2020	S05.9	9	Traumatismo	1
2	29/01/2020	H16.9	1	Queratitis	1
2	29/01/2020	H16.9	1	Queratitis	1
1	29/01/2020	H16.9	1	Queratitis	1
2	29/01/2020	H16.9	1	Queratitis	1
2	29/01/2020	H16.9	1	Queratitis	1
2	29/01/2020	T26	9	Causticación	1
2	29/01/2020	H10.3	0	Conjuntivitis	1
1	29/01/2020	H10.3	0	Conjuntivitis	1
2	29/01/2020	H16.9	1	Queratitis	1
1	29/01/2020	H44.009	8	Endoftalmitis	1
2	29/01/2020	H10.3	0	Conjuntivitis	1
1	29/01/2020	Alta por fuga	11	Alta por fuga	1
2	29/01/2020	H11.3	0	Hiposfagma	1
2	29/01/2020	H0.41	1	ojo seco	1
2	29/01/2020	H43.81	7	DVP	1
1	29/01/2020	H26.9	2	Catarata	1
2	29/01/2020	H10.3	0	Conjuntivitis	1
2	29/01/2020	H43.1	7	Hemovítreo	1
1	29/01/2020	H16.0	1	Úlcera corneal	1
2	29/01/2020	H04.32	5	Dacriocistitis aguda	1

2	29/01/2020	H16.9	1	Queratitis	1
2	29/01/2020	H43.1	7	Hemovítreo	1
1	29/01/2020	H50.9	10	Estrabismo	1
2	29/01/2020	H17.8	1	Infiltrados corneales	1
1	29/01/2020	H43.81	7	DVP	1
1	29/01/2020	H16.9	1	Queratitis	1
2	29/01/2020	H16.9	1	Queratitis	1
1	29/01/2020	H11.44	0	Quiste conjuntival	1
2	29/01/2020	H00.03	4	Celulitis preseptal	1
1	29/01/2020	H16.0	1	Úlcera corneal	1
2	29/01/2020	S05.00	9	Abrasión conjuntival	1
1	29/01/2020	S05.9	9	Traumatismo	1
1	29/01/2020	H43.81	7	DVP	1
1	29/01/2020	H16.9	1	Queratitis	1
2	29/01/2020	S05.30	9	Laceración conjuntival	1
2	29/01/2020	H11.3	0	Hiposfagma	1
1	29/01/2020	H10.3	0	Conjuntivitis	1
1	29/01/2020	S05.9	9	Traumatismo	1
2	29/01/2020	H11.3	0	Hiposfagma	1
1	29/01/2020	T15.0	1	Cuerpo extraño	1
1	29/01/2020	H16.9	1	Queratitis	1
1	30/01/2020	H00.02	4	Orzuelo	1
2	30/01/2020	H31.42	8	Coroidopatía serosa central	1
1	30/01/2020	T15.0	1	Cuerpo extraño	1
2	30/01/2020	S05.9	9	Traumatismo	1
1	30/01/2020	H10.3	0	Conjuntivitis	1
2	30/01/2020	H10.3	0	Conjuntivitis	1
2	30/01/2020	H11.3	0	Hiposfagma	1
2	30/01/2020	H10.3	0	Conjuntivitis	1
2	30/01/2020	H10.3	0	Conjuntivitis	1
1	30/01/2020	H43.81	7	DVP	1
2	30/01/2020	H26.9	2	Catarata	1
2	30/01/2020	H10.3	0	Conjuntivitis	1
2	30/01/2020	G43.9	10	Migraña	1
2	30/01/2020	H01.00	4	Blefaritis	1
1	30/01/2020	H01.00	4	Blefaritis	1
2	30/01/2020	H01.00	4	Blefaritis	1
1	30/01/2020	H16.9	1	Queratitis	1
2	30/01/2020	H16.9	1	Queratitis	1
1	30/01/2020	H11.3	0	Hiposfagma	1
1	30/01/2020	H43.81	7	DVP	1
2	30/01/2020	H16.9	1	Queratitis	1
2	30/01/2020	H10.3	0	Conjuntivitis	1
2	30/01/2020	H43.81	7	DVP	1
1	30/01/2020	H26.9	2	Catarata	1
1	30/01/2020	H11.3	0	Hiposfagma	1
2	30/01/2020	H50.9	10	Estrabismo	1
2	30/01/2020	H16.9	1	Queratitis	1
2	30/01/2020	H11.3	0	Hiposfagma	1
1	30/01/2020	H10.3	0	Conjuntivitis	1
2	30/01/2020	H26.9	2	Catarata	1
1	30/01/2020	H16.9	1	Queratitis	1
2	30/01/2020	H10.3	0	Conjuntivitis	1
1	30/01/2020	H10.3	0	Conjuntivitis	1
2	30/01/2020	Z98.4	2	Post op catarata	1
2	30/01/2020	H01.00	4	Blefaritis	1
1	30/01/2020	G43.109	10	Aura	1
2	30/01/2020	G43.9	10	Migraña	1
2	30/01/2020	H10.81	0	Pingueculitis	1
1	30/01/2020	H10.3	0	Conjuntivitis	1
1	30/01/2020	Alta por fuga	11	Alta por fuga	1
1	30/01/2020	H31.42	8	Coroidopatía serosa central	1
2	30/01/2020	H02	4	Alteración palpebral	1
2	30/01/2020	H16.9	1	Queratitis	1
1	30/01/2020	H00.02	4	Orzuelo	1
2	30/01/2020	H00.02	4	Orzuelo	1

1	30/01/2020	S05.9	9	Traumatismo	1
2	30/01/2020	H16.9	1	Queratitis	1
1	30/01/2020	T15.0	1	Cuerpo extraño	1
2	30/01/2020	S05.9	9	Traumatismo	1
1	30/01/2020	H16.0	1	Úlcera corneal	1
2	31/01/2020	G43.9	10	Migraña	1
1	31/01/2020	H10.3	0	Conjuntivitis	1
2	31/01/2020	H18.20	1	Edema corneal	1
1	31/01/2020	H10.3	0	Conjuntivitis	1
2	31/01/2020	H00.19	4	Chalazión	1
2	31/01/2020	H11.3	0	Hiposfagma	1
1	31/01/2020	H43.81	7	DVP	1
2	31/01/2020	H00.02	4	Orzuelo	1
2	31/01/2020	H04.32	5	Dacriocistitis aguda	1
2	31/01/2020	H02	4	Alteración palpebral	1
1	31/01/2020	H43.81	7	DVP	1
1	31/01/2020	H01.00	4	Blefaritis	1
2	31/01/2020	H16.9	1	Queratitis	1
1	31/01/2020	S05.9	9	Traumatismo	1
2	31/01/2020	H00.19	4	Chalazión	1
1	31/01/2020	H44.009	8	Endoftalmitis	1
2	31/01/2020	H26.9	2	Catarata	1
1	31/01/2020	H43.1	7	Hemovítreo	1
1	31/01/2020	H20	6	Uveitis	1
2	31/01/2020	H16.9	1	Queratitis	1
1	31/01/2020	H43.81	7	DVP	1
1	31/01/2020	H16.9	1	Queratitis	1
1	31/01/2020	H11.3	0	Hiposfagma	1
2	31/01/2020	H04.32	5	Dacriocistitis aguda	1
2	31/01/2020	H11.3	0	Hiposfagma	1
2	31/01/2020	H43.81	7	DVP	1
2	31/01/2020	H02	4	Alteración palpebral	1
1	31/01/2020	H43.81	7	DVP	1
2	31/01/2020	G43.109	10	Aura	1
2	31/01/2020	H10.3	0	Conjuntivitis	1
2	31/01/2020	H11.3	0	Hiposfagma	1
1	31/01/2020	H10.3	0	Conjuntivitis	1
1	31/01/2020	H02	4	Alteración palpebral	1
1	31/01/2020	H44.009	8	Endoftalmitis	1
2	31/01/2020	H16.9	1	Queratitis	1
2	31/01/2020	H27.8	2	OCP	1
1	31/01/2020	H20	6	Uveitis	1
2	31/01/2020	H11.44	0	Quiste conjuntival	1
1	31/01/2020	T15.0	1	Cuerpo extraño	1
2	31/01/2020	H16.9	1	Queratitis	1
2	31/01/2020	B00.1	4	Dermatitis herpética	1
2	31/01/2020	H10.3	0	Conjuntivitis	1
1	31/01/2020	H27.8	2	OCP	1
1	31/01/2020	H16.9	1	Queratitis	1
2	31/01/2020	H16.9	1	Queratitis	1
2	31/01/2020	H33.0	8	Desprendimiento de retina	1
2	31/01/2020	H04.32	5	Dacriocistitis aguda	1
2	31/01/2020	H10.3	0	Conjuntivitis	1
1	31/01/2020	H43.81	7	DVP	1
1	31/01/2020	H16.9	1	Queratitis	1
1	31/01/2020	S05.9	9	Traumatismo	1
2	31/01/2020	G43.9	10	Migraña	1
2	31/01/2020	H10.3	0	Conjuntivitis	1
2	31/01/2020	H01.00	4	Blefaritis	1
1	31/01/2020	H16.9	1	Queratitis	1
1	31/01/2020	H00.02	4	Orzuelo	1
2	31/01/2020	G43.9	10	Migraña	1
1	31/01/2020	H16.0	1	Úlcera corneal	1
2	31/01/2020	T15.0	1	Cuerpo extraño	1
2	01/02/2020	H11.3	0	Hiposfagma	1
1	01/02/2020	H16.0	1	Úlcera corneal	1

1	01/02/2020	H10.3	0	Conjuntivitis	1
1	01/02/2020	T15.0	1	Cuerpo extraño	1
1	01/02/2020	H40.9	3	Glaucoma	1
2	01/02/2020	H11.3	0	Hiposfagma	1
1	01/02/2020	H43.1	7	Hemovítreo	1
1	01/02/2020	H16.0	1	Úlcera corneal	1
1	01/02/2020	H17.8	1	Infiltrados corneales	1
2	01/02/2020	S05.9	9	Traumatismo	1
2	01/02/2020	H04.32	5	Dacriocistitis aguda	1
1	01/02/2020	H20	6	Uveitis	1
2	01/02/2020	H10.3	0	Conjuntivitis	1
1	01/02/2020	H11.3	0	Hiposfagma	1
2	01/02/2020	H16.9	1	Queratitis	1
2	01/02/2020	H16.0	1	Úlcera corneal	1
2	01/02/2020	H17.8	1	Infiltrados corneales	1
2	01/02/2020	B00.1	4	Dermatitis herpética	1
1	01/02/2020	T15.0	1	Cuerpo extraño	1
1	01/02/2020	H10.3	0	Conjuntivitis	1
2	01/02/2020	H16.9	1	Queratitis	1
2	01/02/2020	H02	4	Alteración palpebral	1
2	01/02/2020	H11.3	0	Hiposfagma	1
2	01/02/2020	H04.32	5	Dacriocistitis aguda	1
2	01/02/2020	H10.3	0	Conjuntivitis	1
2	01/02/2020	H15.1	6	Epiescleritis	1
2	01/02/2020	H10.3	0	Conjuntivitis	1
2	01/02/2020	H35.30	8	DMAE	1
1	01/02/2020	H16.9	1	Queratitis	1
2	01/02/2020	S05.9	9	Traumatismo	1
2	01/02/2020	H16.0	1	Úlcera corneal	1
2	01/02/2020	H10.81	0	Pingueculitis	1
1	01/02/2020	H00.02	4	Orzuelo	1
2	01/02/2020	H43.81	7	DVP	1
2	01/02/2020	H16.9	1	Queratitis	1
2	01/02/2020	H16.9	1	Queratitis	1
2	01/02/2020	S05.00	9	Abrasión conjuntival	1
2	01/02/2020	S05.9	9	Traumatismo	1
1	01/02/2020	S05.9	9	Traumatismo	1
1	01/02/2020	T15.0	1	Cuerpo extraño	1
2	01/02/2020	H16.9	1	Queratitis	1
2	01/02/2020	B00.1	4	Dermatitis herpética	1
1	01/02/2020	Alta por fuga	11	Alta por fuga	1
1	01/02/2020	H0.41	1	ojo seco	1
1	01/02/2020	Alta por fuga	11	Alta por fuga	1
1	01/02/2020	H10.3	0	Conjuntivitis	1
1	01/02/2020	H04.32	5	Dacriocistitis aguda	1
2	01/02/2020	H10.3	0	Conjuntivitis	1
2	01/02/2020	H10.3	0	Conjuntivitis	1
1	01/02/2020	H10.3	0	Conjuntivitis	1
1	01/02/2020	H16.0	1	Úlcera corneal	1
2	01/02/2020	H16.9	1	Queratitis	1
2	02/02/2020	H43.81	7	DVP	1
1	02/02/2020	H16.9	1	Queratitis	1
2	02/02/2020	H10.3	0	Conjuntivitis	1
2	02/02/2020	H0.41	1	Ojo seco	1
2	02/02/2020	H10.3	0	Conjuntivitis	1
1	02/02/2020	H16.0	1	Úlcera corneal	1
2	02/02/2020	H16.0	1	Úlcera corneal	1
2	02/02/2020	H0.41	1	Ojo seco	1
2	02/02/2020	H10.3	0	Conjuntivitis	1
1	02/02/2020	H00.02	4	Orzuelo	1
1	02/02/2020	H10.3	0	Conjuntivitis	1
1	02/02/2020	H16.0	1	Úlcera corneal	1
1	02/02/2020	H16.9	1	Queratitis	1
2	02/02/2020	H35.30	8	DMAE	1
2	02/02/2020	H53	11	No patología	1
2	02/02/2020	H16.9	1	Queratitis	1

2	02/02/2020	H17.8	1	Infiltrados corneales	1
1	02/02/2020	H11.3	0	Hiposfagma	1
1	02/02/2020	H16.9	1	Queratitis	1
2	02/02/2020	H20	6	Uveitis	1
2	02/02/2020	H43.1	7	Hemovítreo	1
2	02/02/2020	H01.00	4	Blefaritis	1
1	02/02/2020	H43.81	7	DVP	1
2	02/02/2020	H10.3	0	Conjuntivitis	1
1	02/02/2020	H44.009	8	Endoftalmitis	1
1	02/02/2020	H10.3	0	Conjuntivitis	1
1	02/02/2020	H16.9	1	Queratitis	1
1	02/02/2020	H33.30	8	Desgarro retiniano	1
2	02/02/2020	H43.81	7	DVP	1
2	02/02/2020	H16.9	1	Queratitis	1
2	02/02/2020	H16.9	1	Queratitis	1
2	02/02/2020	H10.3	0	Conjuntivitis	1
2	02/02/2020	H0.41	1	Ojo seco	1
1	02/02/2020	H43.1	7	Hemovítreo	1
1	02/02/2020	H11.3	0	Hiposfagma	1
1	02/02/2020	H11.3	0	Hiposfagma	1
2	02/02/2020	H0.41	1	Ojo seco	1
2	02/02/2020	T26	9	Causticación	1
1	02/02/2020	H10.3	0	Conjuntivitis	1
2	02/02/2020	S05.9	9	Traumatismo	1
2	02/02/2020	H26.9	2	Catarata	1
2	02/02/2020	T15.0	1	Cuerpo extraño	1
1	02/02/2020	H10.3	0	Conjuntivitis	1
2	03/02/2020	H20	6	Uveitis	1
1	03/02/2020	H10.3	0	Conjuntivitis	1
2	03/02/2020	H00.02	4	Orzuelo	1
1	03/02/2020	H11.3	0	Hiposfagma	1
2	03/02/2020	H16.0	1	Úlcera corneal	1
1	03/02/2020	H04.32	5	Dacriocistitis aguda	1
2	03/02/2020	H16.9	1	Queratitis	1
2	03/02/2020	H15.1	6	Epiescleritis	1
2	03/02/2020	H01.00	4	Blefaritis	1
2	03/02/2020	H11.3	0	Hiposfagma	1
1	03/02/2020	H00.02	4	Orzuelo	1
2	03/02/2020	H10.3	0	Conjuntivitis	1
2	03/02/2020	H10.3	0	Conjuntivitis	1
2	03/02/2020	H16.9	1	Queratitis	1
2	03/02/2020	H20	6	Uveitis	1
2	03/02/2020	H10.3	0	Conjuntivitis	1
1	03/02/2020	H01.00	4	Blefaritis	1
2	03/02/2020	H33.0	8	Desprendimiento de retina	1
2	03/02/2020	H16.9	1	Queratitis	1
1	03/02/2020	H43.81	7	DVP	1
2	03/02/2020	H10.3	0	Conjuntivitis	1
1	03/02/2020	H27.8	2	OCP	1
1	03/02/2020	S05.9	9	Traumatismo	1
2	03/02/2020	H11.3	0	Hiposfagma	1
2	03/02/2020	H10.3	0	Conjuntivitis	1
2	03/02/2020	H53.10	11	Problema refractivo	1
1	03/02/2020	H02	4	Alteración palpebral	1
2	03/02/2020	H10.3	0	Conjuntivitis	1
2	03/02/2020	B00.1	4	Dermatitis herpética	1
2	03/02/2020	H53.10	11	Problema refractivo	1
2	03/02/2020	H10.3	0	Conjuntivitis	1
2	03/02/2020	H16.9	1	Queratitis	1
2	03/02/2020	H16.9	1	Queratitis	1
2	03/02/2020	H01.00	4	Blefaritis	1
2	03/02/2020	H04.32	5	Dacriocistitis aguda	1
1	03/02/2020	H10.3	0	Conjuntivitis	1
2	03/02/2020	H53.10	11	Problema refractivo	1
2	03/02/2020	H11.3	0	Hiposfagma	1
2	03/02/2020	H16.9	1	Queratitis	1

2	03/02/2020	H01.00	4	Blefaritis	1
1	03/02/2020	S05.9	9	Traumatismo	1
2	03/02/2020	H11.44	0	Quiste conjuntival	1
1	03/02/2020	H01.00	4	Blefaritis	1
1	03/02/2020	H01.00	4	Blefaritis	1
1	03/02/2020	S05.9	9	Traumatismo	1
2	03/02/2020	H10.3	0	Conjuntivitis	1
2	03/02/2020	H53.10	11	Problema refractivo	1
2	03/02/2020	H16.9	1	Queratitis	1
1	03/02/2020	T15.0	1	Cuerpo extraño	1
1	03/02/2020	B00.1	4	Dermatitis herpética	1
1	03/02/2020	H49.9	10	Parálisis oculomotora	1
2	03/02/2020	H33.0	8	Desprendimiento de retina	1
1	04/02/2020	H00.02	4	Orzuelo	1
2	04/02/2020	H27.8	2	OCP	1
2	04/02/2020	H10.3	0	Conjuntivitis	1
2	04/02/2020	H53	11	No patología	1
1	04/02/2020	G43.9	10	Migraña	1
1	04/02/2020	H27.8	2	OCP	1
2	04/02/2020	H0.41	1	Ojo seco	1
1	04/02/2020	H10.3	0	Conjuntivitis	1
2	04/02/2020	S05.00	9	Abrasión conjuntival	1
1	04/02/2020	H10.3	0	Conjuntivitis	1
1	04/02/2020	H35.3	8	Maculopatía	1
2	04/02/2020	H16.0	1	Úlcera corneal	1
1	04/02/2020	H31.42	8	Coroidopatía serosa central	1
1	04/02/2020	G43.9	10	Migraña	1
2	04/02/2020	S05.6	9	Perforación ocular	1
2	04/02/2020	H43.81	7	DVP	1
2	04/02/2020	H10.3	0	Conjuntivitis	1
1	04/02/2020	H11.3	0	Hiposfagma	1
2	04/02/2020	H0.41	1	Ojo seco	1
1	04/02/2020	H16.9	1	Queratitis	1
1	04/02/2020	T15.0	1	Cuerpo extraño	1
2	04/02/2020	H04.32	5	Dacriocistitis aguda	1
2	04/02/2020	T15.0	1	Cuerpo extraño	1
1	04/02/2020	H10.3	0	Conjuntivitis	1
1	04/02/2020	H11.3	0	Hiposfagma	1
2	04/02/2020	H00.02	4	Orzuelo	1
1	04/02/2020	H26.9	2	Catarata	1
1	04/02/2020	H11.3	0	Hiposfagma	1
2	04/02/2020	H16.0	1	Úlcera corneal	1
1	04/02/2020	H20	6	Uveitis	1
2	04/02/2020	H10.3	0	Conjuntivitis	1
1	04/02/2020	H10.3	0	Conjuntivitis	1
2	04/02/2020	H11.44	0	Quiste conjuntival	1
1	04/02/2020	H16.9	1	Queratitis	1
2	04/02/2020	H16.9	1	Queratitis	1
2	04/02/2020	H16.9	1	Queratitis	1
1	04/02/2020	H10.3	0	Conjuntivitis	1
1	04/02/2020	T15.0	1	Cuerpo extraño	1
1	04/02/2020	H10.3	0	Conjuntivitis	1
2	04/02/2020	H53	11	No patología	1
2	04/02/2020	H16.9	1	Queratitis	1
1	04/02/2020	H00.02	4	Orzuelo	1
2	04/02/2020	S05.9	9	Traumatismo	1
2	04/02/2020	H26.9	2	Catarata	1
1	04/02/2020	H10.3	0	Conjuntivitis	1
2	04/02/2020	H10.3	0	Conjuntivitis	1
1	04/02/2020	H11.3	0	Hiposfagma	1
1	04/02/2020	S05.9	9	Traumatismo	1
1	04/02/2020	H16.9	1	Queratitis	1
1	05/02/2020	T15.0	1	Cuerpo extraño	1
1	05/02/2020	T15.0	1	Cuerpo extraño	1
1	05/02/2020	H11.00	0	Pterigium	1
2	05/02/2020	H43.81	7	DVP	1

1	05/02/2020	H17.8	1	Infiltrados corneales	1
1	05/02/2020	H16.9	1	Queratitis	1
2	05/02/2020	H01.00	4	Blefaritis	1
2	05/02/2020	H16.9	1	Queratitis	1
2	05/02/2020	H27.8	2	OCP	1
1	05/02/2020	S05.9	9	Traumatismo	1
2	05/02/2020	H10.3	0	Conjuntivitis	1
1	05/02/2020	Z98.4	2	Post op catarata	1
2	05/02/2020	H10.3	0	Conjuntivitis	1
2	05/02/2020	H02	4	Alteración palpebral	1
2	05/02/2020	H16.9	1	Queratitis	1
1	05/02/2020	H40.05	3	HTO	1
2	05/02/2020	H10.3	0	Conjuntivitis	1
2	05/02/2020	H16.0	1	Úlcera corneal	1
2	05/02/2020	H35.30	8	DMAE	1
1	05/02/2020	H16.0	1	Úlcera corneal	1
1	05/02/2020	H10.3	0	Conjuntivitis	1
1	05/02/2020	H16.9	1	Queratitis	1
1	05/02/2020	H16.0	1	Úlcera corneal	1
2	05/02/2020	H00.02	4	Orzuelo	1
1	05/02/2020	H16.9	1	Queratitis	1
1	05/02/2020	H16.9	1	Queratitis	1
1	05/02/2020	T15.0	1	Cuerpo extraño	1
2	05/02/2020	H10.3	0	Conjuntivitis	1
2	05/02/2020	H00.02	4	Orzuelo	1
1	05/02/2020	H34.82	8	Trombosis venosa	1
2	05/02/2020	H35.30	8	DMAE	1
2	05/02/2020	H0.41	1	Ojo seco	1
1	05/02/2020	H16.9	1	Queratitis	1
1	05/02/2020	T15.0	1	Cuerpo extraño	1
1	05/02/2020	H43.81	7	DVP	1
2	05/02/2020	H10.3	0	Conjuntivitis	1
2	05/02/2020	H0.41	1	Ojo seco	1
2	05/02/2020	H18.50	1	Distrofia corneal	1
1	05/02/2020	H43.81	7	DVP	1
1	05/02/2020	H15.00	6	Escleritis	1
1	05/02/2020	H10.3	0	Conjuntivitis	1
1	05/02/2020	H16.0	1	Úlcera corneal	1
2	05/02/2020	H01.00	4	Blefaritis	1
2	05/02/2020	H11.3	0	Hiposfagma	1
1	06/02/2020	T15.0	1	Cuerpo extraño	1
2	06/02/2020	H10.3	0	Conjuntivitis	1
2	06/02/2020	H10.3	0	Conjuntivitis	1
1	06/02/2020	S05.9	9	Traumatismo	1
2	06/02/2020	H10.3	0	Conjuntivitis	1
1	06/02/2020	H01.00	4	Blefaritis	1
1	06/02/2020	S05.9	9	Traumatismo	1
1	06/02/2020	H10.3	0	Conjuntivitis	1
1	06/02/2020	H50.9	10	Estrabismo	1
1	06/02/2020	H16.0	1	Úlcera corneal	1
1	06/02/2020	H16.9	1	Queratitis	1
1	06/02/2020	H44.009	8	Endoftalmitis	1
2	06/02/2020	H16.9	1	Queratitis	1
1	06/02/2020	H10.3	0	Conjuntivitis	1
2	06/02/2020	H16.9	1	Queratitis	1
2	06/02/2020	H16.9	1	Queratitis	1
2	06/02/2020	H16.9	1	Queratitis	1
1	06/02/2020	H10.3	0	Conjuntivitis	1
2	06/02/2020	H02	4	Alteración palpebral	1
2	06/02/2020	H31.42	8	Coroidopatía serosa central	1
2	06/02/2020	H10.3	0	Conjuntivitis	1
1	06/02/2020	H16.9	1	Queratitis	1
1	06/02/2020	H00.02	4	Orzuelo	1
2	06/02/2020	H01.00	4	Blefaritis	1
2	06/02/2020	H10.3	0	Conjuntivitis	1
2	06/02/2020	H11.3	0	Hiposfagma	1

2	06/02/2020	H10.3	0	Conjuntivitis	1
2	06/02/2020	H10.3	0	Conjuntivitis	1
2	06/02/2020	H16.9	1	Queratitis	1
2	06/02/2020	H10.3	0	Conjuntivitis	1
2	06/02/2020	S05.9	9	Traumatismo	1
2	06/02/2020	T15.0	1	Cuerpo extraño	1
2	06/02/2020	H11.3	0	Hiposfagma	1
1	06/02/2020	H10.3	0	Conjuntivitis	1
2	06/02/2020	H16.9	1	Queratitis	1
2	06/02/2020	H01.00	4	Blefaritis	1
2	06/02/2020	H00.02	4	Orzuelo	1
2	06/02/2020	H00.02	4	Orzuelo	1
2	06/02/2020	H00.02	4	Orzuelo	1
1	06/02/2020	H16.0	1	Úlcera corneal	1
1	06/02/2020	T15.0	1	Cuerpo extraño	1
1	07/02/2020	H10.3	0	Conjuntivitis	1
2	07/02/2020	H10.3	0	Conjuntivitis	1
1	07/02/2020	H01.00	4	Blefaritis	1
2	07/02/2020	H16.0	1	Úlcera corneal	1
1	07/02/2020	H35.30	8	DMAE	1
1	07/02/2020	H43.81	7	DVP	1
1	07/02/2020	T15.0	1	Cuerpo extraño	1
1	07/02/2020	T15.0	1	Cuerpo extraño	1
2	07/02/2020	H59.88	8	Post op retina	1
2	07/02/2020	G43.9	10	Migraña	1
2	07/02/2020	H43.81	7	DVP	1
2	07/02/2020	H40.05	3	HTO	1
2	07/02/2020	H15.1	6	Epiescleritis	1
1	07/02/2020	H00.02	4	Orzuelo	1
2	07/02/2020	H04.32	5	Dacriocistitis aguda	1
1	07/02/2020	H20	6	Uveitis	1
2	07/02/2020	H16.9	1	Queratitis	1
1	07/02/2020	H00.02	4	Orzuelo	1
2	07/02/2020	H00.02	4	Orzuelo	1
2	07/02/2020	H02	4	Alteración palpebral	1
2	07/02/2020	H10.3	0	Conjuntivitis	1
2	07/02/2020	T15.0	1	Cuerpo extraño	1
2	07/02/2020	H35.3	8	Maculopatía	1
1	07/02/2020	H10.3	0	Conjuntivitis	1
2	07/02/2020	H43.81	7	DVP	1
2	07/02/2020	H0.41	1	ojo seco	1
2	07/02/2020	H01.00	4	Blefaritis	1
2	07/02/2020	H43.81	7	DVP	1
1	07/02/2020	H10.3	0	Conjuntivitis	1
2	07/02/2020	H17.8	1	Infiltrados corneales	1
2	07/02/2020	H16.9	1	Queratitis	1
1	07/02/2020	H20	6	Uveitis	1
2	07/02/2020	H27.8	2	OCP	1
2	07/02/2020	H43.81	7	DVP	1
2	07/02/2020	Z98.4	2	Post op catarata	1
2	07/02/2020	H11.3	0	Hiposfagma	1
1	07/02/2020	S05.30	9	Laceración conjuntival	1
2	07/02/2020	H11.3	0	Hiposfagma	1
1	07/02/2020	H16.9	1	Queratitis	1
1	07/02/2020	H16.9	1	Queratitis	1
1	07/02/2020	S05.9	9	Traumatismo	1
1	07/02/2020	H10.3	0	Conjuntivitis	1
2	07/02/2020	H10.3	0	Conjuntivitis	1
2	07/02/2020	H17.8	1	Infiltrados corneales	1
2	07/02/2020	H10.3	0	Conjuntivitis	1
2	07/02/2020	H43.81	7	DVP	1
2	07/02/2020	H0.41	1	Ojo seco	1
2	07/02/2020	H53.10	11	Problema refractivo	1
1	07/02/2020	H43.81	7	DVP	1
2	07/02/2020	H26.9	2	Catarata	1
2	07/02/2020	H10.3	0	Conjuntivitis	1

2	07/02/2020	H16.9	1	Queratitis	1
2	08/02/2020	H15.00	6	Escleritis	1
2	08/02/2020	H04.32	5	Dacriocistitis aguda	1
2	08/02/2020	T15.0	1	Cuerpo extraño	1
1	08/02/2020	H16.0	1	Úlcera corneal	1
1	08/02/2020	S05.6	9	Perforación ocular	1
2	08/02/2020	H04.32	5	Dacriocistitis aguda	1
1	08/02/2020	H44.009	8	Endoftalmitis	1
2	08/02/2020	H33.30	8	Desgarro retiniano	1
2	08/02/2020	Alta por fuga	11	Alta por fuga	1
1	08/02/2020	H10.3	0	Conjuntivitis	1
1	08/02/2020	S05.9	9	Traumatismo	1
1	08/02/2020	H11.3	0	Hiposfagma	1
2	08/02/2020	H43.81	7	DVP	1
2	08/02/2020	H10.3	0	Conjuntivitis	1
2	08/02/2020	H10.3	0	Conjuntivitis	1
1	08/02/2020	H44.009	8	Endoftalmitis	1
2	08/02/2020	H02	4	Alteración palpebral	1
2	08/02/2020	H11.3	0	Hiposfagma	1
2	08/02/2020	H11.3	0	Hiposfagma	1
2	08/02/2020	H16.9	1	Queratitis	1
1	08/02/2020	H10.3	0	Conjuntivitis	1
1	08/02/2020	H00.19	4	Chalazión	1
1	08/02/2020	S05.9	9	Traumatismo	1
1	08/02/2020	H16.9	1	Queratitis	1
2	08/02/2020	H16.9	1	Queratitis	1
2	08/02/2020	S05.9	9	Traumatismo	1
1	08/02/2020	H10.3	0	Conjuntivitis	1
1	08/02/2020	S05.9	9	Traumatismo	1
1	08/02/2020	H16.0	1	Úlcera corneal	1
2	08/02/2020	H43.1	7	Hemovítreo	1
1	08/02/2020	T15.0	1	Cuerpo extraño	1
1	08/02/2020	H16.0	1	Úlcera corneal	1
1	08/02/2020	H16.0	1	Úlcera corneal	1
2	08/02/2020	H00.02	4	Orzuelo	1
2	08/02/2020	H16.9	1	Queratitis	1
2	08/02/2020	S05.00	9	Abrasión conjuntival	1
1	08/02/2020	S05.9	9	Traumatismo	1
1	08/02/2020	T15.0	1	Cuerpo extraño	1
2	08/02/2020	H16.9	1	Queratitis	1
2	08/02/2020	H16.9	1	Queratitis	1
2	08/02/2020	S05.9	9	Traumatismo	1
1	08/02/2020	H01.00	4	Blefaritis	1
2	08/02/2020	H16.0	1	Úlcera corneal	1
1	08/02/2020	H10.3	0	Conjuntivitis	1
1	08/02/2020	S05.9	9	Traumatismo	1
2	09/02/2020	H16.9	1	Queratitis	1
2	09/02/2020	T26	9	Causticación	1
2	09/02/2020	H16.0	1	Úlcera corneal	1
1	09/02/2020	H34.82	8	Trombosis venosa	1
1	09/02/2020	H46	10	Neuritis óptica	1
2	09/02/2020	H10.3	0	Conjuntivitis	1
1	09/02/2020	H44.009	8	Endoftalmitis	1
1	09/02/2020	H16.0	1	Úlcera corneal	1
2	09/02/2020	H10.3	0	Conjuntivitis	1
1	09/02/2020	H10.3	0	Conjuntivitis	1
2	09/02/2020	H10.3	0	Conjuntivitis	1
2	09/02/2020	H00.02	4	Orzuelo	1
1	09/02/2020	H10.3	0	Conjuntivitis	1
1	09/02/2020	H00.02	4	Orzuelo	1
1	09/02/2020	T15.0	1	Cuerpo extraño	1
2	09/02/2020	H16.0	1	Úlcera corneal	1
1	09/02/2020	T15.0	1	Cuerpo extraño	1
1	09/02/2020	S05.9	9	Traumatismo	1
1	09/02/2020	H10.3	0	Conjuntivitis	1
2	09/02/2020	H53.10	11	Problema refractivo	1

1	09/02/2020	H16.0	1	Úlcera corneal	1
2	09/02/2020	H18.20	1	Edema corneal	1
2	09/02/2020	H10.3	0	Conjuntivitis	1
1	09/02/2020	H10.3	0	Conjuntivitis	1
1	09/02/2020	H11.3	0	Hiposfagma	1
1	09/02/2020	H16.0	1	Úlcera corneal	1
2	09/02/2020	H15.1	6	Epiescleritis	1
2	09/02/2020	H16.9	1	Queratitis	1
1	09/02/2020	T15.0	1	Cuerpo extraño	1
2	09/02/2020	H01.00	4	Blefaritis	1
1	09/02/2020	H16.9	1	Queratitis	1
2	09/02/2020	H11.3	0	Hiposfagma	1
2	09/02/2020	H10.3	0	Conjuntivitis	1
2	09/02/2020	S05.9	9	Traumatismo	1
1	09/02/2020	T15.0	1	Cuerpo extraño	1
2	09/02/2020	H20	6	Uveitis	1
1	09/02/2020	H16.9	1	Queratitis	1
2	09/02/2020	H10.3	0	Conjuntivitis	1
2	09/02/2020	H16.0	1	Úlcera corneal	1
2	09/02/2020	H00.02	4	Orzuelo	1
1	09/02/2020	H20	6	Uveitis	1
1	09/02/2020	H33.0	8	Desprendimiento de retina	1
1	09/02/2020	H33.30	8	Desgarro retiniano	1
2	09/02/2020	G43.9	10	Migraña	1
2	09/02/2020	H10.3	0	Conjuntivitis	1
2	09/02/2020	H10.3	0	Conjuntivitis	1
2	10/02/2020	H10.3	0	Conjuntivitis	1
1	10/02/2020	H10.3	0	Conjuntivitis	1
1	10/02/2020	H16.9	1	Queratitis	1
1	10/02/2020	H43.81	7	DVP	1
1	10/02/2020	H43.81	7	DVP	1
2	10/02/2020	H11.3	0	Hiposfagma	1
1	10/02/2020	H10.3	0	Conjuntivitis	1
2	10/02/2020	H10.3	0	Conjuntivitis	1
1	10/02/2020	H11.3	0	Hiposfagma	1
1	10/02/2020	H16.0	1	Úlcera corneal	1
2	10/02/2020	H43.81	7	DVP	1
2	10/02/2020	H10.3	0	Conjuntivitis	1
2	10/02/2020	H53	11	No patología	1
2	10/02/2020	H01.00	4	Blefaritis	1
2	10/02/2020	H35.30	8	DMAE	1
2	10/02/2020	H10.3	0	Conjuntivitis	1
2	10/02/2020	H16.9	1	Queratitis	1
1	10/02/2020	S05.9	9	Traumatismo	1
1	10/02/2020	H34.9	8	Oclusión arterial	1
1	10/02/2020	H11.3	0	Hiposfagma	1
2	10/02/2020	H11.3	0	Hiposfagma	1
2	10/02/2020	H43.81	7	DVP	1
2	10/02/2020	H10.3	0	Conjuntivitis	1
1	10/02/2020	H10.3	0	Conjuntivitis	1
2	10/02/2020	H10.3	0	Conjuntivitis	1
2	10/02/2020	H16.9	1	Queratitis	1
2	10/02/2020	H43.81	7	DVP	1
1	10/02/2020	H35.3	8	Maculopatía	1
2	10/02/2020	H01.00	4	Blefaritis	1
2	10/02/2020	H10.3	0	Conjuntivitis	1
2	10/02/2020	H46	10	Neuritis óptica	1
2	10/02/2020	H53.9	11	Alteraciones visuales	1
2	10/02/2020	H10.3	0	Conjuntivitis	1
1	10/02/2020	H11.44	0	Quiste conjuntival	1
2	10/02/2020	H16.9	1	Queratitis	1
1	10/02/2020	H16.9	1	Queratitis	1
1	10/02/2020	H01.00	4	Blefaritis	1
2	10/02/2020	H43.81	7	DVP	1
2	10/02/2020	H01.00	4	Blefaritis	1
1	10/02/2020	H43.1	7	Hemovítreo	1

2	10/02/2020	H11.3	0	Hiposfagma	1
2	10/02/2020	H11.3	0	Hiposfagma	1
2	10/02/2020	H10.3	0	Conjuntivitis	1
2	10/02/2020	H53	11	No patología	1
1	10/02/2020	H10.3	0	Conjuntivitis	1
1	10/02/2020	H01.00	4	Blefaritis	1
1	10/02/2020	H40.05	3	HTO	1
2	10/02/2020	H16.9	1	Queratitis	1
2	10/02/2020	H35.30	8	DMAE	1
2	10/02/2020	G43.9	10	Migraña	1
2	10/02/2020	H35.30	8	DMAE	1
2	10/02/2020	H16.0	1	Úlcera corneal	1
1	10/02/2020	H10.3	0	Conjuntivitis	1
1	11/02/2020	H16.9	1	Queratitis	1
1	11/02/2020	H00.02	4	Orzuelo	1
1	11/02/2020	H43.81	7	DVP	1
2	11/02/2020	H02	4	Alteración palpebral	1
2	11/02/2020	H11.3	0	Hiposfagma	1
2	11/02/2020	H10.3	0	Conjuntivitis	1
2	11/02/2020	H35.30	8	DMAE	1
2	11/02/2020	H18.20	1	Edema corneal	1
1	11/02/2020	H11.3	0	Hiposfagma	1
2	11/02/2020	H01.00	4	Blefaritis	1
1	11/02/2020	H10.3	0	Conjuntivitis	1
2	11/02/2020	H35.3	8	Maculopatía	1
2	11/02/2020	H16.9	1	Queratitis	1
2	11/02/2020	H10.81	0	Pingueculitis	1
2	11/02/2020	H43.81	7	DVP	1
1	11/02/2020	H10.3	0	Conjuntivitis	1
1	11/02/2020	H02	4	Alteración palpebral	1
1	11/02/2020	H10.3	0	Conjuntivitis	1
2	11/02/2020	H16.9	1	Queratitis	1
2	11/02/2020	H00.02	4	Orzuelo	1
1	11/02/2020	T15.0	1	Cuerpo extraño	1
1	11/02/2020	H10.3	0	Conjuntivitis	1
1	11/02/2020	T15.0	1	Cuerpo extraño	1
2	11/02/2020	H01.00	4	Blefaritis	1
1	11/02/2020	S05.00	9	Abrasión conjuntival	1
1	11/02/2020	H16.9	1	Queratitis	1
1	11/02/2020	H00.02	4	Orzuelo	1
1	11/02/2020	T15.0	1	Cuerpo extraño	1
2	11/02/2020	H33.30	8	Desgarro retiniano	1
2	11/02/2020	H53.10	11	Problema refractivo	1
2	11/02/2020	H10.5	0	Blefaroconjuntivitis	1
2	11/02/2020	H16.0	1	Úlcera corneal	1
2	11/02/2020	H43.81	7	DVP	1
1	11/02/2020	H20	6	Uveitis	1
1	11/02/2020	H16.9	1	Queratitis	1
2	11/02/2020	H10.81	0	Pingueculitis	1
2	11/02/2020	H46	10	Neuritis óptica	1
2	11/02/2020	H16.9	1	Queratitis	1
1	11/02/2020	H16.9	1	Queratitis	1
1	11/02/2020	G43.109	10	Aura	1
2	11/02/2020	H40.9	3	Glaucoma	1
1	11/02/2020	H02	4	Alteración palpebral	1
1	11/02/2020	H11.44	0	Quiste conjuntival	1
1	11/02/2020	T15.0	1	Cuerpo extraño	1
2	12/02/2020	H16.0	1	Úlcera corneal	1
2	12/02/2020	H16.0	1	Úlcera corneal	1
1	12/02/2020	H16.9	1	Queratitis	1
2	12/02/2020	T15.0	1	Cuerpo extraño	1
2	12/02/2020	H16.0	1	Úlcera corneal	1
2	12/02/2020	H02	4	Alteración palpebral	1
1	12/02/2020	H35.30	8	DMAE	1
2	12/02/2020	H33.30	8	Desgarro retiniano	1
2	12/02/2020	H18.20	1	Edema corneal	1

2	12/02/2020	H16.9	1	Queratitis	1
2	12/02/2020	H11.3	0	Hiposfagma	1
1	12/02/2020	S05.9	9	Traumatismo	1
2	12/02/2020	H53	11	No patología	1
1	12/02/2020	H16.9	1	Queratitis	1
2	12/02/2020	H43.81	7	DVP	1
2	12/02/2020	H10.81	0	Pingueculitis	1
1	12/02/2020	H10.3	0	Conjuntivitis	1
2	12/02/2020	B00.1	4	Dermatitis herpética	1
1	12/02/2020	H33.30	8	Desgarro retiniano	1
1	12/02/2020	H00.02	4	Orzuelo	1
2	12/02/2020	H43.81	7	DVP	1
2	12/02/2020	H10.3	0	Conjuntivitis	1
1	12/02/2020	H53	11	No patología	1
2	12/02/2020	H0.41	1	Ojo seco	1
2	12/02/2020	H04.32	5	Dacriocistitis aguda	1
2	12/02/2020	H43.81	7	DVP	1
2	12/02/2020	H00.19	4	Chalazión	1
2	12/02/2020	H43.81	7	DVP	1
1	12/02/2020	H27.8	2	OCP	1
2	12/02/2020	H35.30	8	DMAE	1
2	12/02/2020	H16.9	1	Queratitis	1
1	12/02/2020	H11.44	0	Quiste conjuntival	1
2	12/02/2020	H11.3	0	Hiposfagma	1
1	12/02/2020	H53	11	No patología	1
1	12/02/2020	H16.0	1	Úlcera corneal	1
2	12/02/2020	H16.9	1	Queratitis	1
1	12/02/2020	H10.81	0	Pingueculitis	1
2	12/02/2020	H43.81	7	DVP	1
2	12/02/2020	H33.0	8	Desprendimiento de retina	1
1	12/02/2020	H20	6	Uveitis	1
2	12/02/2020	H10.3	0	Conjuntivitis	1
2	12/02/2020	H10.3	0	Conjuntivitis	1
2	12/02/2020	H20	6	Uveitis	1
2	12/02/2020	H43.81	7	DVP	1
2	12/02/2020	H0.41	1	Ojo seco	1
1	12/02/2020	H10.3	0	Conjuntivitis	1
1	12/02/2020	T15.0	1	Cuerpo extraño	1
2	12/02/2020	H04.32	5	Dacriocistitis aguda	1
2	12/02/2020	H10.3	0	Conjuntivitis	1
1	12/02/2020	H16.9	1	Queratitis	1
1	12/02/2020	H43.81	7	DVP	1
2	12/02/2020	H11.3	0	Hiposfagma	1
2	12/02/2020	H00.02	4	Orzuelo	1
2	12/02/2020	H33.30	8	Desgarro retiniano	1
1	12/02/2020	T15.0	1	Cuerpo extraño	1
2	12/02/2020	H16.9	1	Queratitis	1
1	12/02/2020	H16.9	1	Queratitis	1
2	12/02/2020	H11.3	0	Hiposfagma	1
1	12/02/2020	T15.0	1	Cuerpo extraño	1
1	12/02/2020	H16.0	1	Úlcera corneal	1
2	12/02/2020	H16.9	1	Queratitis	1
1	13/02/2020	H16.0	1	Úlcera corneal	1
1	13/02/2020	G43.9	10	Migraña	1
1	13/02/2020	H53	11	No patología	1
1	13/02/2020	H02	4	Alteración palpebral	1
2	13/02/2020	H53	11	No patología	1
1	13/02/2020	H11.3	0	Hiposfagma	1
2	13/02/2020	H10.3	0	Conjuntivitis	1
2	13/02/2020	H01.00	4	Blefaritis	1
2	13/02/2020	H10.3	0	Conjuntivitis	1
2	13/02/2020	H04.32	5	Dacriocistitis aguda	1
2	13/02/2020	H10.3	0	Conjuntivitis	1
1	13/02/2020	H11.3	0	Hiposfagma	1
1	13/02/2020	H10.3	0	Conjuntivitis	1
2	13/02/2020	H01.00	4	Blefaritis	1

2	13/02/2020	H10.3	0	Conjuntivitis	1
2	13/02/2020	H11.3	0	Hiposfagma	1
1	13/02/2020	H43.81	7	DVP	1
2	13/02/2020	H10.3	0	Conjuntivitis	1
1	13/02/2020	S05.9	9	Traumatismo	1
2	13/02/2020	H16.9	1	Queratitis	1
2	13/02/2020	H10.3	0	Conjuntivitis	1
1	13/02/2020	H40.05	3	HTO	1
2	13/02/2020	H43.81	7	DVP	1
2	13/02/2020	H16.9	1	Queratitis	1
2	13/02/2020	B00.1	4	Dermatitis herpética	1
2	13/02/2020	H40.9	3	Glaucoma	1
2	13/02/2020	H35.3	8	Maculopatía	1
2	13/02/2020	H10.3	0	Conjuntivitis	1
1	13/02/2020	H01.00	4	Blefaritis	1
1	13/02/2020	H02	4	Alteración palpebral	1
1	13/02/2020	H53	11	No patología	1
1	13/02/2020	H00.02	4	Orzuelo	1
1	13/02/2020	H16.9	1	Queratitis	1
1	13/02/2020	T15.0	1	Cuerpo extraño	1
1	13/02/2020	H00.02	4	Orzuelo	1
2	13/02/2020	H10.3	0	Conjuntivitis	1
2	13/02/2020	H16.0	1	Úlcera corneal	1
2	13/02/2020	H16.0	1	Úlcera corneal	1
1	13/02/2020	H35.30	8	DMAE	1
1	13/02/2020	H11.3	0	Hiposfagma	1
1	13/02/2020	H35.3	8	Maculopatía	1
2	13/02/2020	H00.02	4	Orzuelo	1
2	13/02/2020	H53	11	No patología	1
1	13/02/2020	T15.0	1	Cuerpo extraño	1
2	13/02/2020	T15.0	1	Cuerpo extraño	1
2	13/02/2020	H10.3	0	Conjuntivitis	1
2	13/02/2020	H16.9	1	Queratitis	1
1	13/02/2020	H16.0	1	Úlcera corneal	1
2	13/02/2020	H11.3	0	Hiposfagma	1
2	13/02/2020	S05.9	9	Traumatismo	1
1	13/02/2020	T15.0	1	Cuerpo extraño	1
1	13/02/2020	H01.00	4	Blefaritis	1
1	13/02/2020	T15.0	1	Cuerpo extraño	1
1	13/02/2020	T15.0	1	Cuerpo extraño	1
2	13/02/2020	H10.3	0	Conjuntivitis	1
1	13/02/2020	H11.3	0	Hiposfagma	1
1	13/02/2020	T15.0	1	Cuerpo extraño	1
2	14/02/2020	H10.3	0	Conjuntivitis	1
2	14/02/2020	H15.00	6	Escleritis	1
2	14/02/2020	H35.3	8	Maculopatía	1
2	14/02/2020	T15.0	1	Cuerpo extraño	1
1	14/02/2020	H01.00	4	Blefaritis	1
2	14/02/2020	H04.32	5	Dacriocistitis aguda	1
2	14/02/2020	H11.3	0	Hiposfagma	1
2	14/02/2020	H16.0	1	Úlcera corneal	1
2	14/02/2020	H16.0	1	Úlcera corneal	1
2	14/02/2020	H01.00	4	Blefaritis	1
1	14/02/2020	H15.1	6	Epiescleritis	1
2	14/02/2020	H27.8	2	OCP	1
2	14/02/2020	H16.9	1	Queratitis	1
1	14/02/2020	H10.3	0	Conjuntivitis	1
2	14/02/2020	H43.81	7	DVP	1
2	14/02/2020	H01.00	4	Blefaritis	1
1	14/02/2020	H10.3	0	Conjuntivitis	1
2	14/02/2020	H10.3	0	Conjuntivitis	1
2	14/02/2020	H01.00	4	Blefaritis	1
2	14/02/2020	B00.1	4	Dermatitis herpética	1
2	14/02/2020	H53	11	No patología	1
1	14/02/2020	H27.8	2	OCP	1
1	14/02/2020	H53	11	No patología	1

2	14/02/2020	H10.3	0	Conjuntivitis	1
1	14/02/2020	H59.3	4	Post op párpados	1
2	14/02/2020	H01.00	4	Blefaritis	1
2	14/02/2020	H01.00	4	Blefaritis	1
2	14/02/2020	H16.0	1	Úlcera corneal	1
2	14/02/2020	H16.0	1	Úlcera corneal	1
2	14/02/2020	H16.9	1	Queratitis	1
2	14/02/2020	H11.3	0	Hiposfagma	1
2	14/02/2020	H27.8	2	OCP	1
1	14/02/2020	H16.9	1	Queratitis	1
2	14/02/2020	T26	9	Causticación	1
2	14/02/2020	T15.0	1	Cuerpo extraño	1
1	14/02/2020	S05.9	9	Traumatismo	1
1	15/02/2020	S05.9	9	Traumatismo	1
2	15/02/2020	T15.0	1	Cuerpo extraño	1
2	15/02/2020	H16.9	1	Queratitis	1
2	15/02/2020	S05.9	9	Traumatismo	1
1	15/02/2020	H18.20	1	Edema corneal	1
2	15/02/2020	H10.3	0	Conjuntivitis	1
2	15/02/2020	H04.32	5	Dacriocistitis aguda	1
2	15/02/2020	H10.3	0	Conjuntivitis	1
2	15/02/2020	Z98.4	2	Postoperatorio catarata	1
2	15/02/2020	H04.51	5	Dacriolito	1
2	15/02/2020	H11.3	0	Hiposfagma	1
2	15/02/2020	H53	11	No patología	1
2	15/02/2020	H16.9	1	Queratitis	1
2	15/02/2020	H53	11	No patología	1
1	15/02/2020	H11.3	0	Hiposfagma	1
2	15/02/2020	H16.9	1	Queratitis	1
2	15/02/2020	H40.9	3	Glaucoma	1
1	15/02/2020	H00.02	4	Orzuelo	1
2	15/02/2020	H16.9	1	Queratitis	1
1	15/02/2020	T15.0	1	Cuerpo extraño	1
1	15/02/2020	H31.42	8	Coroidopatía serosa central	1
2	15/02/2020	T15.0	1	Cuerpo extraño	1
2	15/02/2020	H04.41	5	Dacriocistitis crónica	1
2	15/02/2020	H11.3	0	Hiposfagma	1
1	15/02/2020	H00.02	4	Orzuelo	1
1	15/02/2020	H16.3	1	Abceso corneal	1
2	15/02/2020	H10.3	0	Conjuntivitis	1
2	15/02/2020	H33.30	8	Desgarro retiniano	1
1	15/02/2020	H10.81	0	Pingueculitis	1
1	15/02/2020	H53	11	No patología	1
1	15/02/2020	H16.0	1	Úlcera corneal	1
2	15/02/2020	H01.00	4	Blefaritis	1
2	15/02/2020	T15.0	1	Cuerpo extraño	1
1	15/02/2020	H10.3	0	Conjuntivitis	1
2	15/02/2020	H10.3	0	Conjuntivitis	1
2	15/02/2020	H10.3	0	Conjuntivitis	1
1	15/02/2020	H16.0	1	Úlcera corneal	1
2	15/02/2020	H10.3	0	Conjuntivitis	1
2	15/02/2020	H10.3	0	Conjuntivitis	1
1	15/02/2020	H16.0	1	Úlcera corneal	1
2	15/02/2020	H01.00	4	Blefaritis	1
1	15/02/2020	S05.9	9	Traumatismo	1
2	15/02/2020	H00.19	4	Chalazión	1
1	15/02/2020	H43.81	7	DVP	1
2	15/02/2020	H10.3	0	Conjuntivitis	1
2	15/02/2020	Alta por fuga	11	Alta por fuga	1
2	15/02/2020	H11.3	0	Hiposfagma	1
2	15/02/2020	H01.00	4	Blefaritis	1
2	15/02/2020	H16.0	1	Úlcera corneal	1
1	15/02/2020	H59.3	4	Post op párpados	1
2	15/02/2020	H16.9	1	Queratitis	1
1	15/02/2020	S05.9	9	Traumatismo	1
2	16/02/2020	H59.3	4	Post op párpados	1

2	16/02/2020	H16.9	1	Queratitis	1
2	16/02/2020	H01.00	4	Blefaritis	1
2	16/02/2020	H16.9	1	Queratitis	1
2	16/02/2020	H0.41	1	Ojo seco	1
1	16/02/2020	H26.9	2	Catarata	1
2	16/02/2020	S05.6	9	Perforación ocular	1
2	16/02/2020	H20	6	Uveitis	1
2	16/02/2020	H16.9	1	Queratitis	1
2	16/02/2020	H20	6	Uveitis	1
2	16/02/2020	H16.9	1	Queratitis	1
2	16/02/2020	H01.00	4	Blefaritis	1
2	16/02/2020	H10.3	0	Conjuntivitis	1
1	16/02/2020	T26	9	Causticación	1
2	16/02/2020	H10.3	0	Conjuntivitis	1
2	16/02/2020	H00.02	4	Orzuelo	1
2	16/02/2020	H10.3	0	Conjuntivitis	1
2	16/02/2020	H10.3	0	Conjuntivitis	1
2	16/02/2020	H0.41	1	Ojo seco	1
1	16/02/2020	T15.0	1	Cuerpo extraño	1
2	16/02/2020	S05.9	9	Traumatismo	1
1	16/02/2020	H16.9	1	Queratitis	1
1	16/02/2020	H11.3	0	Hiposfagma	1
1	16/02/2020	H10.3	0	Conjuntivitis	1
1	16/02/2020	H16.9	1	Queratitis	1
2	16/02/2020	H10.3	0	Conjuntivitis	1
2	16/02/2020	H0.41	1	Ojo seco	1
2	16/02/2020	H33.0	8	Desprendimiento de retina	1
2	16/02/2020	H00.03	4	Celulitis preseptal	1
2	16/02/2020	H04.32	5	Dacriocistitis aguda	1
2	16/02/2020	H16.0	1	Úlcera corneal	1
2	16/02/2020	T26	9	Causticación	1
2	16/02/2020	H16.9	1	Queratitis	1
2	16/02/2020	H20	6	Uveitis	1
1	16/02/2020	H10.3	0	Conjuntivitis	1
1	16/02/2020	S05.9	9	Traumatismo	1
2	16/02/2020	H15.00	6	Escleritis	1
2	16/02/2020	H16.9	1	Queratitis	1
1	16/02/2020	H16.0	1	Úlcera corneal	1
1	17/02/2020	H10.3	0	Conjuntivitis	1
1	17/02/2020	T15.0	1	Cuerpo extraño	1
1	17/02/2020	H10.3	0	Conjuntivitis	1
2	17/02/2020	H11.3	0	Hiposfagma	1
2	17/02/2020	H43.81	7	DVP	1
2	17/02/2020	H01.00	4	Blefaritis	1
2	17/02/2020	H10.3	0	Conjuntivitis	1
1	17/02/2020	H16.0	1	úlcera corneal	1
2	17/02/2020	H10.3	0	Conjuntivitis	1
2	17/02/2020	H53	11	No patología	1
2	17/02/2020	H35.3	8	Maculopatía	1
2	17/02/2020	B00.1	4	Dermatitis herpética	1
1	17/02/2020	H16.9	1	Queratitis	1
2	17/02/2020	H10.3	0	Conjuntivitis	1
2	17/02/2020	H35.3	8	Maculopatía	1
2	17/02/2020	H43.81	7	DVP	1
2	17/02/2020	H43.81	7	DVP	1
2	17/02/2020	H10.3	0	Conjuntivitis	1
2	17/02/2020	G43.109	10	Aura	1
2	17/02/2020	H11.82	0	Conjuntivocalasia	1
2	17/02/2020	H43.81	7	DVP	1
2	17/02/2020	H17.8	1	Infiltrados corneales	1
2	17/02/2020	T15.0	1	Cuerpo extraño	1
1	17/02/2020	H20	6	Uveitis	1
2	17/02/2020	H10.3	0	Conjuntivitis	1
1	17/02/2020	H10.3	0	Conjuntivitis	1
1	17/02/2020	H17.8	1	Infiltrados corneales	1
1	17/02/2020	T15.0	1	Cuerpo extraño	1

2	17/02/2020	H53	11	No patología	1
2	17/02/2020	H35.3	8	Maculopatía	1
2	17/02/2020	H10.3	0	Conjuntivitis	1
2	17/02/2020	H10.3	0	Conjuntivitis	1
1	17/02/2020	H10.3	0	Conjuntivitis	1
1	17/02/2020	H53	11	No patología	1
1	17/02/2020	H16.0	1	Úlcera corneal	1
2	17/02/2020	H10.3	0	Conjuntivitis	1
1	17/02/2020	T15.0	1	Cuerpo extraño	1
1	17/02/2020	H20	9	Uveitis	1
1	17/02/2020	H00.02	4	Orzuelo	1
1	17/02/2020	S05.9	9	Traumatismo	1
2	17/02/2020	H53	11	No patología	1
1	17/02/2020	S05.9	9	Traumatismo	1
1	17/02/2020	T15.0	1	Cuerpo extraño	1
2	17/02/2020	H53	11	No patología	1
2	18/02/2020	H20	6	Uveitis	1
2	18/02/2020	H16.0	1	úlcera corneal	1
2	18/02/2020	H53	11	No patología	1
1	18/02/2020	H53	11	No patología	1
1	18/02/2020	H16.9	1	Queratitis	1
2	18/02/2020	H10.3	0	Conjuntivitis	1
2	18/02/2020	H43.81	7	DVP	1
1	18/02/2020	H01.00	4	Blefaritis	1
1	18/02/2020	H16.9	1	Queratitis	1
2	18/02/2020	B58.0	8	Toxoplasmosis	1
2	18/02/2020	H40.05	3	HTO	1
1	18/02/2020	H16.9	1	Queratitis	1
1	18/02/2020	H11.3	0	Hiposfagma	1
2	18/02/2020	H0.41	1	Ojo seco	1
1	18/02/2020	H33.30	8	Desgarro retiniano	1
2	18/02/2020	H43.81	7	DVP	1
2	18/02/2020	H02	4	Alteración palpebral	1
1	18/02/2020	H35.3	8	Maculopatía	1
1	18/02/2020	H53	11	No patología	1
2	18/02/2020	H11.3	0	Hiposfagma	1
2	18/02/2020	H10.3	0	Conjuntivitis	1
2	18/02/2020	H43.81	7	DVP	1
1	18/02/2020	S05.9	9	Traumatismo	1
1	18/02/2020	H20	6	Uveitis	1
1	18/02/2020	H16.9	1	Queratitis	1
2	18/02/2020	H20	6	Uveitis	1
2	18/02/2020	H16.9	1	Queratitis	1
1	18/02/2020	H10.3	0	Conjuntivitis	1
1	18/02/2020	H20	6	Uveitis	1
2	18/02/2020	H43.81	7	DVP	1
2	18/02/2020	H00.02	4	Orzuelo	1
2	18/02/2020	H16.9	1	Queratitis	1
1	18/02/2020	H11.3	0	Hiposfagma	1
2	18/02/2020	H27.8	2	OCP	1
2	18/02/2020	H16.9	1	Queratitis	1
2	18/02/2020	H10.3	0	Conjuntivitis	1
2	18/02/2020	H46	10	Neuritis óptica	1
1	18/02/2020	S05.9	9	Traumatismo	1
1	18/02/2020	H10.3	0	Conjuntivitis	1
2	18/02/2020	H26.9	2	Catarata	1
1	18/02/2020	H11.3	0	Hiposfagma	1
2	18/02/2020	H01.00	4	Blefaritis	1
1	18/02/2020	H16.9	1	Queratitis	1
1	18/02/2020	H16.9	1	Queratitis	1
2	18/02/2020	H04.32	5	Dacriocistitis aguda	1
2	18/02/2020	H10.3	0	Conjuntivitis	1
2	19/02/2020	H16.9	1	Queratitis	1
2	19/02/2020	H16.9	1	Queratitis	1
2	19/02/2020	G43.109	11	Aura	1
2	19/02/2020	H16.9	1	Queratitis	1

1	19/02/2020	H53	11	No patología	1
2	19/02/2020	H16.9	1	Queratitis	1
1	19/02/2020	H27.1	2	Luxación catarata	1
2	19/02/2020	H18.20	1	Edema corneal	1
1	19/02/2020	H16.9	1	Queratitis	1
2	19/02/2020	H16.9	1	Queratitis	1
1	19/02/2020	H10.3	0	Conjuntivitis	1
2	19/02/2020	H43.81	7	DVP	1
1	19/02/2020	H26.9	2	Catarata	1
2	19/02/2020	H16.9	1	Queratitis	1
1	19/02/2020	H11.3	0	Hiposfagma	1
2	19/02/2020	H15.00	6	Escleritis	1
1	19/02/2020	H20	6	Uveitis	1
2	19/02/2020	H10.3	0	Conjuntivitis	1
1	19/02/2020	S05.9	9	Traumatismo	1
2	19/02/2020	H10.3	0	Conjuntivitis	1
2	19/02/2020	Alta por fuga	11	Alta por fuga	1
2	19/02/2020	H02	4	Lesión palpebral	1
2	19/02/2020	H16.9	1	Queratitis	1
2	19/02/2020	H16.0	1	Úlcera corneal	1
1	19/02/2020	T15.0	1	Cuerpo extraño	1
2	19/02/2020	H43.81	7	DVP	1
2	19/02/2020	H16.9	1	Queratitis	1
2	19/02/2020	H01.00	4	Blefaritis	1
2	19/02/2020	H43.81	7	DVP	1
2	19/02/2020	H18.20	1	Edema corneal	1
2	19/02/2020	H16.9	1	Queratitis	1
1	19/02/2020	H10.3	0	Conjuntivitis	1
2	19/02/2020	H10.3	0	Conjuntivitis	1
1	19/02/2020	S05.9	9	Traumatismo	1
1	19/02/2020	H17.8	1	Infiltrados corneales	1
2	19/02/2020	H16.0	1	Úlcera corneal	1
1	19/02/2020	H10.3	0	Conjuntivitis	1
2	19/02/2020	H00.19	4	Chalazión	1
2	19/02/2020	H00.02	4	Orzuelo	1
1	19/02/2020	H16.9	1	Queratitis	1
1	19/02/2020	H10.3	0	Conjuntivitis	1
1	19/02/2020	H16.0	1	Úlcera corneal	1
1	19/02/2020	T15.0	1	Cuerpo extraño	1
2	19/02/2020	H20	6	Uveitis	1
1	19/02/2020	H10.3	0	Conjuntivitis	1
2	19/02/2020	H02	4	Alteración palpebral	1
1	19/02/2020	S05.9	9	Traumatismo	1
1	19/02/2020	H10.3	0	Conjuntivitis	1
1	19/02/2020	H17.8	1	Infiltrados corneales	1
1	19/02/2020	H00.02	4	Orzuelo	1
1	19/02/2020	H16.9	1	Queratitis	1
2	19/02/2020	H53	11	No patología	1
2	19/02/2020	H10.3	0	Conjuntivitis	1
1	20/02/2020	H10.3	0	Conjuntivitis	1
1	20/02/2020	H16.9	1	Queratitis	1
1	20/02/2020	H17.8	1	Infiltrados corneales	1
1	20/02/2020	H16.9	1	Queratitis	1
2	20/02/2020	H11.3	0	Hiposfagma	1
1	20/02/2020	B00.1	4	Dermatitis herpética	1
1	20/02/2020	H53	11	No patología	1
2	20/02/2020	H43.81	7	DVP	1
2	20/02/2020	H00.02	4	Orzuelo	1
1	20/02/2020	H10.3	0	Conjuntivitis	1
2	20/02/2020	G43.9	10	Migraña	1
2	20/02/2020	H00.02	4	Orzuelo	1
2	20/02/2020	H0.41	1	Ojo seco	1
2	20/02/2020	H00.02	4	Orzuelo	1
2	20/02/2020	H0.41	1	Ojo seco	1
2	20/02/2020	H16.9	1	Queratitis	1
1	20/02/2020	H00.02	4	Orzuelo	1

2	20/02/2020	S05.6	9	Perforación ocular	1
2	20/02/2020	H15.1	6	Epiescleritis	1
1	20/02/2020	H16.9	1	Queratitis	1
1	20/02/2020	H35.0	8	Hemorragia retiniana	1
2	20/02/2020	H16.9	1	Queratitis	1
2	20/02/2020	H20	6	Uveitis	1
1	20/02/2020	T15.0	1	Cuerpo extraño	1
1	20/02/2020	H10.3	0	Conjuntivitis	1
2	20/02/2020	H43.81	7	DVP	1
1	20/02/2020	H11.3	0	Hiposfagma	1
2	20/02/2020	H43.81	7	DVP	1
2	20/02/2020	H35.3	8	Maculopatía	1
2	20/02/2020	H10.3	0	Conjuntivitis	1
2	20/02/2020	H53	11	No patología	1
1	20/02/2020	S05.9	9	Traumatismo	1
2	20/02/2020	H16.9	1	Queratitis	1
2	20/02/2020	H16.9	1	Queratitis	1
2	20/02/2020	H0.41	1	Ojo seco	1
1	20/02/2020	H10.3	0	Conjuntivitis	1
1	20/02/2020	H01.00	4	Blefaritis	1
2	20/02/2020	H16.9	1	Queratitis	1
2	20/02/2020	H33.0	8	Desprendimiento de retina	1
1	20/02/2020	H15.1	6	Epiescleritis	1
2	20/02/2020	H10.3	0	Conjuntivitis	1
2	20/02/2020	H10.3	0	Conjuntivitis	1
1	20/02/2020	H16.0	1	Úlcera corneal	1
1	20/02/2020	H16.0	1	Úlcera corneal	1
1	20/02/2020	H10.3	0	Conjuntivitis	1
1	20/02/2020	Alta por fuga	11	Alta por fuga	1
2	20/02/2020	H16.0	1	úlcera corneal	1
2	20/02/2020	S05.9	9	Traumatismo	1
2	21/02/2020	H10.3	0	Conjuntivitis	1
1	21/02/2020	H16.0	1	Úlcera corneal	1
2	21/02/2020	H16.9	1	Queratitis	1
1	21/02/2020	H43.81	7	DVP	1
2	21/02/2020	H16.0	1	Úlcera corneal	1
1	21/02/2020	H16.0	1	Úlcera corneal	1
2	21/02/2020	H10.3	0	Conjuntivitis	1
2	21/02/2020	H01.00	4	Blefaritis	1
2	21/02/2020	S05.6	9	Perforación ocular	1
2	21/02/2020	H02	4	Alteración palpebral	1
2	21/02/2020	S05.6	1	Perforación ocular	1
1	21/02/2020	H53	11	No patología	1
2	21/02/2020	H34.82	8	Trombosis venosa	1
2	21/02/2020	H01.00	4	Blefaritis	1
2	21/02/2020	H11.3	0	Hiposfagma	1
1	21/02/2020	H11.3	0	Hiposfagma	1
2	21/02/2020	H11.3	0	Hiposfagma	1
2	21/02/2020	H43.81	7	DVP	1
2	21/02/2020	H01.00	4	Blefaritis	1
1	21/02/2020	H00.02	4	Orzuelo	1
1	21/02/2020	H11.3	0	Hiposfagma	1
2	21/02/2020	H34.82	8	Trombosis venosa	1
2	21/02/2020	H16.9	1	Queratitis	1
1	21/02/2020	H02	4	Alteración palpebral	1
2	21/02/2020	H10.3	0	Conjuntivitis	1
2	21/02/2020	H16.9	1	Queratitis	1
1	21/02/2020	H53	11	No patología	1
2	21/02/2020	H01.00	4	Blefaritis	1
1	21/02/2020	T15.0	1	Cuerpo extraño	1
1	21/02/2020	S05.9	9	Traumatismo	1
2	21/02/2020	H43.81	7	DVP	1
1	21/02/2020	H43.81	7	DVP	1
2	21/02/2020	H53	11	No patología	1
2	21/02/2020	H53	11	No patología	1
2	21/02/2020	H15.1	6	Epiescleritis	1

2	21/02/2020	H10.3	0	Conjuntivitis	1
1	21/02/2020	H16.9	1	Queratitis	1
2	21/02/2020	H10.81	0	Pingueculitis	1
1	21/02/2020	S05.9	9	Traumatismo	1
1	21/02/2020	H10.3	0	Conjuntivitis	1
1	21/02/2020	H16.9	1	Queratitis	1
2	21/02/2020	H16.9	1	Queratitis	1
1	21/02/2020	H00.02	4	Orzuelo	1
2	21/02/2020	H16.0	1	Úlcera corneal	1
2	21/02/2020	H01.00	4	Blefaritis	1
2	21/02/2020	H10.3	0	Conjuntivitis	1
2	21/02/2020	H11.3	0	Hiposfagma	1
2	21/02/2020	H16.9	1	Queratitis	1
2	21/02/2020	H11.3	0	Hiposfagma	1
2	21/02/2020	H0.41	1	Ojo seco	1
1	21/02/2020	T15.0	1	Cuerpo extraño	1
2	21/02/2020	H10.3	0	Conjuntivitis	1
2	21/02/2020	H53	11	No patología	1
1	21/02/2020	H0.41	1	Ojo seco	1
1	21/02/2020	S05.9	9	Traumatismo	1
2	21/02/2020	H10.3	0	Conjuntivitis	1
1	21/02/2020	Alta por fuga	11	Alta por fuga	1
1	21/02/2020	H16.0	1	Úlcera corneal	1
2	21/02/2020	H16.9	1	Queratitis	1
2	22/02/2020	H53	11	No patología	1
1	22/02/2020	H04.32	5	Dacriocistitis aguda	1
1	22/02/2020	H00.02	4	Orzuelo	1
2	22/02/2020	S05.9	9	Traumatismo	1
2	22/02/2020	H10.3	0	Conjuntivitis	1
1	22/02/2020	H16.0	1	Úlcera corneal	1
1	22/02/2020	H26.9	2	Catarata	1
2	22/02/2020	H10.3	0	Conjuntivitis	1
1	22/02/2020	H11.3	0	Hiposfagma	1
1	22/02/2020	H04.32	5	Dacriocistitis aguda	1
1	22/02/2020	H16.0	1	Úlcera corneal	1
2	22/02/2020	S05.30	9	Laceración conjuntival	1
1	22/02/2020	H53	11	No patología	1
2	22/02/2020	H16.0	1	Úlcera corneal	1
1	22/02/2020	H10.3	0	Conjuntivitis	1
2	22/02/2020	H16.9	1	Queratitis	1
1	22/02/2020	T15.0	1	Cuerpo extraño	1
1	22/02/2020	H16.9	1	Queratitis	1
1	22/02/2020	H00.02	4	Orzuelo	1
1	22/02/2020	H00.02	4	Orzuelo	1
2	22/02/2020	H11.3	0	Hiposfagma	1
1	22/02/2020	T15.0	1	Cuerpo extraño	1
2	22/02/2020	H10.3	0	Conjuntivitis	1
2	22/02/2020	H43.1	7	Hemovítreo	1
2	22/02/2020	G43.9	10	Migraña	1
1	22/02/2020	S05.9	9	Traumatismo	1
1	22/02/2020	H16.9	1	Queratitis	1
2	22/02/2020	H16.9	1	Queratitis	1
1	22/02/2020	H10.3	0	Conjuntivitis	1
1	22/02/2020	H10.3	0	Conjuntivitis	1
1	22/02/2020	H16.0	1	Úlcera corneal	1
2	22/02/2020	S05.9	9	Traumatismo	1
2	22/02/2020	H00.02	4	Orzuelo	1
2	22/02/2020	H01.00	4	Blefaritis	1
1	22/02/2020	T15.0	1	Cuerpo extraño	1
2	22/02/2020	H16.0	1	Úlcera corneal	1
1	22/02/2020	H00.02	4	Orzuelo	1
1	22/02/2020	H16.0	1	Úlcera corneal	1
1	22/02/2020	T15.0	1	Cuerpo extraño	1
2	22/02/2020	S05.9	9	Traumatismo	1
2	22/02/2020	H10.3	0	Conjuntivitis	1
2	22/02/2020	H11.3	0	Hiposfagma	1

1	22/02/2020	H10.3	0	Conjuntivitis	1
2	22/02/2020	H10.3	0	Conjuntivitis	1
2	22/02/2020	H00.02	4	Orzuelo	1
1	22/02/2020	H00.02	4	Orzuelo	1
2	22/02/2020	H01.00	4	Blefaritis	1
1	22/02/2020	H10.3	0	Conjuntivitis	1
1	22/02/2020	H16.9	1	Queratitis	1
1	22/02/2020	S05.9	9	Traumatismo	1
2	22/02/2020	H16.9	1	Queratitis	1
2	22/02/2020	H10.3	0	Conjuntivitis	1
2	22/02/2020	H01.00	4	Blefaritis	1
1	23/02/2020	T15.0	1	Cuerpo extraño	1
1	23/02/2020	T15.0	1	Cuerpo extraño	1
2	23/02/2020	H00.02	4	Orzuelo	1
1	23/02/2020	H10.3	0	Conjuntivitis	1
2	23/02/2020	H16.0	1	Úlcera corneal	1
2	23/02/2020	H43.81	7	DVP	1
2	23/02/2020	H10.3	0	Conjuntivitis	1
2	23/02/2020	H16.9	1	Queratitis	1
2	23/02/2020	H10.3	0	Conjuntivitis	1
2	23/02/2020	H10.3	0	Conjuntivitis	1
2	23/02/2020	H16.0	1	Úlcera corneal	1
2	23/02/2020	H00.02	4	Orzuelo	1
1	23/02/2020	H17.8	1	Infiltrados corneales	1
2	23/02/2020	H01.00	4	Blefaritis	1
1	23/02/2020	H18.20	1	Edema corneal	1
1	23/02/2020	H01.00	4	Blefaritis	1
2	23/02/2020	H20	6	Uveitis	1
1	23/02/2020	H01.00	4	Blefaritis	1
1	23/02/2020	H16.9	1	Queratitis	1
1	23/02/2020	H16.0	1	Úlcera corneal	1
1	23/02/2020	H16.9	1	Queratitis	1
1	23/02/2020	H01.00	4	Blefaritis	1
1	23/02/2020	H16.0	1	Úlcera corneal	1
1	23/02/2020	T26	9	Causticación	1
1	23/02/2020	H00.02	4	Orzuelo	1
2	23/02/2020	H43.81	7	DVP	1
1	23/02/2020	T15.0	0	Cuerpo extraño	1
2	23/02/2020	H15.1	6	Epiescleritis	1
2	23/02/2020	H16.9	1	Queratitis	1
2	23/02/2020	H0.41	1	Ojo seco	1
2	23/02/2020	H43.81	7	DVP	1
2	23/02/2020	H43.81	7	DVP	1
2	23/02/2020	G43.9	10	Migraña	1
1	23/02/2020	H16.0	1	Úlcera corneal	1
2	23/02/2020	H11.3	0	Hiposfagma	1
2	23/02/2020	Alta por fuga	11	Alta por fuga	1
1	23/02/2020	H16.0	1	Úlcera corneal	1
1	23/02/2020	H10.3	0	Conjuntivitis	1
2	23/02/2020	H10.3	0	Conjuntivitis	1
1	24/02/2020	H16.9	1	Queratitis	1
1	24/02/2020	H00.03	4	Celulitis preseptal	1
1	24/02/2020	H02.05	4	Distiquiasis	1
2	24/02/2020	H16.9	1	Queratitis	1
2	24/02/2020	H01.00	4	Blefaritis	1
1	24/02/2020	S05.9	9	Traumatismo	1
1	24/02/2020	H16.0	1	Úlcera corneal	1
2	24/02/2020	H11.3	0	Hiposfagma	1
2	24/02/2020	H11.82	0	Conjuntivocalasia	1
1	24/02/2020	H43.81	7	DVP	1
1	24/02/2020	H00.02	4	Orzuelo	1
2	24/02/2020	H11.3	0	Hiposfagma	1
2	24/02/2020	H20	6	Uveitis	1
2	24/02/2020	H16.9	1	Queratitis	1
1	24/02/2020	H16.9	1	Queratitis	1
2	24/02/2020	H0.41	1	Ojo seco	1

2	24/02/2020	H0.41	1	Ojo seco	1
2	24/02/2020	H16.9	1	Queratitis	1
1	24/02/2020	H43.1	7	Hemovítreo	1
1	24/02/2020	T15.0	1	Cuerpo extraño	1
1	24/02/2020	H16.9	1	Queratitis	1
2	24/02/2020	H10.3	0	Conjuntivitis	1
2	24/02/2020	H10.3	0	Conjuntivitis	1
2	24/02/2020	H11.3	0	Hiposfagma	1
2	24/02/2020	H43.81	7	DVP	1
1	24/02/2020	T15.0	1	Cuerpo extraño	1
2	24/02/2020	G43.9	10	Migraña	1
2	24/02/2020	H27.8	2	OCP	1
2	24/02/2020	H43.81	7	DVP	1
2	24/02/2020	H16.9	1	Queratitis	1
1	24/02/2020	H35.3	8	Maculopatía	1
2	24/02/2020	H11.3	0	Hiposfagma	1
2	24/02/2020	B00.1	4	Dermatitis herpética	1
1	24/02/2020	S05.9	9	Traumatismo	1
2	24/02/2020	H53	11	No patología	1
2	24/02/2020	H43.81	7	DVP	1
2	24/02/2020	H43.81	7	DVP	1
2	24/02/2020	Alta por fuga	11	Alta por fuga	1
2	24/02/2020	H10.3	0	Conjuntivitis	1
2	24/02/2020	H43.81	7	DVP	1
2	24/02/2020	H43.81	7	DVP	1
2	24/02/2020	T15.0	1	Cuerpo extraño	1
2	24/02/2020	H10.3	0	Conjuntivitis	1
1	24/02/2020	T15.0	1	Cuerpo extraño	1
2	24/02/2020	H16.9	1	Queratitis	1
1	24/02/2020	H16.9	1	Queratitis	1
1	24/02/2020	H00.03	4	Celulitis preseptal	1
2	24/02/2020	S05.9	9	Traumatismo	1
1	24/02/2020	H16.0	1	Úlcera corneal	1
1	24/02/2020	H16.0	1	Úlcera corneal	1
2	24/02/2020	H0.41	1	Ojo seco	1
2	24/02/2020	G43.9	10	Migraña	1
2	24/02/2020	H16.9	1	Queratitis	1
1	24/02/2020	H16.9	1	Queratitis	1
1	24/02/2020	S05.9	9	Traumatismo	1
2	24/02/2020	H16.0	1	Úlcera corneal	1
1	24/02/2020	H11.44	0	Quiste conjuntival	1
1	25/02/2020	H02	4	Alteración palpebral	1
2	25/02/2020	H16.9	1	Queratitis	1
2	25/02/2020	G43.9	10	Migraña	1
1	25/02/2020	H16.0	1	Úlcera corneal	1
2	25/02/2020	H16.9	1	Queratitis	1
2	25/02/2020	T15.0	1	Cuerpo extraño	1
2	25/02/2020	H35.30	8	DMAE	1
1	25/02/2020	H10.3	0	Conjuntivitis	1
1	25/02/2020	H01.00	4	Blefaritis	1
2	25/02/2020	H43.81	7	DVP	1
1	25/02/2020	T15.0	1	Cuerpo extraño	1
2	25/02/2020	H43.81	7	DVP	1
2	25/02/2020	H16.9	1	Queratitis	1
2	25/02/2020	H16.9	1	Queratitis	1
2	25/02/2020	H16.9	1	Queratitis	1
1	25/02/2020	H16.0	1	Úlcera corneal	1
1	25/02/2020	H53	11	No patología	1
2	25/02/2020	H10.3	0	Conjuntivitis	1
2	25/02/2020	H43.81	7	DVP	1
2	25/02/2020	H11.3	0	Hiposfagma	1
1	25/02/2020	H11.3	0	Hiposfagma	1
2	25/02/2020	H16.9	1	Queratitis	1
2	25/02/2020	E11.319	8	Retinopatía diabética	1
2	25/02/2020	H11.44	0	Quiste conjuntival	1
1	25/02/2020	T15.0	1	Cuerpo extraño	1

1	25/02/2020	H34.82	8	Trombosis venosa	1
2	25/02/2020	H35.37	8	MER	1
2	25/02/2020	H20	6	Uveitis	1
2	25/02/2020	H43.81	7	DVP	1
2	25/02/2020	H27.8	2	OCP	1
1	25/02/2020	H10.3	0	Conjuntivitis	1
2	25/02/2020	H16.9	1	Queratitis	1
2	25/02/2020	H10.3	0	Conjuntivitis	1
2	25/02/2020	H43.81	7	DVP	1
2	25/02/2020	H16.9	1	Queratitis	1
2	25/02/2020	H10.3	0	Conjuntivitis	1
1	25/02/2020	H11.3	0	Hiposfagma	1
2	25/02/2020	H46	10	Neuritis óptica	1
1	25/02/2020	H43.81	7	DVP	1
1	25/02/2020	H10.3	0	Conjuntivitis	1
2	25/02/2020	T15.0	1	Cuerpo extraño	1
1	25/02/2020	H11.3	0	Hiposfagma	1
1	25/02/2020	T15.0	1	Cuerpo extraño	1
1	26/02/2020	T15.0	1	Cuerpo extraño	1
1	26/02/2020	H16.0	1	Úlcera corneal	1
2	26/02/2020	T15.0	1	Cuerpo extraño	1
2	26/02/2020	S05.9	9	Traumatismo	1
1	26/02/2020	H16.0	1	Úlcera corneal	1
1	26/02/2020	H10.3	0	Conjuntivitis	1
2	26/02/2020	H43.81	7	DVP	1
2	26/02/2020	H16.0	1	Úlcera corneal	1
2	26/02/2020	H43.81	7	DVP	1
2	26/02/2020	H16.9	1	Queratitis	1
1	26/02/2020	H10.3	0	Conjuntivitis	1
2	26/02/2020	H0.41	1	Ojo seco	1
2	26/02/2020	H35.30	8	DMAE	1
1	26/02/2020	H10.3	0	Conjuntivitis	1
1	26/02/2020	H33.0	8	Desprendimiento de retina	1
1	26/02/2020	H0.41	1	Ojo seco	1
1	26/02/2020	H10.3	0	Conjuntivitis	1
2	26/02/2020	H15.1	6	Epiescleritis	1
2	26/02/2020	H43.81	7	DVP	1
1	26/02/2020	T15.0	1	Cuerpo extraño	1
1	26/02/2020	Alta por fuga	11	Alta por fuga	1
2	26/02/2020	H10.3	0	Conjuntivitis	1
2	26/02/2020	H16.0	1	Úlcera corneal	1
2	26/02/2020	H16.9	1	Queratitis	1
2	26/02/2020	H16.0	1	Úlcera corneal	1
1	26/02/2020	S05.9	9	Traumatismo	1
2	26/02/2020	H43.81	7	DVP	1
1	26/02/2020	S05.9	9	Traumatismo	1
2	26/02/2020	H02	4	Alteración palpebral	1
2	26/02/2020	H10.3	0	Conjuntivitis	1
2	26/02/2020	H16.9	1	Queratitis	1
2	26/02/2020	H16.0	1	Úlcera corneal	1
1	26/02/2020	H02	4	Alteración palpebral	1
1	26/02/2020	T15.0	1	Cuerpo extraño	1
1	26/02/2020	H10.3	0	Conjuntivitis	1
2	26/02/2020	H16.0	1	Úlcera corneal	1
2	26/02/2020	S05.9	9	Traumatismo	1
1	26/02/2020	H00.02	4	Orzuelo	1
2	26/02/2020	H00.19	4	Chalazión	1
1	26/02/2020	T15.0	1	Cuerpo extraño	1
2	26/02/2020	S05.9	9	Traumatismo	1
2	26/02/2020	H10.3	0	Conjuntivitis	1
1	26/02/2020	H20	6	Uveitis	1
1	26/02/2020	H16.0	1	Úlcera corneal	1
2	26/02/2020	H20	6	Uveitis	1
1	26/02/2020	S05.9	9	Traumatismo	1
2	27/02/2020	H00.02	4	Orzuelo	1
1	27/02/2020	H16.9	1	Queratitis	1

2	27/02/2020	H16.0	1	Úlcera corneal	1
1	27/02/2020	H11.3	0	Hiposfagma	1
2	27/02/2020	H53.10	11	Problema refractivo	1
1	27/02/2020	H10.3	0	Conjuntivitis	1
2	27/02/2020	H01.00	4	Blefaritis	1
1	27/02/2020	H10.3	0	Conjuntivitis	1
1	27/02/2020	H10.3	0	Conjuntivitis	1
2	27/02/2020	H16.9	1	Queratitis	1
2	27/02/2020	H01.00	4	Blefaritis	1
2	27/02/2020	H10.81	0	Pingueculitis	1
1	27/02/2020	H10.3	0	Conjuntivitis	1
1	27/02/2020	H10.3	0	Conjuntivitis	1
2	27/02/2020	H43.81	7	DVP	1
2	27/02/2020	H33.30	8	Desgarro retiniano	1
2	27/02/2020	H33.30	8	Desgarro retiniano	1
1	27/02/2020	H10.3	0	Conjuntivitis	1
2	27/02/2020	H10.3	0	Conjuntivitis	1
2	27/02/2020	H02	4	Alteración palpebral	1
1	27/02/2020	B00.1	4	Dermatitis herpética	1
2	27/02/2020	H43.81	7	DVP	1
2	27/02/2020	H16.9	1	Queratitis	1
2	27/02/2020	H53	11	No patología	1
2	27/02/2020	H16.9	1	Queratitis	1
2	27/02/2020	G43.9	10	Migraña	1
1	27/02/2020	E11.319	8	Retinopatía diabética	1
1	27/02/2020	H10.3	0	Conjuntivitis	1
1	27/02/2020	H15.1	6	Epiescleritis	1
1	27/02/2020	H16.9	1	Queratitis	1
2	27/02/2020	H43.81	7	DVP	1
1	27/02/2020	T15.0	1	Cuerpo extraño	1
1	27/02/2020	H16.0	1	Úlcera corneal	1
2	27/02/2020	H16.0	1	Úlcera corneal	1
2	27/02/2020	H10.3	0	Conjuntivitis	1
2	27/02/2020	G43.9	10	Migraña	1
1	27/02/2020	H11.3	0	Hiposfagma	1
1	27/02/2020	H10.3	0	Conjuntivitis	1
1	27/02/2020	G43.9	10	Migraña	1
1	27/02/2020	S05.00	9	Abrasión conjuntival	1
1	28/02/2020	S05.9	9	Traumatismo	1
1	28/02/2020	H16.9	1	Queratitis	1
1	28/02/2020	H10.3	0	Conjuntivitis	1
2	28/02/2020	H53	11	No patología	1
1	28/02/2020	H15.00	6	Escleritis	1
2	28/02/2020	H01.00	4	Blefaritis	1
2	28/02/2020	H10.3	0	Conjuntivitis	1
2	28/02/2020	H10.3	0	Conjuntivitis	1
1	28/02/2020	B00.1	4	Dermatitis herpética	1
2	28/02/2020	B00.1	4	Dermatitis herpética	1
1	28/02/2020	H18.20	1	Edema corneal	1
2	28/02/2020	H10.3	0	Conjuntivitis	1
1	28/02/2020	H01.00	4	Blefaritis	1
1	28/02/2020	H35.30	8	DMAE	1
1	28/02/2020	H49.9	10	Parálisis oculomotora	1
1	28/02/2020	H01.00	4	Blefaritis	1
1	28/02/2020	H02	4	Alteración palpebral	1
2	28/02/2020	H11.3	0	Hiposfagma	1
2	28/02/2020	H01.00	4	Blefaritis	1
2	28/02/2020	H02	4	Alteración palpebral	1
1	28/02/2020	H26.9	2	Catarata	1
2	28/02/2020	H43.81	7	DVP	1
2	28/02/2020	H16.9	1	Queratitis	1
2	28/02/2020	H16.9	1	Queratitis	1
1	28/02/2020	T15.0	1	Cuerpo extraño	1
2	28/02/2020	H16.9	1	Queratitis	1
2	28/02/2020	S05.9	9	Traumatismo	1
1	28/02/2020	T15.0	1	Cuerpo extraño	1

2	28/02/2020	H10.3	0	Conjuntivitis	1
1	28/02/2020	H15.1	6	Epiescleritis	1
1	28/02/2020	H15.00	6	Escleritis	1
1	28/02/2020	H10.3	0	Conjuntivitis	1
1	29/02/2020	T15.0	1	Cuerpo extraño	1
2	29/02/2020	H16.0	1	Úlcera corneal	1
1	29/02/2020	T15.0	1	Cuerpo extraño	1
1	29/02/2020	H20	6	Uveitis	1
1	29/02/2020	S05.9	9	Traumatismo	1
1	29/02/2020	H00.02	4	Orzuelo	1
2	29/02/2020	H10.3	0	Conjuntivitis	1
1	29/02/2020	H53	11	No patología	1
1	29/02/2020	H00.02	4	Orzuelo	1
1	29/02/2020	H35.3	8	Maculopatía	1
2	29/02/2020	H10.3	0	Conjuntivitis	1
1	29/02/2020	T15.0	1	Cuerpo extraño	1
1	29/02/2020	H16.9	1	Queratitis	1
2	29/02/2020	H00.02	4	Orzuelo	1
1	29/02/2020	H49.9	10	Parálisis oculomotora	1
2	29/02/2020	H10.3	0	Conjuntivitis	1
2	29/02/2020	H10.3	0	Conjuntivitis	1
1	29/02/2020	H53	11	No patología	1
1	29/02/2020	T15.0	1	Cuerpo extraño	1
2	29/02/2020	H16.9	1	Queratitis	1
2	29/02/2020	H16.9	1	Queratitis	1
2	29/02/2020	H02	4	Alteración palpebral	1
1	29/02/2020	T15.0	1	Cuerpo extraño	1
1	29/02/2020	H53	11	No patología	1
1	29/02/2020	H16.0	1	Úlcera corneal	1
2	29/02/2020	H01.00	4	Blefaritis	1
1	29/02/2020	H16.9	1	Queratitis	1
2	29/02/2020	H10.3	0	Conjuntivitis	1
1	29/02/2020	H01.00	4	Blefaritis	1
1	29/02/2020	H16.0	1	Úlcera corneal	1
2	29/02/2020	H10.81	0	Pingueculitis	1
2	29/02/2020	H16.9	1	Queratitis	1
2	29/02/2020	H10.3	0	Conjuntivitis	1
2	29/02/2020	H43.81	7	DVP	1
1	29/02/2020	H00.19	4	Chalazión	1
2	29/02/2020	H16.0	1	Úlcera corneal	1
2	29/02/2020	T15.0	1	Cuerpo extraño	1
1	29/02/2020	H11.3	0	Hiposfagma	1
2	29/02/2020	H00.02	4	Orzuelo	1
2	29/02/2020	H10.3	0	Conjuntivitis	1
2	29/02/2020	H16.9	1	Queratitis	1
1	29/02/2020	T15.0	1	Cuerpo extraño	1
1	29/02/2020	S05.9	9	Traumatismo	1
2	29/02/2020	H16.0	1	Úlcera corneal	1
1	29/02/2020	H20	6	Uveitis	1
2	29/02/2020	H16.0	1	Úlcera corneal	1
2	29/02/2020	H16.9	1	Queratitis	1
1	29/02/2020	T15.0	1	Cuerpo extraño	1
1	29/02/2020	H20	6	Uveitis	1
1	29/02/2020	H53	11	No patología	1
1	29/02/2020	H16.9	1	Queratitis	1
2	29/02/2020	S05.9	9	Traumatismo	1

Sexo	Fecha	CIE-10	Grupo	Diagnóstico	COVID: 1 NO 2 SI	
2	01/04/2020	H04.32	5	Dacriocistitis aguda	2	
1	01/04/2020	H04.32	5	Dacriocistitis aguda	2	
1	01/04/2020	E11.319	8	Retinopatía diabética	2	
1	01/04/2020	H16.9	1	Queratitis	2	
1	02/04/2020	H16.0	1	Abceso corneal	2	
1	02/04/2020	H10.3	0	Conjuntivitis	2	
1	02/04/2020	H16.0	1	Úlcera corneal	2	
2	02/04/2020	H35.0	8	Hemorragia retiniana	2	
1	03/04/2020	H20	6	Uveitis	2	
2	03/04/2020	H16.9	1	Queratitis	2	
1	03/04/2020	H11.3	0	Hiposfagma	2	
2	03/04/2020	H43.81	7	DVP	2	
2	03/04/2020	H10.3	0	Conjuntivitis	2	
2	04/04/2020	H16.9	1	Queratitis	2	
1	04/04/2020	H16.0	1	Úlcera corneal	2	
1	04/04/2020	H16.9	1	Queratitis	2	
2	04/04/2020	H11.3	0	Hiposfagma	2	
1	04/04/2020	H16.9	1	Queratitis	2	
2	05/04/2020	H10.3	0	Conjuntivitis	2	
1	05/04/2020	H00.02	4	Orzuelo	2	
2	05/04/2020	H18.20	1	Edema corneal	2	
2	05/04/2020	H16.0	1	Úlcera corneal	2	
1	06/04/2020	H11.82	0	Conjuntivocalasia	2	
1	06/04/2020	S05.9	9	Traumatismo	2	
2	06/04/2020	H16.9	1	Queratitis	2	
2	06/04/2020	B00.1	4	Dermatitis herpética	2	
2	06/04/2020	H16.9	1	Queratitis	2	
1	06/04/2020	H16.0	1	Úlcera corneal	2	
2	06/04/2020	H43.81	7	DVP	2	
1	07/04/2020	H16.9	1	Queratitis	2	
2	07/04/2020	H10.3	0	Conjuntivitis	2	
2	07/04/2020	H16.9	1	Queratitis	2	
2	07/04/2020	H43.81	7	DVP	2	
1	07/04/2020	H16.0	1	Úlcera corneal	2	
1	07/04/2020	H16.0	1	Úlcera corneal	2	
1	07/04/2020	S05.9	9	Traumatismo	2	
2	08/04/2020	B00.1	4	Dermatitis herpética	2	
1	08/04/2020	H43.81	7	DVP	2	
2	08/04/2020	B00.1	4	Dermatitis herpética	2	
1	08/04/2020	H46	10	Neuritis óptica	2	
2	08/04/2020	H16.9	1	Queratitis	2	
1	08/04/2020	H43.81	7	DVP	2	
2	08/04/2020	H10.3	0	Conjuntivitis	2	
1	09/04/2020	H40.9	3	Glaucoma	2	
1	09/04/2020	H16.9	1	Queratitis	2	
1	09/04/2020	H10.3	0	Blefaroconjuntivitis	2	
2	09/04/2020	H10.3	0	Conjuntivitis	2	
2	09/04/2020	H16.9	1	Queratitis	2	
1	09/04/2020	H49.9	10	Parálisis oculomotora	2	
2	09/04/2020	H40.9	3	Glaucoma	2	
2	10/04/2020	H10.3	0	Conjuntivitis	2	
2	10/04/2020	H0.41	1	Ojo seco	2	
2	10/04/2020	H11.3	0	Hiposfagma	2	
1	10/04/2020	H26.9	2	Catarata	2	
2	10/04/2020	H01.00	4	Blefaritis	2	
2	10/04/2020	H16.9	1	Queratitis	2	
2	10/04/2020	H53.9	11	Alteraciones visuales	2	
2	11/04/2020	H16.9	1	Queratitis	2	
1	11/04/2020	H10.3	0	Conjuntivitis	2	
2	11/04/2020	H20	6	Uveitis	2	
2	11/04/2020	H16.0	1	Úlcera corneal	2	

2	11/04/2020	T26.4	9	Quemadura	2	
2	11/04/2020	H10.3	0	Blefaroconjuntivitis	2	
2	11/04/2020	H00.02	4	Orzuelo	2	
1	11/04/2020	H16.9	1	Queratitis	2	
2	11/04/2020	H16.9	1	Queratitis	2	
2	11/04/2020	H10.3	0	Conjuntivitis	2	
1	12/04/2020	H16.9	1	Queratitis	2	
2	12/04/2020	H0.41	1	ojo seco	2	
2	12/04/2020	H10.3	0	Conjuntivitis	2	
2	12/04/2020	H10.3	0	Conjuntivitis	2	
2	12/04/2020	H20	6	Uveitis	2	
1	12/04/2020	H35.0	8	Hemorragia retiniana	2	
1	12/04/2020	E11.319	8	Retinopatía diabética	2	
2	12/04/2020	B58.0	8	Toxoplasmosis	2	
2	12/04/2020	H16.0	1	Úlcera corneal	2	
2	12/04/2020	H10.3	0	Conjuntivitis	2	
2	12/04/2020	H00.02	4	Orzuelo	2	
1	12/04/2020	H16.9	1	Queratitis	2	
1	12/04/2020	H16.0	1	Úlcera corneal	2	
1	13/04/2020	H10.3	0	Conjuntivitis	2	
2	13/04/2020	H10.3	0	Conjuntivitis	2	
2	13/04/2020	H43.1	7	Hemovítreo	2	
2	13/04/2020	H16.0	1	Úlcera corneal	2	
2	13/04/2020	H16.9	1	Queratitis	2	
2	14/04/2020	H00.02	4	Orzuelo	2	
1	14/04/2020	H16.0	1	Úlcera corneal	2	
1	15/04/2020	T15.0	1	Cuerpo extraño	2	
2	15/04/2020	H16.9	1	Queratitis	2	
2	15/04/2020	H43.81	7	DVP	2	
2	15/04/2020	H17.8	1	Infiltrados corneales	2	
2	15/04/2020	H00.02	4	Orzuelo	2	
2	15/04/2020	H10.3	0	Conjuntivitis	2	
1	15/04/2020	H43.1	7	Hemovítreo	2	
2	15/04/2020	H16.9	1	Queratitis	2	
1	15/04/2020	T15.0	1	Cuerpo extraño	2	
2	15/04/2020	H16.0	1	Úlcera corneal	2	
1	15/04/2020	S05.9	9	Traumatismo	2	
2	16/04/2020	H16.9	1	Queratitis	2	
2	16/04/2020	H01.00	4	Blefaritis	2	
1	16/04/2020	G43.9	10	Migraña	2	
1	16/04/2020	H02	4	Alteración palpebral	2	
2	16/04/2020	H44.009	8	Endoftalmitis	2	
2	16/04/2020	H43.81	7	DVP	2	
1	16/04/2020	H16.9	1	Queratitis	2	
2	17/04/2020	H43.81	7	DVP	2	
2	17/04/2020	H10.3	0	Conjuntivitis	2	
1	17/04/2020	H16.0	1	Úlcera corneal	2	
2	17/04/2020	Alta por fuga	11	Alta por fuga	2	
1	17/04/2020	H16.0	1	Úlcera corneal	2	
1	17/04/2020	H16.0	1	Úlcera corneal	2	
1	17/04/2020	H20	6	Uveitis	2	
1	17/04/2020	H10.3	0	Conjuntivitis	2	
1	17/04/2020	H20	6	Uveitis	2	
2	18/04/2020	G43.9	10	Migraña	2	
2	18/04/2020	H10.3	0	Conjuntivitis	2	
1	18/04/2020	H00.02	4	Orzuelo	2	
2	18/04/2020	H16.0	1	Abceso corneal	2	
2	18/04/2020	H44.009	8	Endoftalmitis	2	
1	18/04/2020	H10.3	0	Conjuntivitis	2	
2	18/04/2020	H16.9	1	Queratitis	2	
2	18/04/2020	H43.1	7	Hemovítreo	2	
1	18/04/2020	H16.0	1	Úlcera corneal	2	

2	18/04/2020	H43.81	7	DVP	2	
2	18/04/2020	H00.02	4	Orzuelo	2	
1	19/04/2020	H16.0	1	Úlcera corneal	2	
2	19/04/2020	H16.0	1	Úlcera corneal	2	
1	19/04/2020	H20	6	Uveitis	2	
2	19/04/2020	H44.009	8	Endoftalmitis	2	
2	19/04/2020	H43.81	7	DVP	2	
2	19/04/2020	H33.30	8	Desgarro retiniano	2	
2	19/04/2020	H16.9	1	Queratitis	2	
2	20/04/2020	H15.00	6	Escleritis	2	
2	20/04/2020	H00.02	4	Orzuelo	2	
2	20/04/2020	H16.9	1	Queratitis	2	
1	20/04/2020	H26.9	2	Post op catarata	2	
1	20/04/2020	H00.02	4	Orzuelo	2	
1	20/04/2020	H43.81	7	DVP	2	
2	20/04/2020	H43.81	7	DVP	2	
2	20/04/2020	H44.009	8	Endoftalmitis	2	
2	20/04/2020	H10.3	0	Conjuntivitis	2	
1	20/04/2020	H16.9	1	Queratitis	2	
1	21/04/2020	H16.9	1	Queratitis	2	
2	21/04/2020	H02	4	Lesión palpebral	2	
1	21/04/2020	H33.30	8	Desgarro retiniano	2	
2	21/04/2020	S05.00	9	Abrasión conjuntival	2	
2	21/04/2020	H16.0	1	Abceso corneal	2	
2	21/04/2020	H31.42	8	Coroidopatía serosa central	2	
1	21/04/2020	H33.0	8	Desprendimiento de retina	2	
2	21/04/2020	H43.81	7	DVP	2	
2	21/04/2020	H11.3	0	Hiposfagma	2	
2	21/04/2020	H01.00	4	Blefaritis	2	
2	21/04/2020	H00.02	4	Orzuelo	2	
2	21/04/2020	H10.3	0	Conjuntivitis	2	
1	21/04/2020	H53	11	No patología	2	
1	21/04/2020	H16.0	3	Atalamia	2	
1	21/04/2020	H02.05	4	Distiquiasis	2	
2	21/04/2020	B00.1	4	Dermatitis herpética	2	
2	21/04/2020	H43.81	7	DVP	2	
2	21/04/2020	H16.9	1	Queratitis	2	
1	21/04/2020	H16.9	1	Queratitis	2	
2	21/04/2020	H16.9	1	Queratitis	2	
2	21/04/2020	H16.0	1	Úlcera corneal	2	
2	22/04/2020	H20	6	Uveitis	2	
2	22/04/2020	H43.81	7	DVP	2	
2	22/04/2020	H20	6	Uveitis	2	
2	22/04/2020	H15.00	6	Escleritis	2	
1	22/04/2020	H11.3	0	Hiposfagma	2	
1	22/04/2020	H16.9	1	Queratitis	2	
1	22/04/2020	T15.0	1	Cuerpo extraño	2	
2	23/04/2020	H16.0	1	Abceso corneal	2	
2	23/04/2020	H26.9	2	Catarata	2	
1	23/04/2020	H10.3	0	Conjuntivitis	2	
1	23/04/2020	H43.81	7	DVP	2	
1	23/04/2020	H16.9	1	Queratitis	2	
2	23/04/2020	G43.109	10	Aura	2	
1	23/04/2020	H33.0	8	Desprendimiento de retina	2	
2	23/04/2020	T26	9	Causticación	2	
2	23/04/2020	H53	11	No patología	2	
2	23/04/2020	H16.9	1	Queratitis	2	
1	23/04/2020	H20	6	Uveitis	2	
2	23/04/2020	H10.3	0	Conjuntivitis	2	
1	23/04/2020	H11.3	0	Hiposfagma	2	
1	23/04/2020	T15.0	1	Cuerpo extraño	2	
1	23/04/2020	H16.9	1	Queratitis	2	

1	23/04/2020	T15.0	1	Cuerpo extraño	2	
2	23/04/2020	H00.02	4	Orzuelo	2	
1	24/04/2020	H10.3	0	Conjuntivitis	2	
1	24/04/2020	H16.0	1	Úlcera corneal	2	
1	24/04/2020	H33.0	8	Desprendimiento de retina	2	
2	24/04/2020	H16.0	1	Úlcera corneal	2	
1	24/04/2020	H02	4	Lesión palpebral	2	
1	24/04/2020	H53	11	No patología	2	
2	24/04/2020	H43.81	7	DVP	2	
2	24/04/2020	H16.0	1	Úlcera corneal	2	
1	24/04/2020	H10.81	0	Pingueculitis	2	
2	24/04/2020	H11.3	0	Hiposfagma	2	
1	24/04/2020	H34.82	8	Trombosis venosa	2	
1	24/04/2020	H11.44	0	Quiste conjuntival	2	
1	24/04/2020	H43.81	7	DVP	2	
2	24/04/2020	G43.9	10	Migraña	2	
2	24/04/2020	H20	6	Uveitis	2	
1	24/04/2020	H10.3	0	Conjuntivitis	2	
1	24/04/2020	H18.20	1	Edema corneal	2	
1	24/04/2020	H16.9	1	Queratitis	2	
2	25/04/2020	H43.81	7	DVP	2	
1	25/04/2020	T15.0	1	Cuerpo extraño	2	
1	25/04/2020	T15.0	1	Cuerpo extraño	2	
1	25/04/2020	H18.50	1	Distrofia corneal	2	
1	25/04/2020	H16.9	1	Queratitis	2	
2	25/04/2020	H00.19	4	Chalazión	2	
1	25/04/2020	H16.0	1	Úlcera corneal	2	
2	25/04/2020	H16.9	1	Queratitis	2	
2	25/04/2020	H43.81	7	DVP	2	
1	25/04/2020	T15.0	1	Cuerpo extraño	2	
1	25/04/2020	T15.0	1	Cuerpo extraño	2	
1	25/04/2020	H35.3	8	Maculopatía	2	
1	26/04/2020	S05.9	9	Traumatismo	2	
2	26/04/2020	H10.3	0	Blefaroconjuntivitis	2	
2	26/04/2020	H47.10	10	Papiledema	2	
2	26/04/2020	H10.3	0	Conjuntivitis	2	
1	26/04/2020	H40.9	3	Glaucoma secundario	2	
1	26/04/2020	H16.0	1	Úlcera corneal	2	
2	26/04/2020	S05.9	9	Traumatismo	2	
1	26/04/2020	S05.9	9	Traumatismo	2	
2	26/04/2020	E11.319	8	Retinopatía diabética	2	
2	26/04/2020	H16.9	1	Queratitis	2	
2	26/04/2020	H16.9	1	Queratitis	2	
1	26/04/2020	H16.0	1	Úlcera corneal	2	
1	26/04/2020	H11.00	0	Pterigium	2	
1	26/04/2020	H16.0	1	Úlcera corneal	2	
1	27/04/2020	H16.0	1	Úlcera corneal	2	
1	27/04/2020	H33.0	8	Desprendimiento de retina	2	
2	27/04/2020	H11.3	0	Hiposfagma	2	
2	27/04/2020	H43.81	7	DVP	2	
2	27/04/2020	H16.0	1	Úlcera corneal	2	
2	27/04/2020	T15.0	1	Cuerpo extraño	2	
2	27/04/2020	H50.9	10	Estrabismo	2	
2	27/04/2020	H16.9	1	Queratitis	2	
2	27/04/2020	H11.3	0	Hiposfagma	2	
2	27/04/2020	T26	9	Causticación	2	
2	27/04/2020	H10.3	0	Conjuntivitis	2	
2	27/04/2020	H10.3	0	Conjuntivitis	2	
2	27/04/2020	H16.9	1	Queratitis	2	
1	27/04/2020	H10.3	0	Conjuntivitis	2	
1	27/04/2020	H53	11	No patología	2	
1	27/04/2020	H43.81	7	DVP	2	

2	27/04/2020	H10.3	0	Conjuntivitis	2	
2	27/04/2020	H43.81	7	DVP	2	
2	27/04/2020	H20	6	Uveitis	2	
2	27/04/2020	H20	6	Uveitis	2	
1	27/04/2020	H00.02	4	Orzuelo	2	
1	27/04/2020	T15.0	1	Cuerpo extraño	2	
2	27/04/2020	H01.00	4	Blefaritis	2	
2	27/04/2020	H27.8	2	OCP	2	
2	27/04/2020	H16.9	1	Queratitis	2	
2	28/04/2020	H00.02	4	Orzuelo	2	
1	28/04/2020	H16.9	1	Queratitis	2	
1	28/04/2020	H40.05	3	HTO	2	
1	28/04/2020	H43.81	7	DVP	2	
2	28/04/2020	H27.8	2	OCP	2	
1	28/04/2020	H43.1	7	Hemovítreo	2	
2	28/04/2020	H33.30	8	Desgarro retiniano	2	
1	28/04/2020	H20	6	Uveitis	2	
2	28/04/2020	H16.9	1	Queratitis	2	
2	28/04/2020	H10.3	0	Conjuntivitis	2	
2	28/04/2020	H43.81	7	DVP	2	
2	28/04/2020	H43.81	7	DVP	2	
1	29/04/2020	H43.1	7	Hemovítreo	2	
2	29/04/2020	H01.00	4	Blefaritis	2	
1	29/04/2020	H10.3	0	Conjuntivitis	2	
2	29/04/2020	H20	6	Uveitis	2	
2	29/04/2020	H43.81	7	DVP	2	
2	29/04/2020	H10.3	0	Conjuntivitis	2	
2	29/04/2020	H43.81	7	DVP	2	
1	29/04/2020	H10.3	0	Conjuntivitis	2	
1	29/04/2020	T15.0	1	Cuerpo extraño	2	
2	29/04/2020	H01.00	4	Blefaritis	2	
2	29/04/2020	H00.02	4	Orzuelo	2	
2	29/04/2020	H20	6	Uveitis	2	
1	29/04/2020	B58.0	8	Toxoplasmosis	2	
2	29/04/2020	H11.3	0	Hiposfagma	2	
1	29/04/2020	H43.81	7	DVP	2	
2	29/04/2020	H00.02	4	Orzuelo	2	
1	29/04/2020	T26	9	Causticación	2	
2	30/04/2020	H43.81	7	DVP	2	
2	30/04/2020	H11.3	0	Hiposfagma	2	
2	30/04/2020	H20	6	Uveitis	2	
2	30/04/2020	H35.30	8	DMAE	2	
1	30/04/2020	H02	4	Lesión palpebral	2	
1	30/04/2020	H16.9	1	Queratitis	2	
1	30/04/2020	H20	6	Uveitis	2	
2	30/04/2020	H11.3	0	Hiposfagma	2	
2	30/04/2020	B00.1	4	Dermatitis herpética	2	
2	30/04/2020	S05.6	9	Perforación ocular	2	
1	30/04/2020	S05.30	9	Laceración conjuntival	2	
1	30/04/2020	H16.0	1	Úlcera corneal	2	
1	30/04/2020	H16.0	1	Úlcera corneal	2	
1	30/04/2020	H16.9	1	Queratitis	2	
1	30/04/2020	H17.8	1	Infiltrados corneales	2	
2	30/04/2020	H16.9	1	Queratitis	2	
2	30/04/2020	H10.3	0	Conjuntivitis	2	
2	01/04/2019 0:25	H10.3	0	Conjuntivitis	1	
1	01/04/2019 4:20	H10.3	0	Conjuntivitis	1	
2	01/04/2019 9:42	S05.9	9	Traumatismo	1	
1	01/04/2019 10:07	H35.30	8	DMAE	1	
1	01/04/2019 10:09	H33.0	8	Desprendimiento de retina	1	
1	01/04/2019 10:11	H35.30	8	DMAE	1	
1	01/04/2019 10:23	H00.02	4	Orzuelo	1	

1	01/04/2019 10:25	H50.9	10	Estrabismo	1	
2	01/04/2019 10:44	H10.3	0	Conjuntivitis	1	
2	01/04/2019 10:45	H10.3	0	Conjuntivitis	1	
2	01/04/2019 10:53	H16.9	1	Queratitis	1	
2	01/04/2019 11:09	H10.3	11	Conjuntivitis	1	
2	01/04/2019 11:19	H20	6	Uveitis	1	
1	01/04/2019 11:19	H26.9	2	Catarata	1	
2	01/04/2019 11:23	H10.3	0	Conjuntivitis	1	
1	01/04/2019 11:29	H16.0	1	Úlcera corneal	1	
2	01/04/2019 11:29	H11.3	0	Hiposfagma	1	
2	01/04/2019 11:33	G43.109	10	Aura	1	
1	01/04/2019 11:38	H53	11	No patología	1	
2	01/04/2019 11:41	H27.8	2	OCP	1	
2	01/04/2019 11:50	H00.02	1	Orzuelo	1	
2	01/04/2019 11:50	H53.9	11	Alteraciones visuales	1	
2	01/04/2019 12:02	H16.0	1	Úlcera corneal	1	
2	01/04/2019 12:24	H10.3	0	Conjuntivitis	1	
2	01/04/2019 12:24	H43.81	7	DVP	1	
2	01/04/2019 12:28	H18.20	1	Edema corneal	1	
2	01/04/2019 12:31	H10.3	0	Conjuntivitis	1	
2	01/04/2019 12:36	H35.3	8	Maculopatía	1	
1	01/04/2019 13:23	H16.9	1	Queratitis	1	
1	01/04/2019 13:37	H10.3	0	Conjuntivitis	1	
1	01/04/2019 13:48	H00.02	4	Orzuelo	1	
1	01/04/2019 14:18	S05.9	9	Traumatismo	1	
2	01/04/2019 14:26	H11.3	0	Hiposfagma	1	
1	01/04/2019 14:31	H16.0	1	Úlcera corneal	1	
1	01/04/2019 16:09	H16.9	1	Queratitis	1	
2	01/04/2019 16:16	H26.9	2	Post op catarata	1	
2	01/04/2019 16:50	H16.9	1	Queratitis	1	
2	01/04/2019 16:54	H43.81	7	DVP	1	
1	01/04/2019 16:56	H16.9	1	Queratitis	1	
2	01/04/2019 17:05	H16.9	1	Queratitis	1	
1	01/04/2019 17:42	H01.00	4	Blefaritis	1	
2	01/04/2019 18:03	H11.00	0	Pterigium	1	
1	01/04/2019 18:13	H11.3	0	Hiposfagma	1	
1	01/04/2019 18:25	H16.0	1	Úlcera corneal	1	
2	01/04/2019 18:46	H26.9	2	Catarata	1	
1	01/04/2019 18:53	H11.3	0	Hiposfagma	1	
1	01/04/2019 19:42	H10.3	0	Conjuntivitis	1	
2	01/04/2019 19:43	H10.3	0	conjuntivitis	1	
2	01/04/2019 19:54	S05.9	9	Traumatismo	1	
2	01/04/2019 20:25	H10.3	0	conjuntivitis	1	
2	01/04/2019 20:28	H16.0	1	Úlcera corneal	1	
1	01/04/2019 20:42	H16.0	1	Úlcera corneal	1	
2	01/04/2019 21:01	H10.3	0	conjuntivitis	1	
1	01/04/2019 21:11	H00.02	4	Orzuelo	1	
1	01/04/2019 21:18	S05.9	9	Traumatismo	1	
2	01/04/2019 21:33	H00.02	4	Orzuelo	1	
2	01/04/2019 21:35	H10.3	0	Conjuntivitis	1	
2	01/04/2019 22:48	H43.81	7	DVP	1	
1	01/04/2019 22:57	H26.9	2	Catarata	1	
2	01/04/2019 23:02	H10.3	0	Conjuntivitis	1	
2	02/04/2019 8:51	H16.9	1	Queratitis	1	
1	02/04/2019 9:26	H16.9	1	Queratitis	1	
2	02/04/2019 9:38	H16.9	1	Queratitis	1	
2	02/04/2019 10:03	H02.05	4	Distiquiasis	1	
1	02/04/2019 10:04	H10.3	1	Conjuntivitis	1	
2	02/04/2019 10:21	H00.19	4	Chalazión	1	
2	02/04/2019 10:27	H11.00	0	Pterigium	1	
2	02/04/2019 10:27	H16.9	1	Queratitis	1	
1	02/04/2019 10:32	H10.3	0	Conjuntivitis	1	

1	02/04/2019 10:39	H16.9	1	Queratitis	1	
1	02/04/2019 10:40	H15.00	6	Epiescleritis	1	
2	02/04/2019 10:40	H16.9	1	Queratitis	1	
1	02/04/2019 10:53	H10.3	0	Conjuntivitis	1	
2	02/04/2019 10:59	H04.32	5	Dacriocistitis aguda	1	
2	02/04/2019 11:26	H27.8	2	OCP	1	
2	02/04/2019 11:27	H16.0	1	Úlcera corneal	1	
2	02/04/2019 11:53	H16.0	1	Úlcera corneal	1	
1	02/04/2019 11:54	H20	6	Uveitis	1	
2	02/04/2019 11:55	H16.9	1	Queratitis	1	
2	02/04/2019 12:01	H10.3	0	Conjuntivitis	1	
2	02/04/2019 12:28	H17.8	1	Infiltrados corneales	1	
2	02/04/2019 12:57	H47.32	10	Drusas de nervio óptico	1	
2	02/04/2019 13:16	H16.9	1	Queratitis	1	
2	02/04/2019 13:18	H53	11	No patología	1	
2	02/04/2019 13:19	H11.00	0	Pterigium	1	
1	02/04/2019 13:55	H11.3	0	Hiposfagma	1	
1	02/04/2019 14:02	H16.9	1	Queratitis	1	
2	02/04/2019 14:10	H53.9	10	Alteraciones visuales	1	
2	02/04/2019 14:32	H43.81	7	DVP	1	
1	02/04/2019 14:37	H16.0	1	Úlcera corneal	1	
2	02/04/2019 14:56	H16.9	1	Queratitis	1	
2	02/04/2019 14:57	H10.3	0	Conjuntivitis	1	
2	02/04/2019 15:00	H16.9	1	Queratitis	1	
2	02/04/2019 15:04	H16.9	1	Queratitis	1	
1	02/04/2019 16:22	H10.3	0	Conjuntivitis	1	
2	02/04/2019 16:30	H16.9	1	Queratitis	1	
2	02/04/2019 16:39	H33.0	8	Desprendimiento de retina	1	
2	02/04/2019 16:45	H16.9	1	Queratitis	1	
1	02/04/2019 16:46	H16.9	1	Queratitis	1	
2	02/04/2019 17:01	H15.00	6	Epiescleritis	1	
1	02/04/2019 17:14	H16.9	1	Queratitis	1	
2	02/04/2019 17:30	H10.3	0	Conjuntivitis	1	
1	02/04/2019 17:30	H11.00	0	Pterigium	1	
2	02/04/2019 18:07	H15.00	6	Escleritis	1	
2	02/04/2019 18:27	G43.9	10	Migraña	1	
2	02/04/2019 19:27	H16.0	1	Úlcera corneal	1	
1	02/04/2019 19:31	H00.19	4	Chalazión	1	
2	02/04/2019 20:19	H53	11	No patología	1	
2	02/04/2019 20:22	H16.9	1	Queratitis	1	
2	02/04/2019 20:25	H17.8	1	Infiltrados corneales	1	
1	02/04/2019 20:37	H11.3	0	Hiposfagma	1	
2	02/04/2019 21:24	H10.3	0	Conjuntivitis	1	
2	02/04/2019 21:27	T15.0	1	Cuerpo extraño	1	
1	02/04/2019 23:32	H53	1	No patología	1	
1	03/04/2019 2:52	H34.9	8	Oclusión arterial	1	
1	03/04/2019 6:58	H02	4	Alteración palpebral	1	
1	03/04/2019 7:10	H16.9	1	Queratitis	1	
2	03/04/2019 8:20	H04.32	5	Dacriocistitis aguda	1	
1	03/04/2019 8:34	H16.9	1	Queratitis	1	
1	03/04/2019 9:07	H33.0	8	Desprendimiento de retina	1	
2	03/04/2019 9:16	H11.3	1	Hiposfagma	1	
2	03/04/2019 9:41	H16.9	1	Queratitis	1	
2	03/04/2019 9:44	H00.02	4	Orzuelo	1	
2	03/04/2019 10:04	H16.9	1	Queratitis	1	
2	03/04/2019 10:09	H43.81	7	DVP	1	
2	03/04/2019 10:22	H16.9	1	Queratitis	1	
1	03/04/2019 10:26	H46	10	Neuritis óptica	1	
1	03/04/2019 10:38	H53.9	11	Alteraciones visuales	1	
1	03/04/2019 10:48	H10.3	0	Conjuntivitis	1	
2	03/04/2019 11:10	H10.3	0	Conjuntivitis	1	
1	03/04/2019 12:04	H43.1	7	Hemovítreo	1	

1	03/04/2019 13:06	H43.81	7	DVP	1	
2	03/04/2019 13:24	G43.9	10	Migraña	1	
1	03/04/2019 13:28	H02	4	Alteración palpebral	1	
2	03/04/2019 14:16	H16.9	1	Queratitis	1	
2	03/04/2019 14:23	H10.3	0	Conjuntivitis	1	
1	03/04/2019 14:28	T15.0	1	Cuerpo extraño	1	
2	03/04/2019 14:51	H16.9	1	Queratitis	1	
1	03/04/2019 15:07	T15.0	1	Cuerpo extraño	1	
2	03/04/2019 16:10	H01.00	4	Blefaritis	1	
2	03/04/2019 16:11	H10.3	0	Conjuntivitis	1	
2	03/04/2019 16:35	H01.00	4	Blefaritis	1	
2	03/04/2019 16:54	H10.3	0	Conjuntivitis	1	
2	03/04/2019 17:03	H20	6	Uveitis	1	
2	03/04/2019 17:06	H35.30	8	DMAE	1	
2	03/04/2019 17:08	H16.9	1	Queratitis	1	
2	03/04/2019 17:37	H35.30	8	DMAE	1	
2	03/04/2019 17:37	H35.3	8	Maculopatía	1	
1	03/04/2019 17:59	H02	4	Alteración palpebral	1	
1	03/04/2019 18:17	H16.9	1	Queratitis	1	
2	03/04/2019 18:38	H10.81	0	Pingueculitis	1	
1	03/04/2019 18:44	H16.9	1	Queratitis	1	
2	03/04/2019 18:49	H20	6	Uveitis	1	
1	03/04/2019 18:51	H16.9	1	Queratitis	1	
2	03/04/2019 19:17	H01.00	4	Blefaritis	1	
2	03/04/2019 19:36	H16.9	1	Queratitis	1	
1	03/04/2019 20:05	H43.81	7	DVP	1	
1	03/04/2019 20:46	H10.3	0	Conjuntivitis	1	
1	03/04/2019 20:53	H16.0	1	Úlcera corneal	1	
1	03/04/2019 20:59	H16.9	1	Queratitis	1	
1	03/04/2019 21:09	H15.00	6	Epiescleritis	1	
2	03/04/2019 21:11	H02	4	Alteración palpebral	1	
2	03/04/2019 21:14	H10.3	0	Conjuntivitis	1	
1	03/04/2019 21:24	H10.3	0	Conjuntivitis	1	
1	03/04/2019 22:09	H10.3	0	Conjuntivitis	1	
2	03/04/2019 22:40	H16.9	1	Queratitis	1	
1	03/04/2019 22:53	H16.0	1	Úlcera corneal	1	
1	04/04/2019 0:16	T15.0	1	Cuerpo extraño	1	
2	04/04/2019 1:14	H16.9	1	Queratitis	1	
2	04/04/2019 2:20	H02	4	Alteración palpebral	1	
1	04/04/2019 8:06	H17.8	1	Infiltrados corneales	1	
2	04/04/2019 8:45	H16.9	1	Queratitis	1	
2	04/04/2019 9:21	H10.3	0	Conjuntivitis	1	
1	04/04/2019 9:22	H43.81	7	DVP	1	
2	04/04/2019 9:26	H10.3	0	Conjuntivitis	1	
2	04/04/2019 9:43	H16.9	1	Queratitis	1	
1	04/04/2019 9:59	H00.02	4	Orzuelo	1	
1	04/04/2019 10:22	H10.3	0	Conjuntivitis	1	
2	04/04/2019 10:40	H59.3	4	Post op párpados	1	
2	04/04/2019 10:41	H10.3	0	Conjuntivitis	1	
2	04/04/2019 10:42	S05.9	9	Traumatismo	1	
2	04/04/2019 11:20	H16.0	1	Úlcera corneal	1	
2	04/04/2019 11:21	H15.00	6	Epiescleritis	1	
2	04/04/2019 11:26	H17.8	1	Infiltrados corneales	1	
2	04/04/2019 11:28	H16.0	1	Úlcera corneal	1	
2	04/04/2019 11:29	H16.9	1	Queratitis	1	
2	04/04/2019 11:38	H17.8	1	Infiltrados corneales	1	
1	04/04/2019 11:46	H10.3	0	Conjuntivitis	1	
2	04/04/2019 11:54	H16.9	1	Queratitis	1	
1	04/04/2019 12:06	H10.3	0	Conjuntivitis	1	
2	04/04/2019 12:20	H16.9	1	Queratitis	1	
2	04/04/2019 12:23	H15.00	6	Epiescleritis	1	
2	04/04/2019 12:33	G43.9	10	Migraña	1	

1	04/04/2019 12:35	H16.0	1	Úlcera corneal	1	
1	04/04/2019 12:42	H02	4	Alteración palpebral	1	
2	04/04/2019 13:28	H27.8	2	OCP	1	
2	04/04/2019 13:51	H43.81	7	DVP	1	
1	04/04/2019 14:08	H04.32	5	Dacriocistitis aguda	1	
1	04/04/2019 15:40	H16.9	1	Queratitis	1	
2	04/04/2019 16:05	H26.9	2	Post op catarata	1	
1	04/04/2019 16:12	H20	6	Uveitis	1	
2	04/04/2019 16:18	H26.9	2	Catarata	1	
1	04/04/2019 16:32	H11.3	0	Hiposfagma	1	
2	04/04/2019 16:43	H43.81	7	DVP	1	
2	04/04/2019 16:46	H02	4	Alteración palpebral	1	
2	04/04/2019 16:47	H10.3	0	Conjuntivitis	1	
1	04/04/2019 16:54	S05.00	1	Abrasión conjuntival	1	
1	04/04/2019 16:55	T15.0	1	Cuerpo extraño	1	
2	04/04/2019 17:23	H10.3	0	Conjuntivitis	1	
2	04/04/2019 17:27	H00.02	4	Orzuelo	1	
2	04/04/2019 17:30	H00.02	4	Orzuelo	1	
1	04/04/2019 17:32	T15.0	1	Cuerpo extraño	1	
2	04/04/2019 17:38	H10.3	0	Conjuntivitis	1	
2	04/04/2019 17:42	H11.3	0	Hiposfagma	1	
2	04/04/2019 17:43	H11.3	0	Hiposfagma	1	
1	04/04/2019 18:00	H20	6	Uveitis	1	
1	04/04/2019 18:35	S05.9	9	Traumatismo	1	
1	04/04/2019 18:45	H16.9	1	Queratitis	1	
2	04/04/2019 18:45	H16.0	1	Úlcera corneal	1	
1	04/04/2019 18:55	H10.3	0	Conjuntivitis	1	
2	04/04/2019 18:57	H16.9	1	Queratitis	1	
1	04/04/2019 18:58	H11.3	0	Hiposfagma	1	
1	04/04/2019 19:05	S05.9	9	Traumatismo	1	
2	04/04/2019 19:17	H16.9	1	Queratitis	1	
2	04/04/2019 19:20	H10.3	0	Conjuntivitis	1	
1	04/04/2019 19:47	H34.9	8	Oclusión arterial	1	
1	04/04/2019 19:51	H11.3	0	Hiposfagma	1	
1	04/04/2019 19:52	H10.3	0	Conjuntivitis	1	
2	04/04/2019 20:16	H53	11	No patología	1	
2	04/04/2019 20:37	H10.3	0	Conjuntivitis	1	
2	04/04/2019 20:42	H16.9	1	Queratitis	1	
2	04/04/2019 21:09	H10.3	0	Conjuntivitis	1	
1	05/04/2019 1:14	H16.9	1	Queratitis	1	
1	05/04/2019 3:57	H16.0	1	Úlcera corneal	1	
1	05/04/2019 9:01	H43.81	7	DVP	1	
1	05/04/2019 9:32	H43.81	7	DVP	1	
2	05/04/2019 9:36	H02	4	Alteración palpebral	1	
1	05/04/2019 10:17	H53	11	No patología	1	
1	05/04/2019 10:39	H16.9	1	Queratitis	1	
2	05/04/2019 10:49	H16.9	1	Queratitis	1	
1	05/04/2019 10:50	H16.9	1	Queratitis	1	
2	05/04/2019 11:05	H10.3	0	Conjuntivitis	1	
2	05/04/2019 11:29	H10.3	0	Conjuntivitis	1	
2	05/04/2019 11:31	H11.3	0	Hiposfagma	1	
1	05/04/2019 11:54	H53	11	No patología	1	
1	05/04/2019 12:05	H01.00	4	Blefaritis	1	
2	05/04/2019 12:29	H53	11	No patología	1	
1	05/04/2019 12:50	T15.0	1	Cuerpo extraño	1	
1	05/04/2019 13:15	H11.00	0	Pterigium	1	
2	05/04/2019 14:48	H10.3	0	Conjuntivitis	1	
2	05/04/2019 14:54	G43.109	10	Aura	1	
2	05/04/2019 15:06	H16.9	1	Queratitis	1	
1	05/04/2019 15:36	H16.9	1	Queratitis	1	
2	05/04/2019 15:59	H16.0	1	Úlcera corneal	1	
1	05/04/2019 16:00	H0.41	1	Ojo seco	1	

2	05/04/2019 16:18	S05.30	1	Laceración conjuntival	1	
2	05/04/2019 16:54	H01.00	4	Blefaritis	1	
1	05/04/2019 17:19	H40.9	3	Glaucoma secundario	1	
2	05/04/2019 17:29	H11.3	1	Hiposfagma	1	
2	05/04/2019 17:35	H20	6	Uveitis	1	
1	05/04/2019 17:51	T15.0	1	Cuerpo extraño	1	
1	05/04/2019 18:07	S05.9	9	Traumatismo	1	
2	05/04/2019 18:17	S05.9	9	Traumatismo	1	
1	05/04/2019 18:28	S05.9	9	Traumatismo	1	
2	05/04/2019 18:52	H01.00	4	Blefaritis	1	
2	05/04/2019 18:53	H05.82	4	Trocleítis	1	
1	05/04/2019 19:03	T15.0	1	Cuerpo extraño	1	
2	05/04/2019 19:33	H10.81	0	Pingueculitis	1	
1	05/04/2019 19:35	B00.1	4	Dermatitis herpética	1	
1	05/04/2019 19:42	H15.00	6	Epiescleritis	1	
2	05/04/2019 19:43	H35.30	8	DMAE	1	
2	05/04/2019 20:01	H10.3	0	Conjuntivitis	1	
1	05/04/2019 20:09	H43.81	7	DVP	1	
2	05/04/2019 20:23	H43.81	7	DVP	1	
1	05/04/2019 20:31	H43.81	7	DVP	1	
1	05/04/2019 20:36	H16.9	1	Queratitis	1	
1	05/04/2019 21:10	H16.9	1	Queratitis	1	
2	05/04/2019 21:30	H15.00	6	Epiescleritis	1	
1	05/04/2019 22:46	H43.81	7	DVP	1	
1	05/04/2019 23:24	H16.0	1	Úlcera corneal	1	
2	05/04/2019 23:37	H20	6	Uveitis	1	
1	06/04/2019 1:21	H10.3	0	Conjuntivitis	1	
1	06/04/2019 1:23	T15.0	1	Cuerpo extraño	1	
1	06/04/2019 6:07	S05.9	9	Traumatismo	1	
2	06/04/2019 8:13	H20	6	Uveitis	1	
2	06/04/2019 9:01	H43.81	7	DVP	1	
1	06/04/2019 9:04	H02	4	Lesión palpebral	1	
2	06/04/2019 9:23	H10.3	0	Conjuntivitis	1	
1	06/04/2019 9:34	H00.19	4	Chalazión	1	
2	06/04/2019 11:01	H16.9	1	Queratitis	1	
1	06/04/2019 11:20	H10.3	0	Conjuntivitis	1	
1	06/04/2019 11:23	H10.3	0	Conjuntivitis	1	
1	06/04/2019 11:28	H11.3	1	Hiposfagma	1	
2	06/04/2019 11:44	H16.9	1	Queratitis	1	
2	06/04/2019 12:04	H16.9	1	Queratitis	1	
2	06/04/2019 12:28	H16.0	1	Úlcera corneal	1	
2	06/04/2019 12:53	H01.00	4	Blefaritis	1	
2	06/04/2019 13:29	H10.3	0	Conjuntivitis	1	
2	06/04/2019 13:36	H16.9	1	Queratitis	1	
2	06/04/2019 14:19	H43.81	7	DVP	1	
2	06/04/2019 14:49	H10.3	0	Conjuntivitis	1	
2	06/04/2019 14:51	H01.00	4	Blefaritis	1	
2	06/04/2019 15:03	H10.3	0	Conjuntivitis	1	
2	06/04/2019 16:02	H43.81	7	DVP	1	
1	06/04/2019 16:15	H10.3	0	Conjuntivitis	1	
1	06/04/2019 16:16	H10.3	0	Conjuntivitis	1	
1	06/04/2019 16:57	T15.0	1	Cuerpo extraño	1	
2	06/04/2019 17:34	H16.9	1	Queratitis	1	
1	06/04/2019 17:58	B00.1	4	Dermatitis herpética	1	
2	06/04/2019 18:07	H10.3	0	Conjuntivitis	1	
2	06/04/2019 18:15	H10.3	0	Conjuntivitis	1	
2	06/04/2019 18:26	H00.19	4	Chalazión	1	
1	06/04/2019 18:52	T15.0	0	Cuerpo extraño	1	
2	06/04/2019 19:08	T15.0	1	Cuerpo extraño	1	
2	06/04/2019 19:31	H10.3	0	Conjuntivitis	1	
2	06/04/2019 19:38	H10.3	0	Conjuntivitis	1	
2	06/04/2019 19:39	H10.3	0	Conjuntivitis	1	

1	06/04/2019 19:46	H10.3	0	Conjuntivitis	1	
2	06/04/2019 19:52	H10.3	0	Conjuntivitis	1	
1	06/04/2019 20:15	H10.3	0	Conjuntivitis	1	
2	06/04/2019 20:17	H10.3	0	Conjuntivitis	1	
1	06/04/2019 20:19	H10.3	0	Conjuntivitis	1	
2	06/04/2019 20:21	H43.81	7	DVP	1	
1	06/04/2019 20:24	T26	9	Causticación	1	
2	06/04/2019 20:48	H16.0	1	Úlcera corneal	1	
1	06/04/2019 21:19	T15.0	1	Cuerpo extraño	1	
2	06/04/2019 21:21	G43.9	10	Migraña	1	
2	06/04/2019 22:03	H43.81	7	DVP	1	
1	06/04/2019 23:14	H04.32	5	Dacriocistitis aguda	1	
1	07/04/2019 0:23	T15.0	1	Cuerpo extraño	1	
2	07/04/2019 7:07	H11.3	0	Hiposfagma	1	
1	07/04/2019 8:07	H34.82	8	Trombosis venosa	1	
2	07/04/2019 8:35	H01.00	4	Blefaritis	1	
1	07/04/2019 9:21	T15.0	1	Cuerpo extraño	1	
1	07/04/2019 9:45	H16.0	1	Úlcera corneal	1	
1	07/04/2019 10:12	H10.3	0	Conjuntivitis	1	
2	07/04/2019 10:18	H10.3	0	Conjuntivitis	1	
2	07/04/2019 10:20	H53	11	No patología	1	
2	07/04/2019 10:21	H10.3	0	Conjuntivitis	1	
1	07/04/2019 10:25	H10.3	0	Conjuntivitis	1	
1	07/04/2019 11:12	H11.3	0	Hiposfagma	1	
2	07/04/2019 11:18	H11.3	0	Hiposfagma	1	
1	07/04/2019 11:21	H16.0	1	Úlcera corneal	1	
2	07/04/2019 11:37	H10.3	0	Conjuntivitis	1	
2	07/04/2019 11:55	H0.41	1	Ojo seco	1	
2	07/04/2019 12:06	H10.3	0	Conjuntivitis	1	
1	07/04/2019 12:12	H01.00	4	Blefaritis	1	
1	07/04/2019 12:14	H01.00	4	Blefaritis	1	
1	07/04/2019 12:23	H10.3	0	Conjuntivitis	1	
1	07/04/2019 12:44	H10.3	0	Conjuntivitis	1	
2	07/04/2019 12:49	H16.0	1	Úlcera corneal	1	
2	07/04/2019 13:49	H11.3	0	Hiposfagma	1	
2	07/04/2019 14:24	H16.9	1	Queratitis	1	
2	07/04/2019 14:34	H43.81	7	DVP	1	
1	07/04/2019 15:16	H16.0	1	Abceso corneal	1	
1	07/04/2019 15:52	H16.9	1	Queratitis	1	
2	07/04/2019 16:29	H16.9	1	Queratitis	1	
2	07/04/2019 17:02	B00.1	4	Dermatitis herpética	1	
2	07/04/2019 17:13	H43.81	7	DVP	1	
2	07/04/2019 17:49	H16.9	1	Queratitis	1	
2	07/04/2019 18:14	H20	6	Uveitis	1	
1	07/04/2019 18:19	H16.9	1	Queratitis	1	
2	07/04/2019 18:43	H16.0	1	Úlcera corneal	1	
1	07/04/2019 19:19	H35.3	8	Maculopatía	1	
1	07/04/2019 19:43	T15.0	1	Cuerpo extraño	1	
2	07/04/2019 20:01	H02.05	4	Distiquiasis	1	
1	07/04/2019 20:34	E11.319	8	Retinopatía diabética	1	
2	07/04/2019 21:20	H10.3	0	Conjuntivitis	1	
2	07/04/2019 22:16	H16.0	1	Úlcera corneal	1	
2	07/04/2019 23:17	H43.81	7	DVP	1	
1	07/04/2019 23:24	H16.0	1	Úlcera corneal	1	
2	08/04/2019 4:01	H53	11	No patología	1	
2	08/04/2019 7:19	H01.00	4	Blefaritis	1	
1	08/04/2019 9:01	H01.00	4	Blefaritis	1	
2	08/04/2019 9:40	H16.9	1	Queratitis	1	
1	08/04/2019 9:40	H43.1	7	Hemovítreo	1	
1	08/04/2019 9:42	H11.3	0	Hiposfagma	1	
2	08/04/2019 9:49	H16.9	1	Queratitis	1	
2	08/04/2019 9:59	H16.9	1	Queratitis	1	

2	08/04/2019 10:01	H43.81	7	DVP	1	
2	08/04/2019 10:21	H10.3	0	Conjuntivitis	1	
2	08/04/2019 10:28	H16.0	1	Úlcera corneal	1	
1	08/04/2019 10:43	H00.02	4	Orzuelo	1	
2	08/04/2019 10:58	H16.9	1	Queratitis	1	
2	08/04/2019 11:11	H01.00	4	Blefaritis	1	
1	08/04/2019 11:20	H10.3	0	Conjuntivitis	1	
2	08/04/2019 11:28	H16.9	1	Queratitis	1	
2	08/04/2019 11:38	H10.3	0	Conjuntivitis	1	
2	08/04/2019 11:45	H16.9	1	Queratitis	1	
1	08/04/2019 12:15	H10.3	0	Conjuntivitis	1	
2	08/04/2019 12:21	H16.9	1	Queratitis	1	
1	08/04/2019 12:25	H10.3	0	Conjuntivitis	1	
2	08/04/2019 12:31	H43.81	7	DVP	1	
2	08/04/2019 12:32	H11.3	1	Hiposfagma	1	
2	08/04/2019 12:34	T15.0	1	Cuerpo extraño	1	
2	08/04/2019 12:47	H16.9	1	Queratitis	1	
2	08/04/2019 12:52	H02	4	Lesión palpebral	1	
1	08/04/2019 13:25	H16.9	1	Queratitis	1	
2	08/04/2019 13:27	H16.9	1	Queratitis	1	
2	08/04/2019 14:33	H01.00	4	Blefaritis	1	
2	08/04/2019 14:37	H10.3	0	Conjuntivitis	1	
1	08/04/2019 15:05	H10.3	0	Conjuntivitis	1	
2	08/04/2019 15:28	H10.3	0	Conjuntivitis	1	
2	08/04/2019 15:39	H43.81	7	DVP	1	
2	08/04/2019 16:01	H0.41	1	Ojo seco	1	
1	08/04/2019 16:24	H11.3	0	Hiposfagma	1	
1	08/04/2019 16:25	H10.3	0	Conjuntivitis	1	
2	08/04/2019 16:28	H10.3	0	Conjuntivitis	1	
1	08/04/2019 16:32	H43.81	7	DVP	1	
2	08/04/2019 16:35	H10.3	0	Conjuntivitis	1	
1	08/04/2019 16:50	H10.3	0	Conjuntivitis	1	
1	08/04/2019 17:17	T15.0	1	Cuerpo extraño	1	
2	08/04/2019 17:21	H16.0	1	Úlcera corneal	1	
1	08/04/2019 17:33	H16.9	1	Queratitis	1	
1	08/04/2019 17:47	S05.9	9	Traumatismo	1	
2	08/04/2019 17:50	H16.9	1	Queratitis	1	
1	08/04/2019 17:59	B00.1	4	Dermatitis herpética	1	
2	08/04/2019 18:12	H11.3	1	Hiposfagma	1	
2	08/04/2019 18:16	H16.9	1	Queratitis	1	
2	08/04/2019 18:39	T15.0	1	Cuerpo extraño	1	
1	08/04/2019 18:56	H00.02	4	Orzuelo	1	
2	08/04/2019 19:10	H43.81	7	DVP	1	
2	08/04/2019 19:11	H16.9	1	Queratitis	1	
2	08/04/2019 19:19	H01.00	4	Blefaritis	1	
1	08/04/2019 19:21	H43.81	7	DVP	1	
2	08/04/2019 19:22	H26.9	2	Catarata	1	
1	08/04/2019 20:24	H16.0	1	Úlcera corneal	1	
2	08/04/2019 20:43	H26.9	2	Catarata	1	
2	08/04/2019 20:48	H10.3	0	Conjuntivitis	1	
2	08/04/2019 20:53	H10.3	0	Conjuntivitis	1	
1	08/04/2019 20:59	H16.9	1	Queratitis	1	
2	08/04/2019 21:09	H01.00	4	Blefaritis	1	
2	08/04/2019 21:26	H01.00	4	Blefaritis	1	
1	08/04/2019 22:36	T15.0	1	Cuerpo extraño	1	
1	08/04/2019 22:53	H26.9	2	Catarata	1	
1	08/04/2019 23:09	H10.3	0	Conjuntivitis	1	
2	08/04/2019 23:52	H02.05	4	Distiquiasis	1	
1	09/04/2019 0:11	S05.9	9	Traumatismo	1	
1	09/04/2019 8:30	H10.3	0	Conjuntivitis	1	
1	09/04/2019 8:34	H11.3	0	Hiposfagma	1	
2	09/04/2019 9:19	H01.00	4	Blefaritis	1	

1	09/04/2019 9:32	H27.8	2	OCP	1	
2	09/04/2019 10:30	H10.3	0	Conjuntivitis	1	
2	09/04/2019 10:45	H10.3	0	Conjuntivitis	1	
2	09/04/2019 10:54	H11.44	0	Quiste conjuntival	1	
2	09/04/2019 11:18	H43.81	7	DVP	1	
2	09/04/2019 11:22	H10.3	0	Conjuntivitis	1	
1	09/04/2019 12:23	H11.3	0	Hiposfagma	1	
2	09/04/2019 12:55	H11.3	0	Hiposfagma	1	
1	09/04/2019 13:00	H00.02	4	Orzuelo	1	
1	09/04/2019 13:02	H16.9	1	Queratitis	1	
2	09/04/2019 13:08	H16.9	1	Queratitis	1	
2	09/04/2019 13:22	H10.3	0	Conjuntivitis	1	
2	09/04/2019 13:23	H53.9	10	Alteraciones visuales	1	
2	09/04/2019 13:37	H10.3	0	Conjuntivitis	1	
2	09/04/2019 13:47	H43.81	7	DVP	1	
2	09/04/2019 15:36	H10.3	0	Conjuntivitis	1	
2	09/04/2019 15:36	H16.9	1	Queratitis	1	
2	09/04/2019 15:40	H33.30	8	Desgarro retiniano	1	
2	09/04/2019 15:50	H02	4	Lesión palpebral	1	
2	09/04/2019 16:18	H10.3	1	Conjuntivitis	1	
2	09/04/2019 16:39	H10.3	0	Conjuntivitis	1	
2	09/04/2019 17:03	H53.9	8	Alteraciones visuales	1	
2	09/04/2019 17:37	G43.109	10	Aura	1	
2	09/04/2019 17:44	H10.3	0	Conjuntivitis	1	
1	09/04/2019 18:17	H11.3	0	Hiposfagma	1	
2	09/04/2019 18:36	H10.3	0	Conjuntivitis	1	
2	09/04/2019 18:52	H33.30	8	Desgarro retiniano	1	
2	09/04/2019 19:06	H27.8	2	OCP	1	
2	09/04/2019 19:09	H53	11	No patología	1	
1	09/04/2019 19:23	T15.0	1	Cuerpo extraño	1	
1	09/04/2019 19:30	H10.3	0	Conjuntivitis	1	
2	09/04/2019 20:09	H16.9	1	Queratitis	1	
2	09/04/2019 20:16	H01.00	4	Blefaritis	1	
2	09/04/2019 20:22	H33.30	8	Desgarro retiniano	1	
1	09/04/2019 20:31	H16.0	1	Úlcera corneal	1	
1	09/04/2019 21:00	H10.3	0	Conjuntivitis	1	
2	09/04/2019 21:02	H16.0	1	Úlcera corneal	1	
2	09/04/2019 21:12	E11.319	8	Retinopatía diabética	1	
1	09/04/2019 21:27	T15.0	1	Cuerpo extraño	1	
2	09/04/2019 21:51	S05.9	9	Traumatismo	1	
2	09/04/2019 21:52	H15.00	6	Escleritis	1	
1	09/04/2019 22:03	T15.0	1	Cuerpo extraño	1	
1	09/04/2019 22:10	H02	4	Lesión palpebral	1	
2	09/04/2019 22:29	H16.9	1	Queratitis	1	
1	09/04/2019 22:33	T15.0	1	Cuerpo extraño	1	
2	09/04/2019 22:35	H16.0	1	Úlcera corneal	1	
1	09/04/2019 23:10	H10.3	0	Conjuntivitis	1	
1	09/04/2019 23:30	H11.3	1	Hiposfagma	1	
1	09/04/2019 23:48	T15.0	1	Cuerpo extraño	1	
1	10/04/2019 2:06	H16.0	1	Úlcera corneal	1	
1	10/04/2019 5:38	H16.9	1	Queratitis	1	
2	10/04/2019 8:20	H10.3	0	Conjuntivitis	1	
1	10/04/2019 8:22	H34.82	8	Trombosis venosa	1	
2	10/04/2019 8:26	H00.03	5	Celulitis preseptal	1	
1	10/04/2019 9:21	H35.0	8	Hemorragia retiniana	1	
1	10/04/2019 9:35	H20	6	Uveitis	1	
2	10/04/2019 9:42	H16.9	1	Queratitis	1	
2	10/04/2019 9:43	H10.3	0	Conjuntivitis	1	
1	10/04/2019 9:48	H16.9	1	Queratitis	1	
2	10/04/2019 10:20	H53	11	No patología	1	
2	10/04/2019 10:25	H35.30	8	DMAE	1	
1	10/04/2019 10:35	H16.0	1	Úlcera corneal	1	

2	10/04/2019 10:55	H10.3	0	Conjuntivitis	1	
2	10/04/2019 11:02	H10.3	0	Conjuntivitis	1	
2	10/04/2019 11:25	H10.3	0	Conjuntivitis	1	
2	10/04/2019 12:18	H16.9	1	Queratitis	1	
2	10/04/2019 12:39	H10.3	0	Conjuntivitis	1	
1	10/04/2019 12:59	T15.0	1	Cuerpo extraño	1	
1	10/04/2019 13:11	H20	6	Uveitis	1	
2	10/04/2019 13:21	H43.81	7	DVP	1	
2	10/04/2019 13:24	H16.0	1	Úlcera corneal	1	
2	10/04/2019 14:12	H00.02	4	Orzuelo	1	
2	10/04/2019 14:39	H16.0	1	Úlcera corneal	1	
2	10/04/2019 14:52	H16.9	1	Queratitis	1	
2	10/04/2019 14:55	H10.3	0	Conjuntivitis	1	
2	10/04/2019 15:20	H01.00	4	Blefaritis	1	
1	10/04/2019 15:45	H16.0	1	Úlcera corneal	1	
1	10/04/2019 15:49	H53.10	11	Problema refractivo	1	
2	10/04/2019 15:51	H43.81	7	DVP	1	
2	10/04/2019 15:53	H01.00	4	Blefaritis	1	
1	10/04/2019 15:59	H43.1	7	Hemovítreo	1	
2	10/04/2019 16:33	H10.3	0	Conjuntivitis	1	
2	10/04/2019 17:01	H16.9	1	Queratitis	1	
2	10/04/2019 18:05	H43.81	7	DVP	1	
2	10/04/2019 18:15	H43.81	7	DVP	1	
2	10/04/2019 18:25	H10.3	0	Conjuntivitis	1	
2	10/04/2019 18:42	H53.10	2	Problema refractivo	1	
1	10/04/2019 18:50	H43.81	7	DVP	1	
1	10/04/2019 20:01	H10.3	0	Conjuntivitis	1	
1	10/04/2019 20:03	T15.0	1	Cuerpo extraño	1	
1	10/04/2019 20:43	S05.9	9	Traumatismo	1	
2	10/04/2019 20:59	H16.9	1	Queratitis	1	
2	10/04/2019 21:08	H53	11	No patología	1	
1	10/04/2019 21:10	H33.0	8	Desprendimiento de retina	1	
1	10/04/2019 23:18	Alta por fuga	11	Alta por fuga	1	
1	10/04/2019 23:52	H10.3	0	Conjuntivitis	1	
1	11/04/2019 2:48	H16.9	1	Queratitis	1	
1	11/04/2019 2:51	H11.3	0	Hiposfagma	1	
1	11/04/2019 6:17	H15.1	6	Epiescleritis	1	
2	11/04/2019 8:42	H10.3	0	Conjuntivitis	1	
2	11/04/2019 9:24	H01.00	4	Blefaritis	1	
1	11/04/2019 9:39	H40.05	3	HTO	1	
2	11/04/2019 10:30	H10.3	0	Conjuntivitis	1	
1	11/04/2019 11:06	H16.9	1	Queratitis	1	
1	11/04/2019 11:06	B00.1	4	Dermatitis herpética	1	
1	11/04/2019 11:07	H16.9	1	Queratitis	1	
2	11/04/2019 11:35	H27.8	2	OCP	1	
1	11/04/2019 12:00	H26.9	2	Catarata	1	
2	11/04/2019 12:31	H43.81	7	DVP	1	
2	11/04/2019 13:09	H10.3	0	Conjuntivitis	1	
1	11/04/2019 13:48	H16.0	1	Úlcera corneal	1	
2	11/04/2019 13:49	H35.3	8	Maculopatía	1	
1	11/04/2019 14:22	H16.0	1	Úlcera corneal	1	
2	11/04/2019 14:54	H16.9	1	Queratitis	1	
1	11/04/2019 15:11	H16.9	1	Queratitis	1	
2	11/04/2019 15:22	H16.9	1	Queratitis	1	
2	11/04/2019 15:57	S05.9	8	Traumatismo	1	
1	11/04/2019 15:59	T15.0	1	Cuerpo extraño	1	
1	11/04/2019 16:05	T15.0	1	Cuerpo extraño	1	
2	11/04/2019 16:07	H43.1	7	Hemovítreo	1	
2	11/04/2019 16:07	H43.81	7	DVP	1	
2	11/04/2019 16:09	H10.3	0	Conjuntivitis	1	
2	11/04/2019 16:24	H10.3	0	Conjuntivitis	1	
1	11/04/2019 16:50	T15.0	1	Cuerpo extraño	1	

1	11/04/2019 16:58	H16.9	1	Queratitis	1	
2	11/04/2019 17:34	H26.9	2	Catarata	1	
1	11/04/2019 17:41	H10.3	0	Conjuntivitis	1	
2	11/04/2019 17:41	H53	11	No patología	1	
2	11/04/2019 18:13	H16.0	1	Úlcera corneal	1	
1	11/04/2019 18:17	S05.9	9	Traumatismo	1	
2	11/04/2019 18:31	H53	11	No patología	1	
2	11/04/2019 19:14	H43.81	7	DVP	1	
2	11/04/2019 19:18	H11.3	0	Hiposfagma	1	
1	11/04/2019 19:20	H10.3	0	Conjuntivitis	1	
1	11/04/2019 19:27	H00.02	4	Orzuelo	1	
2	11/04/2019 19:34	H10.3	0	Conjuntivitis	1	
1	11/04/2019 19:39	H10.3	0	Conjuntivitis	1	
1	11/04/2019 19:43	H04.41	5	Dacriocistitis crónica	1	
1	11/04/2019 20:00	H01.00	4	Blefaritis	1	
1	11/04/2019 20:47	T15.0	1	Cuerpo extraño	1	
1	11/04/2019 20:51	T15.0	1	Cuerpo extraño	1	
2	11/04/2019 20:56	H53	11	No patología	1	
2	11/04/2019 20:58	H16.9	1	Queratitis	1	
1	11/04/2019 21:05	H43.81	7	DVP	1	
1	11/04/2019 21:15	H16.9	1	Queratitis	1	
1	11/04/2019 21:29	H43.81	7	DVP	1	
2	11/04/2019 21:35	T15.0	1	Cuerpo extraño	1	
1	11/04/2019 21:38	H20	6	Uveitis	1	
1	11/04/2019 22:21	T15.0	1	Cuerpo extraño	1	
1	11/04/2019 22:25	T15.0	1	Cuerpo extraño	1	
1	11/04/2019 22:52	H16.9	1	Queratitis	1	
2	11/04/2019 23:57	H10.3	0	Conjuntivitis	1	
2	12/04/2019 0:23	H16.9	1	Queratitis	1	
1	12/04/2019 3:22	S05.9	9	Traumatismo	1	
1	12/04/2019 9:19	H01.00	4	Blefaritis	1	
1	12/04/2019 9:53	H16.9	1	Queratitis	1	
2	12/04/2019 9:55	H43.81	7	DVP	1	
1	12/04/2019 9:58	H16.9	1	Queratitis	1	
1	12/04/2019 10:21	S05.9	9	Traumatismo	1	
2	12/04/2019 10:47	H16.9	1	Queratitis	1	
1	12/04/2019 11:03	H01.00	4	Blefaritis	1	
1	12/04/2019 11:05	H43.81	7	DVP	1	
2	12/04/2019 11:24	H16.9	1	Queratitis	1	
1	12/04/2019 11:58	H10.3	0	Conjuntivitis	1	
2	12/04/2019 12:09	H16.9	1	Queratitis	1	
1	12/04/2019 12:52	H16.9	1	Queratitis	1	
1	12/04/2019 12:58	H16.0	1	Úlcera corneal	1	
1	12/04/2019 13:35	H18.20	1	Edema corneal	1	
2	12/04/2019 14:02	H53	11	No patología	1	
1	12/04/2019 15:18	H43.81	7	DVP	1	
2	12/04/2019 15:21	H10.3	0	Conjuntivitis	1	
2	12/04/2019 15:27	H10.3	0	Conjuntivitis	1	
1	12/04/2019 15:34	H59.3	5	Postop via lagrimal	1	
2	12/04/2019 16:15	H16.0	1	Úlcera corneal	1	
2	12/04/2019 16:29	H27.10	2	Luxación LIO	1	
1	12/04/2019 16:59	H43.1	7	Hemovítreo	1	
2	12/04/2019 17:22	H16.9	1	Queratitis	1	
2	12/04/2019 18:15	S05.9	9	Traumatismo	1	
1	12/04/2019 18:29	H16.0	1	Úlcera corneal	1	
2	12/04/2019 18:46	H18.50	1	Distrofia corneal	1	
2	12/04/2019 18:53	H43.81	7	DVP	1	
1	12/04/2019 19:09	H16.9	1	Queratitis	1	
2	12/04/2019 19:20	H16.9	1	Queratitis	1	
2	12/04/2019 19:25	H10.3	0	Conjuntivitis	1	
2	12/04/2019 19:26	H10.3	0	Conjuntivitis	1	
1	12/04/2019 19:46	H16.9	1	Queratitis	1	

1	12/04/2019 19:47	H10.3	0	Conjuntivitis	1	
1	12/04/2019 19:57	H01.00	4	Blefaritis	1	
2	12/04/2019 19:59	H16.0	1	Úlcera corneal	1	
2	12/04/2019 20:23	H43.81	7	DVP	1	
2	12/04/2019 20:45	H16.9	1	Queratitis	1	
1	12/04/2019 20:52	T15.0	1	Cuerpo extraño	1	
1	12/04/2019 21:28	H16.9	1	Queratitis	1	
2	12/04/2019 21:41	H00.02	4	Orzuelo	1	
1	12/04/2019 21:41	B00.1	11	Dermatitis herpética	1	
1	12/04/2019 21:42	H53	11	No patología	1	
1	12/04/2019 21:53	T15.0	1	Cuerpo extraño	1	
1	12/04/2019 21:54	T15.0	1	Cuerpo extraño	1	
1	12/04/2019 22:03	H16.9	1	Queratitis	1	
2	12/04/2019 22:10	H16.9	1	Queratitis	1	
2	12/04/2019 22:27	H11.3	0	Hiposfagma	1	
1	12/04/2019 22:49	H16.0	1	Úlcera corneal	1	
1	13/04/2019 0:10	S05.9	9	Traumatismo	1	
2	13/04/2019 3:50	H16.9	1	Queratitis	1	
1	13/04/2019 7:12	H16.0	1	Úlcera corneal	1	
1	13/04/2019 7:55	H16.0	1	Úlcera corneal	1	
1	13/04/2019 9:46	H10.3	0	Conjuntivitis	1	
2	13/04/2019 10:14	H00.02	4	Orzuelo	1	
2	13/04/2019 10:37	H43.81	7	DVP	1	
2	13/04/2019 10:51	H59.3	5	Postop via lagrimal	1	
2	13/04/2019 10:52	H00.19	4	Chalazión	1	
1	13/04/2019 11:12	H10.3	0	Conjuntivitis	1	
2	13/04/2019 11:15	H02	4	Alteración palpebral	1	
1	13/04/2019 11:46	H59.3	4	Post op párpados	1	
1	13/04/2019 11:58	T15.0	1	Cuerpo extraño	1	
1	13/04/2019 11:58	H16.0	1	Úlcera corneal	1	
2	13/04/2019 12:11	H00.03	5	Celulitis preseptal	1	
2	13/04/2019 12:24	H10.3	0	Conjuntivitis	1	
1	13/04/2019 12:51	H16.0	1	Úlcera corneal	1	
1	13/04/2019 13:06	S05.9	9	Traumatismo	1	
1	13/04/2019 13:52	H20	6	Uveitis	1	
1	13/04/2019 13:53	H33.0	8	Desprendimiento de retina	1	
2	13/04/2019 13:56	H11.3	0	Hiposfagma	1	
2	13/04/2019 14:25	B00.1	11	Dermatitis herpética	1	
1	13/04/2019 15:13	H16.0	1	Úlcera corneal	1	
2	13/04/2019 15:25	H10.3	0	Conjuntivitis	1	
1	13/04/2019 15:56	H16.0	1	Úlcera corneal	1	
1	13/04/2019 16:17	T15.0	1	Cuerpo extraño	1	
1	13/04/2019 16:46	T15.0	1	Cuerpo extraño	1	
2	13/04/2019 17:06	H16.0	1	Úlcera corneal	1	
1	13/04/2019 17:24	H10.3	0	Conjuntivitis	1	
1	13/04/2019 17:25	S05.30	9	Laceración conjuntival	1	
1	13/04/2019 17:38	H16.0	1	Úlcera corneal	1	
2	13/04/2019 17:38	H11.3	0	Hiposfagma	1	
1	13/04/2019 17:41	T15.0	1	Cuerpo extraño	1	
1	13/04/2019 17:41	H33.0	8	Desprendimiento de retina	1	
2	13/04/2019 17:50	H35.37	8	MER	1	
2	13/04/2019 18:18	H11.00	0	Pterigium	1	
2	13/04/2019 18:19	S05.9	9	Traumatismo	1	
2	13/04/2019 18:21	H43.81	7	DVP	1	
2	13/04/2019 18:39	H00.02	4	Orzuelo	1	
1	13/04/2019 18:39	H16.9	1	Queratitis	1	
1	13/04/2019 19:39	T15.0	1	Cuerpo extraño	1	
1	13/04/2019 20:04	T26	9	Causticación	1	
2	13/04/2019 20:30	S05.30	9	Laceración conjuntival	1	
1	13/04/2019 20:40	H43.81	7	DVP	1	
1	13/04/2019 20:49	H16.0	1	Úlcera corneal	1	
2	13/04/2019 21:12	H16.9	1	Queratitis	1	

1	13/04/2019 21:34	H11.3	0	Hiposfagma	1	
2	13/04/2019 22:28	H16.9	1	Queratitis	1	
1	13/04/2019 22:49	Alta por fuga	11	Alta por fuga	1	
1	13/04/2019 23:07	T15.0	1	Cuerpo extraño	1	
1	13/04/2019 23:27	B00.1	11	Dermatitis herpética	1	
2	13/04/2019 23:39	H53	11	No patología	1	
2	14/04/2019 4:09	H02	4	Alteración palpebral	1	
2	14/04/2019 8:48	H16.9	1	Queratitis	1	
2	14/04/2019 9:14	H02	4	Alteración palpebral	1	
1	14/04/2019 9:46	T15.0	1	Cuerpo extraño	1	
1	14/04/2019 10:07	H10.3	0	Conjuntivitis	1	
1	14/04/2019 10:31	H16.9	1	Queratitis	1	
1	14/04/2019 10:47	H44.009	8	Endoftalmitis	1	
2	14/04/2019 11:09	H11.44	1	Quiste conjuntival	1	
1	14/04/2019 11:23	T15.0	1	Cuerpo extraño	1	
1	14/04/2019 11:25	H10.3	0	Conjuntivitis	1	
2	14/04/2019 11:31	H11.3	0	Hiposfagma	1	
1	14/04/2019 11:35	T15.0	1	Cuerpo extraño	1	
2	14/04/2019 11:44	H16.9	1	Queratitis	1	
1	14/04/2019 12:17	H43.81	7	DVP	1	
2	14/04/2019 12:18	H43.81	7	DVP	1	
1	14/04/2019 12:21	H10.3	0	Conjuntivitis	1	
2	14/04/2019 12:36	H53	11	No patología	1	
2	14/04/2019 12:37	H26.9	2	Post op catarata	1	
2	14/04/2019 12:42	S05.9	1	Traumatismo	1	
1	14/04/2019 12:47	B00.1	4	Dermatitis herpética	1	
1	14/04/2019 13:50	H10.3	0	Conjuntivitis	1	
2	14/04/2019 14:46	H01.00	4	Blefaritis	1	
1	14/04/2019 14:47	H00.02	4	Orzuelo	1	
1	14/04/2019 15:02	H16.0	1	Úlcera corneal	1	
1	14/04/2019 16:00	H01.00	4	Blefaritis	1	
1	14/04/2019 16:02	H43.1	7	Hemovítreo	1	
2	14/04/2019 16:03	H53	11	No patología	1	
1	14/04/2019 16:15	H10.3	0	Conjuntivitis	1	
2	14/04/2019 16:52	H10.3	0	Conjuntivitis	1	
2	14/04/2019 17:08	H16.9	1	Queratitis	1	
2	14/04/2019 17:13	H33.30	8	Desgarro retiniano	1	
2	14/04/2019 17:39	T15.0	1	Cuerpo extraño	1	
1	14/04/2019 17:54	H16.0	1	Úlcera corneal	1	
2	14/04/2019 18:23	H10.3	0	Conjuntivitis	1	
2	14/04/2019 18:33	H16.9	1	Queratitis	1	
2	14/04/2019 18:37	H10.3	0	Conjuntivitis	1	
2	14/04/2019 20:15	H11.3	0	Hiposfagma	1	
2	14/04/2019 20:50	H01.00	4	Blefaritis	1	
1	14/04/2019 21:22	H10.3	0	Conjuntivitis	1	
2	14/04/2019 23:59	H10.3	0	Conjuntivitis	1	
2	15/04/2019 0:16	H16.9	1	Queratitis	1	
1	15/04/2019 0:41	H16.0	1	Úlcera corneal	1	
1	15/04/2019 1:12	H16.9	1	Queratitis	1	
2	15/04/2019 1:59	H16.9	1	Queratitis	1	
2	15/04/2019 2:50	H16.9	1	Queratitis	1	
1	15/04/2019 6:12	H10.3	0	Conjuntivitis	1	
1	15/04/2019 7:06	H10.3	0	Conjuntivitis	1	
2	15/04/2019 8:15	H53	11	No patología	1	
1	15/04/2019 8:32	H43.1	7	Hemovítreo	1	
2	15/04/2019 8:40	H10.3	0	Conjuntivitis	1	
2	15/04/2019 8:51	H20	6	Uveitis	1	
1	15/04/2019 8:58	H01.00	4	Blefaritis	1	
1	15/04/2019 9:02	H27.8	2	OCP	1	
2	15/04/2019 9:35	H43.81	7	DVP	1	
1	15/04/2019 9:44	H16.0	1	Úlcera corneal	1	
1	15/04/2019 10:06	H16.9	1	Queratitis	1	

2	15/04/2019 10:07	H10.3	0	Conjuntivitis	1	
1	15/04/2019 11:07	H35.30	8	DMAE	1	
2	15/04/2019 11:08	H27.8	2	OCP	1	
2	15/04/2019 11:10	H10.3	0	Conjuntivitis	1	
2	15/04/2019 11:31	H10.3	0	Conjuntivitis	1	
2	15/04/2019 12:30	H16.9	1	Queratitis	1	
1	15/04/2019 12:40	H43.81	7	DVP	1	
1	15/04/2019 12:41	H43.81	7	DVP	1	
2	15/04/2019 12:44	H53	11	No patología	1	
2	15/04/2019 12:56	H16.9	1	Queratitis	1	
2	15/04/2019 13:49	S05.30	9	Laceración conjuntival	1	
1	15/04/2019 15:01	H40.9	3	Glaucoma	1	
2	15/04/2019 15:44	H15.00	6	Epiescleritis	1	
1	15/04/2019 16:06	Alta por fuga	11	Alta por fuga	1	
2	15/04/2019 16:17	H00.02	3	Orzuelo	1	
2	15/04/2019 16:23	H16.0	1	Úlcera corneal	1	
2	15/04/2019 16:24	H43.81	7	DVP	1	
2	15/04/2019 16:40	H43.81	7	DVP	1	
2	15/04/2019 16:43	H10.3	0	Conjuntivitis	1	
2	15/04/2019 16:57	H10.3	0	Conjuntivitis	1	
2	15/04/2019 16:57	H16.0	1	Úlcera corneal	1	
2	15/04/2019 16:59	H10.3	0	Conjuntivitis	1	
2	15/04/2019 17:04	H43.81	7	DVP	1	
2	15/04/2019 17:08	H16.0	1	Úlcera corneal	1	
1	15/04/2019 17:46	H10.3	0	Conjuntivitis	1	
2	15/04/2019 18:08	H10.3	0	Conjuntivitis	1	
2	15/04/2019 18:12	H0.41	1	Ojo seco	1	
1	15/04/2019 18:14	H00.02	3	Orzuelo	1	
1	15/04/2019 18:36	H53	11	No patología	1	
2	15/04/2019 19:17	H10.3	0	Conjuntivitis	1	
2	15/04/2019 19:23	H00.02	3	Orzuelo	1	
1	15/04/2019 19:37	H11.3	0	Hiposfagma	1	
2	15/04/2019 20:08	H10.3	0	Conjuntivitis	1	
2	15/04/2019 20:37	H16.9	1	Queratitis	1	
2	15/04/2019 20:56	H16.0	1	Úlcera corneal	1	
2	15/04/2019 21:22	H00.03	4	Celulitis preseptal	1	
1	15/04/2019 21:25	H01.00	4	Blefaritis	1	
2	15/04/2019 21:31	H16.9	1	Queratitis	1	
1	15/04/2019 21:57	H43.81	7	DVP	1	
1	15/04/2019 22:12	H16.9	1	Queratitis	1	
1	15/04/2019 22:24	H16.9	1	Queratitis	1	
1	15/04/2019 22:29	T15.0	1	Cuerpo extraño	1	
2	15/04/2019 22:35	H16.9	1	Queratitis	1	
1	15/04/2019 23:10	T15.0	1	Cuerpo extraño	1	
1	15/04/2019 23:44	H11.3	0	Hiposfagma	1	
1	16/04/2019 2:09	H16.0	1	Úlcera corneal	1	
1	16/04/2019 4:18	T15.0	1	Cuerpo extraño	1	
2	16/04/2019 7:59	H43.81	7	DVP	1	
2	16/04/2019 8:13	T15.0	1	Cuerpo extraño	1	
2	16/04/2019 8:30	H10.3	0	Conjuntivitis	1	
1	16/04/2019 8:40	H16.9	1	Queratitis	1	
2	16/04/2019 9:24	H10.3	0	Conjuntivitis	1	
1	16/04/2019 9:30	H16.9	1	Queratitis	1	
1	16/04/2019 10:37	H18.20	1	Edema corneal	1	
2	16/04/2019 11:09	H16.9	1	Queratitis	1	
2	16/04/2019 12:00	H20	6	Uveitis	1	
2	16/04/2019 12:18	H10.3	0	Conjuntivitis	1	
2	16/04/2019 12:49	G43.109	11	Aura	1	
1	16/04/2019 14:50	H16.9	1	Queratitis	1	
2	16/04/2019 15:29	H16.9	1	Queratitis	1	
1	16/04/2019 15:30	H10.3	0	Conjuntivitis	1	
1	16/04/2019 15:40	H16.0	1	Úlcera corneal	1	

1	16/04/2019 17:10	H11.82	0	Conjuntivocalasia	1	
2	16/04/2019 17:21	H27.8	2	OCP	1	
2	16/04/2019 17:25	H53.10	11	Problema refractivo	1	
2	16/04/2019 17:50	H35.37	8	MER	1	
1	16/04/2019 18:53	H11.3	0	Hiposfagma	1	
2	16/04/2019 19:32	H15.00	6	Epiescleritis	1	
2	16/04/2019 19:36	G43.109	11	Aura	1	
1	16/04/2019 19:41	H10.3	0	Conjuntivitis	1	
2	16/04/2019 19:44	H10.3	0	Conjuntivitis	1	
1	16/04/2019 20:17	H16.0	1	Úlcera corneal	1	
2	16/04/2019 20:37	H16.9	1	Queratitis	1	
2	16/04/2019 20:51	H16.0	1	Úlcera corneal	1	
2	16/04/2019 21:20	H01.00	4	Blefaritis	1	
1	16/04/2019 22:43	T15.0	1	Cuerpo extraño	1	
2	16/04/2019 23:08	H16.9	1	Queratitis	1	
1	17/04/2019 0:04	T15.0	1	Cuerpo extraño	1	
1	17/04/2019 7:48	H10.3	0	Conjuntivitis	1	
2	17/04/2019 7:59	H11.3	0	Hiposfagma	1	
2	17/04/2019 9:06	H01.00	4	Blefaritis	1	
2	17/04/2019 9:07	S05.9	9	Traumatismo	1	
1	17/04/2019 9:37	H11.00	0	Pterigium	1	
2	17/04/2019 9:58	H10.3	0	Conjuntivitis	1	
2	17/04/2019 10:06	H16.9	1	Queratitis	1	
2	17/04/2019 10:07	H43.81	7	DVP	1	
1	17/04/2019 10:16	H11.3	0	Hiposfagma	1	
2	17/04/2019 10:22	H20	6	Uveitis	1	
2	17/04/2019 10:47	H59.3	4	Post op párpados	1	
2	17/04/2019 10:55	H10.81	0	Pingueculitis	1	
2	17/04/2019 11:13	H15.00	6	Epiescleritis	1	
2	17/04/2019 11:19	H43.81	7	DVP	1	
1	17/04/2019 11:26	H10.3	0	Conjuntivitis	1	
2	17/04/2019 11:55	H33.30	8	Desgarro retiniano	1	
2	17/04/2019 12:17	H11.3	0	Hiposfagma	1	
2	17/04/2019 12:29	H02	4	Lesión palpebral	1	
2	17/04/2019 13:01	H16.9	1	Queratitis	1	
2	17/04/2019 13:23	H43.81	7	DVP	1	
2	17/04/2019 13:44	H10.3	0	Conjuntivitis	1	
2	17/04/2019 14:26	H10.3	0	Conjuntivitis	1	
1	17/04/2019 15:13	H16.0	1	Úlcera corneal	1	
1	17/04/2019 15:15	B00.1	4	Dermatitis herpética	1	
2	17/04/2019 16:22	H16.9	1	Queratitis	1	
2	17/04/2019 16:22	H43.81	7	DVP	1	
1	17/04/2019 16:25	H16.0	1	Úlcera corneal	1	
1	17/04/2019 18:40	H43.81	7	DVP	1	
2	17/04/2019 18:45	H18.50	1	Distrofia corneal	1	
2	17/04/2019 18:46	H27.8	2	OCP	1	
2	17/04/2019 19:07	H10.3	0	Conjuntivitis	1	
2	17/04/2019 19:11	H43.81	7	DVP	1	
2	17/04/2019 19:29	H16.0	1	Úlcera corneal	1	
2	17/04/2019 20:00	H04.32	5	Dacriocistitis aguda	1	
1	17/04/2019 20:54	H16.0	1	Úlcera corneal	1	
1	17/04/2019 20:57	H10.3	0	Conjuntivitis	1	
1	17/04/2019 21:05	H10.3	0	Conjuntivitis	1	
2	17/04/2019 21:17	T15.0	1	Cuerpo extraño	1	
1	17/04/2019 22:40	H43.1	7	Hemovítreo	1	
1	17/04/2019 22:41	H16.0	1	Úlcera corneal	1	
2	17/04/2019 23:01	H16.9	1	Queratitis	1	
2	17/04/2019 23:39	H20	6	Uveitis	1	
1	17/04/2019 23:42	S05.9	9	Traumatismo	1	
2	18/04/2019 2:09	H16.9	1	Queratitis	1	
1	18/04/2019 3:43	H16.0	1	Úlcera corneal	1	
2	18/04/2019 9:17	H16.0	1	Úlcera corneal	1	

1	18/04/2019 9:56	H10.3	0	Conjuntivitis	1	
2	18/04/2019 10:02	H16.9	1	Queratitis	1	
1	18/04/2019 10:14	H10.3	0	Conjuntivitis	1	
1	18/04/2019 10:21	H43.81	7	DVP	1	
1	18/04/2019 10:53	H11.3	0	Hiposfagma	1	
2	18/04/2019 11:13	H16.0	1	Úlcera corneal	1	
1	18/04/2019 11:25	H43.81	7	DVP	1	
2	18/04/2019 11:27	H10.3	0	Conjuntivitis	1	
1	18/04/2019 11:33	H16.9	1	Queratitis	1	
1	18/04/2019 11:48	T15.0	1	Cuerpo extraño	1	
2	18/04/2019 11:57	H10.3	0	Conjuntivitis	1	
1	18/04/2019 12:04	H43.81	7	DVP	1	
1	18/04/2019 12:28	H11.3	0	hIPOsfagma	1	
1	18/04/2019 12:43	H16.0	1	Úlcera corneal	1	
2	18/04/2019 12:47	T15.0	1	Cuerpo extraño	1	
2	18/04/2019 13:00	H10.3	0	Conjuntivitis	1	
2	18/04/2019 13:16	H11.3	1	Hiposfagma	1	
2	18/04/2019 14:59	H10.3	0	Conjuntivitis	1	
2	18/04/2019 15:02	H10.3	0	Conjuntivitis	1	
2	18/04/2019 15:03	H10.3	0	Conjuntivitis	1	
2	18/04/2019 15:42	S05.9	9	Traumatismo	1	
1	18/04/2019 15:54	H11.3	0	Hiposfagma	1	
2	18/04/2019 16:30	H10.3	0	Conjuntivitis	1	
2	18/04/2019 17:08	H43.81	7	DVP	1	
2	18/04/2019 17:42	H33.0	8	Desprendimiento de retina	1	
1	18/04/2019 18:00	S05.9	9	Traumatismo	1	
1	18/04/2019 18:29	H16.0	1	Úlcera corneal	1	
1	18/04/2019 18:30	H10.3	0	Conjuntivitis	1	
2	18/04/2019 18:46	H16.0	1	Úlcera corneal	1	
1	18/04/2019 19:03	H43.81	7	DVP	1	
1	18/04/2019 19:36	H15.00	6	Epiescleritis	1	
2	18/04/2019 19:37	H53	11	No patología	1	
1	18/04/2019 20:53	H40.05	3	HTO	1	
2	18/04/2019 21:30	H35.3	8	Maculopatía	1	
2	18/04/2019 23:33	H10.81	0	Pingueculitis	1	
2	19/04/2019 0:28	H10.3	0	Conjuntivitis	1	
2	19/04/2019 0:45	Alta por fuga	11	Alta por fuga	1	
1	19/04/2019 9:18	H16.9	1	Queratitis	1	
2	19/04/2019 9:33	H10.3	0	Conjuntivitis	1	
2	19/04/2019 10:49	H26.9	2	Catarata	1	
1	19/04/2019 10:52	H16.0	1	Úlcera corneal	1	
2	19/04/2019 11:07	H01.00	4	Blefaritis	1	
1	19/04/2019 11:29	H04.32	5	Dacriocistitis aguda	1	
2	19/04/2019 11:35	H43.81	7	DVP	1	
2	19/04/2019 11:38	H10.3	0	Conjuntivitis	1	
1	19/04/2019 11:39	H53	11	No patología	1	
1	19/04/2019 11:42	H53	11	No patología	1	
2	19/04/2019 11:58	H27.8	2	OCP	1	
1	19/04/2019 12:10	H10.3	0	Conjuntivitis	1	
1	19/04/2019 12:11	H16.0	1	Úlcera corneal	1	
2	19/04/2019 12:14	H10.3	0	Conjuntivitis	1	
1	19/04/2019 12:22	H10.3	0	Conjuntivitis	1	
1	19/04/2019 12:25	T15.0	1	Cuerpo extraño	1	
2	19/04/2019 12:30	H10.3	0	Conjuntivitis	1	
1	19/04/2019 12:41	H16.0	1	Úlcera corneal	1	
2	19/04/2019 12:47	H43.81	7	DVP	1	
2	19/04/2019 12:50	H40.05	3	HTO	1	
2	19/04/2019 13:11	H10.3	0	Conjuntivitis	1	
1	19/04/2019 13:36	H10.3	0	Conjuntivitis	1	
2	19/04/2019 13:46	H10.3	0	Conjuntivitis	1	
2	19/04/2019 13:50	H16.0	1	Úlcera corneal	1	
1	19/04/2019 14:10	H16.0	1	Úlcera corneal	1	

1	19/04/2019 14:13	H15.00	6	Epiescleritis	1	
1	19/04/2019 14:14	H10.3	0	Conjuntivitis	1	
1	19/04/2019 14:26	H10.3	0	Conjuntivitis	1	
2	19/04/2019 14:48	H10.3	0	Conjuntivitis	1	
2	19/04/2019 15:06	H11.3	0	Hiposfagma	1	
2	19/04/2019 16:00	H10.3	0	Conjuntivitis	1	
1	19/04/2019 16:03	H10.3	0	Conjuntivitis	1	
1	19/04/2019 16:12	H16.0	1	Úlcera corneal	1	
2	19/04/2019 16:55	H11.3	4	Hiposfagma	1	
2	19/04/2019 16:55	H01.00	1	Blefaritis	1	
1	19/04/2019 17:37	H16.9	1	Queratitis	1	
2	19/04/2019 18:02	H16.9	0	Queratitis	1	
2	19/04/2019 18:05	H10.3	0	Conjuntivitis	1	
1	19/04/2019 18:56	H10.3	0	Conjuntivitis	1	
1	19/04/2019 18:57	T26	9	Causticación	1	
2	19/04/2019 19:15	H10.3	0	Conjuntivitis	1	
1	19/04/2019 19:46	H16.0	1	Úlcera corneal	1	
2	19/04/2019 23:17	H00.02	4	Orzuelo	1	
1	19/04/2019 23:30	H10.3	0	Conjuntivitis	1	
1	20/04/2019 0:30	H11.3	0	Hiposfagma	1	
1	20/04/2019 2:37	H16.9	1	Queratitis	1	
2	20/04/2019 6:43	H20	6	Uveitis	1	
2	20/04/2019 8:52	H16.9	1	Queratitis	1	
1	20/04/2019 8:55	H16.9	1	Queratitis	1	
2	20/04/2019 9:29	H10.3	0	Conjuntivitis	1	
2	20/04/2019 10:08	H53	11	No patología	1	
2	20/04/2019 10:28	H10.3	0	Conjuntivitis	1	
2	20/04/2019 10:36	H00.02	4	Orzuelo	1	
2	20/04/2019 10:44	H10.3	0	Conjuntivitis	1	
2	20/04/2019 10:49	H11.3	0	Hiposfagma	1	
1	20/04/2019 11:08	H16.9	1	Queratitis	1	
2	20/04/2019 11:15	H10.3	0	Conjuntivitis	1	
1	20/04/2019 12:01	H00.02	4	Orzuelo	1	
2	20/04/2019 12:12	T15.0	1	Cuerpo extraño	1	
2	20/04/2019 12:19	H53	11	No patología	1	
1	20/04/2019 12:29	H10.3	0	Conjuntivitis	1	
2	20/04/2019 12:57	T15.0	1	Cuerpo extraño	1	
1	20/04/2019 13:56	H02.05	4	Distiquiasis	1	
1	20/04/2019 13:58	H10.3	1	Conjuntivitis	1	
2	20/04/2019 15:05	H11.44	0	Quiste conjuntival	1	
1	20/04/2019 15:09	H43.81	7	DVP	1	
2	20/04/2019 15:23	H16.0	1	Úlcera corneal	1	
2	20/04/2019 16:15	H53.10	10	Problema refractivo	1	
2	20/04/2019 16:41	H00.02	4	Orzuelo	1	
1	20/04/2019 17:02	H10.3	0	Conjuntivitis	1	
1	20/04/2019 17:10	H00.02	4	Orzuelo	1	
2	20/04/2019 17:12	H10.3	0	Conjuntivitis	1	
1	20/04/2019 17:23	T15.0	1	Cuerpo extraño	1	
2	20/04/2019 17:37	H16.9	1	Queratitis	1	
2	20/04/2019 17:51	H43.81	7	DVP	1	
1	20/04/2019 17:54	H10.3	0	Conjuntivitis	1	
1	20/04/2019 18:24	H00.02	4	Orzuelo	1	
1	20/04/2019 19:06	H10.3	0	Conjuntivitis	1	
2	20/04/2019 19:26	H27.8	2	OCP	1	
1	20/04/2019 21:10	H00.02	4	Orzuelo	1	
2	20/04/2019 21:20	H10.3	0	Conjuntivitis	1	
2	20/04/2019 22:22	H10.3	0	Conjuntivitis	1	
2	20/04/2019 22:50	H10.3	0	Conjuntivitis	1	
1	20/04/2019 23:13	H16.0	1	Úlcera corneal	1	
1	20/04/2019 23:59	H10.3	0	Conjuntivitis	1	
2	21/04/2019 0:29	T15.0	1	Cuerpo extraño	1	
2	21/04/2019 1:54	T15.0	1	Cuerpo extraño	1	

2	21/04/2019 2:43	H40.9	3	Glaucoma facomórfico	1	
2	21/04/2019 6:13	H10.3	0	Conjuntivitis	1	
2	21/04/2019 9:38	H16.0	1	Úlcera corneal	1	
1	21/04/2019 9:39	H16.9	1	Queratitis	1	
1	21/04/2019 10:22	H10.3	0	Conjuntivitis	1	
2	21/04/2019 11:02	H43.81	7	DVP	1	
1	21/04/2019 11:49	H16.0	1	Úlcera corneal	1	
1	21/04/2019 13:19	H00.02	4	Orzuelo	1	
2	21/04/2019 13:27	H16.9	1	Queratitis	1	
2	21/04/2019 14:06	H17.8	1	Infiltrados corneales	1	
1	21/04/2019 14:08	H10.3	0	Conjuntivitis	1	
1	21/04/2019 14:51	H20	6	Uveitis	1	
2	21/04/2019 14:55	H16.0	1	Úlcera corneal	1	
1	21/04/2019 15:26	H10.3	0	Conjuntivitis	1	
1	21/04/2019 15:41	H17.8	1	Infiltrados corneales	1	
2	21/04/2019 16:15	T15.0	1	Cuerpo extraño	1	
1	21/04/2019 16:30	H16.9	1	Queratitis	1	
2	21/04/2019 16:32	H20	6	Uveitis	1	
1	21/04/2019 18:08	H16.0	1	Úlcera corneal	1	
2	21/04/2019 18:38	H16.9	1	Queratitis	1	
1	21/04/2019 19:27	H00.02	4	Orzuelo	1	
2	21/04/2019 19:34	H53	11	No patología	1	
1	21/04/2019 19:48	H10.3	0	Conjuntivitis	1	
2	21/04/2019 19:48	T15.0	1	Cuerpo extraño	1	
2	21/04/2019 19:56	H16.0	1	Úlcera corneal	1	
2	21/04/2019 20:01	H10.3	0	Conjuntivitis	1	
1	21/04/2019 20:56	H10.3	0	Conjuntivitis	1	
1	21/04/2019 21:07	H10.3	0	Conjuntivitis	1	
2	21/04/2019 21:27	H16.0	1	Úlcera corneal	1	
1	21/04/2019 22:47	S05.9	9	Traumatismo	1	
1	21/04/2019 22:59	H10.3	0	Conjuntivitis	1	
1	21/04/2019 23:03	H10.3	0	Conjuntivitis	1	
2	21/04/2019 23:55	H16.9	1	Queratitis	1	
2	22/04/2019 0:33	H11.3	0	Hiposfagma	1	
2	22/04/2019 0:46	H16.0	1	Úlcera corneal	1	
2	22/04/2019 1:49	H10.3	0	Conjuntivitis	1	
1	22/04/2019 6:22	S05.9	9	Traumatismo	1	
2	22/04/2019 8:36	H16.0	1	Úlcera corneal	1	
2	22/04/2019 9:27	H16.9	1	Queratitis	1	
1	22/04/2019 9:52	H11.3	0	Hiposfagma	1	
2	22/04/2019 10:18	H02	4	Alteración palpebral	1	
2	22/04/2019 10:26	T26	9	Causticación	1	
2	22/04/2019 10:41	H00.02	4	Orzuelo	1	
2	22/04/2019 10:47	T15.0	1	Cuerpo extraño	1	
1	22/04/2019 10:57	H01.00	4	Blefaritis	1	
2	22/04/2019 11:05	H27.8	2	OCP	1	
2	22/04/2019 11:31	H16.0	1	Úlcera corneal	1	
2	22/04/2019 11:37	H15.1	5	Epiescleritis	1	
1	22/04/2019 11:45	H53	11	No patología	1	
1	22/04/2019 12:11	T15.0	1	Cuerpo extraño	1	
1	22/04/2019 12:22	H10.3	0	Conjuntivitis	1	
2	22/04/2019 12:24	H16.0	1	Úlcera corneal	1	
2	22/04/2019 12:38	H16.9	1	Queratitis	1	
2	22/04/2019 12:52	H16.9	1	Queratitis	1	
1	22/04/2019 12:54	H02	4	Alteración palpebral	1	
2	22/04/2019 13:01	H10.3	0	Conjuntivitis	1	
2	22/04/2019 13:02	H43.81	7	DVP	1	
2	22/04/2019 13:10	B00.1	4	Dermatitis herpética	1	
2	22/04/2019 13:37	H01.00	4	Blefaritis	1	
2	22/04/2019 13:53	T15.0	1	Cuerpo extraño	1	
1	22/04/2019 14:22	S05.30	0	Laceración conjuntival	1	
1	22/04/2019 14:39	H01.00	4	Blefaritis	1	

1	22/04/2019 14:48	H16.9	1	Queratitis	1	
2	22/04/2019 15:00	T15.0	1	Cuerpo extraño	1	
1	22/04/2019 15:07	H16.0	1	Úlcera corneal	1	
2	22/04/2019 15:13	H16.9	1	Queratitis	1	
1	22/04/2019 15:30	H10.3	0	Conjuntivitis	1	
2	22/04/2019 15:37	H16.9	1	Queratitis	1	
2	22/04/2019 15:55	H26.9	2	Catarata	1	
1	22/04/2019 16:26	T15.0	1	Cuerpo extraño	1	
1	22/04/2019 16:42	T15.0	1	Cuerpo extraño	1	
2	22/04/2019 16:54	H16.9	1	Queratitis	1	
1	22/04/2019 16:59	T15.0	1	Cuerpo extraño	1	
2	22/04/2019 17:04	H11.3	0	Hiposfagma	1	
2	22/04/2019 17:13	H10.3	0	Conjuntivitis	1	
1	22/04/2019 17:15	H16.9	1	Queratitis	1	
1	22/04/2019 18:11	H16.0	1	Úlcera corneal	1	
1	22/04/2019 18:18	H43.81	7	DVP	1	
2	22/04/2019 18:26	H16.0	1	Úlcera corneal	1	
1	22/04/2019 18:34	H15.1	5	Epiescleritis	1	
1	22/04/2019 19:03	H43.81	7	DVP	1	
1	22/04/2019 19:27	T15.0	1	Cuerpo extraño	1	
2	22/04/2019 20:23	H15.1	7	Epiescleritis	1	
1	22/04/2019 20:49	T15.0	1	Cuerpo extraño	1	
1	22/04/2019 22:10	H16.9	1	Queratitis	1	
2	22/04/2019 23:17	H16.9	1	Queratitis	1	
2	22/04/2019 23:27	H10.3	0	Conjuntivitis	1	
1	23/04/2019 1:32	T15.0	1	Cuerpo extraño	1	
2	23/04/2019 8:02	H16.9	1	Queratitis	1	
1	23/04/2019 8:14	H20	6	Uveitis	1	
1	23/04/2019 8:54	H16.9	1	Queratitis	1	
2	23/04/2019 9:14	H43.81	7	DVP	1	
2	23/04/2019 9:38	H16.9	1	Queratitis	1	
1	23/04/2019 9:44	H10.3	0	Conjuntivitis	1	
2	23/04/2019 9:54	H34.82	8	Trombosis venosa	1	
2	23/04/2019 9:56	H16.9	1	Queratitis	1	
1	23/04/2019 9:59	H16.0	1	Úlcera corneal	1	
2	23/04/2019 10:06	H33.30	8	Desgarro retiniano	1	
1	23/04/2019 10:09	H02	4	Alteración palpebral	1	
2	23/04/2019 10:21	H26.9	2	Catarata	1	
2	23/04/2019 10:59	H10.3	0	Conjuntivitis	1	
1	23/04/2019 11:24	H10.3	0	Conjuntivitis	1	
1	23/04/2019 11:30	H01.00	4	Blefaritis	1	
1	23/04/2019 11:42	H27.8	2	OCP	1	
2	23/04/2019 11:56	H10.3	0	Conjuntivitis	1	
2	23/04/2019 11:57	H10.3	0	Conjuntivitis	1	
2	23/04/2019 12:01	H02	4	Lesión palpebral	1	
1	23/04/2019 12:25	H17.8	1	Infiltrados corneales	1	
2	23/04/2019 13:19	G43.109	10	Aura	1	
1	23/04/2019 13:45	H02	4	Lesión palpebral	1	
1	23/04/2019 14:28	G45.3	10	Amaurosis fugax	1	
2	23/04/2019 14:39	H01.00	4	Blefaritis	1	
2	23/04/2019 14:45	G43.109	10	Aura	1	
2	23/04/2019 15:12	H16.0	1	Úlcera corneal	1	
1	23/04/2019 15:21	H20	6	Uveitis	1	
2	23/04/2019 15:28	H10.3	0	Conjuntivitis	1	
2	23/04/2019 15:43	H16.9	1	Queratitis	1	
1	23/04/2019 16:56	H16.0	1	Abceso corneal	1	
2	23/04/2019 17:01	H02	4	Alteración palpebral	1	
1	23/04/2019 17:04	H10.3	0	Conjuntivitis	1	
1	23/04/2019 17:17	H34.82	8	Trombosis venosa	1	
2	23/04/2019 18:13	H43.1	7	Hemovítreo	1	
2	23/04/2019 18:25	H35.3	8	Maculopatía	1	
2	23/04/2019 18:36	H50.9	10	Estrabismo	1	

2	23/04/2019 18:52	H01.00	4	Blefaritis	1	
1	23/04/2019 18:54	H43.81	7	DVP	1	
2	23/04/2019 18:57	H11.3	0	Hiposfagma	1	
1	23/04/2019 19:12	H33.30	8	Desgarro retiniano	1	
1	23/04/2019 20:00	H01.00	4	Blefaritis	1	
2	23/04/2019 20:09	H16.0	1	Úlcera corneal	1	
1	23/04/2019 20:14	S05.9	9	Traumatismo	1	
2	23/04/2019 20:24	H16.9	1	Queratitis	1	
2	23/04/2019 20:44	H16.9	1	Queratitis	1	
2	23/04/2019 20:57	S05.9	9	Traumatismo	1	
1	23/04/2019 21:00	H11.44	0	Quiste conjuntival	1	
2	23/04/2019 23:29	H10.3	0	Conjuntivitis	1	
2	23/04/2019 23:41	H43.81	7	DVP	1	
2	24/04/2019 4:15	H16.9	1	Queratitis	1	
1	24/04/2019 4:48	H10.3	0	Conjuntivitis	1	
1	24/04/2019 8:08	H10.3	0	Conjuntivitis	1	
2	24/04/2019 9:18	H16.9	1	Queratitis	1	
2	24/04/2019 9:50	H10.3	0	Conjuntivitis	1	
1	24/04/2019 9:54	H10.3	0	Conjuntivitis	1	
1	24/04/2019 10:05	H11.3	0	Hiposfagma	1	
2	24/04/2019 10:19	H02	4	Alteración palpebral	1	
1	24/04/2019 10:22	H47.10	10	Papiledema	1	
2	24/04/2019 10:38	H11.3	0	Hiposfagma	1	
2	24/04/2019 10:40	G43.109	10	Aura	1	
2	24/04/2019 10:54	H16.9	1	Queratitis	1	
1	24/04/2019 11:11	H18.20	1	Edema corneal	1	
2	24/04/2019 11:53	G43.109	10	Aura	1	
2	24/04/2019 12:01	H10.3	0	Conjuntivitis	1	
2	24/04/2019 12:25	H27.8	2	OCP	1	
2	24/04/2019 12:51	H11.3	0	Hiposfagma	1	
2	24/04/2019 13:01	H16.9	1	Queratitis	1	
1	24/04/2019 13:03	H16.9	1	Queratitis	1	
2	24/04/2019 14:13	H20	6	Uveitis	1	
2	24/04/2019 14:17	H16.9	1	Queratitis	1	
2	24/04/2019 14:35	H10.3	0	Conjuntivitis	1	
1	24/04/2019 14:40	H26.9	2	Catarata	1	
2	24/04/2019 15:04	H59.3	4	Post op párpados	1	
2	24/04/2019 15:16	T15.0	1	Cuerpo extraño	1	
2	24/04/2019 15:45	H16.9	1	Queratitis	1	
1	24/04/2019 16:00	H16.9	1	Queratitis	1	
1	24/04/2019 16:01	H00.02	4	Orzuelo	1	
2	24/04/2019 16:12	H11.3	0	Hiposfagma	1	
2	24/04/2019 16:17	H43.81	7	DVP	1	
2	24/04/2019 16:32	H10.3	0	Conjuntivitis	1	
1	24/04/2019 16:38	H35.3	8	Maculopatía	1	
2	24/04/2019 16:48	H15.1	6	Epiescleritis	1	
2	24/04/2019 17:41	H10.81	0	Pingueculitis	1	
1	24/04/2019 17:57	H16.0	1	Úlcera corneal	1	
2	24/04/2019 18:00	B00.1	4	Dermatitis herpética	1	
2	24/04/2019 18:06	H16.9	1	Queratitis	1	
2	24/04/2019 18:14	H43.81	7	DVP	1	
2	24/04/2019 18:21	H43.81	7	DVP	1	
1	24/04/2019 18:56	H16.0	1	Úlcera corneal	1	
1	24/04/2019 19:32	H10.3	0	Conjuntivitis	1	
1	24/04/2019 20:30	T15.0	1	Cuerpo extraño	1	
2	24/04/2019 21:03	T26	9	Causticación	1	
2	24/04/2019 21:05	H01.00	4	Blefaritis	1	
1	24/04/2019 21:16	H53	11	No patología	1	
2	24/04/2019 21:56	H43.81	7	DVP	1	
1	24/04/2019 21:59	H10.3	0	Conjuntivitis	1	
1	24/04/2019 22:12	H11.3	0	Hiposfagma	1	
2	24/04/2019 22:45	H11.3	0	Hiposfagma	1	

2	24/04/2019 23:35	H10.3	0	Conjuntivitis	1	
2	25/04/2019 1:42	H53	11	No patología	1	
1	25/04/2019 7:16	H10.3	0	Conjuntivitis	1	
1	25/04/2019 8:39	H01.00	4	Blefaritis	1	
1	25/04/2019 9:10	T15.0	1	Cuerpo extraño	1	
2	25/04/2019 9:18	H10.3	0	Conjuntivitis	1	
2	25/04/2019 9:34	H20	6	Uveitis	1	
2	25/04/2019 10:34	H16.9	1	Queratitis	1	
2	25/04/2019 10:37	H16.9	1	Queratitis	1	
1	25/04/2019 10:44	H26.9	2	Post op catarata	1	
1	25/04/2019 10:55	H00.02	4	Orzuelo	1	
1	25/04/2019 11:04	H16.0	1	Úlcera corneal	1	
2	25/04/2019 11:13	H11.3	0	Hiposfagma	1	
2	25/04/2019 11:17	H43.1	7	Hemovítreo	1	
1	25/04/2019 11:18	H00.02	4	Orzuelo	1	
1	25/04/2019 11:27	H16.9	1	Queratitis	1	
2	25/04/2019 12:29	H16.9	1	Queratitis	1	
1	25/04/2019 12:30	H00.02	4	Orzuelo	1	
2	25/04/2019 12:32	H16.9	1	Queratitis	1	
2	25/04/2019 12:52	H35.3	8	Maculopatía	1	
2	25/04/2019 12:53	H01.00	4	Blefaritis	1	
2	25/04/2019 13:00	H01.00	4	Blefaritis	1	
2	25/04/2019 13:22	H15.1	6	Epiescleritis	1	
1	25/04/2019 13:38	H16.9	1	Queratitis	1	
2	25/04/2019 13:50	H10.3	0	Conjuntivitis	1	
1	25/04/2019 14:29	H35.3	8	Maculopatía	1	
1	25/04/2019 14:35	H10.3	0	Conjuntivitis	1	
1	25/04/2019 14:44	S05.9	9	Traumatismo	1	
2	25/04/2019 15:20	H16.9	1	Queratitis	1	
2	25/04/2019 15:53	H53	11	No patología	1	
1	25/04/2019 15:56	H01.00	4	Blefaritis	1	
2	25/04/2019 16:29	H10.3	0	Conjuntivitis	1	
1	25/04/2019 17:14	H10.3	0	Conjuntivitis	1	
2	25/04/2019 17:45	H11.3	0	Hiposfagma	1	
2	25/04/2019 18:04	H10.3	0	Conjuntivitis	1	
1	25/04/2019 18:12	T15.0	1	Cuerpo extraño	1	
2	25/04/2019 18:25	H10.3	0	Conjuntivitis	1	
2	25/04/2019 18:36	H10.3	0	Conjuntivitis	1	
2	25/04/2019 18:47	H16.9	1	Queratitis	1	
1	25/04/2019 19:12	H40.05	4	HTO	1	
2	25/04/2019 19:46	H16.9	1	Queratitis	1	
2	25/04/2019 19:53	H43.81	7	DVP	1	
1	25/04/2019 20:11	H33.0	8	Desprendimiento de retina	1	
1	25/04/2019 21:28	H11.3	0	Hiposfagma	1	
2	25/04/2019 21:32	H16.9	1	Queratitis	1	
1	25/04/2019 21:38	T15.0	1	Cuerpo extraño	1	
2	25/04/2019 22:13	H01.00	4	Blefaritis	1	
1	25/04/2019 23:32	H16.9	1	Queratitis	1	
1	25/04/2019 23:59	T15.0	1	Cuerpo extraño	1	
2	26/04/2019 0:07	H16.0	1	Úlcera corneal	1	
2	26/04/2019 0:15	H16.9	1	Queratitis	1	
1	26/04/2019 1:42	S05.9	9	Traumatismo	1	
2	26/04/2019 1:48	T15.0	1	Cuerpo extraño	1	
2	26/04/2019 2:50	H00.02	4	Orzuelo	1	
1	26/04/2019 3:59	T15.0	1	Cuerpo extraño	1	
2	26/04/2019 8:36	H43.1	7	Hemovítreo	1	
1	26/04/2019 9:37	H16.0	1	Úlcera corneal	1	
2	26/04/2019 9:58	H10.3	0	Conjuntivitis	1	
2	26/04/2019 10:16	H10.3	0	Conjuntivitis	1	
2	26/04/2019 10:16	H47.32	10	Drusas de nervio óptico	1	
1	26/04/2019 10:27	H43.81	7	DVP	1	
2	26/04/2019 10:28	H43.81	7	DVP	1	

2	26/04/2019 10:45	H01.00	4	Blefaritis	1	
1	26/04/2019 10:46	H01.00	4	Blefaritis	1	
2	26/04/2019 10:47	H01.00	4	Blefaritis	1	
2	26/04/2019 10:56	H10.3	0	Conjuntivitis	1	
1	26/04/2019 11:21	H10.3	0	Conjuntivitis	1	
2	26/04/2019 11:51	H16.9	1	Queratitis	1	
1	26/04/2019 12:11	H10.3	0	Conjuntivitis	1	
2	26/04/2019 12:42	H11.3	0	Hiposfagma	1	
2	26/04/2019 12:49	H11.3	0	Hiposfagma	1	
2	26/04/2019 13:18	H53	11	No patología	1	
2	26/04/2019 13:52	H11.00	0	Pterigium	1	
2	26/04/2019 15:07	H16.9	1	Queratitis	1	
1	26/04/2019 15:12	H02.05	4	Distiquiasis	1	
1	26/04/2019 15:21	H10.3	0	Conjuntivitis	1	
1	26/04/2019 16:16	H01.00	4	Blefaritis	1	
2	26/04/2019 16:20	H16.9	1	Queratitis	1	
1	26/04/2019 16:31	H11.3	0	Hiposfagma	1	
1	26/04/2019 17:01	H00.02	4	Orzuelo	1	
1	26/04/2019 17:06	H16.9	1	Queratitis	1	
2	26/04/2019 17:20	H10.3	0	Conjuntivitis	1	
2	26/04/2019 17:46	H26.9	2	Catarata	1	
2	26/04/2019 18:10	H16.9	1	Queratitis	1	
2	26/04/2019 18:42	H11.3	0	Hiposfagma	1	
2	26/04/2019 19:15	H16.9	1	Queratitis	1	
1	26/04/2019 19:32	H11.3	0	Hiposfagma	1	
1	26/04/2019 20:11	T15.0	1	Cuerpo extraño	1	
2	26/04/2019 20:32	H16.9	1	Queratitis	1	
1	26/04/2019 21:07	T15.0	1	Cuerpo extraño	1	
2	26/04/2019 21:26	H00.02	4	Orzuelo	1	
2	26/04/2019 22:18	H11.3	0	Hiposfagma	1	
2	26/04/2019 23:16	H10.3	0	Conjuntivitis	1	
1	27/04/2019 0:29	H43.81	7	DVP	1	
1	27/04/2019 2:11	H53	11	No patología	1	
1	27/04/2019 4:26	T15.0	1	Cuerpo extraño	1	
2	27/04/2019 8:16	H15.1	6	Epiescleritis	1	
1	27/04/2019 8:49	T15.0	1	Cuerpo extraño	1	
2	27/04/2019 9:02	H16.0	1	Úlcera corneal	1	
1	27/04/2019 9:13	H10.3	0	Conjuntivitis	1	
1	27/04/2019 9:40	H26.9	2	Post op catarata	1	
1	27/04/2019 9:50	T15.0	1	Cuerpo extraño	1	
1	27/04/2019 10:04	H01.00	4	Blefaritis	1	
1	27/04/2019 10:11	H10.3	0	Conjuntivitis	1	
1	27/04/2019 10:23	H26.9	2	Post op catarata	1	
2	27/04/2019 10:27	H04.32	5	Dacriocistitis aguda	1	
1	27/04/2019 10:35	T15.0	1	Cuerpo extraño	1	
1	27/04/2019 10:59	H16.0	1	Úlcera corneal	1	
1	27/04/2019 11:06	H01.00	4	Blefaritis	1	
2	27/04/2019 11:21	H10.3	0	Conjuntivitis	1	
1	27/04/2019 11:25	H10.3	0	Conjuntivitis	1	
2	27/04/2019 11:28	H20	6	Uveitis	1	
2	27/04/2019 11:54	H43.81	7	DVP	1	
2	27/04/2019 11:56	H16.9	1	Queratitis	1	
2	27/04/2019 11:57	H10.3	0	Conjuntivitis	1	
1	27/04/2019 12:13	H16.0	1	Úlcera corneal	1	
2	27/04/2019 12:23	H04.32	5	Dacriocistitis aguda	1	
1	27/04/2019 12:35	H16.0	1	Úlcera corneal	1	
1	27/04/2019 12:50	H10.3	0	Conjuntivitis	1	
2	27/04/2019 12:58	H20	6	Uveitis	1	
2	27/04/2019 13:07	H10.3	0	Conjuntivitis	1	
1	27/04/2019 13:15	H10.3	0	Conjuntivitis	1	
1	27/04/2019 13:19	H10.3	0	Conjuntivitis	1	
2	27/04/2019 13:20	H10.3	0	Conjuntivitis	1	

2	27/04/2019 13:24	H10.3	0	Conjuntivitis	1	
2	27/04/2019 13:28	H16.9	1	Queratitis	1	
1	27/04/2019 13:42	T15.0	1	Cuerpo extraño	1	
2	27/04/2019 14:04	H15.1	6	Epiescleritis	1	
2	27/04/2019 14:38	H16.9	1	Queratitis	1	
1	27/04/2019 14:39	H16.0	1	Úlcera corneal	1	
2	27/04/2019 15:04	H16.9	1	Queratitis	1	
1	27/04/2019 15:23	T15.0	1	Cuerpo extraño	1	
2	27/04/2019 15:25	H10.3	0	Conjuntivitis	1	
2	27/04/2019 15:40	H16.9	1	Queratitis	1	
1	27/04/2019 15:43	H10.3	0	Conjuntivitis	1	
2	27/04/2019 16:13	H43.81	7	DVP	1	
2	27/04/2019 16:51	H43.81	7	DVP	1	
2	27/04/2019 17:13	H10.3	0	Conjuntivitis	1	
2	27/04/2019 17:47	H16.0	1	Úlcera corneal	1	
2	27/04/2019 17:58	T15.0	1	Cuerpo extraño	1	
2	27/04/2019 19:02	H10.3	0	Conjuntivitis	1	
2	27/04/2019 19:39	H10.3	0	Conjuntivitis	1	
1	27/04/2019 19:48	H53.10	10	Problema refractivo	1	
2	27/04/2019 20:08	H59.88	8	Post op retina	1	
2	27/04/2019 20:46	E11.319	8	Retinopatía diabética	1	
2	27/04/2019 20:49	H00.02	4	Orzuelo	1	
2	27/04/2019 20:56	H01.00	4	Blefaritis	1	
2	27/04/2019 20:58	H10.3	0	Conjuntivitis	1	
2	27/04/2019 21:09	H10.3	0	Conjuntivitis	1	
2	28/04/2019 2:12	T15.0	1	Cuerpo extraño	1	
1	28/04/2019 5:14	H16.9	1	Queratitis	1	
1	28/04/2019 10:12	H11.3	0	Hiposfagma	1	
2	28/04/2019 10:50	H16.9	1	Queratitis	1	
1	28/04/2019 11:20	T15.0	1	Cuerpo extraño	1	
2	28/04/2019 11:42	H01.00	4	Blefaritis	1	
2	28/04/2019 12:02	H20	6	Uveitis	1	
1	28/04/2019 12:21	H01.00	4	Blefaritis	1	
2	28/04/2019 12:24	H01.00	4	Blefaritis	1	
1	28/04/2019 12:27	T15.0	1	Cuerpo extraño	1	
2	28/04/2019 13:00	H16.0	1	Úlcera corneal	1	
2	28/04/2019 13:03	H10.3	0	Conjuntivitis	1	
1	28/04/2019 13:03	S05.9	9	Traumatismo	1	
1	28/04/2019 13:05	H16.0	0	Úlcera corneal	1	
1	28/04/2019 13:13	H10.3	0	Conjuntivitis	1	
2	28/04/2019 13:42	H10.3	0	Conjuntivitis	1	
1	28/04/2019 14:08	H10.3	0	Conjuntivitis	1	
2	28/04/2019 14:24	H00.02	4	Orzuelo	1	
2	28/04/2019 14:26	H35.3	8	Maculopatía	1	
1	28/04/2019 14:28	H00.02	4	Orzuelo	1	
1	28/04/2019 14:55	H10.3	1	Conjuntivitis	1	
2	28/04/2019 14:56	H16.0	1	Úlcera corneal	1	
1	28/04/2019 15:02	H10.3	0	Conjuntivitis	1	
1	28/04/2019 16:23	H40.9	3	Glaucoma agudo	1	
2	28/04/2019 16:55	H16.9	1	Queratitis	1	
1	28/04/2019 16:57	H16.9	1	Queratitis	1	
2	28/04/2019 16:59	H11.44	0	Quiste conjuntival	1	
2	28/04/2019 17:24	H10.3	0	Conjuntivitis	1	
2	28/04/2019 17:29	H16.0	1	Úlcera corneal	1	
1	28/04/2019 17:57	H10.3	0	Conjuntivitis	1	
2	28/04/2019 18:30	H11.3	0	Hiposfagma	1	
2	28/04/2019 19:30	H10.3	0	Conjuntivitis	1	
2	28/04/2019 20:01	H16.9	1	Queratitis	1	
1	28/04/2019 20:12	H10.3	0	Conjuntivitis	1	
2	28/04/2019 20:14	Alta por fuga	11	Alta por fuga	1	
1	28/04/2019 20:22	T15.0	1	Cuerpo extraño	1	
1	28/04/2019 21:04	S05.9	9	Traumatismo	1	

1	28/04/2019 21:09	T15.0	1	Cuerpo extraño	1	
1	28/04/2019 22:19	H16.0	1	Úlcera corneal	1	
1	28/04/2019 22:59	H16.9	1	Queratitis	1	
1	28/04/2019 23:46	H16.9	1	Queratitis	1	
1	29/04/2019 5:06	H16.0	1	Úlcera corneal	1	
2	29/04/2019 5:32	H10.3	0	Conjuntivitis	1	
1	29/04/2019 9:07	H16.0	1	Úlcera corneal	1	
1	29/04/2019 9:10	H11.44	0	Quiste conjuntival	1	
2	29/04/2019 9:20	H20	6	Uveitis	1	
1	29/04/2019 9:21	H10.3	0	Conjuntivitis	1	
2	29/04/2019 10:07	H16.9	1	Queratitis	1	
2	29/04/2019 10:14	H01.00	4	Blefaritis	1	
2	29/04/2019 10:29	H11.44	0	Quiste conjuntival	1	
2	29/04/2019 10:49	H10.3	0	Conjuntivitis	1	
1	29/04/2019 10:54	H11.3	0	Hiposfagma	1	
2	29/04/2019 11:02	H16.9	1	Queratitis	1	
2	29/04/2019 11:09	H10.3	0	Conjuntivitis	1	
2	29/04/2019 11:11	H20	6	Uveitis	1	
1	29/04/2019 11:12	H10.3	0	Conjuntivitis	1	
2	29/04/2019 11:52	H00.03	4	Celulitis preseptal	1	
2	29/04/2019 12:22	H16.0	1	Úlcera corneal	1	
2	29/04/2019 12:26	H10.3	0	Conjuntivitis	1	
2	29/04/2019 12:29	H10.3	0	Conjuntivitis	1	
1	29/04/2019 12:33	H11.3	0	Hiposfagma	1	
2	29/04/2019 12:47	H01.00	4	Blefaritis	1	
1	29/04/2019 13:06	H20	6	Uveitis	1	
2	29/04/2019 13:13	H43.81	7	DVP	1	
1	29/04/2019 13:31	H16.9	1	Queratitis	1	
1	29/04/2019 13:51	S05.9	9	Avulsión traumática	1	
2	29/04/2019 13:59	H10.3	0	Conjuntivitis	1	
1	29/04/2019 14:09	H43.81	7	DVP	1	
2	29/04/2019 14:25	H01.00	4	Blefaritis	1	
1	29/04/2019 14:28	H10.3	0	Conjuntivitis	1	
1	29/04/2019 14:54	H10.3	0	Conjuntivitis	1	
1	29/04/2019 15:32	H43.81	7	DVP	1	
1	29/04/2019 15:49	H16.9	1	Queratitis	1	
1	29/04/2019 16:24	H20	6	Uveitis	1	
2	29/04/2019 16:54	H02.05	4	Distiquiasis	1	
2	29/04/2019 16:59	H10.3	0	Conjuntivitis	1	
1	29/04/2019 17:11	E11.319	8	Retinopatía diabética	1	
1	29/04/2019 17:33	H02	4	Lesión palpebral	1	
2	29/04/2019 17:44	H16.9	1	Queratitis	1	
2	29/04/2019 17:49	H01.00	4	Blefaritis	1	
2	29/04/2019 18:06	H27.8	2	OCP	1	
1	29/04/2019 18:35	B00.1	11	Dermatitis herpética	1	
1	29/04/2019 19:51	H59.3	5	Postop via lagrimal	1	
1	29/04/2019 20:30	H16.0	1	Úlcera corneal	1	
1	29/04/2019 20:40	H10.3	0	Conjuntivitis	1	
2	29/04/2019 20:50	H02.05	4	Distiquiasis	1	
2	29/04/2019 21:03	H11.3	0	Hiposfagma	1	
2	29/04/2019 21:08	H10.3	0	Conjuntivitis	1	
1	29/04/2019 21:23	H10.3	0	Conjuntivitis	1	
1	29/04/2019 21:32	H10.3	0	Conjuntivitis	1	
1	29/04/2019 22:04	T15.0	1	Cuerpo extraño	1	
1	29/04/2019 22:37	H16.9	1	Queratitis	1	
1	30/04/2019 0:13	H02	4	Alteración palpebral	1	
2	30/04/2019 1:02	H10.3	0	Conjuntivitis	1	
1	30/04/2019 2:49	H00.02	4	Orzuelo	1	
2	30/04/2019 9:23	H10.3	0	Conjuntivitis	1	
1	30/04/2019 9:23	H10.3	0	Conjuntivitis	1	
2	30/04/2019 9:25	H10.3	0	Conjuntivitis	1	
1	30/04/2019 9:25	H46	10	Neuritis óptica	1	

2	30/04/2019 9:29	S05.9	9	Traumatismo	1	
2	30/04/2019 9:45	H10.3	0	Conjuntivitis	1	
2	30/04/2019 9:49	H20	6	Uveitis	1	
1	30/04/2019 9:55	S05.9	9	Traumatismo	1	
2	30/04/2019 10:10	Alta por fuga	11	Alta por fuga	1	
1	30/04/2019 10:31	H35.30	8	DMAE	1	
2	30/04/2019 10:37	H16.9	1	Queratitis	1	
1	30/04/2019 11:11	H00.02	4	Orzuelo	1	
2	30/04/2019 11:34	H10.3	0	Conjuntivitis	1	
1	30/04/2019 11:39	H20	6	Uveitis	1	
2	30/04/2019 11:43	H10.3	0	Conjuntivitis	1	
2	30/04/2019 12:12	H01.00	4	Blefaritis	1	
1	30/04/2019 12:24	H43.1	8	Hemovítreo	1	
1	30/04/2019 12:29	H15.1	6	Epiescleritis	1	
1	30/04/2019 12:40	T15.0	1	Cuerpo extraño	1	
2	30/04/2019 12:47	H16.9	1	Queratitis	1	
2	30/04/2019 12:51	H43.81	7	DVP	1	
2	30/04/2019 13:39	H16.9	1	Queratitis	1	
2	30/04/2019 14:47	H10.3	0	Conjuntivitis	1	
2	30/04/2019 15:43	H20	6	Uveitis	1	
2	30/04/2019 16:28	H16.9	1	Queratitis	1	
2	30/04/2019 16:44	T15.0	1	Cuerpo extraño	1	
2	30/04/2019 17:01	H16.9	1	Queratitis	1	
1	30/04/2019 17:03	H01.00	4	Blefaritis	1	
1	30/04/2019 17:18	S05.9	9	Traumatismo	1	
2	30/04/2019 17:21	H10.3	0	Conjuntivitis	1	
2	30/04/2019 18:27	H10.3	0	Conjuntivitis	1	
1	30/04/2019 18:28	H10.3	0	Conjuntivitis	1	
2	30/04/2019 18:49	H00.02	4	Orzuelo	1	
1	30/04/2019 18:50	H43.81	7	DVP	1	
1	30/04/2019 18:53	H53	11	No patología	1	
2	30/04/2019 19:05	H01.00	4	Blefaritis	1	
1	30/04/2019 19:27	Alta por fuga	11	Alta por fuga	1	
1	30/04/2019 19:37	T15.0	1	Cuerpo extraño	1	
1	30/04/2019 20:00	S05.9	9	Traumatismo	1	
1	30/04/2019 20:13	S05.30	0	Laceración conjuntival	1	
2	30/04/2019 20:26	H20	6	Uveitis	1	
2	30/04/2019 20:28	H02	4	Alteración palpebral	1	
2	30/04/2019 20:32	H43.81	7	DVP	1	
2	30/04/2019 20:48	H15.1	6	Epiescleritis	1	
1	30/04/2019 20:51	H10.3	0	Conjuntivitis	1	
1	30/04/2019 21:17	G43.9	10	Migraña	1	
1	30/04/2019 21:20	H10.3	0	Conjuntivitis	1	
2	30/04/2019 21:21	H16.9	1	Queratitis	1	
1	30/04/2019 23:32	H10.3	0	Conjuntivitis	1	
2	30/04/2019 23:39	H10.3	0	Conjuntivitis	1	
2	30/04/2019 23:42	H11.3	0	Hiposfagma	1	
1	30/04/2019 23:57	S05.9	9	Traumatismo	1	

COVID	Varón		Mujer		TOTAL	
	Recuento	Promedio de EDAD	Recuento	Promedio de EDAD	Recuento	Promedio de EDAD
**2020**						
mar	452	64,22	347	63,34	799	63,84
abr	925	63,92	766	65,14	1691	64,47
may	152	66,43	169	68,48	321	67,51
jun	56	65,63	35	68,25	91	66,64
jul	57	47,34	40	40,67	97	44,59
ago	216	47,54	188	44,69	404	46,22
sep	515	55,83	439	56,76	954	56,26
oct	402	60,72	365	60,77	767	60,74
nov	244	60,68	206	63,5	450	61,97
dic	187	59,19	161	59,74	348	59,44
**2021**						
ene	273	60,04	214	62,41	487	61,08
feb	360	61,92	292	64,12	652	62,91
mar	222	62,71	182	66,45	404	64,4
abr	223	57,01	211	59,84	434	58,39
may	204	53,05	155	55,58	359	54,14
jun	49	56,57	44	51,16	93	54,01
**Total general**	**4537**	**60,14**	**3814**	**61,18**	**8351**	**60,61**

**2020**	N
mar	799
apr	1691
may	321
jun	91
jul	97
aug	404
sep	954
oct	767
nov	450
dec	348
jan	487
feb	652

URGENCIAS ATENDIDAS POR FECHA DE ALTA DE OFTALMOLOGÍA													
año	ene	feb	mar	abr	may	jun	jul	ago	sep	oct	nov	dic	total
2015	1136	1119	1287	1232	1434	1458	1203	1026	1193	1228	1167	1167	14650
2016	1130	1175	1225	1337	1369	1356	1184	1006	992	1050	1082	1081	13987
2017	946	863	1112	928	1571	1512	1442	1284	1364	1390	1343	1431	15186
2018	1292	1274	1423	1417	1469	1440	1452	1288	1378	1472	1446	1498	16849
2019	1496	1449	1681	1480	1577	1527	1536	1354	1414	1463	1393	1372	17742
2020	1476	1381	501	302	807	1030	989	889	761	801	838	988	10763
promedio	1200	1176	1261,75	1228,5	1460,75	1441,5	1320,25	1151	1231,75	1285	1259,5	1294,25	15310,25
no cov	1479	1378	1679	1481	1575	1528	1535	1354	1414	1465	1391	1370	17649
covid	674	843	499	302	808	1033	987	888	760	801	839	985	9419

1200	1176	1231,75	1285	1259,5	1294,25	7446,5
1479	1378	1414	1465	1391	1370	8497

	meses	otros meses	
previos	7446,5	7863,75	15310,25
2019	8497	9152	17649
	15943,5	17015,75	
